# Enantioselective S−H Insertion Reactions of α‐Carbonyl Sulfoxonium Ylides

**DOI:** 10.1002/anie.202005563

**Published:** 2020-05-28

**Authors:** Patrícia B. Momo, Alexandria N. Leveille, Elliot H. E. Farrar, Matthew N. Grayson, Anita E. Mattson, Antonio C. B. Burtoloso

**Affiliations:** ^1^ Institute of Chemistry of São Carlos University of São Paulo CEP 13560-970 São Carlos SP Brazil; ^2^ Department Chemistry and Biochemistry Worcester Polytechnic Institute 100 Institute Road Worcester MA 01609 USA; ^3^ Department of Chemistry University of Bath Claverton Down Bath BA2 7AY UK

**Keywords:** aryl thiol, enantioselectivity, organocatalysis, S−H insertion, sulfoxonium ylide

## Abstract

The first example of enantioselective S−H insertion reactions of sulfoxonium ylides is reported. Under the influence of thiourea catalysis, excellent levels of enantiocontrol (up to 95 % *ee*) and yields (up to 97 %) are achieved for 31 examples in S−H insertion reactions of aryl thiols and α‐carbonyl sulfoxonium ylides.

## Graphical abstract

**The first enantioselective** S−H insertion reaction of α‐carbonyl sulfoxonium ylides was developed under thiourea catalysis. Up to 95 % *ee* and 97 % isolated yields were obtained in 31 examples.

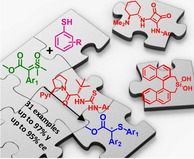

Sulfoxonium ylides are powerful reagents for a variety of reactivity patterns, including insertion reactions.^1^, ^2^, ^3^, ^4^ Owing to their stability, long shelf life, and ease of use, sulfoxonium ylides may enable the safer execution of insertion chemistry when compared to more traditionally employed α‐diazocarbonyls. Industrial applications are of particular relevance as Mangion and co‐workers from Merck noted that sulfoxonium ylides may be more suitable for large scale work as they do not lead to the production of gas or rapid exotherms.^5^ Further adding to their allure, recent methodologies developed separately in the Burtoloso and Aissa laboratories enable direct access to more decorated sulfoxonium ylides from safe commercially available starting materials in a single step.^6^, ^7^, ^8^

Due to their promising synthetic profile, the application of sulfoxonium ylides in insertion chemistry has been gaining traction in the past decade. To date, efforts have typically focused on transition metal catalyzed insertion chemistry of sulfoxonium ylides (Scheme 1 A). Select transition metal catalyst systems (e.g., [Ir(cod)Cl]_2_, [Pt(cod)Cl]_2_, [AuCl(SMe_2_)]) enable non‐enantioselective N−H, S−H, and O−H insertion reactions of a range of substrates in high yield.^5^, ^9^, ^10^, ^11^, ^12^, ^13^ To the best of our knowledge, excluding the contributions employing diazocarbonyls,^14^ no examples of enantioselective insertion reactions of sulfoxonium ylides have been reported.

**Scheme 1:**
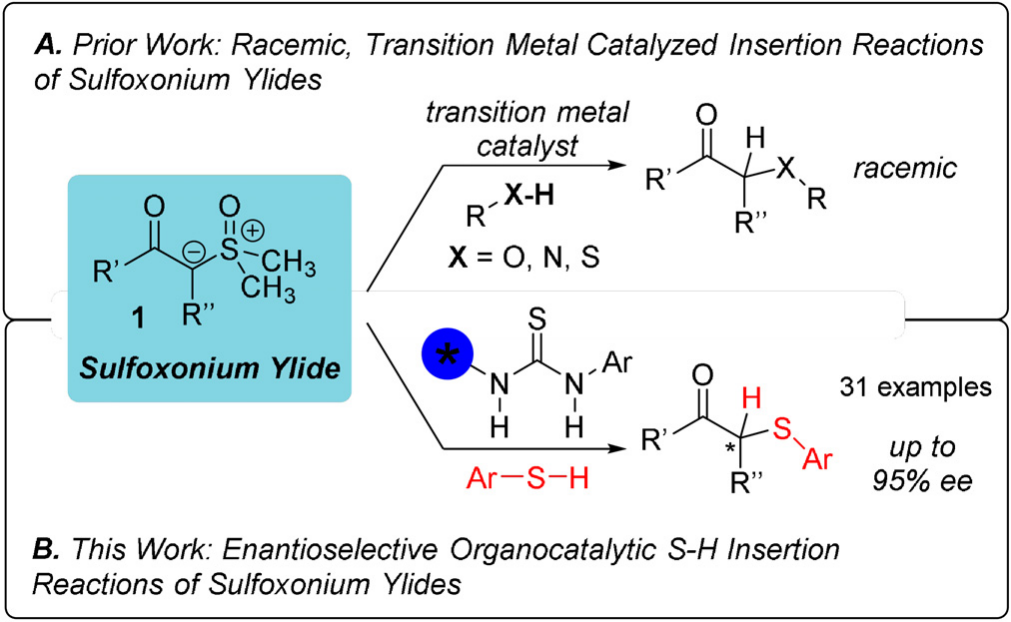
X−H Insertion reactions from sulfoxonium ylides.

Inspired by the unrealized potential of enantioselective insertion reactions of sulfoxonium ylides, we initiated investigations to advance a metal‐free approach to control the enantioselectivity of sulfoxonium ylide insertion reactions into polar X−H bonds (X=S, N, O). As a starting point for exploration, we reasoned that our recently identified catalyst‐free S−H bond insertion reactions^15^ could be coupled with dual hydrogen bond donor catalysts, like silanediols,^16^, ^17^, ^18^, ^19^, ^20^ squaramides,^21^, ^22^, ^23^, ^24^, ^25^ or (thio)ureas,^26^, ^27^, ^28^, ^29^, ^30^ to effect the desired transformation with high levels of enantiocontrol. In this communication, we describe our successful development of the insertion reaction of sulfoxonium carbonyl compounds into aryl thiols with high levels of enantiocontrol under the influence of thiourea catalysis (Scheme 1 B).

The investigations began with the insertion of sulfoxonium ylide **1 a** into the S−H bond of thiophenol **2 a** to generate **3 a** in the presence of a variety (15 examples; see SI) of dual hydrogen bond donor catalysts, including thioureas, ureas, squaramides, and silanediols (Table 1, catalysts **4**–**8** as selected examples). Varying degrees of success were achieved with a variety of (thio)urea and squaramide catalysts. For instance, 10 mol % of thioureas **4 a** and **4 b**, advanced by the Jacobsen laboratory, delivered the desired product **3 a** in up to 50 % enantiomeric excess if the reaction was conducted in toluene at 23 °C.^31^ On the other hand, thiourea **5** was able to produce only racemic product **3 a** under the same reaction conditions.^32^, ^33^, ^34^ Squaramide **6** provided **3 a** in 47 % yield and 26 % enantiomeric excess.^35^ The BINOL‐based silanediols **7 a** and **7 b** were unable to control enantioselectivity of the insertion event.^36^ Quinine‐derived urea **8 a** and thiourea **8 b** furnished **3 a**, but with only low levels of enantiocontrol, 4 % and 12 % *ee*, respectively.^37^, ^38^, ^39^ Strong Bronsted acids such as (*S*)‐TRIP and (*S*)‐VAPOL were also evaluated (**9 a** and **9 b**), providing **3 a** in only 2 and 20 % *ee*, respectively (Table 1).

**Table 1:** Organocatalyst screening for the enantioselective S−H insertion. 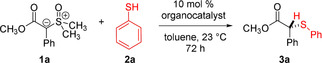

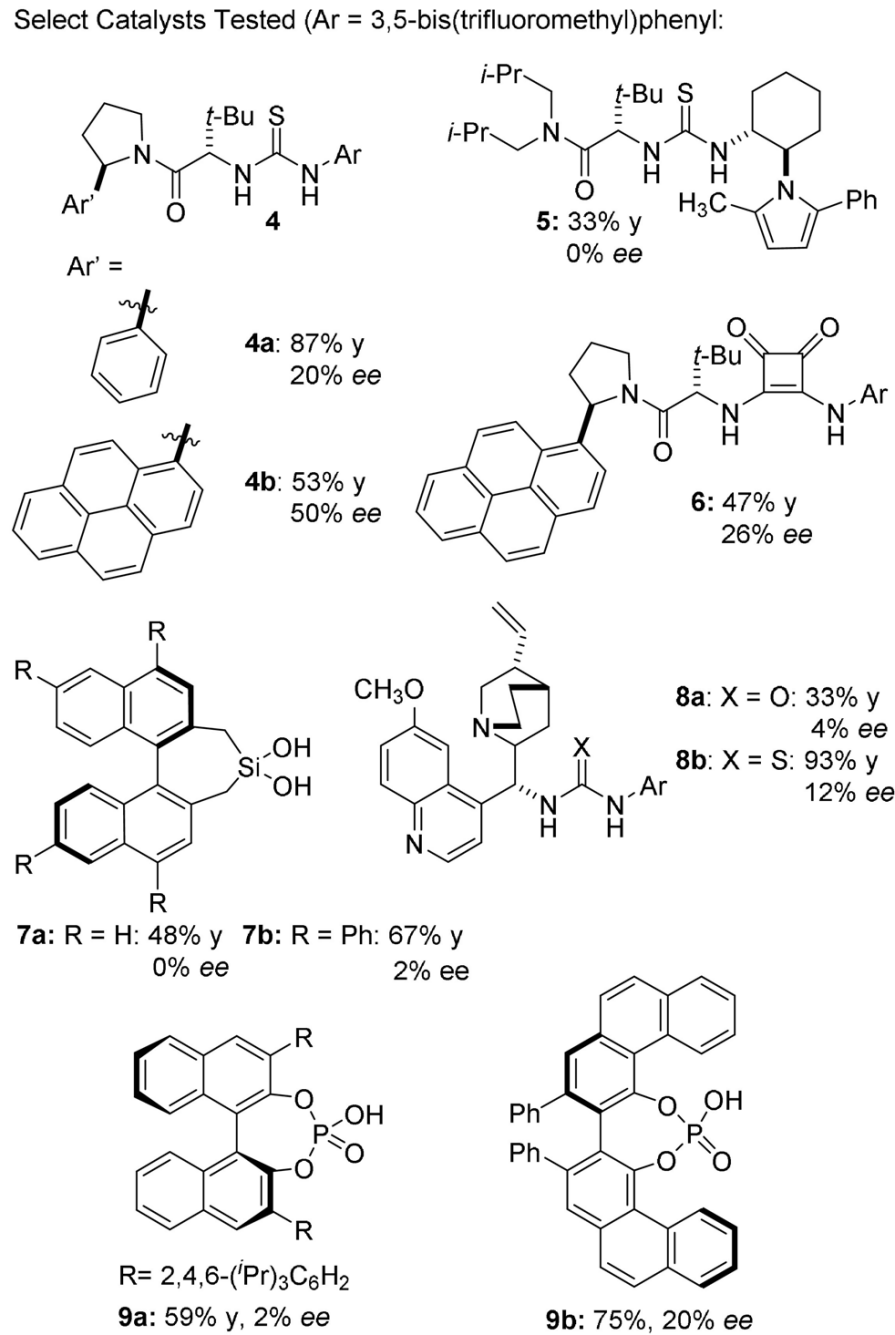

Encouraged by the promising levels of enantiocontrol in our initial screening, further optimization of the reaction parameters was pursued with thiourea catalyst **4 b** (Table 2). The nature of the solvent dramatically affected both the reaction rate and enantiomeric excess observed. While the original solvent employed, toluene, was able to give rise to **3 a** in 53 % yield and 50 % *ee* (entry 1), the ethereal solvents such as methyl *t*‐butyl ether (MTBE) produced **3 a** in 64 % enantiomeric excess (entry 2). Diethyl ether afforded **3 a** in just 24 % *ee* (entry 3). Halogenated solvents, including chloroform and dichloromethane, were the most advantageous in the reaction system producing high yields and good enantiomeric excess (74 % and 62 %, entries 4 and 5). A reduction in the reaction temperature, from 23 °C to −28 °C, provided further improvements in enantiomeric excess (85 % *ee*), and extended reaction times enabled achievement of high yields (entry 6). Further cooling of the reaction led to poor conversion after 7 days (entry 7). Finally, the effect of catalyst loading on the reaction platform was assessed (entries 8–10). The reduction of the catalyst loading from 10 mol % to 5 mol % resulted in only mild reductions in yield and stereocontrol (68 % yield, 74 % *ee*, entry 8), but a catalyst loading of only 1 mol % proved to be rather ineffective for this process (entry 9). Interestingly, doubling the amount of catalyst **4 b** to 20 mol % did not affect yield and enantioselectivity (entry 10). Although not depicted in the table (see SI), the effect of the concentration of the reaction on yield and stereocontrol was also tested but it was found to have a minimal effect in chloroform with 0.05 m to 0.5 m reactions providing nearly identical enantiomeric excesses (72–74 % *ee* in all cases, see Supporting Information for details). The absolute stereochemistry of **3 a** (*R*) was assigned by the comparison of its optical activity with literature values.^40^

**Table 2:** Optimization studies employing organocatalyst **4 b**. 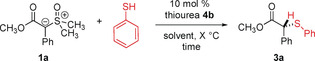

Entry^[a]^	Solvent	*T* [°C]	*t* [h]	Yield [%]	*ee* [%]
1	toluene	23	72	53	50
2	MTBE	23	96	74	64
3	Et_2_O	23	96	74	24
4	CHCl_3_	23	24	82	74
5	CH_2_Cl_2_	23	24	85	62
6	CHCl_3_	−28	96	87	85
7	CHCl_3_	−45	168	48	86
8^[b]^	CHCl_3_	−28	168	68	74
9^[c]^	CHCl_3_	−28	168	6	30
10^[d]^	CHCl_3_	−28	96	87	86

[a] 0.1 mmol scale; See Supporting Information for detailed experimental procedures. [b] 5 mol % catalyst loading. [c] 1 mol % catalyst loading. [d] 20 mol % catalyst loading.

With optimized reaction parameters identified, attention was directed toward the study of the effect of substrate structure on enantiocontrol and yield. First, the tolerance of the reaction toward S−H insertion reaction partners was put to the test (Table 3).

**Table 3:** Substrate scope based on aryl thiols and ylide **1 a**. 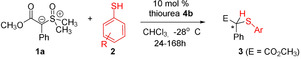

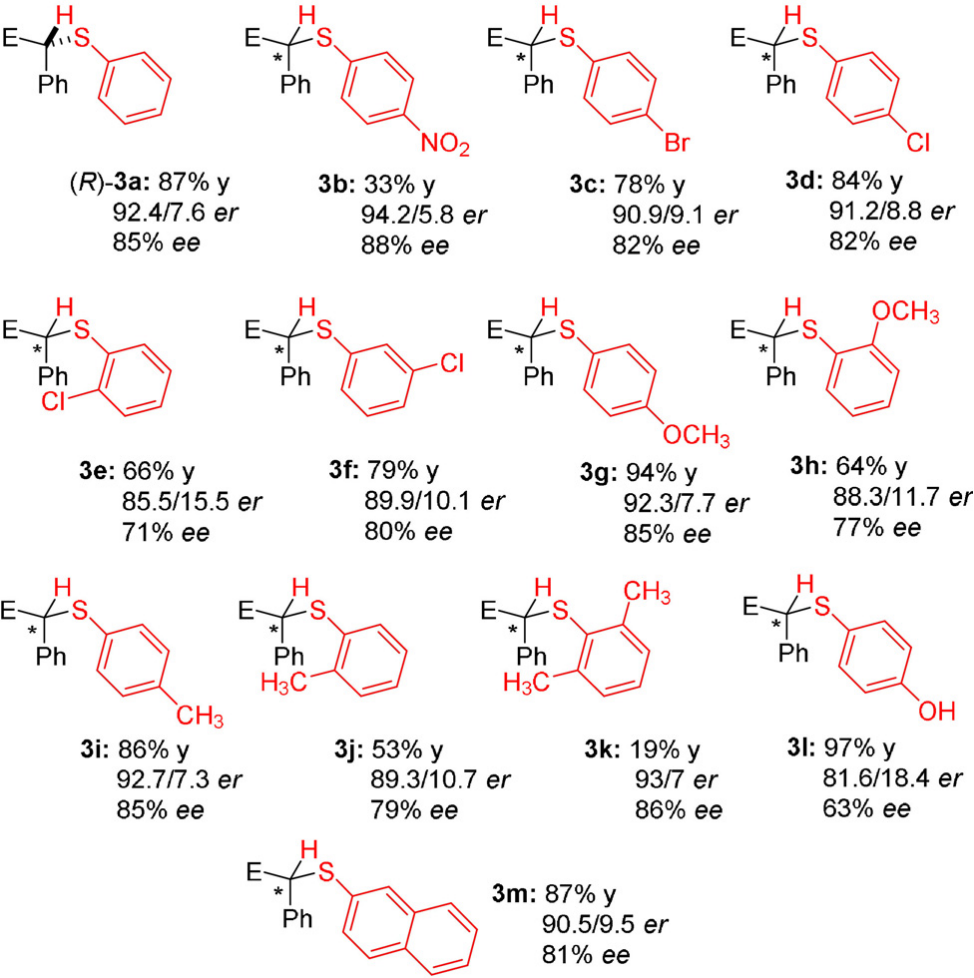

The reaction was able to accommodate a diverse set of aryl thiols. Electron withdrawing group in the *para* position of thiophenol, including nitro, bromo, and chloro, gave rise to product **3 b**–**3 d** with excellent levels of enantiocontrol and good yields, except for the nitro group. *o*‐Chloro thiophenol gave rise to **3 e** in 66 % yield and 71 % *ee* while *m*‐chlorothiophenol gave rise to **3 f** in 79 % yield and 80 % *ee*. Electron donating methoxy groups on the thiophenol, such as *p*‐ and *o*‐methoxy, gave rise to the corresponding compounds **3 g** and **3 h** in high yield and 85 % *ee* and 77 % *ee*, respectively. Methyl groups were also well tolerated giving rise to **3 i**–**3 k** with high levels of stereocontrol. Notably, the sterically encumbered product **3 k** was formed in 86 % *ee*, albeit in low yield. The reaction occurred in the presence of an unprotected phenol, affording **3 l** in 97 % yield and 63 % *ee*. 2‐Naphthalenethiol reacted to give **3 m** in 87 % yield and 81 % *ee*. All attempts to conduct the reaction with an aliphatic thiol S−H insertion reaction partner resulted in no reaction, with all starting materials remaining. The influence of the structure of the sulfoxonium ylide on the thiourea‐catalyzed insertion reaction of a variety of aryl thiols was probed next (Table 4).

**Table 4:** Substrate scope based on both sulfoxonium ylides and aryl thiols. 
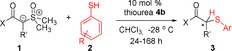

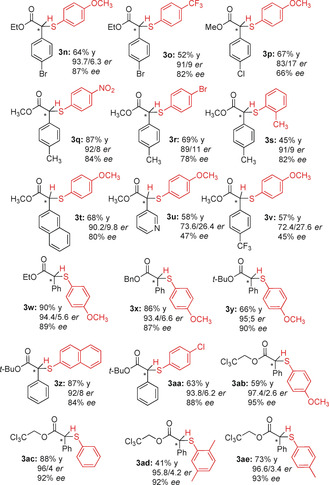

Halogen substituents in the *para*‐position of the aryl group on the sulfoxonium ylides were well tolerated in the reaction, giving rise to products **3 n**–**p** in good yields and 66–87 % *ee*. The introduction of a methyl group to the *para* position of the phenyl sulfoxonium ylide gave rise products **3 q**–**s** in good yield and high enantiomeric excess (84 % *ee*, 78 % *ee*, 82 % *ee*, respectively). The reaction of sulfoxonium ylide **1** (X=OCH_3_, R′=2‐naphthyl) with 4‐methoxybenzenethiol gave rise to **3 t** in 68 % yield and 80 % *ee*. Select heterocycles can be accommodated on the sulfoxonium ylide. For example, the reaction of **1** (X=OCH_3_, R′=pyridine) with 4‐methoxylbenzenethiol afforded **3 u** in 58 % yield and 47 % *ee*. A *para*‐trifluormethyl group was also accommodated well in the process, giving rise to **3 v** in 57 % yield and 45 % *ee*.

Next, we observed that the enantiomeric excess of the insertion reaction could be influenced by the type of ester on the sulfoxonium ylide **1**. For instance, the methyl ester gave rise to **3 g** in 94 % yield and 80 % *ee* while the improved enantiomeric excess was observed when the ethyl ester was employed giving rise to **3 w** in 90 % yield and 89 % *ee*. Benzyl ester **1** (R′=Ph) gave rise to **3 x** in 86 % yield and 87 % *ee*. An even better enantiomeric excess resulted from insertion reactions of the *t*‐butyl ester, compound **3 y** being isolated in 66 % yield and 90 % *ee*. Additional *t*‐butyl sulfoxonium ylides also gave rise to products with high levels of enantiocontrol, but not in the same range. For example, **3 z** and **3 aa** were isolated in 84 % and 88 % *ee*, respectively. Finally, the highest enantiomeric excess resulted from insertion reactions of the 2,2,2‐trichloroethyl esters: **3 ab**–**3 ae** were isolated in 95 % *ee*, 92 % *ee*, 92 % *ee* and 93 % *ee*, respectively.

To understand the mechanism of this new mode of enantioselective insertion, as well as the stereochemical outcome of the products, we decided to perform some NMR studies and DFT calculations. The interaction of the thiourea **4 b** and the sulfoxonium ylide **1 a** was first studied by ^1^H NMR spectroscopy (see spectra in SI). In this set of titration experiments, aliquots of **1 a** were added to a 10 mm solution of the thiourea in CDCl_3_. The two N−H protons of the thiourea are visible around 9 ppm in the ^1^H NMR before any sulfoxonium ylide is introduced. As aliquots of **1 a** are added to **4 b**, there are clear shifts in the N−H protons which may indicate that the sulfoxonium ylide is interacting with the thiourea. On the other hand, the same titration experiments conducted with **4 b** and the thiophenol resulted in no change to the NMR spectra of **4 b** or the thiophenol.

From these studies, and mechanistic insights already published for the non‐enantioselective version (see ref. 15), our current working hypothesis for the reaction pathway is depicted in Scheme 2 A. We propose that the thiourea catalyst **4 b** may hydrogen bond to the sulfoxonium ylide to generate complex such as complex **4 b**:**1 a**. The hydrogen bond complex **4 b**:**1 a** may then deprotonate the thiophenol (rate‐determining step^15^) to give rise to the corresponding ion pair **10**. The thiolate may then participate in a substitution reaction to give rise to the product **3** while simultaneously releasing the thiourea back into the catalytic cycle. Subsequent DFT investigations (Gaussian16 (Revision A.03),^41^ see SI) allowed modelling of the selectivity‐determining protonation step from **4 b:1 a** to **10** in order to determine the origins of selectivity.^42^ These calculations were performed on the optimised conditions in Table 2 (entry 6, 85 % *ee*), however the trifluoromethyl and pyrene groups of the catalyst were truncated to fluorines and phenyl, respectively, to reduce computational expense. Superimposition of the lowest energy conformations of catalyst **4 b** and the model catalyst (RMSD=0.095 over core atoms, see SI) indicates that these truncations do not significantly impact the conformation of the catalyst, and hence allow reasonable approximation of the full transition structures (TSs). Overall, calculations predicted a computed *ee* of 82 % based on a Boltzmann weighting at 245.15 K over all structures within 3 kcal mol^−1^ of the lowest in free energy. The lowest energy TSs leading to the major and minor enantiomers of the product, **TS‐1** and **TS‐2**, respectively, are depicted in Scheme 2 B. In agreement with the NMR results, interactions were observed between the thiourea catalyst and the sulfoxonium ylide, but not between the thiourea and thiophenol. In **TS‐1**, the ylide S=O forms hydrogen bonds with the two acidic protons of the thiourea, whilst a non‐classical C−H⋅⋅⋅O hydrogen bond is formed between the carbonyl oxygen and the 3,5‐bisfluorophenyl ring. In **TS‐2**, the roles of the carbonyl and the S=O groups are reversed. A weak C−H⋅⋅⋅O interaction exists in both structures between an ylide methyl and the carbonyl of the catalyst.

**Scheme 2:**
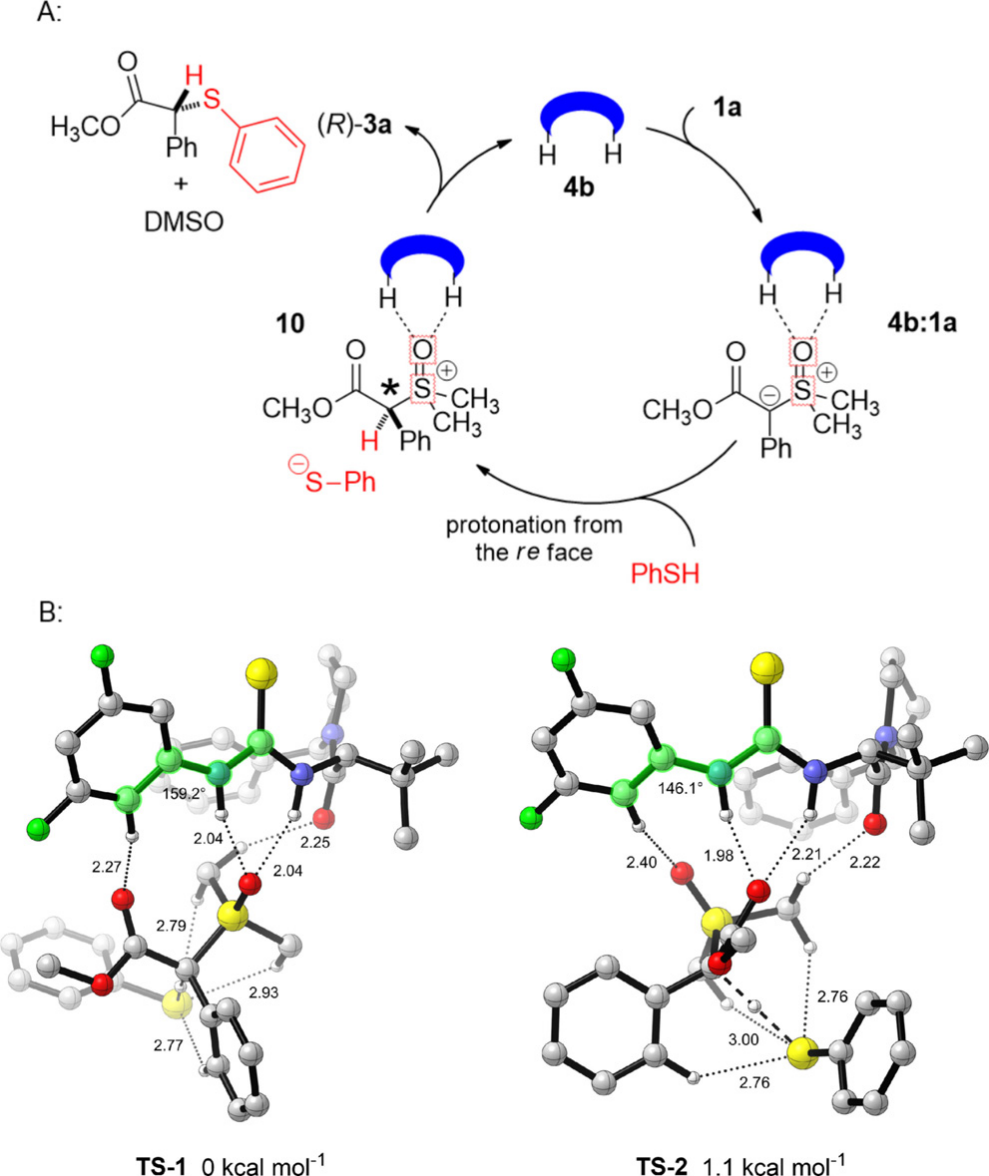
a) Plausible reaction pathway. b) Relative free energies of TS‐1 and TS‐2 (M06‐2X/def2tzvpp/IEFPCM(chloroform)//B3LYP/6‐31G(d)). Distances in angstroms [Å]. Highlighted atoms indicate measured dihedral angle.

Whilst the ylide orientation is parallel to the thiourea unit of the catalyst in **TS‐1**, the two species are approximately perpendicular in **TS‐2**. This avoids unfavorable steric interactions between the ylide ester group and the *t*‐butyl of the catalyst and allows the formation of the methyl C−H⋅⋅⋅O interaction with the catalyst carbonyl. However, as a result, the C−H⋅⋅⋅O interaction between the ylide and 3,5‐bisfluorophenyl ring in **TS‐2** is made longer and, according to NBO analysis, 0.7 kcal mol^−1^ weaker than in **TS‐1**. Additionally, the 3,5‐bisfluorophenyl ring is twisted slightly out of plane, inducing additional torsional strain relative to **TS‐1** (see highlighted dihedral angle). Due to their greater potential for steric interactions with the catalyst *t*‐butyl, larger ester groups on the ylide, such as *t*‐butyl and trichloroethyl, enhance both of these effects, raising the energy of **TS‐2** relative to **TS‐1** and improving selectivity. Whilst the ylide‐catalyst hydrogen bond distances do not depend significantly on the ylide orientation, NBO analysis revealed these interactions to be 2.0 kcal mol^−1^ stronger in **TS‐1** than in **TS‐2**, indicating that the S=O functionality is a better hydrogen bond acceptor than the carbonyl. The overall preference for the binding mode in **TS‐1** is a major factor in selectivity.

Additionally, non‐classical C−H⋅⋅⋅S hydrogen bonds were also found to be essential to selectivity. While three such interactions are formed between the ylide and thiophenol in **TS‐1** and **TS‐2**, only one interaction is formed when the thiophenol approaches the opposite face of the ylide, but keeping the same binding modes (see **TS‐1′** and **TS‐2′** in SI). Accordingly, such TSs are 3.8 kcal mol^−1^ and 3.4 kcal mol^−1^ higher in energy than **TS‐1** and **TS‐2**, respectively. NBO analysis of these C−H⋅⋅⋅S interactions in **TS‐1** revealed a combined strength 3.7 kcal mol^−1^ stronger than the single interaction formed by the alternative approach TS, accounting well for the overall 3.8 kcal mol^−1^ difference.

The synthetic utility of the S−H insertion products was put to the test and several of the results are depicted in Scheme 3. **3 a** was oxidized from the thioether to the sulfone using 3.2 equiv of *m*‐CPBA to give rise to **11** in 91 % yield with 80 % enantiomeric excess. The reduction of the **3 a** to alcohol **12** occurred in 85 % yield with retention of enantiomeric excess upon treatment with 3.0 equiv of lithium aluminum hydride in diethyl ether. The addition of methyl magnesium bromide gave rise to alcohol **13** in 48 % yield with 82 % enantiomeric excess. It is also worth to mention that compound **3 a** could be prepared in a larger scale (1 mmol) without any change in yield and enantiomeric ratio.

**Scheme 3:**
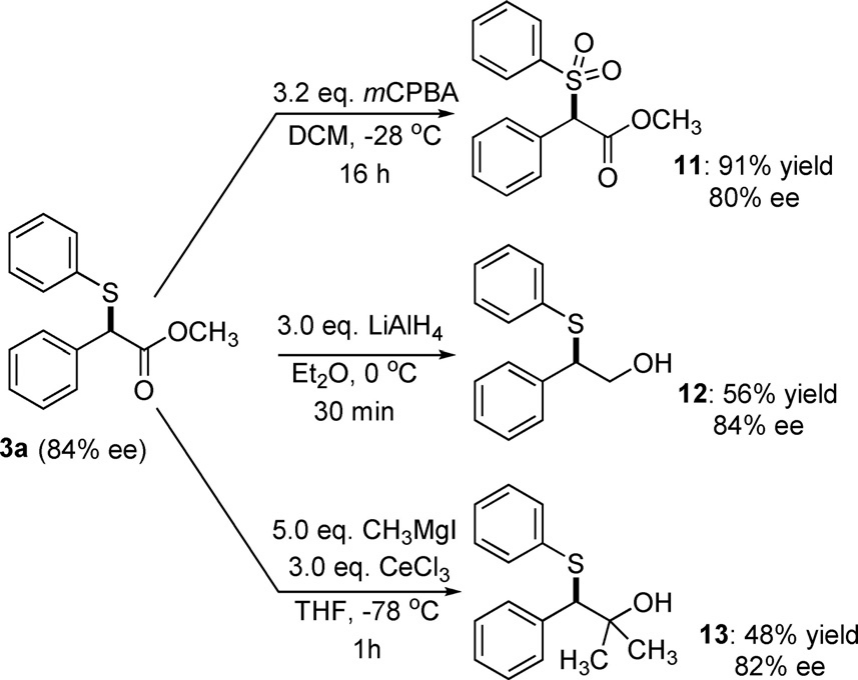
Synthetic manipulations of S−H insertion product **3 a**.

In summary, an enantioselective S‐H insertion reaction of α‐carbonyl sulfoxonium ylides is reported. The transformation relies on the influence of a known chiral thiourea catalyst to control the enantioselectivity of a diverse array of aryl thiols in several α‐carbonyl sulfoxonium ylide reaction partners. High yields and high levels of enantiocontrol are observed with a relatively broad substrate scope. Significant additional investigations on the mechanism indicate how classical and non‐classical hydrogen bonding interactions play key roles in determining selectivity. Current efforts are directed toward exploring other thiourea‐catalyzed insertion reactions of α‐carbonyl sulfoxonium ylides and our progress will be reported in due course.

## Conflict of interest

The authors declare no conflict of interest.

## Supporting information

As a service to our authors and readers, this journal provides supporting information supplied by the authors. Such materials are peer reviewed and may be re‐organized for online delivery, but are not copy‐edited or typeset. Technical support issues arising from supporting information (other than missing files) should be addressed to the authors.

Supplementary
