# Supplementary material for: Enantioselective S−H Insertion Reactions of α‐Carbonyl Sulfoxonium Ylides
Source: Angew Chem Int Ed Engl. 2020 May 28;59(36):15554–9. doi: 10.1002/anie.202005563 (PMC7606806; doi:10.1002/anie.202005563)
Supplement: Supplementary file 1 — Supplementary [file ANIE-59-15554-s001.pdf]

Angewandte Chemie
Eine Zeitschrift der Gesellschaft Deutscher Chemiker

Supporting Information

# Enantioselective S–H Insertion Reactions of α-Carbonyl Sulfoxonium Ylides

*Patrícia B. Momo, Alexandria N. Leveille, Elliot H. E. Farrar, Matthew N. Grayson, Anita E. Mattson,* and Antonio C. B. Burtoloso**

anie_202005563_sm_miscellaneous_information.pdf

**TABLE OF CONTENTS** **Page**


**1. General Information** *S2*

**2. Optimization of the Reaction Conditions** *S3*

2.1 Screening of the Ligands *S3*

2.2 Screening of the Solvents *S4*

**3. Sulfoxonium Ylides Synthesized** *S5*

**4. General Procedure for Asymmetric S-H Insertions of Sulfoxonium Ylides** *S8*

**5. General procedure for the titration of chiral catalyst 4b with 1a and 2a** *S 26*

**6. $^{1}H$ and $^{13}C$ NMR Spectra** *S28*

**7. HPLC spectra** *S72*

**8. Computational Details** *S107*

**9. References** *S401*

## 1. General Information

***Reaction Setup:*** Air- and moisture-sensitive reactions were conducted in flame- or oven-dried glassware equipped with tightly fitted rubber septa and under a positive pressure of dry argon. Reagents and solvents were handled by using standard syringe techniques. Unless stated otherwise, all the yields refer to isolated products after flash column chromatography.

***NMR Spectroscopy:*** $^1$H NMR spectra were acquired using a Bruker BioSpin 500MHz Avance III Digital NMR spectrometer and calibrated using the solvent signal ($CDCl_3$ 7.26 ppm). Multiplicities were determined using MNova software. $^{13}$C NMR spectra were acquired using a Bruker BioSpin 126MHz Avance III Digital NMR spectrometer and calibrated using the solvent signal ($CDCl_3$ 77.16 ppm). $^1$H NMR multiplicities are reported as follows: s = singlet; d = doublet; t = triplet; q = quartet; m = multiplet.

***Infrared Spectroscopy:*** IR spectra were measured on Bruker Vertex 70 with an ATR accessory.

***Mass Spectroscopy:*** High-Resolution mass spectra were acquired using an Agilent 6520 Q-TOF mass spectrometer.

***Melting Points:*** All melting points were measured Barnstead Electrotherm 1001D/1001 Mel-Temp Capillary Melting Point apparatus and are uncorrected.

***Optical Rotation:*** Optical rotations were acquired on a Jasco Digital Polarimeter with a 1 dm cell and a sodium lamp.

***HPLC:*** Enantiomeric excess was determined by high performance liquid chromatography (HPLC) using Agilent 1260 equip with a diode array detector.

***Solvents/chemicals:*** Ethyl acetate, hexanes, methanol, and chloroform were used as received. Acetonitrile and dimethyl sulfoxide were dried over 4Å molecular sieves prior to use. All other reagents were used directly as received from the manufacturer.

***Catalyst:*** Catalyst **4a**, **5** and **9** are commercially available, **4b**, **6**, **7** and **8** are known and were synthetized according to literature procedure.[1-4]

# 2. Optimization of the Reaction Conditions

## 2.1. Table S1 Screening of the Ligands

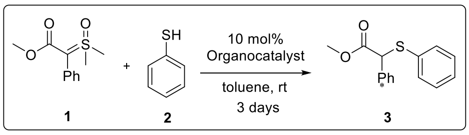


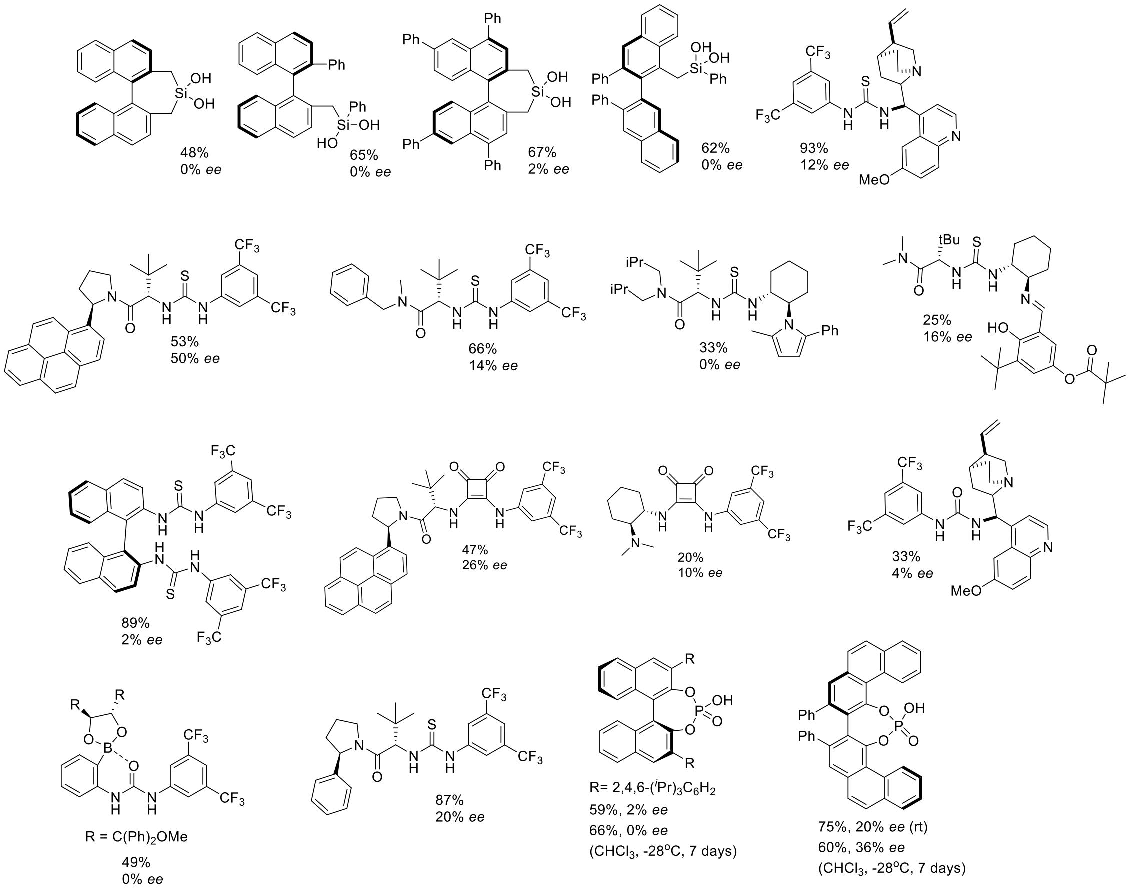

## 2.2. Table S2 Screening of the Solvents[a]

| Entry | Solvent | Time (h) | Yield (%) | *er* (%) | ee (%) |
|---|---|---|---|---|---|
| 1 | 1,4-dioxane | 48 | 86 | 75/25 | 50 |
| 2 | Toluene[b] | 72 | 53 | 75/25 | 50 |
| 3 | MTBE | 96 | 74 | 82/18 | 64 |
| 4 | Ether | 96 | 74 | 62/38 | 24 |
| 5 | $CHCl_3$ | 24 | 82 | 87/13 | 74 |
| 6 | EtOAc | 96 | 80 | 77/23 | 54 |
| 7 | THF | 96 | 81 | 66/34 | 32 |
| 8 | DCM | 24 | 85 | 81/19 | 62 |
| 9 | $PhCF_3$ | 96 | 92 | 55/45 | 10 |
| 10 | ACN | 24 | 85 | 67/33 | 34 |
| 11 | PhCl | 48 | 84 | 81/19 | 62 |
| 12 | 1.2-DCE | 4 | 79 | 87/13 | 74 |

[a] Catalyst **4b** was used for the solvent screening. [b]not all starting material was consumed

## 2.3. Table S3 Optimization of experimental conditions[a]

| Entry | Solvent | [ ](M) | T (°C) | t (h) | Yield (%) | *er* (%) | ee (%) |
|---|---|---|---|---|---|---|---|
| 1 | $CHCl_3$ | 0.5 | rt | 18 | 75 | 87/13 | 74 |
| 2 | $CHCl_3$ | 0.2 | rt | 24 | 82 | 87/13 | 74 |
| 3 | $CHCl_3$ | 0.1 | rt | 72 | 73 | 87/13 | 74 |
| 4 | $CHCl_3$ | 0.05 | rt | 72 | 87 | 86/14 | 72 |
| 5 | DCE | 0.5 | rt | 2.5 | 82 | 83/17 | 66 |
| 6 | DCE | 0.2 | rt | 4 | 79 | 87/13 | 74 |
| 7 | DCE | 0.1 | rt | 48 | 97 | 87/13 | 74 |
| 8 | DCE | 0.05 | rt | 48 | 80 | 85/25 | 70 |
| 9 | $CHCl_3$ | 0.2 | 5 | 72 | 75 | 89/11 | 78 |
| 10 | DCE | 0.2 | 5 | 72 | 82 | 89/11 | 78 |
| 11 | $CHCl_3$ | 0.2 | -28 | 168 | 85 | 92/8 | 84 |
| 12 | DCE | 0.2 | -28 | 168 | 58 | 92/8 | 84 |
| 13 | $CHCl_3$ | 0.5 | -28 | 96 | 87 | 92.4/7.6 | 85 |
| 14[b] | $CHCl_3$ | 0.5 | -45 | 168 | 48 | 93/7 | 86 |
| 15 | $CHCl_3$ | 1.0 | -28 | 96 | 80 | 77/23 | 54 |
| 16[c] | $CHCl_3$ | 0.5 | -28 | 72 | 89 | 93/7 | 86 |
| 17[b,d] | $CHCl_3$ | 0.5 | -28 | 168 | 68 | 87/13 | 74 |
| 13[b,e] | $CHCl_3$ | 0.5 | -28 | 168 | 6 | 65/35 | 30 |

[a]Catalyst **4b** (10 mol%) was used for the solvent, temperature, and concentration screening. [b]Reaction was stopped after 7 days; not all starting material was consumed. [c]Reaction performed with 20 mol% of **4b**. [d]Reaction performed with 5 mol% of **4b**. [e]Reaction performed with 1 mol% of **4b.**

## 3. Sulfoxonium Ylides Synthesized

Methyl 2-(dimethyl(oxo)-sulfaneylidene)-2-phenylacetate **1a**

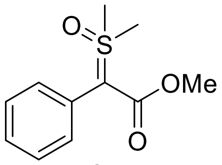

**1a**

Prepared according to the literature.[5] The spectroscopy data were in good agreement with the literature.[5] White solid (2.9 mmol scale, 531.0 mg, 82%). **Rf** = 0.46 (9:1 CHCl3:MeOH); **$^1$H NMR** (500 MHz, $CDCl_3$): δ 7.29 – 7.19 (m, 5H), 3.55 (s, 3H), 3.35 (s, 6H) ppm; **$^{13}$C NMR** (126 MHz, $CDCl_3$): δ 166.8, 133.9 (2C), 132.5, 128.6 (2C), 127.4, 70.3, 50.6, 43.3 (2C) ppm.

Ethyl 2-(dimethyl(oxo)-sulfaneylidene)-2-phenylacetate **1b**

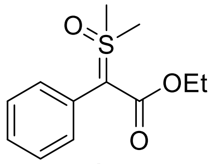

**1b**

Prepared according to the literature from ethyl 2-phenylethanoate.[5] The spectroscopy data were in good agreement with the literature.[6] White solid (1.7 mmol scale, 179.3 mg, 62%). **Rf** = 0.46 (95:5 EtOAc/MeOH); **$^1$H NMR** (500 MHz, $CDCl_3$) δ 7.24 – 7.22 (m, 4H), 7.20 – 7.14 (m, 1H), 4.04 (q, J = 7.1 Hz, 2H), 3.34 (s, 6H), 1.12 (t, J = 7.1 Hz, 3H); **$^{13}$C NMR** (126 MHz, $CDCl_3$) δ 166.5, 133.7 (2C), 132.8, 128.4 (2C), 127.0, 70.4; 59.00, 43.4 (2C), 14.9 ppm.

Benzyl 2-(dimethyl(oxo)-sulfaneylidene)-2-phenylacetate **1c**

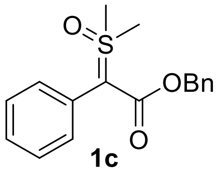

**1c**

Prepared according to literature procedure from benzyl 2-phenylacetate.[5] Purification by crystallization from EtOAc/ Hexane 8:2 afforded **1c** as a white solid (5.0 mmol scale, 1.11 g, 75%). **Rf** = 0.65 (95:5 EtOAc/MeOH); **m.p.** = 83˚C; **$^1$H NMR** (500 MHz, $CDCl_3$) δ 7.31 – 7.08 (m, 10H), 5.06 (s, 2H), 3.32 (s, 6H) ppm; **$^{13}$C NMR** (126 MHz, $CDCl_3$) δ 165.9, 137.9, 133.8 (2C), 132.5, 128.5 (2C), 128.4 (2C), 127.4, 127.3, 127.2 (2C), 70.7, 64.5, 43.3 (2C) ppm; **IR** (neat): ν ($cm^{-1}$) = 3032, 3018, 2933, 1739, 1610, 1490, 1411, 1386, 1325, 1309, 1213, 1154, 1077, 1066, 1018, 939, 902, 853, 777, 761, 744, 694, 657, 581, 525; **HRMS** (ESI): calcd for $C_{17}H_{19}O_3S$ [M+H$^+$]: 303.1055; found: 303.1049.

Methyl 2-(dimethyl(oxo)-sulfaneylidene)-2-(4-(trifluoromethyl)phenyl)acetate **1d**

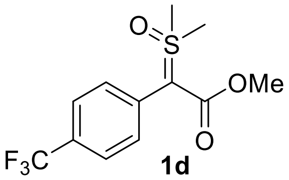


**1d**

Prepared according to the literature.[6] Purification by column chromatography in 95:5 EtOAc/MeOH afforded **1d** as a white solid (0.4 mmol scale, 110 mg, 94%). **Rf** = 0.63 (95:5 EtOAc/MeOH); **m.p.** = 163˚C; **$^1$H NMR** (500 MHz, $CDCl_3$) δ 7.58 – 7.52 (m, 2H), 7.43-7.41 (m, 2H), 3.64 (s, 3H), 3.48 (s, 6H) ppm; **$^{13}$C NMR** (126 MHz, $CDCl_3$) δ 166.2, 136.4, 133.1 (2C), 128.40 (q, *J* = 32.5 Hz), 125.2 (q, *J* = 3.8 Hz, 2C), 124.46 (q, *J* = 271.9 Hz), 68.7, 50.7, 43.8 (2C) ppm; **IR** (neat): ν (cm$^{-1}$) = 3089, 3011, 2999, 2925, 1624, 1438, 1327, 1220, 1172, 1123, 1068, 1036, 872, 849, 766, 758, 691, 608; **HRMS** (ESI): calcd for $C_{12}H_{14}F_3O_3S$ [M+H$^+$]: 295.0610; found: 295.0610.

Methyl 2-(4-chlorophenyl)-2-(dimethyl(oxo)-sulfaneylidene)acetate **1e**

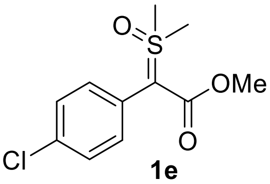


**1e**

Prepared according to the literature.[6] Purification by column chromatography in 95:5 EtOAc/MeOH afforded **1e** as a white solid (0.4 mmol scale, 101.2 mg, 97%). **Rf** = 0.48 (95:5 EtOAc/MeOH); **m.p.** = 140 ˚C; **$^1$H NMR** (500 MHz, $CDCl_3$) δ 7.25 – 7.12 (m, 4H), 3.54 (s, 3H), 3.36 (s, 6H) ppm; **$^{13}$C NMR** (126 MHz, $CDCl_3$) δ 166.5, 134.9 (2C), 133.1, 130.9, 128.7 (2C), 68.7, 50.6, 43.5 (2C) ppm; **IR** (neat): ν (cm$^{-1}$) = 3031, 3010, 3001, 2943, 2925, 1622, 1482, 1437, 1419, 1350, 1328, 1214, 1191, 1167, 1085, 1044, 1011, 946, 889, 832, 754, 721, 691, 528; **HRMS** (ESI): calcd for $C_{11}H_{14}ClO_3S$ [M+H$^+$]: 261.0347; found: 261.0347.

Methyl 2-(dimethyl(oxo)-sulfaneylidene)-2-(naphthalen-2-yl)acetate **1f**

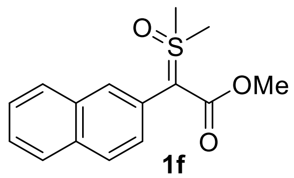


**1f**

Prepared according to the literature.[6] Purification by column chromatography in 95:5 EtOAc/MeOH afforded **1f** as a white solid (0.4 mmol scale, 81.3 mg, 73%). **Rf** = 0.43 (95:5 EtOAc/MeOH); **m.p.** = 106 ˚C; **$^1$H NMR** (500 MHz, $CDCl_3$) δ 7.85 – 7.75 (m, 4H), 7.49 – 7.43 (m, 2H), 7.41 (dd, *J* = 8.4, 1.8 Hz, 1H), 3.63 (s, 3H), 3.44 (s, 6H) ppm; **$^{13}$C NMR** (126 MHz, $CDCl_3$) δ 166.8, 133.7, 132.6, 132.2, 131.9, 130.1, 128.0 (2C), 127.7, 126.2 (2C), 70.7, 50.6, 43.3 (2C) ppm; **IR** (neat): ν (cm$^{-1}$) = 3049, 3019, 3005, 2941, 1636, 1622, 1592, 1429, 1355, 1312, 1295, 1256, 1215, 1171, 1121, 1088, 1022, 934,

865, 822, 749, 721, 692, 539, 520; **HRMS** (ESI): calcd for $C_{15}H_{17}O_3S$ [M+H$^+$]: 277.0893; found: 277.0893.

Ethyl 2-(4-bromophenyl)-2-(dimethyl(oxo)-sulfaneylidene)acetate **1g**

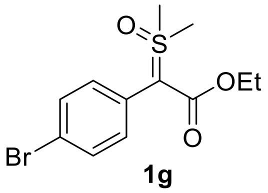


**1g**

Prepared according to the literature from ethyl 2-(4-bromophenyl)acetate.[5] White solid (3.07 mmol scale, 502mg, 51%). **Rf** = 0.50 (95:5 EtOAc/MeOH); **m.p.** = 158 ˚C; **$^1$H NMR** (500 MHz, $CDCl_3$) δ 7.48 – 7.36 (m, 2H), 7.21 – 7.13 (m, 2H), 4.10 (q, *J* = 7.1 Hz, 2H), 3.43 (s, 6H), 1.19 (t, *J* = 7.1 Hz, 3H) ppm; **$^{13}$C NMR** (126 MHz, $CDCl_3$) δ 166.1, 135.0 (2C), 131.6, 131.5 (2C), 120.9, 68.8, 59.1, 43.6 (2C), 14.9 ppm; **IR** (neat): ν (cm$^{-1}$) = 3023, 2995, 2920, 1617, 1214, 1167, 1085, 1042, 837, 757, 718, 676, 524; **HRMS** (ESI): calcd for $C_{12}H_{16}BrO_3S$ [M+H$^+$]: 318.9998; found: 318.9998.

Methyl 2-(dimethyl(oxo)-sulfaneylidene)-2-(p-tolyl)acetate **1h**

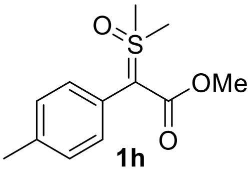


**1h**

Prepared according to the literature from methyl p-tolylacetate.[5] White solid (3.15 mmol scale, 234 mg, 31%). **Rf** = 0.51 (95:5 EtOAc/MeOH); **m.p.** = 125 ˚C; **$^1$H NMR** (500 MHz, $CDCl_3$) δ 7.26 – 6.99 (m, 4H), 3.58 (s, 3H), 3.36 (s, 6H), 2.33 (s, 3H) ppm; **$^{13}$C NMR** (126 MHz, $CDCl_3$) δ 166.9, 137.2, 133.8 (2C), 129.3 (2C), 126.6; 70.0, 50.4, 43.0 (2C), 21.2 ppm; **IR** (neat): ν (cm$^{-1}$) = 3007, 2971, 2947, 2873, 1605, 1447, 1431, 1374, 1321, 1282, 1181, 1109, 1067, 1049, 1024, 1005, 977, 884, 792, 750, 689, 582, 503, 490; **HRMS** (ESI): calcd for $C_{12}H_{17}O_3S$ [M+H$^+$]: 241.0893; found: 241.0893.

*Tert*-butyl 2-(dimethyl(oxo)-sulfaneylidene)-2-phenylacetate **1i**

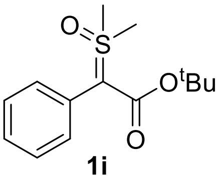


**1i**

Prepared according to literature procedures.[5,7] White solid (1.61 mmol scale,160 mg, 37%). **Rf** = 0.47 (95:5 EtOAc/MeOH); **m.p.** = 71 ˚C; **$^1$H NMR** (500 MHz, $CDCl_3$) δ 7.32 – 7.26 (m, 4H), 7.25 – 7.15 (m, 1H), 3.38 (s, 6H), 1.44 (s, 9H) ppm; **$^{13}$C NMR** (126 MHz, $CDCl_3$) δ 166.2, 133.5 (2C), 128.2 (2C), 126.6, 116.3, 71.5, 43.7 (2C), 41.1, 29.0 (3C) ppm; **IR** (neat): ν (cm$^{-1}$) = 3392, 3010, 2969, 2925, 1738, 1600, 1590, 1473, 1365, 1332,

1241, 1196, 1161, 1084, 1012, 972, 941, 850, 765, 736, 699, 648, 522; **HRMS** (ESI): calcd for $C_{14}H_{21}O_3S$ [M+H$^+$]: 269.1206; found: 269.1206.

Methyl 2-(dimethyl(oxo)-sulfaneylidene)-2-(pyridin-3-yl)acetate **1j**

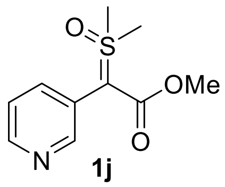


**1j**

Prepared according to the literature.[6] Purification by column chromatography in 95:5 EtOAc/MeOH afforded **1j** as a beige solid (0.4 mmol scale, 76 mg, 83%). **Rf** = 0.08 (95:5 EtOAc/MeOH); **m.p.** = 124˚C; **$^1$H NMR** (500 MHz, $CDCl_3$) δ 8.51 (d, *J* = 1.7 Hz, 1H), 8.43 (dd, *J* = 4.8, 1.7 Hz, 1H), 7.60 (dt, *J* = 7.9, 2.0 Hz, 1H), 7.22 (ddd, *J* = 7.9, 4.8, 0.9 Hz, 1H), 3.61 (s, 3H), 3.46 (s, 6H) ppm; **$^{13}$C NMR** (126 MHz, $CDCl_3$) δ 166.4, 154.0, 147.5, 140.4, 128.7, 123.2, 50.6, 43.7 (2C) ppm; **IR** (neat): ν (cm$^{-1}$) = 3029, 3009, 2943, 2925, 1737, 1621, 1479, 1439, 1417, 1351, 1324, 1224, 1195, 1167, 1086, 1054, 1032, 1005, 946, 889, 811, 755, 722, 690, 617, 520; **HRMS** (ESI): calcd for $C_{10}H_{14}NO_3S$ [M+H$^+$]: 228.0689; found: 228.0689.

2,2,2-trichloroethyl 2-(dimethyl(oxo)-l6-sulfaneylidene)-2-phenylacetate **1k**

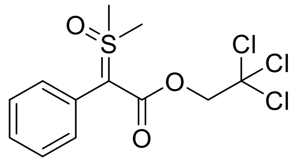


**1k**

Prepared according to the literature.[8] The spectroscopy data were in good agreement with the literature.[8] With solid (3.0 mmol scale, 827.1 mg, 80 %). **Rf** = 0.35 (1:1 Hexane EtOAc); **$^1$H NMR** (500 MHz, $CDCl_3$) δ 7.40 – 7.24 (m, 5H), 4.75 (s, 2H), 3.45 (s, 6H). ppm; **$^{13}$C NMR** (126 MHz, $CDCl_3$) δ 163.8, 133.8 (3C), 131.5, 128.5 (2C), 127.5, 96.6, 73.0, 71.1, 43.2 (2C) ppm.

## 4. General Procedure for Enantioselective S-H Insertion of Sulfoxonium Ylides

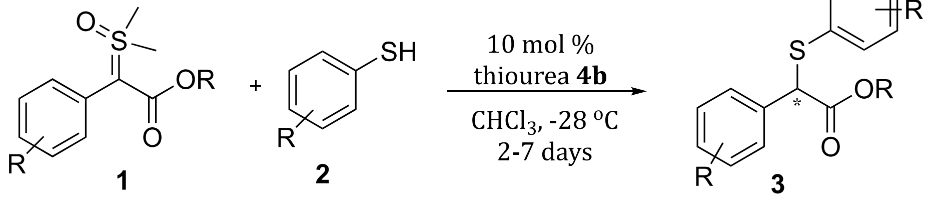


To a 2 mL reaction vial with a Teflon coated septum screw-top was added 0.1 mmol of sulfoxonium ylide (1 equiv.), 0.2 mL of $CHCl_3$ (0.5 M), and 0.01 mmol thiourea catalyst

**4b** (0.1 eq.) The reaction was stirred at -28 ˚C for twenty minutes before addition of 0.1 mmol thiol (1 equiv.). The reaction was stirred in the freezer (-28˚C) until all starting materials were consumed (1-7 days). The product was purified by flash column chromatography in 1:9 EtOAc/Hexanes. The *ee* value was determined by chiral HPLC analysis of the purified product.

Methyl (*R*)-2-phenyl-2-(phenylthio)acetate **3a**

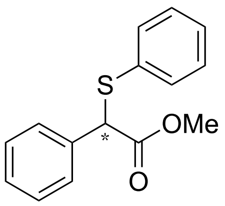


**3a**

Prepared from **1a** and benzenethiol. The spectroscopy data were in good agreement with the literature.[5,9] White solid (22.4 mg, 87%). **Rf** = 0.41 (9:1 EtOAc/Hex); **$^1$H NMR** (500 MHz, CDCl3): δ 7.38 – 7.34 (m, 2H), 7.31 – 7.27 (m, 2H), 7.26-7.22 (m, 3H), 7.20 – 7.17 (m, 3H), 4.84 (s, 1H), 3.60 (s, 3H) ppm; **$^{13}$C NMR** (126 MHz, CDCl3): δ 171.0, 135.8, 133.9, 132.8 (2C), 129.1 (2C), 128.8 (2C), 128.7 (2C), 128.5, 128.2, 56.5, 52.9 ppm; the preferred absolute stereochemistry of **3a** $[\alpha]_D^{23}$ = -110.6 (c = 1.94, $CHCl_3$) for 85% *ee* was stablished as *R* by comparing the sign of the optical rotation (lit.[9] $[\alpha]_D^{22}$ = +97.8 (c = 0.732, $CHCl_3$) for 74% *ee* of (*S*)-**3a**); **HPLC**: $t_R$ = 12.64 min (major, *R*), $t_R$ = 14.38 min (minor, *S*), *ee* = 85%; (CHIRALPAK ® OD-H, 90.0:10.0 hexane/isopropanol, 1 mL/min, 254 nm, 22 °C).

Methyl (*R*)-2-phenyl-2-(phenylthio)acetate **3a** (1.0 mmol scale)

Same general experiment procedure. White solid (223 mg, 86% y, 84% *ee*).

(-)-methyl 2-((4-nitrophenyl)thio)-2-phenylacetate **3b**

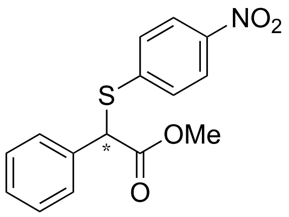


**3b**

Prepared from **1a** and 4-nitrobenzenethiol. The reaction was allowed to stir for 2 days. Purification by column chromatography in 1:9 EtOAc/Hex afforded **3b** as a white solid (16.2 mg, 53%). **Rf** = 0.22 (1:9 EtOAc/Hex); **m.p** = 108˚C; **$^1$H NMR** (500 MHz, $CDCl_3$): δ 8.07 – 8.00 (m, 2H), 7.45 – 7.39 (m, 2H), 7.35 – 7.25 (m, 5H), 5.05 (s, 1H), 3.67 (s, 3H) ppm; **$^{13}$C NMR** (126 MHz, $CDCl_3$): δ 170.2, 146.4, 144.5, 134.4, 129.3 (2C), 129.2 (2C), 129.1, 128.6 (2C), 124.2 (2C), 54.7, 53.4 ppm; **IR** (neat): ν ($cm^{-1}$) = 3109, 3058, 3032, 2962, 2921, 2841, 1730, 1595, 1578, 1510, 1480, 1455, 1433, 1338, 1283, 1220, 1184, 1152, 1120, 1091, 983, 924, 892, 866, 854, 836, 816, 768, 737, 680, 568, 476; **HRMS**

(ESI): calcd for $C_{15}H_{14}NO_4S$ [M+H$^+$]: 304.0638; found: 304.0638; $[\alpha]_D^{23}$ = -96.9 (c = 0.83, $CHCl_3$); **HPLC**: $t_R$ = 17.97 min (minor), $t_R$ = 19.06 min (major), *ee* = 88%; (CHIRALPAK ® OD-H, 90.0:10.0 hexane/isopropanol, 0.6 mL/min, 254 nm, 22 °C).

(-)-methyl 2-((4-bromophenyl)thio)-2-phenylacetate **3c**

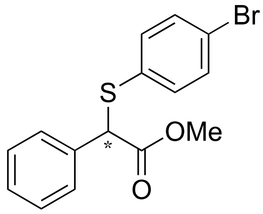


**3c**

Prepared from **1a** and 4-bromobenzenethiol. The reaction was allowed to stir for 28 hours and the spectroscopy data were in good agreement with the literature.[10] White solid (26.3 mg, 78%). **Rf** = 0.23 (1:9 EtOAc/Hex); **m.p** = 51 ˚C; **$^1$H NMR** (500 MHz, $CDCl_3$): δ 7.36 – 7.28 (m, 4H), 7.28 – 7.22 (m, 3H), 7.16 – 7.11 (m, 2H), 4.80 (s, 1H), 3.62 (s, 3H) ppm; **$^{13}$C NMR** (126 MHz, $CDCl_3$): δ 170.8, 135.4, 134.5 (2C), 132.8, 132.2 (2C), 128.9 (2C), 128.6 (3C), 122.6, 56.43, 52.97 ppm; **HRMS** (ESI): calcd for $C_{15}H_{14}BrO_2S$ [M+H$^+$]: 336.9892; found: 336.9892; $[\alpha]_D^{23}$ = -106.6 (c = 2.21, $CHCl_3$); **HPLC**: $t_R$ = 4.74 min (major), $t_R$ = 5.47 min (minor), *ee* = 82%; (CHIRALPAK ® OD-H, 90.0:10.0 hexane/isopropanol, 1 mL/min, 254 nm, 22 °C).

Methyl (-) 2-((4-chlorophenyl)thio)-2-phenylacetate **3d**

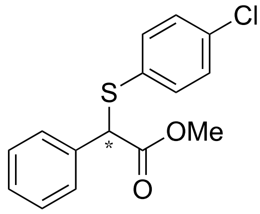


**3d**

Prepared from **1a** and 4-chlorobenzenethiol. The reaction was allowed to stir for 22 hours and the spectroscopy data were in good agreement with the literature.[10] White solid (24.5 mg, 84%). **Rf** = 0.73 (1:9 EtOAc/Hex); **$^1$H NMR** (500 MHz, $CDCl_3$): δ 7.35 – 7.31 (m, 2H), 7.28 – 7.19 (m, 5H), 7.17 – 7.13 (m, 2H), 4.79 (s, 1H), 3.62 (s, 3H) ppm; **$^{13}$C NMR** (126 MHz, CDCl3): δ 170.8, 135.4, 134.6, 134.4 (2C), 132.1, 129.3 (2C), 128.9 (2C), 128.7 (2C), 128.6, 56.6, 52.9 ppm; $[\alpha]_D^{23}$ = -11.4 (c =1.74, $CHCl_3$); **HPLC**: $t_R$ = 4.61 min (major), $t_R$ = 5.34 min (minor), *ee* = 82%; (CHIRALPAK ® OD-H, 90.0:10.0 hexane/isopropanol, 1 mL/min, 254 nm, 22 °C).

Methyl (-) 2-((2-chlorophenyl)thio)-2-phenylacetate **3e**

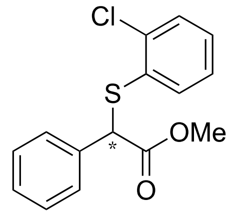

**3e**

Prepared from **1a** and 2-chlorobenzenethiol. The reaction was allowed to stir for 6 days. Purification by column chromatography in 1:9 EtOAc/Hex afforded **3e** as a colorless oil (19.2 mg, 66%). **Rf** = 0.67 (1:9 EtOAc/Hex); **$^1$H NMR** (500 MHz, $CDCl_3$): δ 7.47 (m, 2H), 7.40 (dd, *J* = 7.9, 1.5 Hz, 1H), 7.36 – 7.27 (m, 4H), 7.19 (td, *J* = 7.7, 1.7 Hz, 1H), 7.14 (td, *J* = 7.6, 1.5 Hz, 1H), 5.07 (s, 1H), 3.68 (s, 3H) ppm; **$^{13}$C NMR** (126 MHz, CDCl3): δ 170.7, 136.7, 135.1, 133.5, 133.0, 130.1, 129.1, 128.9 (2C), 128.8 (2C), 128.6, 127.3, 54.5, 53.0 ppm; **IR** (neat): ν ($cm^{-1}$) = 3062, 3031, 2952, 1736, 1495, 1451, 1433, 1274, 1214, 1148, 1035, 1007, 743, 728, 695, 659, 496; **HRMS** (ESI): calcd for $C_{15}H_{14}ClO_2S$ [M+H$^+$]: 293.0398; found: 293.0398; $[\alpha]_D^{23}$ = -86.1 (c = 1.22, $CHCl_3$); **HPLC**: $t_R$ = 5.39 min (major), $t_R$ = 6.56 min (minor), *ee* = 77%; (CHIRALPAK ® OD-H, 90.0:10.0 hexane/isopropanol, 1 mL/min, 254 nm, 22 °C).

Methyl (-) 2-((3-chlorophenyl)thio)-2-phenylacetate **3f**

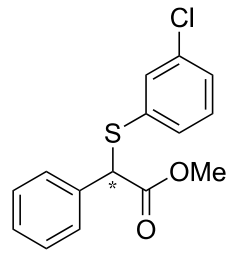

**3f**

Prepared from **1a** and 3-chlorobenzenethiol. The reaction was allowed to stir for 22 hours. Purification by column chromatography in 1:9 EtOAc/Hex afforded **3f** as a colorless oil (22.9 mg, 79%). **Rf** = 0.19 (1:9 EtOAc/Hex); **$^1$H NMR** (500 MHz, $CDCl_3$): δ 7.39 – 7.32 (m, 2H), 7.29 – 7.21 (m, 4H), 7.17 – 7.08 (m, 3H), 4.85 (s, 1H), 3.62 (s, 3H) ppm; **$^{13}$C NMR** (126 MHz, $CDCl_3$): δ 170.7, 135.9, 135.3, 134.7, 132.1, 130.4, 130.1, 129.0 (2C), 128.7, 128.6 (2C), 128.2, 56.2, 53.0 ppm; **IR** (neat): ν ($cm^{-1}$) = 3063, 3031, 2952, 1737, 1576, 1563, 1496, 1461, 1434, 1399, 1280, 1214, 1190, 1148, 1073, 1007, 778, 727, 696, 664; **HRMS** (ESI): calcd for $C_{15}H_{14}ClO_2S$ [M+H$^+$]: 293.0398; found: 293.0398; $[\alpha]_D^{23}$ = -111.1 (c = 1.35, $CHCl_3$); **HPLC**: $t_R$ = 4.73 min (major), $t_R$ =5.45 min (minor), *ee* = 80%; (CHIRALPAK ® OD-H, 90.0:10.0 hexane/isopropanol, 1 mL/min, 254 nm, 22 °C).

Methyl (-) 2-((4-methoxyphenyl)thio)-2-phenylacetate **3g**

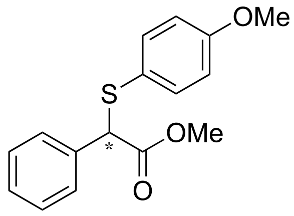

**3g**

Prepared from **1a** and 4-methoxybenzenethiol. The reaction was allowed to stir for 6 days and the spectroscopy data were in good agreement with the literature.[11] Colorless oil (28.8 mg, 94%). **Rf** = 0.18 (1:9 EtOAc/Hex); **$^1$H NMR** (500 MHz, $CDCl_3$) δ 7.31 (m, 2H), 7.27 – 7.20 (m, 5H), 6.74 – 6.70 (m, 2H), 4.69 (s, 1H), 3.72 (s, 3H), 3.60 (s, 3H) ppm; **$^{13}$C NMR** (126 MHz, $CDCl_3$) δ 171.2, 160.4, 136.4 (2C), 136.0, 128.7 (4C), 128.3, 123.8, 114.6 (2C), 57.6, 55.4, 52.7 ppm; $[\alpha]_D^{23}$ = -77.6 (c = 2.71, $CHCl_3$); **HPLC**: $t_R$ = 5.51 min (92%), $t_R$ = 7.03 min (8%), *ee* = 80%; (CHIRALPAK ® OD-H, 90.0:10.0 hexane/isopropanol, 1 mL/min, 254 nm, 22 °C).

Methyl (-) 2-((2-methoxyphenyl)thio)-2-phenylacetate **3h**

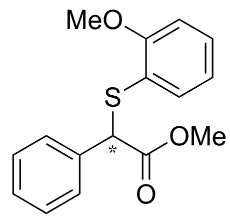

**3h**

Prepared from **1a** and 2-methoxybenzenethiol. The reaction was allowed to stir for 7 days. Purification by column chromatography in 1:4 EtOAc/Hex afforded **3h** as a colorless oil (18.6 mg, 64%); **Rf** = 0.47 (1:4 EtOAc/Hex); **$^1$H NMR** (500 MHz, $CDCl_3$): δ 7.39 – 7.31 (m, 2H), 7.27 – 7.13 (m, 5H), 6.82 – 6.71 (m, 2H), 5.00 (s, 1H), 3.80 (s, 3H), 3.58 (s, 3H) ppm; **$^{13}$C NMR** (126 MHz, $CDCl_3$): δ 171.1, 158.9, 135.9, 134.1, 129.7, 128.6 (2C), 128.5 (2C), 128.2, 121.6, 120.9, 110.8, 55.8, 53.8, 52.6 ppm; **IR** (neat): ν ($cm^{-1}$) = 3063, 3030, 3006, 2951, 2837, 1735, 1581, 1476, 1454, 1432, 1273, 1243, 1213, 1182, 1146, 1070, 1022, 750, 729, 696, 576; **HRMS** (ESI): calcd for for $C_{16}H_{17}O_3S$ [M+H$^+$]: 289.0893; found: 289.0893; $[\alpha]_D^{23}$ = -100.0 (c = 1.77, $CHCl_3$); **HPLC**: $t_R$ = 7.57 min (major), $t_R$ 16.78 min (minor), *ee* = 77%; (CHIRALPAK ® OD-H, 90.0:10.0 hexane/isopropanol, 1 mL/min, 254 nm, 22 °C).

Methyl (-) 2-phenyl-2-(p-tolylthio)acetate **3i**

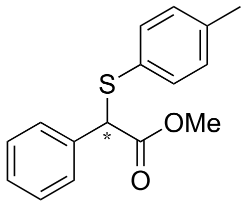

**3i**

Prepared from **1a** and 4-methylbenzenethiol. The reaction was allowed to stir for 7 days and the spectroscopy data were in good agreement with the literature.[11] Colorless oil (23.3 mg, 86%). **Rf** = 0.71 (1:4 EtOAc/Hex); **$^1$H NMR** (500 MHz, $CDCl_3$): δ 7.42 – 7.29 (m, 2H), 7.28 – 7.13 (m, 5H), 7.07 – 6.88 (m, 2H), 4.76 (s, 1H), 3.59 (s, 3H), 2.23 (s, 3H) ppm; **$^{13}$C NMR** (126 MHz, $CDCl_3$): δ 171.1, 138.5, 135.9, 133.5 (2C), 130.0, 129.9 (2C), 128.8 (2C), 128.7 (2C), 128.4, 56.9, 52.8, 21.3 ppm; $[\alpha]_D^{23}$ = -106.1 (c = 1.97, $CHCl_3$); **HPLC**: $t_R$= 9.65 min (major), $t_R$= 12.58 min (minor), *ee* = 85%; (CHIRALPAK® OD-H, 95.0:5.0 hexane/isopropanol, 0.5 mL/min, 254 nm, 22 °C).

Methyl (-) 2-phenyl-2-(o-tolylthio)acetate **3j**

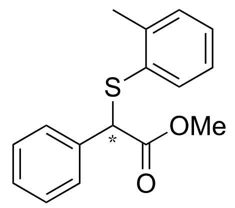


**3j**

Prepared from **1a** and 2-methylbenzenethiol. The reaction was allowed to stir for 7 days. Purification by column chromatography in 1:9 EtOAc/Hex afforded **3j** as a colorless oil (14.5 mg, 53%); **Rf** = 0.69 (1:4 EtOAc/Hex); **$^1$H NMR** (500 MHz, $CDCl_3$): δ 7.41 – 7.33 (m, 2H), 7.28 – 7.19 (m, 4H), 7.13 – 7.05 (m, 2H), 7.05 – 6.97 (m, 1H), 4.77 (s, 1H), 3.58 (s, 3H), 2.30 (s, 3H) ppm; **$^{13}$C NMR** (126 MHz, $CDCl_3$): δ 171.1, 140.6, 135.8, 133.1 (2C), 130.5, 128.8 (2C), 128.6 (2C), 128.4, 128.2, 126.6, 55.6, 52.8, 20.7 ppm; **IR** (neat): ν ($cm^{-1}$) = 3061, 3030, 2951, 1737, 1600, 1589, 1469, 1454, 1433, 1344, 1278, 1213, 1146, 1007, 749, 728, 696, 499; **HRMS** (ESI): calcd for $C_{16}H_{17}O_2S$ [$M+H^+$]: 273.0944; found: 273.0944; $[\alpha]_D^{23}$ = -82.5 (c = 1.31, $CHCl_3$); **HPLC**: $t_R$ = 12.19 min (minor), $t_R$ = 12.63 min (major), *ee* = 79%; (CHIRALPAK ® AD-H, 98.0:2.0 hexane/isopropanol, 0.5 mL/min, 254 nm, 22 °C).

Methyl (-)-2-((2,6-dimethylphenyl)thio)-2-phenylacetate **3k**

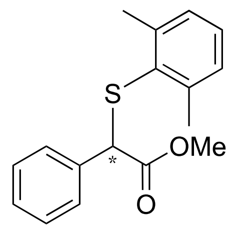


**3k**

Prepared from **1a** and 2,6-dimethylbenzenethiol. The reaction was allowed to stir for 7 days. Purification by column chromatography in 1:9 EtOAc/Hex afforded **3k** as a white solid (5.4 mg, 19%); **Rf** = 0.70 (1:4 EtOAc/Hex); **m.p.** = 61 ˚C; **$^1$H NMR** (500 MHz, $CDCl_3$): δ 7.36 – 7.29 (m, 2H), 7.23-7.21 (m, 3H), 7.09 – 6.95 (m, 3H), 4.51 (s, 1H), 3.52 (s, 3H), 2.36 (s, 6H) ppm; **$^{13}$C NMR** (126 MHz, $CDCl_3$): δ 171.2, 144.0 (2C), 136.3, 131.6, 129.2, 128.6 (2 C), 128.6 (2C), 128.3 (3C), 55.2, 52.6, 21.9 (2C) ppm; **IR** (neat): ν ($cm^{-1}$) = 3057, 2953**,** 2923, 2853, 1744, 1585, 1496**,** 1455, 1435, 1377**,** 1338, 1300, 1280,

1207, 1151, 1076, 1053, 1031, 1008, 896, 857, 775, 735, 696, 560; **HRMS** (ESI): calcd for $C_{17}H_{19}O_2S$ [M+H$^+$]: 287.1100; found: 287.1100; $[\alpha]_D^{23}$ = -101.3 (c = 0.39, $CHCl_3$); **HPLC**: $t_R$ = 4.58 min (major), $t_R$ = 5.73 min (minor), *ee* = 86%; (CHIRALPAK ® OD-H, 98.0:2.0 hexane/isopropanol, 1 mL/min, 254 nm, 22 °C).

Methyl (-) 2-((4-hydroxyphenyl)thio)-2-phenylacetate **3l**

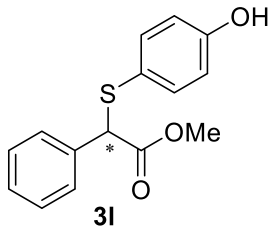


**3l**

Prepared from **1a** and 4-mercaptophenol. The reaction was allowed to stir for 2 days. Purification by column chromatography in 1:4 EtOAc/Hex afforded **3l** as a white solid (26.5 mg, 97%); **Rf** = 0.28 (1:4 EtOAc/Hex); **m.p.** = 63 ˚C; **$^1$H NMR** (500 MHz, $CDCl_3$): δ 7.34 – 7.29 (m, 2H), 7.27 – 7.17 (m, 5H), 6.66 – 6.60 (m, 2H), 5.58 (s, 1H), 4.70 (s, 1H), 3.60 (s, 3H) ppm; **$^{13}$C NMR** (126 MHz, $CDCl_3$): δ 171.6, 156.7, 136.6 (2C), 135.7, 128.8 (2C), 128.7 (2C), 128.4, 123.6, 116.2 (2C), 57.6, 52.9 ppm; **IR** (neat): ν (cm$^{-1}$) = 3383, 3059, 3039, 3005, 2953, 2849, 1703, 1599, 1581, 1494, 1455, 1431, 1285, 1269, 1246, 1214, 1200, 1010, 829, 694, 520; **HRMS** (ESI): calcd for $C_{15}H_{15}O_3S$ [M+H$^+$]: 275.0736; found: 275.0736; $[\alpha]_D^{23}$ = -76.9 (c = 2.65, $CHCl_3$); **HPLC**: $t_R$ = 8.12 min (major), $t_R$ =10.08 min (minor), *ee* =63%; (CHIRALPAK ® OD-H, 90.0:10.0 hexane/isopropanol, 1 mL/min, 254 nm, 22 °C).

Methyl (-) 2-(naphthalen-2-ylthio)-2-phenylacetate **3m**

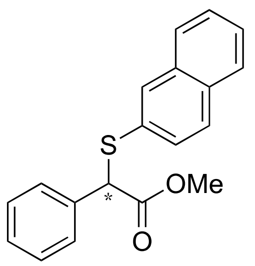


**3m**

Prepared from **1a** and naphthalene-2-thiol. The reaction was allowed to stir for 7 days. Purification by column chromatography in 1:9 EtOAc/Hex afforded **3m** as a white solid (26.9 mg, 87%); **Rf** = 0.60 (1:4 EtOAc/Hex); **m.p.** = 100 ˚C; **$^1$H NMR** (500 MHz, $CDCl_3$): δ 7.76 (d, *J* = 1.5 Hz, 1H), 7.73 – 7.68 (m, 1H), 7.67 – 7.61 (m, 2H), 7.42 – 7.32 (m, 5H), 7.27 – 7.20 (m, 3H), 4.94 (s, 1H), 3.59 (s, 3H) ppm; **$^{13}$C NMR** (126 MHz, $CDCl_3$): δ 171.0, 135.7, 133.7, 132.8, 131.8, 131.2, 129.7, 128.9 (2C), 128.7, 128.7 (2C), 128.5, 127.8, 127.7, 126.7, 126.6, 56.4, 52.9 ppm; **IR** (neat): ν (cm$^{-1}$) = 3054, 3002, 2955, 1742, 1583, 1495, 1456, 1432, 1338, 1299, 1281, 1188, 1145, 1077, 1001, 964, 903, 895, 870, 820, 807, 746, 731, 698, 509; **HRMS** (ESI): calcd for $C_{19}H_{17}O_2S$ [M+H$^+$]: 309.0944; found: 309.0944; $[\alpha]_D^{23}$ = -114.9 (c = 2.64, $CHCl_3$); **HPLC**: $t_R$ = 6.64 min (major), $t_R$ = 8.50 min

(minor), *ee* = 81%; (CHIRALPAK ® OD-H, 95.0:5.0 hexane/isopropanol, 1 mL/min, 254 nm, 22 °C).

Ethyl (-) 2-(4-bromophenyl)-2-((4-methoxyphenyl)thio)- phenylacetate **3n**

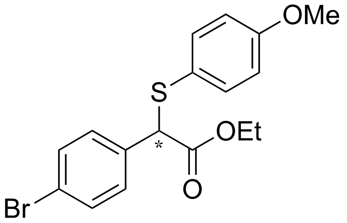


**3n**

Prepared from **1g** and 4-methoxybenzenethiol. The reaction was allowed to stir for 6 days. Purification by column chromatography in 1:9 EtOAc/Hex afforded **3n** as a colorless oil (24.2 mg, 64%); **Rf** = 0.19 (1:9 EtOAc/Hex); **$^{1}$H NMR** (500 MHz, $CDCl_3$) δ 7.47 – 7.42 (m, 2H), 7.35 – 7.30 (m, 2H), 7.30 – 7.25 (m, 2H, 1H-$CDCl_3$), 6.84 – 6.78 (m, 2H), 4.69 (s, 1H), 4.20 – 4.08 (m, 2H), 3.81 (s, 3H), 1.20 (t, *J* = 7.1 Hz, 3H) ppm. **$^{13}$C NMR** (126 MHz, $CDCl_3$) δ 170.2, 160.5, 136.5 (2C), 135.2, 131.8 (2C), 130.5 (2C), 123.3, 122.4, 114.7 (2C), 61.9, 56.9, 55.5, 14.15 ppm; **IR** (neat): ν ($cm^{-1}$) = 2979, 2960, 2928, 2838, 2361, 1731, 1591, 1571, 1492, 1462, 1442, 1406, 1367, 1324, 1287, 1272, 1246, 1210, 1172, 1145, 1096, 1073, 1027, 1011, 958, 878, 826, 755, 710, 641, 627; **HRMS** (ESI): calcd for $C_{17}H_{18}BrO_3S$ [M+H$^+$]: 381.0155; found: 381.0155; $[\alpha]_D^{23}$ = -1,485.1 (c = 2.05, $CHCl_3$); **HPLC**: $t_R$ = 15.01 min (major), $t_R$ = 17.85 min (minor), *ee* = 87%; (CHIRALPAK ® OJ, 90.0:10.0 hexane/isopropanol, 1 mL/min, 254 nm, 22 °C).

Ethyl (-) 2-(4-bromophenyl)-2-((4-(trifluoromethyl)phenyl)thio)- phenylacetate **3o**

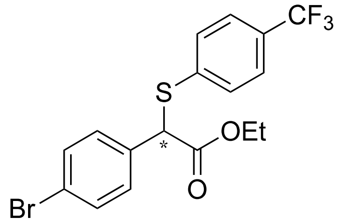


**3o**

Prepared from **1g** and 4-(trifluoromethyl)benzenethiol. The reaction was allowed to stir for 3 days. Purification by column chromatography in 1:9 EtOAc/Hex afforded **3o** as a white solid (22.5 mg, 54%); **Rf** = 0.66 (1:9 EtOAc/Hex); **m.p** = 46 ˚C; **$^{1}$H NMR** (500 MHz, $CDCl_3$) δ 7.56 – 7.45 (m, 4H), 7.44 – 7.37 (m, 2H), 7.37 – 7.31 (m, 2H), 4.93 (s, 1H), 4.23 – 4.07 (m, 2H), 1.19 (t, *J* = 7.1 Hz, 3H) ppm; **$^{13}$C NMR** (126 MHz, $CDCl_3$) δ 169.6, 138.9, 134.3, 132.2 (2C), 131.3 (2C), 130.3 (2C), 126.0, 126.0 (2C), 123.0, 62.4, 55.0, 14.1 ppm; **$^{19}$F NMR** (471 MHz, $CDCl_3$) δ -62.70 ppm. **IR** (neat): ν ($cm^{-1}$) = 2988, 2966, 2925, 2325, 1913, 1737, 1605, 1571, 1488, 1402, 1369, 1325, 1295, 1270, 1212, 1157, 1119, 1094, 1075, 1064, 1026, 1011, 967, 950, 875, 822, 781, 724, 712, 701, 657, 632; **HRMS** (ESI): calcd for $C_{17}H_{15}BrF_3O_2S$ [M+H$^+$]: 418.9923; found: 418.9923; $[\alpha]_D^{23}$ = -95.7 (c = 1.39, $CHCl_3$); **HPLC**: $t_R$ = 15.85 min (major), $t_R$ = 16.49 min

(minor), *ee* = 82%; (CHIRALPAK ® AD-H, 98.0:2.0 hexane/isopropanol, 0.5 mL/min, 254 nm, 22 °C).

Methyl (-) 2-(4-chlorophenyl)-2-((4-methoxyphenyl)thio)acetate **3p**

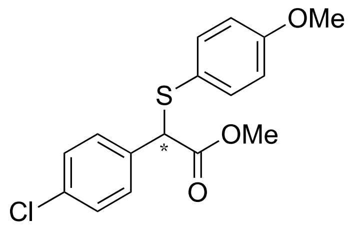


**3p**

Prepared from **1e** and 4-methoxybenzenthiol. The reaction was allowed to stir for 7 days. Purification by column chromatography in 1:4 EtOAc/Hex afforded **3p** as a colorless oil (21.5 mg, 67%); **Rf** = 0.38 (1:4 EtOAc/Hex); **$^1$H NMR** (500 MHz, $CDCl_3$) δ 7.25 – 7.17 (m, 6H), 6.77 – 6.66 (m, 2H), 4.63 (s, 1H), 3.71 (s, 3H), 3.60 (s, 3H) ppm. **$^{13}$C NMR** (126 MHz, $CDCl_3$) δ 170.8, 160.6, 136.6 (2C), 134.6, 134.2, 130.1 (2C), 128.8 (2C), 123.2, 114.7 (2C), 56.7, 55.4, 52.8 ppm; **IR** (neat): ν ($cm^{-1}$) = 3003, 2952, 2838, 2045, 1735, 1591, 1571, 1491, 1461, 1435, 1408, 1325, 1287, 1274, 1245, 1212, 1172, 1146, 1091, 1029, 1015, 908, 826, 799, 766, 719, 693, 641, 627; **HRMS** (ESI): calcd for $C_{16}H_{16}ClO_3S$ [M+H$^+$]: 323.0503; found: 323.0503; $[\alpha]_D^{23}$ = -35.3 (c = 2.10, $CHCl_3$); **HPLC**: $t_R$ = 16.67 min (major), $t_R$ = 24.03 min (minor), *ee* = 66%, (CHIRALPAK ® OJ, 80.0:20.0 hexane/isopropanol, 1 mL/min, 254 nm, 22 °C).

Methyl (-) 2-((4-nitrophenyl)thio)-2-(*p*-tolyl)-phenylacetate **3q**

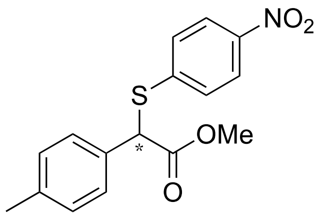


**3q**

Prepared from **1h** and 4-nitrobenzenethiol. The reaction was allowed to stir for 2 days. Purification by column chromatography in 1:9 EtOAc/Hex afforded **3q** as a yellow solid (27.7 mg, 87%); **Rf** = 0.33 (1:9 EtOAc/Hex); **m.p** = 111 °C; **$^1$H NMR** (500 MHz, $CDCl_3$) δ 8.15 – 8.05 (m, 2H), 7.41 – 7.34 (m, 4H), 7.20 – 7.14 (m, 2H), 5.10 (s, 1H), 3.73 (s, 3H), 2.35 (s, 3H) ppm. **$^{13}$C NMR** (126 MHz, $CDCl_3$) δ 170.3, 144.8, 139.1, 131.3, 130.0 (2C), 129.0 (2C), 128.4 (2C), 126.7, 124.2 (2C), 54.4, 53.3, 21.3 ppm; **IR** (neat): ν ($cm^{-1}$) = 2953, 2922, 1737, 1596, 1577, 1510, 1480, 1434, 1399, 1336, 1032, 1278, 1221, 1208, 1187, 1150, 1111, 1093, 1009, 907, 853, 838, 792, 740, 682, 626; **HRMS** (ESI): calcd for $C_{16}H_{15}NNaO_4S$ [M+Na$^+$]: 340.0614; found: 340.0614; $[\alpha]_D^{23}$ = - 99.4 (c = 2.34, $CHCl_3$); **HPLC**: $t_R$ = 8.87 min (minor), $t_R$ = 9.79 min (major), *ee* = 84%; (CHIRALPAK ® OD-H, 90.0:10.0 hexane/isopropanol, 1 mL/min, 254 nm, 22 °C).

Methyl (-) 2-((4-bromophenyl)thio)-2-(*p*-tolyl)-phenylacetate **3r**

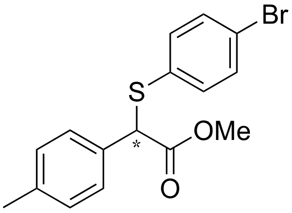

**3r**

Prepared from **1h** and 4-bromobenzenethiol. The reaction was allowed to stir for 3 days. Purification by column chromatography in 1:9 EtOAc/Hex afforded **3r** as a light yellow solid (24.1 mg, 69%); **Rf** = 0.50 (1:9 EtOAc/Hex); **m.p** = 54 ˚C; **$^1$H NMR** (500 MHz, $CDCl_3$) δ 7.42 – 7.35 (m, 2H), 7.33 – 7.28 (m, 2H), 7.24 – 7.19 (m, 2H), 7.16 – 7.11 (m, 2H), 4.85 (s, 1H), 3.68 (s, 3H), 2.33 (s, 3H) ppm; **$^{13}$C NMR** (126 MHz, $CDCl_3$) δ 170.9, 138.6, 134.3 (2C), 133.1, 132.3, 132.2 (2C), 129.6 (2C), 128.5 (2C), 122.5, 56.2, 52.9, 21.3 ppm; **IR** (neat): ν ($cm^{-1}$) = 2951, 2921, 2362, 1907, 1737, 1607, 1565, 1512, 1493, 1473, 1434, 1385, 1347, 1303, 1276, 1220, 1207, 1187, 1148, 1111, 1091, 1069, 1008, 908, 857, 816, 739, 730, 700, 629, 613; **HRMS** (ESI): calcd for $C_{16}H_{15}BrNaO_2S$ [M+Na$^+$]: 372.9868; found: 372.9868; $[\alpha]_D^{23}$ = -114.2 (c = 1.44, $CHCl_3$); **HPLC**: $t_R$ = 4.39 min (major), $t_R$ = 5.08 min (minor), *ee* = 78%; (CHIRALPAK ® OD-H, 90.0:10.0 hexane/isopropanol, 1 mL/min, 254 nm, 22 °C).

Methyl (-) 2-(*p*-tolyl)-2-(*o*-tolylthio)-phenylacetate **3s**

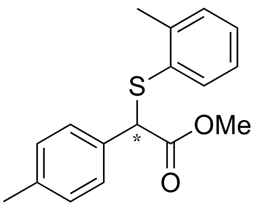

**3s**

Prepared from **1h** and 2-methylbenzenethiol. The reaction was allowed to stir for 6 days. Purification by column chromatography in 1:9 EtOAc/Hex afforded **3s** as a colorless oil (17.1 mg, 45%); **Rf** = 0.56 (1:9 EtOAc/Hex); **$^1$H NMR** (500 MHz, $CDCl_3$) δ 7.35 – 7.32 (m, 3H), 7.20 – 7.08 (m, 5H), 4.83 (s, 1H), 3.65 (s, 3H), 2.39 (s, 3H), 2.33 (s, 3H) ppm; **$^{13}$C NMR** (126 MHz, $CDCl_3$) δ 171.2, 140.4, 138.4, 133.4, 132.8, 132.7, 130.5, 129.5 (2C), 128.5 (2C), 128.1, 126.6, 55.3, 52.8, 21.3, 20.7 ppm; **IR** (neat): ν ($cm^{-1}$) = 2951, 2923, 2361, 1738, 1589, 1512, 1469, 1455, 1434, 1380, 1332, 1303, 1276, 1219, 1206, 1187, 1146, 1063, 1047, 1005, 946, 907, 825, 792, 740, 711, 681, 635, 611; **HRMS** (ESI): calcd for $C_{17}H_{19}O_2S$ [M+H$^+$]: 287.1100; found: 287.1100; $[\alpha]_D^{23}$ = -92.3 (c = 1.04, $CHCl_3$); **HPLC**: $t_R$ = 4.86 min (minor); $t_R$ = 5.25 min (major), *ee* = 82% (CHIRALPAK ® OD-H, 90.0:10.0 hexane/isopropanol, 1 mL/min, 254 nm, 22 °C).

Methyl (-) 2-((4-methoxyphenyl)thio)-2-(naphthalen-2-yl)acetate **3t**

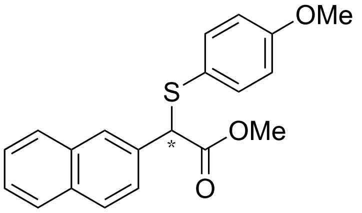


**3t**

Prepared from **1f** and 4-methoxybenzenthiol. The reaction was allowed to stir for 7 days. Purification by column chromatography in 1:4 EtOAc/Hex afforded **3t** as a beige solid (23 mg, 68%); **Rf** = 0.38 (1:4 EtOAc/Hex); **m.p** = 87 ˚C; **$^1$H NMR** (500 MHz, $CDCl_3$) δ 7.84-7.80 (m, 2H), 7.79 – 7.74 (m, 2H), 7.60 (dd, *J* = 8.5, 1.9 Hz, 1H), 7.52 – 7.43 (m, 2H), 7.36 – 7.29 (m, 2H), 6.81 – 6.74 (m, 2H), 4.93 (s, 1H), 3.77 (s, 3H), 3.70 (s, 3H) ppm; **$^{13}$C NMR** (126 MHz, $CDCl_3$) δ 171.1, 160.4, 136.4 (2C), 133.3, 133.2 (2C), 128.6, 128.2, 127.9, 127.8, 126.5, 126.4, 126.3, 123.7, 114.7 (2C), 57.8, 55.4, 52.8 ppm; **IR** (neat): ν ($cm^{-1}$) = 3003, 2951, 2837, 1734, 1591, 1571, 1493, 1461, 1435, 1406, 1367, 1286, 1245, 1173, 1145, 1104, 1029, 1007, 957, 899, 860, 827, 809, 749, 675, 640, 627; **HRMS** (ESI): calcd for $C_{20}H_{19}O_3S$ [M+H$^+$]: 339.1049; found: 339.1049; $[\alpha]_D^{23}$ = -122.8 (c = 1.49, $CHCl_3$); **HPLC**: $t_R$ = 8.62 min (major), $t_R$ = 11.69 min (minor), *ee* = 80%, (CHIRALPAK ® OD-H, 95.0:5.0 hexane/isopropanol, 1 mL/min, 254 nm, 22 °C).

Methyl (-) 2-((4-methoxyphenyl)thio)-2-(pyridin-3-yl)acetate **3u**

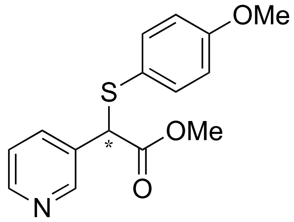


**3u**

Prepared from **1j** and 4-methoxybenzenthiol. The reaction was allowed to stir for 7 days. Purification by column chromatography in 1:4 EtOAc/Hex afforded **3u** as a colorless oil (16.8 mg, 58%); **Rf** = 0.22 (2:3 EtOAc/Hex); **$^1$H NMR** (500 MHz,$CDCl_3$) δ 8.43 (d, *J* = 24.1 Hz, 2H), 7.76 – 7.70 (m, 1H), 7.24 – 7.17 (m, 3H), 6.76 – 6.69 (m, 2H), 4.66 (s, 1H), 3.71 (s, 3H), 3.63 (s, 3H) ppm; **$^{13}$C NMR** (126 MHz, $CDCl_3$) δ 170.4, 160.8, 149.8, 149.5, 136.9 (2C), 136.3, 132.2, 123.6, 122.6, 114.8 (2C), 55.5, 54.6, 52.9 ppm; **IR** (neat): ν ($cm^{-1}$) = 2952, 2920, 2839, 1734, 1633, 1591, 1572, 1493, 1462, 1425, 1350, 1287, 1245, 1173, 1149, 1104, 1027, 1006, 958, 906, 830, 800, 711, 641, 627; **HRMS** (ESI): calcd for $C_{15}H_{16}NO_3S$ [M+H$^+$]: 290.0845; found: 290.0845; $[\alpha]_D^{23}$ = -35.3 (c = 1.58, $CHCl_3$); **HPLC**: $t_R$ = 11.20 min (major), $t_R$ = 13.52 min (minor), *ee* = 47%, (CHIRALPAK ® OD-H, 90.0:10.0 hexane/isopropanol, 1 mL/min, 254 nm, 22 °C).

Methyl (-) 2-((4-methoxyphenyl)thio)-2-(4-(trifluoromethyl)phenyl)acetate **3v**

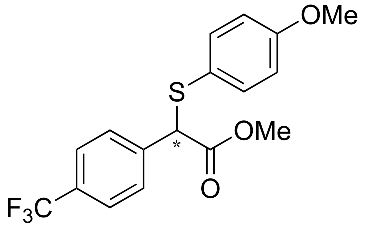

**3v**

Prepared from **1d** and 4-methoxybenzenthiol. The reaction was allowed to stir for 6 days. Purification by column chromatography in 1:4 EtOAc/Hex afforded **3v** as a colorless oil (20.2 mg, 57%); **Rf** = 0.24 (1:9 EtOAc/Hex); **$^{1}$H NMR** (500 MHz, CDCl3) δ 7.62 – 7.52 (m, 2H), 7.51 – 7.45 (m, 2H), 7.34 – 7.27 (m, 2H), 6.85 – 6.73 (m, 2H), 4.77 (s, 1H), 3.79 (s, 3H), 3.69 (s, 3H) ppm; **$^{13}$C NMR** (126 MHz, CDCl3) δ 170.5, 160.7, 140.1, 136.7 (2C), 134.4, 130.46 (q, *J* = 32.6 Hz), 129,2 (2C), 125.61 (q, *J* = 3.8 Hz, 2C), 124.12 (q, *J* = 272.0 Hz), 122.9, 114.8, 57.0, 55.5, 52.9 ppm; **IR** (neat): ν (cm$^{-1}$) = 2954, 2840, 1736, 1618, 1591, 1571, 1494, 1463, 1437, 1420, 1324, 1286, 1246, 1216, 1163, 1122, 1067, 1029, 1019, 910, 828, 799, 753, 720, 694, 640, 627; **HRMS** (ESI): calcd for $C_{17}H_{16}F_3O_3S$ [M+H$^+$]: 357.0767; found: 357.0767; $[\alpha]_D^{23}$ = -41,8 (c = 1.32, $CHCl_3$); **HPLC**: $t_R$ = 10.99 min (major), $t_R$ = 15.80 min (minor), *ee* = 45%, (CHIRALPAK ® OJ, 90.0:10.0 hexane/isopropanol, 1 mL/min, 254 nm, 22 °C).

Ethyl (-) 2-((4-methoxyphenyl)thio)-2-phenylacetate **3w**

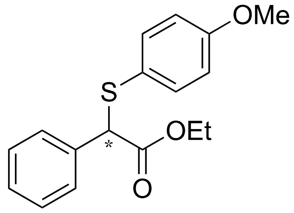

**3w**

Prepared from **1b** and 4-methoxybenzenthiol. The reaction was allowed to stir for 7 days. Purification by column chromatography in 1:4 EtOAc/Hex afforded **3w** as a colorless oil (27.3 mg, 90%); **Rf** = 0.42 (1:4 EtOAc/Hex); **$^{1}$H NMR** (500 MHz, $CDCl_3$) δ 7.35 – 7.30 (m, 2H), 7.27 – 7.18 (m, 5H), 6.75 – 6.68 (m, 2H), 4.67 (s, 1H), 4.04 (qq, *J* = 10.8, 7.1 Hz, 2H), 3.70 (s, 3H), 1.10 (t, *J* = 7.1 Hz, 3H) ppm. **$^{13}$C NMR** (126 MHz, $CDCl_3$) δ 170.7, 160.3, 136.3 (2C), 136.0, 128.7 (2C), 128.7 (2C), 128.2, 123.9, 114.6 (2C), 61.7, 57.6, 55.4, 14.2 ppm; **IR** (neat): ν (cm$^{-1}$) = 2980, 2837, 1731, 1653, 1591, 1571, 1541, 1493, 1455, 1392, 1367, 1341, 1285, 1245, 1212, 1172, 1143, 1095, 1076, 1026, 956, 916, 874, 829, 799, 727, 696, 641, 627; **HRMS** (ESI): calcd for $C_{17}H_{19}O_3S$ [M+H$^+$]: 303.1049; found: 303.1049; $[\alpha]_D^{23}$ = -82.8 (c = 2.49, $CHCl_3$); **HPLC**: $t_R$ = 5.71 min (major), $t_R$ = 7.85 min (minor), *ee* = 89%, (CHIRALPAK ® OD-H, 95.0:5.0 hexane/isopropanol, 1 mL/min, 254 nm, 22 °C).

Benzyl (-) 2-((4-methoxyphenyl)thio)-2-phenylacetate **3x**

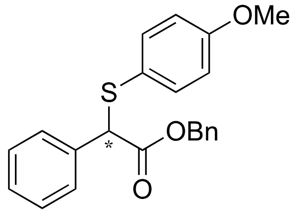

**3x**

Prepared according to general procedure from **1c** and 4-methoxybenzenethiol. Reaction was allowed to stir for 7 days. Purification by column chromatography in 1:4 EtOAc/Hex afforded **3x** as a white solid (31.4 mg, 86%); **Rf** = 0.38 (1:4 EtOAc/Hex); **m.p** = 40 ˚C; **$^{1}$H NMR** (500 MHz, $CDCl_3$) δ 7.46 – 7.39 (m, 2H), 7.35 – 7.28 (m, 8H), 7.24 – 7.17 (m, 2H), 6.80 – 6.71 (m, 2H), 5.14 (d, *J* = 12.3 Hz, 1H), 5.05 (d, *J* = 12.3 Hz, 1H), 4.83 (s, 1H), 3.78 (s, 3H) ppm; **$^{13}$C NMR** (126 MHz, $CDCl_3$) δ 170.6, 160.3, 136.3 (2C), 135.7, 135.5, 128.8 (2C), 128.7 (2C), 128.6 (2C), 128.4, 128.3, 128.3 (2C), 123.8, 114.6 (2C), 67.3, 57.4, 55.4 ppm; **IR** (neat): ν ($cm^{-1}$) = 3064, 3031, 2958, 2837, 1889, 1734, 1653, 1591, 1571, 1541, 1493, 1455, 1406, 1376, 1342, 1285, 1246, 1213, 1173, 1136, 1105, 1076, 1029, 1004, 971, 915, 828, 800, 733, 695, 641, 627; **HRMS** (ESI): calcd for $C_{22}H_{21}O_3S$ [M+H$^+$]: 365.1206; found: 365.1206; $[\alpha]_D^{23}$ = -63.2 (c = 2.67, $CHCl_3$); **HPLC**: $t_R$ = 7.50 min (major), $t_R$ = 8.70 min (minor), *ee* = 87%, (CHIRALPAK ® OD-H, 95.0:5.0 hexane/isopropanol, 1 mL/min, 254 nm, 22 °C).

*Tert*-butyl (-) 2-((4-methoxyphenyl)thio)-2-phenylacetate **3y**

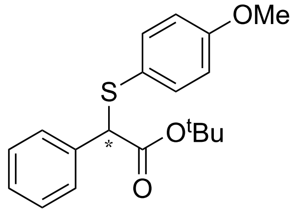

**3y**

Prepared from **1i** and 4-methoxybenzenthiol. The reaction was allowed to stir for 4 days. Purification by column chromatography in 1:4 EtOAc/Hex afforded **3y** as a white solid (21.8 mg, 66%); **Rf** = 0.40 (1:9 EtOAc/Hex); **m.p** = 42 ˚C; **$^{1}$H NMR** (500 MHz, $CDCl_3$) δ 7.34 – 7.29 (m, 2H), 7.28 – 7.17 (m, 5H), 6.74 – 6.69 (m, 2H), 4.59 (s, 1H), 3.71 (s, 3H), 1.30 (s, 9H) ppm; **$^{13}$C NMR** (126 MHz, $CDCl_3$) δ 169.7, 160.2, 136.5, 136.1 (2), 128.7 (2C), 128.6 (2C), 128.1, 124.4, 114.5 (2C), 82.1, 58.4, 55.5, 28.0 (3C) ppm; **IR** (neat): ν ($cm^{-1}$) = 2978, 2837, 1728, 1653, 1591, 1571, 1541, 1493, 1455, 1393, 1368, 1346, 1285, 1245, 1172, 1133, 1105, 1075, 1030, 1008, 961, 915, 829, 746, 696, 641, 627; **HRMS** (ESI): calcd for $C_{19}H_{23}O_3S$ [M+H$^+$]: 331.1362; found: 331.1362; $[\alpha]_D^{23}$ = -75.7 (c = 2.11, $CHCl_3$); **HPLC**: $t_R$ = 4.65 min (minor), $t_R$ = 4.91 min (major), *ee* = 90%, (CHIRALPAK ® AD-H, 90.0:10.0 hexane/isopropanol, 1 mL/min, 254 nm, 22 °C).

*Tert*-butyl (-) 2-(naphthalen-2-ylthio)-2-phenylacetate **3z**

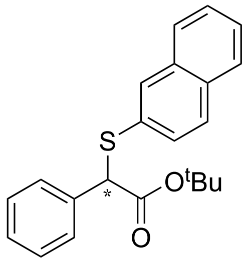

**3z**

Prepared from **1i** and naphthalene-2-thiol. The reaction was allowed to stir for 2 days. Purification by column chromatography in 1:4 EtOAc/Hex afforded **3z** as a white solid (30.4 mg, 87%); **Rf** = 0.44 (1:9 EtOAc/Hex); **m.p** = 75 ˚C; **$^1$H NMR** (500 MHz, $CDCl_3$) δ 7.87 – 7.83 (m, 1H), 7.82 – 7.76 (m, 1H), 7.75 – 7.71 (m, 2H), 7.50 – 7.44 (m, 5H), 7.36 – 7.27 (m, 3H), 4.95 (s, 1H), 1.35 (s, 9H) ppm; **$^{13}$C NMR** (126 MHz, $CDCl_3$) δ 169.6, 136.2, 133.7, 132.6, 132.0, 131.0, 129.4, 128.8 (2C), 128.6 (2C), 128.5, 128.3, 127.8, 127.6, 126.6, 126.4, 82.4, 57.0, 27.9 (3C) ppm; **IR** (neat): ν ($cm^{-1}$) = 3059, 2976, 2932, 2325, 1732, 1623, 1587, 1501, 1488, 1455, 1395, 1369, 1337, 1307, 1280, 1251, 1218, 1197, 1132, 1074, 1031, 1011, 960, 939, 920, 899, 871, 861, 852, 811, 781, 757, 742, 716, 700, 631; **HRMS** (ESI): calcd for $C_{22}H_{23}O_2S$ [M+H$^+$]: 351.1413; found: 351.1413 ; $[\alpha]_D^{23}$ = -87.0 (c = 2.25, $CHCl_3$); **HPLC**: $t_R$ = 21.89 min (major), $t_R$ = 24.85 min (minor), *ee* = 84%, (CHIRALPAK ® OJ, 95.0:5.0 hexane/isopropanol, 0.9 mL/min, 254 nm, 22 °C).

(-)-*tert*-butyl 2-((4-chlorophenyl)thio)-2-phenylacetate **3aa**

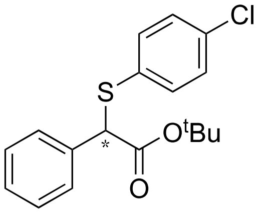

**3aa**

Prepared according to general procedure from **1i** and 4-chlorobenzenethiol. Reaction was allowed to stir for 2 days. Purification by column chromatography in 1:4 EtOAc/Hex afforded **3aa** as a white solid (21.0 mg, 63%); **Rf** = 0.38 (1:9 EtOAc/Hex); **m.p** = 54 ˚C; **$^1$H NMR** (500 MHz, $CDCl_3$) δ 7.44 – 7.38 (m, 2H), 7.36 – 7.27 (m, 5H), 7.25 – 7.17 (m, 2H), 4.78 (s, 1H), 1.37 (s, 9H) ppm; **$^{13}$C NMR** (126 MHz, $CDCl_3$) δ 169.3, 135.9, 134.1, 134.0 (2C), 132.8, 129.1 (2C), 128.8 (2C), 128.6 (2C), 128.4, 82.6, 57.4, 28.0 (3C) ppm; **IR** (neat): ν ($cm^{-1}$) = 3069, 2987, 2971, 2934, 2325, 1720, 1601, 1574, 1497, 1478, 1456, 1391, 1366, 1350, 1313, 1290, 1262, 1248, 1225, 1190, 1136, 1096, 1077, 1032, 1014, 960, 917, 865, 848, 825, 810, 768, 741, 726, 692; **HRMS** (ESI): calcd for $C_{18}H_{19}ClO_2S$ [M+H$^+$]: 335.0867; found: 335.0867; $[\alpha]_D^{23}$ = -78.3 (c = 1.50, $CHCl_3$); **HPLC:** $t_R$ = 4.72 min (minor), $t_R$ = 4.93 min (major), *ee* = 88%, (CHIRALPAK ® AD-H, 95.0:5.0 hexane/isopropanol, 1 mL/min, 254 nm, 22 °C).

(-)-2,2,2-trichloroethyl 2-((4-methoxyphenyl)thio)-2-phenylacetate **3ab**

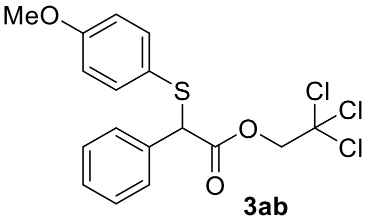


**3ab**

Prepared from **1k** and 4-methoxybenzenthiol. The reaction was allowed to stir for 7 days. Purification by column chromatography in 1:19 EtOAc/Hex afforded **3ab** as a colorless oil (24.0 mg, 59%); Rf = 0.42 (1:9 EtOAc/Hex); **$^1$H NMR** (400 MHz, $CDCl_3$) δ 7.49 – 7.43 (m, 2H), 7.41 – 7.29 (m, 5H), 6.83 – 6.77 (m, 2H), 4.89 (s, 1H), 4.71 (d, *J* = 11.9 Hz, 1H), 4.66 (d, *J* = 11.9 Hz, 1H), 3.78 (s, 3H) ppm. **$^{13}$C NMR** (101 MHz, $CDCl_3$) δ 169.23, 160.5, 136.4 (2C), 134.9, 132.8, 128.8 (2C), 128.6 (2C), 123.4, 114.7 (2C), 94.6, 74.7, 57.2, 55.5 ppm; **IR** (neat): ν ($cm^{-1}$) = 2960, 2837, 1749, 1590, 1492, 1245, 1172, 1119, 1026, 802, 694, 640, 570; **HRMS** (ESI): calcd for $C_{17}H_{14}Cl_3O_3S$ [M-H]: 402.9729; found: 402.9745; $[\alpha]_D^{21}$ = -53.6 (c = 2.05, $CHCl_3$); **HPLC:** $t_R$ = 7.83 min (major); $t_R$ = 8.95 min (minor), *ee* = 95% (Lux® Cellulose-4, 99.0:1.0 hexane/isopropanol, 1 mL/min, 254 nm, 22 °C).

(-)-2,2,2-trichloroethyl 2-phenyl-2-(phenylthio)acetate **3ac**

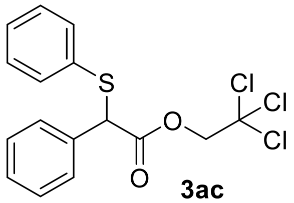


**3ac**

Prepared from **1k** and benzenthiol. The reaction was allowed to stir for 7 days. Purification by column chromatography in 1:19 EtOAc/Hex afforded **3ac** as a colorless oil (33.0 mg, 88%); Rf = 0.53 (1:9 EtOAc/Hex); **$^1$H NMR** (500 MHz, $CDCl_3$) δ 7.52 – 7.48 (m, 2H), 7.46 – 7.38 (m, 2H), 7.37 – 7.24 (m, 6H), 5.04 (s, 1H), 4.72 (d, *J* = 11.9 Hz, 1H), 4.66 (d, *J* = 11.9 Hz, 1H) ppm. **$^{13}$C NMR** (126 MHz, $CDCl_3$) δ 169.2, 134.8, 133.4, 133.0 (2C), 129.3 (2C), 128.9 (2C), 128.8 (3C), 128.4, 94.5, 74.8, 56.2 ppm. **IR** (neat): ν ($cm^{-1}$) = 3061, 2954, 1750, 1583, 1481, 1439, 1370, 1278, 1208, 1120, 1023, 814, 688, 570; **HRMS** (ESI): calcd for $C_{16}H_{12}Cl_3O_2S$ [M-H]: 372.9624; found: 372.9637; $[\alpha]_D^{22}$ = -71.4 (c = 2.40, $CHCl_3$); **HPLC:** $t_R$ = 6.11 min (major); tR = 7.01 min (minor), *ee* = 92% (CHIRALPAK ® AD-H, 95.0:5.0 hexane/isopropanol, 1 mL/min, 254 nm, 22 °C).

(-)-2,2,2-trichloroethyl 2-((2,5-dimethylphenyl)thio)-2-phenylacetate **3ad**

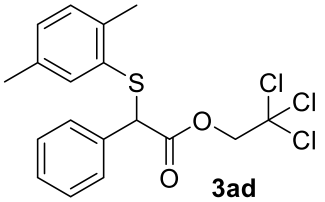

**3ad**

Prepared from **1k** and 2,5-dimethylbenzenthiol. The reaction was allowed to stir for 7 days. Purification by column chromatography in 1:19 EtOAc/Hex afforded **3ad** as a colorless oil (16.5 mg, 41%); Rf = 0.53 (1:9 EtOAc/Hex); $^{1}$**H NMR** (400 MHz, $CDCl_3$) δ 7.54 – 7.49 (m, 2H), 7.39 – 7.31 (m, 3H), 7.25 – 7.20 (m, 1H), 7.08 (d, *J* = 7.7 Hz, 1H), 7.01 – 6.96 (m, 1H), 4.96 (s, 1H), 4.71 (d, *J* = 11.9 Hz, 1H), 4.63 (d, *J* = 11.9 Hz, 1H), 2.37 (s, 3H), 2.25 (s, 3H) ppm. $^{13}$**C NMR** (101 MHz, $CDCl_3$) δ 169.3, 137.5, 136.3, 134.9, 133.9, 132.4, 130.5, 129.4, 128.9 (2C), 128.8 (2C), 128.7, 94.6, 74.8, 55.3, 20.9, 20.3 ppm. **IR** (neat): ν ($cm^{-1}$) = 2953, 2922, 1753, 1603, 1488, 1454, 1370, 1278, 1205, 1183, 1119, 1023, 902, 809, 716, 694, 570; **HRMS** (ESI): calcd for $C_{18}H_{16}Cl_3O_2S$ [M-H]: 400.9937; found: 400.9953; $[\alpha]_D^{21}$ = -52.9 (c = 1.20, $CHCl_3$); **HPLC:** $t_R$ = 11.97 min (minor); $t_R$ = 12.99 min (major), *ee* = 92% (CHIRALPAK ® AD-H, 99.0:1.0 hexane/isopropanol, 0.5 mL/min, 254 nm, 22 °C).

(-)-2,2,2-trichloroethyl 2-phenyl-2-(p-tolylthio)acetate **3ae**

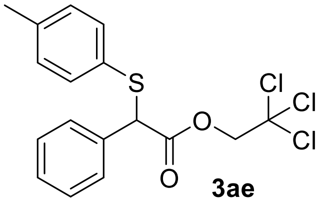

**3ae**

Prepared from **1k** and 4-methylbenzenthiol. The reaction was allowed to stir for 7 days. Purification by column chromatography in 1:49 EtOAc/Hex afforded **3ae** as a colorless oil (28.3 mg, 73%); Rf = 0.39 (1:9 EtOAc/Hex); $^{1}$**H NMR** (400 MHz, $CDCl_3$) δ 7.53 – 7.44 (m, 2H), 7.39 – 7.27 (m, 5H), 7.13 – 7.03 (m, 2H), 4.97 (s, 1H), 4.71 (d, *J* = 11.9 Hz, 1H), 4.65 (d, *J* = 11.9 Hz, 1H), 2.31 (s, 3H) ppm. $^{13}$**C NMR** (101 MHz, $CDCl_3$) δ 169.2, 138.8, 134.9, 133.6 (2C), 130.00 (2C) 129.6, 128.9 (2C), 128.8 (2C), 128.7, 94.5, 74.7, 56.6, 21.3 ppm. **IR** (neat): ν ($cm^{-1}$) = 3030, 2955, 2922, 1751, 1493, 1453, 1370, 1277, 1209, 1120, 1018, 801, 693, 570; **HRMS** (ESI): calcd for $C_{17}H_{14}Cl_3O_2S$ [M-H]: 386.9780; found: 386.9795 $[\alpha]_D^{22}$ = -61.3 (c = 2.03, $CHCl_3$); **HPLC:** $t_R$ = 5.36 min (major); $t_R$ = 7.71 min (minor), *ee* = 93% (CHIRALPAK ® AD-H, 97.0:1.5:1.5 hexane/methanol/ethanol, 1 mL/min, 254 nm, 22 °C).

(-)-Methyl 2-phenyl-2-(phenylsulfonyl)acetate **11**

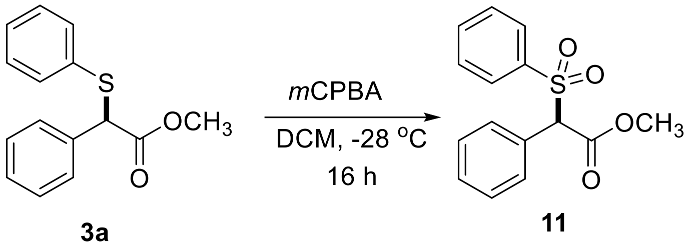

To a solution of **3a** (26.0 mg, 0.1 mmol) in DCM (0.5 mL) was added *m*CPBA (73. 6 mg, 0.32 mmol) at -28 °C, and the solution was stirred for 16 h in this temperature. The reaction was quenched by the addition of saturated aqueous $Na_2SO_3$ (3 mL) and the organic layer was separated. The aqueous layer was extracted with DCM and the combined organic extracts was washed with brine, dry over $Na_2SO_4$, filtered and the volatile was removed under reduced pressure. Purification by column chromatography in 1:4 EtOAc/Hex afforded **11** as white solid (26.3 mg, 91%); **Rf** = 0.24 (1:9 EtOAc/Hex); **m.p** = 83 ˚C; **$^1$H NMR** (500 MHz, $CDCl_3$) δ 7.57 – 7.49 (m, 3H), 7.38 – 7.19 (m, 7H), 5.04 (s, 1H), 3.70 (s, 3H) ppm; **$^{13}$C NMR** (126 MHz, $CDCl_3$) δ 165.4, 136.4, 134.3, 130.4 (2C), 130.1 (2C), 129.8, 128.7 (2C), 128.7 (2C), 128.0, 75.4, 53.3 ppm; **IR** (neat): ν ($cm^{-1}$) = 3065, 2955, 1741, 1585, 1497, 1478, 1448, 1435, 1324, 1310, 1285, 1219, 1198, 1144, 1081, 1007, 928, 909, 867, 818, 759, 718, 687, 614; **HRMS** (ESI): calcd for $C_{15}H_{15}O_4S$ [M+H$^+$]:291.0686; found: 291.0686; $[\alpha]_D^{22}$ = -9.7 (c = 2.15, $CHCl_3$); **HPLC**: $t_R$ = 12.92 min (minor), $t_R$ = 14.17 min (major), *ee* = 81%, (CHIRALPAK ® OD-H, 90.0:10.0 hexane/isopropanol, 1 mL/min, 254 nm, 22 °C).

(-)-2-phenyl-2-(phenylthio)ethan-1-ol **12**

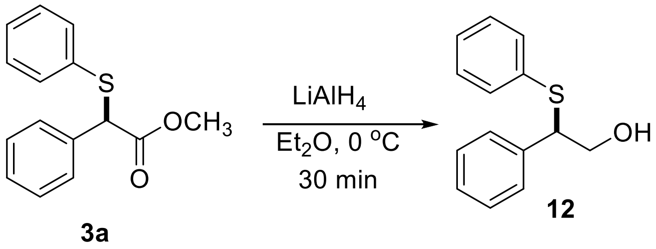

To a solution of $LiAlH_4$ (12 mg, 0.3 mmol) in $Et_2O$ (0.5 mL) was added dropwise **3a** in $Et_2O$ (0.5 mL) at 0 °C, and the solution was stirred for 30 min. The reaction was quenched with aqueous HCl (1 mol.$L^{-1}$) and the organic layer was separated. After usual workup the crude product was purified by column chromatography in 1:9 EtOAc/Hex afforded **12** as a colorless oil (19.5 mg, 85%); **Rf** = 0.22 (1:4 EtOAc/Hex); the spectroscopy data were in good agreement with the literature[9] **$^1$H NMR** (500 MHz, $CDCl_3$) δ 7.29 – 7.09 (m, 10H), 4.23 (t, *J* = 6.8 Hz, 1H), 3.85 (dd, *J* = 11.5, 6.8 Hz, 1H), 3.81 (dd, *J* = 11.5, 6.8 Hz, 1H), 1.91 (s, 1H) ppm; **$^{13}$C NMR** (126 MHz, $CDCl_3$) δ 139.1, 133.9, 132.7 (2C), 129.1 (2C), 128.9 (2C), 128.2 (2C), 127.9, 127.7, 65.4, 56.2 ppm; $[\alpha]_D^{23}$ = -130.2 (c = 1.28, $CHCl_3$); **HPLC**: $t_R$ = 21.29 min (major), $t_R$ = 26.82 min (minor), *ee* = 85%, (CHIRALPAK ® OD-H, 90.0:10.0 hexane/isopropanol, 1 mL/min, 254 nm, 22 °C).

()-2-methyl-1-phenyl-1-(phenylthio)propan-2-ol **13**

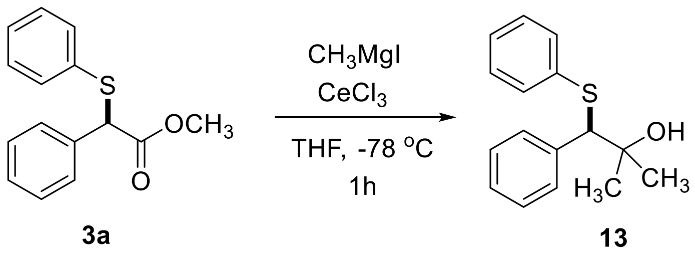

The solution of $CeCl_3$ (81 mg, 0.33 mmol) in THF (0.7 mL) was stirred for 1 h. To the suspension of $CeCl_3$ was added solution of **3a** (28 mg, 0.11 mmol) in THF (0.3 mL). the mixture was stirred for 1 h and cooled to –78 °C. Then, the MeMgI (2 mol.$L^{-1}$ in ether, 0.27 mL, 0.54 mmol) was added dropwise to the mixture. After 1 h, the reaction was quenched with acetic acid (1 mL). The aqueous layer was extracted with DCM and the combined organic extracts were washed with saturated aqueous $NaHCO_3$ and brine, dried over $Na_2SO_4$, filtered and concentrated under reduced pressure. The crude product was purified by column chromatography in 1:9 EtOAc/Hex and **13** was afforded as white solid (6.0 mg, 23%); **Rf** = 0.36 (1:4 EtOAc/Hex); the spectroscopy data were in good agreement with the literature.[9] **$^1$H NMR** (500 MHz, $CDCl_3$) δ 7.38 – 7.29 (m, 2H), 7.24 – 7.15 (m, 5H), 7.13 – 7.01 (m, 3H), 4.13 (s, 1H), 2.36 (sl, 1H), 1.25 (s, 3H), 1.21 (s, 3H) ppm. **$^{13}$C NMR** (126 MHz, $CDCl_3$) δ 140.1, 135.9, 131.2 (2C), 129.6 (2C), 129.0 (2C), 128.2 (2C), 127.53, 126.96, 73.4, 67.6, 28.1, 27.2 ppm; $[\boldsymbol{\alpha}]_{\boldsymbol{D}}^{\mathbf{23}}$ = -209.4 (c = 0.54, $CHCl_3$); **HPLC**: $t_R$ = 11.18 min (major), $t_R$ = 14.51 min (minor), *ee* = 86%, (CHIRALPAK ® OD-H, 95.0:5.0 hexane/isopropanol, 1 mL/min, 254 nm, 22 °C).

## 5. General procedure for the titration of chiral catalyst 4b with 1a and 2a

A stock solution of chiral catalyst **4b** (50 mM) in $CDCl_3$ was prepared in a 1 mL volumetric flask. Then, 100 µL of this stock solution was transfer to NMR tubes and, after added quantitatively portions of **1a** or **2a**, $CDCl_3$ was added to quantities enough to final 500 µL (final concentration of catalyst = 10 mM). $^1$H NMR spectra of the solutions were recorded after the addition of each equivalent, ensuring that the concentration of **4b** did not change.

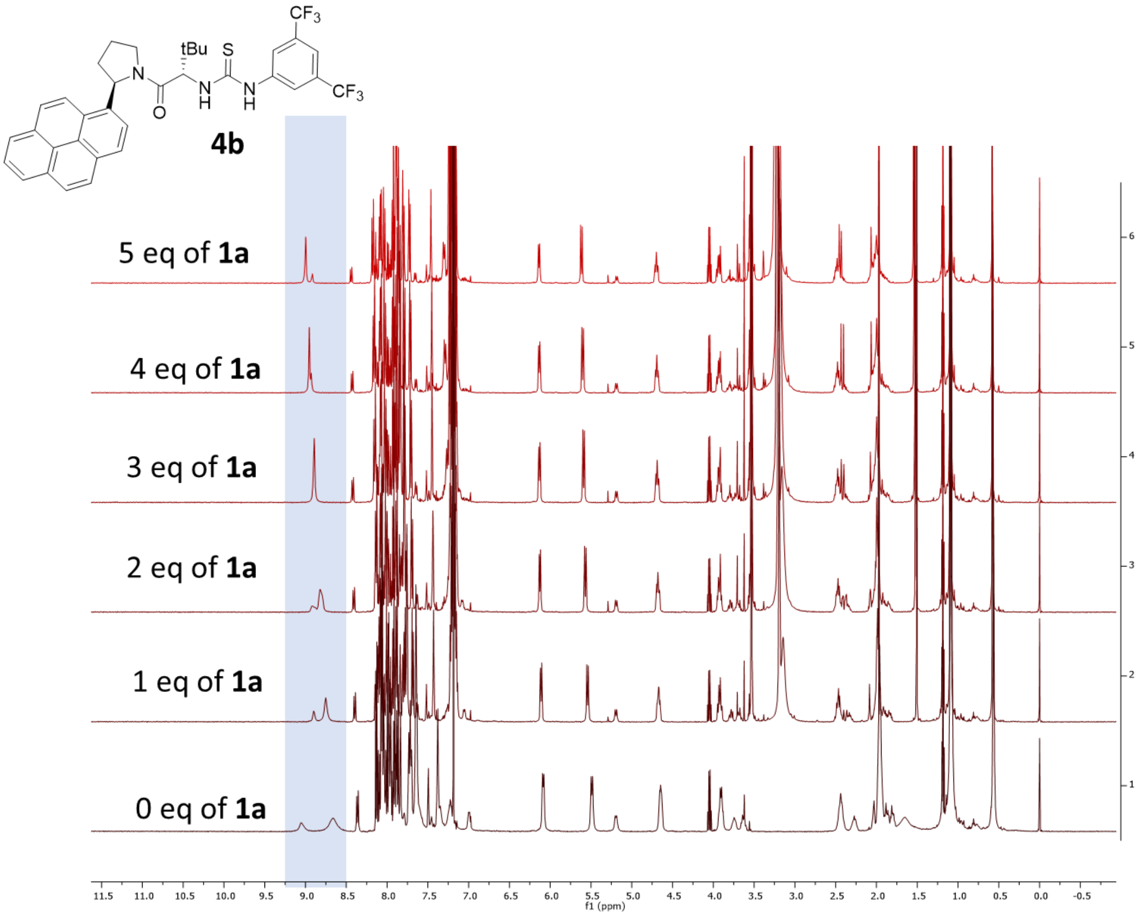


**Titration of 4b with 1a [4b]=10.0x10$^{-3}$ M in $CDCl_3$**

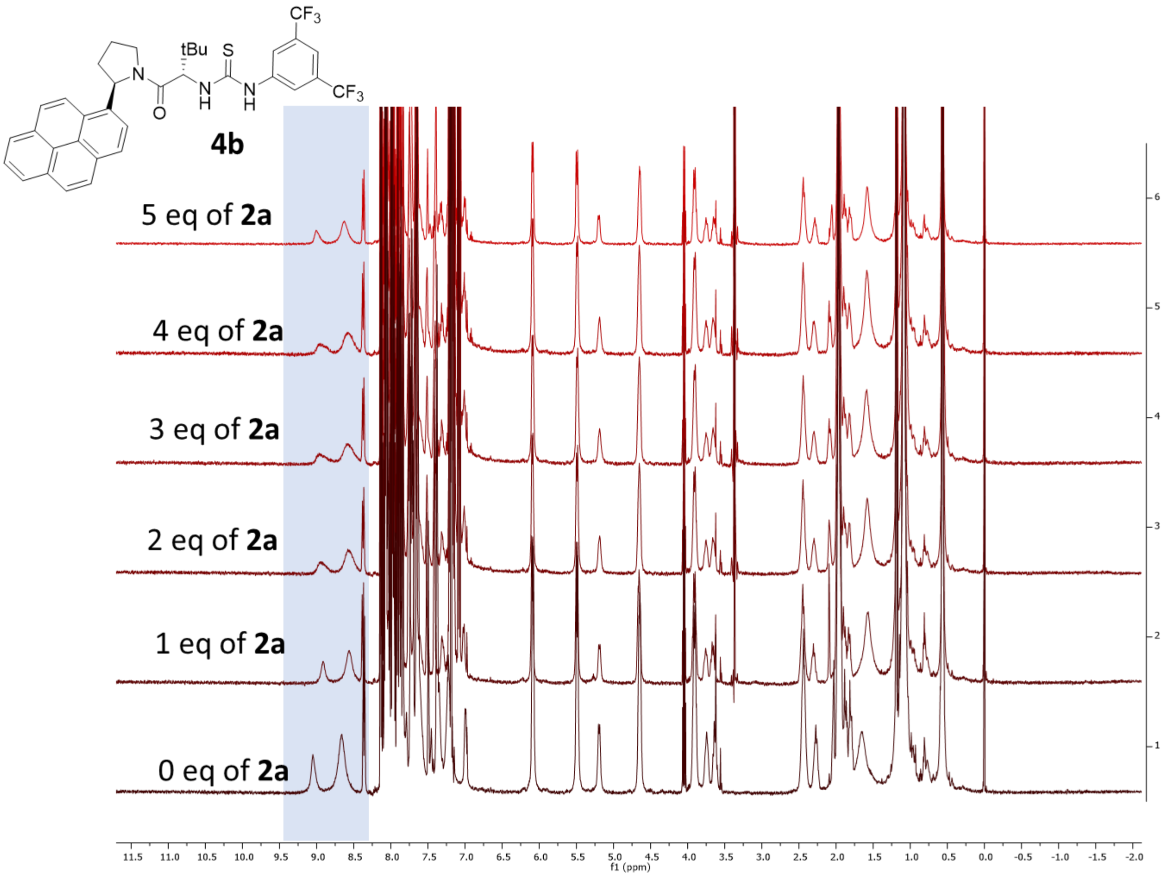


**Titration of 4b with 2a [4b]=10.0x10$^{-3}$ M in $CDCl_3$**

## 6. $^1$H and $^{13}$C NMR Spectra

Only spectra for new sulfoxonium ylides are provided. For spectra of other sulfoxonium ylide, please see: Org. Lett.2016,18,12,3034-3037; Organic Letters, 20 (22), p. 7206-7211, 2018 and Organic Letters 2019, 21, 1, 296-299.

**$^1$H NMR (500 MHz, CDCl$_3$)** benzyl 2-(dimethyl(oxo)-$\lambda^6$-sulfanylidene)-2-phenylacetate **1c**

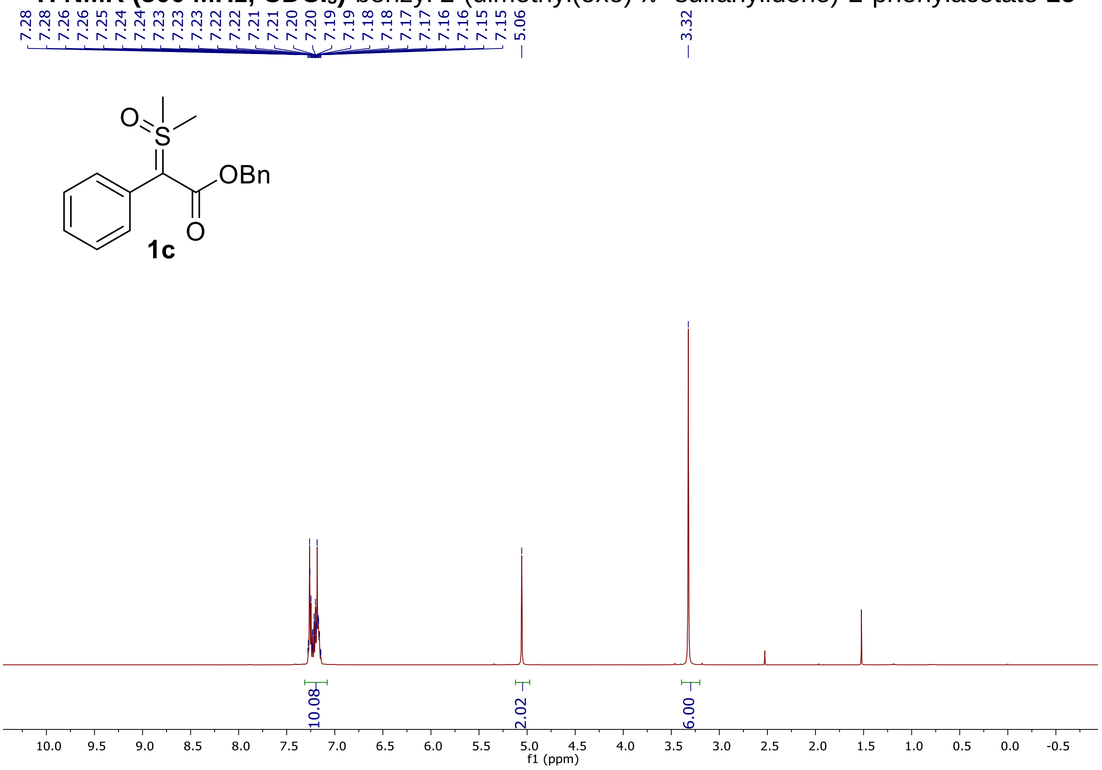


**$^{13}$C NMR (126 MHz, CDCl$_3$)** benzyl 2-(dimethyl(oxo)-$\lambda^6$-sulfanylidene)-2-phenylacetate **1c**

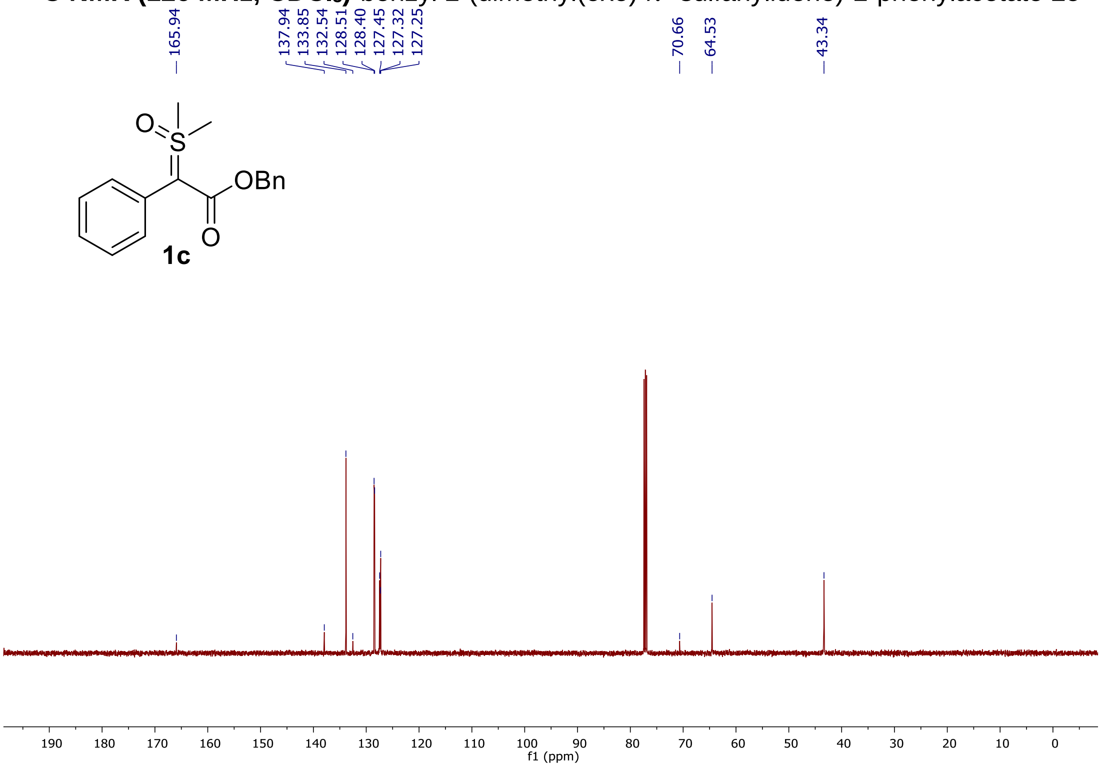

**$^{1}$H NMR (500 MHz, CDCl$_3$)** methyl 2-(dimethyl(oxo)- $\lambda^6$-sulfanylidene)-2-(4-(trifluoromethyl)phenyl)acetate **1d**

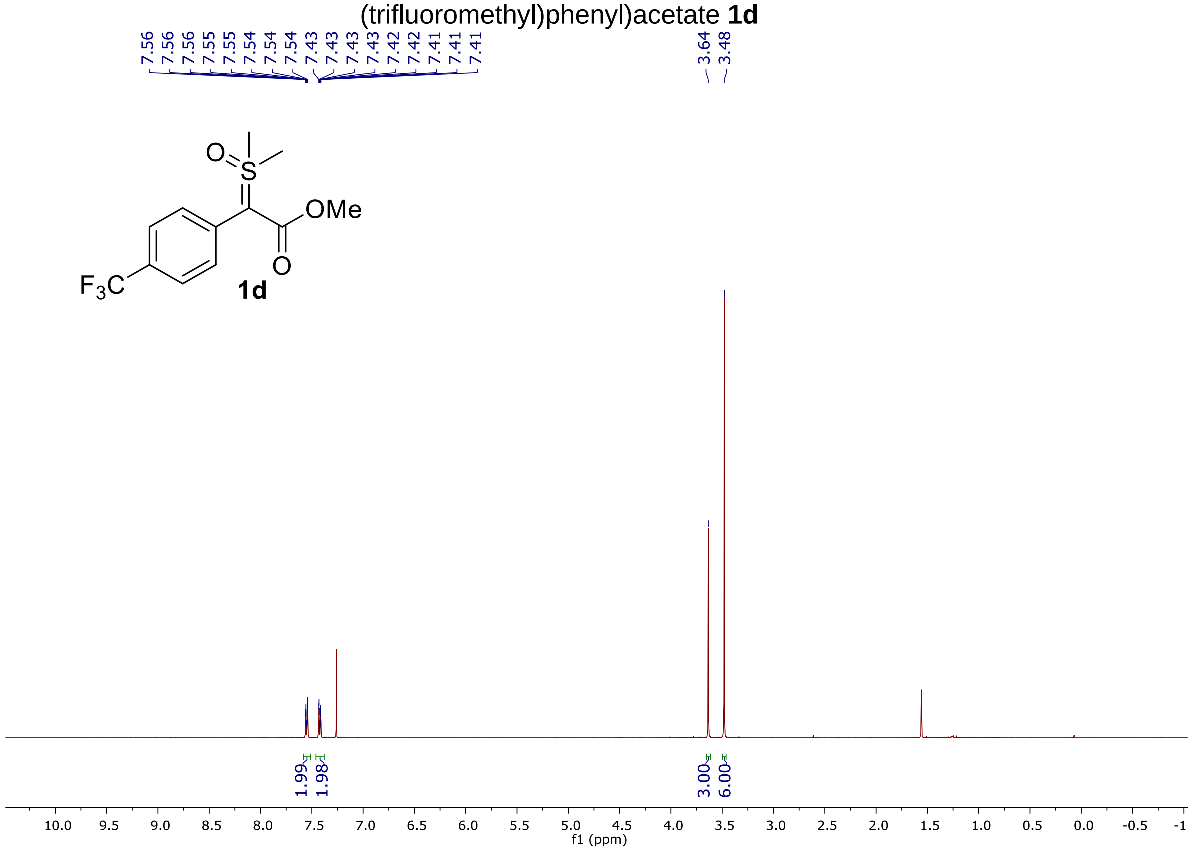


**$^{13}$C NMR (126 MHz, CDCl$_3$)** methyl 2-(dimethyl(oxo)- $\lambda^6$-sulfanylidene)-2-(4-(trifluoromethyl)phenyl)acetate **1d**

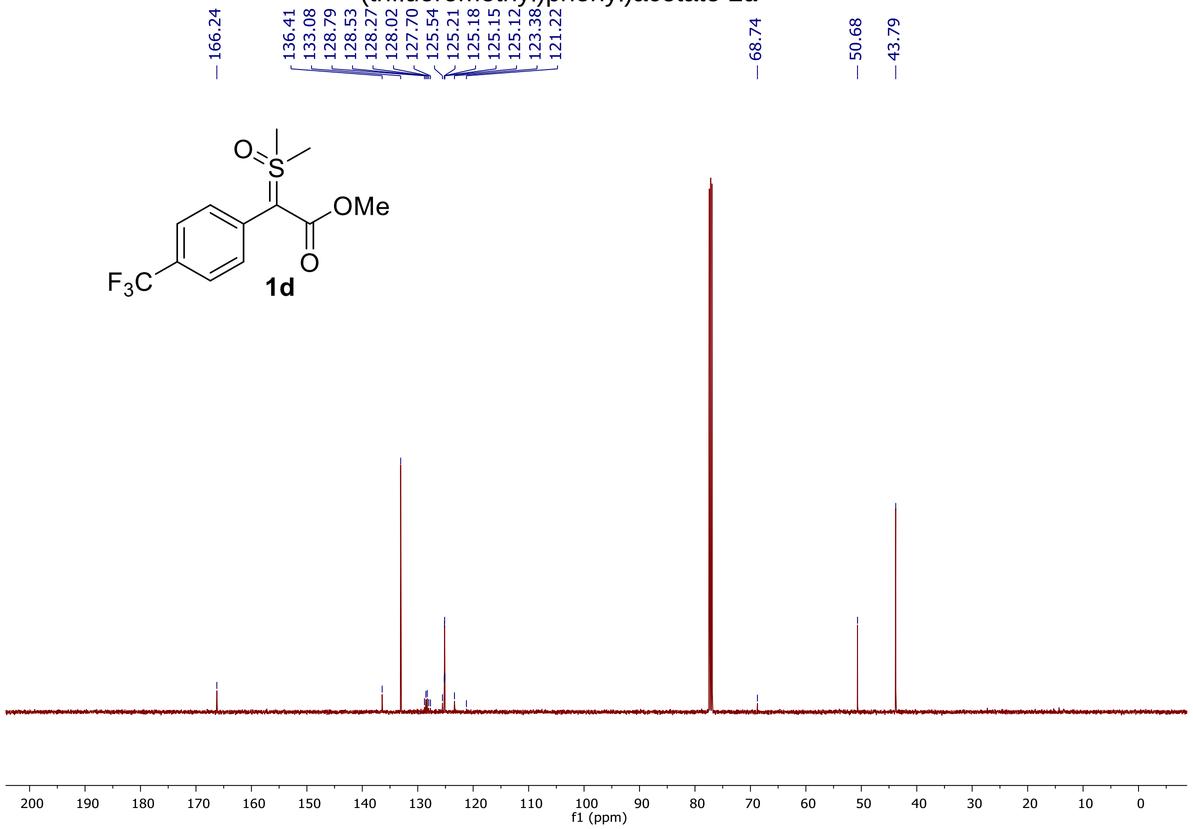

**$^1$H NMR (500 MHz, CDCl$_3$)** methyl 2-(4-chlorophenyl)-2-(dimethyl(oxo)-$\lambda^6$-sulfanylidene)acetate **1e**

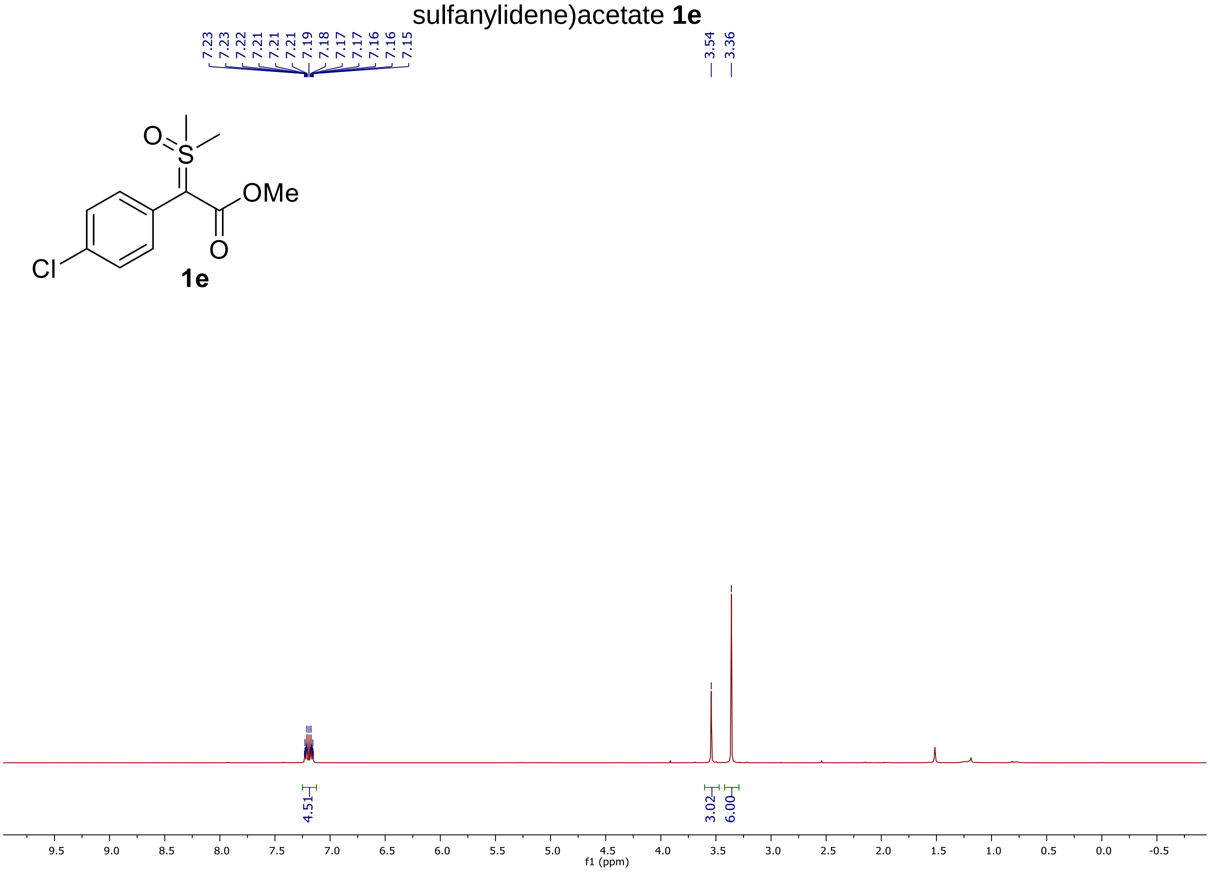


**$^{13}$C NMR (126 MHz, CDCl$_3$)** methyl 2-(4-chlorophenyl)-2-(dimethyl(oxo)-$\lambda^6$-sulfanylidene)acetate **1e**

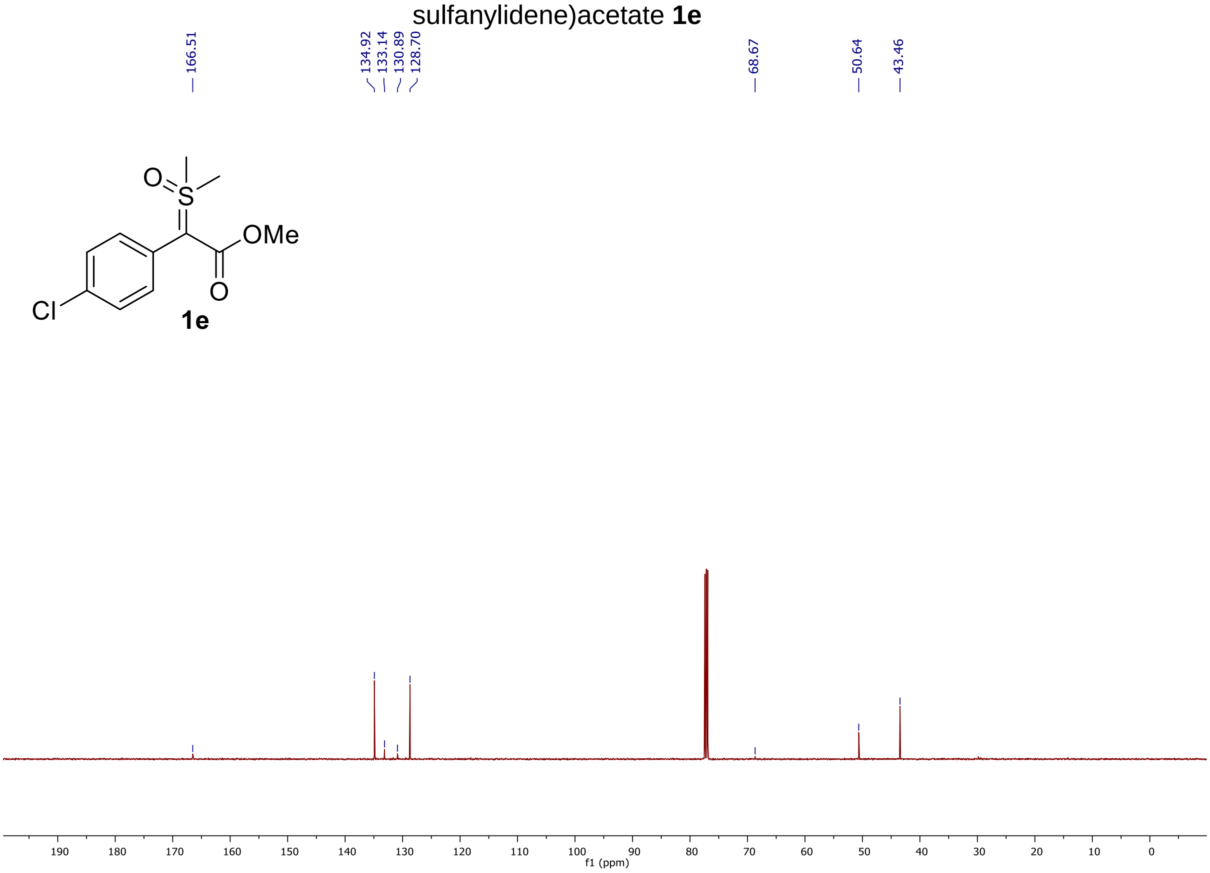

**$^{1}$H NMR (500 MHz, CDCl$_3$)** methyl 2-(dimethyl(oxo)-$\lambda^6$-sulfanylidene)-2-(naphthalen-2-yl)acetate **1f**

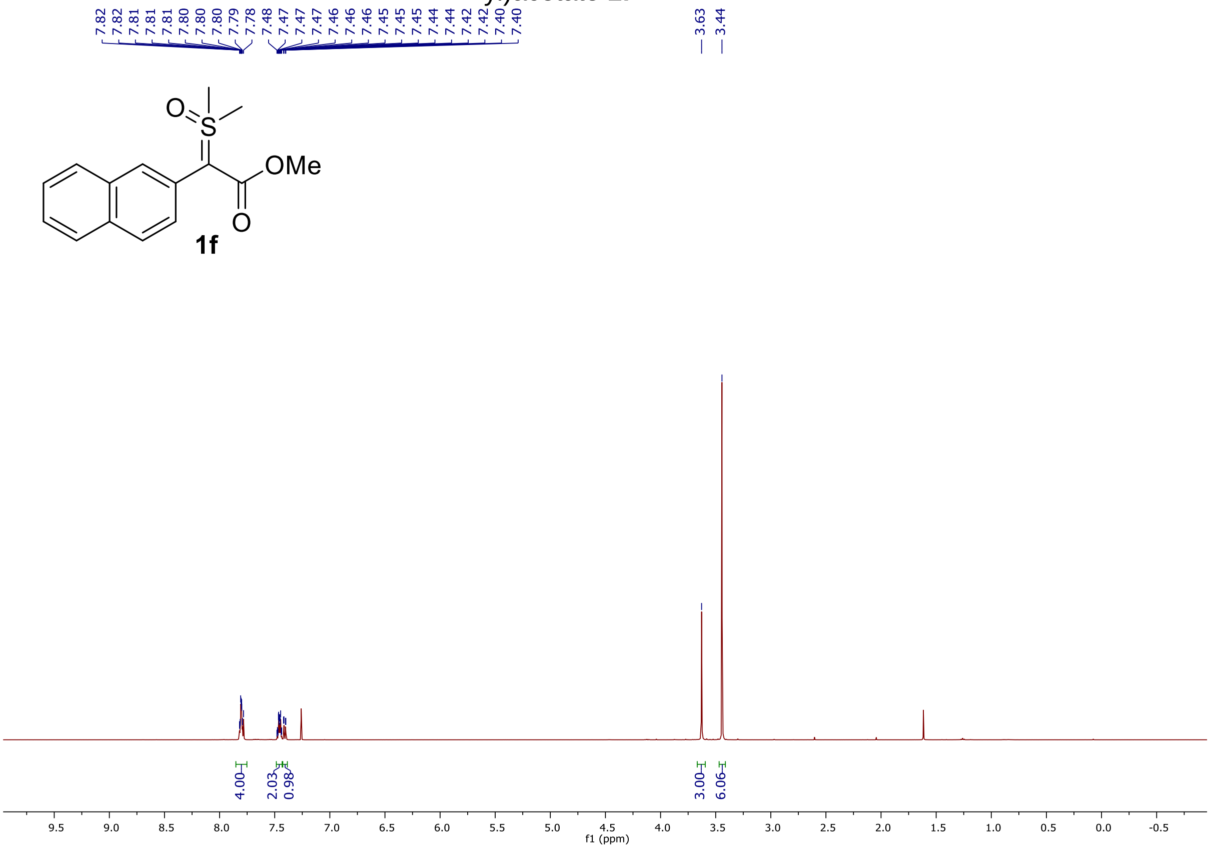


**$^{13}$C NMR (126 MHz, CDCl$_3$)** methyl 2-(dimethyl(oxo)-$\lambda^6$-sulfanylidene)-2-(naphthalen-2-yl)acetate **1f**

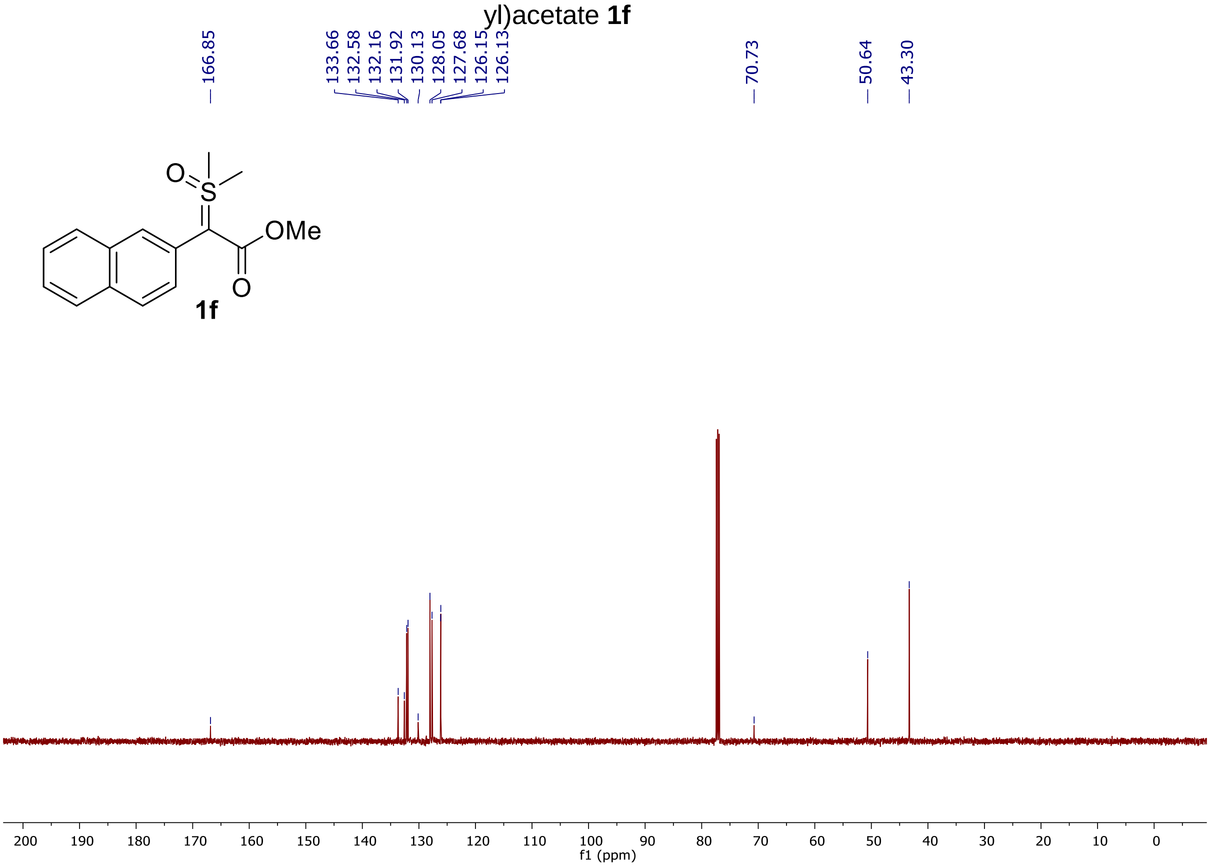

**$^{1}$H NMR (500 MHz, CDCl$_3$)** ethyl 2-(4-bromophenyl)-2-(dimethyl(oxo)- $\lambda^6$-sulfanylidene)acetate **1g**

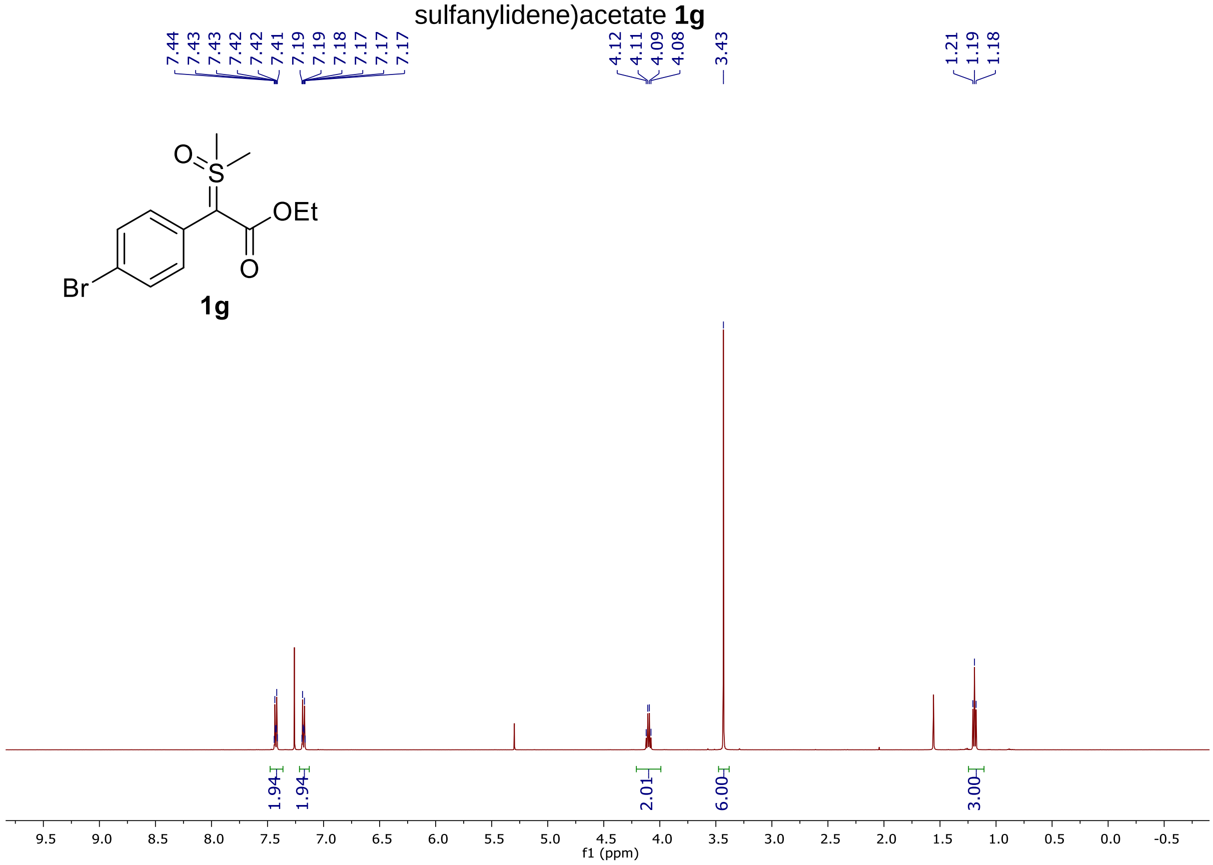


**$^{13}$C NMR (126 MHz, CDCl$_3$)** ethyl 2-(4-bromophenyl)-2-(dimethyl(oxo)- $\lambda^6$-sulfanylidene)acetate **1g**

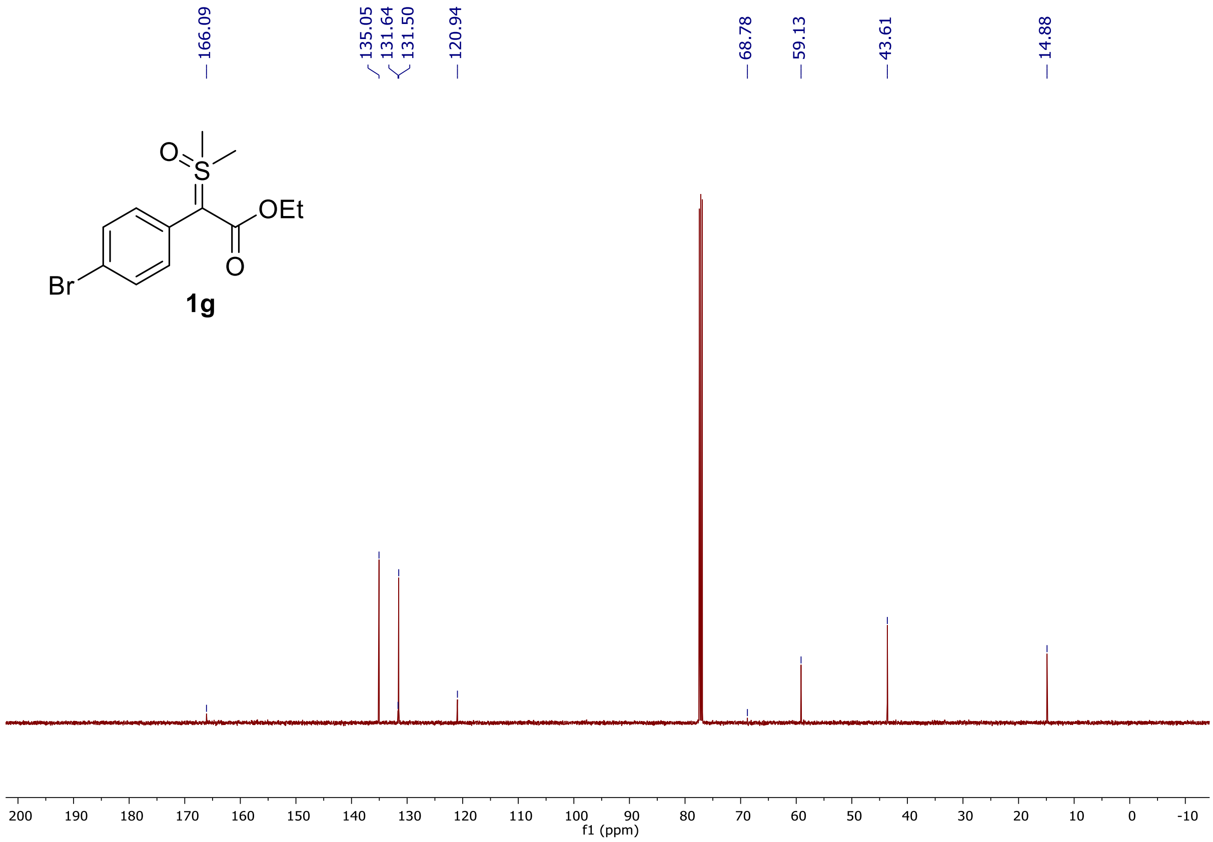

**$^{1}$H NMR (500 MHz, CDCl$_3$)** methyl 2-(dimethyl(oxo)- $\lambda^6$-sulfanylidene)-2-(*p*-tolyl)acetate **1h**

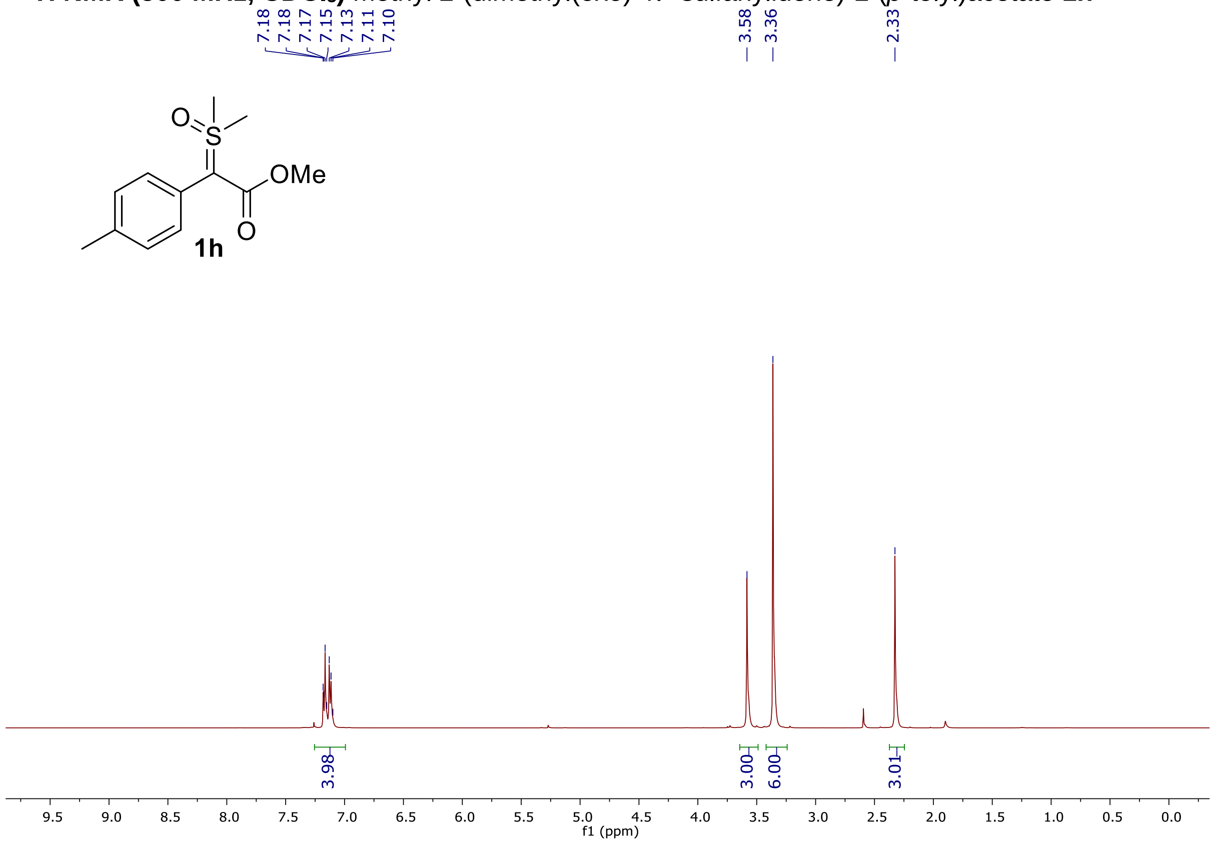


**$^{13}$C NMR (126 MHz, CDCl$_3$)** methyl 2-(dimethyl(oxo)- $\lambda^6$-sulfanylidene)-2-(*p*-tolyl)acetate **1h**

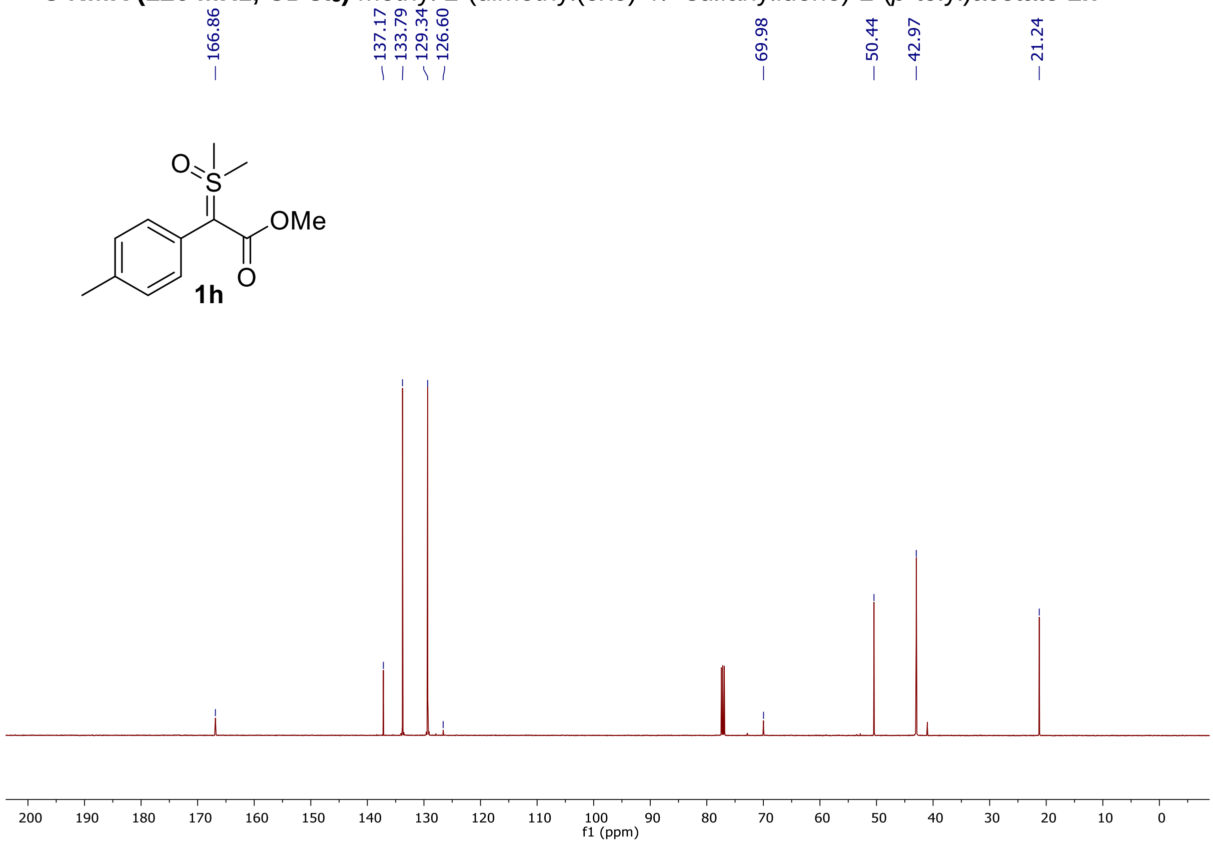

**$^{1}$H NMR (500 MHz, CDCl$_3$)** *tert*-butyl 2-(dimethyl(oxo)- λ$^6$-sulfanylidene)-2-phenylacetate **1i**

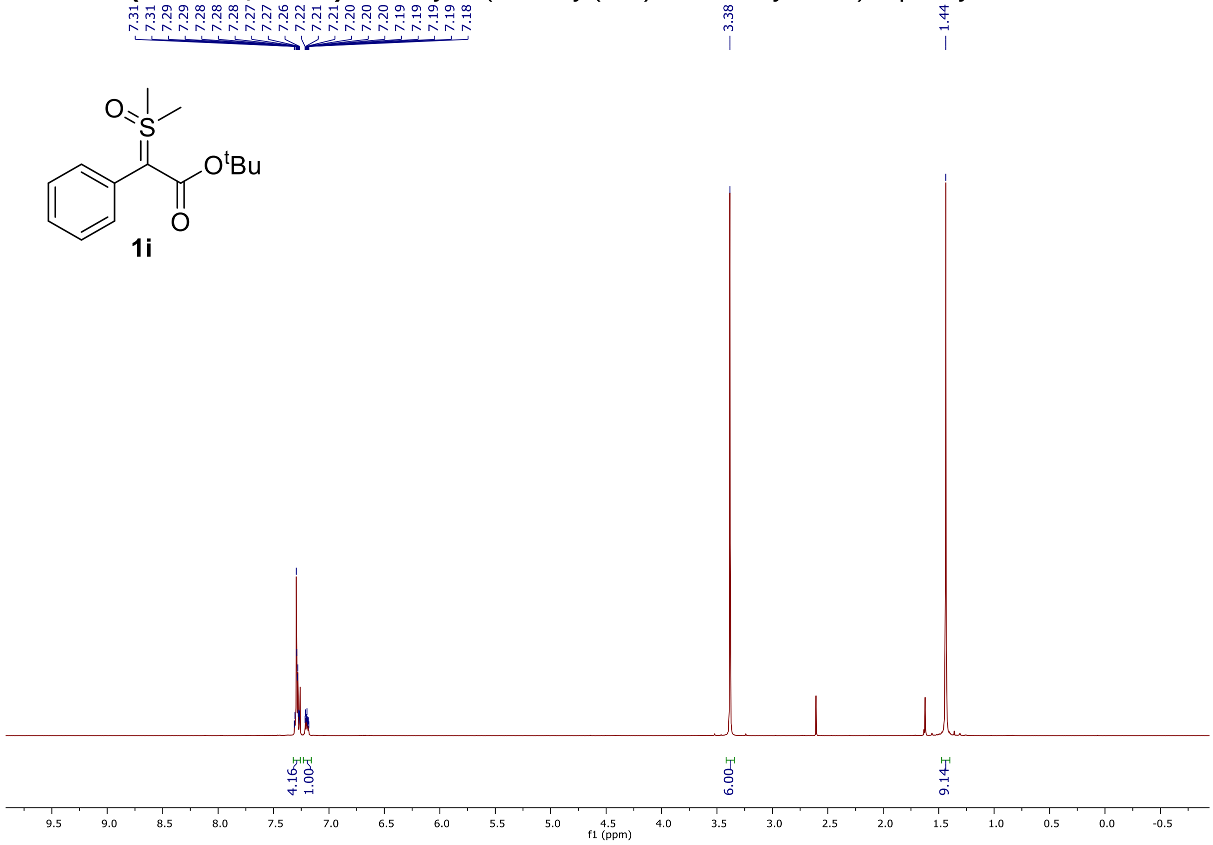


**$^{13}$C NMR (126 MHz, CDCl$_3$)** *tert*-butyl 2-(dimethyl(oxo)- λ6-sulfanylidene)-2-phenylacetate **1i**

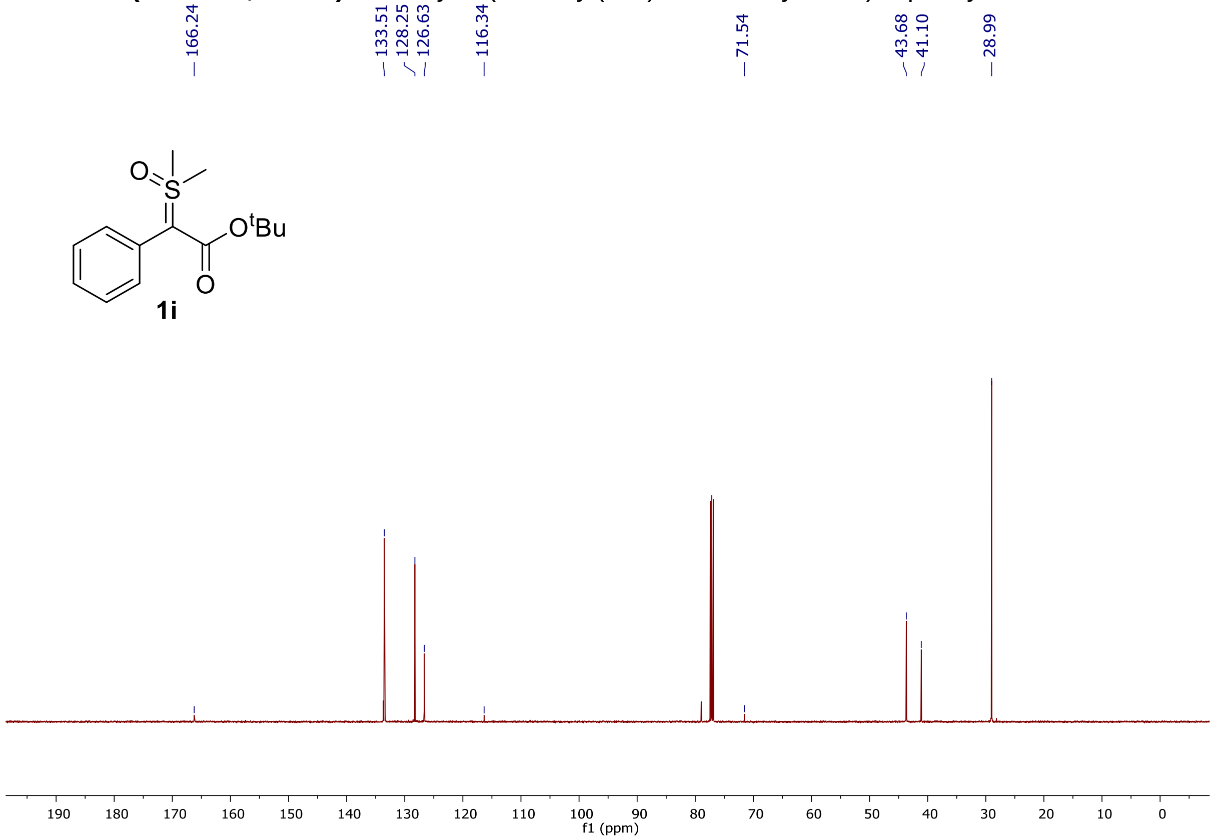

**$^{1}$H NMR (500 MHz, CDCl$_3$)** methyl 2-(dimethyl(oxo)-$\lambda^6$-sulfanylidene)-2-(pyridin-3-yl)acetate
**1j**

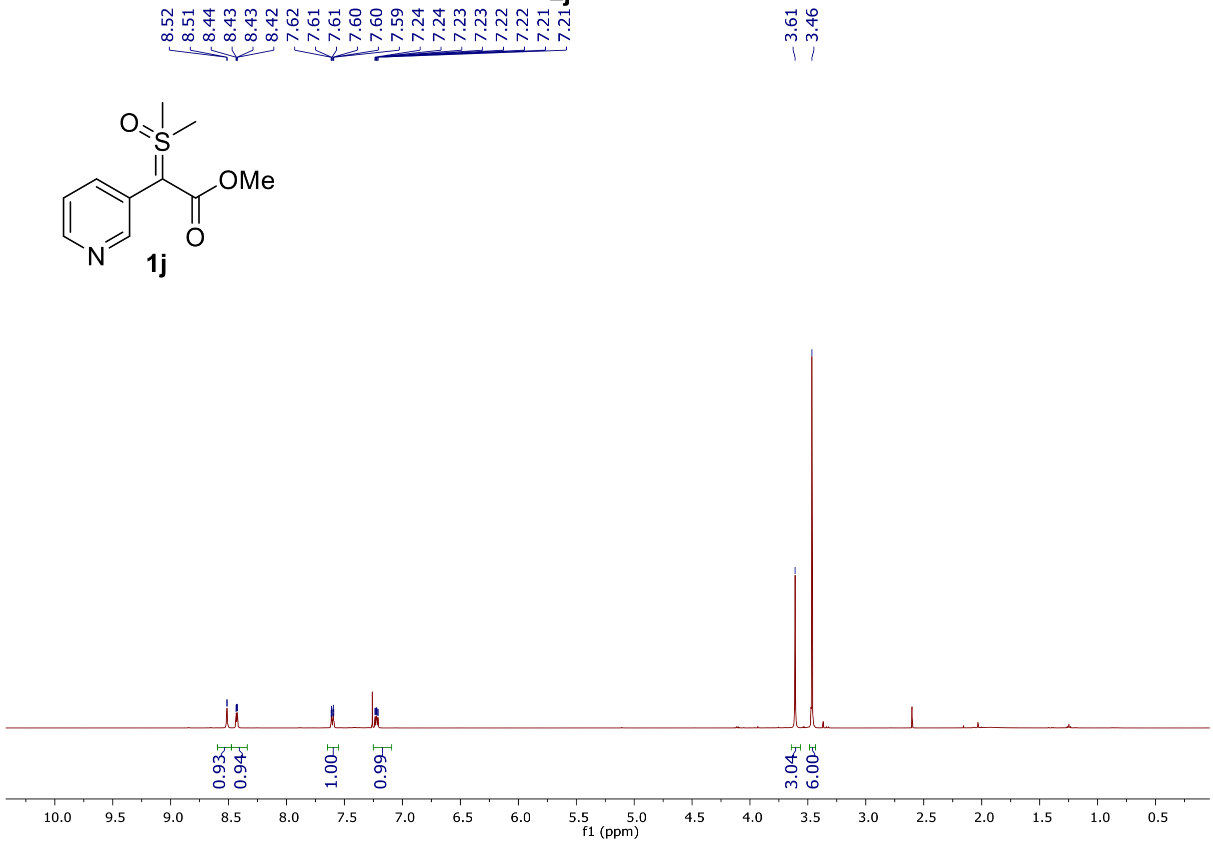


**$^{13}$C NMR (126 MHz, CDCl$_3$)** methyl 2-(dimethyl(oxo)-$\lambda^6$-sulfanylidene)-2-(pyridin-3-yl)acetate
**1j**

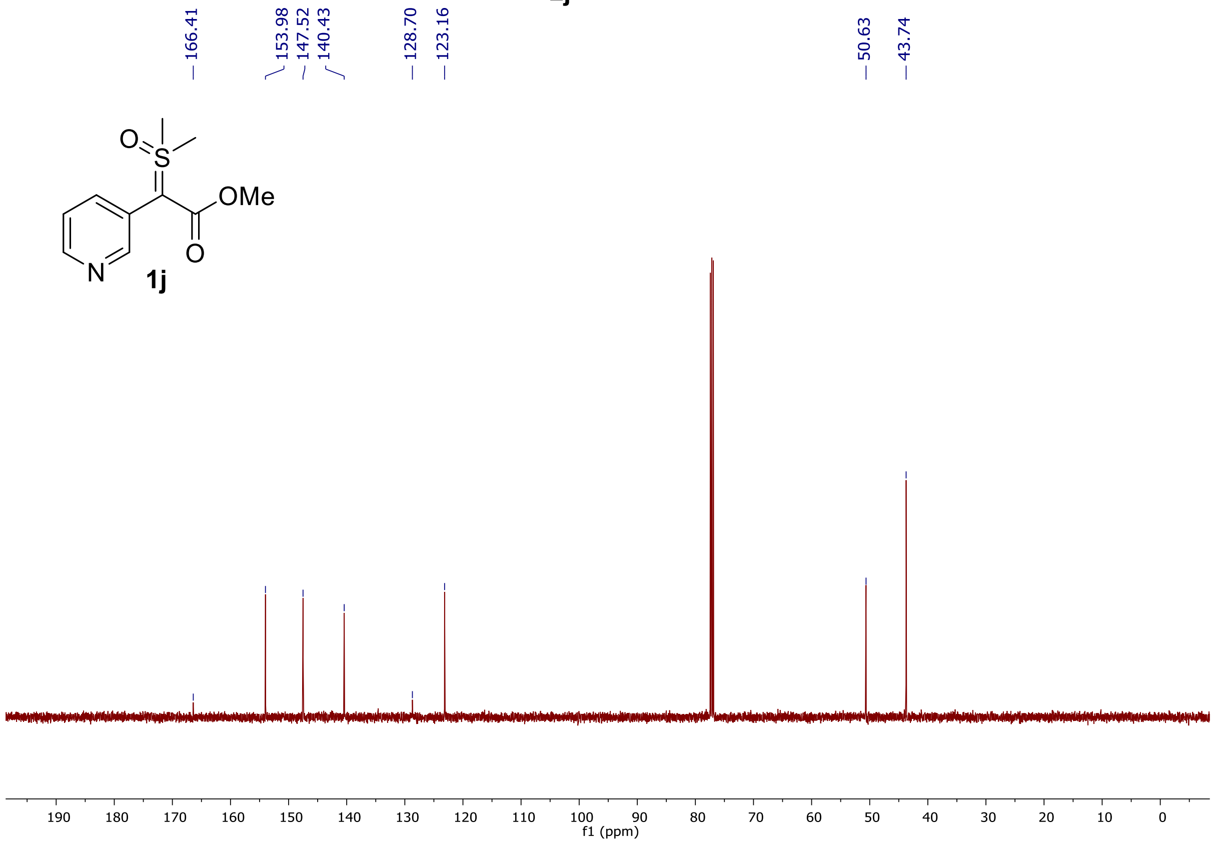

**$^{1}$H NMR (500 MHz, CDCl$_3$)** 2,2,2-trichloroethyl 2-(dimethyl(oxo)-λ$^6$-sulfaneylidene)-2-phenylacetate **1k**

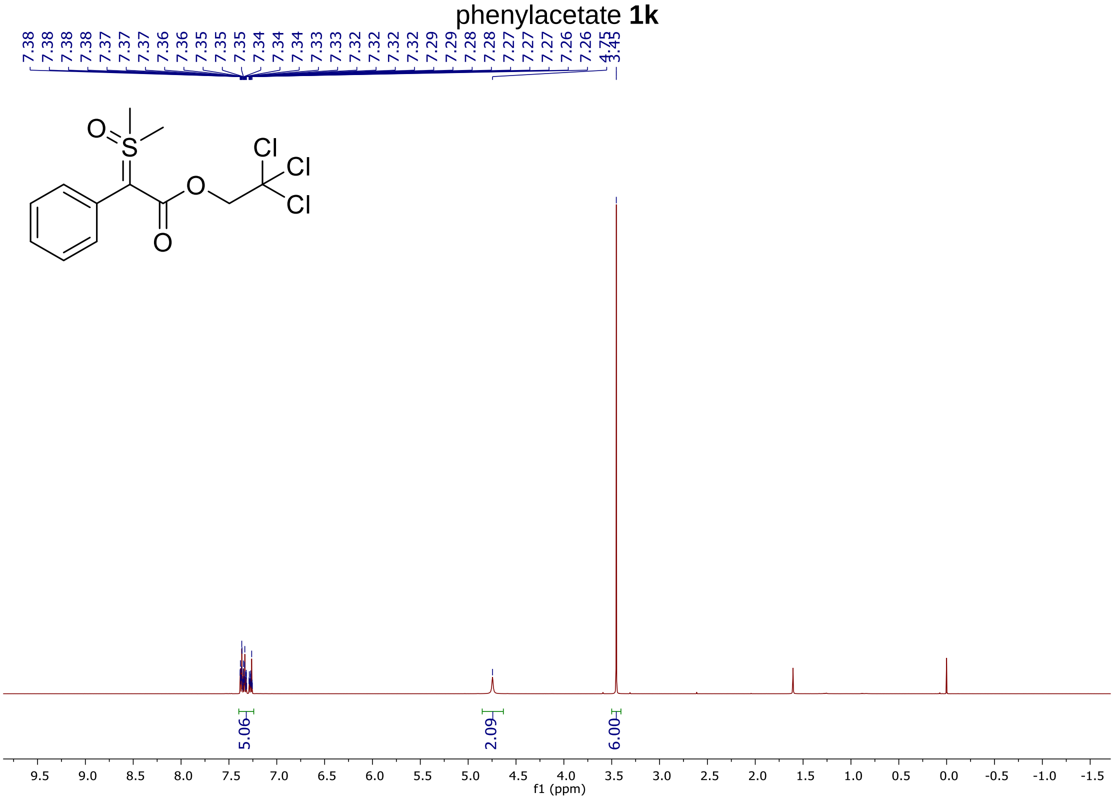


**$^{13}$C NMR (126 MHz, CDCl$_3$)** 2,2,2-trichloroethyl 2-(dimethyl(oxo)-λ$^6$-sulfaneylidene)-2-phenylacetate **1k**

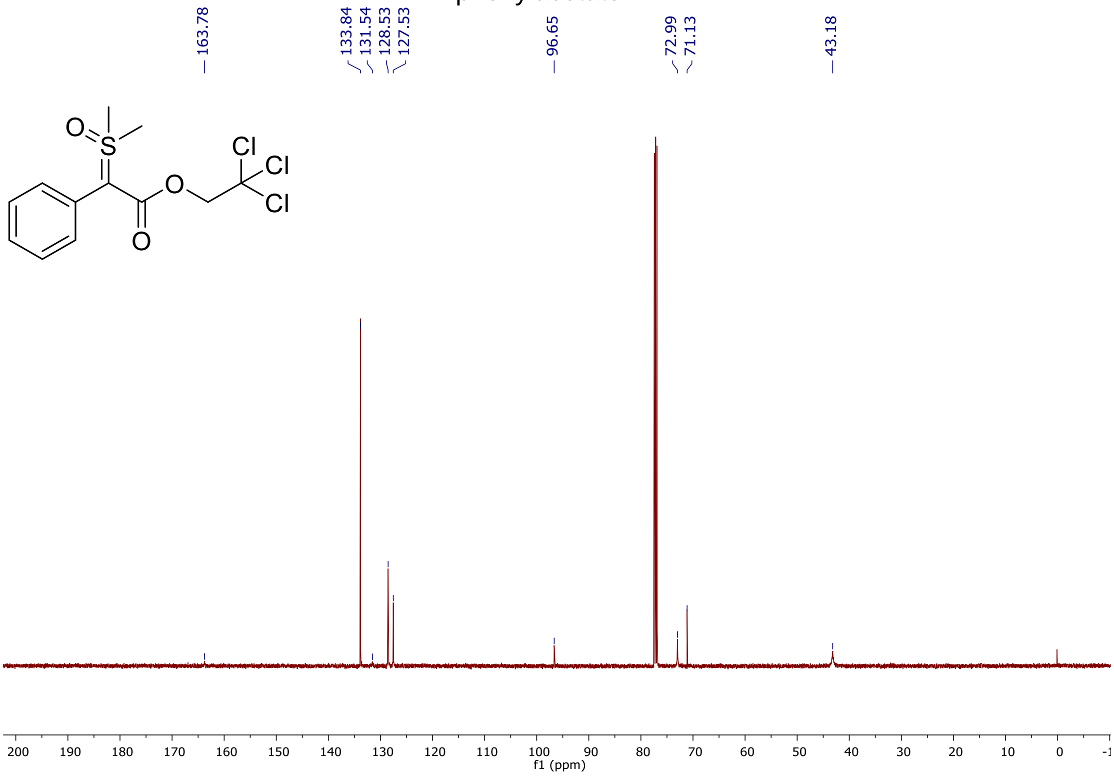

**$^1$H NMR (500 MHz, CDCl$_3$)** methyl 2-phenyl-2-(phenylthio)acetate **3a**

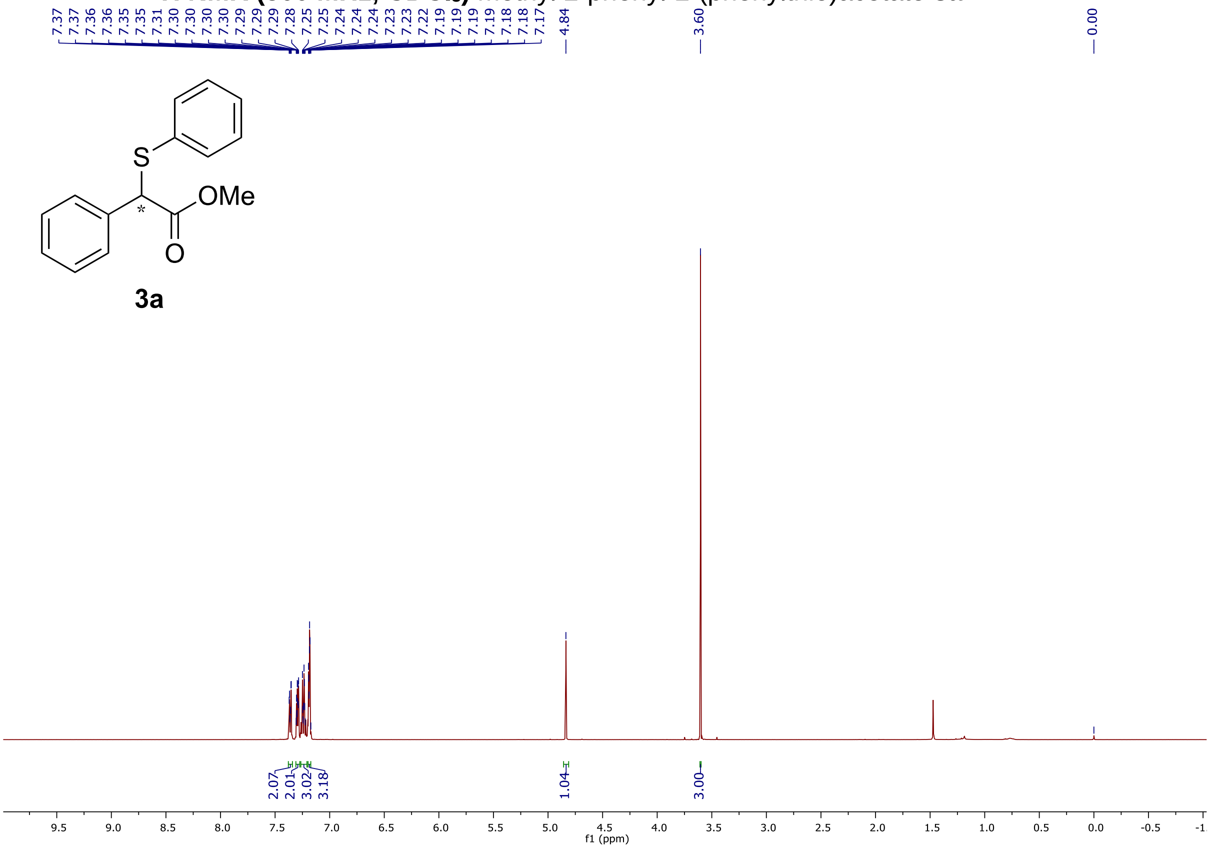


**$^{13}$C NMR (126 MHz, CDCl$_3$)** methyl 2-phenyl-2-(phenylthio)acetate **3a**

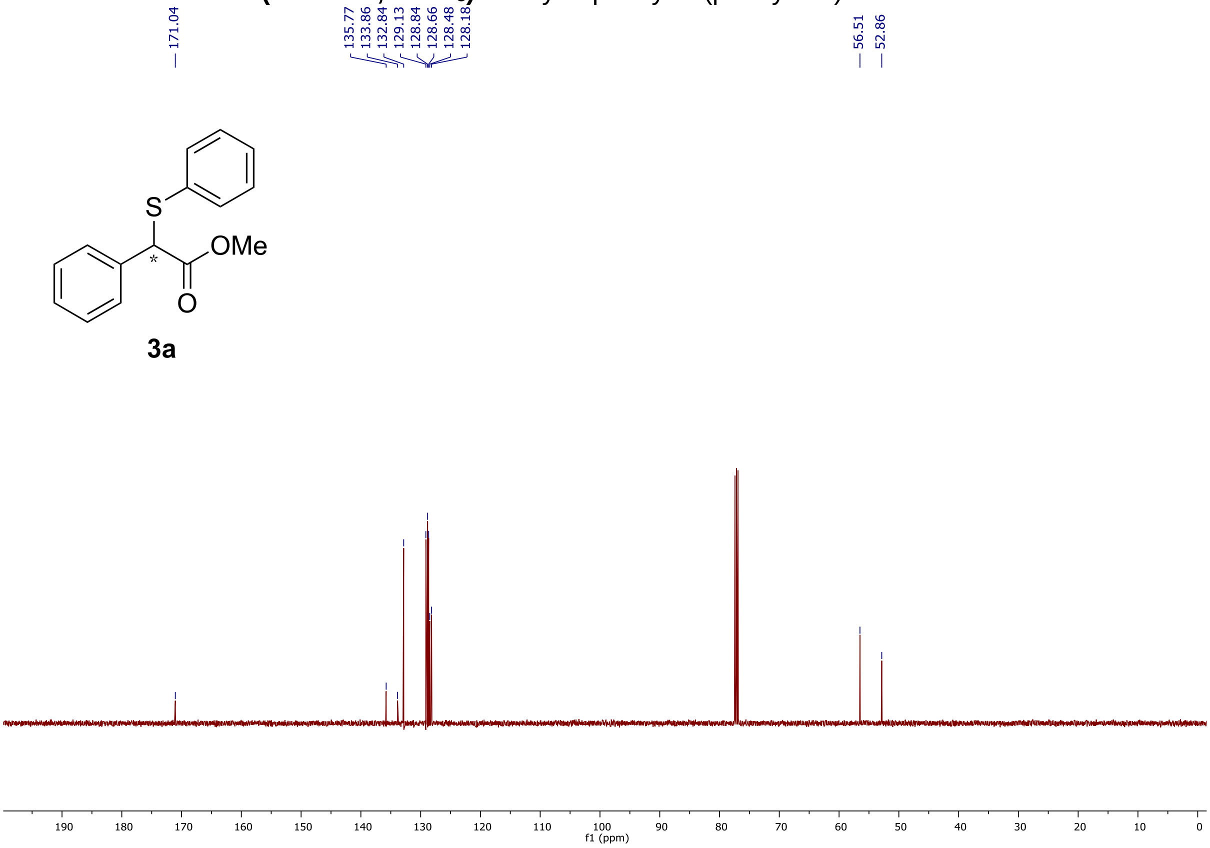

**$^{1}$H NMR (500 MHz, CDCl$_3$)** methyl 2-((4-nitrophenyl)thio)-2-phenylacetate **3b**

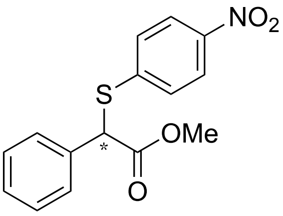

**3b**

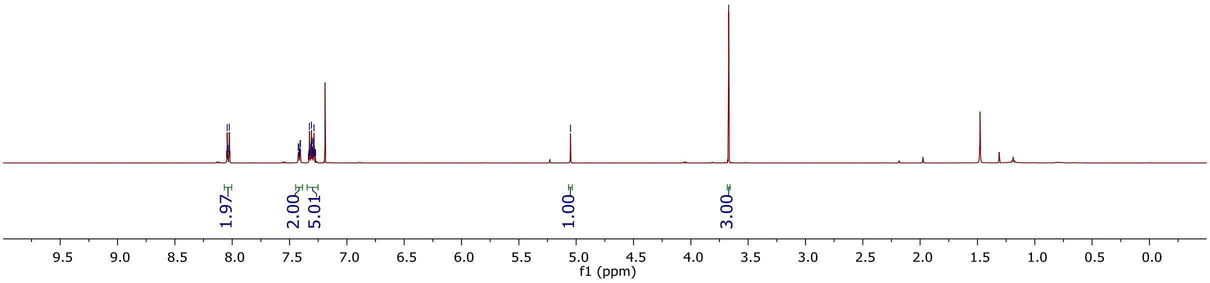


**$^{13}$C NMR (126 MHz, CDCl$_3$)** methyl 2-((4-nitrophenyl)thio)-2-phenylacetate **3b**

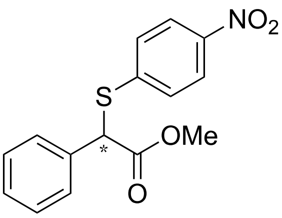

**3b**

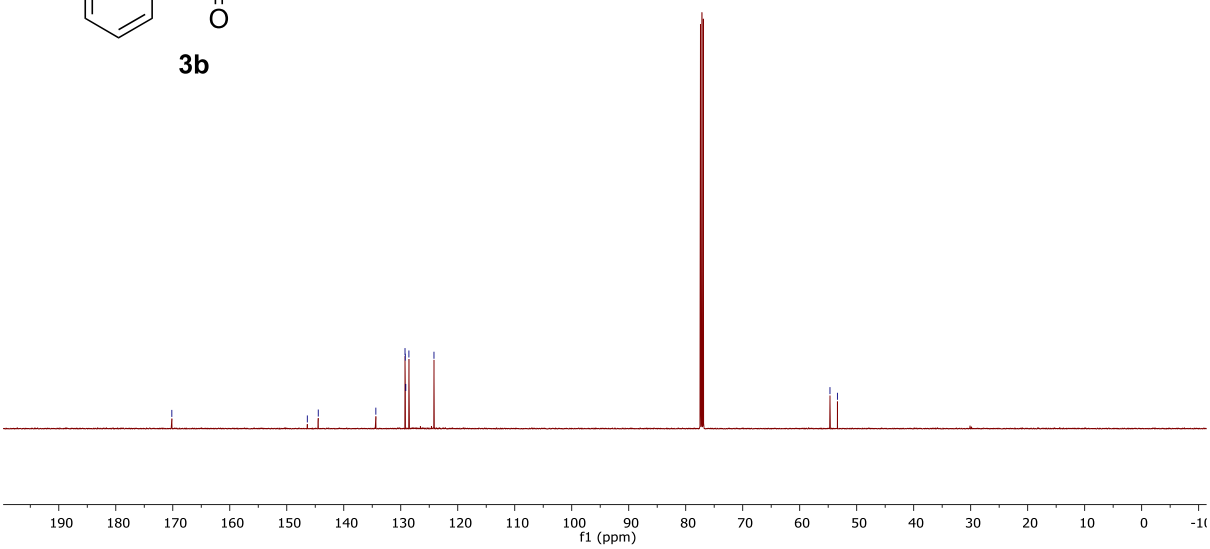

**$^{1}$H NMR (500 MHz, CDCl$_3$)** methyl 2-((4-bromophenyl)thio)-2-phenylacetate **3c**

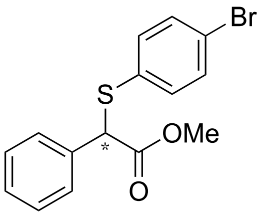

**3c**

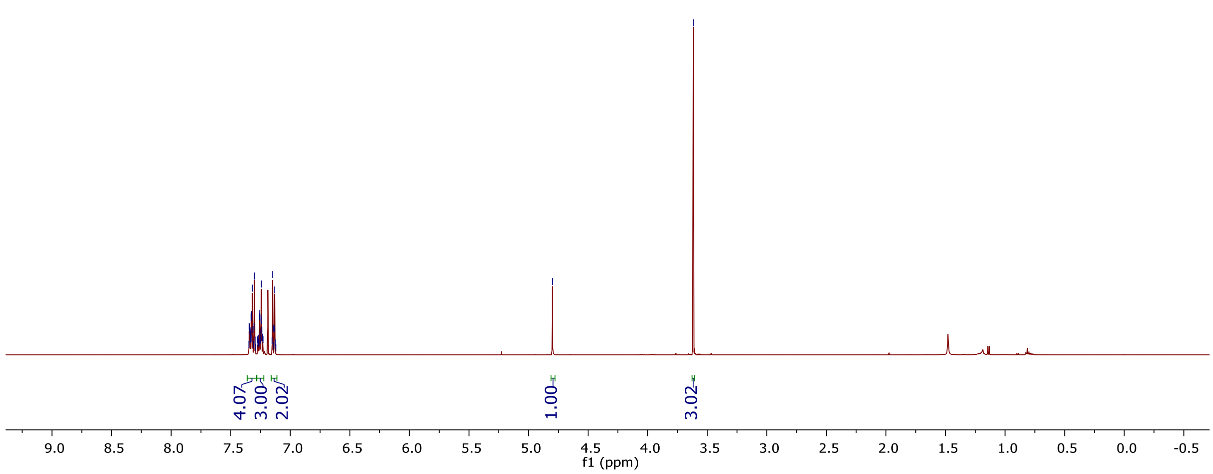


**$^{13}$C NMR (126 MHz, CDCl$_3$)** methyl 2-((4-bromophenyl)thio)-2-phenylacetate **3c**

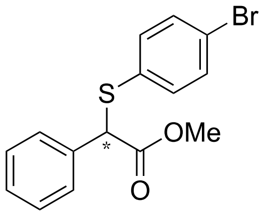

**3c**

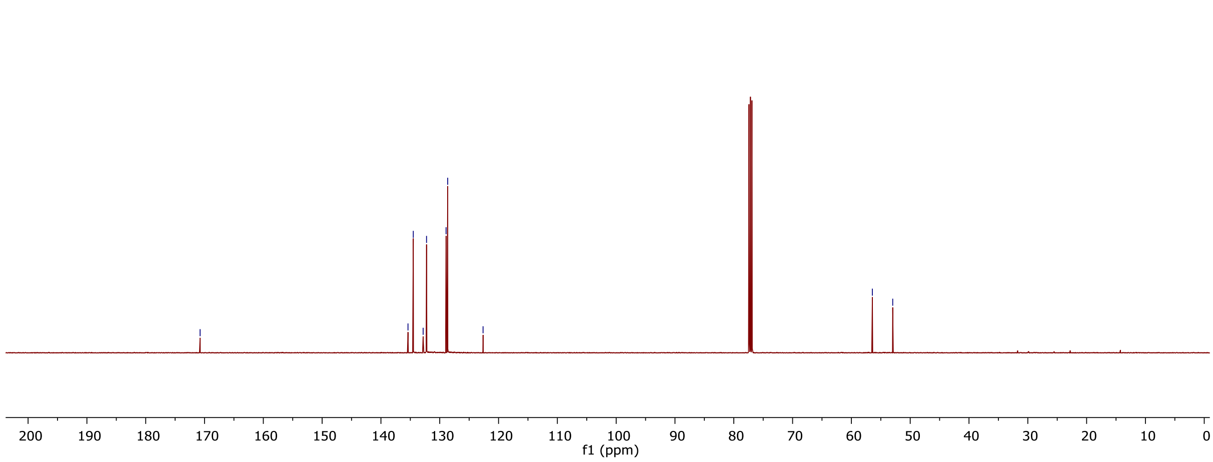

**$^{1}$H NMR (500 MHz, CDCl$_3$)** methyl 2-((4-chlorophenyl)thio)-2-phenylacetate **3d**

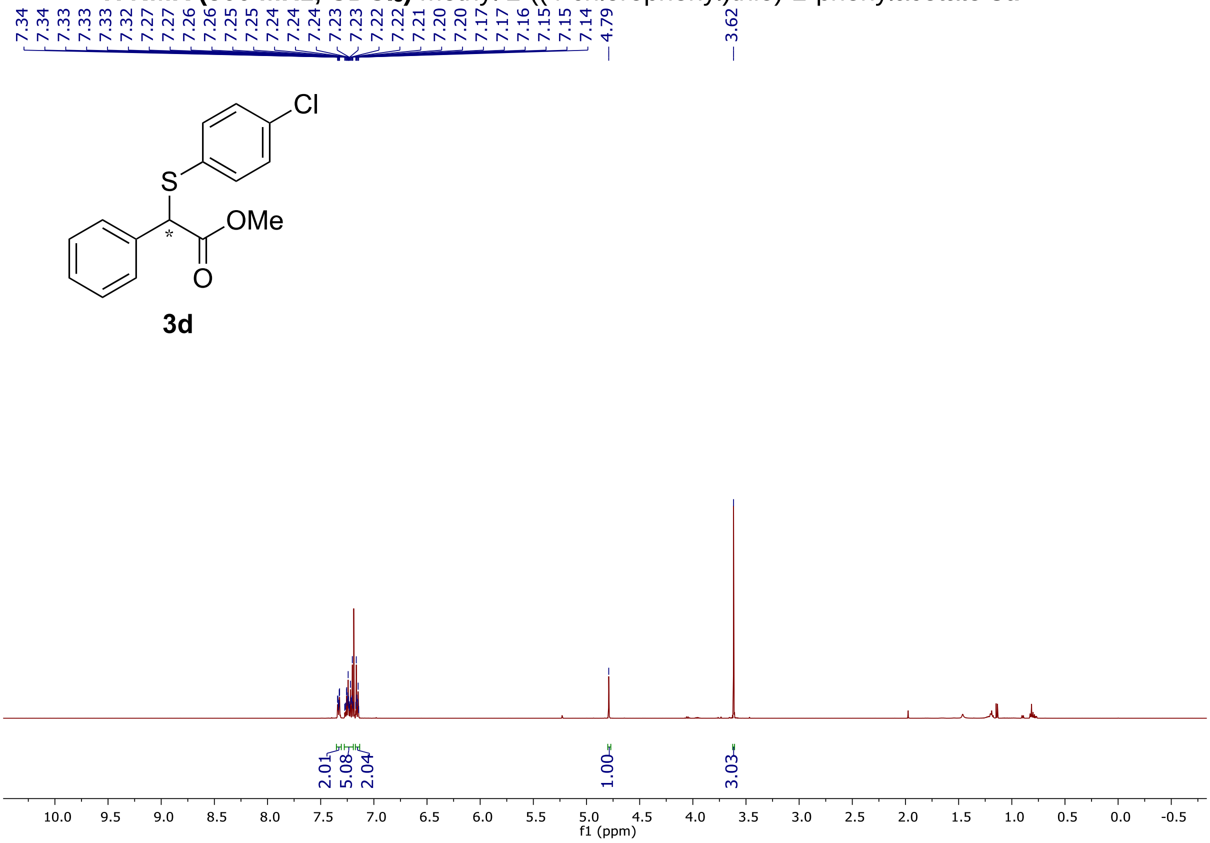


**$^{13}$C NMR (126 MHz, CDCl$_3$)** methyl 2-((4-chlorophenyl)thio)-2-phenylacetate **3d**

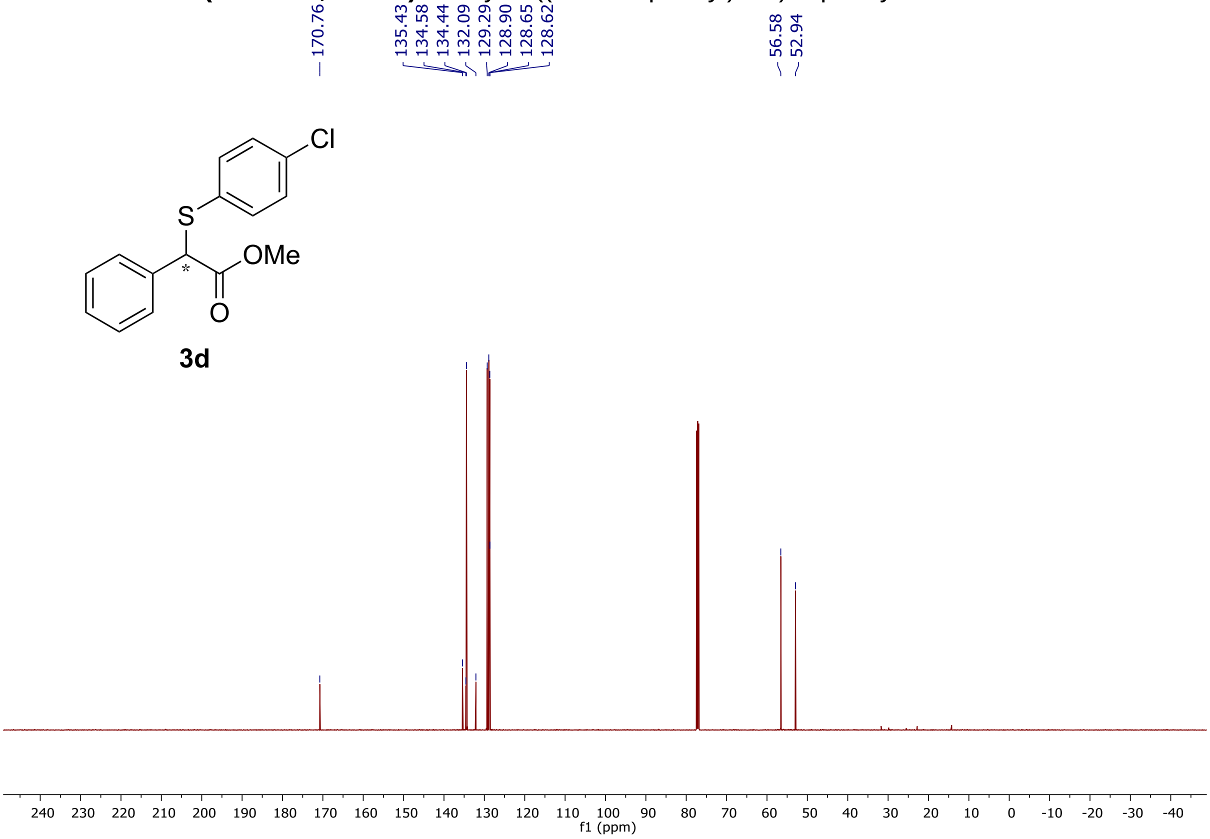

**$^{1}$H NMR (500 MHz, CDCl$_3$)** methyl 2-((2-chlorophenyl)thio)-2-phenylacetate **3e**

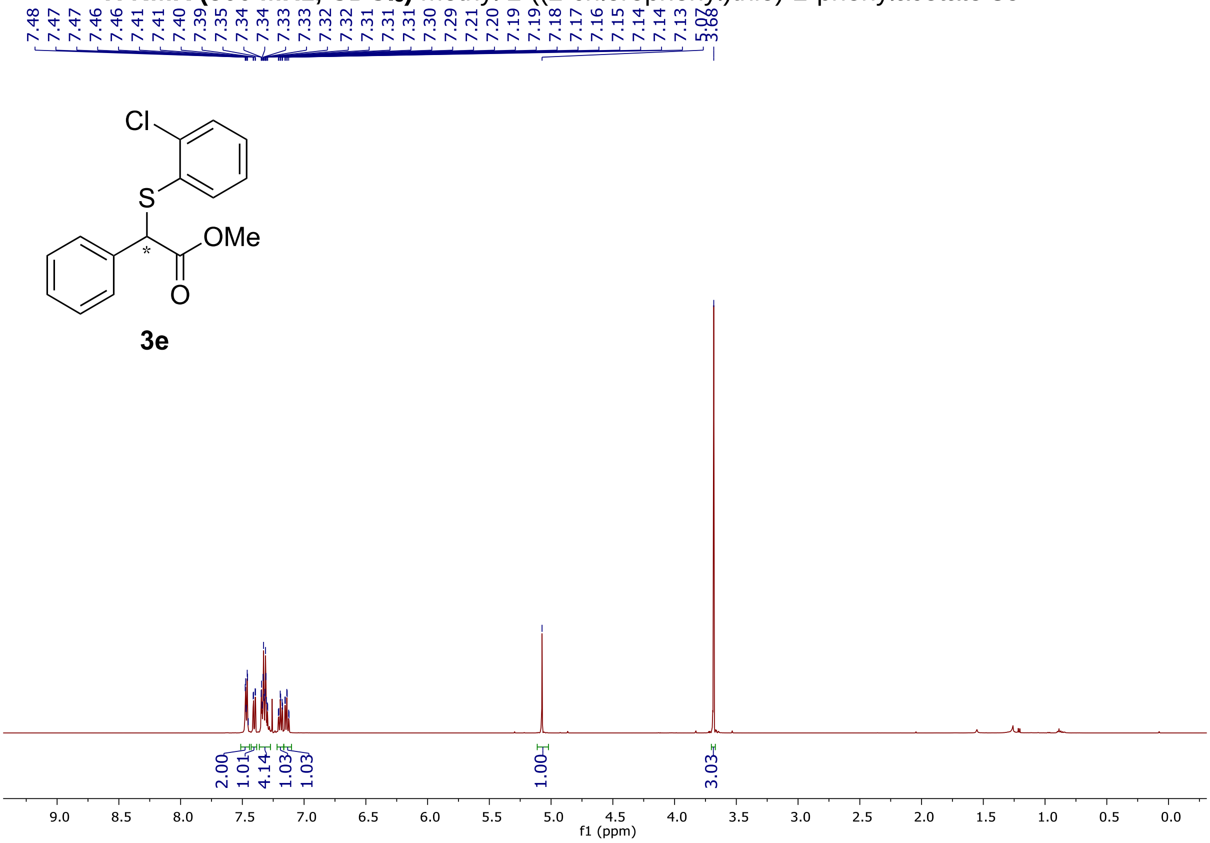


**$^{13}$C NMR (126 MHz, CDCl$_3$)** methyl 2-((2-chlorophenyl)thio)-2-phenylacetate **3e**

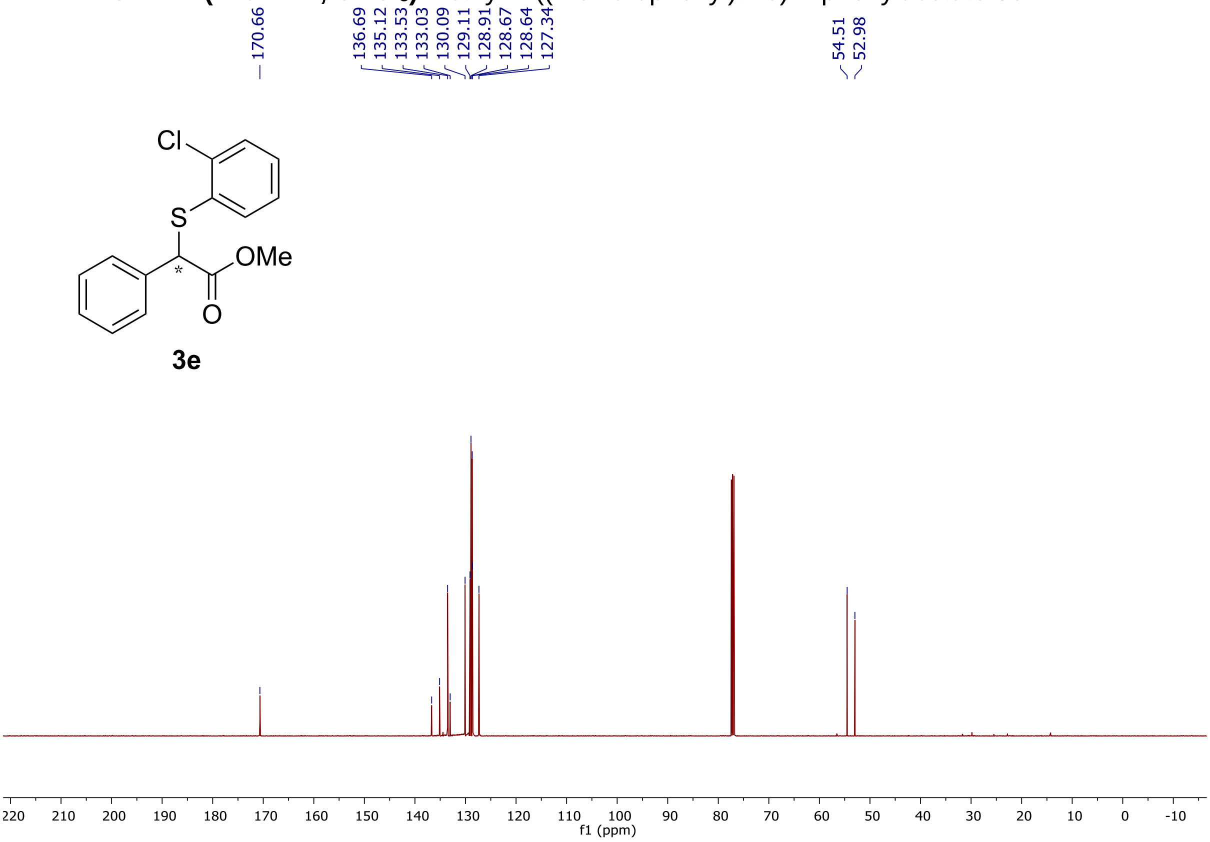

**$^{1}$H NMR (500 MHz, CDCl$_3$)** methyl 2-((3-chlorophenyl)thio)-2-phenylacetate **3f**

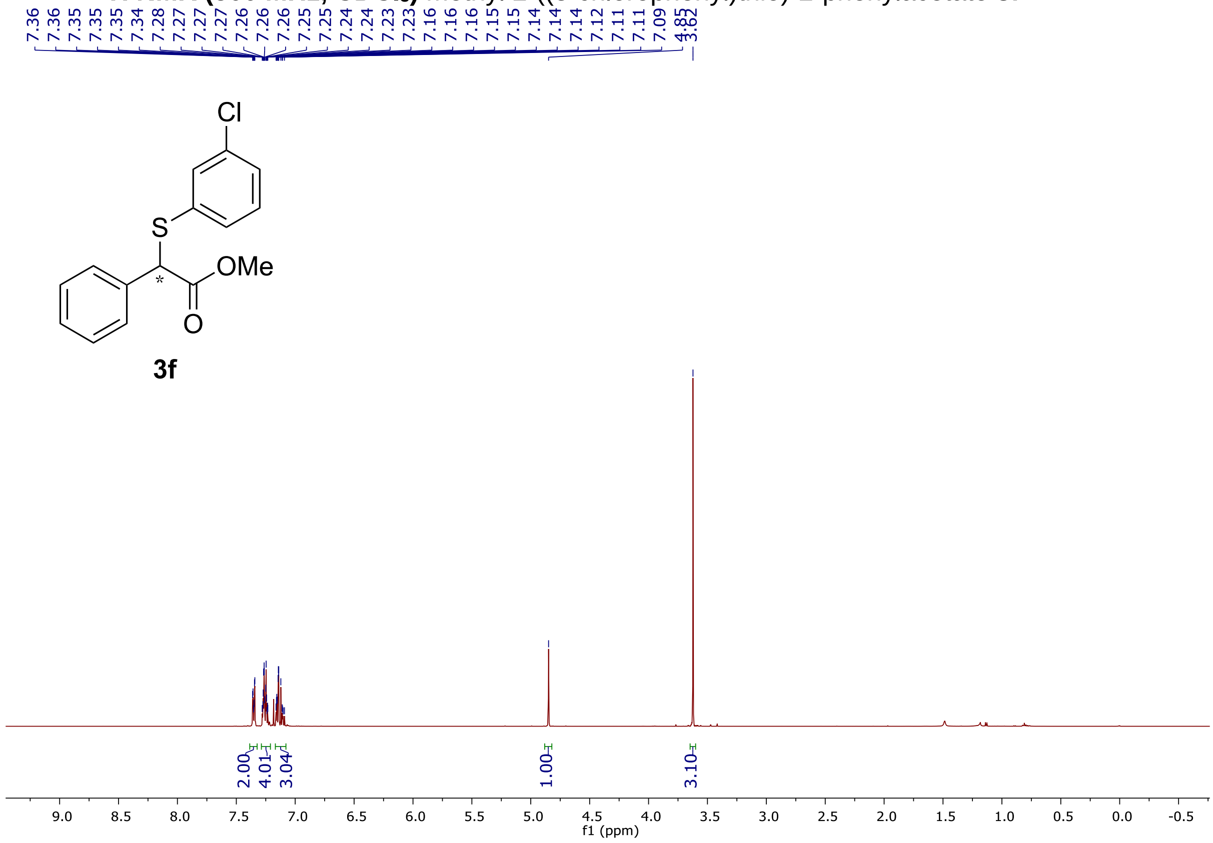


**$^{13}$C NMR (126 MHz, CDCl$_3$)** methyl 2-((3-chlorophenyl)thio)-2-phenylacetate **3f**

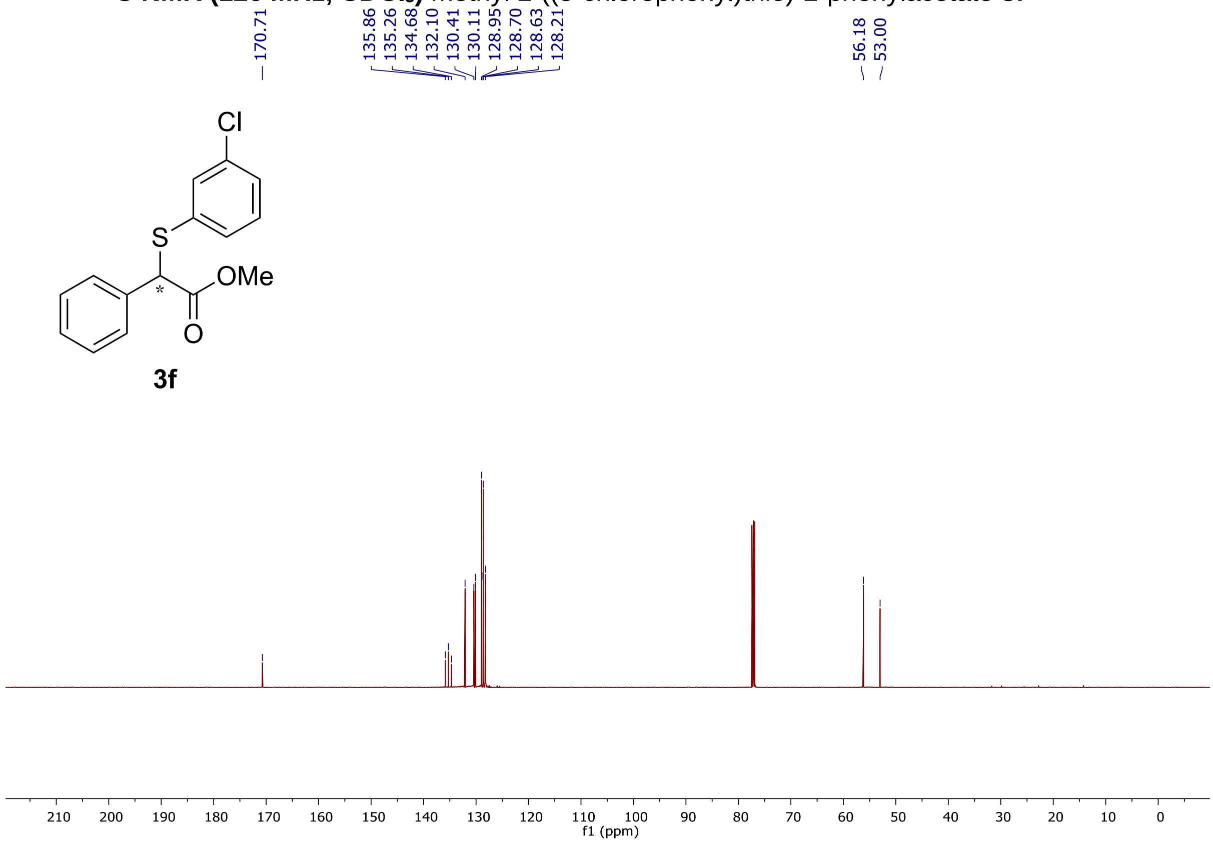

**$^{1}$H NMR (500 MHz, CDCl$_3$)** methyl 2-((4-methoxyphenyl)thio)-2-phenylacetate **3g**

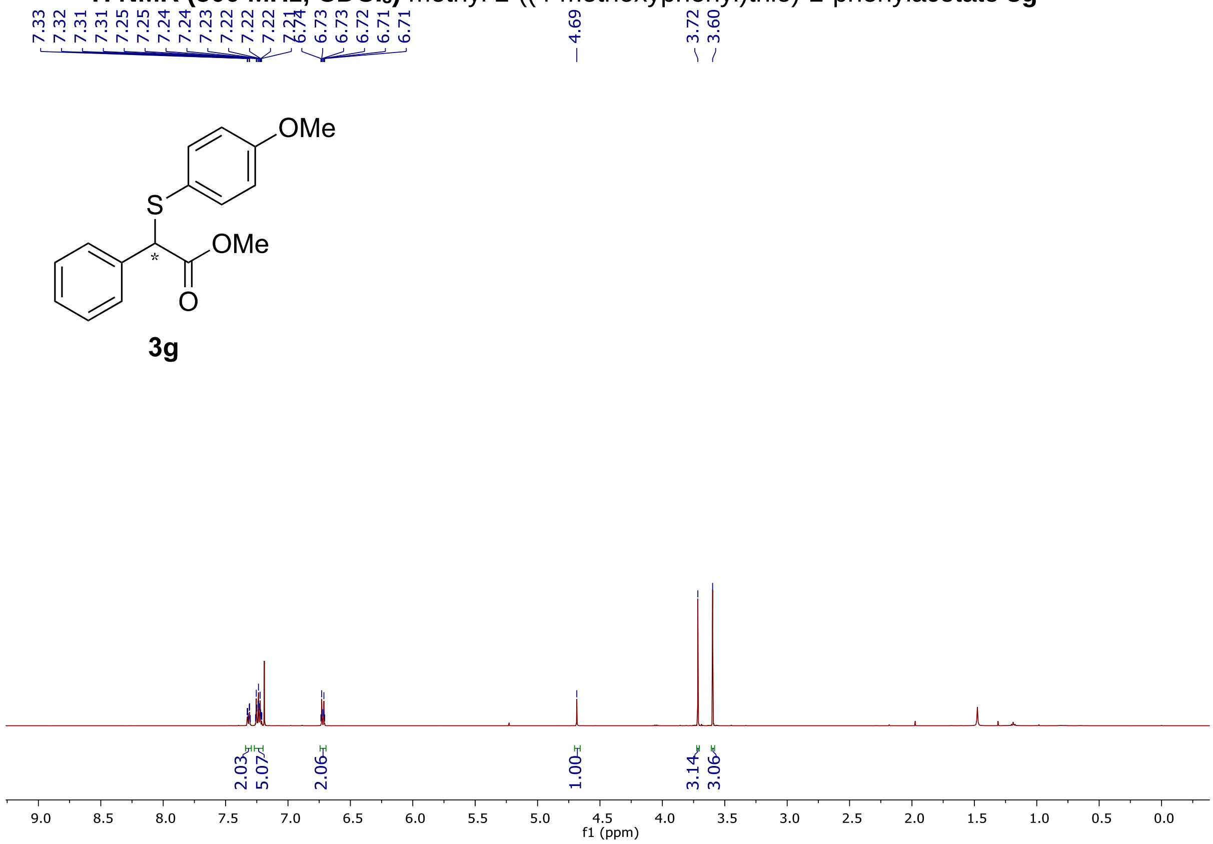


**$^{13}$C NMR (126 MHz, CDCl$_3$)** methyl 2-((4-methoxyphenyl)thio)-2-phenylacetate **3g**

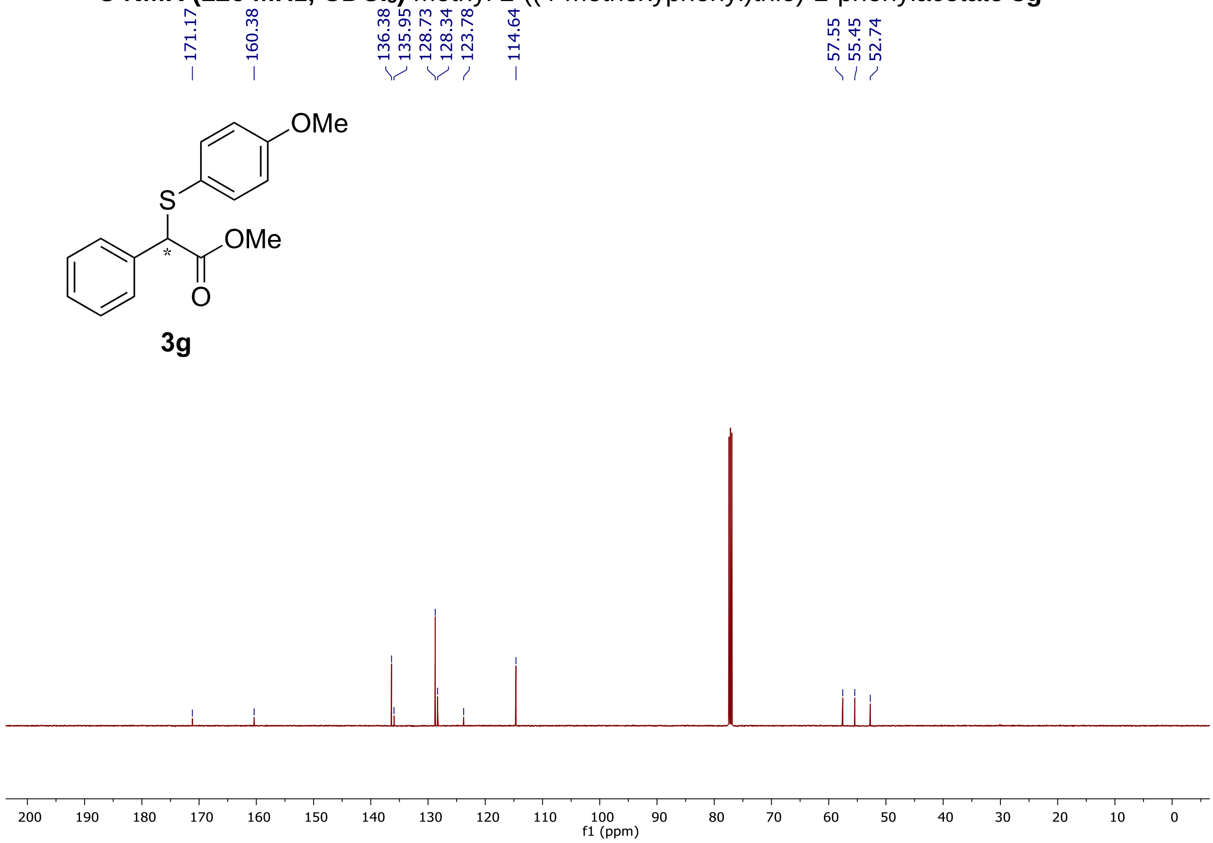

**$^{1}$H NMR (500 MHz, CDCl$_3$)** methyl 2-((2-methoxyphenyl)thio)-2-phenylacetate **3h**

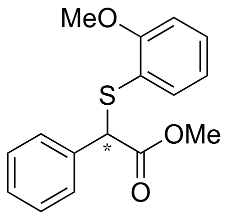


**3h**

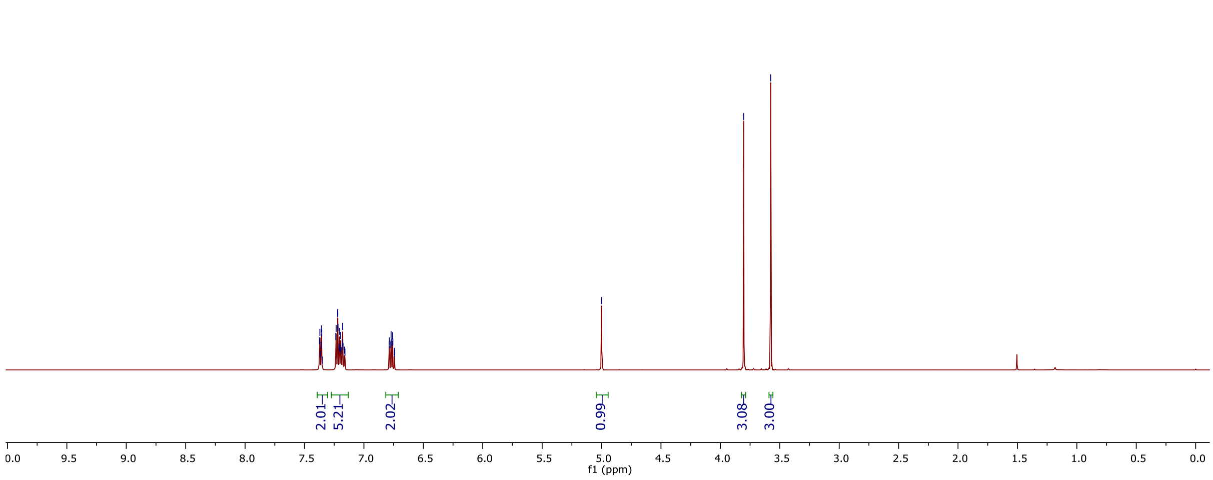


**$^{13}$C NMR (126 MHz, CDCl$_3$)** methyl 2-((2-methoxyphenyl)thio)-2-phenylacetate **3h**

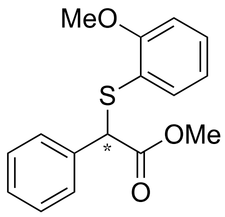


**3h**

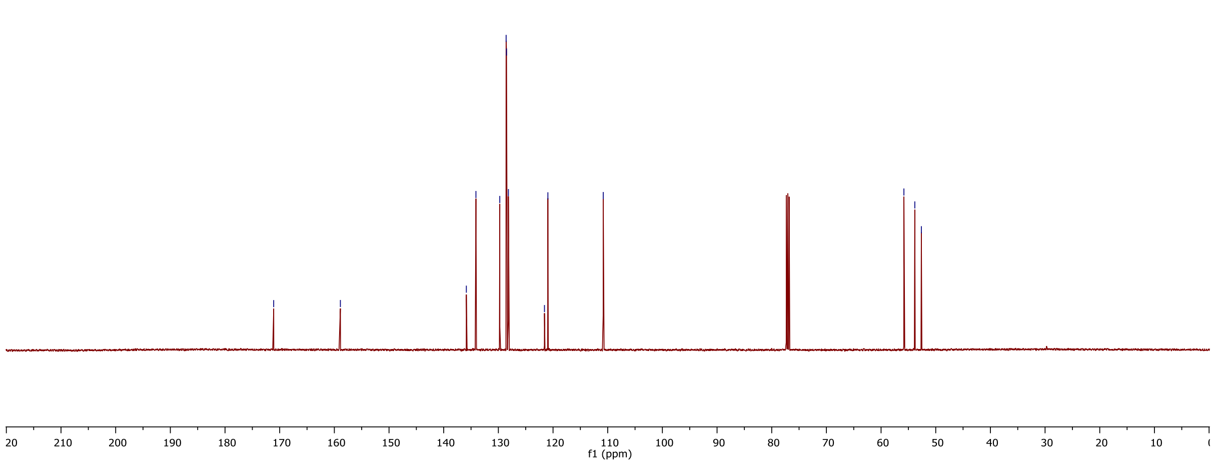

**$^{1}H$ NMR (500 MHz, $CDCl_3$)** methyl 2-phenyl-2-(*p*-tolylthio)acetate **3i**

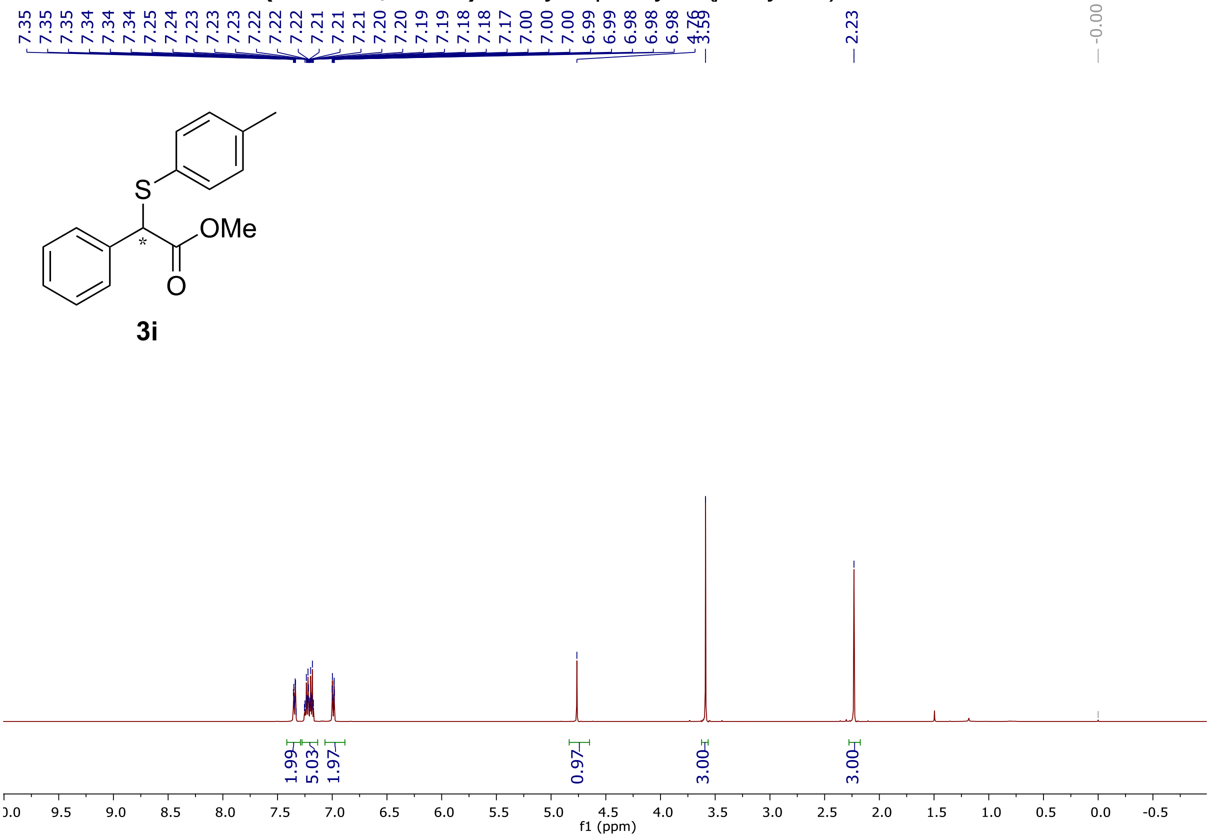


**$^{13}C$ NMR (126 MHz, $CDCl_3$)** methyl 2-phenyl-2-(*p*-tolylthio)acetate **3i**

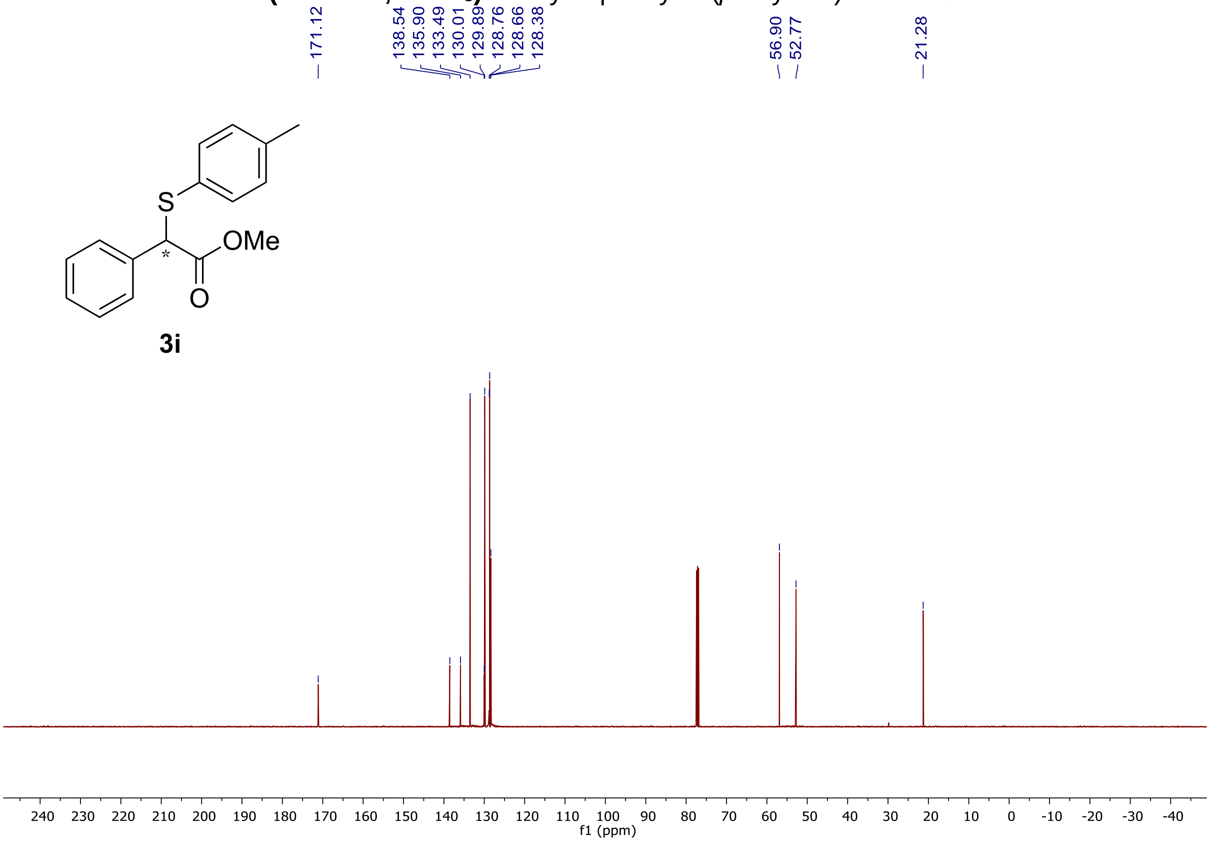

**$^1$H NMR (500 MHz, $CDCl_3$)** methyl 2-phenyl-2-(*o*-tolylthio)acetate **3j**

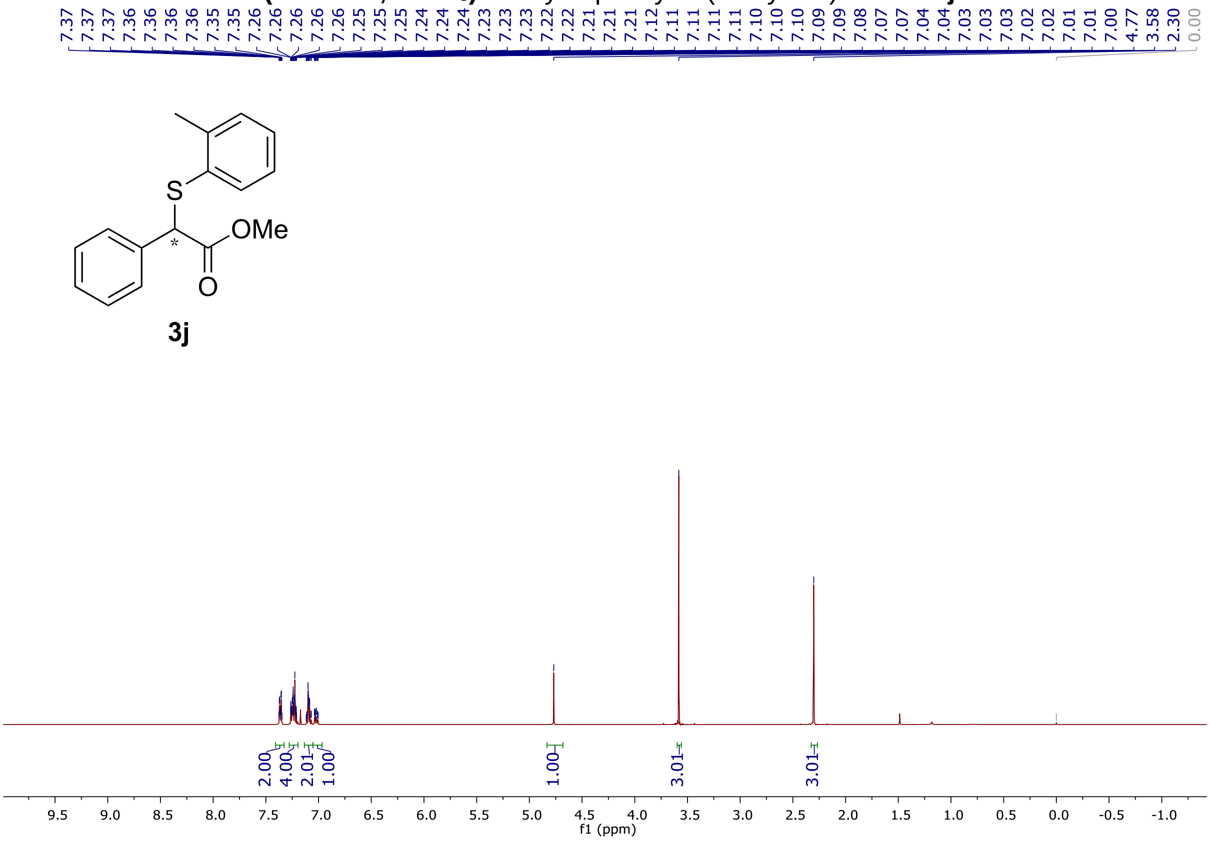


**$^{13}$C NMR (126 MHz, $CDCl_3$)** methyl 2-phenyl-2-(*o*-tolylthio)acetate **3j**

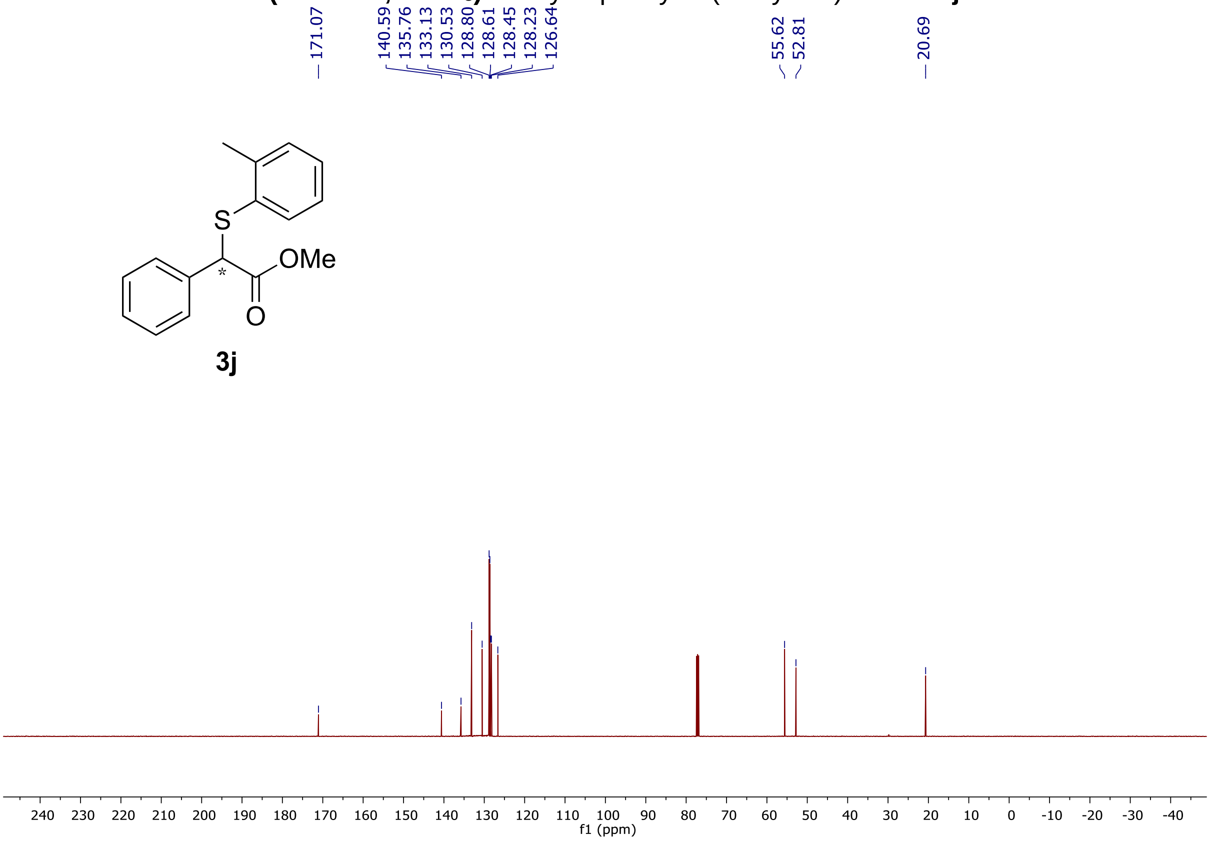

**$^{1}$H NMR (500 MHz, CDCl$_3$)** methyl 2-((2,6-dimethylphenyl)thio)-2-phenylacetate **3k**

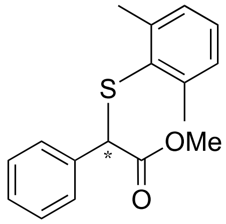


**3k**

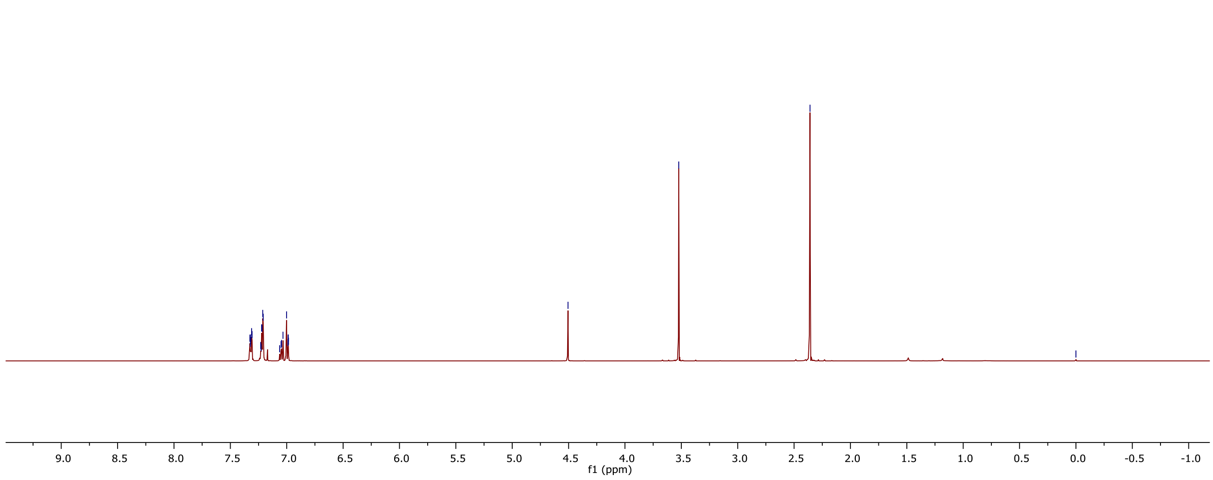


**$^{13}$C NMR (126 MHz, CDCl$_3$)** methyl 2-((2,6-dimethylphenyl)thio)-2-phenylacetate **3k**

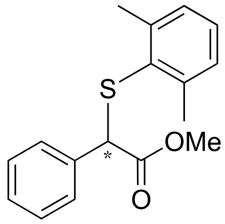


**3k**

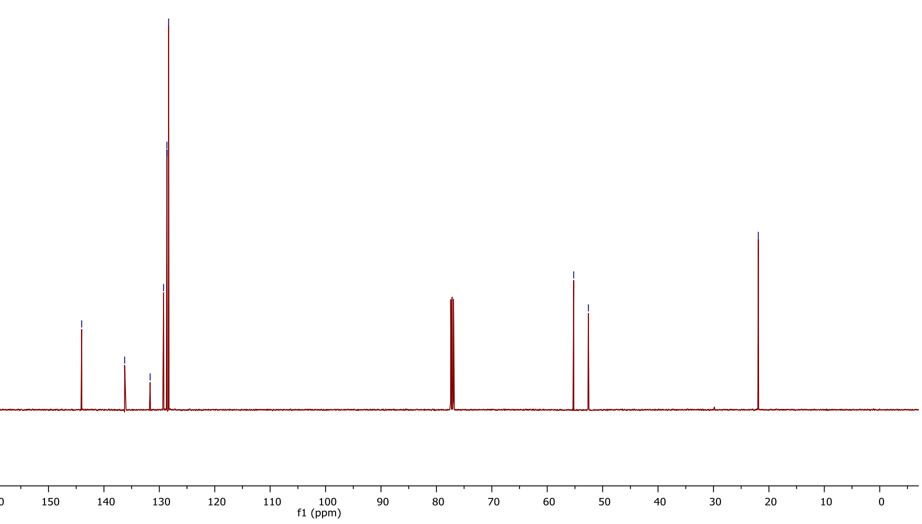

**$^1$H NMR (500 MHz, CDCl$_3$)** methyl 2-((4-hydroxyphenyl)thio)-2-phenylacetate **3l**

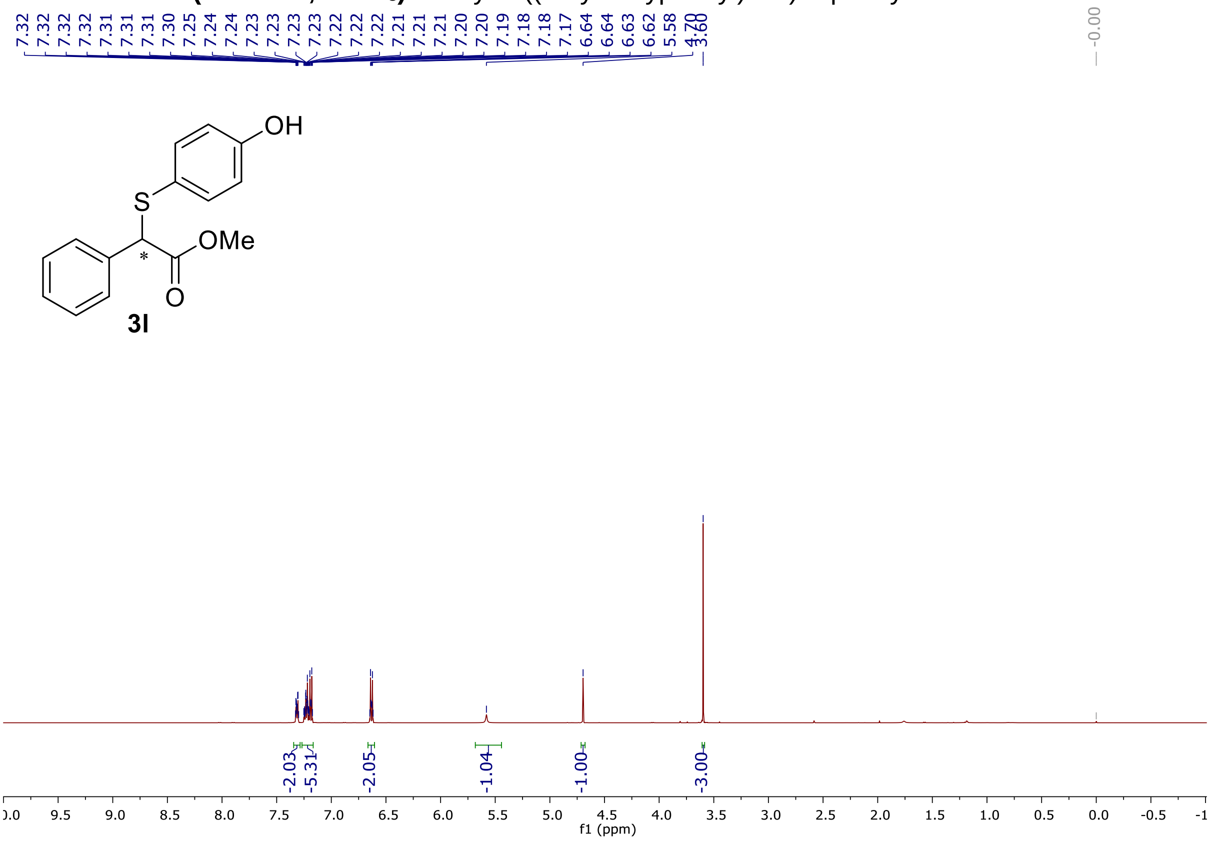


**$^{13}$C NMR (126 MHz, CDCl$_3$)** methyl 2-((4-hydroxyphenyl)thio)-2-phenylacetate **3l**

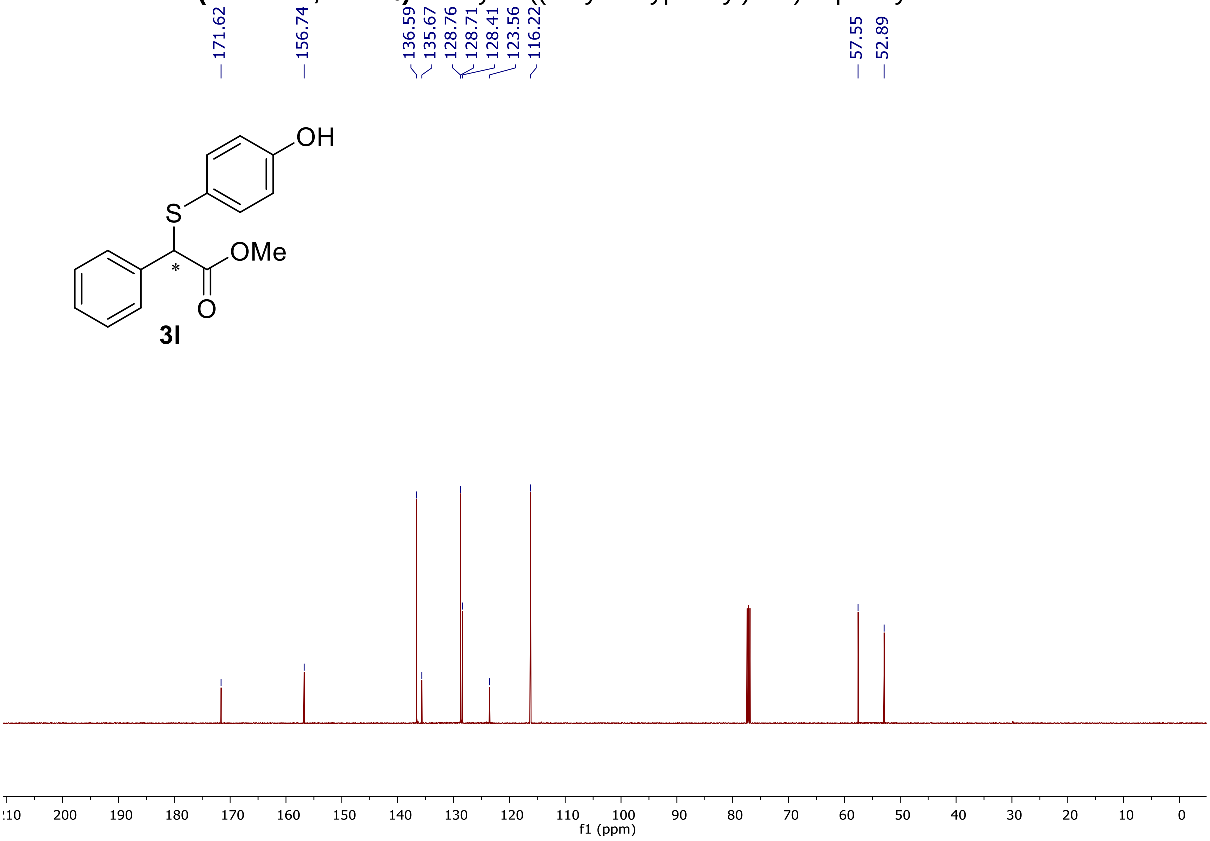

**$^1$H NMR (500 MHz, $CDCl_3$)** methyl 2-(naphthalen-2-ylthio)-2-phenylacetate **3m**

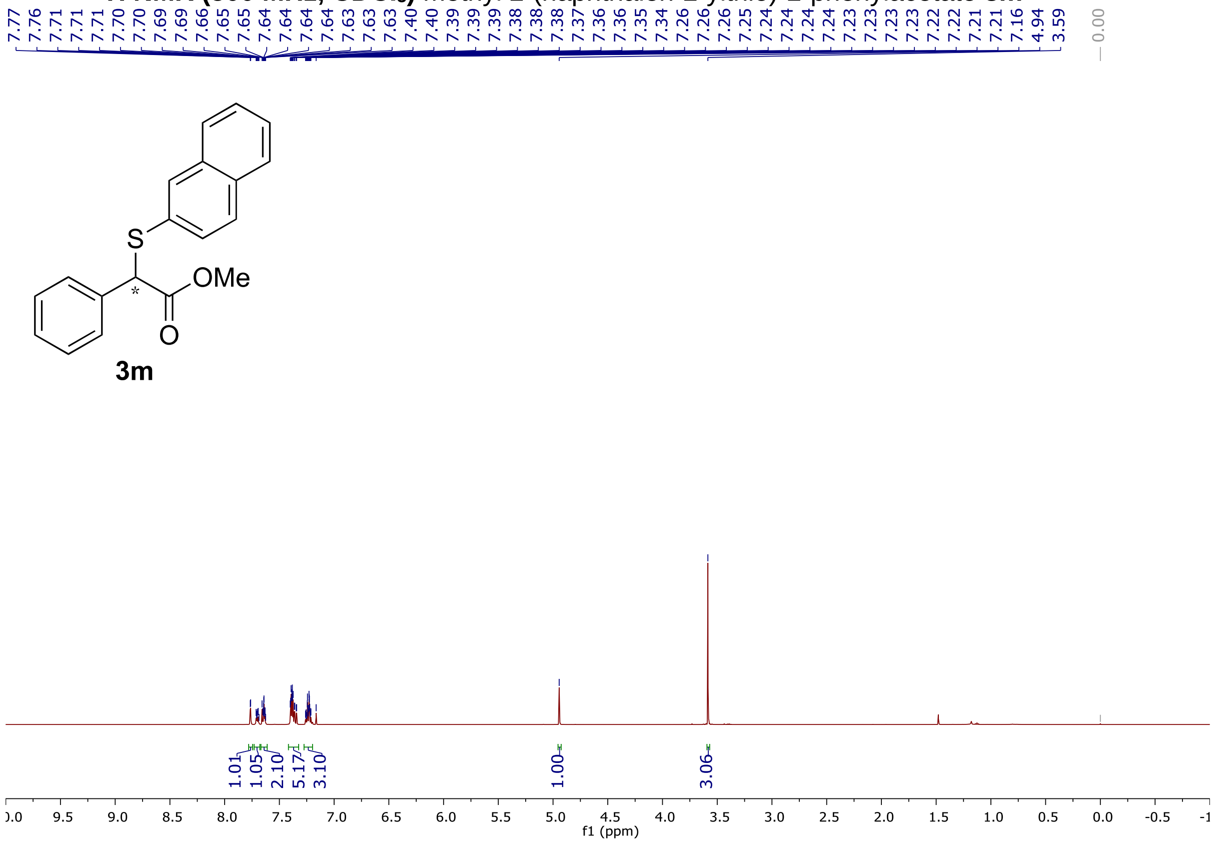


**$^{13}$C NMR (126 MHz, $CDCl_3$)** methyl 2-(naphthalen-2-ylthio)-2-phenylacetate **3m**

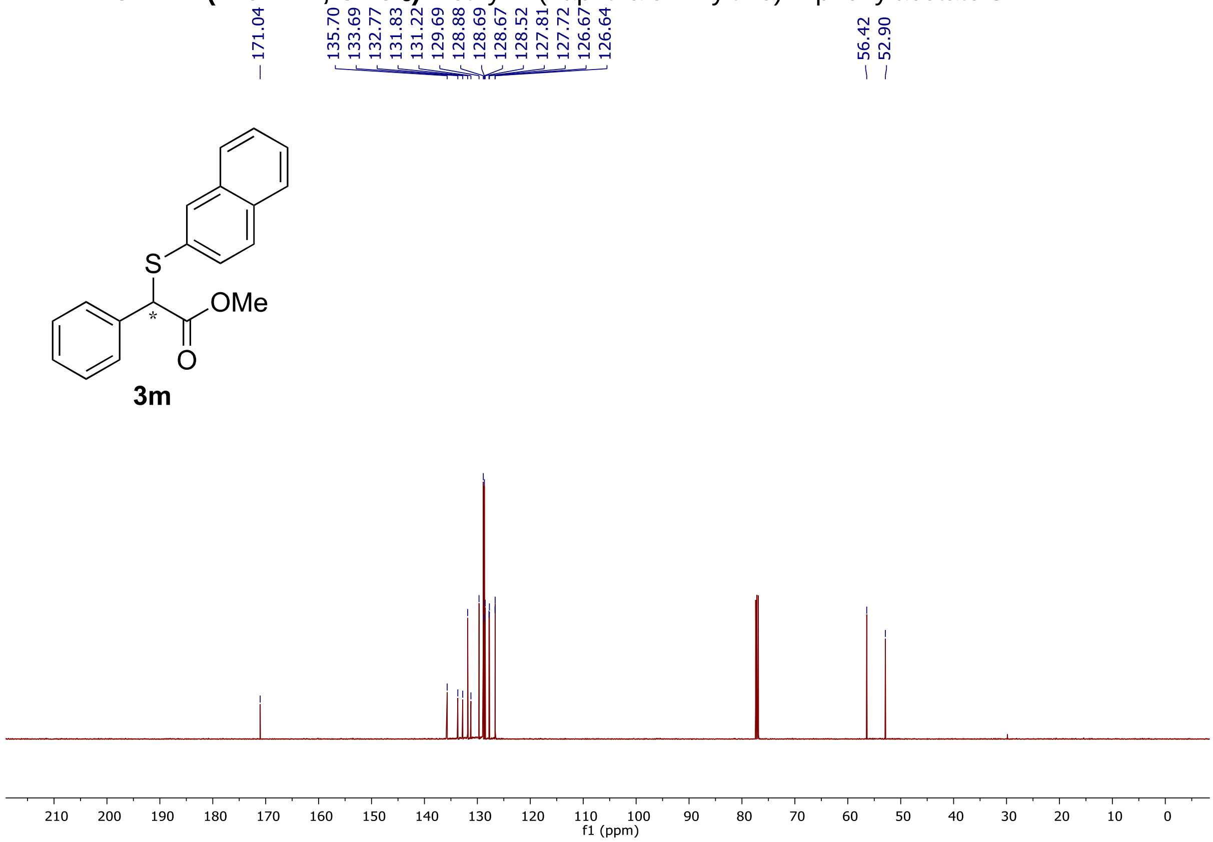

**$^1$H NMR (500 MHz, CDCl$_3$)** ethyl 2-(4-bromophenyl)-2-((4-methoxyphenyl)thio)- phenylacetate **3n**

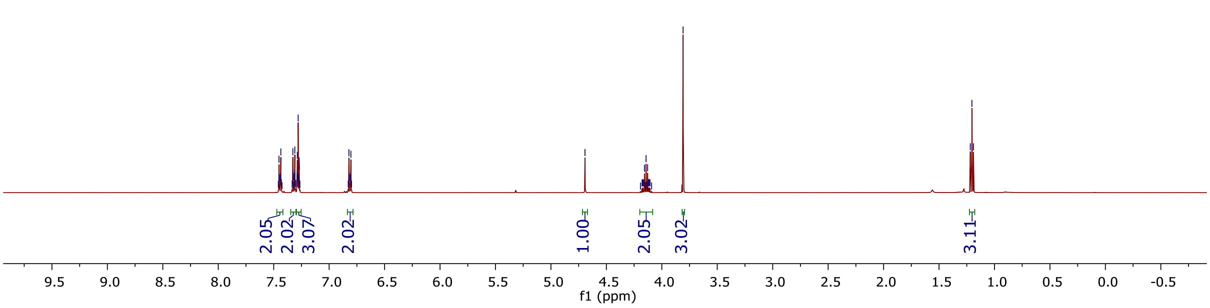


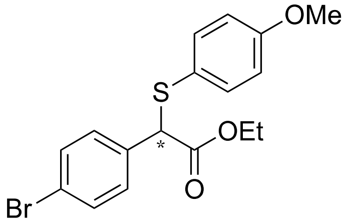


**3n**

**$^{13}$C NMR (126 MHz, CDCl$_3$)** ethyl 2-(4-bromophenyl)-2-((4-methoxyphenyl)thio)- phenylacetate **3n**

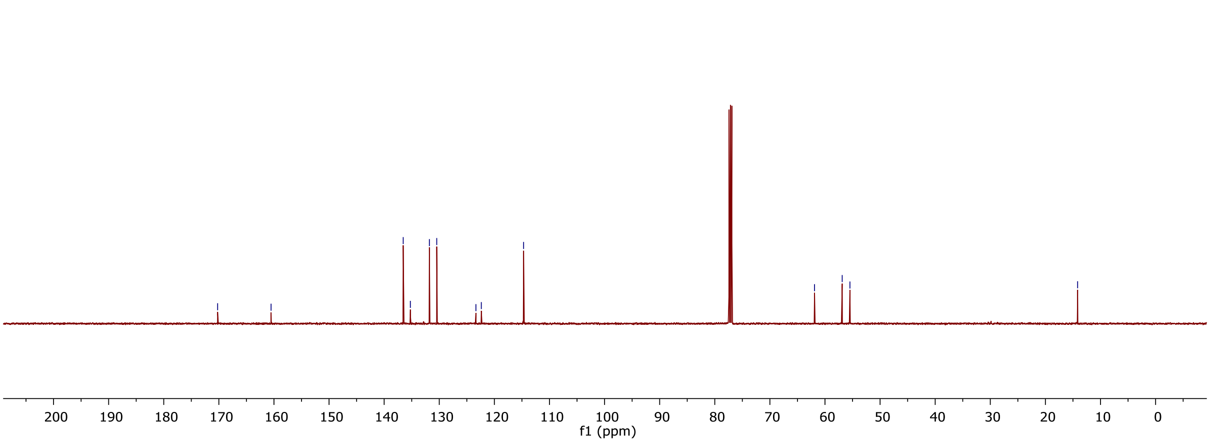


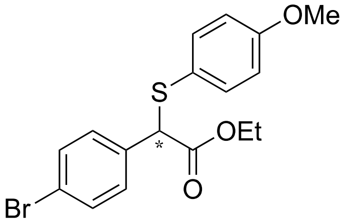


**3n**

**$^{1}$H NMR (500 MHz, CDCl3)** Ethyl 2-(4-bromophenyl)-2-((4-(trifluoromethyl)phenyl)thio)-phenylacetate **3o**

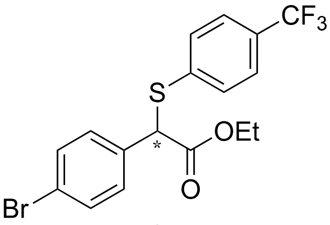


**3o**

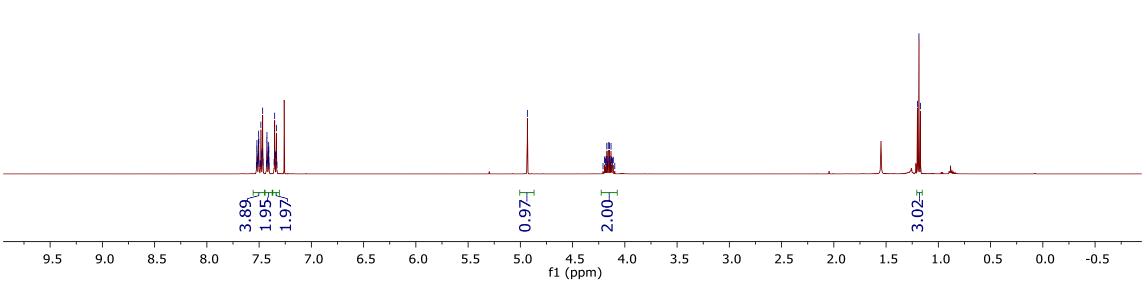


**$^{13}$C NMR (126 MHz, CDCl3)** Ethyl 2-(4-bromophenyl)-2-((4-(trifluoromethyl)phenyl)thio)-phenylacetate **3o**

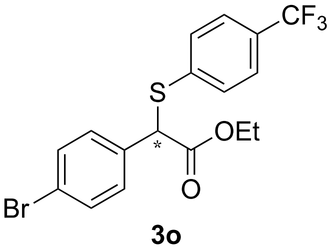


**3o**

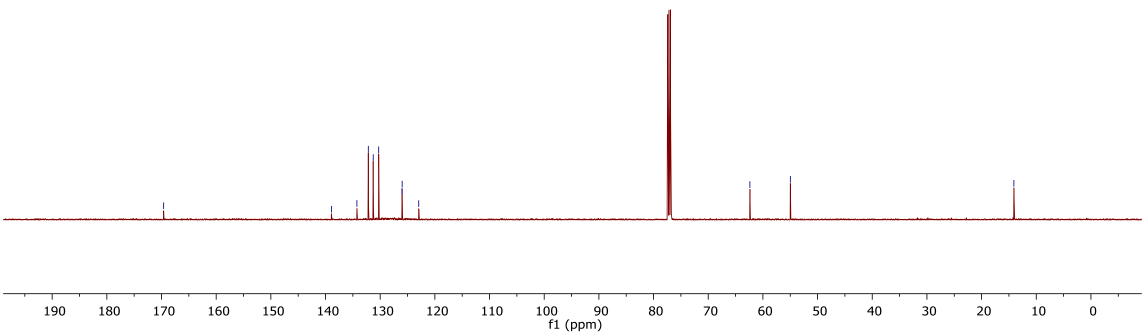

**$^{19}$F NMR (471 MHz, CDCl3)** Ethyl 2-(4-bromophenyl)-2-((4-(trifluoromethyl)phenyl)thio)-phenylacetate **3o**

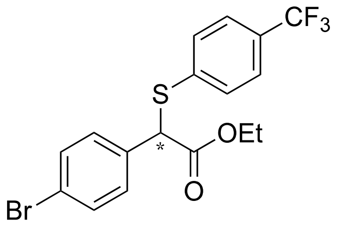


**3o**

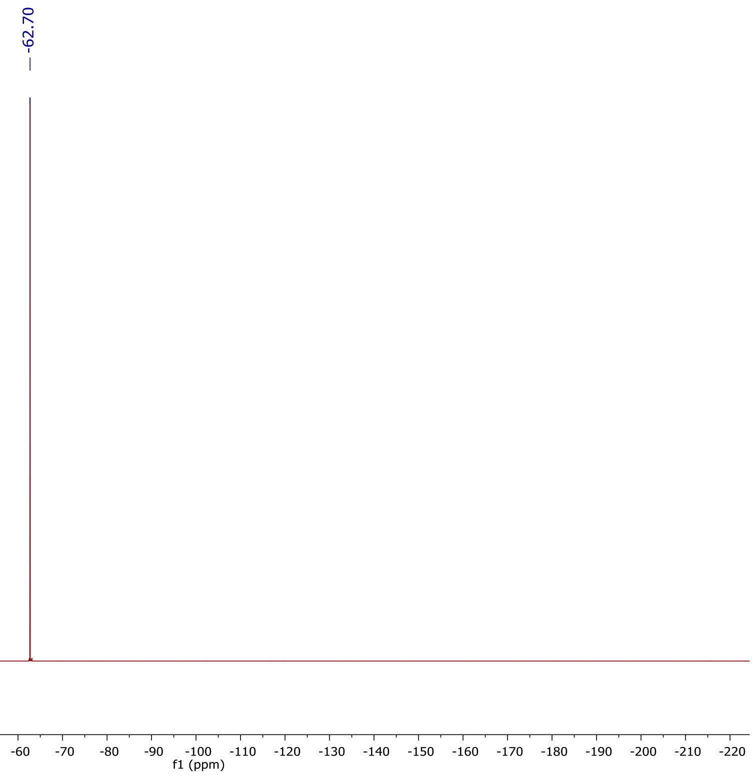

**$^{1}$H NMR (500 MHz, CDCl$_3$)** methyl 2-(4-chlorophenyl)-2-((4-methoxyphenyl)thio)acetate **3p**

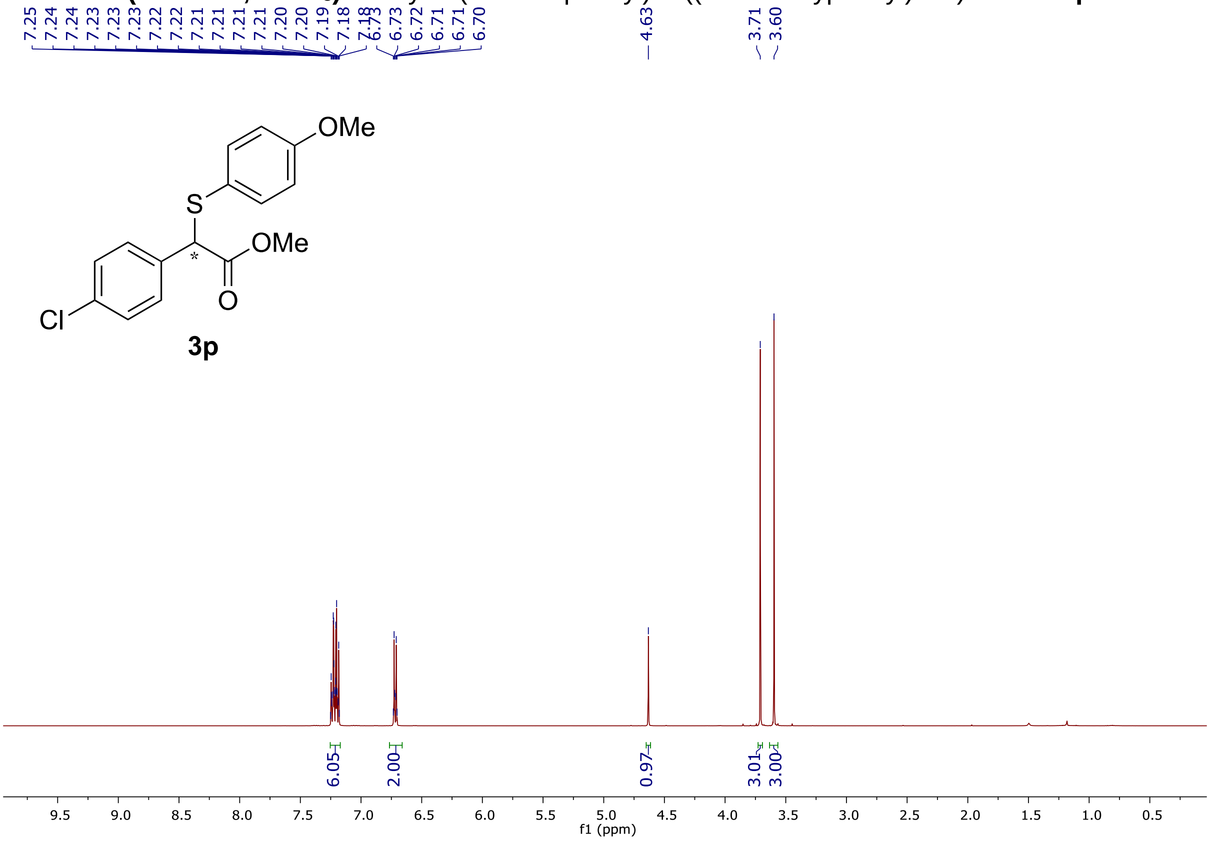


**$^{13}$C NMR (126 MHz, CDCl$_3$)** methyl 2-(4-chlorophenyl)-2-((4-methoxyphenyl)thio)acetate **3p**

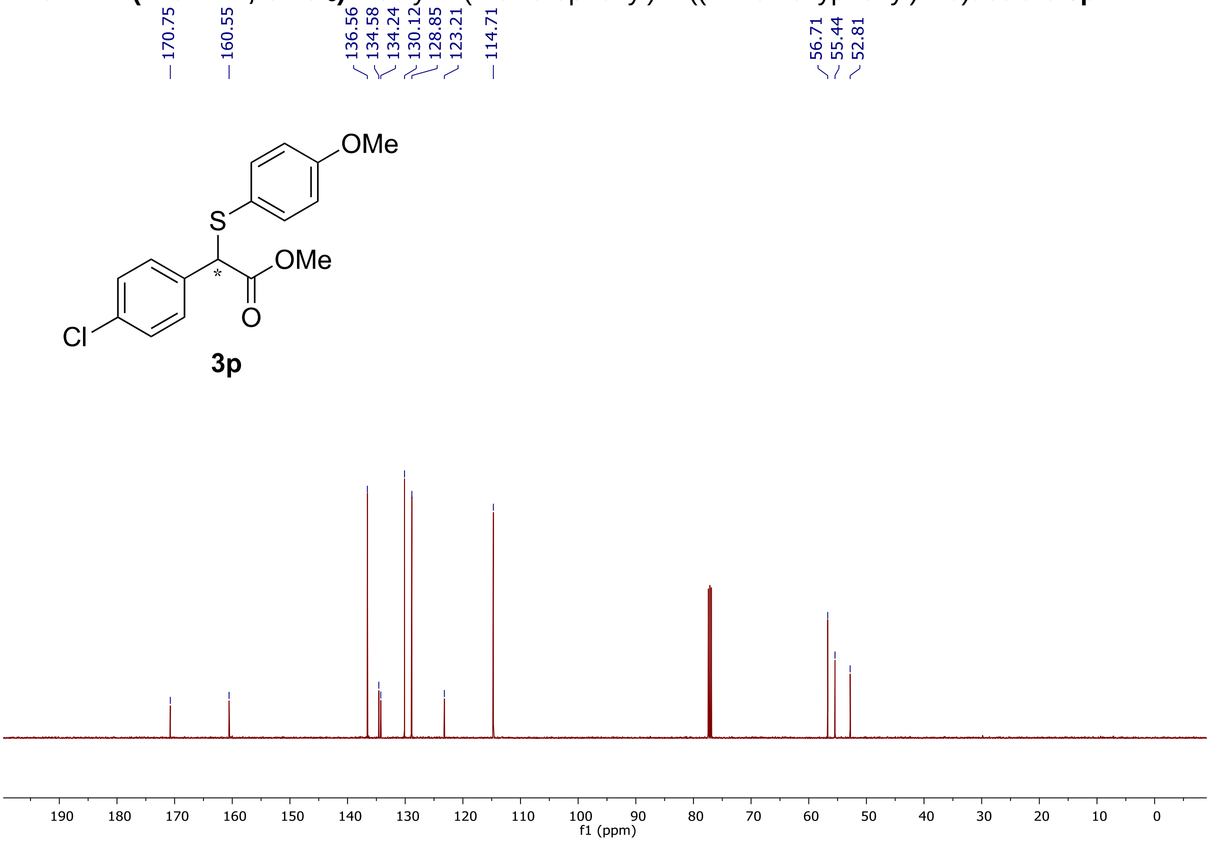


**$^{1}$H NMR (500 MHz, CDCl$_3$)** methyl 2-((4-nitrophenyl)thio)-2-(*p*-tolyl)-phenylacetate **3q**

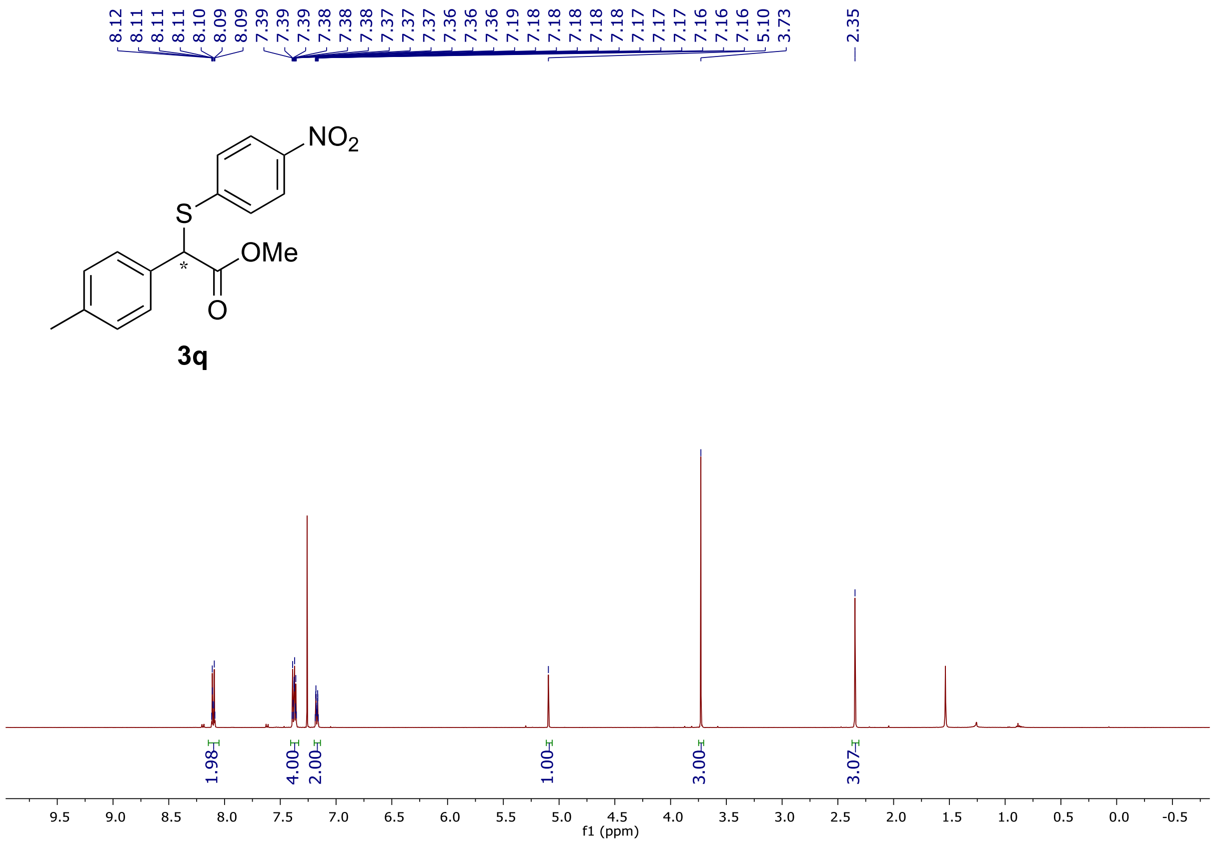


**$^{13}$C NMR (126 MHz, $CDCl_3$)** methyl 2-((4-nitrophenyl)thio)-2-(*p*-tolyl)-phenylacetate **3q**

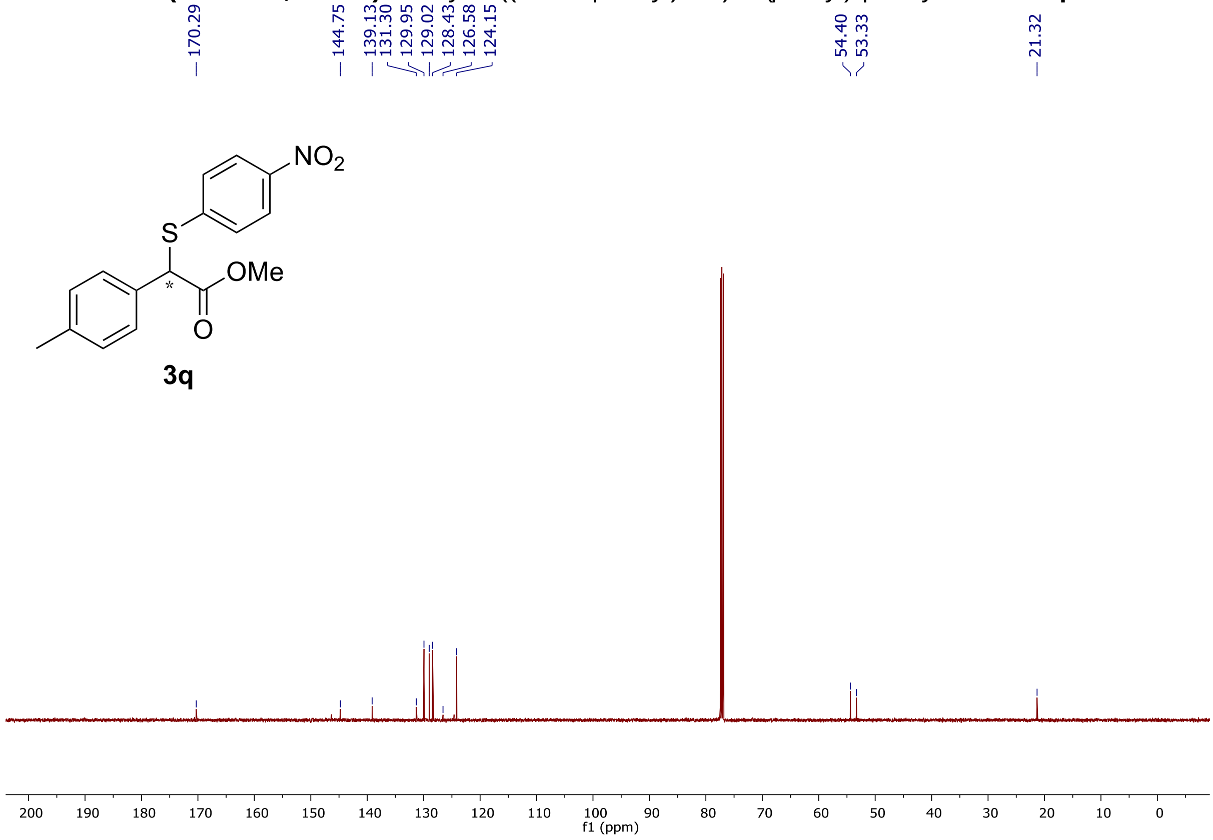

**$^1$H NMR (500 MHz, CDCl$_3$)** methyl 2-((4-bromophenyl)thio)-2-(*p*-tolyl)-phenylacetate **3r**

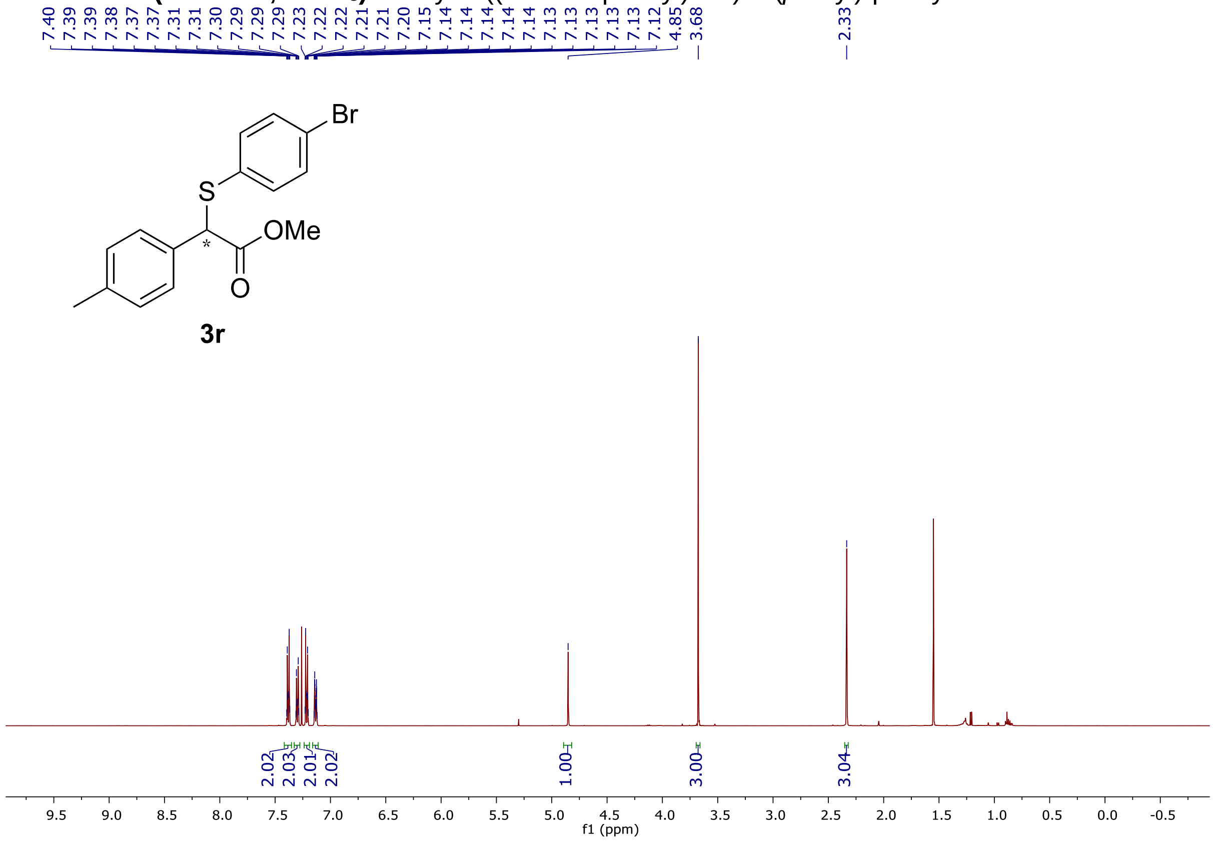


**$^{13}$C NMR (126 MHz, CDCl$_3$)** methyl 2-((4-bromophenyl)thio)-2-(*p*-tolyl)-phenylacetate **3r**

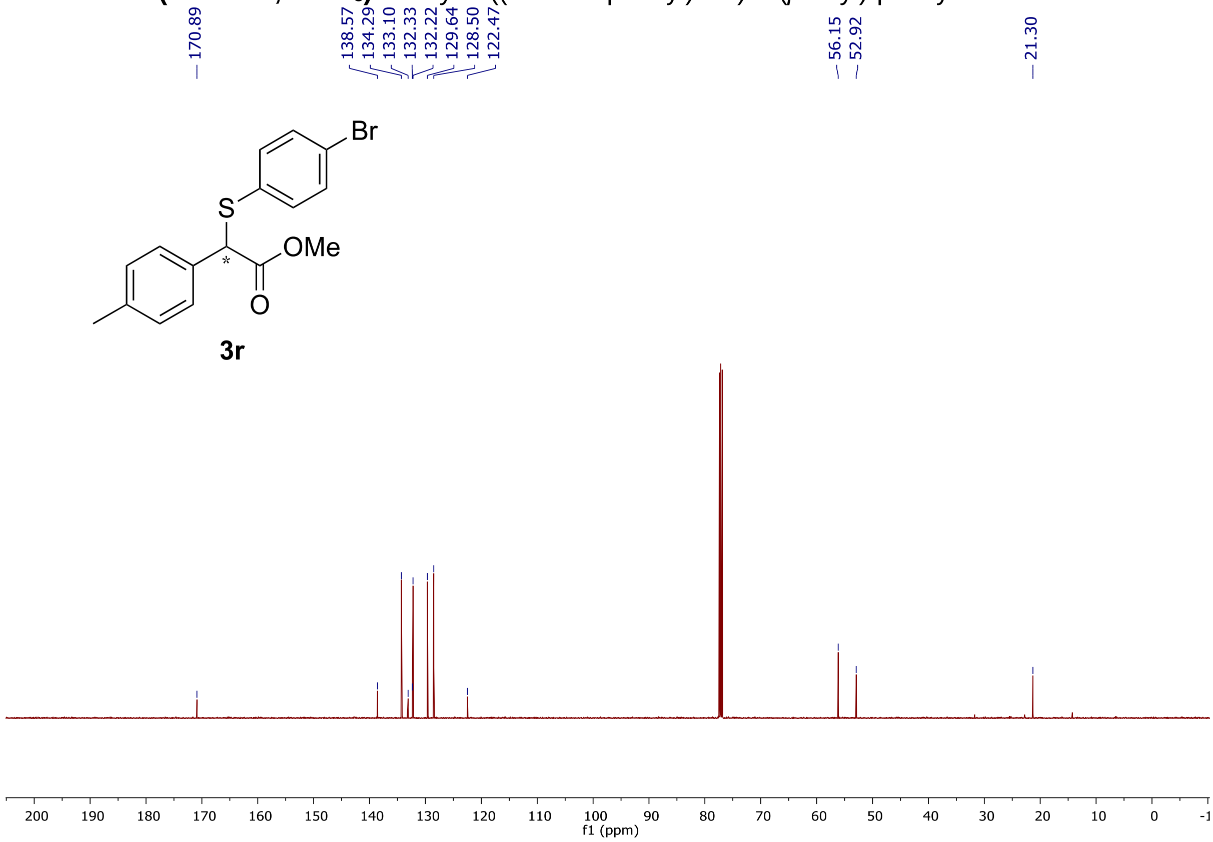

**$^{1}$H NMR (500 MHz, CDCl$_3$)** methyl 2-(*p*-tolyl)-2-(*o*-tolylthio)-phenylacetate **3s**

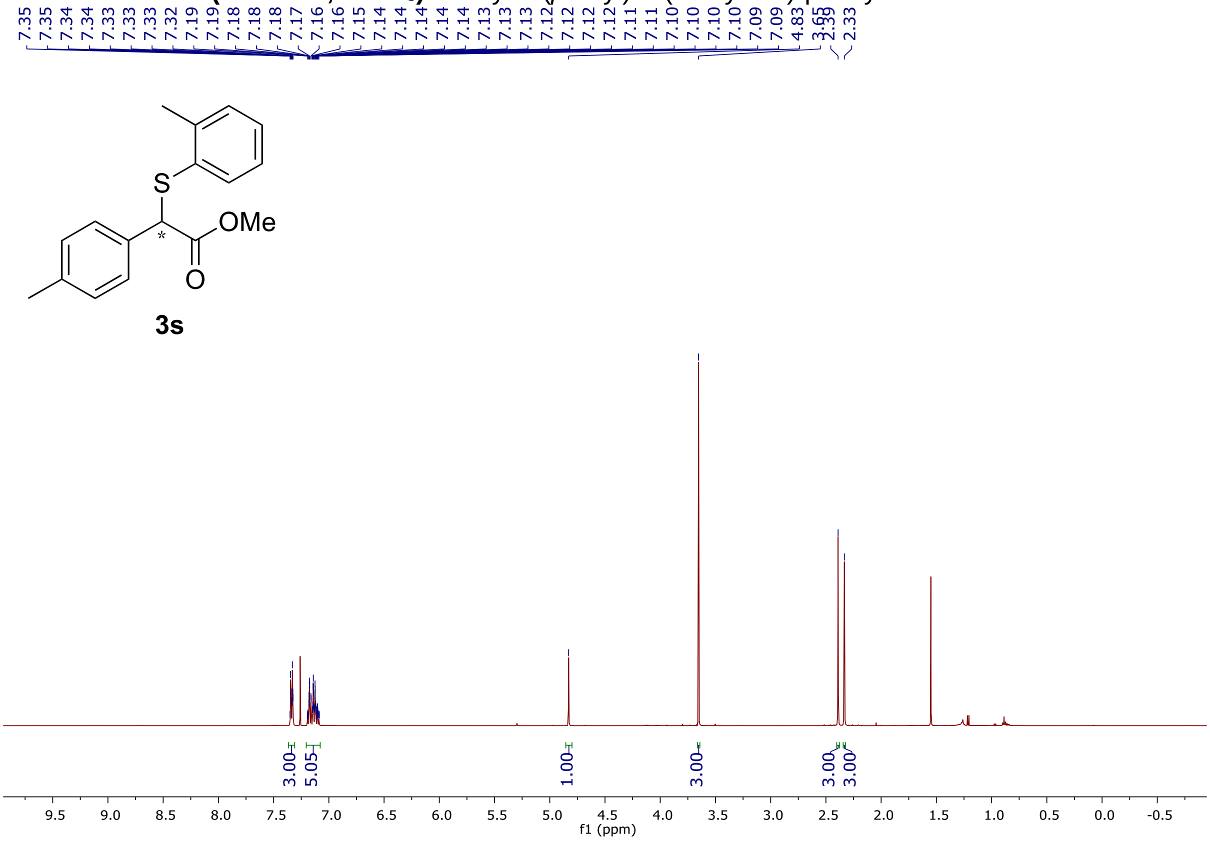


**$^{13}$C NMR (126 MHz, CDCl$_3$)** methyl 2-(*p*-tolyl)-2-(*o*-tolylthio)-phenylacetate **3s**

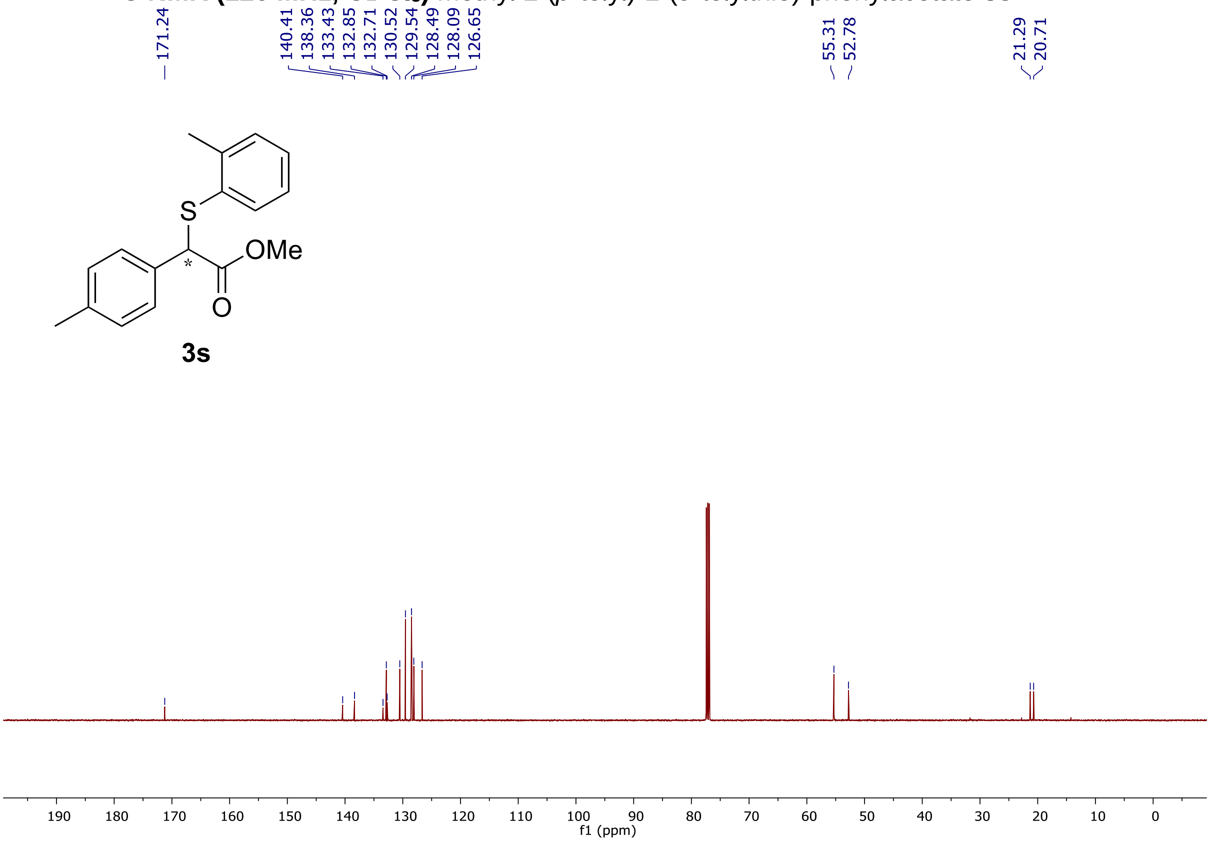

**$^{1}$H NMR (500 MHz, CDCl$_3$)** methyl 2-((4-methoxyphenyl)thio)-2-(naphthalen-2-yl)acetate **3t**

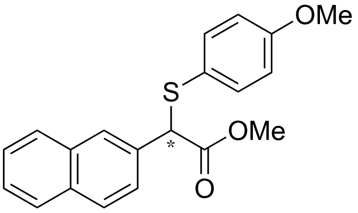

**3t**

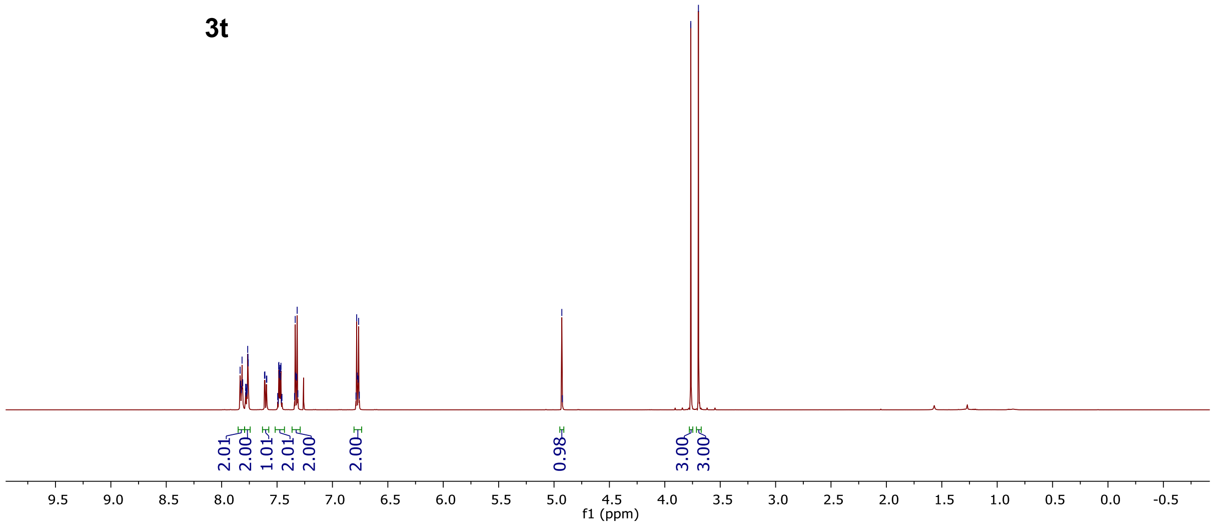


**$^{13}$C NMR (126 MHz, CDCl$_3$)** methyl 2-((4-methoxyphenyl)thio)-2-(naphthalen-2-yl)acetate **3t**

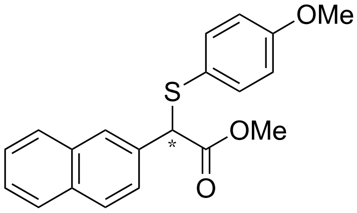

**3t**

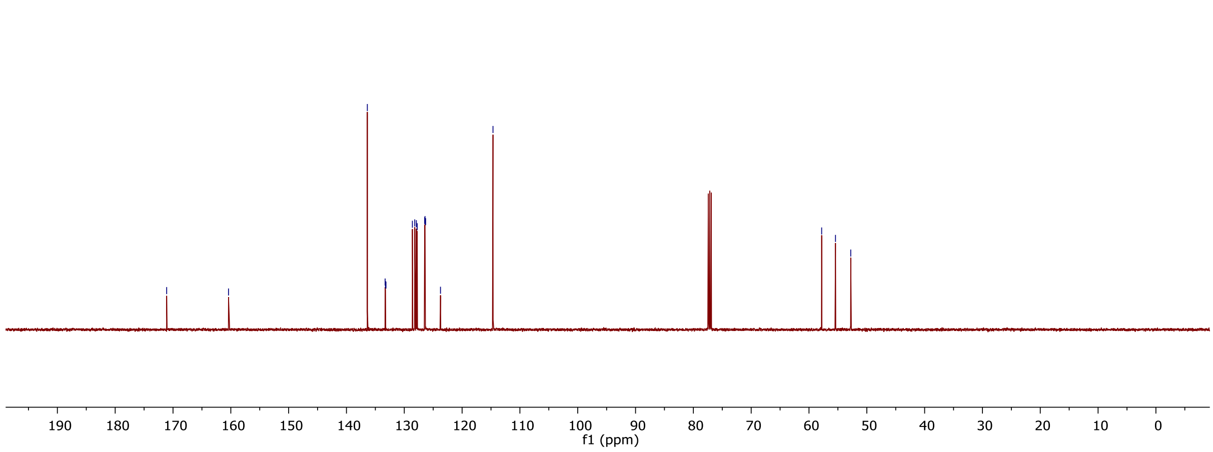

**$^{1}$H NMR (500 MHz, $CDCl_3$)** methyl 2-((4-methoxyphenyl)thio)-2-(pyridin-3-yl)acetate **3u**

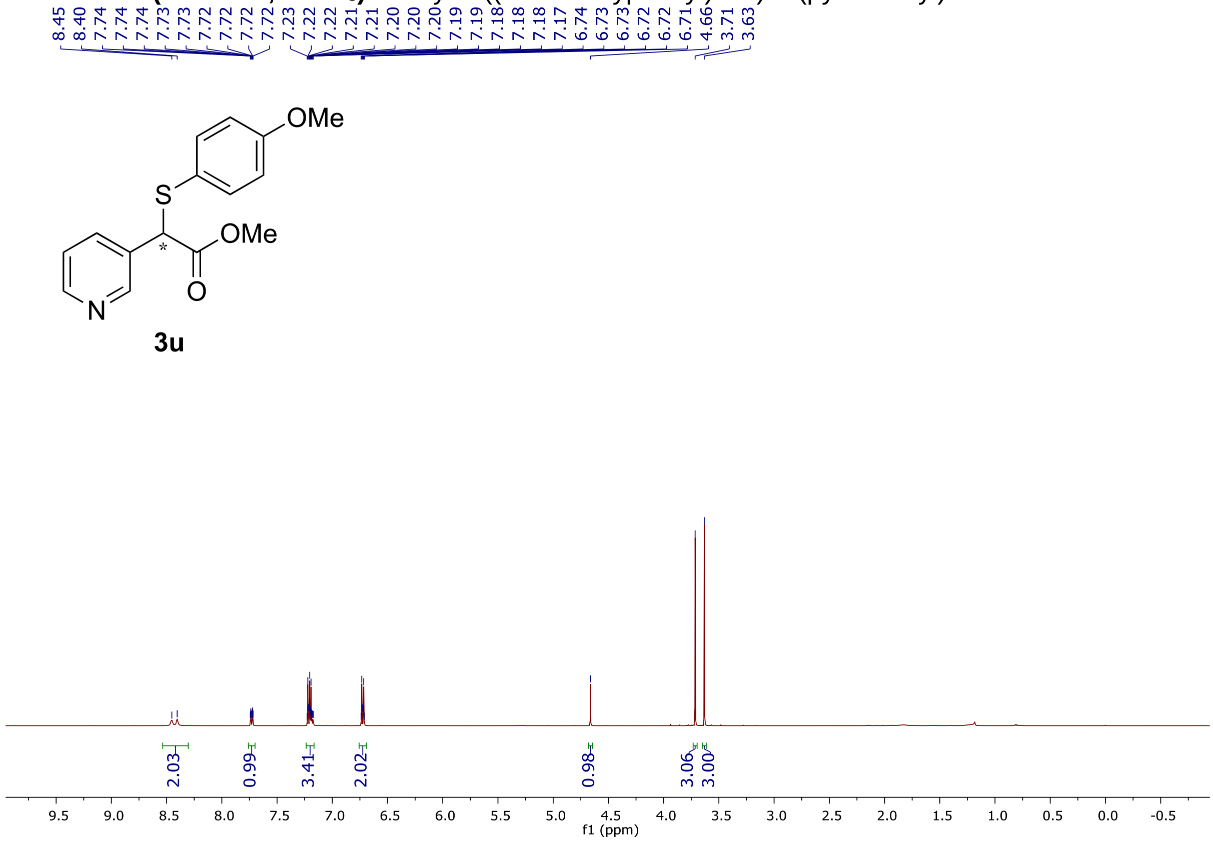


**$^{13}$C NMR (126 MHz, $CDCl_3$)** methyl 2-((4-methoxyphenyl)thio)-2-(pyridin-3-yl)acetate **3u**

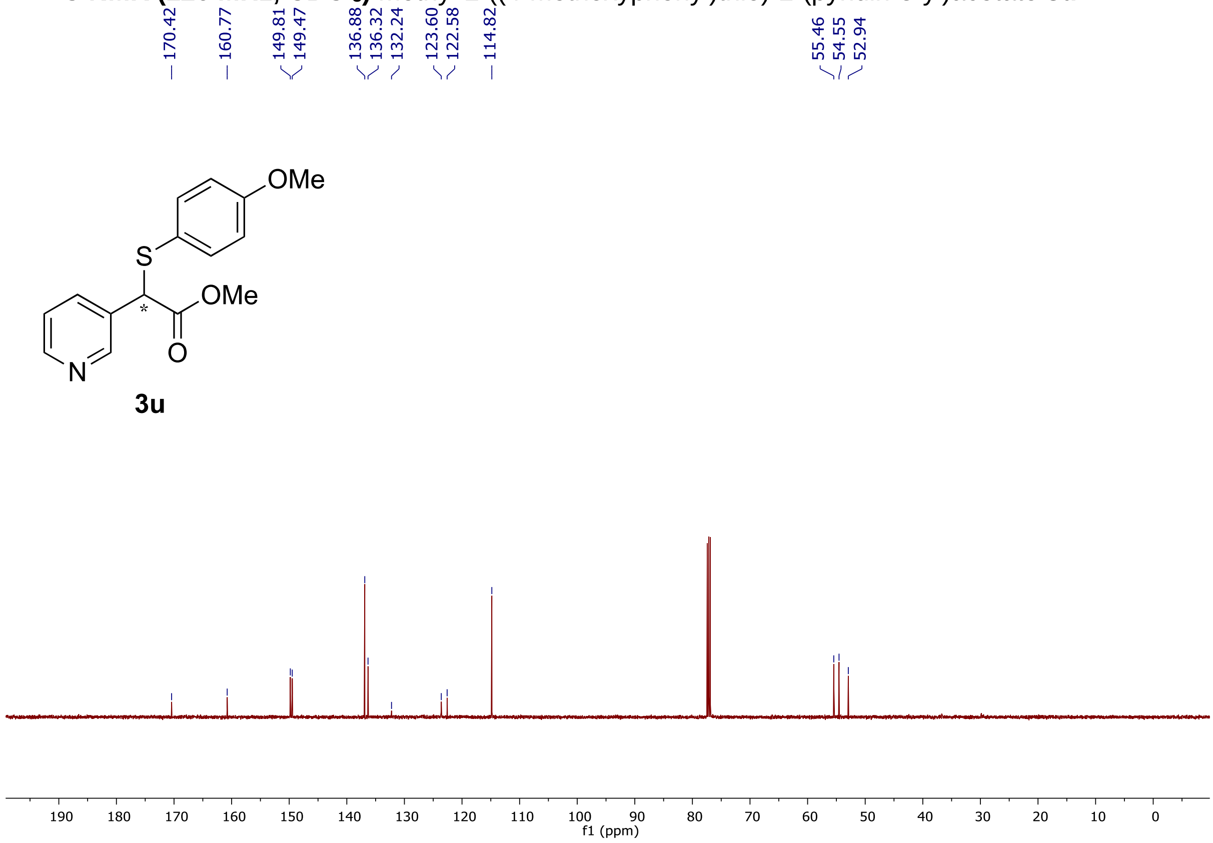

**$^1$H NMR (500 MHz, CDCl$_3$)** methyl 2-((4-methoxyphenyl)thio)-2-(4-(trifluoromethyl)phenyl)acetate **3v**

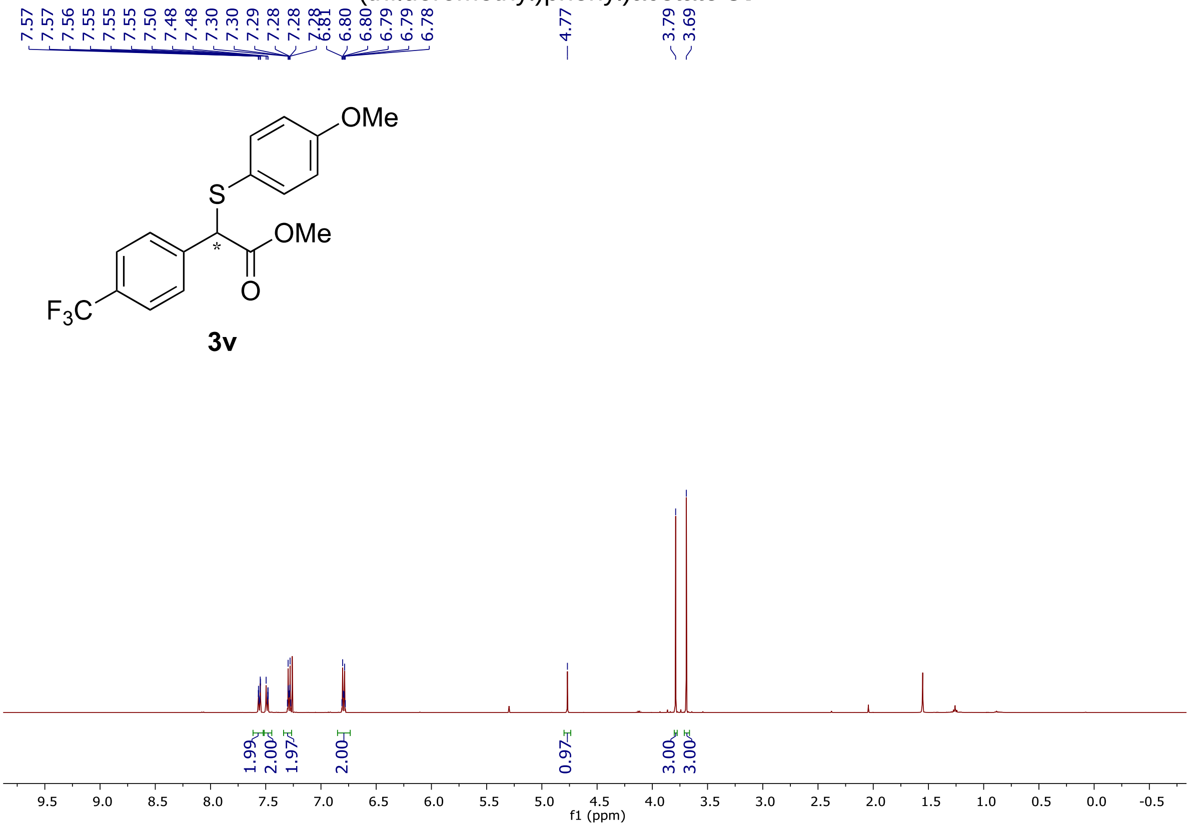


**3v**

**$^{13}$C NMR (126 MHz, CDCl$_3$)** methyl 2-((4-methoxyphenyl)thio)-2-(4-(trifluoromethyl)phenyl)acetate **3v**

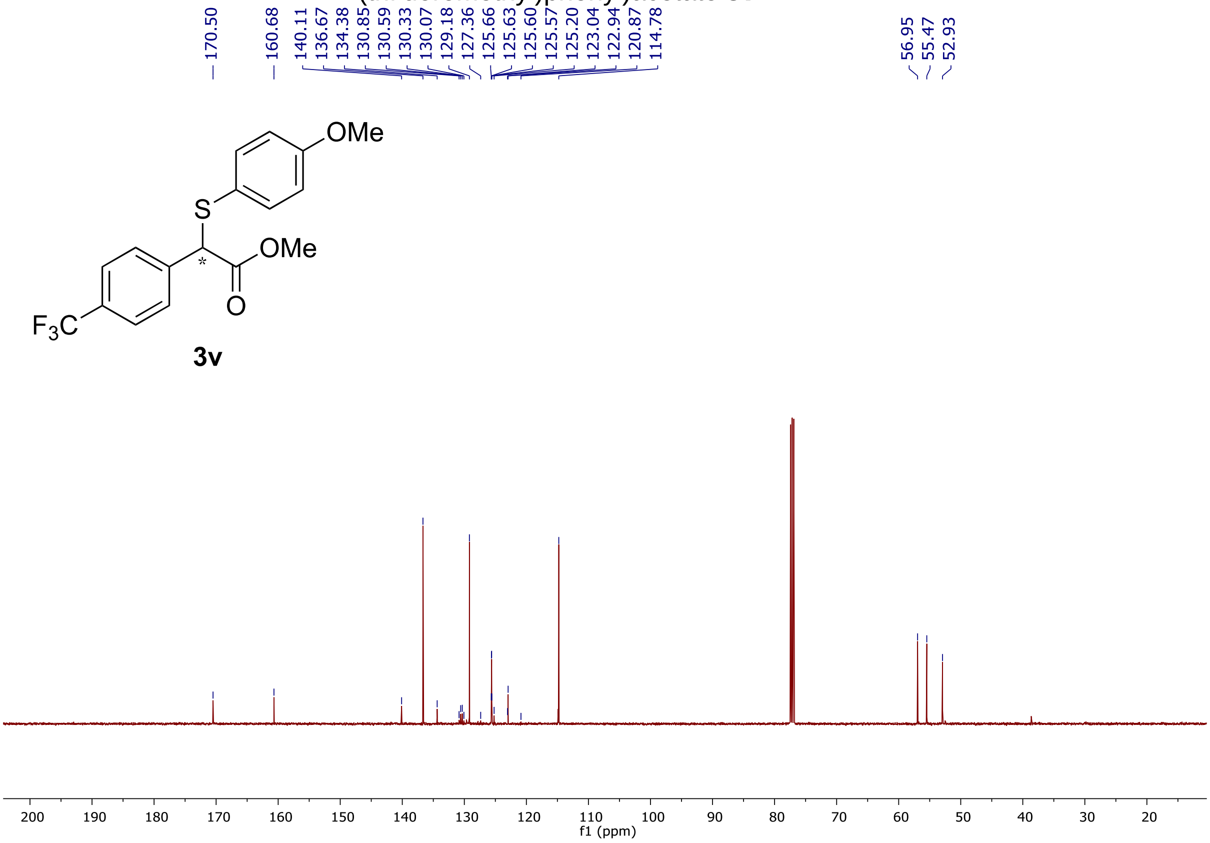


**3v**

**$^1$H NMR (500 MHz, CDCl$_3$)** ethyl 2-((4-methoxyphenyl)thio)-2-phenylacetate **3w**

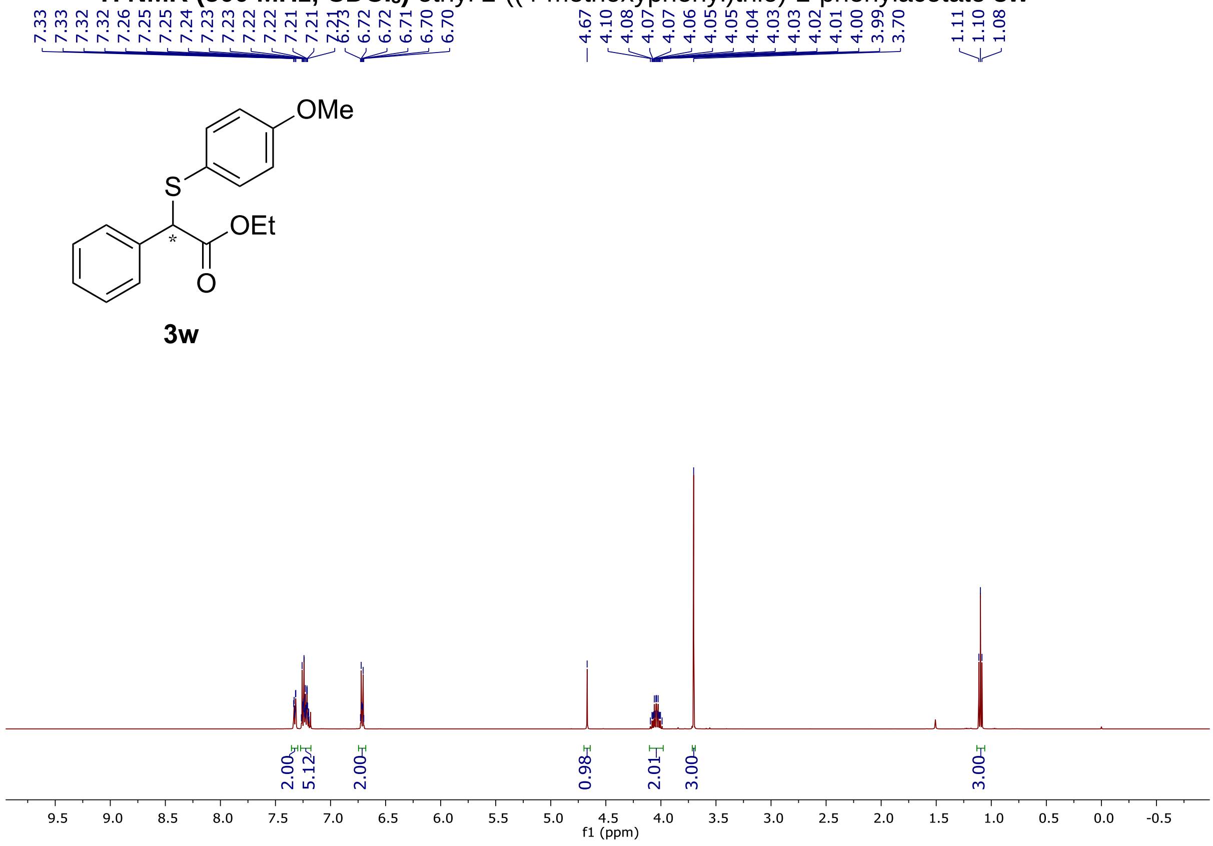


**$^{13}$C NMR (126 MHz, CDCl$_3$)** ethyl 2-((4-methoxyphenyl)thio)-2-phenylacetate **3w**

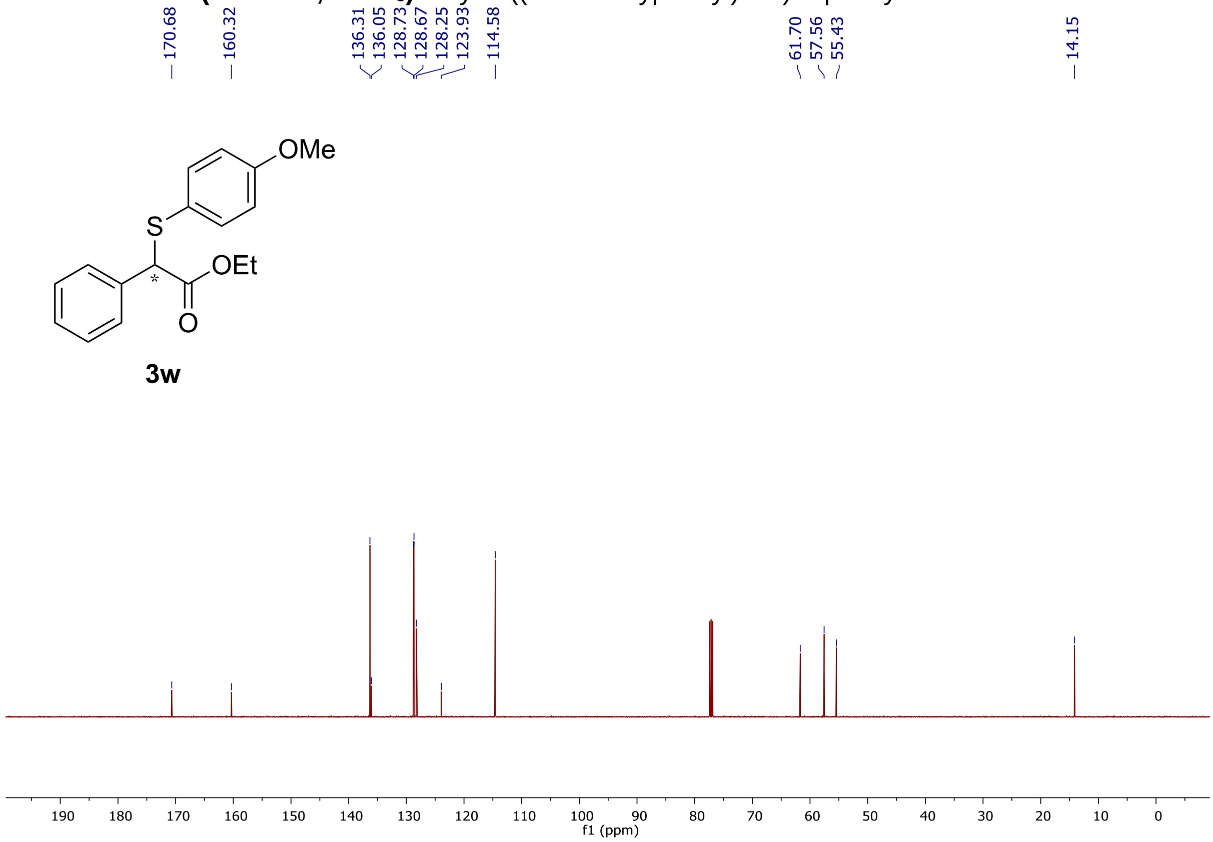

**$^{1}$H NMR (500 MHz, CDCl$_3$)** benzyl 2-((4-methoxyphenyl)thio)-2-phenylacetate **3x**

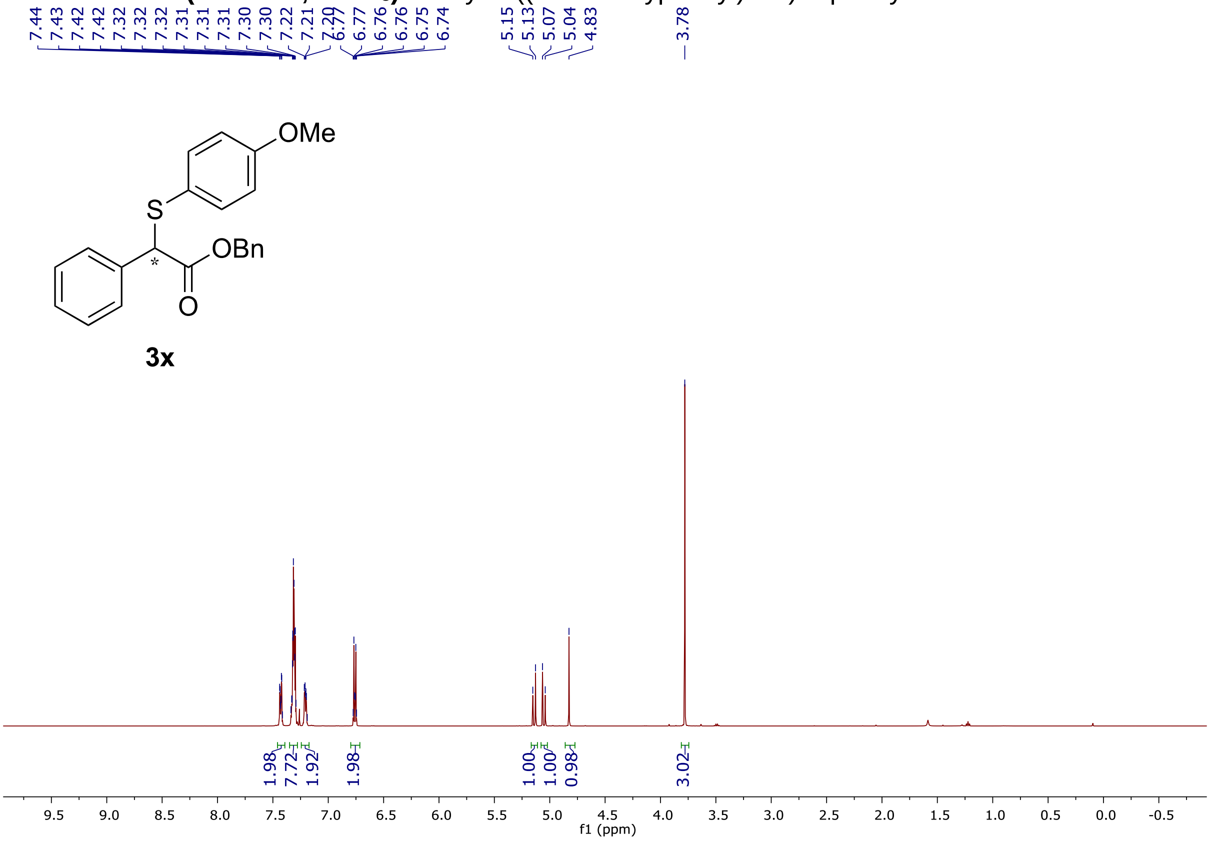


**$^{13}$C NMR (126 MHz, CDCl$_3$)** benzyl 2-((4-methoxyphenyl)thio)-2-phenylacetate **3x**

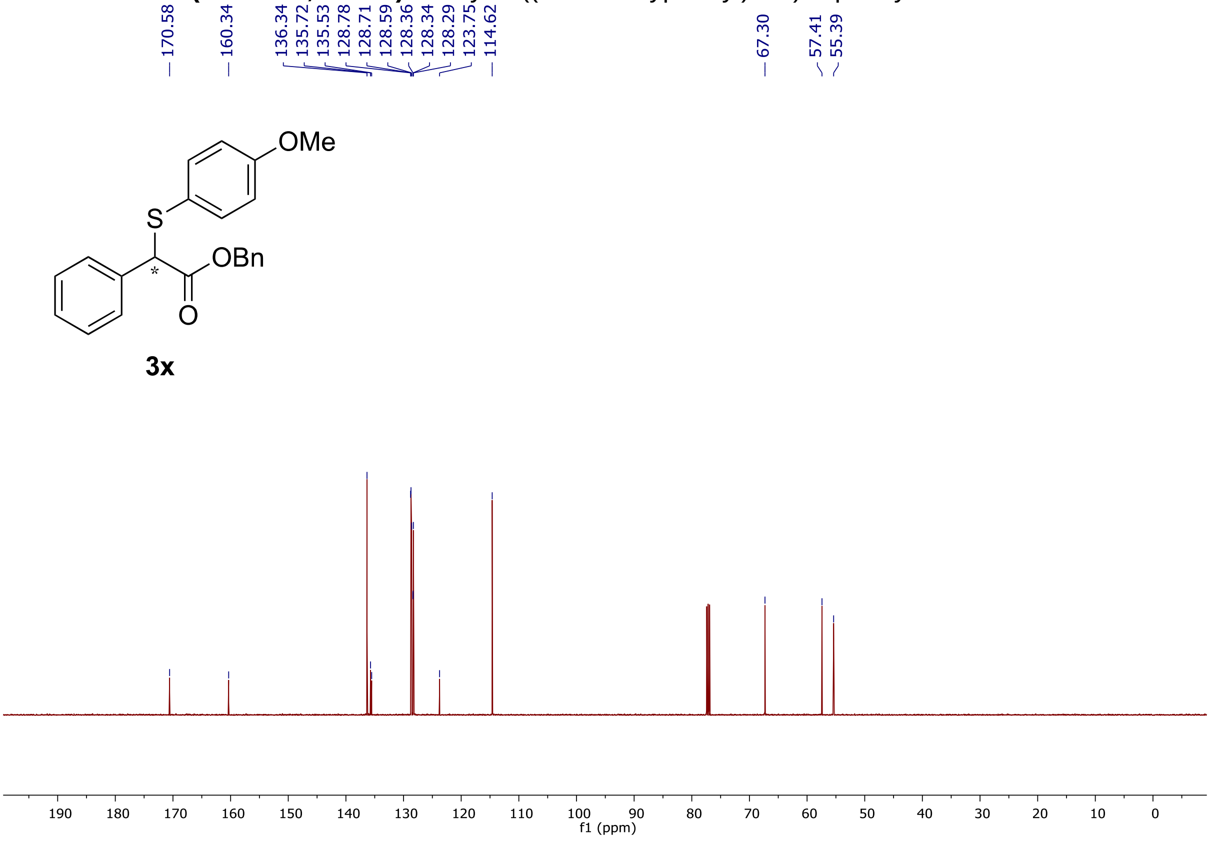

**$^{1}$H NMR (500 MHz, CDCl3)** *tert*-butyl 2-((4-methoxyphenyl)thio)-2-phenylacetate **3y**

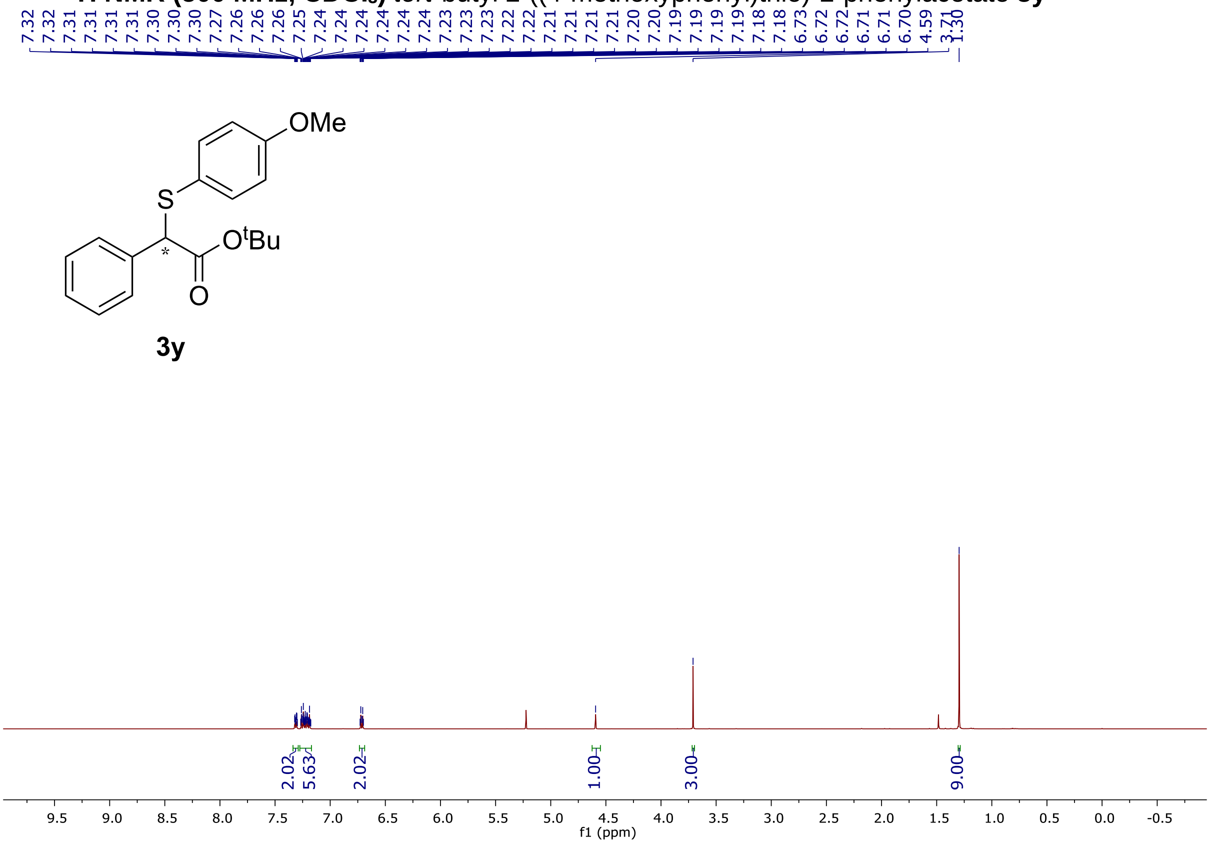


**$^{13}$C NMR (126 MHz, CDCl3)** *tert*-butyl 2-((4-methoxyphenyl)thio)-2-phenylacetate **3y**

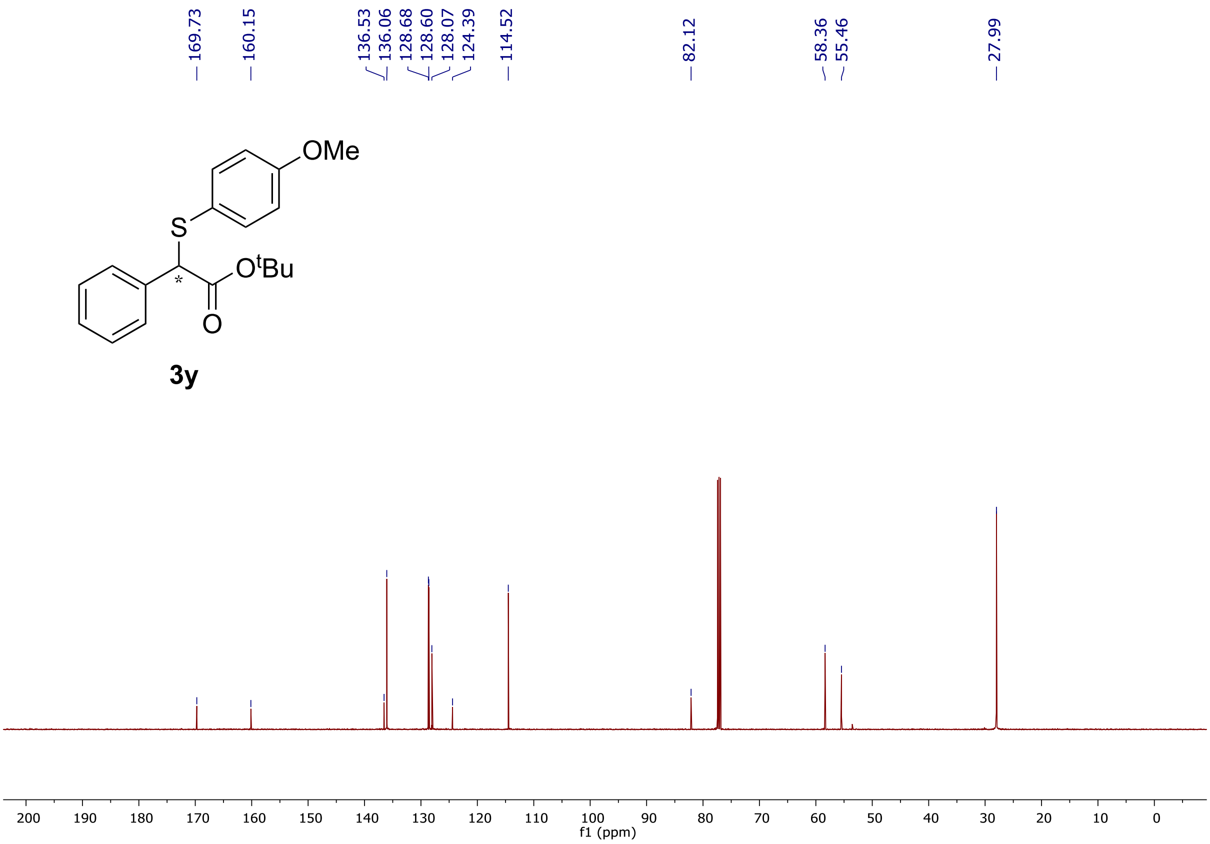

**$^1$H NMR (500 MHz, CDCl$_3$)** *tert*-butyl 2-(naphthalen-2-ylthio)-2-phenylacetate **3z**

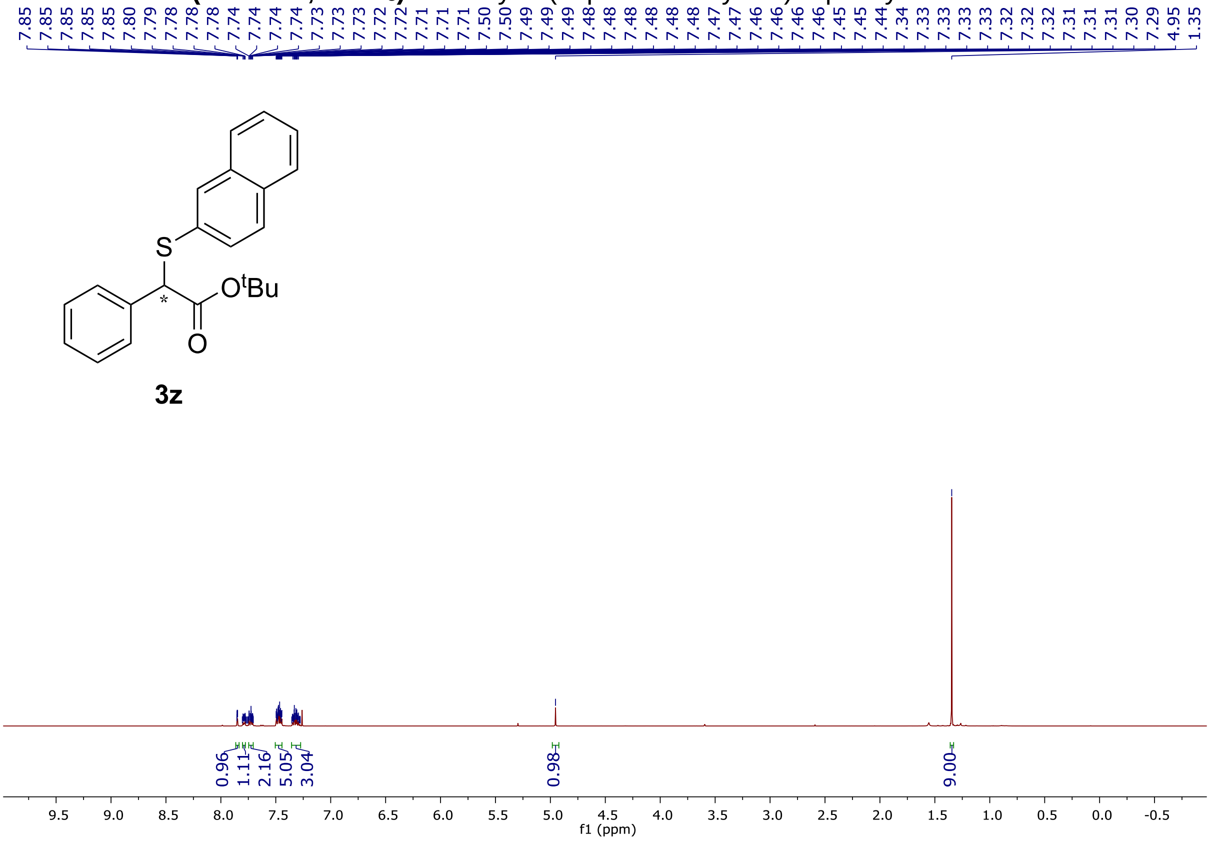


**$^{13}$C NMR (126 MHz, CDCl$_3$)** *tert*-butyl 2-(naphthalen-2-ylthio)-2-phenylacetate **3z**

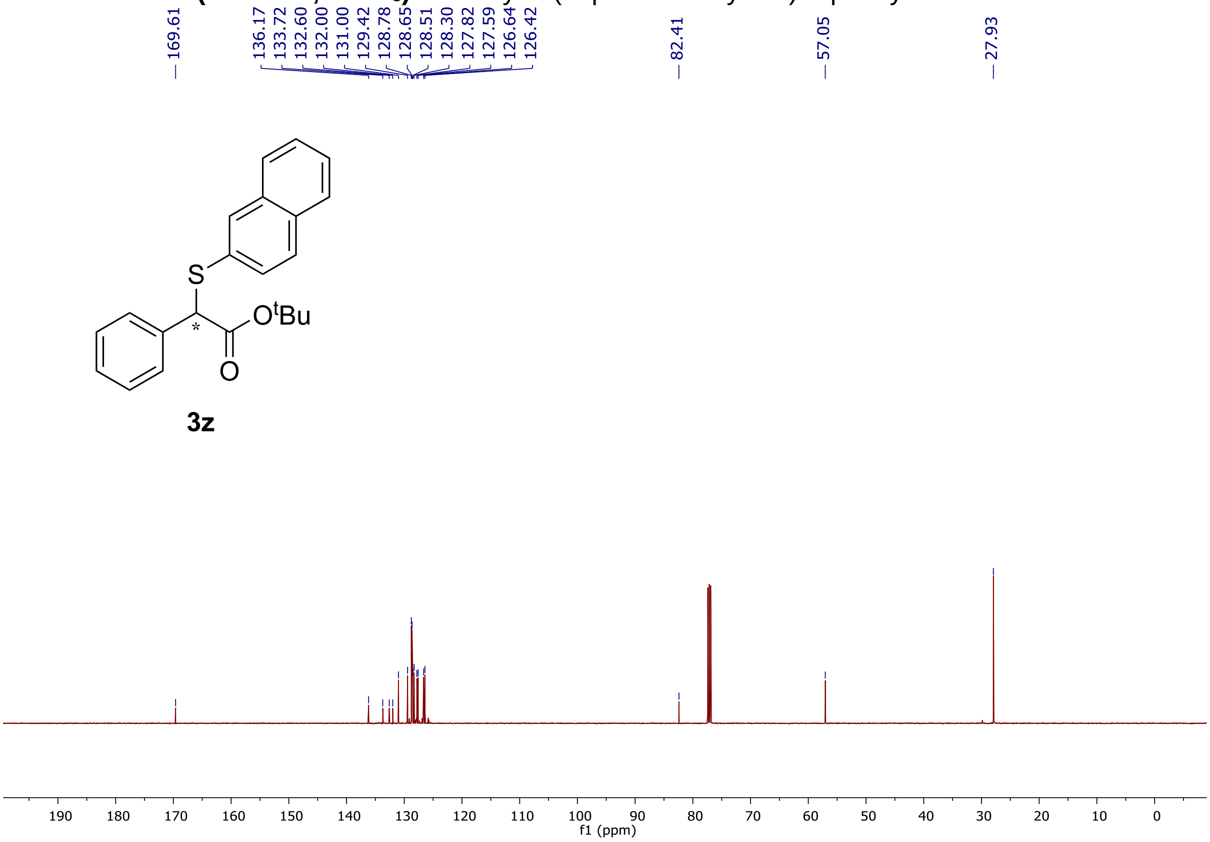

**¹H NMR (500 MHz, CDCl₃)** *tert*-butyl 2-((4-chlorophenyl)thio)-2-phenylacetate **3aa**

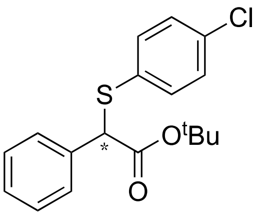


**3aa**

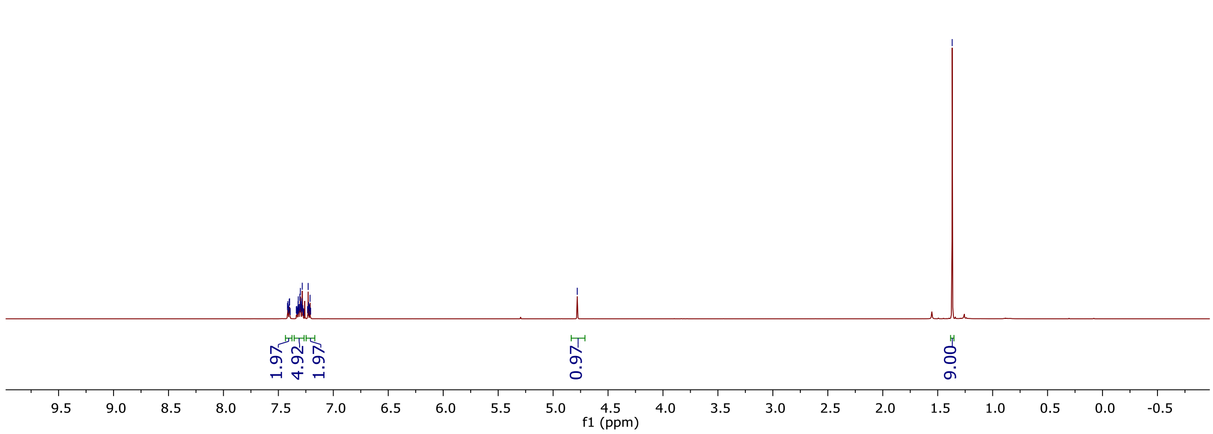


**¹³C NMR (126 MHz, CDCl₃)** *tert*-butyl 2-((4-chlorophenyl)thio)-2-phenylacetate **3aa**

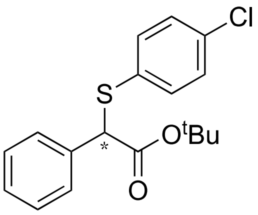


**3aa**

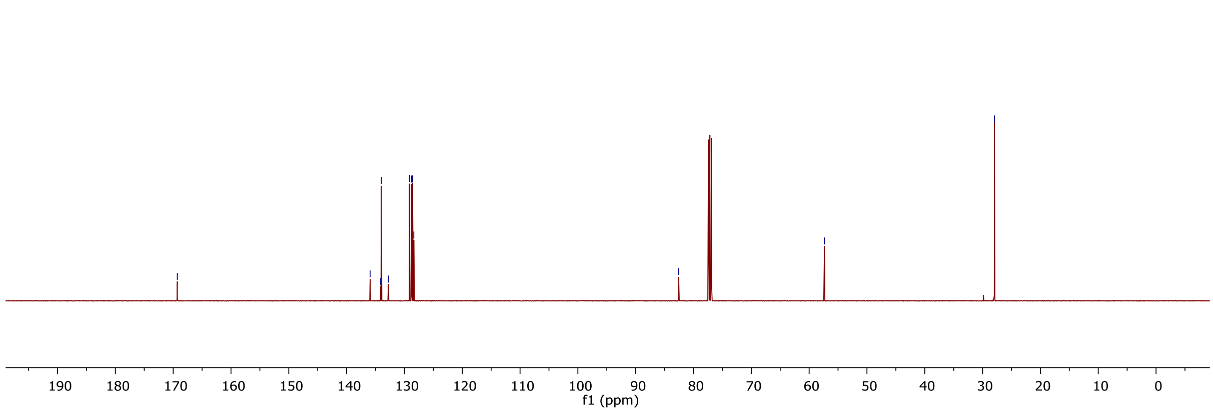

**$^{1}$H NMR (400 MHz, CDCl₃)** 2,2,2-trichloroethyl 2-((4-methoxyphenyl)thio)-2-phenylacetate **3ab**

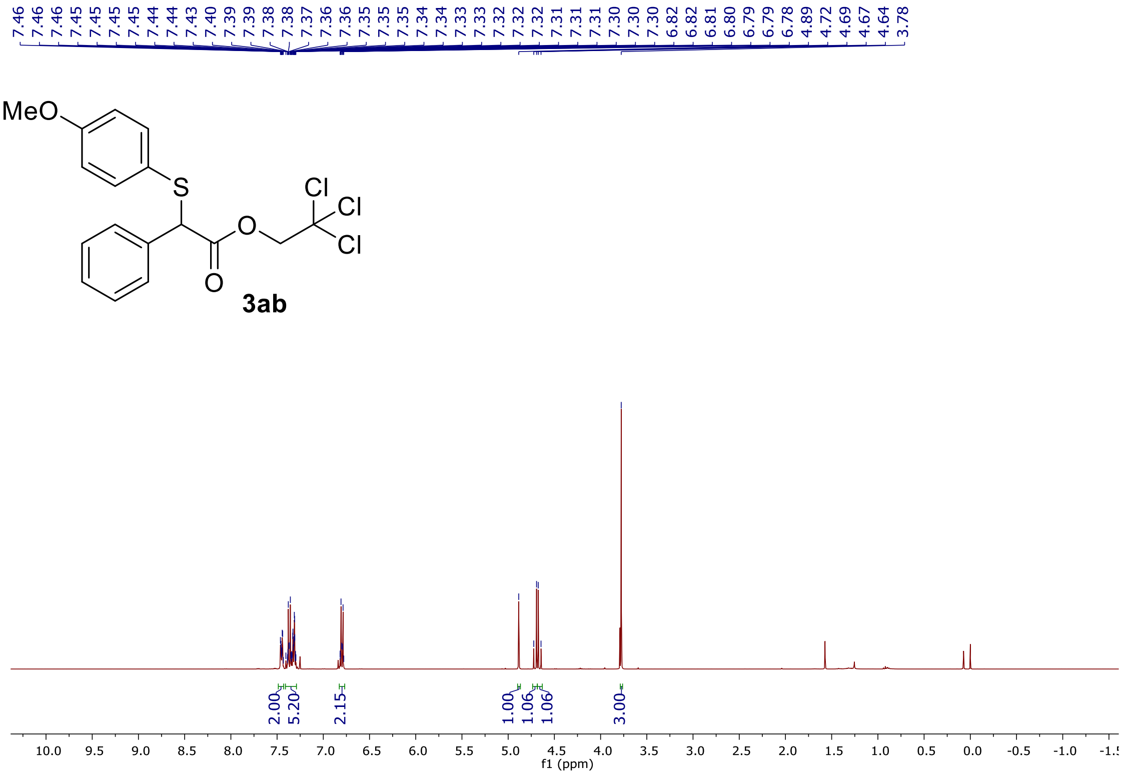


**$^{13}$C NMR (101 MHz, CDCl₃)** 2,2,2-trichloroethyl 2-((4-methoxyphenyl)thio)-2-phenylacetate **3ab**

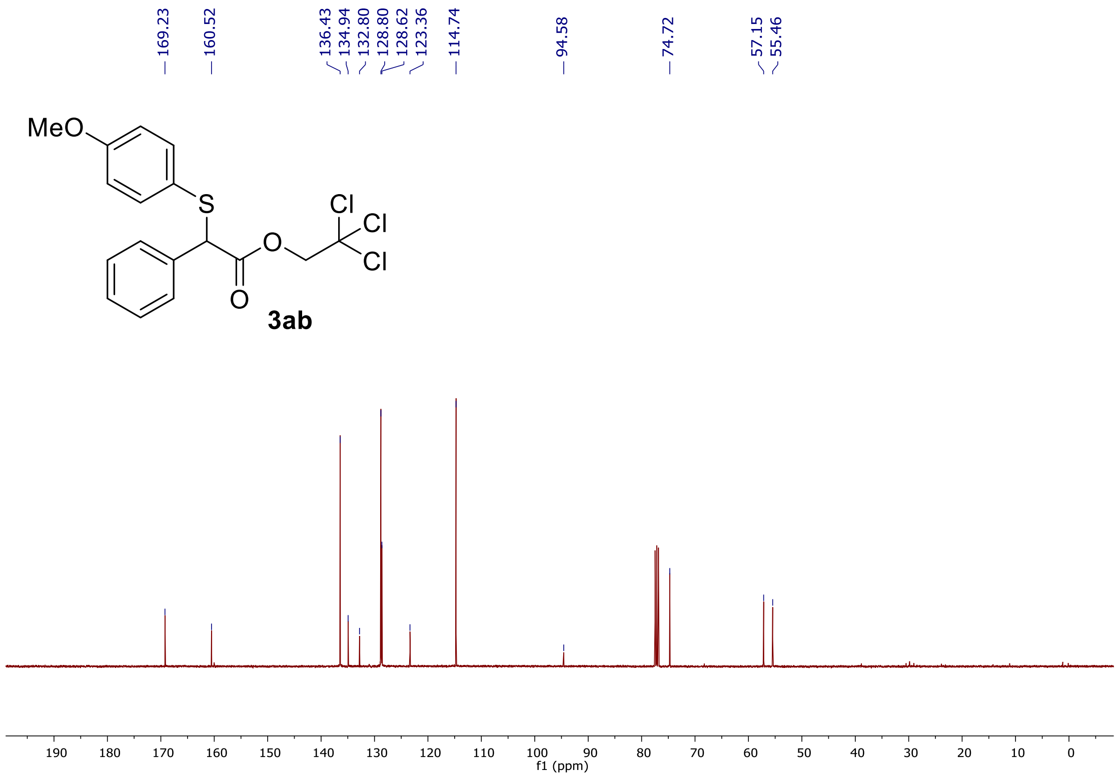

**$^{1}$H NMR (500 MHz, CDCl$_3$)** 2,2,2-trichloroethyl 2-phenyl-2-(phenylthio)acetate **3ac**

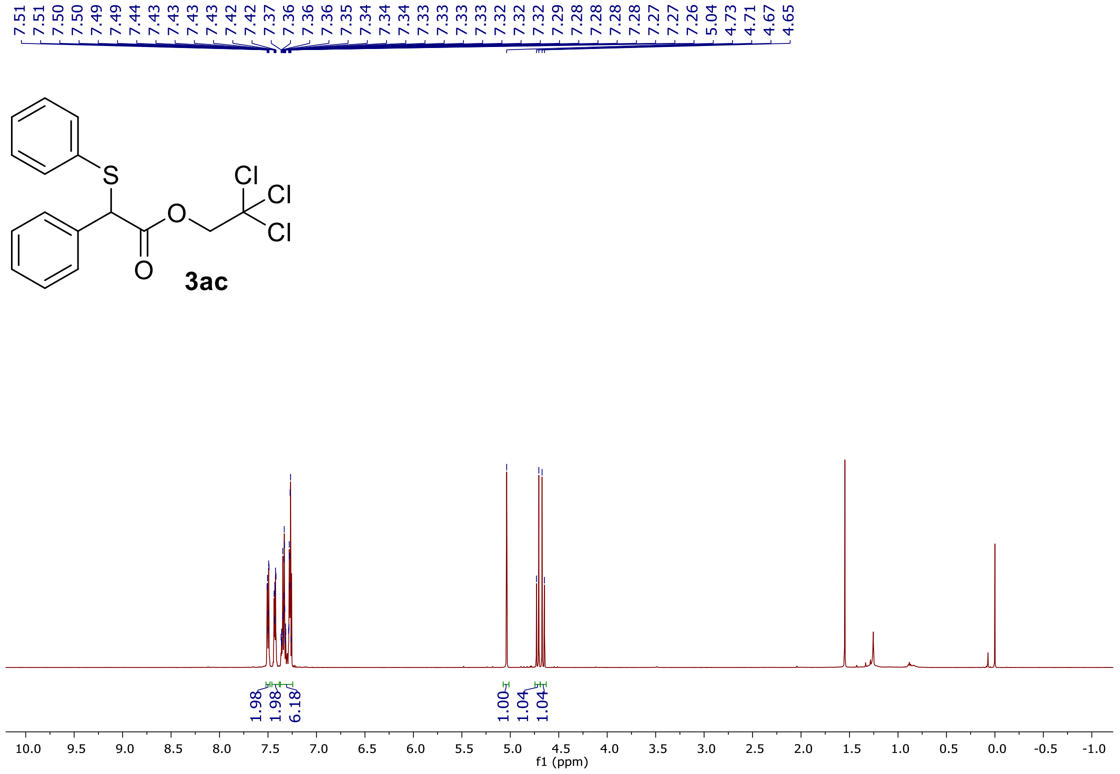


**$^{13}$C NMR (126 MHz, CDCl$_3$)** 2,2,2-trichloroethyl 2-phenyl-2-(phenylthio)acetate **3ac**

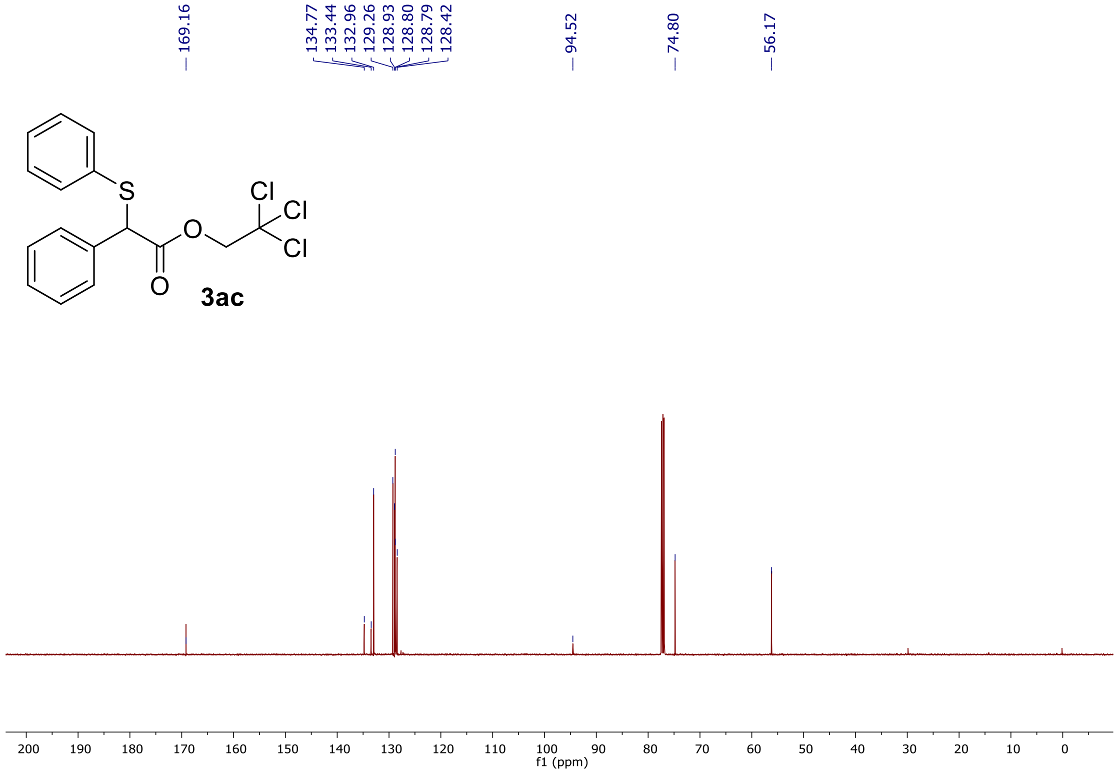

**$^1$H NMR (400 MHz, $CDCl_3$)** 2,2,2-trichloroethyl 2-((2,5-dimethylphenyl)thio)-2-phenylacetate **3ad**

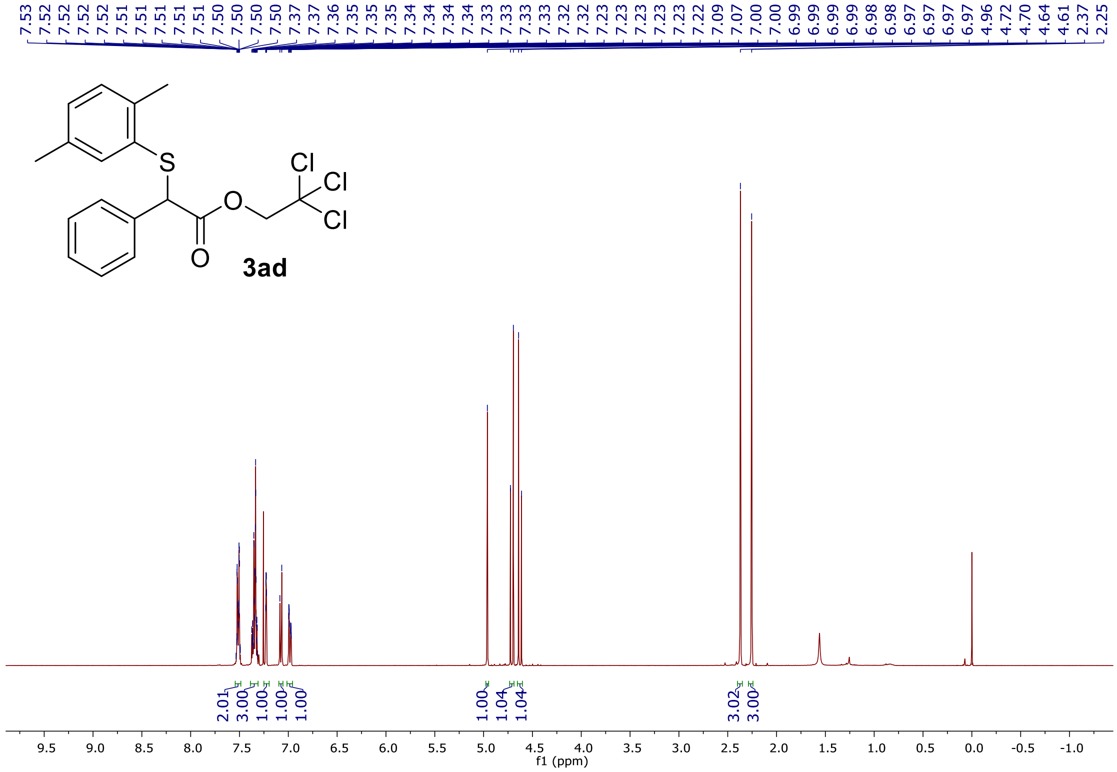


**$^{13}$C NMR (101 MHz, $CDCl_3$)** 2,2,2-trichloroethyl 2-((2,5-dimethylphenyl)thio)-2-phenylacetate **3ad**

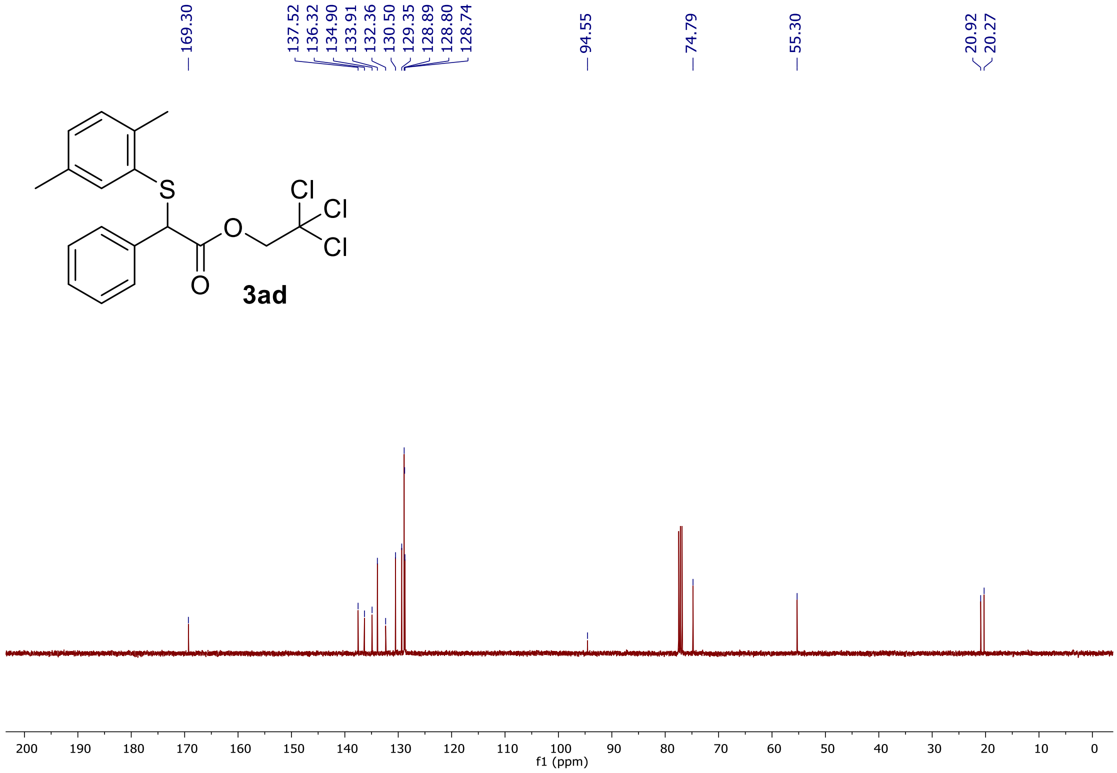

**$^1$H NMR (400 MHz, CDCl$_3$)** 2,2,2-trichloroethyl 2-phenyl-2-(p-tolylthio)acetate **3ae**

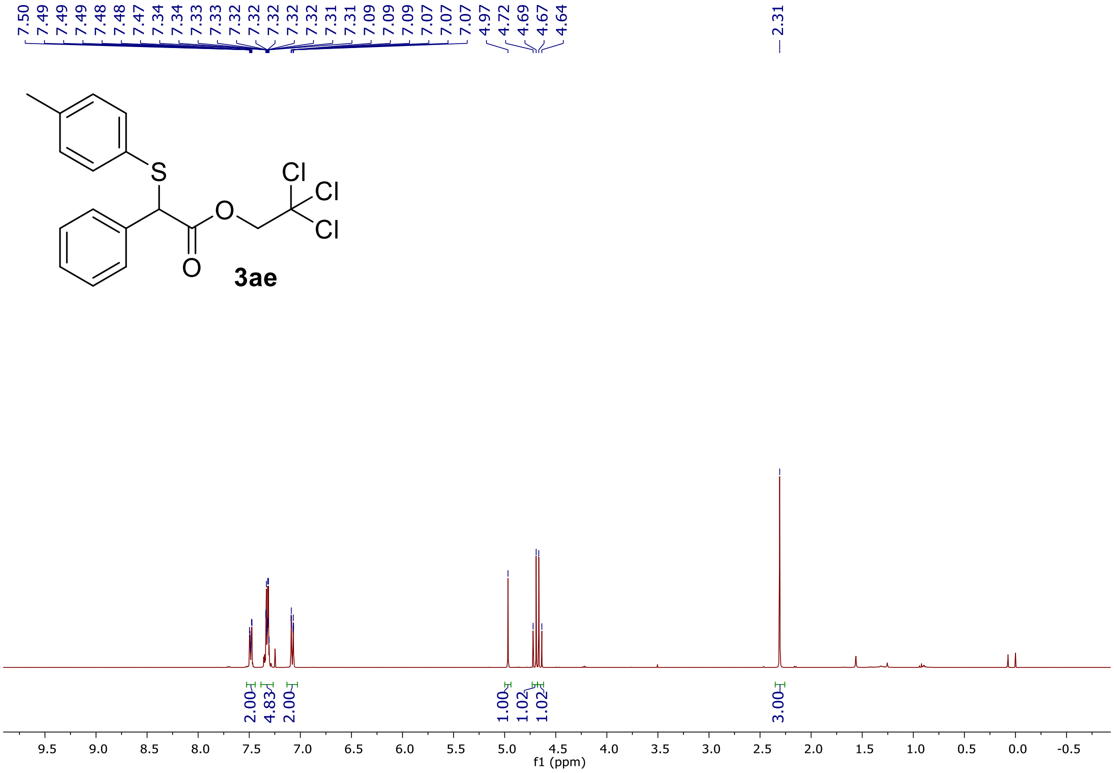


**$^{13}$C NMR (101 MHz, CDCl$_3$)** 2,2,2-trichloroethyl 2-phenyl-2-(p-tolylthio)acetate **3ae**

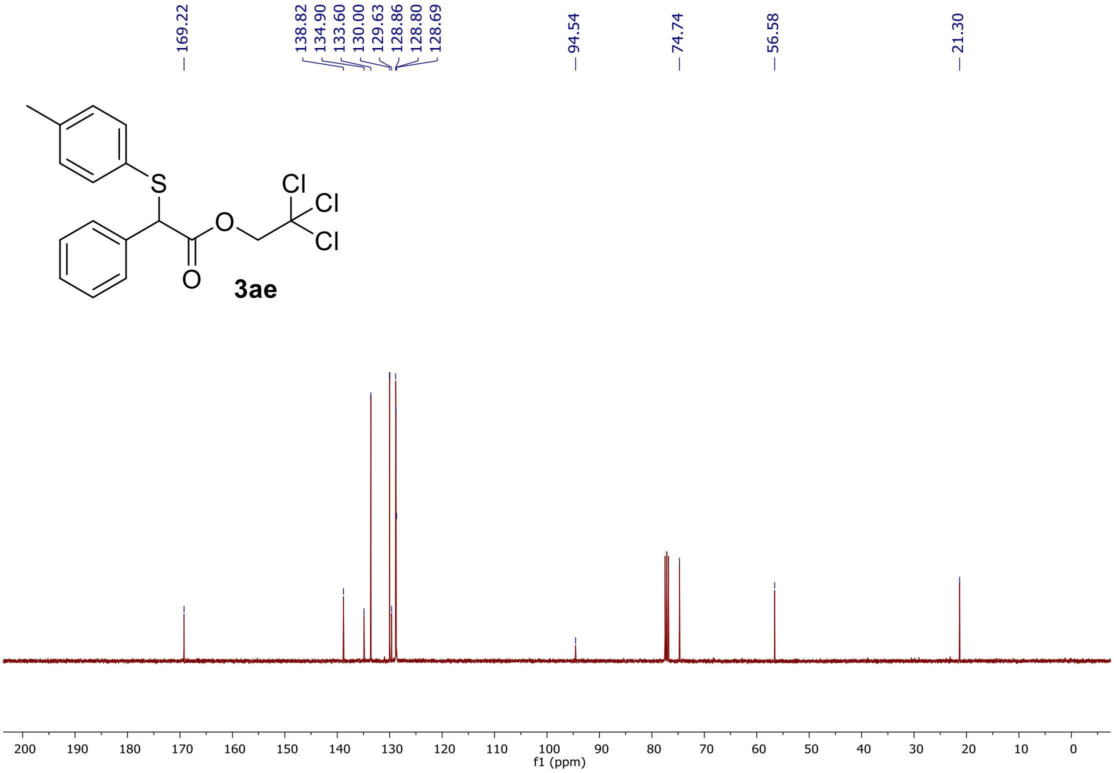

**¹H NMR (500 MHz, CDCl₃)** methyl 2-phenyl-2-(phenylsulfonyl)acetate **11**

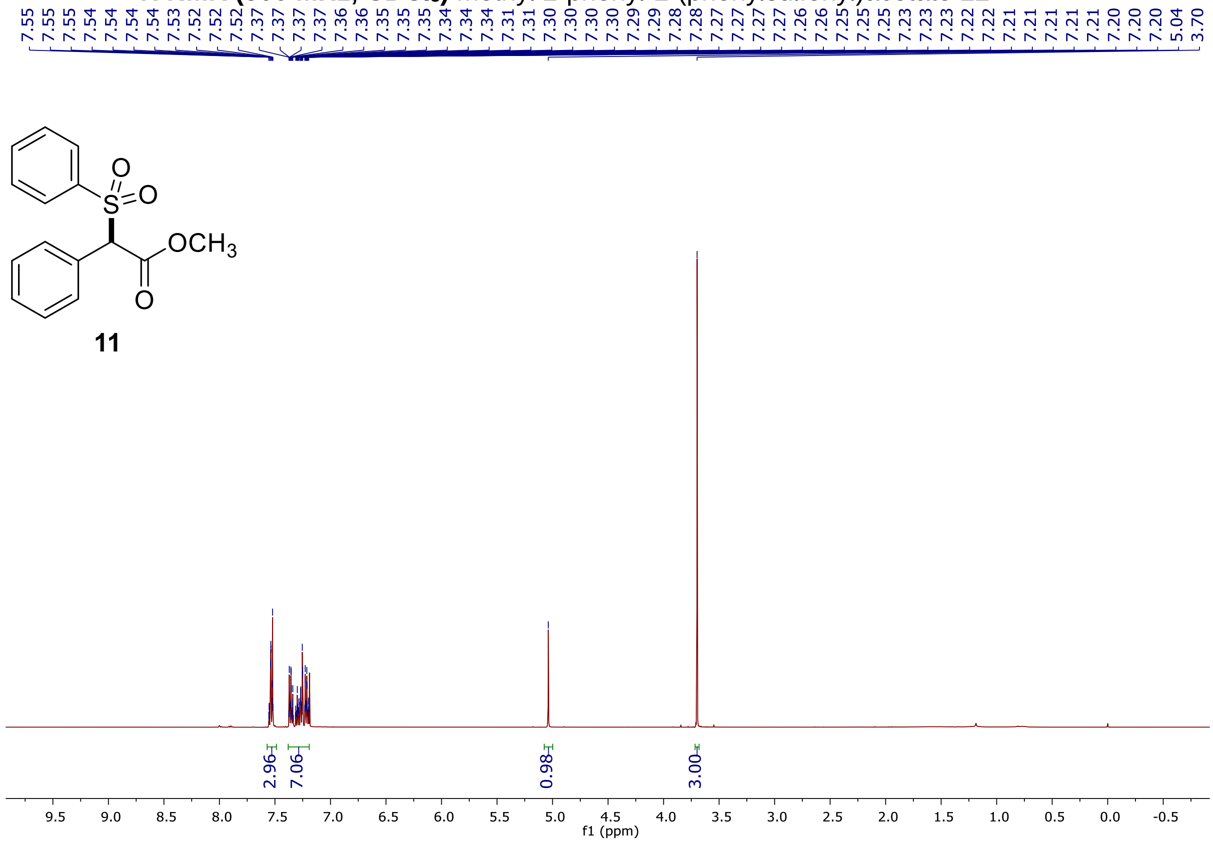


**¹³C NMR (126 MHz, CDCl₃)** methyl 2-phenyl-2-(phenylsulfonyl)acetate **11**

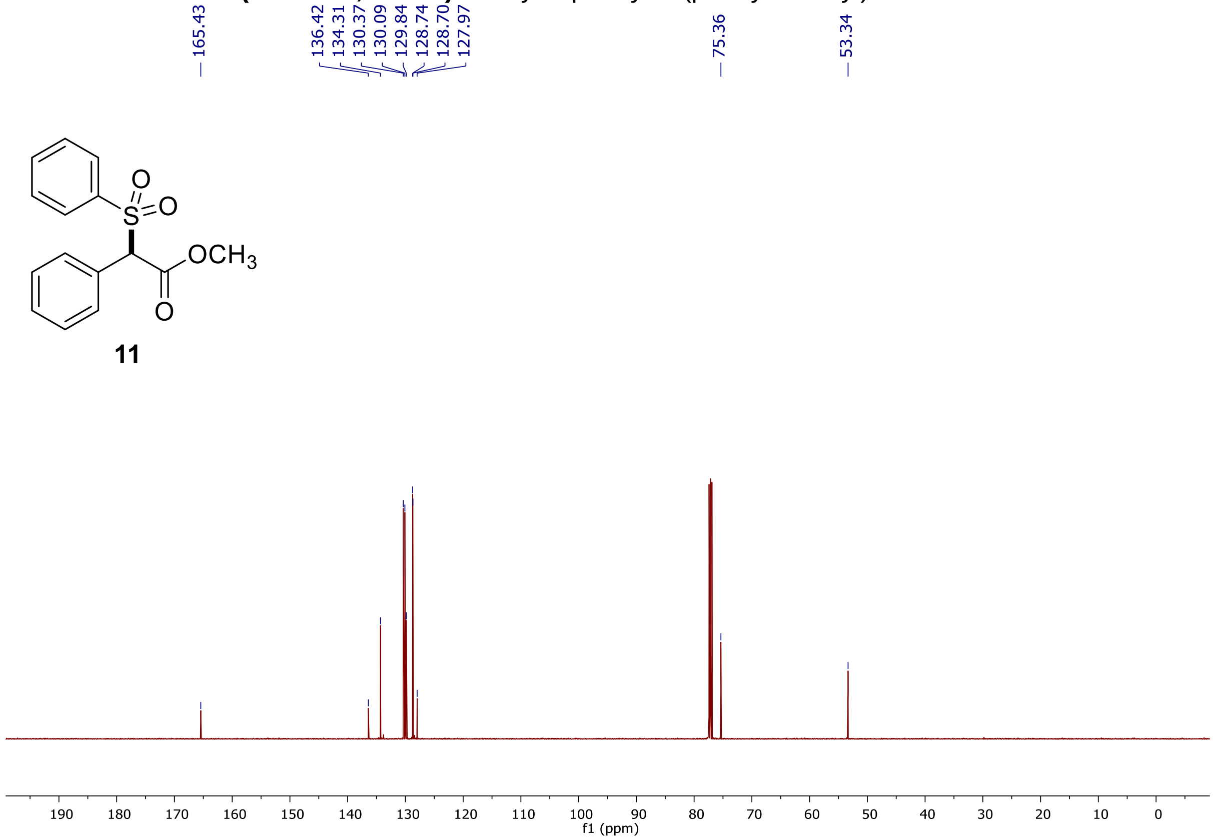

**$^{1}H$ NMR (500 MHz, CDCl$_3$)** 2-phenyl-2-(phenylthio)ethan-1-ol **12**

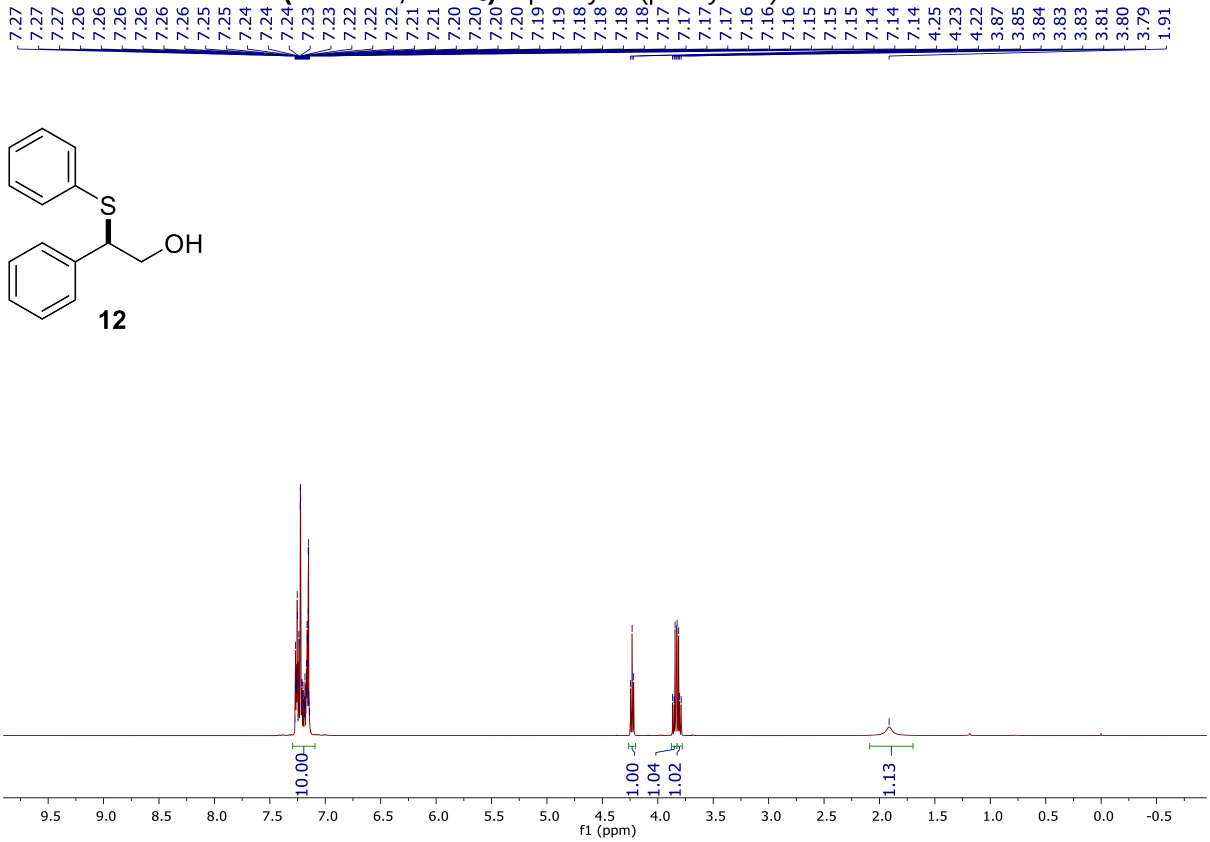


**$^{13}C$ NMR (126 MHz, CDCl$_3$)** 2-phenyl-2-(phenylthio)ethan-1-ol **12**

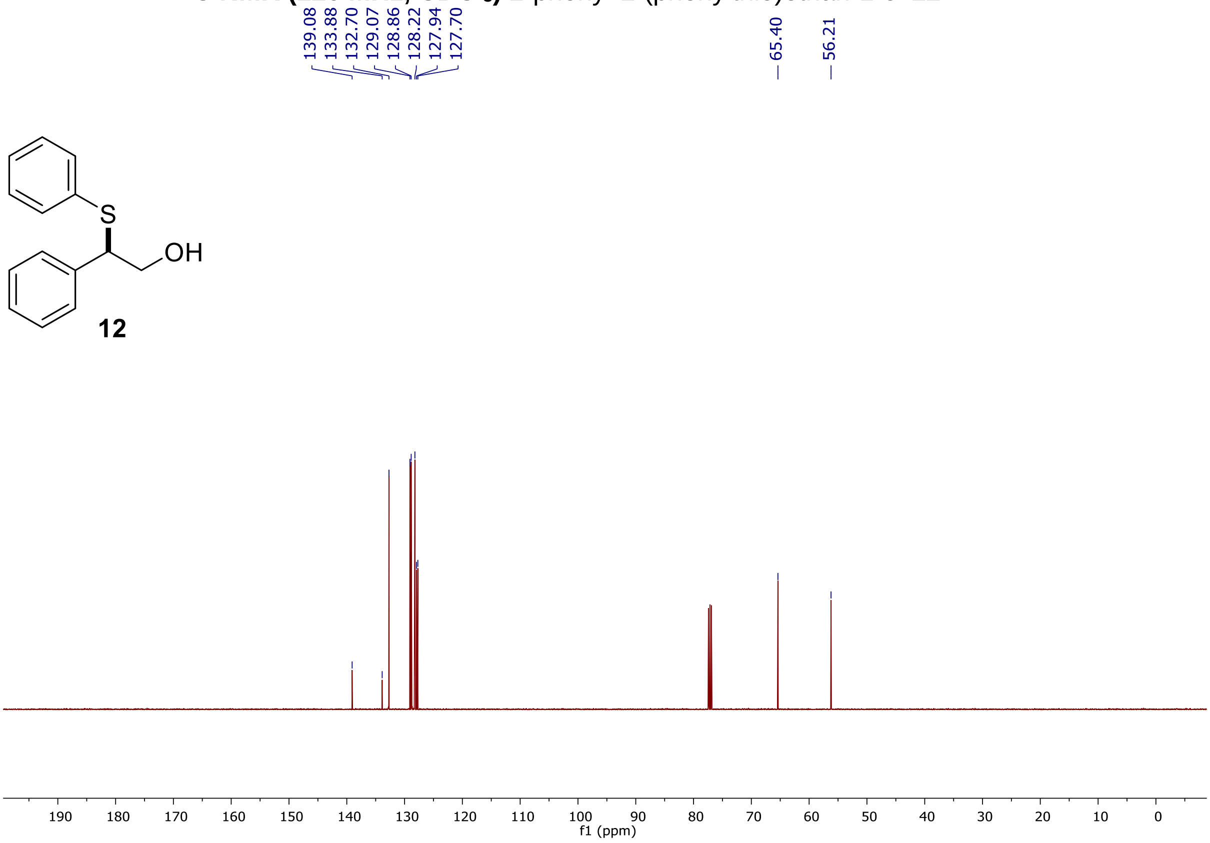

**$^{1}H$ NMR (500 MHz, $CDCl_3$)** 2-methyl-1-phenyl-1-(phenylthio)propan-2-ol **13**

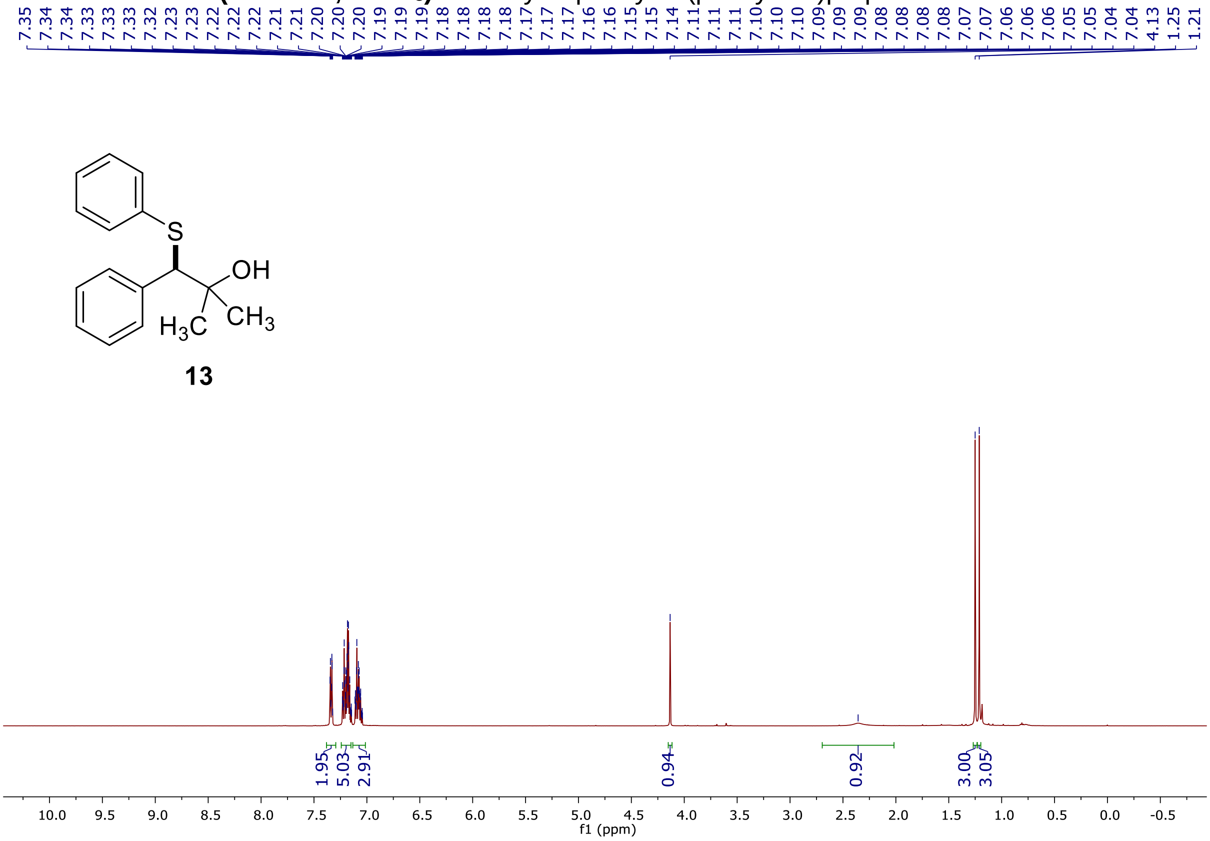


**$^{13}C$ NMR (126 MHz, $CDCl_3$)** 2-methyl-1-phenyl-1-(phenylthio)propan-2-ol **13**

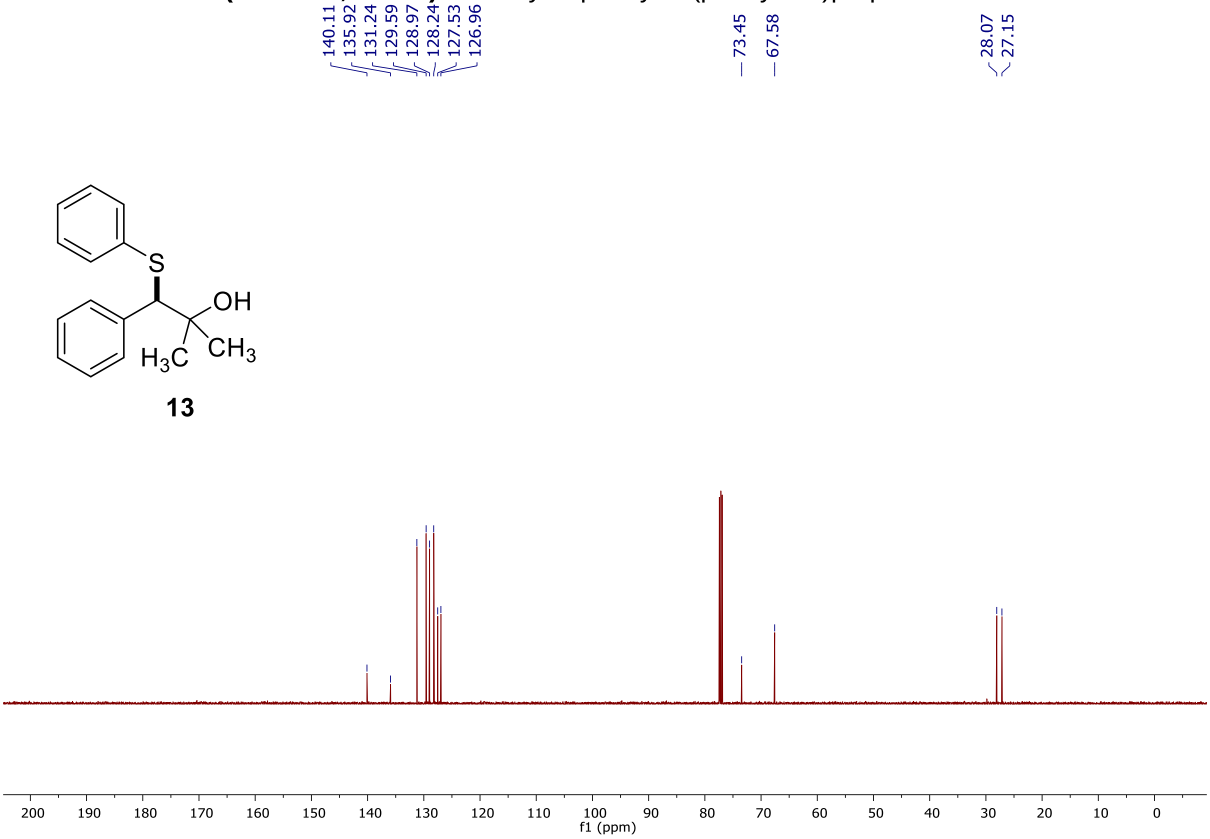

## 7. HPLC Chromatogram

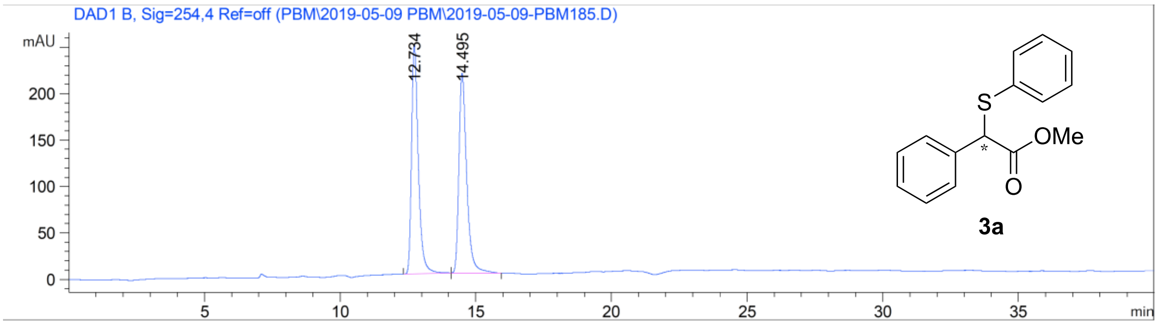


| Peak # | RetTime [min] | Type | Width [min] | Area [mAU*s] | Height [mAU] | Area % |
|---|---|---|---|---|---|---|
| 1 | 12.734 | BB | 0.2587 | 4287.51611 | 247.34314 | 49.9286 |
| 2 | 14.495 | BB | 0.2931 | 4299.78613 | 215.21329 | 50.0714 |

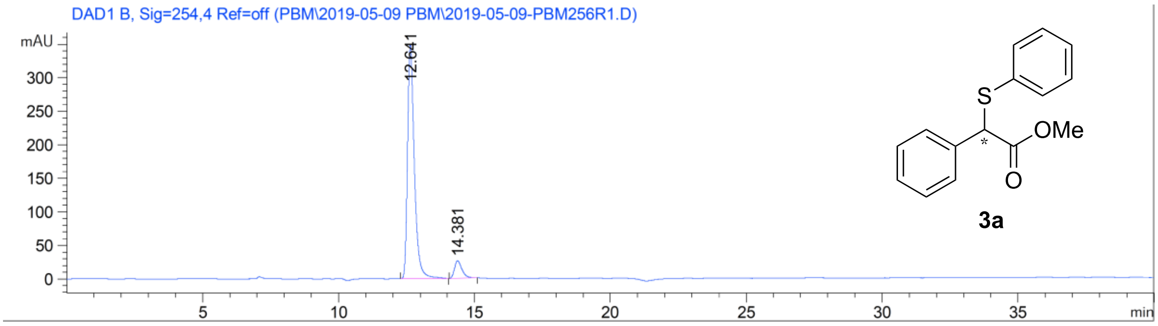


| Peak # | RetTime [min] | Type | Width [min] | Area [mAU*s] | Height [mAU] | Area % |
|---|---|---|---|---|---|---|
| 1 | 12.641 | BB | 0.2617 | 6062.21631 | 349.83530 | 92.3702 |
| 2 | 14.381 | BB | 0.2682 | 500.74197 | 26.23638 | 7.6298 |

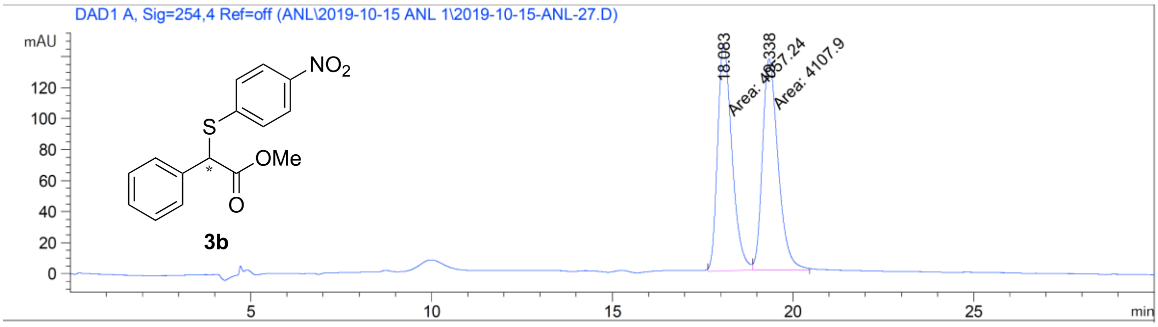


| Peak # | RetTime [min] | Type | Width [min] | Area [mAU*s] | Height [mAU] | Area % |
|---|---|---|---|---|---|---|
| 1 | 18.083 | MM | 0.4625 | 4057.23584 | 146.21498 | 49.6898 |
| 2 | 19.338 | MM | 0.5020 | 4107.89893 | 136.39131 | 50.3102 |

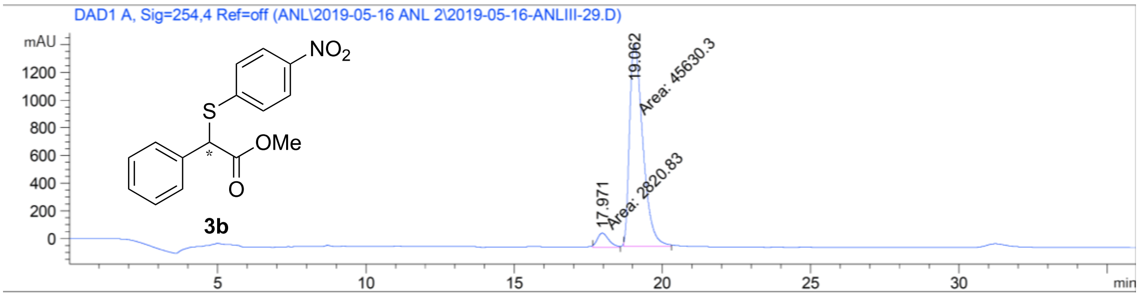


| Peak # | RetTime [min] | Type | Width [min] | Area [mAU*s] | Height [mAU] | Area % |
|---|---|---|---|---|---|---|
| 1 | 17.971 | MM | 0.4536 | 2820.82935 | 103.63873 | 5.8220 |
| 2 | 19.062 | MM | 0.5186 | 4.56303e4 | 1466.52783 | 94.1780 |

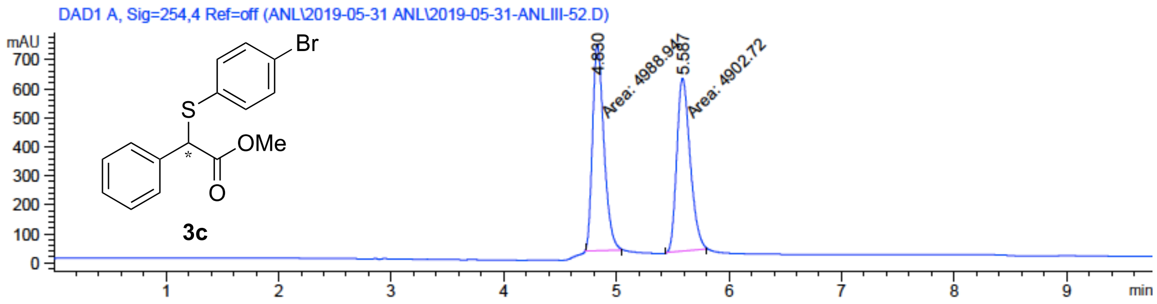


| Peak # | RetTime [min] | Type | Width [min] | Area [mAU*s] | Height [mAU] | Area % |
|---|---|---|---|---|---|---|
| 1 | 4.830 | MM | 0.1168 | 4988.94189 | 712.17615 | 50.4358 |
| 2 | 5.587 | MM | 0.1373 | 4902.71826 | 595.04065 | 49.5642 |

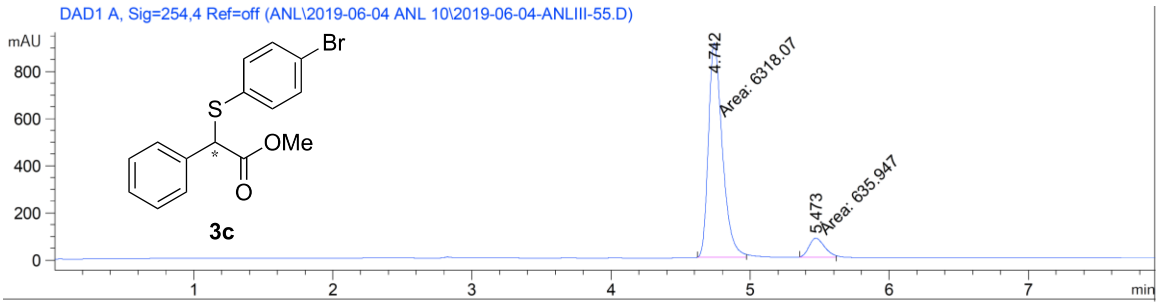


| Peak # | RetTime [min] | Type | Width [min] | Area [mAU*s] | Height [mAU] | Area % |
|---|---|---|---|---|---|---|
| 1 | 4.742 | MM | 0.1157 | 6318.06885 | 909.78101 | 90.8550 |
| 2 | 5.473 | MM | 0.1301 | 635.94714 | 81.48165 | 9.1450 |

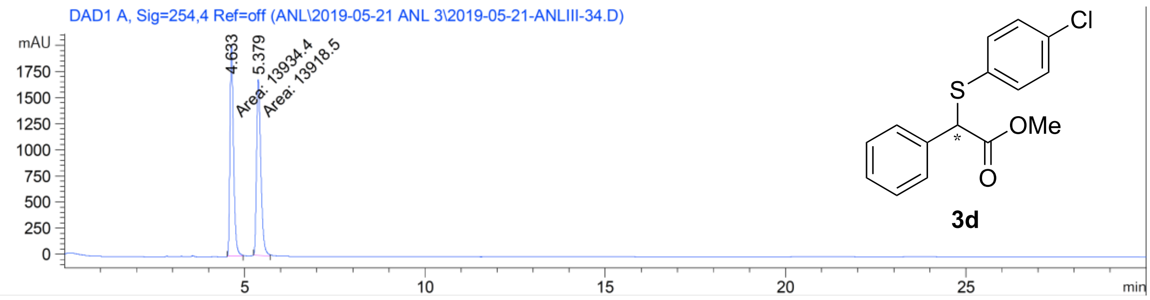


| Peak # | RetTime [min] | Type | Width [min] | Area [mAU*s] | Height [mAU] | Area % |
|---|---|---|---|---|---|---|
| 1 | 4.633 | MM | 0.1145 | 1.39344e4 | 2028.22925 | 50.0286 |
| 2 | 5.379 | MM | 0.1376 | 1.39185e4 | 1686.20532 | 49.9714 |

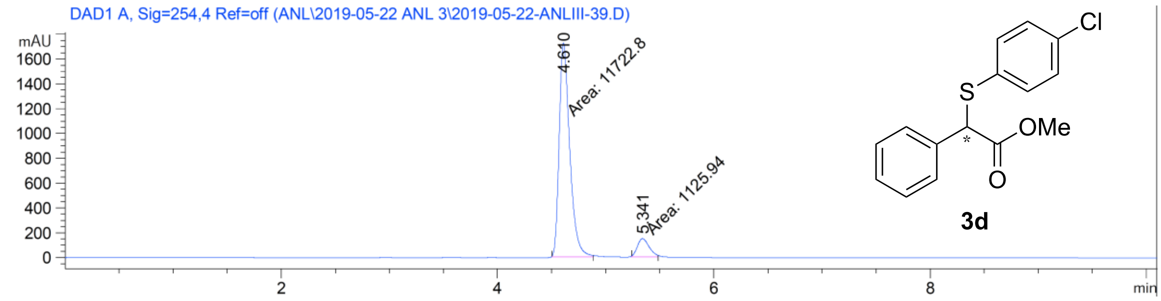


| Peak # | RetTime [min] | Type | Width [min] | Area [mAU*s] | Height [mAU] | Area % |
|---|---|---|---|---|---|---|
| 1 | 4.610 | MM | 0.1133 | 1.17228e4 | 1725.06494 | 91.2369 |
| 2 | 5.341 | MM | 0.1263 | 1125.94202 | 148.60855 | 8.7631 |

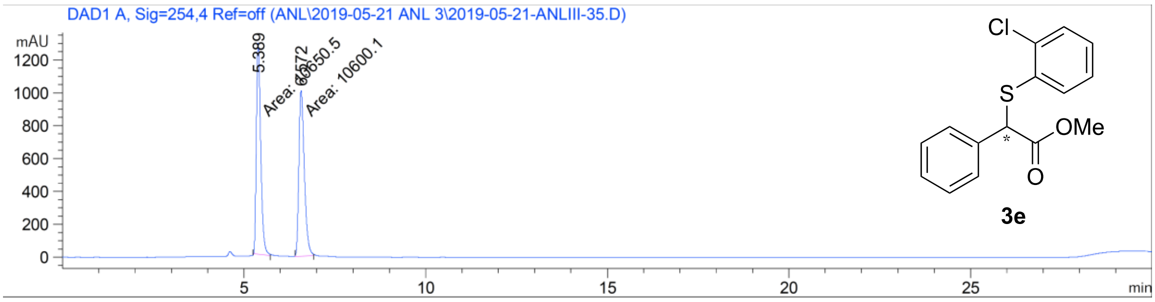


| Peak # | RetTime [min] | Type | Width [min] | Area [mAU*s] | Height [mAU] | Area % |
|---|---|---|---|---|---|---|
| 1 | 5.389 | MM | 0.1384 | 1.06505e4 | 1282.83325 | 50.1188 |
| 2 | 6.572 | MM | 0.1753 | 1.06001e4 | 1007.62024 | 49.8812 |

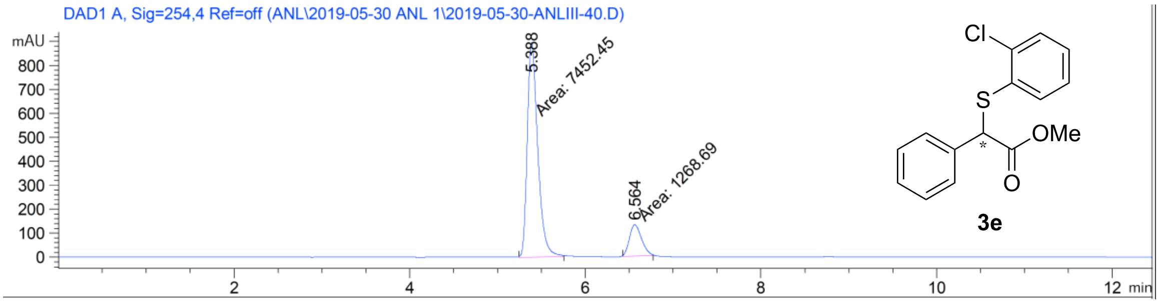


| Peak # | RetTime [min] | Type | Width [min] | Area [mAU*s] | Height [mAU] | Area % |
|---|---|---|---|---|---|---|
| 1 | 5.388 | MM | 0.1387 | 7452.44678 | 895.69061 | 85.4527 |
| 2 | 6.564 | MM | 0.1610 | 1268.69080 | 131.29871 | 14.5473 |

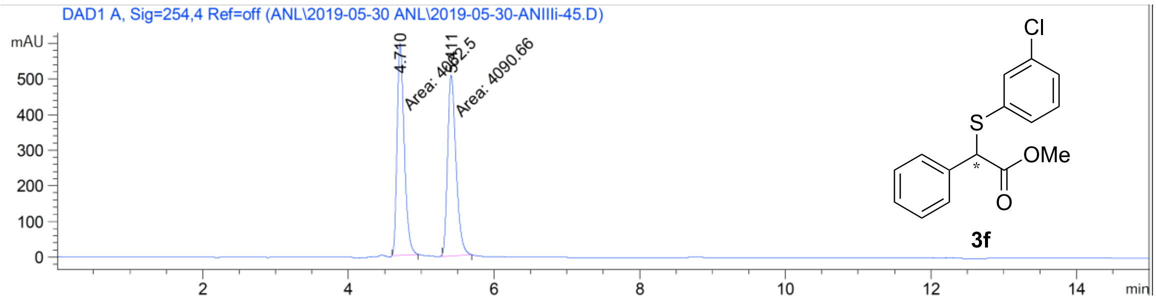


| Peak # | RetTime [min] | Type | Width [min] | Area [mAU*s] | Height [mAU] | Area % |
|---|---|---|---|---|---|---|
| 1 | 4.710 | MM | 0.1138 | 4062.50439 | 594.99518 | 49.8273 |
| 2 | 5.411 | MM | 0.1343 | 4090.66113 | 507.56866 | 50.1727 |

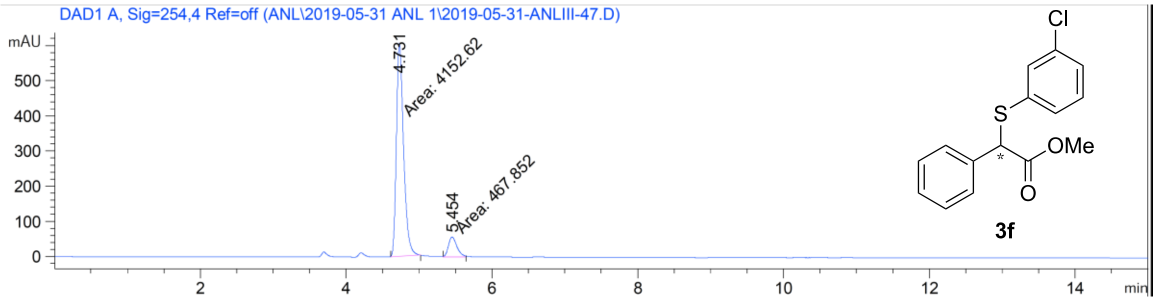


| Peak # | RetTime [min] | Type | Width [min] | Area [mAU*s] | Height [mAU] | Area % |
|---|---|---|---|---|---|---|
| 1 | 4.731 | MM | 0.1141 | 4152.61914 | 606.47009 | 89.8744 |
| 2 | 5.454 | MM | 0.1358 | 467.85150 | 57.41867 | 10.1256 |

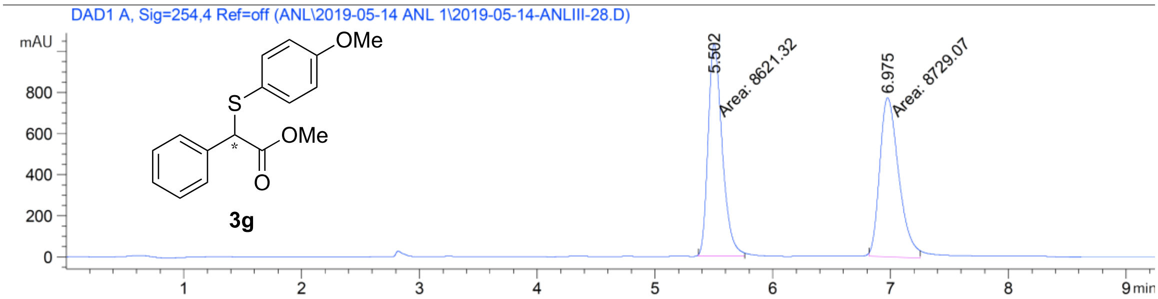


| Peak # | RetTime [min] | Type | Width [min] | Area [mAU*s] | Height [mAU] | Area % |
|---|---|---|---|---|---|---|
| 1 | 5.502 | MM | 0.1389 | 8621.31738 | 1034.63025 | 49.6895 |
| 2 | 6.975 | MM | 0.1876 | 8729.06641 | 775.37866 | 50.3105 |

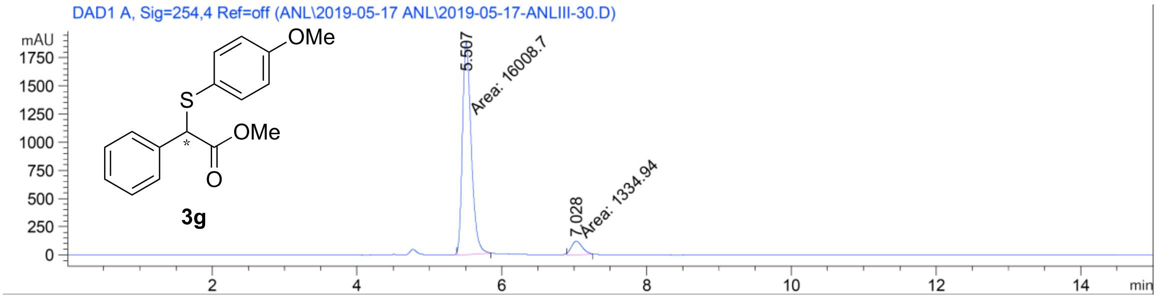


| Peak # | RetTime [min] | Type | Width [min] | Area [mAU*s] | Height [mAU] | Area % |
|---|---|---|---|---|---|---|
| 1 | 5.507 | MM | 0.1409 | 1.60087e4 | 1892.98438 | 92.3030 |
| 2 | 7.028 | MM | 0.1811 | 1334.94348 | 122.87484 | 7.6970 |

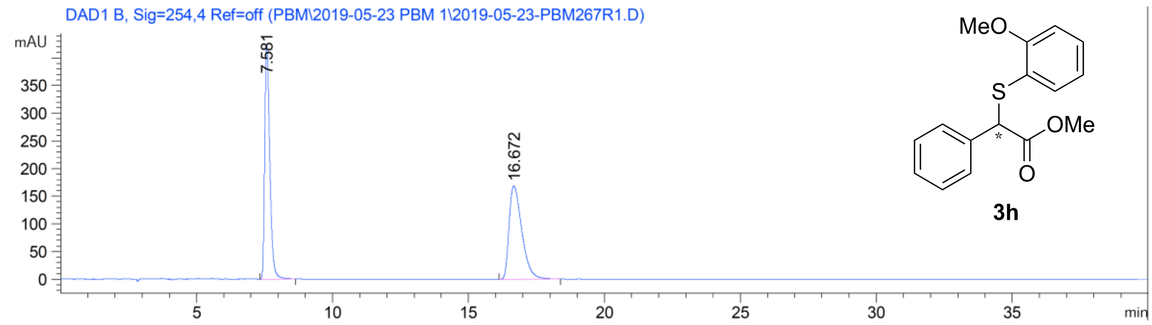


| Peak # | RetTime [min] | Type | Width [min] | Area [mAU*s] | Height [mAU] | Area % |
|---|---|---|---|---|---|---|
| 1 | 7.581 | BB | 0.2003 | 5549.55859 | 423.63123 | 50.0780 |
| 2 | 16.672 | BB | 0.4522 | 5532.26855 | 169.11491 | 49.9220 |

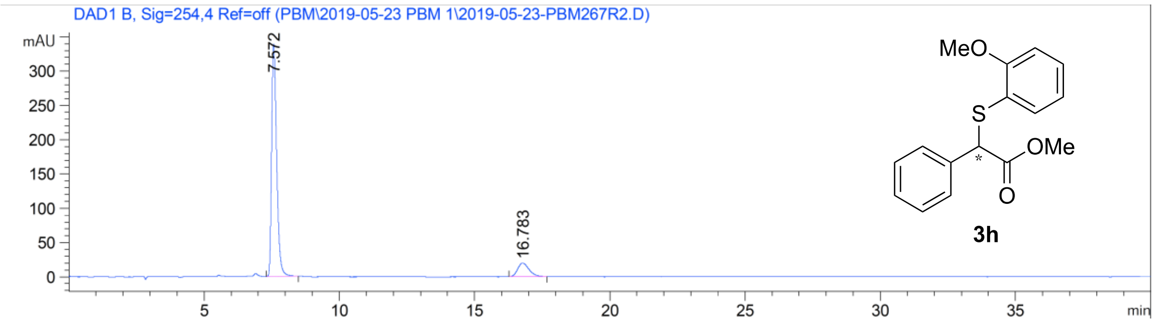


| Peak # | RetTime [min] | Type | Width [min] | Area [mAU*s] | Height [mAU] | Area % |
|---|---|---|---|---|---|---|
| 1 | 7.572 | BB | 0.1989 | 4383.11328 | 338.87076 | 88.3213 |
| 2 | 16.783 | BB | 0.3533 | 579.57922 | 19.72390 | 11.6787 |

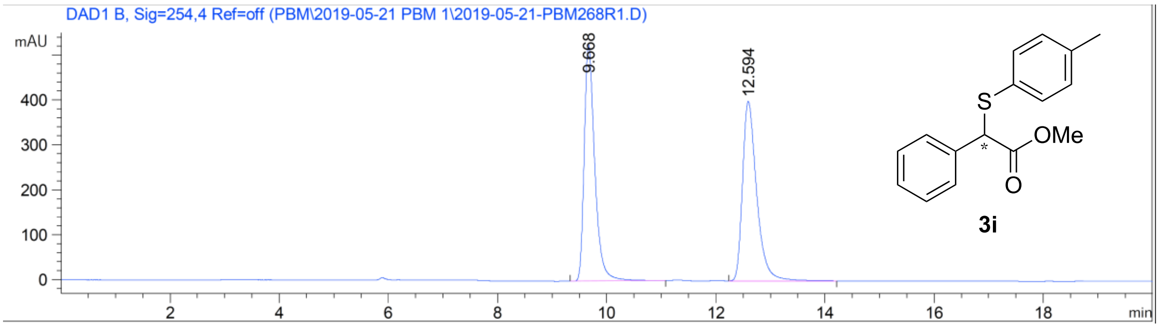


| Peak # | RetTime [min] | Type | Width [min] | Area [mAU*s] | Height [mAU] | Area % |
|---|---|---|---|---|---|---|
| 1 | 9.668 | BB | 0.2026 | 7075.19678 | 526.94604 | 49.6218 |
| 2 | 12.594 | BB | 0.2719 | 7183.04688 | 399.18561 | 50.3782 |

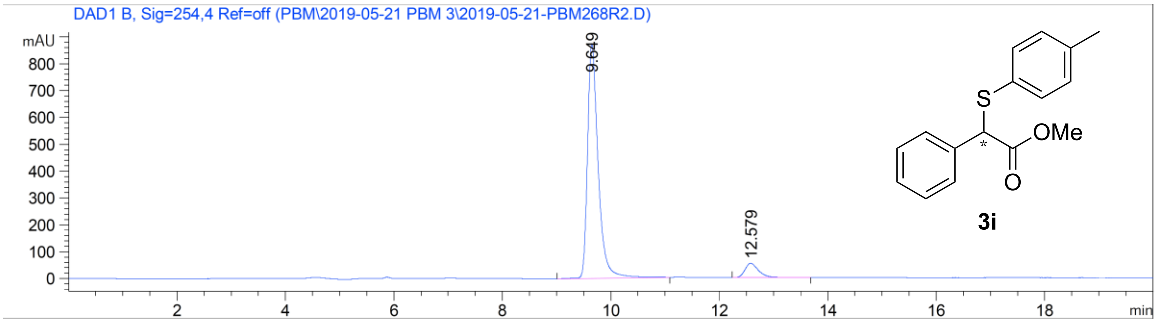


| Peak # | RetTime [min] | Type | Width [min] | Area [mAU*s] | Height [mAU] | Area % |
|---|---|---|---|---|---|---|
| 1 | 9.649 | BB | 0.2047 | 1.18921e4 | 874.03796 | 92.7290 |
| 2 | 12.579 | BB | 0.2627 | 932.47021 | 53.14710 | 7.2710 |

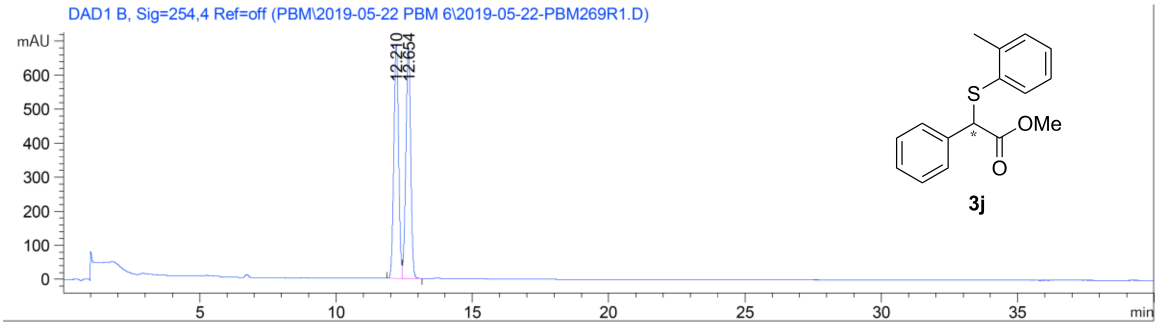


| Peak # | RetTime [min] | Type | Width [min] | Area [mAU*s] | Height [mAU] | Area % |
|---|---|---|---|---|---|---|
| 1 | 12.210 | BV | 0.1895 | 8323.66211 | 688.56458 | 49.9466 |
| 2 | 12.654 | VB | 0.1934 | 8341.45117 | 676.36066 | 50.0534 |

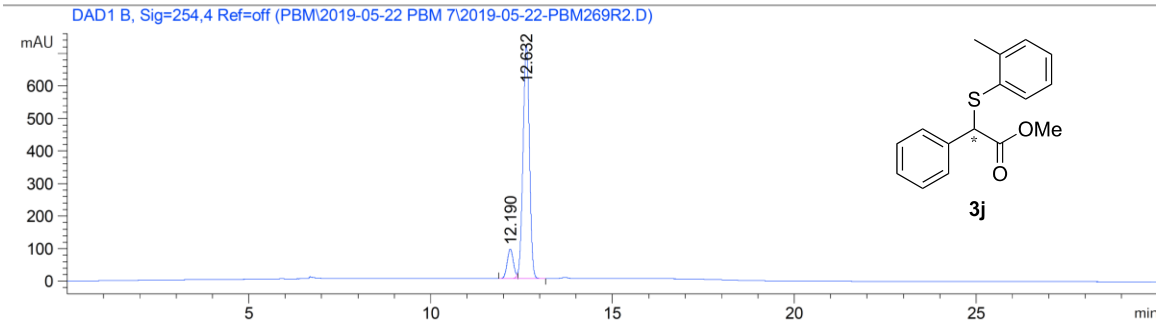


| Peak # | RetTime [min] | Type | Width [min] | Area [mAU*s] | Height [mAU] | Area % |
|---|---|---|---|---|---|---|
| 1 | 12.190 | BV E | 0.1836 | 1057.93762 | 90.67658 | 10.6690 |
| 2 | 12.632 | VB R | 0.1932 | 8858.07617 | 716.70190 | 89.3310 |

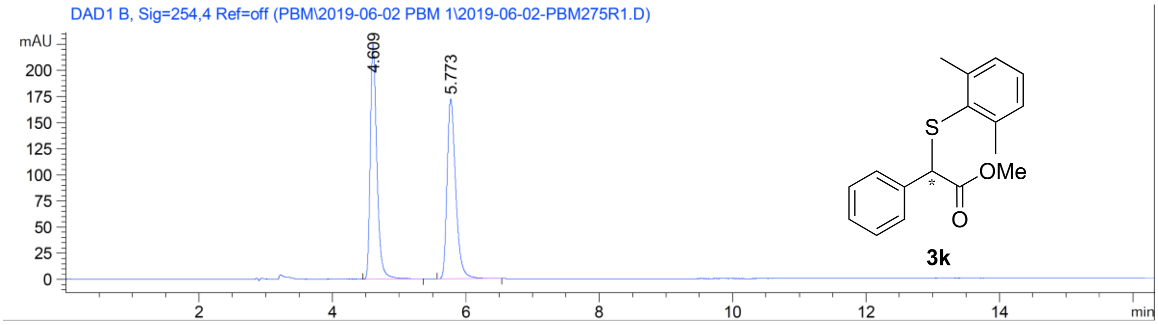


| Peak # | RetTime [min] | Type | Width [min] | Area [mAU*s] | Height [mAU] | Area % |
|---|---|---|---|---|---|---|
| 1 | 4.609 | BB | 0.1054 | 1549.75476 | 224.89700 | 50.0517 |
| 2 | 5.773 | BB | 0.1377 | 1546.55615 | 172.25754 | 49.9483 |

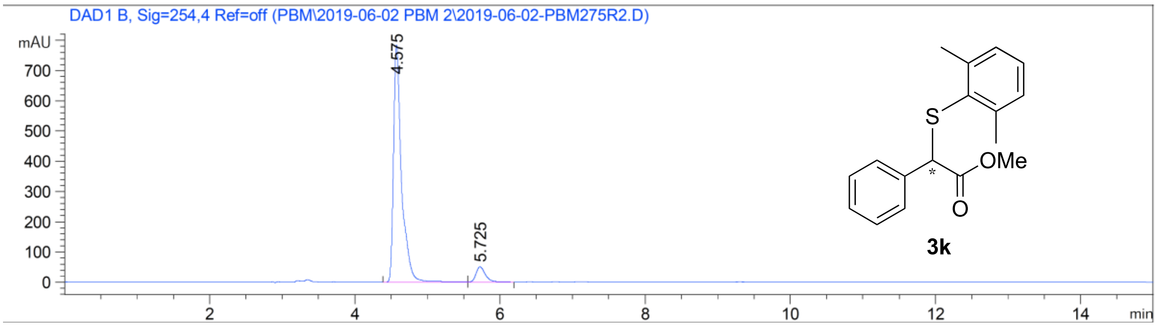


| Peak # | RetTime [min] | Type | Width [min] | Area [mAU*s] | Height [mAU] | Area % |
|---|---|---|---|---|---|---|
| 1 | 4.575 | BV R | 0.1129 | 5989.56006 | 782.56653 | 93.0793 |
| 2 | 5.725 | VB | 0.1344 | 445.33978 | 50.26474 | 6.9207 |

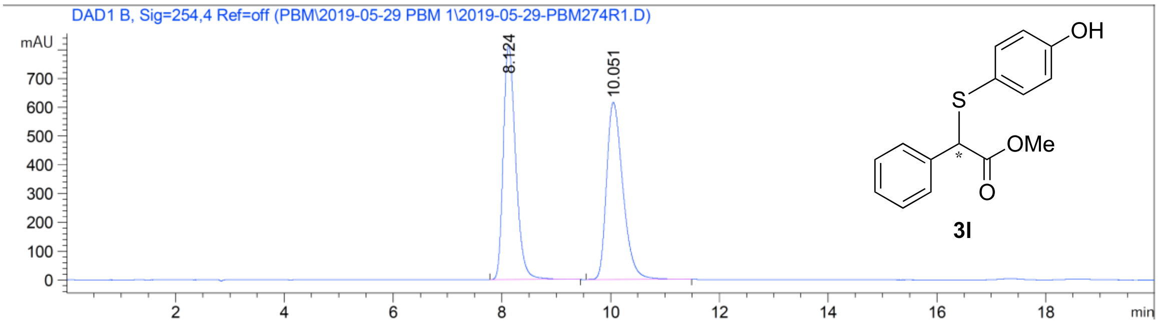


| Peak # | RetTime [min] | Type | Width [min] | Area [mAU*s] | Height [mAU] | Area % |
|---|---|---|---|---|---|---|
| 1 | 8.124 | BB | 0.2423 | 1.28294e4 | 814.54883 | 50.0365 |
| 2 | 10.051 | BB | 0.3164 | 1.28107e4 | 615.26337 | 49.9635 |

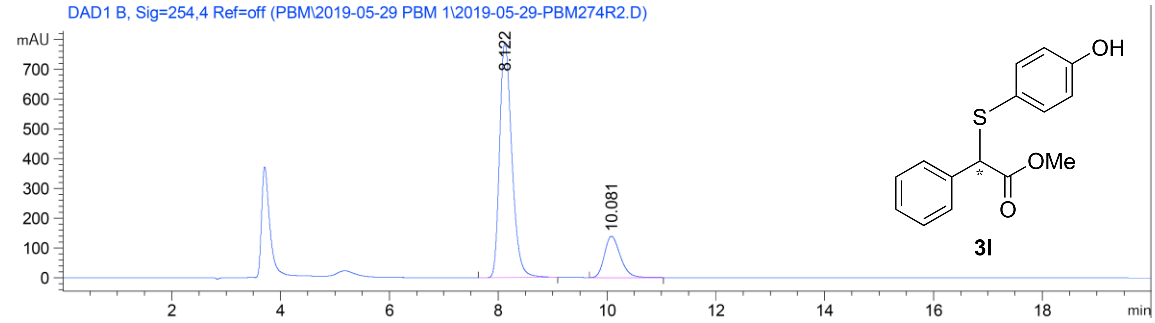


| Peak # | RetTime [min] | Type | Width [min] | Area [mAU*s] | Height [mAU] | Area % |
|---|---|---|---|---|---|---|
| 1 | 8.122 | BB | 0.2422 | 1.23829e4 | 788.40338 | 81.5552 |
| 2 | 10.081 | BB | 0.3059 | 2800.54736 | 138.50043 | 18.4448 |

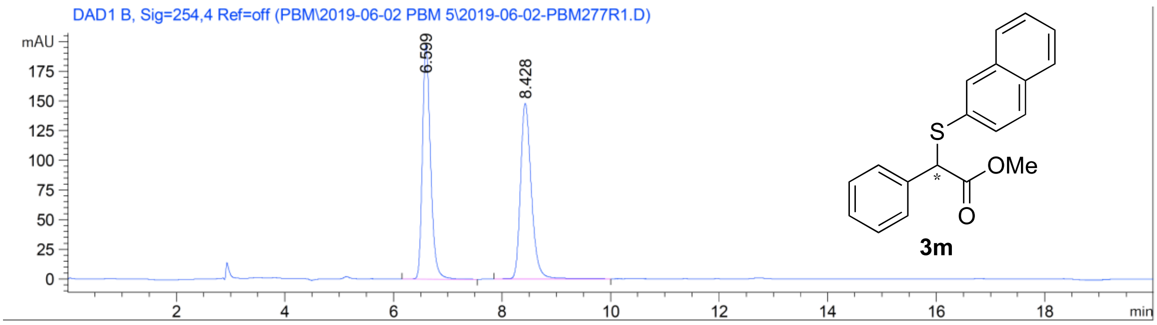


| Peak # | RetTime [min] | Type | Width [min] | Area [mAU*s] | Height [mAU] | Area % |
|---|---|---|---|---|---|---|
| 1 | 6.599 | BB | 0.1615 | 2057.32031 | 197.60246 | 49.8714 |
| 2 | 8.428 | BB | 0.2128 | 2067.92749 | 148.14830 | 50.1286 |

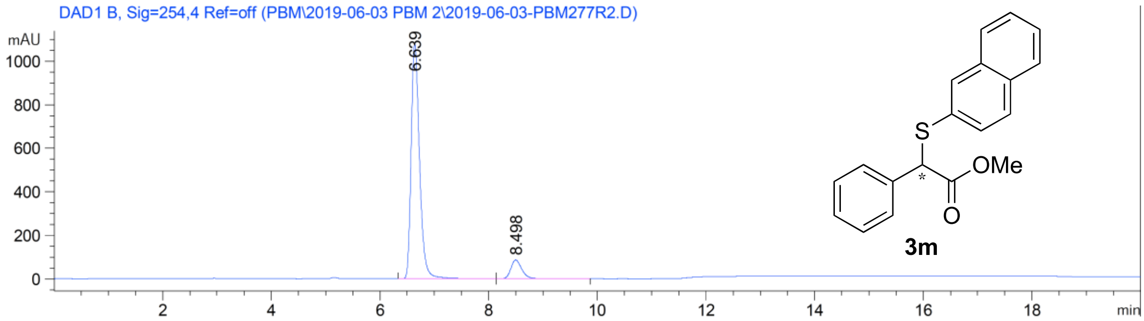


| Peak # | RetTime [min] | Type | Width [min] | Area [mAU*s] | Height [mAU] | Area % |
|---|---|---|---|---|---|---|
| 1 | 6.639 | BV | 0.1654 | 1.16576e4 | 1084.25684 | 90.4936 |
| 2 | 8.498 | VB | 0.2172 | 1224.63623 | 86.46131 | 9.5064 |

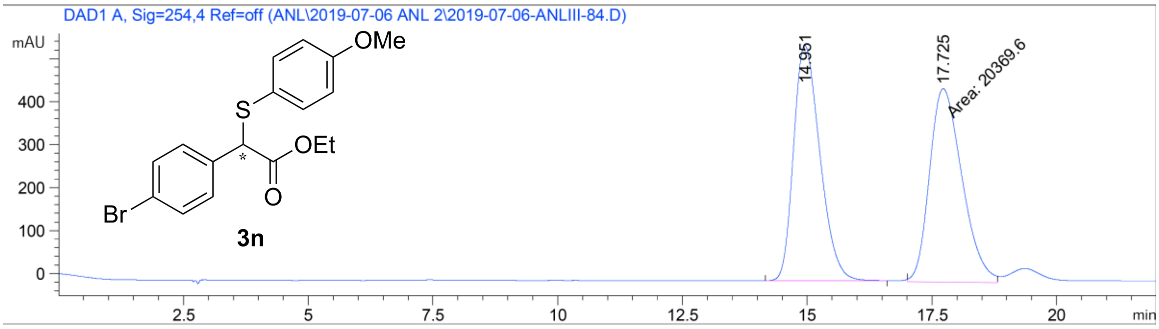


| Peak # | RetTime [min] | Type | Width [min] | Area [mAU*s] | Height [mAU] | Area % |
|---|---|---|---|---|---|---|
| 1 | 14.951 | BB | 0.5620 | 2.00991e4 | 547.18494 | 49.6658 |
| 2 | 17.725 | MM | 0.7556 | 2.03696e4 | 449.27490 | 50.3342 |

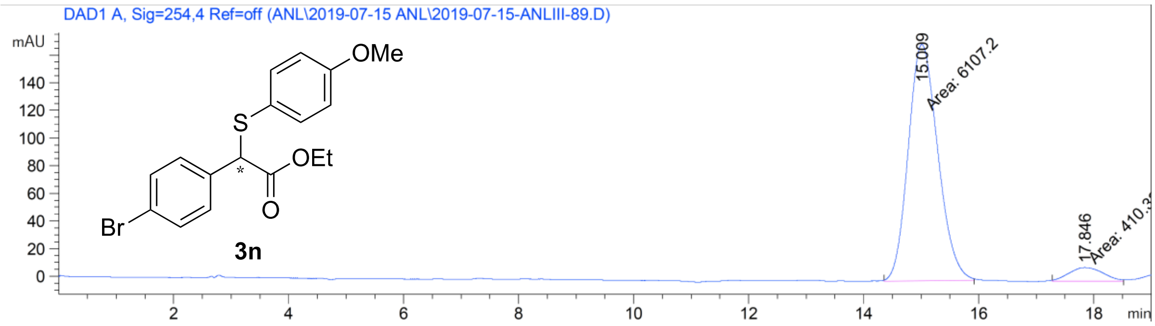


| Peak # | RetTime [min] | Type | Width [min] | Area [mAU*s] | Height [mAU] | Area % |
|---|---|---|---|---|---|---|
| 1 | 15.009 | MM | 0.5956 | 6107.19922 | 170.89825 | 93.7043 |
| 2 | 17.846 | MM | 0.6891 | 410.32306 | 9.92463 | 6.2957 |

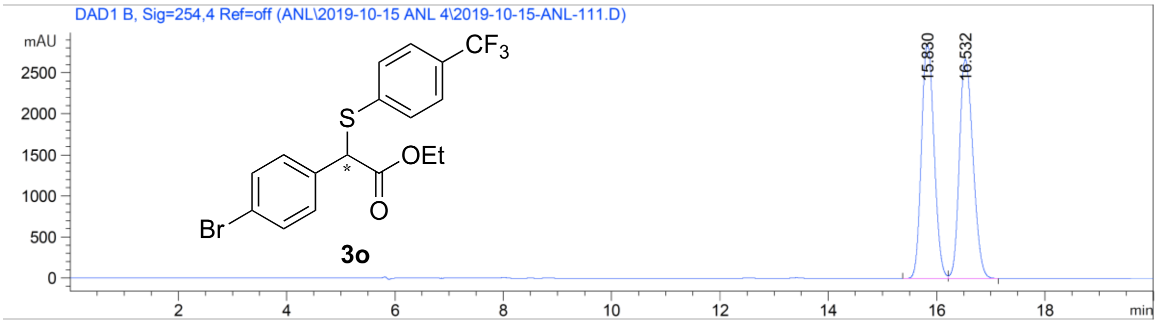


| Peak # | RetTime [min] | Type | Width [min] | Area [mAU*s] | Height [mAU] | Area % |
|---|---|---|---|---|---|---|
| 1 | 15.830 | BV | 0.1914 | 4.62993e4 | 2851.61182 | 49.8137 |
| 2 | 16.532 | VB | 0.2106 | 4.66456e4 | 2666.55591 | 50.1863 |

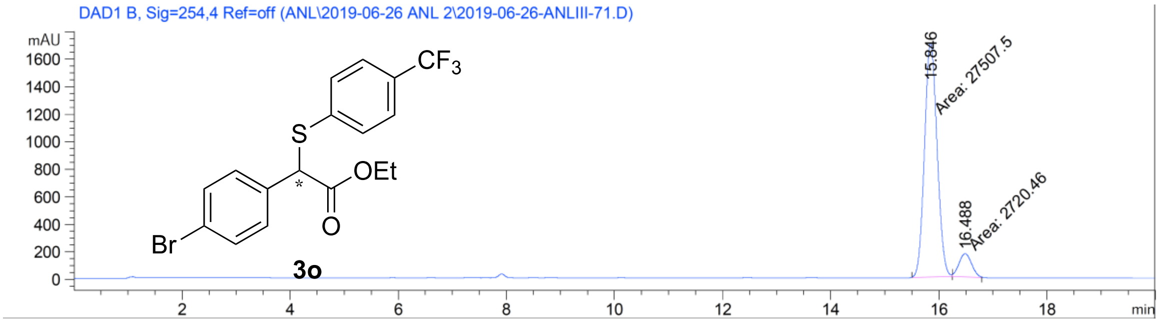


| Peak # | RetTime [min] | Type | Width [min] | Area [mAU*s] | Height [mAU] | Area % |
|---|---|---|---|---|---|---|
| 1 | 15.846 | MM | 0.2697 | 2.75075e4 | 1699.82117 | 91.0002 |
| 2 | 16.488 | MM | 0.2692 | 2720.46338 | 168.45412 | 8.9998 |

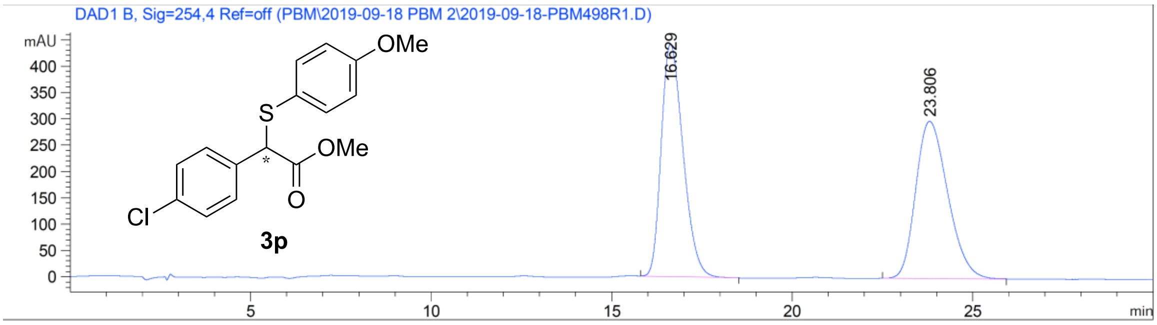


| Peak # | RetTime [min] | Type | Width [min] | Area [mAU*s] | Height [mAU] | Area % |
|---|---|---|---|---|---|---|
| 1 | 16.629 | BB | 0.5956 | 1.85462e4 | 441.07492 | 50.1879 |
| 2 | 23.806 | BB | 0.7255 | 1.84074e4 | 298.46500 | 49.8121 |

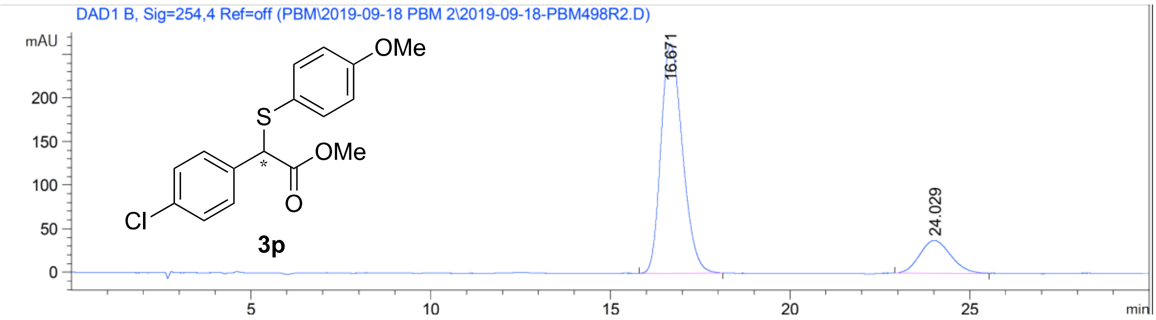


| Peak # | RetTime [min] | Type | Width [min] | Area [mAU*s] | Height [mAU] | Area % |
|---|---|---|---|---|---|---|
| 1 | 16.671 | BB | 0.5790 | 1.09625e4 | 263.41971 | 82.9918 |
| 2 | 24.029 | BB | 0.7017 | 2246.63110 | 37.56859 | 17.0082 |

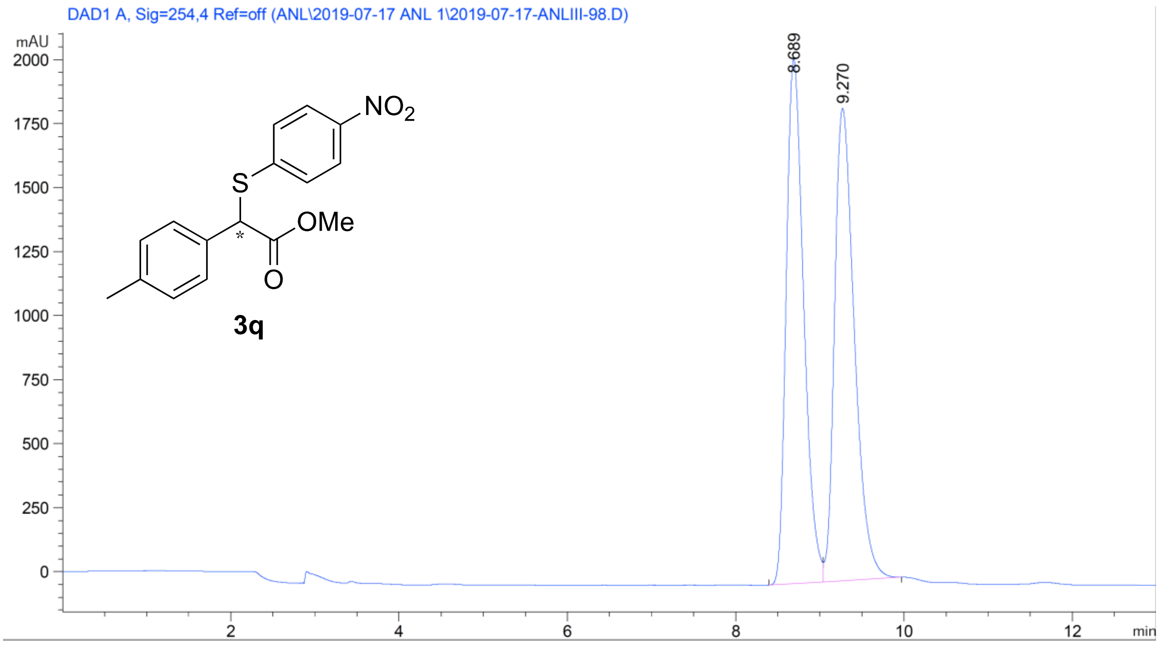


| Peak # | RetTime [min] | Type | Width [min] | Area [mAU*s] | Height [mAU] | Area % |
|---|---|---|---|---|---|---|
| 1 | 8.689 | BV | 0.2240 | 2.96029e4 | 2049.30640 | 49.4743 |
| 2 | 9.270 | VB | 0.2526 | 3.02320e4 | 1845.24121 | 50.5257 |

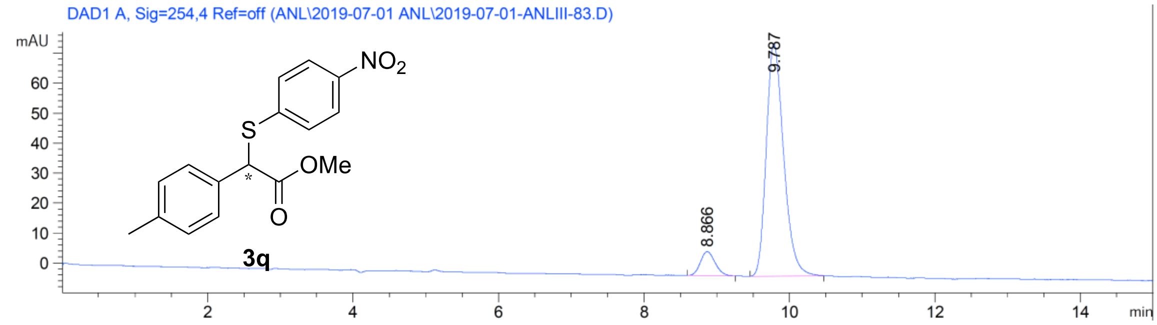


| Peak # | RetTime [min] | Type | Width [min] | Area [mAU*s] | Height [mAU] | Area % |
|---|---|---|---|---|---|---|
| 1 | 8.866 | BB | 0.2011 | 111.80010 | 8.01981 | 8.0185 |
| 2 | 9.787 | BB | 0.2522 | 1282.47876 | 77.44730 | 91.9815 |

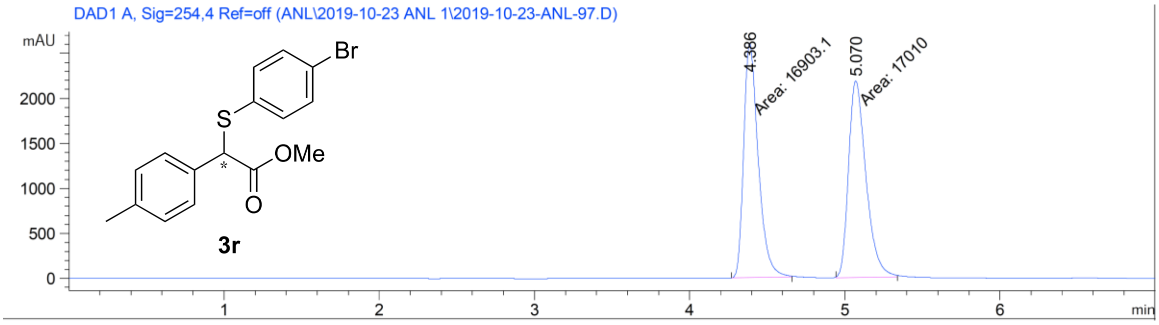


| Peak # | RetTime [min] | Type | Width [min] | Area [mAU*s] | Height [mAU] | Area % |
|---|---|---|---|---|---|---|
| 1 | 4.386 | MM | 0.1084 | 1.69031e4 | 2599.30615 | 49.8424 |
| 2 | 5.070 | MM | 0.1300 | 1.70100e4 | 2181.32837 | 50.1576 |

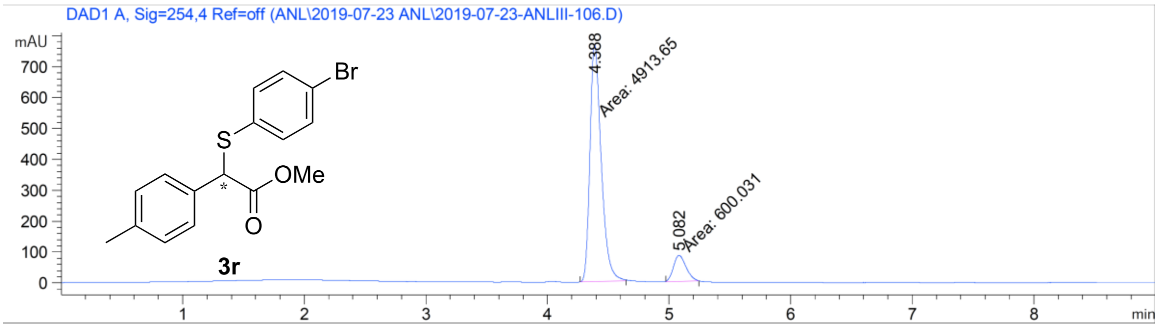


| Peak # | RetTime [min] | Type | Width [min] | Area [mAU*s] | Height [mAU] | Area % |
|---|---|---|---|---|---|---|
| 1 | 4.388 | MM | 0.1068 | 4913.65283 | 766.77258 | 89.1174 |
| 2 | 5.082 | MM | 0.1191 | 600.03131 | 83.95432 | 10.8826 |

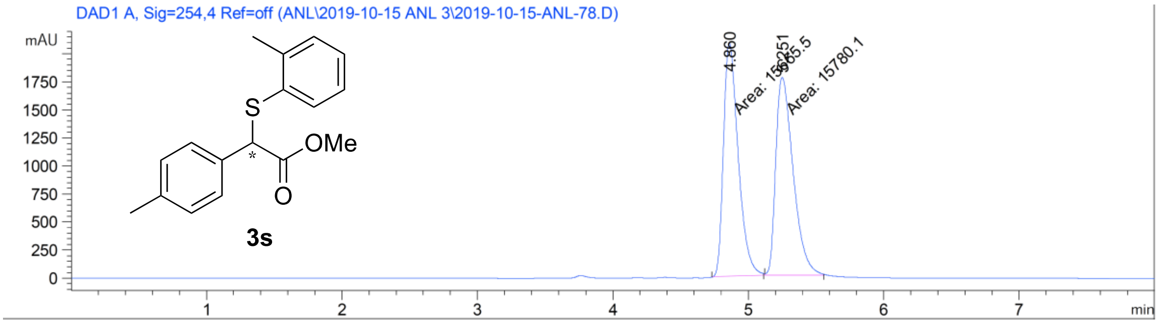


| Peak # | RetTime [min] | Type | Width [min] | Area [mAU*s] | Height [mAU] | Area % |
|---|---|---|---|---|---|---|
| 1 | 4.860 | MM | 0.1256 | 1.56655e4 | 2078.02954 | 49.8179 |
| 2 | 5.251 | MM | 0.1491 | 1.57801e4 | 1764.39966 | 50.1821 |

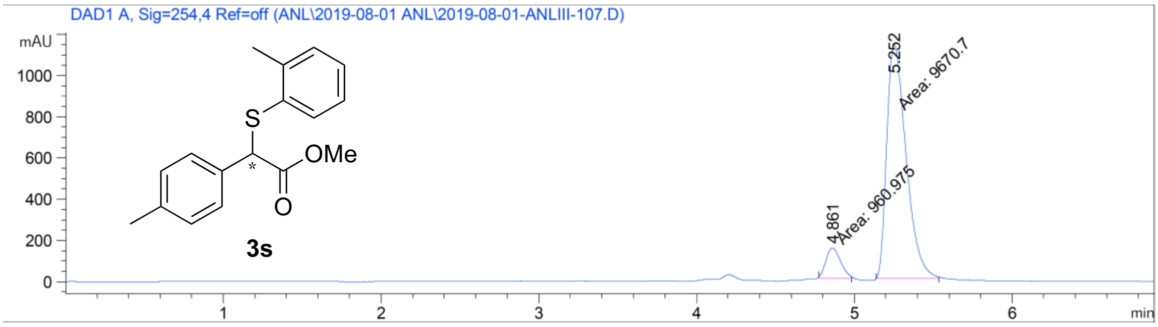


| Peak # | RetTime [min] | Type | Width [min] | Area [mAU*s] | Height [mAU] | Area % |
|---|---|---|---|---|---|---|
| 1 | 4.861 | MM | 0.1080 | 960.97528 | 148.30014 | 9.0388 |
| 2 | 5.252 | MM | 0.1418 | 9670.70215 | 1136.30225 | 90.9612 |

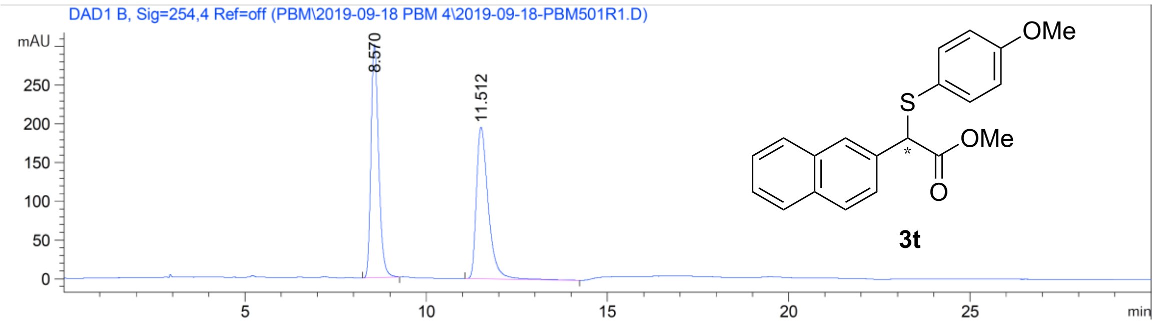


| Peak # | RetTime [min] | Type | Width [min] | Area [mAU*s] | Height [mAU] | Area % |
|---|---|---|---|---|---|---|
| 1 | 8.570 | BB | 0.2253 | 4402.89307 | 301.61163 | 49.5735 |
| 2 | 11.512 | BB | 0.3478 | 4478.65283 | 195.83733 | 50.4265 |

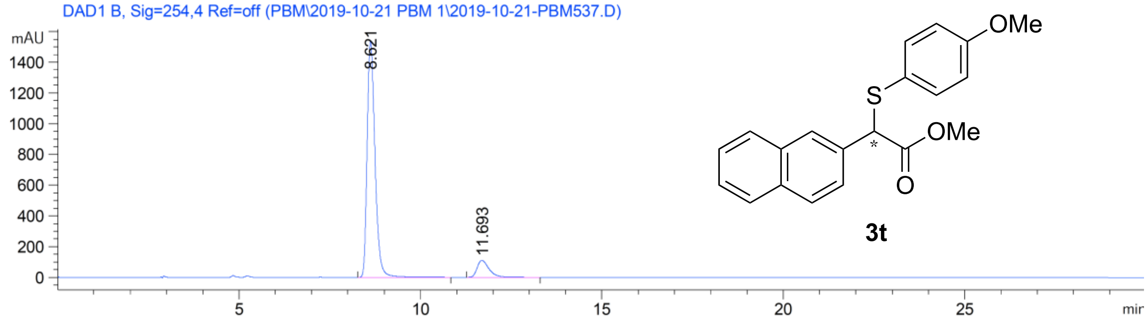


| Peak # | RetTime [min] | Type | Width [min] | Area [mAU*s] | Height [mAU] | Area % |
|---|---|---|---|---|---|---|
| 1 | 8.621 | BB | 0.2313 | 2.31060e4 | 1537.99048 | 90.2114 |
| 2 | 11.693 | BB | 0.3486 | 2507.17847 | 109.73164 | 9.7886 |

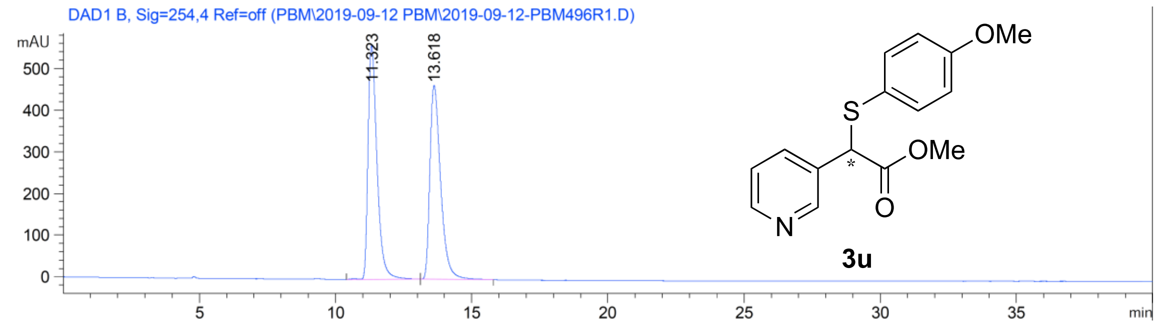


| Peak # | RetTime [min] | Type | Width [min] | Area [mAU*s] | Height [mAU] | Area % |
|---|---|---|---|---|---|---|
| 1 | 11.323 | VB R | 0.3462 | 1.28756e4 | 562.74652 | 50.1114 |
| 2 | 13.618 | BB | 0.4160 | 1.28184e4 | 465.12823 | 49.8886 |

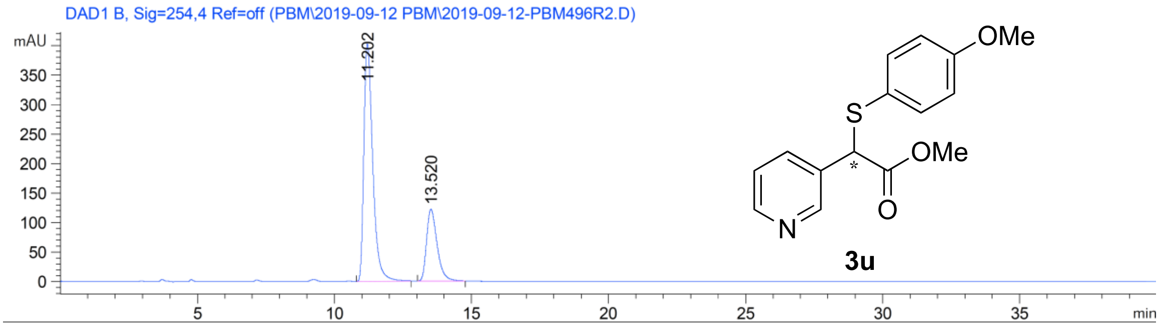


| Peak # | RetTime [min] | Type | Width [min] | Area [mAU*s] | Height [mAU] | Area % |
|---|---|---|---|---|---|---|
| 1 | 11.202 | BB | 0.3400 | 9034.04492 | 402.33542 | 73.5713 |
| 2 | 13.520 | BB | 0.3842 | 3245.25586 | 121.47859 | 26.4287 |

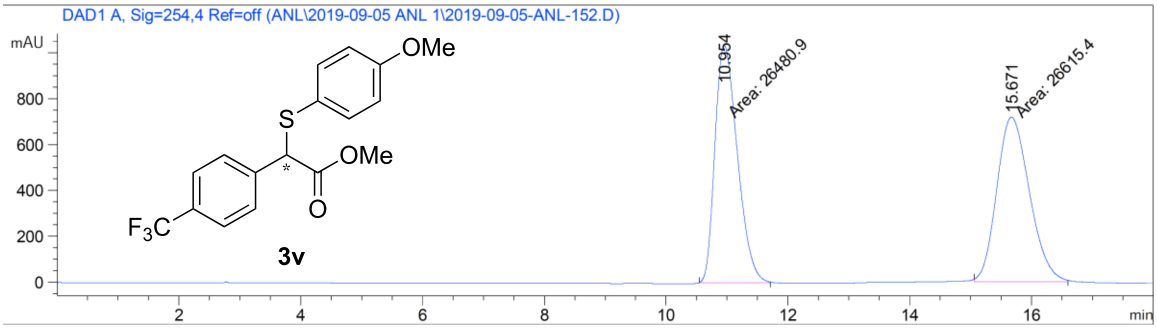


| Peak # | RetTime [min] | Type | Width [min] | Area [mAU*s] | Height [mAU] | Area % |
|---|---|---|---|---|---|---|
| 1 | 10.954 | MM | 0.4275 | 2.64809e4 | 1032.28027 | 49.8733 |
| 2 | 15.671 | MM | 0.6211 | 2.66154e4 | 714.20123 | 50.1267 |

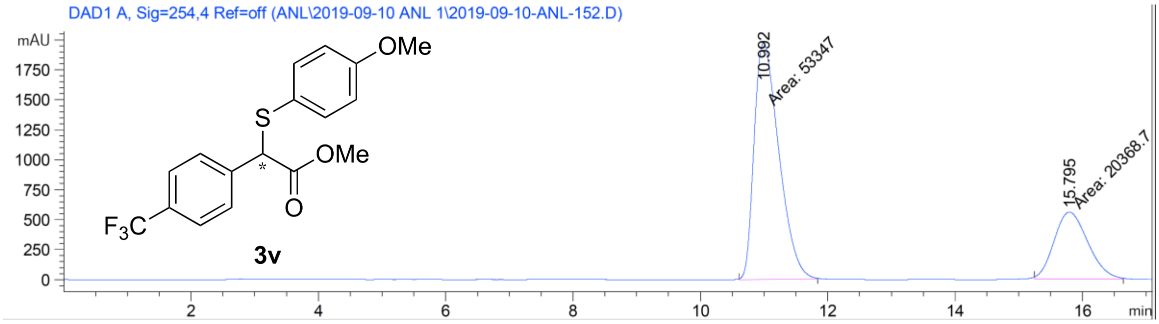


| Peak # | RetTime [min] | Type | Width [min] | Area [mAU*s] | Height [mAU] | Area % |
|---|---|---|---|---|---|---|
| 1 | 10.992 | MM | 0.4509 | 5.33470e4 | 1971.76733 | 72.3685 |
| 2 | 15.795 | MM | 0.6108 | 2.03687e4 | 555.77057 | 27.6315 |

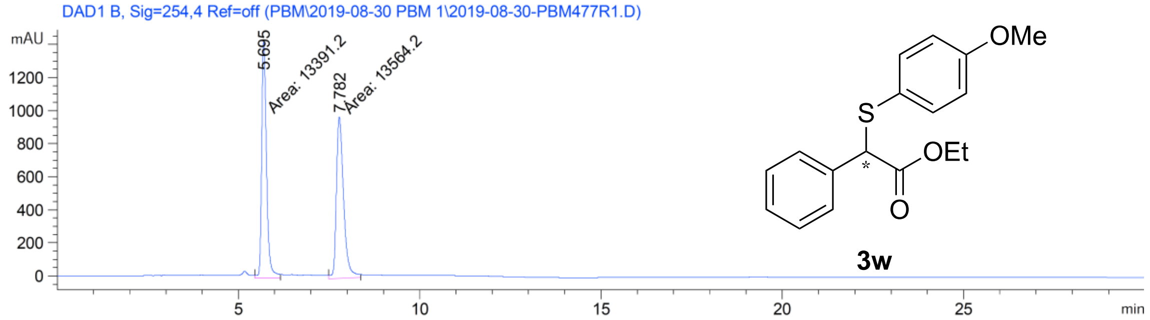


| Peak # | RetTime [min] | Type | Width [min] | Area [mAU*s] | Height [mAU] | Area % |
|---|---|---|---|---|---|---|
| 1 | 5.695 | MM | 0.1559 | 1.33912e4 | 1431.46667 | 49.6791 |
| 2 | 7.782 | MM | 0.2317 | 1.35642e4 | 975.90668 | 50.3209 |

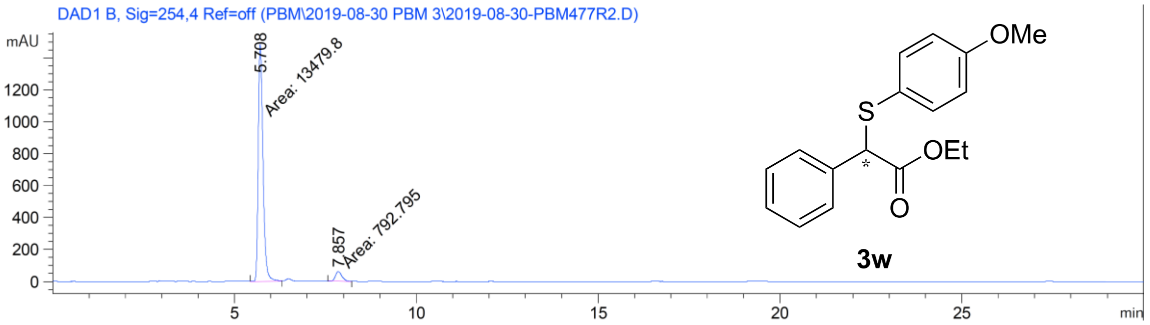


| Peak # | RetTime [min] | Type | Width [min] | Area [mAU*s] | Height [mAU] | Area % |
|---|---|---|---|---|---|---|
| 1 | 5.708 | MM | 0.1510 | 1.34798e4 | 1488.18005 | 94.4453 |
| 2 | 7.857 | MM | 0.2170 | 792.79486 | 60.90101 | 5.5547 |

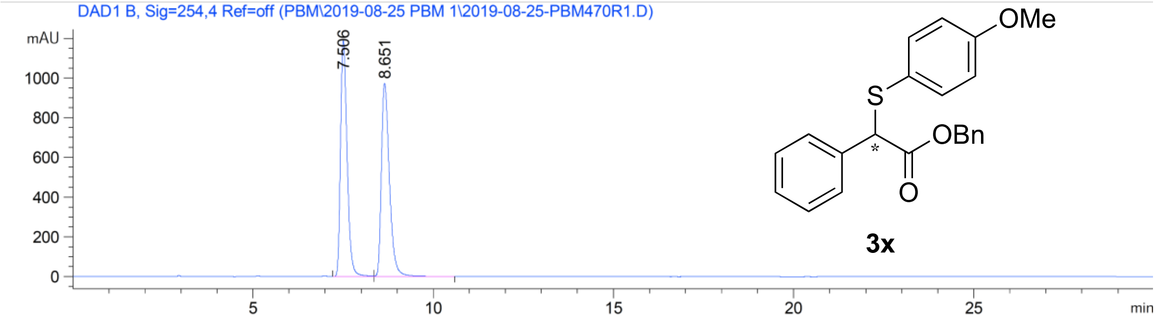


| Peak # | RetTime [min] | Type | Width [min] | Area [mAU*s] | Height [mAU] | Area % |
|---|---|---|---|---|---|---|
| 1 | 7.506 | BV | 0.1950 | 1.50764e4 | 1188.24866 | 49.7699 |
| 2 | 8.651 | VB | 0.2393 | 1.52158e4 | 973.93750 | 50.2301 |

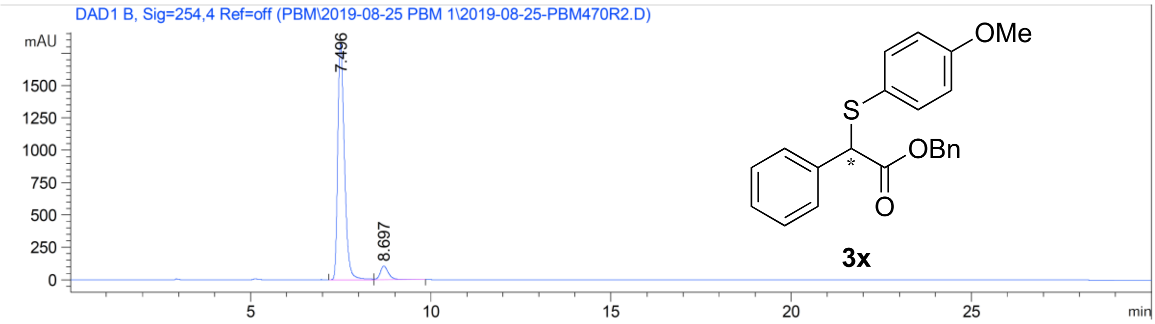


| Peak # | RetTime [min] | Type | Width [min] | Area [mAU*s] | Height [mAU] | Area % |
|---|---|---|---|---|---|---|
| 1 | 7.496 | BV | 0.1981 | 2.34048e4 | 1819.50891 | 93.3941 |
| 2 | 8.697 | VB | 0.2408 | 1655.44507 | 104.51083 | 6.6059 |

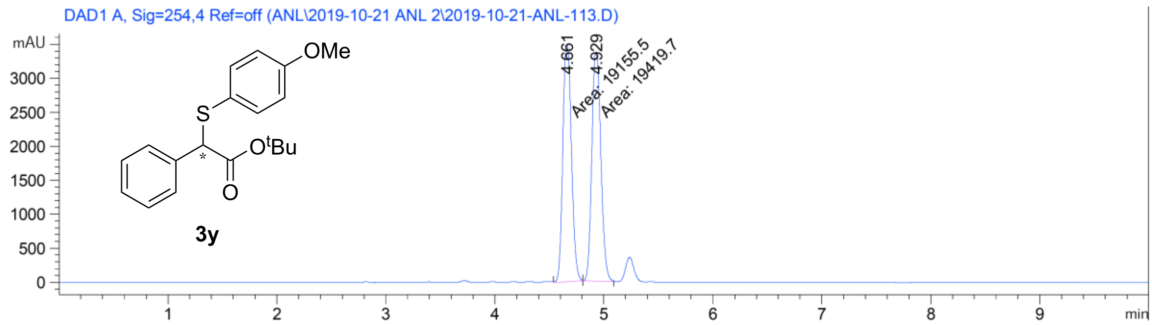


| Peak # | RetTime [min] | Type | Width [min] | Area [mAU*s] | Height [mAU] | Area % |
|---|---|---|---|---|---|---|
| 1 | 4.661 | MM | 0.0919 | 1.91555e4 | 3475.36865 | 49.6575 |
| 2 | 4.929 | MM | 0.0962 | 1.94197e4 | 3364.91992 | 50.3425 |

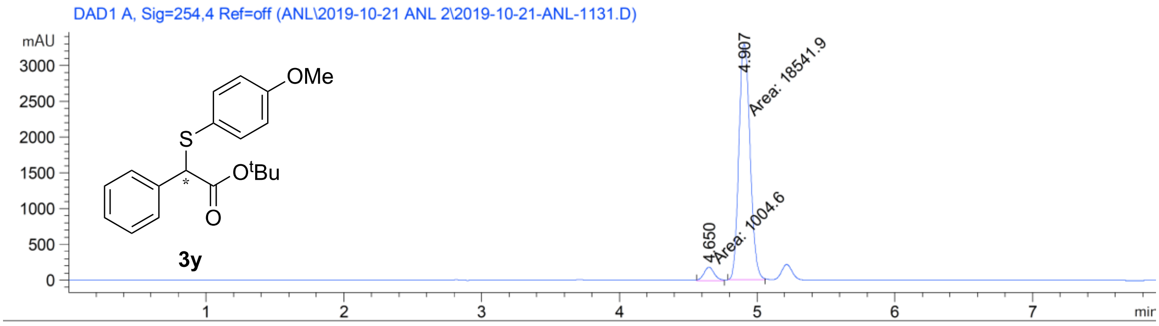


| Peak # | RetTime [min] | Type | Width [min] | Area [mAU*s] | Height [mAU] | Area % |
|---|---|---|---|---|---|---|
| 1 | 4.650 | MM | 0.0880 | 1004.59747 | 190.32745 | 5.1395 |
| 2 | 4.907 | MM | 0.0938 | 1.85419e4 | 3295.51489 | 94.8605 |

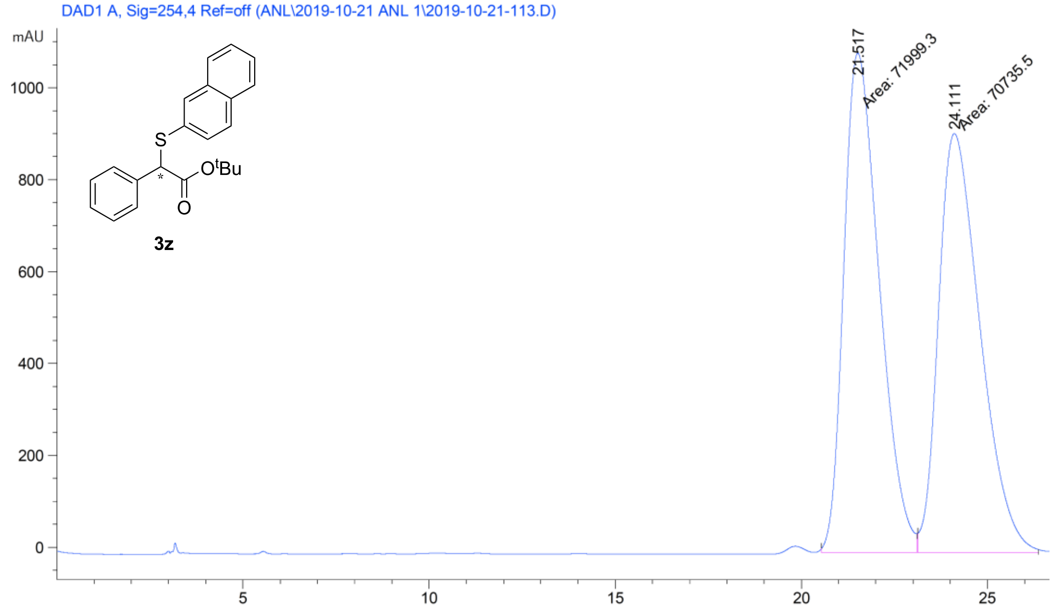


| Peak # | RetTime [min] | Type | Width [min] | Area [mAU*s] | Height [mAU] | Area % |
|---|---|---|---|---|---|---|
| 1 | 21.517 | MM | 1.1043 | 7.19993e4 | 1086.63892 | 50.4427 |
| 2 | 24.111 | MM | 1.2930 | 7.07355e4 | 911.75879 | 49.5573 |

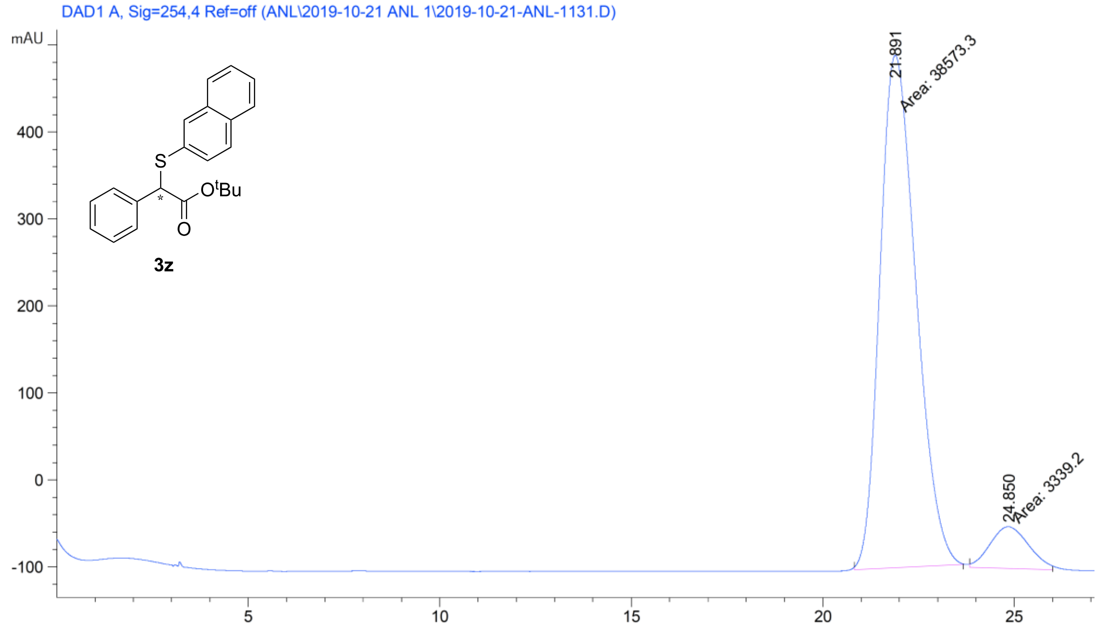


| Peak # | RetTime [min] | Type | Width [min] | Area [mAU*s] | Height [mAU] | Area % |
|---|---|---|---|---|---|---|
| 1 | 21.891 | MM | 1.0914 | 3.85733e4 | 589.03247 | 92.0329 |
| 2 | 24.850 | MM | 1.1662 | 3339.20020 | 47.72198 | 7.9671 |

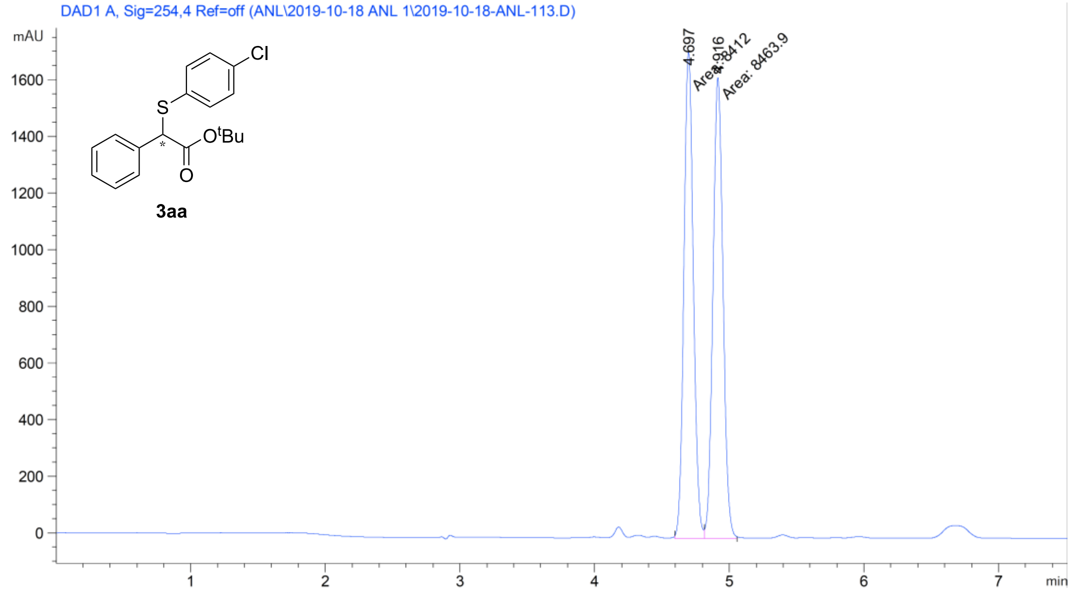


| Peak # | RetTime [min] | Type | Width [min] | Area [mAU*s] | Height [mAU] | Area % |
|---|---|---|---|---|---|---|
| 1 | 4.697 | MM | 0.0816 | 8411.99902 | 1718.07410 | 49.8462 |
| 2 | 4.916 | MM | 0.0866 | 8463.89746 | 1628.05530 | 50.1538 |

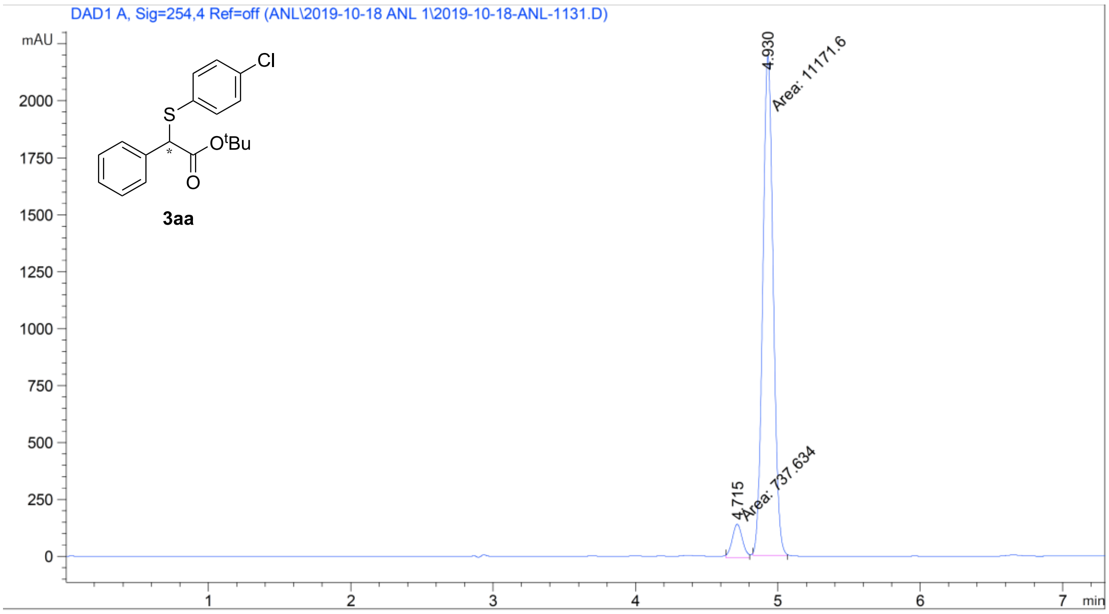


| Peak # | RetTime [min] | Type | Width [min] | Area [mAU*s] | Height [mAU] | Area % |
|---|---|---|---|---|---|---|
| 1 | 4.715 | MM | 0.0835 | 737.63434 | 147.25058 | 6.1938 |
| 2 | 4.930 | MM | 0.0849 | 1.11716e4 | 2192.22339 | 93.8062 |

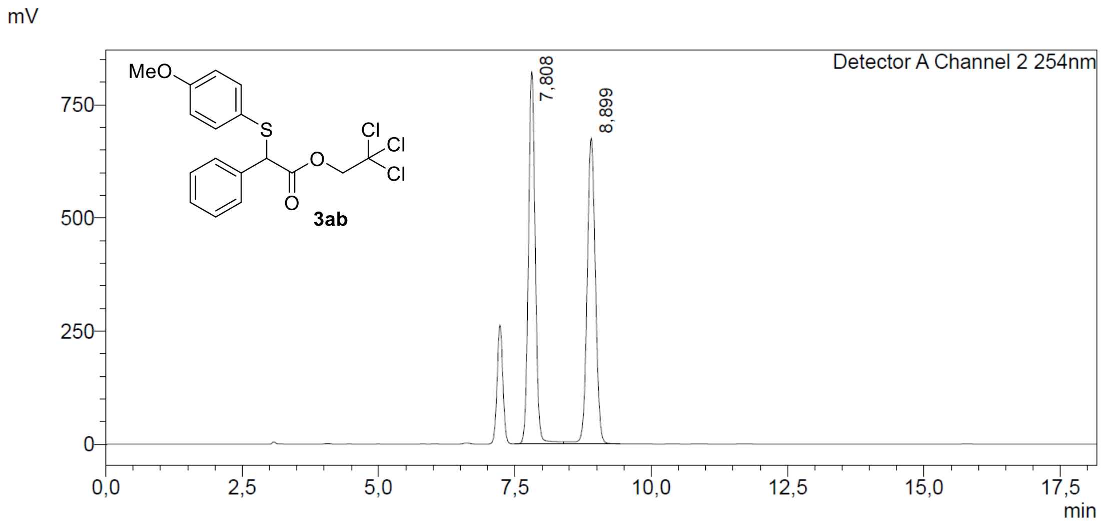


Detector A Channel 2 254nm

| Peak# | Ret. Time | Area | Area% |
|---|---|---|---|
| 1 | 7,808 | 7127103 | 50,101 |
| 2 | 8,899 | 7098342 | 49,899 |
| Total | | 14225444 | 100,000 |

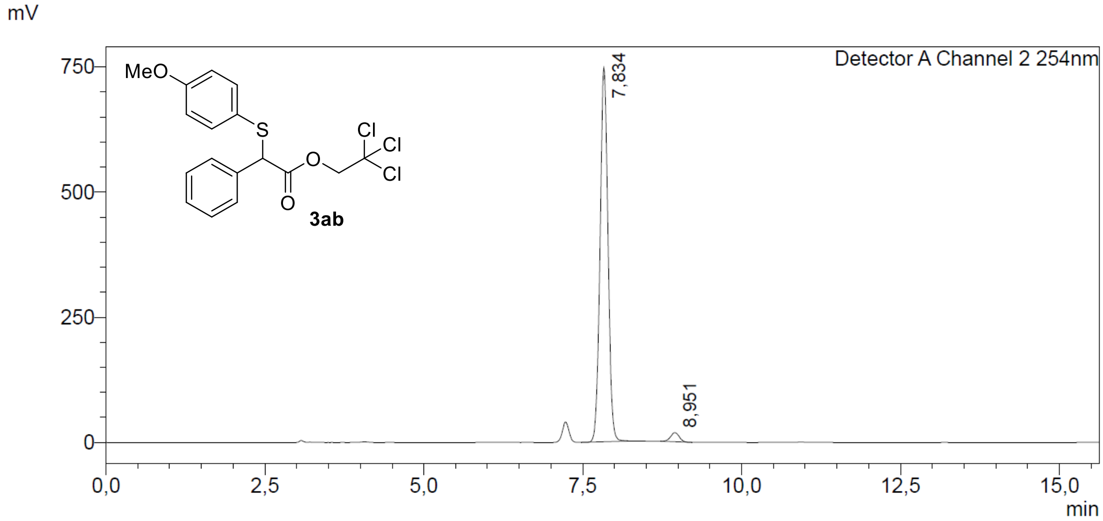


Detector A Channel 2 254nm

| Peak# | Ret. Time | Area | Area% |
|---|---|---|---|
| 1 | 7,834 | 6466196 | 97,388 |
| 2 | 8,951 | 173412 | 2,612 |
| Total | | 6639608 | 100,000 |

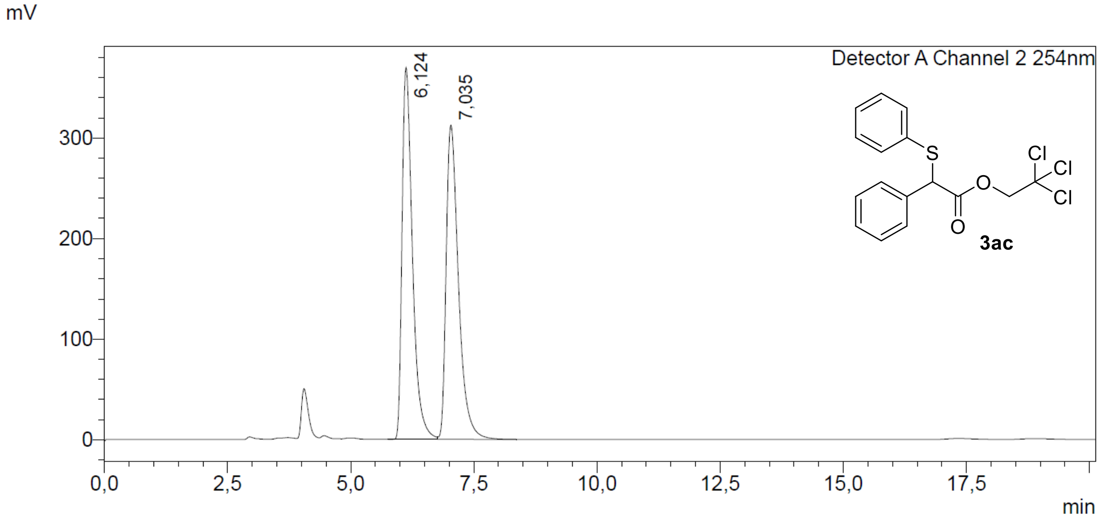


Detector A Channel 2 254nm

| Peak# | Ret. Time | Area | Area% |
|---|---|---|---|
| 1 | 6,124 | 5420746 | 49,922 |
| 2 | 7,035 | 5437605 | 50,078 |
| Total | | 10858351 | 100,000 |

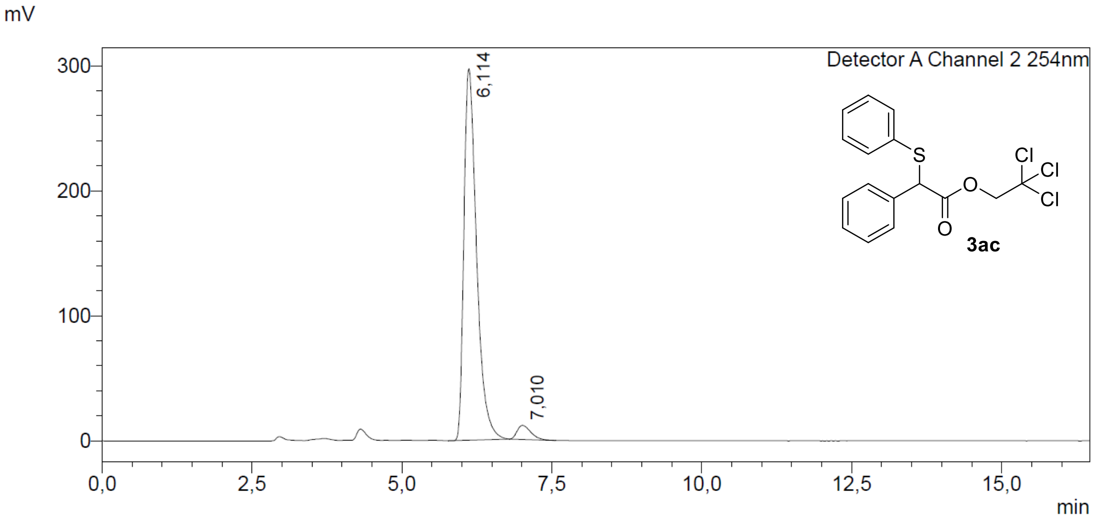


Detector A Channel 2 254nm

| Peak# | Ret. Time | Area | Area% |
|---|---|---|---|
| 1 | 6,114 | 4352225 | 96,071 |
| 2 | 7,010 | 178007 | 3,929 |
| Total | | 4530232 | 100,000 |

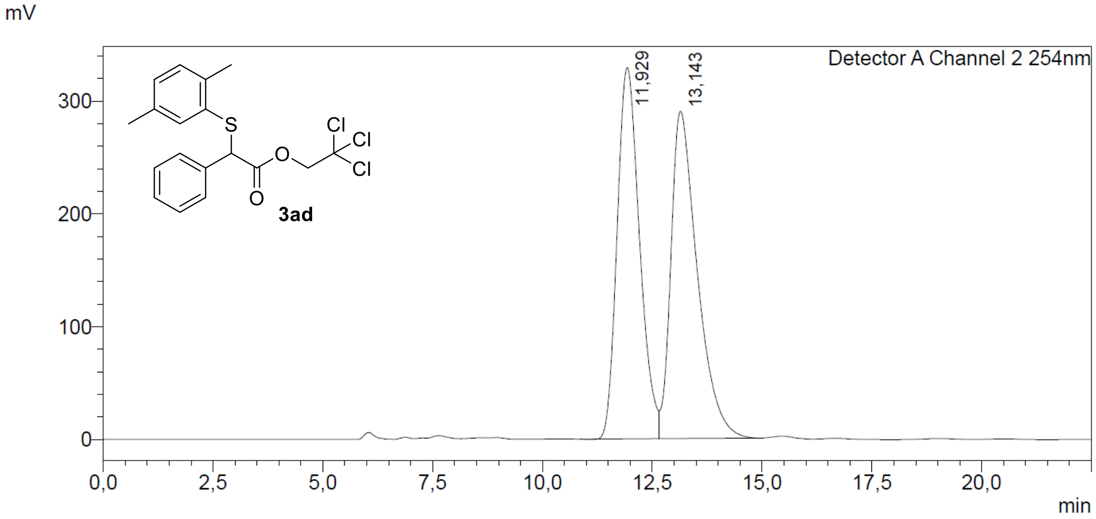


Detector A Channel 2 254nm

| Peak# | Ret. Time | Area | Area% |
|---|---|---|---|
| 1 | 11,929 | 11695000 | 49,053 |
| 2 | 13,143 | 12146786 | 50,947 |
| Total | | 23841786 | 100,000 |

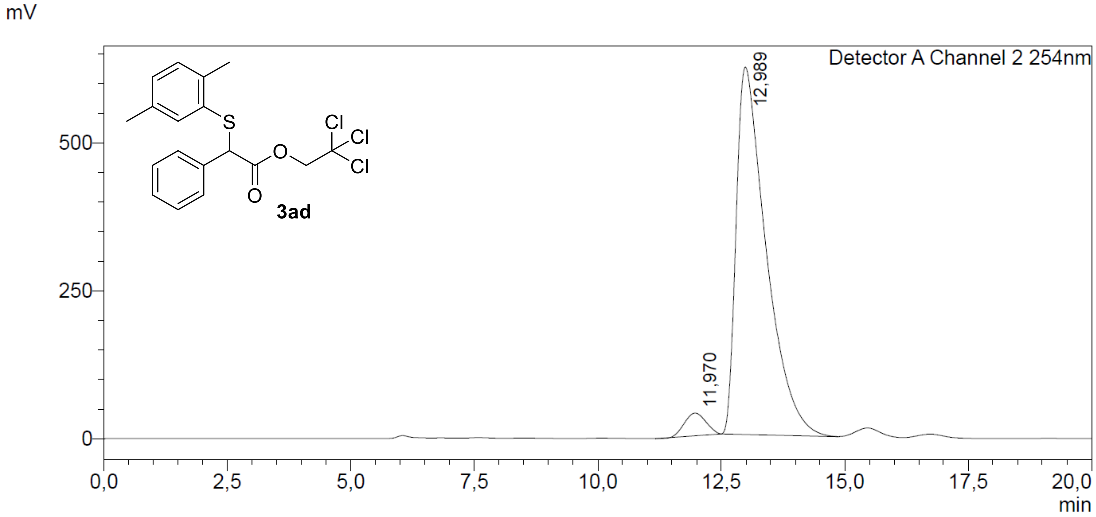


Detector A Channel 2 254nm

| Peak# | Ret. Time | Area | Area% |
|---|---|---|---|
| 1 | 11,970 | 1158349 | 4,236 |
| 2 | 12,989 | 26187733 | 95,764 |
| Total | | 27346082 | 100,000 |

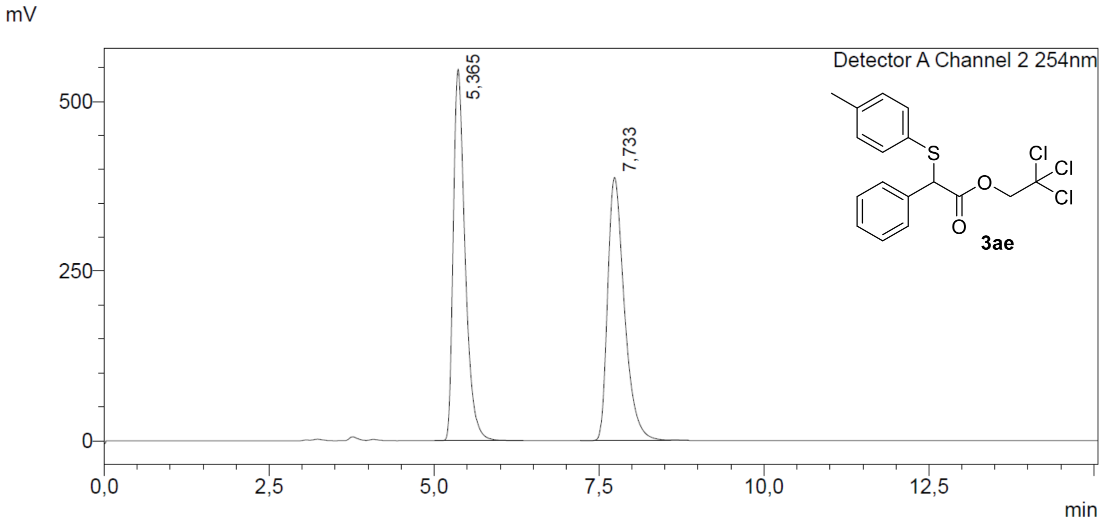


Detector A Channel 2 254nm

| Peak# | Ret. Time | Area | Area% |
|---|---|---|---|
| 1 | 5,365 | 6825129 | 49,909 |
| 2 | 7,733 | 6850139 | 50,091 |
| Total | | 13675268 | 100,000 |

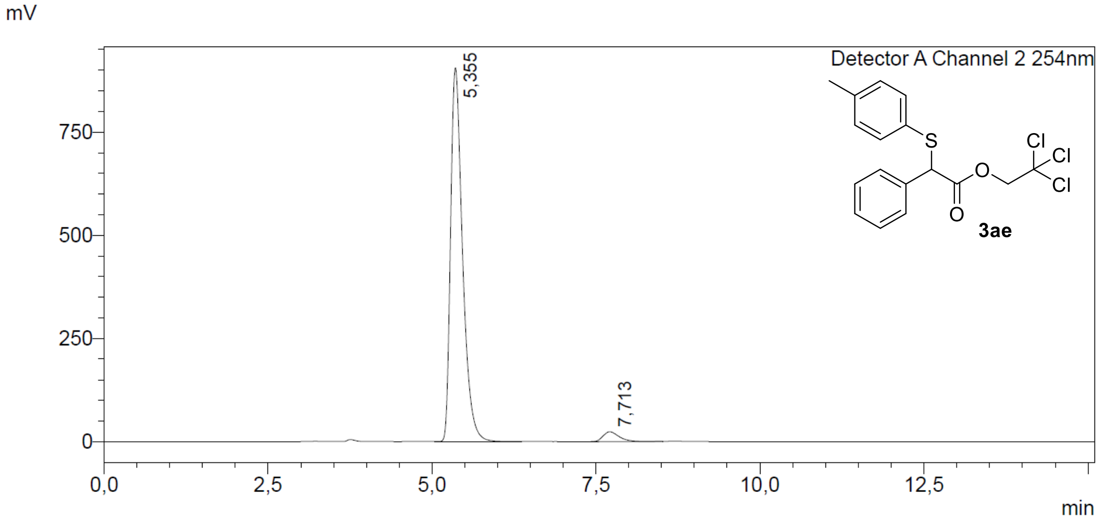


Detector A Channel 2 254nm

| Peak# | Ret. Time | Area | Area% |
|---|---|---|---|
| 1 | 5,355 | 11477241 | 96,601 |
| 2 | 7,713 | 403807 | 3,399 |
| Total | | 11881048 | 100,000 |

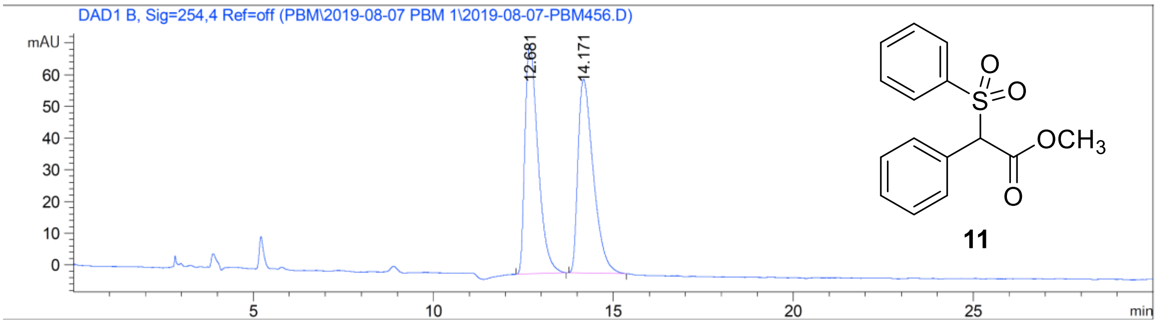


| Peak # | RetTime [min] | Type | Width [min] | Area [mAU*s] | Height [mAU] | Area % |
|---|---|---|---|---|---|---|
| 1 | 12.681 | BB | 0.3465 | 1825.39124 | 72.19901 | 50.2229 |
| 2 | 14.171 | BB | 0.3873 | 1809.18835 | 61.04647 | 49.7771 |

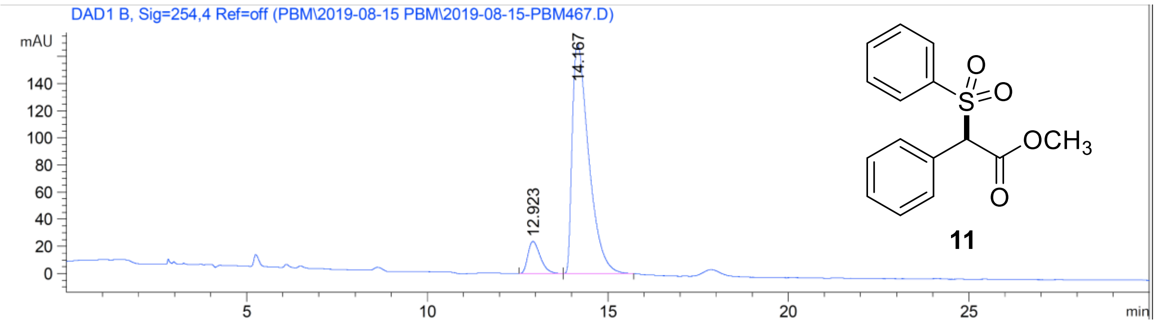


| Peak # | RetTime [min] | Type | Width [min] | Area [mAU*s] | Height [mAU] | Area % |
|---|---|---|---|---|---|---|
| 1 | 12.923 | BB | 0.2865 | 571.80792 | 23.82357 | 9.6060 |
| 2 | 14.167 | BB | 0.4398 | 5380.79590 | 169.27803 | 90.3940 |

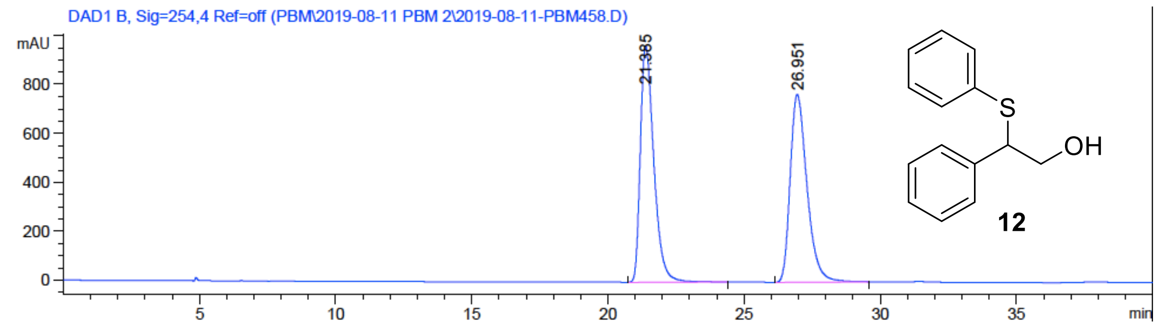


| Peak # | RetTime [min] | Type | Width [min] | Area [mAU*s] | Height [mAU] | Area % |
|---|---|---|---|---|---|---|
| 1 | 21.385 | BB | 0.5273 | 3.31988e4 | 965.19373 | 50.1863 |
| 2 | 26.951 | BB | 0.6587 | 3.29522e4 | 767.82159 | 49.8137 |

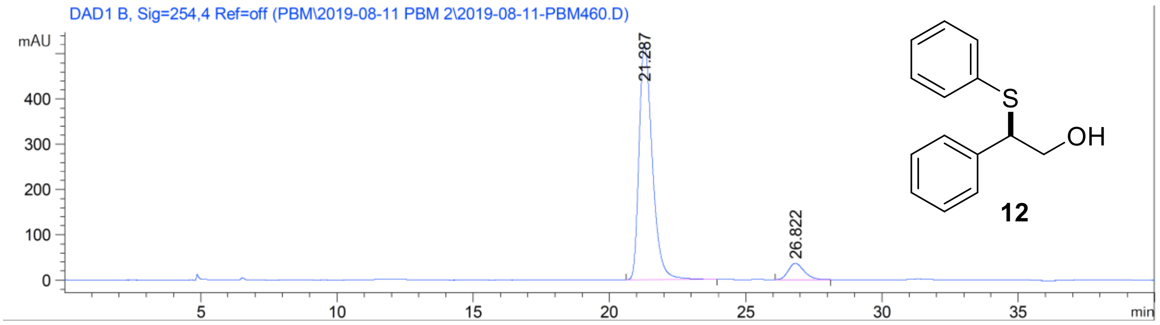


| Peak # | RetTime [min] | Type | Width [min] | Area [mAU*s] | Height [mAU] | Area % |
|---|---|---|---|---|---|---|
| 1 | 21.287 | BB | 0.5126 | 1.74892e4 | 521.08350 | 92.3553 |
| 2 | 26.822 | BB | 0.5764 | 1447.67236 | 35.98093 | 7.6447 |

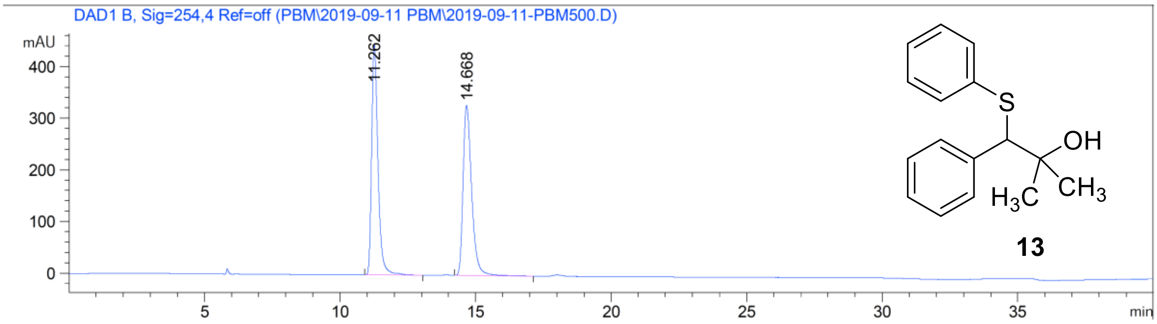


| Peak # | RetTime [min] | Type | Width [min] | Area [mAU*s] | Height [mAU] | Area % |
|---|---|---|---|---|---|---|
| 1 | 11.262 | BB | 0.2469 | 7288.33008 | 445.43866 | 50.2291 |
| 2 | 14.668 | BB | 0.3327 | 7221.83594 | 329.54968 | 49.7709 |

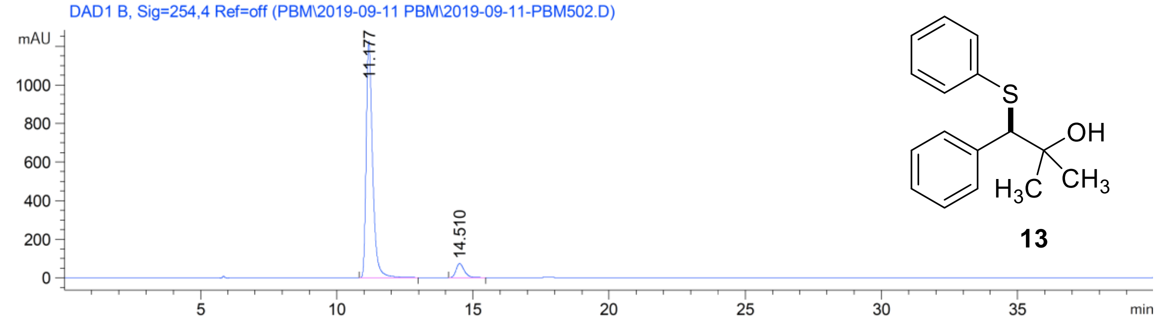


| Peak # | RetTime [min] | Type | Width [min] | Area [mAU*s] | Height [mAU] | Area % |
|---|---|---|---|---|---|---|
| 1 | 11.177 | BB | 0.2477 | 1.99956e4 | 1223.22229 | 92.8370 |
| 2 | 14.510 | BB | 0.3180 | 1542.78491 | 73.46097 | 7.1630 |

## 8. Computational Details

Conformational searches were carried out for each possible binding mode of the transition structure (TS) for the model system (Table 2, entry 10, pyrene truncated to phenyl, trifluoromethyls truncated to fluorines) using the conformational search tool within Schrödinger's MacroModel (version 11.6)[12,13] with the OPLS2005 force field.[14] A Monte Carlo Multiple Minimum (MCMM)[15] / low-mode sampling approach[16] was used to explore the possible conformations of the TS. A total of 157 unique conformation were obtained. These conformations were subsequently optimised by DFT calculations carried out using Gaussian16 (Revision A.03)[17] with the B3LYP density functional[18,19] the split-valence polarised 6-31G(d) basis set.[20]

Single point energy (SPE) calculations were used to correct the Gibbs free energy derived from the original B3LYP calculations.[21] These were performed using the M06-2X[22] density functional and the polarised triple-ζ valence quality (def2-TZVPP) basis set[23]. The integral equation formalism version of the polarisable continuum model (IEF-PCM)[24] (chloroform) was used to incorporate the effect of solvent. All DFT calculations were performed using an ultrafine integration grid. All temperature (245.15 K) and concentration-corrected (1 mol/l) quasiharmonic (Grimme approximation[25]) free energies were calculated with GoodVibes[26] with a vibrational scaling factor of 0.977.[27]

Similar methods have previously been used for the successful modelling of ureas.[28,29]

### Catalyst Truncations

Scheme S1 depicts the lowest energy conformations of catalyst **4b** and the model catalyst (pyrene truncated to phenyl, trifluoromethyls truncated to fluorines). The orientation of the phenyl/pyrene unit is very similar in both structures, and superimposition of these structures over the core atoms highlighted on the model catalyst revealed an RMSD value of 0.095. This indicates that the truncations do not have a significant impact on the conformation of the catalyst, and hence allow for a reasonable approximation of the full transition structures.

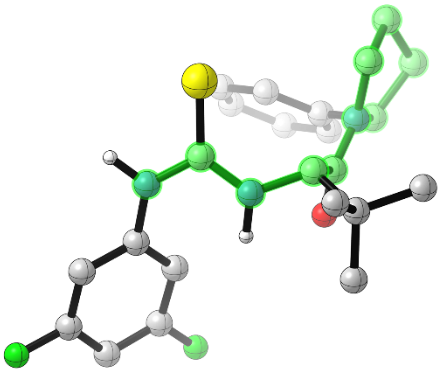

**Model Catalyst**

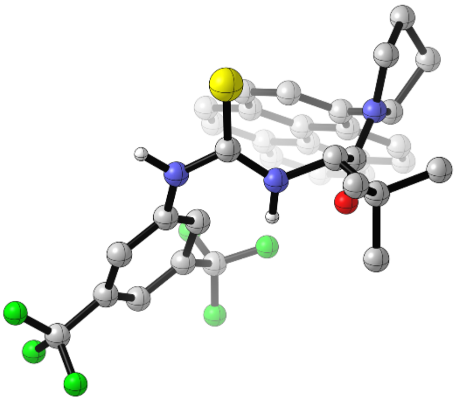

**4b**

**Scheme S1.** Molecular geometries for catalyst **4b** and the model catalyst (B3LYP/6-31G(d)). Highlighted atoms on model catalyst indicate core atoms over which RMSD was calculated for both structures.

### Natural Bond Orbital (NBO) Analysis

NBO analyses were performed using Gaussian16 (Revision A.03)[17] with the M06-2X[22] density functional, the def2-TZVPP basis set[23], IEF-PCM[24] (chloroform) solvent model, and an ultrafine integration grid.

A full list of approximated bond strengths is given below:

- **TS-1** combined ylide S=O···H-N thiourea hydrogen bonds: 12.9 kcal mol$^{-1}$
- **TS-2** combined ylide C=O···H-N thiourea hydrogen bonds: 10.9 kcal mol$^{-1}$
- **TS-1** ylide C=O···H-C 3,5-bisfluorophenyl non-classical hydrogen bond: 2.7 kcal mol$^{-1}$
- **TS-2** ylide S=O···H-C 3,5-bisfluorophenyl non-classical hydrogen bond: 2.0 kcal mol$^{-1}$
- **TS-1** combined ylide C-H···S thiophenol non-classical hydrogen bonds: 5.3 kcal mol$^{-1}$
- **TS-1'** ylide C-H···S thiophenol non-classical hydrogen bond: 1.6 kcal mol$^{-1}$

**Alternative TS Conformations**

Scheme S2 depicts **TS-1'** and **TS-2'**, representing the same binding modes as **TS-1** and **TS-2**, respectively, but with the opposite approach of the thiophenol. These approaches allow for the formation of only one C-H···S interaction between the ylide and thiophenol, accounting for their 3.8 kcal $mol^{-1}$ and 3.4 kcal $mol^{-1}$ higher energy compared to **TS-1** and **TS-2**, respectively. NBO analyses of these C-H···S interactions in **TS-1** revealed a combined strength of 5.3 kcal $mol^{-1}$ for the three interactions, compared to 1.6 kcal $mol^{-1}$ for the single interaction in **TS-1'.** This 3.7 kcal $mol^{-1}$ difference corresponds extremely well with the observed free energy difference of 3.8 kcal $mol^{-1}$ between **TS-1** and **TS-1'**.

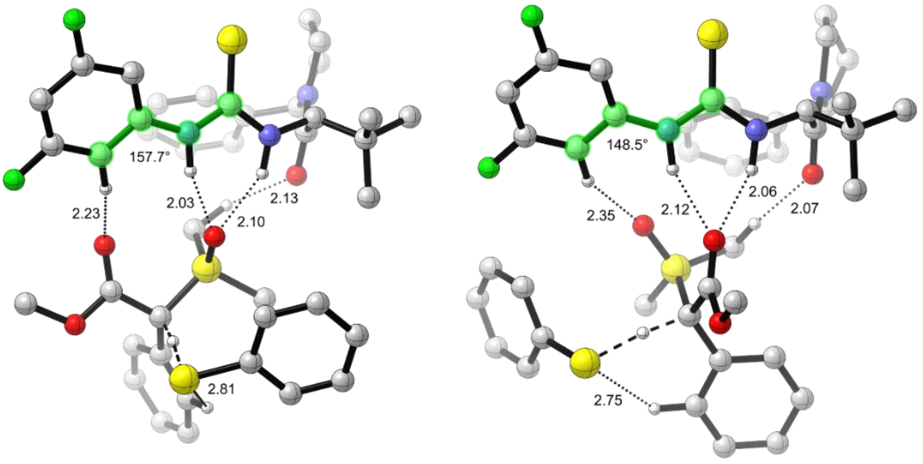


**TS-1'** 3.8 kcal $mol^{-1}$ **TS-2'** 4.5 kcal $mol^{-1}$

**Scheme S2.** Relative free energies of **TS-1'** and **TS-2'** (M06-2X/def2tzvpp/IEFPCM(chloroform)//B3LYP/6-31G(d)). Distances in angstrom (Å). Highlighted atoms indicate measured dihedral angle.

**Energies and molecular geometries of computed structures**

All energies in Hartrees, coordinates in Å. Cartesian coordinates generated by ESIgen software.[30] Additional energies and molecular geometries for catalyst **4b** and model catalyst available on request.

Model Catalyst

B3LYP/6-31G(d) Energy = -1730.087551
M06-2X/def2tzvpp/IEFPCM(chloroform) Energy = -1730.086391
M06-2X/def2tzvpp/IEFPCM(chloroform)//B3LYP/6-31G(d) Quasiharmonic Free Energy = -1729.66654

Frequencies (Top 3 out of 165)

1. 11.4650 cm-1
2. 17.4872 cm-1
3. 26.1134 cm-1

B3LYP/6-31G(d) Molecular Geometry in Cartesian Coordinates

| | | | |
|---|---|---|---|
| C | 1.30621 | -0.83558 | -0.68016 |
| C | 0.57513 | -2.04374 | -0.05285 |
| H | 1.07662 | -2.37738 | 0.85478 |
| C | -1.00911 | -1.06183 | 1.59925 |
| N | -2.15801 | -0.31315 | 1.71920 |
| H | -2.42012 | -0.15976 | 2.68436 |
| N | -0.73558 | -1.54839 | 0.36595 |
| H | -1.31161 | -1.21816 | -0.39899 |
| C | 3.25588 | 0.64164 | -0.80991 |

| | | | |
|---|---|---|---|
| C | 3.35368 | -1.23366 | 0.78062 |
| C | 4.71971 | 0.37361 | -0.38660 |
| H | 3.14190 | 0.61574 | -1.89759 |
| C | 4.57362 | -0.32000 | 0.97547 |
| H | 2.78120 | -1.37420 | 1.70228 |
| H | 3.65986 | -2.21838 | 0.40625 |
| H | 5.31037 | 1.29217 | -0.34451 |
| H | 5.19142 | -0.30389 | -1.10926 |
| H | 4.36768 | 0.42096 | 1.75516 |
| H | 5.46351 | -0.88237 | 1.27259 |
| N | 2.55112 | -0.52369 | -0.23842 |
| O | 0.72999 | -0.15476 | -1.53572 |
| S | -0.08766 | -1.35914 | 2.98542 |
| C | 0.44039 | -3.27366 | -1.01359 |
| C | -0.17864 | -4.43846 | -0.21595 |
| H | -0.29548 | -5.31818 | -0.85979 |
| H | 0.45377 | -4.72281 | 0.63331 |
| H | -1.16534 | -4.17238 | 0.17631 |
| C | -0.44222 | -2.96723 | -2.24084 |
| H | -0.45656 | -3.83682 | -2.90830 |
| H | -1.48284 | -2.77180 | -1.95440 |
| H | -0.07458 | -2.10344 | -2.80014 |
| C | 1.84493 | -3.68541 | -1.49690 |
| H | 2.50384 | -3.93189 | -0.65564 |
| H | 1.77552 | -4.57723 | -2.12985 |
| H | 2.32201 | -2.89691 | -2.08990 |
| C | -3.00808 | 0.24122 | 0.73597 |
| C | -4.38165 | 0.28674 | 1.02150 |
| C | -2.51679 | 0.79224 | -0.45993 |
| C | -5.23717 | 0.88006 | 0.10400 |
| H | -4.78283 | -0.14391 | 1.93240 |
| C | -3.43110 | 1.35028 | -1.34800 |
| H | -1.45901 | 0.83567 | -0.69917 |
| C | -4.79713 | 1.41528 | -1.10089 |
| H | -5.48225 | 1.86566 | -1.80827 |
| C | 2.73036 | 1.98423 | -0.30970 |
| C | 3.00381 | 3.13340 | -1.06363 |
| C | 2.03017 | 2.12238 | 0.89375 |
| C | 2.59766 | 4.39219 | -0.62340 |
| H | 3.53437 | 3.03952 | -2.00931 |
| C | 1.62029 | 3.38263 | 1.33590 |
| H | 1.78406 | 1.24398 | 1.48404 |
| C | 1.90445 | 4.52132 | 0.58225 |
| H | 2.81500 | 5.27078 | -1.22537 |
| H | 1.07394 | 3.46925 | 2.27150 |
| H | 1.58254 | 5.50069 | 0.92605 |
| F | -2.96664 | 1.87732 | -2.49352 |
| F | -6.55262 | 0.92158 | 0.38520 |

Catalyst **4b**

B3LYP/6-31G(d) Energy = -2589.213578
M06-2X/def2tzvpp/IEFPCM(chloroform) Energy = -2589.270019
M06-2X/def2tzvpp/IEFPCM(chloroform)//B3LYP/6-31G(d) Quasiharmonic Free Energy = -2588.729743

Frequencies (Top 3 out of 225)

1. 7.5319 cm-1
2. 9.4476 cm-1
3. 13.1780 cm-1

B3LYP/6-31G(d) Molecular Geometry in Cartesian Coordinates

| | | | |
|---|---|---|---|
| C | 0.675669 | 2.665418 | -0.607231 |
| C | 2.140074 | 3.040467 | -0.302439 |
| H | 2.218527 | 3.660946 | 0.590132 |
| C | 3.248862 | 1.419621 | 1.247537 |
| N | 3.528516 | 0.079887 | 1.426814 |
| H | 4.065412 | -0.094388 | 2.266744 |
| N | 2.786261 | 1.768678 | 0.025505 |
| H | 2.580157 | 1.033168 | -0.642382 |
| C | -1.726247 | 2.946850 | -0.239925 |
| C | -0.205470 | 4.441982 | 0.992545 |
| C | -2.501438 | 4.172612 | 0.302270 |
| H | -1.827958 | 2.849108 | -1.323081 |
| C | -1.645877 | 4.638234 | 1.489970 |
| H | 0.490386 | 4.204752 | 1.803188 |
| H | 0.158513 | 5.340812 | 0.480030 |
| H | -3.527252 | 3.922272 | 0.582685 |
| H | -2.538178 | 4.953293 | -0.467864 |
| H | -1.833393 | 4.000924 | 2.360705 |
| H | -1.840587 | 5.673680 | 1.783324 |
| N | -0.323286 | 3.318600 | 0.040105 |
| O | 0.443880 | 1.735396 | -1.387269 |
| S | 3.557529 | 2.492033 | 2.513408 |
| C | -2.145865 | 1.628661 | 0.409702 |
| C | -1.428394 | 1.100415 | 1.486507 |
| C | -3.292183 | 0.938831 | -0.058086 |
| C | -1.799016 | -0.091663 | 2.102028 |
| H | -0.547029 | 1.624308 | 1.844332 |
| C | -3.679021 | -0.288663 | 0.570486 |
| C | -4.104204 | 1.415626 | -1.148401 |
| C | -2.917553 | -0.809643 | 1.660860 |
| H | -1.214509 | -0.474878 | 2.934978 |
| C | -4.829016 | -1.004111 | 0.114992 |
| C | -5.201406 | 0.733516 | -1.579625 |
| H | -3.839279 | 2.343147 | -1.644030 |
| C | -3.321386 | -2.046377 | 2.270358 |
| C | -5.209681 | -2.231004 | 0.742442 |
| C | -5.607652 | -0.498460 | -0.968997 |
| H | -5.791890 | 1.119108 | -2.407421 |
| C | -4.415471 | -2.725873 | 1.832903 |
| H | -2.726821 | -2.430390 | 3.095707 |
| C | -6.344174 | -2.913648 | 0.276083 |
| C | -6.735391 | -1.215605 | -1.401157 |
| H | -4.709039 | -3.661225 | 2.303144 |
| C | -7.097238 | -2.410434 | -0.783722 |
| H | -6.631568 | -3.847250 | 0.753634 |
| H | -7.325010 | -0.826510 | -2.227709 |
| H | -7.971773 | -2.954405 | -1.130202 |
| C | 2.860677 | 3.787645 | -1.477866 |
| C | 4.266316 | 4.196629 | -0.996342 |
| H | 4.800185 | 4.720399 | -1.798236 |
| H | 4.216989 | 4.862494 | -0.127668 |
| H | 4.859528 | 3.322439 | -0.710313 |
| C | 2.989971 | 2.906101 | -2.735604 |
| H | 3.477518 | 3.477176 | -3.534441 |
| H | 3.612771 | 2.024646 | -2.543482 |
| H | 2.017389 | 2.562673 | -3.096548 |
| C | 2.058800 | 5.056603 | -1.827698 |
| H | 1.964702 | 5.724874 | -0.963013 |
| H | 2.571308 | 5.616503 | -2.618020 |
| H | 1.052802 | 4.819373 | -2.193013 |

| | | | |
|---|---|---|---|
| C | 3.083568 | -1.053169 | 0.708556 |
| C | 3.954023 | -2.146239 | 0.597283 |
| C | 1.787723 | -1.149770 | 0.178113 |
| C | 3.534755 | -3.315090 | -0.034155 |
| H | 4.956958 | -2.078592 | 1.006248 |
| C | 1.389423 | -2.322183 | -0.464826 |
| H | 1.080276 | -0.334630 | 0.275621 |
| C | 2.252873 | -3.411216 | -0.576524 |
| H | 1.930763 | -4.319565 | -1.071087 |
| C | -0.033867 | -2.449060 | -0.956092 |
| C | 4.503871 | -4.458364 | -0.198569 |
| F | -0.548993 | -1.263884 | -1.321172 |
| F | -0.833341 | -2.959146 | 0.009654 |
| F | -0.114835 | -3.284059 | -2.016573 |
| F | 5.208075 | -4.347288 | -1.347221 |
| F | 5.399851 | -4.502613 | 0.811646 |
| F | 3.869445 | -5.648782 | -0.236066 |

TS Conformation 1 (**TS-1**)

B3LYP/6-31G(d) Energy = -3411.901231
M06-2X/def2tzvpp/IEFPCM(chloroform) Energy = -3411.900519
M06-2X/def2tzvpp/IEFPCM(chloroform)//B3LYP/6-31G(d) Quasiharmonic Free Energy = -3411.173257

Frequencies (Top 3 out of 291)

1. -994.6660 cm-1
2. 6.8189 cm-1
3. 10.0747 cm-1

B3LYP/6-31G(d) Molecular Geometry in Cartesian Coordinates

| | | | |
|---|---|---|---|
| C | 3.257255 | -1.363583 | 1.001978 |
| C | 3.784020 | 0.017924 | 1.439809 |
| H | 4.673754 | 0.284561 | 0.871062 |
| C | 2.832807 | 1.816908 | -0.023774 |
| N | 1.628103 | 2.430924 | -0.260256 |
| H | 0.846656 | 2.059556 | 0.275976 |
| N | 2.765405 | 0.990653 | 1.065515 |
| H | 1.839604 | 0.799079 | 1.442651 |
| C | 3.520926 | -3.430535 | -0.289578 |
| C | 5.374852 | -1.809709 | -0.333332 |
| C | 4.811847 | -4.105439 | -0.811020 |
| H | 3.110214 | -3.968359 | 0.569900 |
| C | 5.639831 | -2.930054 | -1.349768 |
| H | 5.406408 | -0.813279 | -0.782809 |
| H | 6.100735 | -1.846125 | 0.488099 |
| H | 4.600057 | -4.868715 | -1.563946 |
| H | 5.334614 | -4.586556 | 0.024814 |
| H | 5.277744 | -2.634318 | -2.340059 |
| H | 6.705765 | -3.158303 | -1.437710 |
| N | 4.017803 | -2.117736 | 0.174760 |
| O | 2.132178 | -1.745289 | 1.368618 |
| S | 4.233283 | 2.026201 | -0.945827 |
| C | 2.427468 | -3.325739 | -1.348134 |
| C | 2.317920 | -2.230712 | -2.214483 |
| C | 1.519355 | -4.384488 | -1.485983 |
| C | 1.324279 | -2.198100 | -3.196486 |
| H | 2.992333 | -1.385003 | -2.111371 |
| C | 0.527446 | -4.354648 | -2.466025 |
| C | 0.426019 | -3.258425 | -3.326137 |

| | | | |
|---|---|---|---|
| H | 1.251693 | -1.337573 | -3.856214 |
| O | -2.337985 | 1.067586 | -0.889635 |
| O | -0.198686 | 0.834574 | 1.529408 |
| C | -3.122887 | 0.935639 | 0.044091 |
| C | -2.885635 | 0.068424 | 1.226467 |
| O | -4.322331 | 1.532640 | 0.085912 |
| C | -4.661835 | 2.312049 | -1.080869 |
| H | -4.800986 | 1.649561 | -1.939167 |
| H | -5.595392 | 2.813325 | -0.825482 |
| H | -3.876579 | 3.036828 | -1.306842 |
| S | -1.143579 | -0.306638 | 1.290260 |
| C | -0.978878 | -1.503810 | 2.621407 |
| H | 0.075269 | -1.791776 | 2.622401 |
| H | -1.266540 | -1.009990 | 3.550218 |
| H | -1.635381 | -2.349531 | 2.398105 |
| C | -0.685143 | -1.267382 | -0.163809 |
| H | -1.427276 | -2.065795 | -0.262162 |
| H | -0.718427 | -0.590416 | -1.014196 |
| H | 0.318089 | -1.652183 | 0.036879 |
| C | -3.427560 | 0.516904 | 2.565936 |
| C | -4.407064 | -0.245648 | 3.219830 |
| C | -2.981236 | 1.707927 | 3.165872 |
| C | -4.927081 | 0.175222 | 4.445096 |
| H | -4.772434 | -1.157224 | 2.757221 |
| C | -3.496198 | 2.119836 | 4.394118 |
| H | -2.227326 | 2.310190 | 2.667218 |
| C | -4.471602 | 1.353955 | 5.036666 |
| H | -5.691018 | -0.421663 | 4.935934 |
| H | -3.140353 | 3.042179 | 4.845150 |
| H | -4.877065 | 1.678014 | 5.991470 |
| C | -3.894636 | -3.349795 | -2.053222 |
| C | -4.276026 | -3.172188 | -3.383855 |
| C | -5.190201 | -2.175968 | -3.731500 |
| C | -5.727335 | -1.364191 | -2.730048 |
| C | -5.357021 | -1.544921 | -1.396460 |
| C | -4.427673 | -2.537411 | -1.038312 |
| H | -3.180423 | -4.124740 | -1.789181 |
| H | -3.855497 | -3.816497 | -4.152756 |
| H | -5.485271 | -2.037850 | -4.768164 |
| H | -6.452161 | -0.594142 | -2.985034 |
| H | -5.793627 | -0.922048 | -0.621252 |
| S | -3.958999 | -2.792510 | 0.668231 |
| H | -3.425911 | -1.239584 | 0.945013 |
| H | 1.584456 | -5.236893 | -0.812545 |
| H | -0.171173 | -5.182726 | -2.551892 |
| H | -0.352727 | -3.226999 | -4.082895 |
| C | 4.156162 | 0.104613 | 2.960746 |
| C | 1.238860 | 3.441806 | -1.161931 |
| C | 2.110161 | 4.392759 | -1.709772 |
| C | -0.137891 | 3.504766 | -1.443310 |
| C | 1.572863 | 5.374797 | -2.532745 |
| H | 3.171788 | 4.372099 | -1.513876 |
| C | -0.607964 | 4.518981 | -2.260362 |
| H | -0.831903 | 2.762691 | -1.063480 |
| C | 0.219094 | 5.478767 | -2.831169 |
| H | -0.166138 | 6.261241 | -3.472976 |
| F | 2.411787 | 6.290338 | -3.059224 |
| F | -1.935757 | 4.574615 | -2.517145 |
| C | 5.214754 | -0.970778 | 3.275500 |
| H | 6.115773 | -0.838689 | 2.664231 |
| H | 5.519560 | -0.902637 | 4.325965 |
| H | 4.831881 | -1.984937 | 3.110474 |

| | | | |
|---|---|---|---|
| C | 2.940262 | -0.099761 | 3.885198 |
| H | 2.505151 | -1.095267 | 3.765321 |
| H | 3.250946 | 0.016315 | 4.930371 |
| H | 2.156451 | 0.642180 | 3.694922 |
| C | 4.761897 | 1.498274 | 3.219558 |
| H | 4.041240 | 2.291618 | 2.998904 |
| H | 5.058848 | 1.591264 | 4.270912 |
| H | 5.649175 | 1.671128 | 2.599803 |

TS Conformation 2

B3LYP/6-31G(d) Energy = -3411.895891
M06-2X/def2tzvpp/IEFPCM(chloroform) Energy = -3411.899706
M06-2X/def2tzvpp/IEFPCM(chloroform)//B3LYP/6-31G(d) Quasiharmonic Free Energy = -3411.172881

Frequencies (Top 3 out of 291)

1. -985.4798 cm-1
2. 6.2248 cm-1
3. 10.7903 cm-1

B3LYP/6-31G(d) Molecular Geometry in Cartesian Coordinates

| | | | |
|---|---|---|---|
| C | -3.474213 | 0.245928 | 0.827265 |
| C | -3.765571 | -1.174357 | 0.295810 |
| H | -4.639816 | -1.154772 | -0.353875 |
| C | -2.619392 | -1.392679 | -1.895717 |
| N | -1.482410 | -1.809224 | -2.550548 |
| H | -1.450525 | -1.478228 | -3.506334 |
| N | -2.646169 | -1.587564 | -0.553309 |
| H | -1.739326 | -1.663972 | -0.089073 |
| C | -4.132383 | 2.579979 | 1.161318 |
| C | -5.728330 | 1.101478 | 0.008322 |
| C | -5.537475 | 3.227237 | 1.125491 |
| H | -3.759434 | 2.497549 | 2.186646 |
| C | -6.211094 | 2.556190 | -0.079685 |
| H | -5.640699 | 0.623521 | -0.970794 |
| H | -6.401109 | 0.505698 | 0.637553 |
| H | -5.484510 | 4.315735 | 1.043911 |
| H | -6.077450 | 2.980040 | 2.047950 |
| H | -5.861491 | 3.012807 | -1.011689 |
| H | -7.302325 | 2.627674 | -0.059301 |
| N | -4.400171 | 1.217789 | 0.650494 |
| O | -2.397162 | 0.483956 | 1.394830 |
| S | -3.845466 | -0.666148 | -2.810047 |
| C | -3.098591 | 3.347911 | 0.343390 |
| C | -2.860233 | 3.084241 | -1.011253 |
| C | -2.390170 | 4.387312 | 0.961528 |
| C | -1.937131 | 3.848914 | -1.729751 |
| H | -3.372716 | 2.263365 | -1.505859 |
| C | -1.469685 | 5.152026 | 0.245469 |
| C | -1.240561 | 4.885100 | -1.106461 |
| H | -1.761549 | 3.627167 | -2.779125 |
| O | 2.503096 | -0.147420 | -1.559528 |
| O | 0.202348 | -1.367982 | 0.562880 |
| C | 3.179678 | -0.347202 | -0.558257 |
| C | 2.726793 | -0.163623 | 0.843287 |
| O | 4.474600 | -0.713553 | -0.606809 |
| C | 5.024712 | -0.844127 | -1.930698 |
| H | 4.978741 | 0.113666 | -2.455315 |
| H | 6.060116 | -1.151232 | -1.779925 |

| | | | |
|---|---|---|---|
| H | 4.475383 | -1.600183 | -2.495252 |
| S | 0.950294 | -0.120368 | 0.899446 |
| C | 0.582462 | 0.390191 | 2.586950 |
| H | -0.503470 | 0.493495 | 2.628690 |
| H | 0.942073 | -0.394887 | 3.253098 |
| H | 1.094168 | 1.337782 | 2.776029 |
| C | 0.380135 | 1.284195 | -0.075965 |
| H | 0.977849 | 2.151748 | 0.217817 |
| H | 0.547555 | 1.023543 | -1.118706 |
| H | -0.676298 | 1.407360 | 0.170379 |
| C | 3.305112 | -1.078365 | 1.901757 |
| C | 4.128442 | -0.558510 | 2.911974 |
| C | 3.046867 | -2.460783 | 1.883295 |
| C | 4.679170 | -1.401074 | 3.879290 |
| H | 4.349245 | 0.504234 | 2.927437 |
| C | 3.590913 | -3.297818 | 2.856812 |
| H | 2.416105 | -2.877243 | 1.103222 |
| C | 4.409423 | -2.769898 | 3.857674 |
| H | 5.320665 | -0.983571 | 4.650859 |
| H | 3.379576 | -4.363564 | 2.829517 |
| H | 4.837364 | -3.423354 | 4.613482 |
| C | 3.299566 | 4.486290 | -0.501750 |
| C | 3.775193 | 4.985271 | -1.715174 |
| C | 4.868615 | 4.388944 | -2.345768 |
| C | 5.487626 | 3.291082 | -1.743899 |
| C | 5.022638 | 2.795526 | -0.524915 |
| C | 3.915296 | 3.382657 | 0.111684 |
| H | 2.444539 | 4.950388 | -0.018116 |
| H | 3.286098 | 5.843391 | -2.170756 |
| H | 5.237092 | 4.777495 | -3.291439 |
| H | 6.348334 | 2.824652 | -2.218250 |
| H | 5.519006 | 1.951760 | -0.054918 |
| S | 3.323473 | 2.776745 | 1.686427 |
| H | 3.050731 | 1.229429 | 1.219365 |
| H | -2.555373 | 4.594818 | 2.017082 |
| H | -0.925085 | 5.948732 | 0.745152 |
| H | -0.519181 | 5.474291 | -1.665861 |
| C | -4.047968 | -2.215183 | 1.436427 |
| C | -0.515183 | -2.782121 | -2.205416 |
| C | -0.824064 | -3.896350 | -1.413327 |
| C | 0.766934 | -2.640521 | -2.753724 |
| C | 0.173280 | -4.830093 | -1.168543 |
| H | -1.813975 | -4.052991 | -1.007047 |
| C | 1.713022 | -3.618838 | -2.488205 |
| H | 1.044370 | -1.765694 | -3.329184 |
| C | 1.457181 | -4.729277 | -1.692168 |
| H | 2.214317 | -5.478262 | -1.496203 |
| F | -0.125801 | -5.900281 | -0.406019 |
| F | 2.948446 | -3.482818 | -3.016876 |
| C | -5.181851 | -1.678788 | 2.333959 |
| H | -6.097335 | -1.490754 | 1.760267 |
| H | -5.426080 | -2.417333 | 3.105708 |
| H | -4.897962 | -0.751923 | 2.844860 |
| C | -2.810348 | -2.494508 | 2.311556 |
| H | -2.455504 | -1.586267 | 2.804267 |
| H | -3.068694 | -3.228831 | 3.083771 |
| H | -1.978225 | -2.909657 | 1.733504 |
| C | -4.522039 | -3.528171 | 0.782283 |
| H | -3.756559 | -3.951514 | 0.125044 |
| H | -4.749235 | -4.273807 | 1.552908 |
| H | -5.428488 | -3.374227 | 0.184704 |

TS Conformation 3

B3LYP/6-31G(d) Energy = -3411.899589
M06-2X/def2tzvpp/IEFPCM(chloroform) Energy = -3411.899448
M06-2X/def2tzvpp/IEFPCM(chloroform)//B3LYP/6-31G(d) Quasiharmonic Free Energy = -3411.172242

Frequencies (Top 3 out of 291)

1. -1008.8820 cm-1
2. 7.3459 cm-1
3. 9.8422 cm-1

B3LYP/6-31G(d) Molecular Geometry in Cartesian Coordinates

| | | | |
|---|---|---|---|
| C | 3.149803 | -1.431260 | 1.046709 |
| C | 3.689214 | -0.053743 | 1.491907 |
| H | 4.611656 | 0.183902 | 0.962606 |
| C | 2.876599 | 1.758489 | -0.040480 |
| N | 1.698762 | 2.387902 | -0.361942 |
| H | 0.880635 | 2.038333 | 0.132077 |
| N | 2.715099 | 0.929310 | 1.037094 |
| H | 1.762712 | 0.757179 | 1.351334 |
| C | 3.405935 | -3.493454 | -0.274943 |
| C | 5.361934 | -2.024493 | -0.051302 |
| C | 4.644972 | -4.121640 | -0.964947 |
| H | 3.084869 | -4.108311 | 0.574117 |
| C | 5.849231 | -3.458406 | -0.285161 |
| H | 5.479611 | -1.402031 | -0.947212 |
| H | 5.884587 | -1.536417 | 0.770805 |
| H | 4.631035 | -3.872663 | -2.031655 |
| H | 4.639330 | -5.212100 | -0.881627 |
| H | 6.758790 | -3.493805 | -0.891955 |
| H | 6.064225 | -3.938667 | 0.677002 |
| N | 3.931046 | -2.212121 | 0.262387 |
| O | 1.994596 | -1.773312 | 1.349698 |
| S | 4.345479 | 1.952442 | -0.849494 |
| C | 2.228459 | -3.333381 | -1.222608 |
| C | 1.175257 | -4.253698 | -1.183083 |
| C | 2.201282 | -2.316929 | -2.187310 |
| C | 0.112457 | -4.161870 | -2.083961 |
| H | 1.180939 | -5.041764 | -0.432929 |
| C | 1.138328 | -2.221004 | -3.086449 |
| C | 0.088832 | -3.142676 | -3.037760 |
| H | -0.706771 | -4.873154 | -2.031175 |
| O | -2.302837 | 1.169543 | -1.050085 |
| O | -0.256808 | 0.922728 | 1.416447 |
| C | -3.132897 | 1.114076 | -0.148722 |
| C | -2.983934 | 0.311130 | 1.093118 |
| O | -4.314552 | 1.746037 | -0.200040 |
| C | -4.549499 | 2.520767 | -1.395303 |
| H | -3.731034 | 3.222440 | -1.570896 |
| H | -4.644320 | 1.858574 | -2.259813 |
| H | -5.485506 | 3.047925 | -1.212112 |
| S | -1.271217 | -0.168170 | 1.235301 |
| C | -1.213753 | -1.275062 | 2.650845 |
| H | -0.184053 | -1.638799 | 2.691460 |
| H | -1.932588 | -2.081305 | 2.474188 |
| H | -1.472055 | -0.693940 | 3.536874 |
| C | -0.846987 | -1.250877 | -0.138987 |
| H | -1.614586 | -2.026905 | -0.190285 |
| H | 0.140928 | -1.657471 | 0.094286 |

| | | | |
|---|---|---|---|
| H | -0.847225 | -0.637184 | -1.036643 |
| C | -3.540500 | 0.887567 | 2.378519 |
| C | -2.944339 | 1.999055 | 3.000950 |
| C | -4.697122 | 0.332824 | 2.946927 |
| C | -3.482757 | 2.528608 | 4.173331 |
| H | -2.056707 | 2.448413 | 2.565563 |
| C | -5.240279 | 0.875233 | 4.112608 |
| H | -5.178786 | -0.512631 | 2.466539 |
| C | -4.633329 | 1.968326 | 4.731895 |
| H | -3.008367 | 3.385982 | 4.643113 |
| H | -6.140363 | 0.438397 | 4.536537 |
| H | -5.056813 | 2.385915 | 5.641434 |
| C | -3.864714 | -3.993443 | -1.445286 |
| C | -3.886982 | -4.203926 | -2.824609 |
| C | -4.285285 | -3.182932 | -3.691275 |
| C | -4.679208 | -1.953560 | -3.158473 |
| C | -4.675856 | -1.744392 | -1.779062 |
| C | -4.256992 | -2.758073 | -0.898796 |
| H | -3.550288 | -4.790001 | -0.776658 |
| H | -3.593094 | -5.172373 | -3.223513 |
| H | -4.300947 | -3.347292 | -4.765319 |
| H | -5.003749 | -1.153786 | -3.820144 |
| H | -5.017776 | -0.796427 | -1.373382 |
| S | -4.246173 | -2.520207 | 0.870091 |
| H | -3.605180 | -0.972016 | 0.909571 |
| H | 3.003063 | -1.583317 | -2.224230 |
| H | 1.128092 | -1.420956 | -3.821981 |
| H | -0.748637 | -3.061495 | -3.724819 |
| C | 3.970225 | 0.063652 | 3.030430 |
| C | 1.379461 | 3.403287 | -1.284063 |
| C | 2.301172 | 4.312562 | -1.820564 |
| C | 0.014270 | 3.514403 | -1.607513 |
| C | 1.824371 | 5.302261 | -2.671357 |
| H | 3.355316 | 4.253627 | -1.594633 |
| C | -0.393430 | 4.532989 | -2.451361 |
| H | -0.717739 | 2.804949 | -1.236802 |
| C | 0.484814 | 5.453277 | -3.010728 |
| H | 0.148036 | 6.240415 | -3.673700 |
| F | 2.712341 | 6.177420 | -3.186172 |
| F | -1.711074 | 4.634185 | -2.747397 |
| C | 4.570420 | 1.458114 | 3.297828 |
| H | 4.811837 | 1.567253 | 4.361842 |
| H | 5.489863 | 1.615953 | 2.722724 |
| H | 3.867692 | 2.251559 | 3.026075 |
| C | 4.996063 | -1.011323 | 3.441176 |
| H | 4.631107 | -2.026341 | 3.242960 |
| H | 5.951407 | -0.878617 | 2.919005 |
| H | 5.202593 | -0.942234 | 4.515276 |
| C | 2.696831 | -0.111325 | 3.881262 |
| H | 2.268422 | -1.110308 | 3.764898 |
| H | 2.940456 | 0.037276 | 4.940059 |
| H | 1.928594 | 0.624187 | 3.617529 |

TS Conformation 4

B3LYP/6-31G(d) Energy = -3411.894187
M06-2X/def2tzvpp/IEFPCM(chloroform) Energy = -3411.898763
M06-2X/def2tzvpp/IEFPCM(chloroform)//B3LYP/6-31G(d) Quasiharmonic Free Energy = -3411.172207

Frequencies (Top 3 out of 291)

1. -1002.0489 cm-1
2. 5.9506 cm-1
3. 11.3003 cm-1

B3LYP/6-31G(d) Molecular Geometry in Cartesian Coordinates

| | | | |
|---|---|---|---|
| C | -3.458457 | 0.352949 | 0.801628 |
| C | -3.759319 | -1.092457 | 0.341027 |
| H | -4.647986 | -1.100903 | -0.289031 |
| C | -2.677414 | -1.367168 | -1.875065 |
| N | -1.560498 | -1.806109 | -2.551565 |
| H | -1.554514 | -1.497592 | -3.515364 |
| N | -2.664910 | -1.531393 | -0.527372 |
| H | -1.745556 | -1.604167 | -0.088122 |
| C | -4.083776 | 2.724434 | 0.961491 |
| C | -5.758083 | 1.163128 | 0.073228 |
| C | -5.413285 | 3.461344 | 0.655520 |
| H | -3.876067 | 2.738639 | 2.038261 |
| C | -6.483890 | 2.364003 | 0.689036 |
| H | -5.767995 | 1.192217 | -1.022790 |
| H | -6.187112 | 0.212600 | 0.390234 |
| H | -5.359840 | 3.904018 | -0.345109 |
| H | -5.593212 | 4.273323 | 1.365770 |
| H | -7.389315 | 2.629641 | 0.135343 |
| H | -6.772635 | 2.135700 | 1.722174 |
| N | -4.378628 | 1.320730 | 0.573964 |
| O | -2.373468 | 0.616396 | 1.341006 |
| S | -3.926055 | -0.655136 | -2.766995 |
| C | -2.896607 | 3.340115 | 0.239216 |
| C | -2.684878 | 3.129538 | -1.130317 |
| C | -2.031877 | 4.193624 | 0.932859 |
| C | -1.624521 | 3.757043 | -1.785849 |
| H | -3.332221 | 2.451770 | -1.681772 |
| C | -0.974437 | 4.826618 | 0.276985 |
| C | -0.765503 | 4.608645 | -1.085949 |
| H | -1.466932 | 3.575541 | -2.845818 |
| O | 2.530350 | -0.268357 | -1.600138 |
| O | 0.204197 | -1.366954 | 0.534327 |
| C | 3.208410 | -0.450773 | -0.597003 |
| C | 2.765368 | -0.234819 | 0.803515 |
| O | 4.501381 | -0.828189 | -0.644354 |
| C | 5.031076 | -1.033504 | -1.967225 |
| H | 4.982870 | -0.107159 | -2.545603 |
| H | 6.066656 | -1.339063 | -1.815718 |
| H | 4.467044 | -1.813349 | -2.482976 |
| S | 0.989488 | -0.139122 | 0.860331 |
| C | 0.632513 | 0.398420 | 2.541200 |
| H | 0.958751 | -0.394829 | 3.214968 |
| H | 1.179103 | 1.328195 | 2.723259 |
| H | -0.449402 | 0.541745 | 2.575080 |
| C | 0.463673 | 1.267621 | -0.133682 |
| H | 0.634850 | 0.991802 | -1.171798 |
| H | -0.592277 | 1.417566 | 0.099563 |
| H | 1.074127 | 2.126635 | 0.155175 |
| C | 3.320509 | -1.159940 | 1.866820 |
| C | 4.218527 | -0.671492 | 2.827902 |
| C | 2.972039 | -2.522374 | 1.896915 |
| C | 4.751439 | -1.524214 | 3.796124 |
| H | 4.511721 | 0.373219 | 2.805228 |
| C | 3.497781 | -3.368430 | 2.872963 |
| H | 2.286284 | -2.916810 | 1.152773 |
| C | 4.389735 | -2.871504 | 3.825525 |

| | | | |
|---|---|---|---|
| H | 5.450958 | -1.131020 | 4.529037 |
| H | 3.215594 | -4.417959 | 2.883569 |
| H | 4.803718 | -3.532396 | 4.582621 |
| C | 4.676797 | 2.833791 | -0.779492 |
| C | 4.957918 | 3.457364 | -1.995640 |
| C | 4.394896 | 4.697131 | -2.305142 |
| C | 3.543745 | 5.307917 | -1.381701 |
| C | 3.247570 | 4.681392 | -0.170441 |
| C | 3.807900 | 3.432430 | 0.149486 |
| H | 5.139203 | 1.881545 | -0.536609 |
| H | 5.632322 | 2.976074 | -2.700450 |
| H | 4.622335 | 5.184133 | -3.249594 |
| H | 3.107046 | 6.279266 | -1.603632 |
| H | 2.582640 | 5.159625 | 0.543334 |
| S | 3.447693 | 2.666545 | 1.722032 |
| H | 3.132927 | 1.144421 | 1.182758 |
| H | -2.181001 | 4.357203 | 1.998112 |
| H | -0.307687 | 5.479297 | 0.834010 |
| H | 0.068833 | 5.084398 | -1.593474 |
| C | -3.999555 | -2.090604 | 1.527999 |
| C | -0.599156 | -2.787623 | -2.214929 |
| C | 0.671342 | -2.675132 | -2.796320 |
| C | -0.904564 | -3.883999 | -1.396878 |
| C | 1.609060 | -3.663060 | -2.536819 |
| H | 0.946961 | -1.815591 | -3.395251 |
| C | 0.084433 | -4.828423 | -1.160050 |
| H | -1.886652 | -4.018507 | -0.964702 |
| C | 1.356713 | -4.755713 | -1.715732 |
| H | 2.107471 | -5.512370 | -1.524809 |
| F | 2.833330 | -3.555088 | -3.097825 |
| F | -0.211847 | -5.881336 | -0.372488 |
| C | -2.736159 | -2.325574 | 2.379372 |
| H | -2.386003 | -1.397828 | 2.837832 |
| H | -2.964118 | -3.041503 | 3.178048 |
| H | -1.910758 | -2.742866 | 1.793825 |
| C | -4.479054 | -3.432822 | 0.940384 |
| H | -3.723633 | -3.881166 | 0.288356 |
| H | -4.688148 | -4.145460 | 1.746582 |
| H | -5.397074 | -3.309137 | 0.353462 |
| C | -5.111246 | -1.532460 | 2.440637 |
| H | -6.056890 | -1.404998 | 1.900089 |
| H | -5.301093 | -2.230933 | 3.263318 |
| H | -4.834032 | -0.570033 | 2.885311 |

TS Conformation 5

B3LYP/6-31G(d) Energy = -3411.899548
M06-2X/def2tzvpp/IEFPCM(chloroform) Energy = -3411.899227
M06-2X/def2tzvpp/IEFPCM(chloroform)//B3LYP/6-31G(d) Quasiharmonic Free Energy = -3411.171603

Frequencies (Top 3 out of 291)

1. -1027.9929 cm-1
2. 8.5578 cm-1
3. 10.3617 cm-1

B3LYP/6-31G(d) Molecular Geometry in Cartesian Coordinates

| | | | |
|---|---|---|---|
| C | 3.039451 | -1.473257 | 1.078992 |
| C | 3.652974 | -0.107536 | 1.458764 |
| H | 4.576806 | 0.064869 | 0.906805 |

| | | | |
|---|---|---|---|
| C | 2.915552 | 1.715902 | -0.099672 |
| N | 1.768334 | 2.403721 | -0.414873 |
| H | 0.942585 | 2.112067 | 0.103312 |
| N | 2.720507 | 0.906138 | 0.986526 |
| H | 1.764872 | 0.788233 | 1.315111 |
| C | 3.194817 | -3.646261 | -0.067789 |
| C | 5.216640 | -2.254443 | 0.028627 |
| C | 4.403377 | -4.390595 | -0.693377 |
| H | 2.845319 | -4.167029 | 0.831372 |
| C | 5.638480 | -3.723922 | -0.074528 |
| H | 5.355306 | -1.724059 | -0.922095 |
| H | 5.765960 | -1.715486 | 0.799484 |
| H | 4.399372 | -4.241204 | -1.778549 |
| H | 4.347885 | -5.467302 | -0.509951 |
| H | 6.543797 | -3.854911 | -0.674523 |
| H | 5.834488 | -4.124246 | 0.927448 |
| N | 3.781210 | -2.348769 | 0.360415 |
| O | 1.862747 | -1.729226 | 1.385587 |
| S | 4.384105 | 1.823541 | -0.923887 |
| C | 2.025182 | -3.515537 | -1.029176 |
| C | 2.062769 | -2.615006 | -2.102551 |
| C | 0.911561 | -4.351185 | -0.888985 |
| C | 1.005773 | -2.550830 | -3.011384 |
| H | 2.912933 | -1.947158 | -2.219904 |
| C | -0.145525 | -4.292082 | -1.799394 |
| C | -0.102216 | -3.389849 | -2.864172 |
| H | 1.047314 | -1.841228 | -3.833674 |
| O | -2.403555 | 1.403527 | -1.099211 |
| O | -0.256994 | 1.065212 | 1.385671 |
| C | -3.136871 | 1.423518 | -0.114705 |
| C | -2.989257 | 0.524855 | 1.059354 |
| O | -4.208076 | 2.219985 | -0.013224 |
| C | -4.474176 | 3.055478 | -1.159956 |
| H | -5.292357 | 3.706159 | -0.852226 |
| H | -3.590447 | 3.636865 | -1.431491 |
| H | -4.775153 | 2.439595 | -2.011516 |
| S | -1.288125 | -0.002837 | 1.162225 |
| C | -1.246568 | -1.186030 | 2.515254 |
| H | -1.993156 | -1.957818 | 2.301467 |
| H | -0.227136 | -1.580024 | 2.521959 |
| H | -1.480071 | -0.646188 | 3.433511 |
| C | -0.875151 | -1.009923 | -0.270978 |
| H | -0.849985 | -0.340433 | -1.127700 |
| H | 0.097109 | -1.457324 | -0.048324 |
| H | -1.657159 | -1.763822 | -0.385287 |
| C | -3.530296 | 0.995160 | 2.392288 |
| C | -4.695413 | 0.412391 | 2.913234 |
| C | -2.912064 | 2.034015 | 3.110800 |
| C | -5.224480 | 0.854698 | 4.126724 |
| H | -5.197771 | -0.374488 | 2.359787 |
| C | -3.436198 | 2.463230 | 4.329660 |
| H | -2.018680 | 2.506119 | 2.712667 |
| C | -4.594771 | 1.874630 | 4.840734 |
| H | -6.131028 | 0.397137 | 4.513223 |
| H | -2.944689 | 3.265366 | 4.873571 |
| H | -5.006872 | 2.214092 | 5.787248 |
| C | -3.988376 | -1.793847 | -2.112569 |
| C | -3.911472 | -2.217283 | -3.441173 |
| C | -4.034188 | -3.569056 | -3.766899 |
| C | -4.231585 | -4.497822 | -2.740396 |
| C | -4.301344 | -4.081667 | -1.412045 |
| C | -4.183397 | -2.719703 | -1.072021 |

| | | | |
|---|---|---|---|
| H | -3.895022 | -0.735721 | -1.889218 |
| H | -3.762969 | -1.478644 | -4.225587 |
| H | -3.985882 | -3.893972 | -4.802751 |
| H | -4.335100 | -5.554802 | -2.975134 |
| H | -4.456467 | -4.810777 | -0.621691 |
| S | -4.316600 | -2.255414 | 0.645277 |
| H | -3.623618 | -0.719156 | 0.736077 |
| H | 0.866592 | -5.046670 | -0.053489 |
| H | -1.012547 | -4.933727 | -1.670608 |
| H | -0.933984 | -3.333878 | -3.559971 |
| C | 3.963104 | 0.051122 | 2.989854 |
| C | 1.488724 | 3.423225 | -1.344813 |
| C | 0.124610 | 3.615234 | -1.634460 |
| C | 2.450022 | 4.263879 | -1.922129 |
| C | -0.242035 | 4.644708 | -2.483958 |
| H | -0.640751 | 2.959499 | -1.233512 |
| C | 2.012583 | 5.268454 | -2.776687 |
| H | 3.504209 | 4.141683 | -1.723481 |
| C | 0.676258 | 5.498998 | -3.082205 |
| H | 0.370997 | 6.295799 | -3.748860 |
| F | -1.558361 | 4.825062 | -2.745246 |
| F | 2.939002 | 6.077281 | -3.330264 |
| C | 4.629141 | 1.425549 | 3.198819 |
| H | 3.958603 | 2.240286 | 2.909544 |
| H | 4.891609 | 1.560215 | 4.254893 |
| H | 5.545524 | 1.521591 | 2.605610 |
| C | 4.945116 | -1.052544 | 3.430386 |
| H | 5.163865 | -0.954040 | 4.499743 |
| H | 4.535611 | -2.057443 | 3.271889 |
| H | 5.900815 | -0.979696 | 2.897365 |
| C | 2.693926 | -0.034583 | 3.860568 |
| H | 2.216753 | -1.015270 | 3.784773 |
| H | 2.958037 | 0.138493 | 4.910659 |
| H | 1.957238 | 0.726599 | 3.580477 |

TS Conformation 6 (**TS-2**)

B3LYP/6-31G(d) Energy = -3411.894149
M06-2X/def2tzvpp/IEFPCM(chloroform) Energy = -3411.897427
M06-2X/def2tzvpp/IEFPCM(chloroform)//B3LYP/6-31G(d) Quasiharmonic Free Energy = -3411.171553

Frequencies (Top 3 out of 291)

1. -981.1927 cm-1
2. 7.9105 cm-1
3. 12.2209 cm-1

B3LYP/6-31G(d) Molecular Geometry in Cartesian Coordinates

| | | | |
|---|---|---|---|
| C | -3.095264 | -1.505982 | -0.251137 |
| C | -3.173707 | -0.823616 | -1.631268 |
| H | -4.085321 | -0.234555 | -1.721003 |
| C | -2.216103 | 1.484529 | -1.508988 |
| N | -1.010205 | 2.088409 | -1.256177 |
| H | -0.249281 | 1.454248 | -1.017971 |
| N | -2.074213 | 0.135015 | -1.679698 |
| H | -1.159118 | -0.263564 | -1.483761 |
| C | -4.112710 | -2.080606 | 1.900864 |
| C | -5.485958 | -0.817384 | 0.295042 |
| C | -5.605060 | -2.131158 | 2.309904 |
| H | -3.699443 | -3.086605 | 1.779270 |

| | | | |
|---|---|---|---|
| C | -6.199859 | -0.877554 | 1.652689 |
| H | -5.368559 | 0.203930 | -0.077679 |
| H | -6.028098 | -1.403186 | -0.458512 |
| H | -5.733912 | -2.156122 | 3.394977 |
| H | -6.066886 | -3.033702 | 1.891170 |
| H | -5.957349 | 0.011749 | 2.244630 |
| H | -7.287193 | -0.921728 | 1.543406 |
| N | -4.170709 | -1.435015 | 0.574520 |
| O | -2.049776 | -2.065576 | 0.104657 |
| S | -3.696551 | 2.292176 | -1.593897 |
| C | -3.241985 | -1.337265 | 2.910553 |
| C | -2.936871 | 0.023361 | 2.792316 |
| C | -2.757458 | -2.039217 | 4.023482 |
| C | -2.168170 | 0.668985 | 3.764072 |
| H | -3.281295 | 0.581573 | 1.926565 |
| C | -1.997251 | -1.396526 | 5.001420 |
| C | -1.700054 | -0.036172 | 4.874468 |
| H | -1.928805 | 1.721598 | 3.645472 |
| O | 0.957268 | -0.111003 | -0.863356 |
| O | 0.969097 | 1.248159 | 2.110792 |
| C | 2.181229 | -0.068024 | -0.694245 |
| C | 2.858106 | -0.222845 | 0.609585 |
| O | 3.055379 | 0.066741 | -1.692138 |
| C | 2.515463 | 0.064578 | -3.028059 |
| H | 3.366274 | 0.267906 | -3.677472 |
| H | 2.089969 | -0.916726 | -3.251535 |
| H | 1.752939 | 0.839274 | -3.137412 |
| S | 1.658436 | -0.055492 | 1.928788 |
| C | 2.629733 | -0.453615 | 3.396536 |
| H | 3.091323 | -1.435469 | 3.263890 |
| H | 3.386099 | 0.322749 | 3.514922 |
| H | 1.931763 | -0.449448 | 4.236299 |
| C | 0.507278 | -1.437775 | 1.843208 |
| H | -0.169199 | -1.286145 | 1.002636 |
| H | 1.105642 | -2.346598 | 1.711493 |
| H | -0.045585 | -1.434913 | 2.785700 |
| C | 4.105813 | 0.598957 | 0.860771 |
| C | 4.054287 | 2.004416 | 0.882454 |
| C | 5.339326 | -0.037769 | 1.064275 |
| C | 5.208838 | 2.752398 | 1.109254 |
| H | 3.112451 | 2.520536 | 0.722694 |
| C | 6.494011 | 0.715657 | 1.282278 |
| H | 5.389796 | -1.122177 | 1.036903 |
| C | 6.432303 | 2.109479 | 1.309153 |
| H | 5.148770 | 3.837092 | 1.124100 |
| H | 7.443700 | 0.208637 | 1.430395 |
| H | 7.332855 | 2.693068 | 1.481023 |
| C | 4.492994 | -4.139698 | -1.546217 |
| C | 4.351168 | -4.512766 | -2.882714 |
| C | 3.109934 | -4.419128 | -3.516043 |
| C | 2.009169 | -3.954868 | -2.792891 |
| C | 2.145080 | -3.589096 | -1.451371 |
| C | 3.392819 | -3.669983 | -0.808626 |
| H | 5.461380 | -4.209381 | -1.059771 |
| H | 5.216717 | -4.875873 | -3.431995 |
| H | 3.000364 | -4.711102 | -4.557192 |
| H | 1.031802 | -3.892383 | -3.266077 |
| H | 1.274316 | -3.249731 | -0.897814 |
| S | 3.600345 | -3.222205 | 0.910415 |
| H | 3.195079 | -1.599030 | 0.727788 |
| H | -2.975934 | -3.100619 | 4.123808 |
| H | -1.637338 | -1.957806 | 5.860194 |

| | | | |
|---|---|---|---|
| H | -1.111767 | 0.470085 | 5.635570 |
| C | -3.143076 | -1.829396 | -2.832205 |
| C | -0.677089 | 3.449733 | -1.111434 |
| C | -1.241875 | 4.471338 | -1.885904 |
| C | 0.345662 | 3.742351 | -0.194780 |
| C | -0.763380 | 5.763479 | -1.712648 |
| H | -2.031547 | 4.275343 | -2.596828 |
| C | 0.794832 | 5.049313 | -0.088049 |
| H | 0.746979 | 2.974858 | 0.458006 |
| C | 0.261344 | 6.094301 | -0.831361 |
| H | 0.618703 | 7.111500 | -0.729742 |
| F | -1.302733 | 6.749257 | -2.457883 |
| F | 1.794261 | 5.313585 | 0.783360 |
| C | -1.798778 | -2.574540 | -2.945001 |
| H | -1.577557 | -3.148515 | -2.041984 |
| H | -1.831748 | -3.262681 | -3.798488 |
| H | -0.968944 | -1.879641 | -3.118960 |
| C | -3.391427 | -1.028911 | -4.125612 |
| H | -2.622881 | -0.263195 | -4.270921 |
| H | -3.373435 | -1.698196 | -4.994026 |
| H | -4.364517 | -0.524871 | -4.105510 |
| C | -4.277812 | -2.856122 | -2.646963 |
| H | -5.255745 | -2.365115 | -2.567497 |
| H | -4.319482 | -3.530577 | -3.509779 |
| H | -4.128274 | -3.474298 | -1.754165 |

TS Conformation 7

B3LYP/6-31G(d) Energy = -3411.89911
M06-2X/def2tzvpp/IEFPCM(chloroform) Energy = -3411.898308
M06-2X/def2tzvpp/IEFPCM(chloroform)//B3LYP/6-31G(d) Quasiharmonic Free Energy = -3411.171411

Frequencies (Top 3 out of 291)

1. -1000.3356 cm-1
2. 9.8184 cm-1
3. 10.9483 cm-1

B3LYP/6-31G(d) Molecular Geometry in Cartesian Coordinates

| | | | |
|---|---|---|---|
| C | -2.633462 | -2.430307 | -0.651475 |
| C | -3.288003 | -1.562203 | -1.742828 |
| H | -4.353165 | -1.446608 | -1.548084 |
| C | -3.361884 | 0.840342 | -1.047087 |
| N | -2.502844 | 1.889571 | -0.824296 |
| H | -1.514523 | 1.662900 | -0.909144 |
| N | -2.715130 | -0.227446 | -1.604486 |
| H | -1.697869 | -0.193814 | -1.626116 |
| C | -2.818200 | -3.860756 | 1.328267 |
| C | -4.907927 | -3.104713 | 0.265892 |
| C | -4.010076 | -4.717625 | 1.817607 |
| H | -2.029033 | -4.485340 | 0.899758 |
| C | -5.225066 | -3.805627 | 1.594977 |
| H | -5.327318 | -2.096479 | 0.208047 |
| H | -5.281484 | -3.688100 | -0.584443 |
| H | -3.888796 | -5.033520 | 2.856738 |
| H | -4.091330 | -5.617152 | 1.195194 |
| H | -5.298680 | -3.068637 | 2.401711 |
| H | -6.171665 | -4.351660 | 1.553283 |
| N | -3.427289 | -3.064617 | 0.241674 |
| O | -1.394937 | -2.493248 | -0.568100 |

| | | | |
|---|---|---|---|
| S | -5.009700 | 0.840140 | -0.675098 |
| C | -2.199503 | -3.006379 | 2.430678 |
| C | -2.643273 | -1.709843 | 2.719407 |
| C | -1.174169 | -3.555089 | 3.213576 |
| C | -2.079459 | -0.983263 | 3.771380 |
| H | -3.418505 | -1.252036 | 2.111218 |
| C | -0.610082 | -2.832170 | 4.264650 |
| C | -1.063325 | -1.541351 | 4.548491 |
| H | -2.437146 | 0.021639 | 3.979705 |
| O | 3.297310 | 0.481913 | -2.558197 |
| O | 0.228818 | 0.579654 | -1.086882 |
| C | 3.541684 | 0.974468 | -1.464383 |
| C | 2.904257 | 0.571941 | -0.180892 |
| O | 4.489018 | 1.902762 | -1.259802 |
| C | 5.293814 | 2.238486 | -2.404649 |
| H | 5.915066 | 3.074061 | -2.082184 |
| H | 5.917099 | 1.384791 | -2.682789 |
| H | 4.662379 | 2.524944 | -3.248697 |
| S | 1.346613 | -0.228274 | -0.497759 |
| C | 1.638985 | -1.710360 | -1.477595 |
| H | 2.480751 | -2.239873 | -1.016789 |
| H | 0.704434 | -2.273899 | -1.434165 |
| H | 1.890135 | -1.370739 | -2.480698 |
| C | 0.860732 | -0.888640 | 1.103684 |
| H | -0.026122 | -1.501890 | 0.924442 |
| H | 1.689537 | -1.482736 | 1.496708 |
| H | 0.641970 | -0.046406 | 1.760464 |
| C | 2.791600 | 1.597082 | 0.924054 |
| C | 3.364373 | 1.351476 | 2.180357 |
| C | 2.115957 | 2.811772 | 0.712543 |
| C | 3.259143 | 2.299653 | 3.200242 |
| H | 3.904461 | 0.424127 | 2.347539 |
| C | 2.001815 | 3.752870 | 1.735096 |
| H | 1.685737 | 3.019512 | -0.263623 |
| C | 2.574195 | 3.495530 | 2.983655 |
| H | 3.714872 | 2.099410 | 4.166288 |
| H | 1.456088 | 4.674932 | 1.560223 |
| H | 2.488230 | 4.228287 | 3.781594 |
| C | 7.260204 | -1.608549 | 0.714434 |
| C | 8.480955 | -1.448291 | 0.058601 |
| C | 8.525720 | -1.296748 | -1.328732 |
| C | 7.332365 | -1.314237 | -2.053657 |
| C | 6.108919 | -1.484605 | -1.402443 |
| C | 6.053550 | -1.629464 | -0.005195 |
| H | 7.231569 | -1.718824 | 1.794433 |
| H | 9.401655 | -1.437789 | 0.637386 |
| H | 9.477335 | -1.171045 | -1.838342 |
| H | 7.350593 | -1.210374 | -3.136498 |
| H | 5.191401 | -1.507481 | -1.982727 |
| S | 4.511742 | -1.878192 | 0.864175 |
| H | 3.675788 | -0.546569 | 0.285544 |
| H | -0.809770 | -4.556595 | 2.993547 |
| H | 0.186779 | -3.274032 | 4.856934 |
| H | -0.625047 | -0.975300 | 5.365904 |
| C | -3.141380 | -2.131916 | -3.195907 |
| C | -2.736580 | 3.212100 | -0.401471 |
| C | -3.934110 | 3.906510 | -0.618945 |
| C | -1.650539 | 3.864351 | 0.209240 |
| C | -4.011078 | 5.229851 | -0.204440 |
| H | -4.786053 | 3.437686 | -1.087916 |
| C | -1.789937 | 5.190410 | 0.586678 |
| H | -0.718417 | 3.344918 | 0.401769 |

| | | | |
|---|---|---|---|
| C | -2.961717 | 5.912067 | 0.400633 |
| H | -3.053134 | 6.946231 | 0.708168 |
| F | -5.163631 | 5.895189 | -0.415294 |
| F | -0.738368 | 5.811123 | 1.170610 |
| C | -1.676510 | -2.207063 | -3.665754 |
| H | -1.090796 | -2.898438 | -3.054648 |
| H | -1.641849 | -2.556435 | -4.704390 |
| H | -1.190821 | -1.224914 | -3.638600 |
| C | -3.931390 | -1.209770 | -4.145700 |
| H | -4.987257 | -1.147674 | -3.858812 |
| H | -3.524456 | -0.193917 | -4.143189 |
| H | -3.879818 | -1.593106 | -5.171515 |
| C | -3.757561 | -3.544394 | -3.236600 |
| H | -3.228278 | -4.242570 | -2.577111 |
| H | -4.815212 | -3.530097 | -2.946439 |
| H | -3.702875 | -3.949448 | -4.253340 |

TS Conformation 8

B3LYP/6-31G(d) Energy = -3411.899073
M06-2X/def2tzvpp/IEFPCM(chloroform) Energy = -3411.898233
M06-2X/def2tzvpp/IEFPCM(chloroform)//B3LYP/6-31G(d) Quasiharmonic Free Energy = -3411.171041

Frequencies (Top 3 out of 291)

1. -992.6756 cm-1
2. 7.8522 cm-1
3. 10.6633 cm-1

B3LYP/6-31G(d) Molecular Geometry in Cartesian Coordinates

| | | | |
|---|---|---|---|
| C | 3.403818 | -1.591999 | 0.571254 |
| C | 3.911030 | -0.327285 | 1.299402 |
| H | 4.810152 | 0.054383 | 0.816230 |
| C | 2.986365 | 1.731115 | 0.205337 |
| N | 1.786544 | 2.389253 | 0.086956 |
| H | 1.004779 | 1.935643 | 0.554634 |
| N | 2.896193 | 0.697836 | 1.099381 |
| H | 1.964002 | 0.438544 | 1.414142 |
| C | 3.700160 | -3.330971 | -1.144555 |
| C | 5.612733 | -1.871485 | -0.651952 |
| C | 4.948966 | -3.772627 | -1.952669 |
| H | 3.405151 | -4.111304 | -0.433292 |
| C | 6.142021 | -3.214102 | -1.166929 |
| H | 5.692268 | -1.082770 | -1.410556 |
| H | 6.133906 | -1.533824 | 0.243100 |
| H | 4.912460 | -3.322869 | -2.950820 |
| H | 4.978138 | -4.858176 | -2.082091 |
| H | 7.042399 | -3.098815 | -1.777459 |
| H | 6.387572 | -3.865478 | -0.319555 |
| N | 4.195009 | -2.167938 | -0.364619 |
| O | 2.262176 | -2.025370 | 0.801418 |
| S | 4.402651 | 2.112818 | -0.630219 |
| C | 2.510028 | -3.022260 | -2.038326 |
| C | 1.496841 | -3.974231 | -2.197236 |
| C | 2.438724 | -1.830593 | -2.772848 |
| C | 0.434258 | -3.747815 | -3.074458 |
| H | 1.536128 | -4.898810 | -1.625165 |
| C | 1.375234 | -1.599322 | -3.646217 |
| C | 0.370676 | -2.558336 | -3.802557 |
| H | -0.347398 | -4.494952 | -3.180951 |

| | | | |
|---|---|---|---|
| O | -2.285273 | 1.260490 | -0.721235 |
| O | -0.057661 | 0.503525 | 1.572020 |
| C | -3.010194 | 0.965841 | 0.225883 |
| C | -2.751452 | -0.166821 | 1.152928 |
| O | -4.157051 | 1.593870 | 0.502929 |
| C | -4.558194 | 2.611384 | -0.439519 |
| H | -3.738756 | 3.306608 | -0.634056 |
| H | -4.871585 | 2.138719 | -1.373914 |
| H | -5.400203 | 3.118046 | 0.031676 |
| S | -1.010542 | -0.549980 | 1.087669 |
| C | -0.828329 | -2.027146 | 2.097343 |
| H | 0.219184 | -2.321260 | 1.994594 |
| H | -1.508377 | -2.793590 | 1.716537 |
| H | -1.076422 | -1.758638 | 3.124682 |
| C | -0.575752 | -1.138368 | -0.557761 |
| H | -1.344440 | -1.861662 | -0.851674 |
| H | 0.415686 | -1.589202 | -0.464884 |
| H | -0.586028 | -0.270850 | -1.213521 |
| C | -3.255456 | -0.072281 | 2.574926 |
| C | -2.853909 | 0.981661 | 3.414545 |
| C | -4.144395 | -1.036478 | 3.073033 |
| C | -3.327865 | 1.066001 | 4.722509 |
| H | -2.168460 | 1.735570 | 3.038163 |
| C | -4.622094 | -0.944833 | 4.381655 |
| H | -4.469664 | -1.845822 | 2.425862 |
| C | -4.214214 | 0.102023 | 5.209016 |
| H | -3.008898 | 1.886513 | 5.359485 |
| H | -5.316781 | -1.693844 | 4.752087 |
| H | -4.587419 | 0.170369 | 6.227435 |
| C | -4.688327 | -0.996455 | -2.192637 |
| C | -5.623599 | -0.445901 | -3.071418 |
| C | -6.946000 | -0.894057 | -3.072040 |
| C | -7.323963 | -1.898834 | -2.179011 |
| C | -6.395807 | -2.443740 | -1.291647 |
| C | -5.061981 | -2.001612 | -1.283378 |
| H | -3.661105 | -0.645272 | -2.215601 |
| H | -5.309483 | 0.327574 | -3.769037 |
| H | -7.670748 | -0.468996 | -3.761303 |
| H | -8.349956 | -2.259391 | -2.167794 |
| H | -6.698336 | -3.218924 | -0.593797 |
| S | -3.892327 | -2.745281 | -0.154795 |
| H | -3.299333 | -1.332448 | 0.532761 |
| H | 3.207390 | -1.071289 | -2.650610 |
| H | 1.332112 | -0.667947 | -4.204651 |
| H | -0.457365 | -2.378040 | -4.482419 |
| C | 4.230944 | -0.543584 | 2.820083 |
| C | 1.401328 | 3.556855 | -0.600870 |
| C | 2.280479 | 4.571569 | -1.002439 |
| C | 0.018276 | 3.699054 | -0.818777 |
| C | 1.744198 | 5.696037 | -1.617901 |
| H | 3.346647 | 4.492667 | -0.851502 |
| C | -0.449317 | 4.852016 | -1.425950 |
| H | -0.684933 | 2.917292 | -0.552216 |
| C | 0.385472 | 5.880723 | -1.845722 |
| H | 0.002022 | 6.773218 | -2.324237 |
| F | 2.591294 | 6.672342 | -2.003091 |
| F | -1.782239 | 4.980242 | -1.622405 |
| C | 4.790095 | 0.779577 | 3.379671 |
| H | 5.060147 | 0.658426 | 4.435419 |
| H | 5.685933 | 1.098385 | 2.834602 |
| H | 4.052589 | 1.584614 | 3.308260 |
| C | 5.304422 | -1.640844 | 2.962856 |

| | | | |
|---|---|---|---|
| H | 4.971272 | -2.600363 | 2.549302 |
| H | 6.241133 | -1.356795 | 2.468193 |
| H | 5.534991 | -1.804057 | 4.021747 |
| C | 2.988666 | -0.954225 | 3.635304 |
| H | 2.591233 | -1.918260 | 3.306930 |
| H | 3.257571 | -1.036928 | 4.695128 |
| H | 2.187719 | -0.210264 | 3.560474 |

TS Conformation 9

B3LYP/6-31G(d) Energy = -3411.89393
M06-2X/def2tzvpp/IEFPCM(chloroform) Energy = -3411.898008
M06-2X/def2tzvpp/IEFPCM(chloroform)//B3LYP/6-31G(d) Quasiharmonic Free Energy = -3411.171035

Frequencies (Top 3 out of 291)

1. -1016.7714 cm-1
2. 10.4436 cm-1
3. 12.6970 cm-1

B3LYP/6-31G(d) Molecular Geometry in Cartesian Coordinates

| | | | |
|---|---|---|---|
| C | -3.126236 | -0.078350 | 0.935122 |
| C | -3.163358 | -1.611412 | 1.118740 |
| H | -4.105688 | -2.006587 | 0.741028 |
| C | -2.334641 | -2.626904 | -0.989807 |
| N | -1.243641 | -3.134670 | -1.658121 |
| H | -1.428661 | -3.266471 | -2.644283 |
| N | -2.118232 | -2.203230 | 0.280909 |
| H | -1.158545 | -1.939908 | 0.511744 |
| C | -4.249394 | 2.040233 | 0.435515 |
| C | -5.612537 | -0.008585 | 0.391979 |
| C | -5.754422 | 2.393423 | 0.498186 |
| H | -3.702168 | 2.474874 | 1.277384 |
| C | -6.432435 | 1.174346 | -0.142442 |
| H | -5.566793 | -0.845545 | -0.309636 |
| H | -6.020439 | -0.364700 | 1.345875 |
| H | -5.976406 | 3.333371 | -0.013175 |
| H | -6.063710 | 2.492963 | 1.546087 |
| H | -6.351031 | 1.226274 | -1.233360 |
| H | -7.492523 | 1.086135 | 0.111911 |
| N | -4.265982 | 0.570569 | 0.598430 |
| O | -2.059440 | 0.536443 | 1.085589 |
| S | -3.813008 | -2.557458 | -1.812463 |
| C | -3.583587 | 2.508555 | -0.855295 |
| C | -3.118438 | 3.829008 | -0.927781 |
| C | -3.460786 | 1.689677 | -1.984266 |
| C | -2.551405 | 4.325410 | -2.101257 |
| H | -3.196982 | 4.473311 | -0.054079 |
| C | -2.888835 | 2.184856 | -3.159720 |
| C | -2.434248 | 3.502274 | -3.224210 |
| H | -2.196448 | 5.352075 | -2.138150 |
| O | 2.335224 | -0.669109 | -2.396262 |
| O | 0.744897 | -1.133909 | 0.503534 |
| C | 3.191995 | -0.318750 | -1.594474 |
| C | 2.937542 | 0.417510 | -0.328178 |
| O | 4.508892 | -0.488500 | -1.801043 |
| C | 4.863045 | -1.132701 | -3.038687 |
| H | 4.486065 | -0.560336 | -3.889877 |
| H | 5.952912 | -1.157650 | -3.045442 |
| H | 4.452003 | -2.143972 | -3.067130 |

| | | | |
|---|---|---|---|
| S | 1.234701 | 0.230539 | 0.141878 |
| C | 1.027787 | 1.342492 | 1.544041 |
| H | 1.355225 | 2.344805 | 1.260832 |
| H | -0.037356 | 1.307400 | 1.780685 |
| H | 1.635250 | 0.946829 | 2.358881 |
| C | 0.219859 | 0.976451 | -1.147534 |
| H | 0.698280 | 1.914323 | -1.447586 |
| H | 0.213436 | 0.267046 | -1.972653 |
| H | -0.768581 | 1.121560 | -0.707440 |
| C | 3.904100 | 0.214398 | 0.816673 |
| C | 4.921314 | 1.157283 | 1.027967 |
| C | 3.844926 | -0.915411 | 1.651337 |
| C | 5.849981 | 0.979473 | 2.053682 |
| H | 4.984959 | 2.020761 | 0.373415 |
| C | 4.763681 | -1.079268 | 2.688953 |
| H | 3.080265 | -1.668574 | 1.484529 |
| C | 5.769567 | -0.133233 | 2.892698 |
| H | 6.636281 | 1.715698 | 2.198723 |
| H | 4.699963 | -1.955524 | 3.328851 |
| H | 6.490112 | -0.266473 | 3.695370 |
| C | 3.099821 | 4.608679 | 1.121593 |
| C | 2.545091 | 5.394195 | 2.133057 |
| C | 1.268371 | 5.941127 | 1.985653 |
| C | 0.552512 | 5.692359 | 0.812930 |
| C | 1.101142 | 4.900610 | -0.197203 |
| C | 2.384919 | 4.339227 | -0.060801 |
| H | 4.099217 | 4.201425 | 1.239888 |
| H | 3.118327 | 5.585903 | 3.037229 |
| H | 0.840143 | 6.557206 | 2.771918 |
| H | -0.440568 | 6.116277 | 0.681213 |
| H | 0.537238 | 4.712213 | -1.106377 |
| S | 3.085521 | 3.348710 | -1.377446 |
| H | 2.990175 | 1.821337 | -0.744446 |
| H | -3.789672 | 0.654514 | -1.945621 |
| H | -2.798557 | 1.533481 | -4.025022 |
| H | -1.986966 | 3.884259 | -4.137639 |
| C | -3.031279 | -2.057435 | 2.618317 |
| C | -0.046190 | -3.713906 | -1.175734 |
| C | 1.078558 | -3.660764 | -2.010201 |
| C | 0.017061 | -4.389960 | 0.050179 |
| C | 2.244619 | -4.282923 | -1.590737 |
| H | 1.069228 | -3.104327 | -2.939572 |
| C | 1.220030 | -4.974819 | 0.421176 |
| H | -0.843635 | -4.480693 | 0.698590 |
| C | 2.358867 | -4.946668 | -0.375164 |
| H | 3.283327 | -5.418653 | -0.066709 |
| F | 3.328985 | -4.231408 | -2.394949 |
| F | 1.278632 | -5.622658 | 1.601638 |
| C | -1.649436 | -1.733631 | 3.219155 |
| H | -1.448690 | -0.659823 | 3.203599 |
| H | -1.615562 | -2.076795 | 4.260023 |
| H | -0.836161 | -2.235383 | 2.684595 |
| C | -3.281146 | -3.576907 | 2.692172 |
| H | -3.223092 | -3.919102 | 3.731882 |
| H | -4.273270 | -3.839445 | 2.305913 |
| H | -2.539190 | -4.137407 | 2.115729 |
| C | -4.115484 | -1.343462 | 3.451165 |
| H | -5.123173 | -1.571530 | 3.083782 |
| H | -4.064399 | -1.678764 | 4.493107 |
| H | -3.985286 | -0.255489 | 3.449817 |

TS Conformation 10

B3LYP/6-31G(d) Energy = -3411.89812
M06-2X/def2tzvpp/IEFPCM(chloroform) Energy = -3411.898008
M06-2X/def2tzvpp/IEFPCM(chloroform)//B3LYP/6-31G(d) Quasiharmonic Free Energy = -3411.17099

Frequencies (Top 3 out of 291)

1. -991.0075 cm-1
2. 8.2552 cm-1
3. 10.8120 cm-1

B3LYP/6-31G(d) Molecular Geometry in Cartesian Coordinates

| | | | |
|---|---|---|---|
| C | 3.133771 | -1.990269 | 0.764751 |
| C | 3.720310 | -0.829419 | 1.598481 |
| H | 4.729160 | -0.590988 | 1.262301 |
| C | 3.303830 | 1.368231 | 0.463381 |
| N | 2.251519 | 2.208651 | 0.192301 |
| H | 1.346169 | 1.873911 | 0.514501 |
| N | 2.918160 | 0.345641 | 1.288041 |
| H | 1.920340 | 0.232881 | 1.451771 |
| C | 3.403792 | -3.745269 | -0.938969 |
| C | 5.427631 | -2.611688 | -0.132999 |
| C | 4.673312 | -4.376738 | -1.569169 |
| H | 2.888252 | -4.471669 | -0.299909 |
| C | 5.809762 | -4.019158 | -0.603059 |
| H | 5.741761 | -1.842558 | -0.849739 |
| H | 5.857091 | -2.365488 | 0.837381 |
| H | 4.855982 | -3.922478 | -2.549069 |
| H | 4.550482 | -5.452458 | -1.723229 |
| H | 6.797342 | -4.046167 | -1.072799 |
| H | 5.821912 | -4.705158 | 0.252441 |
| N | 3.954191 | -2.679259 | -0.063529 |
| O | 1.916771 | -2.236059 | 0.813611 |
| S | 4.874510 | 1.538432 | -0.130809 |
| C | 2.421701 | -3.247549 | -1.986969 |
| C | 2.645021 | -2.054959 | -2.688739 |
| C | 1.307982 | -4.027060 | -2.318849 |
| C | 1.770581 | -1.653169 | -3.699269 |
| H | 3.495671 | -1.427309 | -2.434229 |
| C | 0.434282 | -3.630290 | -3.333379 |
| C | 0.663551 | -2.440760 | -4.027309 |
| H | 1.954880 | -0.723039 | -4.230349 |
| O | -1.729351 | 1.714799 | -1.283469 |
| O | -0.069370 | 0.623990 | 1.305641 |
| C | -2.660190 | 1.525779 | -0.507959 |
| C | -2.740380 | 0.398799 | 0.457051 |
| O | -3.764231 | 2.287429 | -0.490199 |
| C | -3.793111 | 3.356849 | -1.460989 |
| H | -4.687671 | 3.931628 | -1.222699 |
| H | -2.895871 | 3.974799 | -1.382659 |
| H | -3.858691 | 2.944249 | -2.470759 |
| S | -1.093710 | -0.266350 | 0.667341 |
| C | -1.321429 | -1.740531 | 1.670671 |
| H | -2.085229 | -2.360941 | 1.190011 |
| H | -0.342939 | -2.225850 | 1.703401 |
| H | -1.644989 | -1.416081 | 2.660421 |
| C | -0.508630 | -0.927000 | -0.904589 |
| H | 0.356361 | -1.551620 | -0.667159 |
| H | -1.323839 | -1.496511 | -1.358539 |
| H | -0.251680 | -0.071280 | -1.524439 |

| | | | |
|---|---|---|---|
| C | -3.417920 | 0.629469 | 1.787491 |
| C | -4.436720 | -0.230902 | 2.223191 |
| C | -3.039100 | 1.705879 | 2.608741 |
| C | -5.066980 | -0.012712 | 3.449591 |
| H | -4.744739 | -1.059722 | 1.594841 |
| C | -3.667701 | 1.918819 | 3.834011 |
| H | -2.248991 | 2.376619 | 2.283181 |
| C | -4.684260 | 1.058898 | 4.256941 |
| H | -5.862120 | -0.680712 | 3.768761 |
| H | -3.365081 | 2.756039 | 4.456961 |
| H | -5.177290 | 1.226748 | 5.210761 |
| C | -6.561039 | -2.929362 | -0.932899 |
| C | -7.945479 | -2.766253 | -0.916929 |
| C | -8.509269 | -1.488353 | -0.898149 |
| C | -7.667320 | -0.375123 | -0.901779 |
| C | -6.280670 | -0.531192 | -0.930469 |
| C | -5.704429 | -1.813822 | -0.942829 |
| H | -6.130078 | -3.926522 | -0.938449 |
| H | -8.586898 | -3.644653 | -0.913369 |
| H | -9.588579 | -1.362664 | -0.881599 |
| H | -8.089820 | 0.627087 | -0.888229 |
| H | -5.642360 | 0.347228 | -0.943039 |
| S | -3.940379 | -2.071641 | -0.998239 |
| H | -3.362030 | -0.676071 | -0.226319 |
| H | 1.118642 | -4.949870 | -1.774599 |
| H | -0.428988 | -4.244660 | -3.574079 |
| H | -0.017349 | -2.127380 | -4.813759 |
| C | 3.775291 | -1.108509 | 3.141611 |
| C | 2.161189 | 3.436781 | -0.491459 |
| C | 3.235819 | 4.309441 | -0.709169 |
| C | 0.866989 | 3.799360 | -0.908789 |
| C | 2.978848 | 5.517411 | -1.346149 |
| H | 4.241009 | 4.060872 | -0.403299 |
| C | 0.680068 | 5.024230 | -1.525999 |
| H | 0.021739 | 3.130560 | -0.788249 |
| C | 1.716208 | 5.918241 | -1.767419 |
| H | 1.552208 | 6.870101 | -2.256929 |
| F | 4.013738 | 6.356842 | -1.554119 |
| F | -0.571402 | 5.363820 | -1.915019 |
| C | 2.377551 | -1.323179 | 3.755441 |
| H | 1.886341 | -2.207879 | 3.341731 |
| H | 2.471001 | -1.457919 | 4.839631 |
| H | 1.723280 | -0.459829 | 3.590631 |
| C | 4.445810 | 0.103192 | 3.818841 |
| H | 5.450840 | 0.280372 | 3.419181 |
| H | 3.861780 | 1.016091 | 3.668441 |
| H | 4.534840 | -0.069508 | 4.897941 |
| C | 4.631051 | -2.364218 | 3.401471 |
| H | 4.217331 | -3.254138 | 2.912011 |
| H | 5.663561 | -2.228048 | 3.057701 |
| H | 4.673531 | -2.572878 | 4.476491 |

TS Conformation 11

B3LYP/6-31G(d) Energy = -3411.887626
M06-2X/def2tzvpp/IEFPCM(chloroform) Energy = -3411.895487
M06-2X/def2tzvpp/IEFPCM(chloroform)//B3LYP/6-31G(d) Quasiharmonic Free Energy = -3411.16973

Frequencies (Top 3 out of 291)

1. -985.6572 cm-1

2. 8.4448 cm-1
3. 10.9926 cm-1

B3LYP/6-31G(d) Molecular Geometry in Cartesian Coordinates

| | | | |
|---|---|---|---|
| C | -2.575308 | -1.460672 | 0.609774 |
| C | -2.628185 | -0.832348 | -0.798696 |
| H | -3.492968 | -0.175776 | -0.884966 |
| C | -1.467705 | 1.379681 | -1.132911 |
| N | -0.196084 | 1.897804 | -1.187550 |
| H | 0.558586 | 1.219441 | -1.289890 |
| N | -1.448851 | 0.029509 | -0.908289 |
| H | -0.605258 | -0.332898 | -0.470787 |
| C | -4.449758 | 0.062182 | 1.431623 |
| C | -3.340748 | -1.552080 | 2.921392 |
| C | -4.594623 | 0.498879 | 2.911751 |
| H | -4.010070 | 0.874566 | 0.846807 |
| C | -4.434956 | -0.809578 | 3.698444 |
| H | -3.439036 | -2.641572 | 2.949387 |
| H | -2.337773 | -1.305055 | 3.292293 |
| H | -5.544875 | 1.006462 | 3.095113 |
| H | -3.783100 | 1.192957 | 3.161522 |
| H | -5.370930 | -1.378284 | 3.683641 |
| H | -4.159569 | -0.649349 | 4.744960 |
| N | -3.479015 | -1.056636 | 1.537947 |
| O | -1.698340 | -2.288378 | 0.909638 |
| S | -2.880482 | 2.273156 | -1.371170 |
| C | -5.780890 | -0.323550 | 0.798915 |
| C | -6.508118 | 0.661347 | 0.115726 |
| C | -6.331496 | -1.606387 | 0.913720 |
| C | -7.761850 | 0.375985 | -0.425393 |
| H | -6.080292 | 1.653765 | -0.003438 |
| C | -7.586461 | -1.893570 | 0.373078 |
| C | -8.307297 | -0.902599 | -0.294918 |
| H | -8.307282 | 1.150619 | -0.957737 |
| O | 3.942046 | -3.129590 | 1.757365 |
| O | 0.742671 | -0.241596 | 1.123612 |
| C | 4.279444 | -1.969009 | 1.535527 |
| C | 3.361268 | -0.830154 | 1.313511 |
| O | 5.565690 | -1.594046 | 1.413548 |
| C | 6.537691 | -2.642552 | 1.574531 |
| H | 6.403923 | -3.408114 | 0.806203 |
| H | 7.505799 | -2.153867 | 1.466476 |
| H | 6.446183 | -3.105508 | 2.560552 |
| S | 1.655439 | -1.375156 | 1.414104 |
| C | 1.401304 | -2.733002 | 0.260711 |
| H | 0.323541 | -2.915425 | 0.284723 |
| H | 1.726703 | -2.380820 | -0.720577 |
| H | 1.998713 | -3.582609 | 0.587254 |
| C | 1.225198 | -2.085847 | 3.021059 |
| H | 1.902395 | -2.918121 | 3.218307 |
| H | 1.343803 | -1.286788 | 3.755927 |
| H | 0.180715 | -2.399401 | 2.942146 |
| C | 3.581702 | 0.441520 | 2.090136 |
| C | 4.216479 | 0.424054 | 3.345475 |
| C | 3.143228 | 1.673541 | 1.578986 |
| C | 4.424245 | 1.603756 | 4.056516 |
| H | 4.566532 | -0.517195 | 3.760956 |
| C | 3.341303 | 2.852395 | 2.297804 |
| H | 2.641962 | 1.713625 | 0.619972 |
| C | 3.984160 | 2.822815 | 3.535225 |
| H | 4.925875 | 1.570100 | 5.019977 |

| | | | |
|---|---|---|---|
| H | 2.995517 | 3.794036 | 1.881112 |
| H | 4.143582 | 3.742931 | 4.090823 |
| C | 2.568992 | -1.887080 | -3.458454 |
| C | 2.771202 | -3.100257 | -4.118300 |
| C | 3.859912 | -3.911384 | -3.791915 |
| C | 4.752919 | -3.489263 | -2.804517 |
| C | 4.563169 | -2.271448 | -2.150666 |
| C | 3.462093 | -1.452336 | -2.462153 |
| H | 1.719361 | -1.261542 | -3.717937 |
| H | 2.070744 | -3.412558 | -4.889113 |
| H | 4.013663 | -4.857290 | -4.304020 |
| H | 5.610940 | -4.106672 | -2.548318 |
| H | 5.277389 | -1.934821 | -1.404494 |
| S | 3.211683 | 0.105188 | -1.629826 |
| H | 3.374467 | -0.444223 | -0.077854 |
| H | -5.768702 | -2.393043 | 1.408087 |
| H | -7.997378 | -2.895209 | 0.469605 |
| H | -9.282002 | -1.127656 | -0.719291 |
| C | -2.746126 | -1.872446 | -1.969421 |
| C | 0.246154 | 3.237274 | -1.215060 |
| C | 1.488407 | 3.479431 | -1.826558 |
| C | -0.449297 | 4.289383 | -0.602415 |
| C | 1.998522 | 4.770145 | -1.817495 |
| H | 2.050348 | 2.682400 | -2.301554 |
| C | 0.111393 | 5.558146 | -0.638294 |
| H | -1.403541 | 4.136267 | -0.121259 |
| C | 1.334757 | 5.842626 | -1.233996 |
| H | 1.744985 | 6.844751 | -1.247553 |
| F | 3.187758 | 4.996700 | -2.409715 |
| F | -0.560151 | 6.568515 | -0.046770 |
| C | -3.956886 | -2.786963 | -1.701045 |
| H | -4.880830 | -2.211746 | -1.577703 |
| H | -4.100603 | -3.472617 | -2.544360 |
| H | -3.810731 | -3.397025 | -0.802010 |
| C | -2.990770 | -1.082907 | -3.271568 |
| H | -2.157509 | -0.408724 | -3.494042 |
| H | -3.097089 | -1.776756 | -4.114084 |
| H | -3.901310 | -0.477416 | -3.211267 |
| C | -1.486835 | -2.739332 | -2.145965 |
| H | -0.592615 | -2.124404 | -2.298818 |
| H | -1.323029 | -3.391182 | -1.284926 |
| H | -1.600216 | -3.372418 | -3.034215 |

TS Conformation 12

B3LYP/6-31G(d) Energy = -3411.892443
M06-2X/def2tzvpp/IEFPCM(chloroform) Energy = -3411.895127
M06-2X/def2tzvpp/IEFPCM(chloroform)//B3LYP/6-31G(d) Quasiharmonic Free Energy = -3411.169702

Frequencies (Top 3 out of 291)

1. -992.1045 cm-1
2. 7.5798 cm-1
3. 9.1472 cm-1

B3LYP/6-31G(d) Molecular Geometry in Cartesian Coordinates

| | | | |
|---|---|---|---|
| C | 2.706199 | -2.211130 | 0.779927 |
| C | 3.421313 | -1.150035 | 1.639772 |
| H | 4.442481 | -0.996109 | 1.294063 |
| C | 3.179319 | 1.109352 | 0.588786 |

| | | | |
|---|---|---|---|
| N | 2.187351 | 2.013299 | 0.297980 |
| H | 1.242652 | 1.706762 | 0.521141 |
| N | 2.726387 | 0.111885 | 1.405330 |
| H | 1.730137 | 0.092535 | 1.608217 |
| C | 2.741040 | -3.885379 | -1.003904 |
| C | 4.864756 | -2.766805 | -0.455081 |
| C | 3.932316 | -4.646876 | -1.635668 |
| H | 2.153816 | -4.539673 | -0.352027 |
| C | 5.026530 | -3.575850 | -1.749181 |
| H | 5.164900 | -1.721541 | -0.567904 |
| H | 5.449238 | -3.212301 | 0.360878 |
| H | 3.667838 | -5.101958 | -2.593587 |
| H | 4.254433 | -5.446024 | -0.956833 |
| H | 4.841264 | -2.934807 | -2.618044 |
| H | 6.031917 | -3.994675 | -1.849395 |
| N | 3.417955 | -2.874972 | -0.166690 |
| O | 1.494065 | -2.414283 | 0.926278 |
| S | 4.763341 | 1.202831 | 0.009921 |
| C | 1.791923 | -3.289131 | -2.040010 |
| C | 1.918522 | -1.980408 | -2.519184 |
| C | 0.771117 | -4.097828 | -2.559424 |
| C | 1.047351 | -1.491195 | -3.495669 |
| H | 2.686659 | -1.328000 | -2.114090 |
| C | -0.095726 | -3.616703 | -3.541468 |
| C | 0.040717 | -2.308001 | -4.014336 |
| H | 1.152785 | -0.467505 | -3.842639 |
| O | -0.299401 | 0.817359 | 1.505636 |
| O | -0.920202 | 0.846046 | -1.612783 |
| C | -1.459308 | 1.224215 | 1.611904 |
| C | -2.564820 | 0.897481 | 0.684841 |
| O | -1.881418 | 1.964963 | 2.639535 |
| C | -0.903155 | 2.275429 | 3.650323 |
| H | -0.061518 | 2.819393 | 3.214566 |
| H | -1.429507 | 2.897543 | 4.373359 |
| H | -0.542165 | 1.358778 | 4.122889 |
| S | -1.888281 | 0.098397 | -0.767209 |
| C | -1.252741 | -1.508229 | -0.260278 |
| H | -1.106093 | -2.086431 | -1.175620 |
| H | -0.302237 | -1.363945 | 0.254077 |
| H | -1.992240 | -1.959806 | 0.408800 |
| C | -3.345071 | -0.319149 | -1.744016 |
| H | -3.799790 | 0.618604 | -2.065443 |
| H | -2.975475 | -0.884341 | -2.602363 |
| H | -4.040511 | -0.908551 | -1.141874 |
| C | -3.554665 | 1.999968 | 0.368176 |
| C | -4.809891 | 1.997345 | 0.994193 |
| C | -3.231812 | 3.054998 | -0.503849 |
| C | -5.723918 | 3.022366 | 0.746712 |
| H | -5.055408 | 1.196456 | 1.684641 |
| C | -4.156148 | 4.067921 | -0.761472 |
| H | -2.257587 | 3.091035 | -0.982858 |
| C | -5.404284 | 4.054896 | -0.136211 |
| H | -6.689193 | 3.010727 | 1.245806 |
| H | -3.891338 | 4.873475 | -1.440799 |
| H | -6.119789 | 4.849365 | -0.330596 |
| C | -4.572227 | -3.581716 | 0.649776 |
| C | -5.296300 | -4.300231 | -0.302596 |
| C | -6.397154 | -3.723087 | -0.939371 |
| C | -6.773044 | -2.420989 | -0.601555 |
| C | -6.057376 | -1.703339 | 0.359044 |
| C | -4.934107 | -2.265803 | 0.996967 |
| H | -3.721106 | -4.039486 | 1.146206 |

| | | | |
|---|---|---|---|
| H | -4.997526 | -5.317016 | -0.547077 |
| H | -6.959960 | -4.282887 | -1.681490 |
| H | -7.636522 | -1.962683 | -1.078443 |
| H | -6.372677 | -0.699638 | 0.628956 |
| S | -4.005860 | -1.374048 | 2.238650 |
| H | -3.262459 | -0.155074 | 1.329655 |
| H | 0.652117 | -5.113974 | -2.188367 |
| H | -0.876758 | -4.262382 | -3.935271 |
| H | -0.628895 | -1.930249 | -4.782933 |
| C | 3.488116 | -1.519155 | 3.160845 |
| C | 2.235636 | 3.228302 | -0.414511 |
| C | 1.092663 | 3.557178 | -1.160992 |
| C | 3.307612 | 4.127003 | -0.336013 |
| C | 1.044440 | 4.783690 | -1.805068 |
| H | 0.278278 | 2.850190 | -1.273954 |
| C | 3.203658 | 5.330607 | -1.020624 |
| H | 4.199372 | 3.900273 | 0.230163 |
| C | 2.085825 | 5.701843 | -1.760947 |
| H | 2.034327 | 6.652618 | -2.276793 |
| F | -0.064781 | 5.092508 | -2.513109 |
| F | 4.232954 | 6.198642 | -0.945704 |
| C | 4.387582 | -0.480702 | 3.859542 |
| H | 4.450840 | -0.695846 | 4.932910 |
| H | 5.404845 | -0.490160 | 3.451290 |
| H | 3.990017 | 0.532156 | 3.739240 |
| C | 4.124436 | -2.916247 | 3.301130 |
| H | 4.256910 | -3.164799 | 4.360262 |
| H | 3.496244 | -3.696681 | 2.856602 |
| H | 5.113867 | -2.957629 | 2.828345 |
| C | 2.100181 | -1.523802 | 3.831349 |
| H | 1.637488 | -0.530257 | 3.799840 |
| H | 1.419375 | -2.229231 | 3.349711 |
| H | 2.202940 | -1.800472 | 4.887470 |

TS Conformation 13

B3LYP/6-31G(d) Energy = -3411.89846
M06-2X/def2tzvpp/IEFPCM(chloroform) Energy = -3411.896482
M06-2X/def2tzvpp/IEFPCM(chloroform)//B3LYP/6-31G(d) Quasiharmonic Free Energy = -3411.169298

Frequencies (Top 3 out of 291)

1. -990.1809 cm-1
2. 6.2298 cm-1
3. 11.0019 cm-1

B3LYP/6-31G(d) Molecular Geometry in Cartesian Coordinates

| | | | |
|---|---|---|---|
| C | 2.818912 | -1.408124 | 0.149214 |
| C | 3.211978 | -0.201957 | 1.028507 |
| H | 4.071586 | 0.318078 | 0.609277 |
| C | 2.059132 | 1.914010 | 0.284351 |
| N | 0.787558 | 2.434066 | 0.252104 |
| H | 0.061980 | 1.808665 | 0.595032 |
| N | 2.090369 | 0.729014 | 0.962582 |
| H | 1.198591 | 0.334522 | 1.251900 |
| C | 4.650456 | -0.901947 | -1.550584 |
| C | 3.097199 | -2.793008 | -1.835351 |
| C | 4.528530 | -1.287113 | -3.047137 |
| H | 4.416447 | 0.158593 | -1.425171 |
| C | 4.072013 | -2.752525 | -3.018550 |

| | | | |
|---|---|---|---|
| H | 3.078487 | -3.751133 | -1.307416 |
| H | 2.068413 | -2.575970 | -2.147819 |
| H | 5.467531 | -1.132262 | -3.584252 |
| H | 3.761029 | -0.659363 | -3.515443 |
| H | 4.925761 | -3.414603 | -2.839498 |
| H | 3.596590 | -3.069842 | -3.951106 |
| N | 3.568948 | -1.717942 | -0.936489 |
| O | 1.789000 | -2.057136 | 0.409932 |
| S | 3.420672 | 2.621919 | -0.422037 |
| C | 6.038356 | -1.165230 | -0.983101 |
| C | 6.457626 | -2.433224 | -0.559004 |
| C | 6.952453 | -0.103801 | -0.930715 |
| C | 7.762449 | -2.637705 | -0.107798 |
| H | 5.755762 | -3.262233 | -0.556819 |
| C | 8.257297 | -0.306025 | -0.478990 |
| C | 8.667717 | -1.575633 | -0.069146 |
| H | 8.069447 | -3.627516 | 0.219851 |
| O | -3.004327 | 0.876169 | -0.829407 |
| O | -0.803692 | 0.131309 | 1.406132 |
| C | -3.754956 | 0.407716 | 0.020895 |
| C | -3.420984 | -0.754389 | 0.886024 |
| O | -4.993658 | 0.865180 | 0.243304 |
| C | -5.425883 | 1.928042 | -0.633940 |
| H | -5.520184 | 1.548029 | -1.654350 |
| H | -6.396390 | 2.237464 | -0.246318 |
| H | -4.715964 | 2.757882 | -0.618664 |
| S | -1.651023 | -0.970088 | 0.842735 |
| C | -1.343579 | -2.487474 | 1.756869 |
| H | -0.268895 | -2.666433 | 1.669981 |
| H | -1.643740 | -2.319131 | 2.791652 |
| H | -1.930794 | -3.286225 | 1.295171 |
| C | -1.143712 | -1.414686 | -0.829613 |
| H | -1.816446 | -2.213949 | -1.159475 |
| H | -1.260041 | -0.521224 | -1.439861 |
| H | -0.104956 | -1.746231 | -0.739080 |
| C | -3.965166 | -0.781816 | 2.297237 |
| C | -4.836079 | -1.807200 | 2.695430 |
| C | -3.627427 | 0.221770 | 3.222571 |
| C | -5.357141 | -1.825825 | 3.990424 |
| H | -5.117219 | -2.576896 | 1.983265 |
| C | -4.142885 | 0.194452 | 4.517423 |
| H | -2.958168 | 1.023784 | 2.924587 |
| C | -5.009970 | -0.830104 | 4.904147 |
| H | -6.036863 | -2.621582 | 4.282745 |
| H | -3.872081 | 0.976501 | 5.221483 |
| H | -5.415807 | -0.848423 | 5.912092 |
| C | -4.323972 | -3.075892 | -3.282184 |
| C | -4.809210 | -2.529755 | -4.471092 |
| C | -5.817015 | -1.563973 | -4.447033 |
| C | -6.342283 | -1.157268 | -3.218611 |
| C | -5.867165 | -1.707947 | -2.027172 |
| C | -4.843243 | -2.671440 | -2.040985 |
| H | -3.539634 | -3.827276 | -3.308069 |
| H | -4.394649 | -2.860152 | -5.420676 |
| H | -6.192738 | -1.138243 | -5.373508 |
| H | -7.139103 | -0.417388 | -3.186154 |
| H | -6.294562 | -1.399886 | -1.077486 |
| S | -4.236375 | -3.397994 | -0.523938 |
| H | -3.843937 | -1.953198 | 0.222569 |
| H | 6.634557 | 0.889923 | -1.237753 |
| H | 8.949456 | 0.530845 | -0.440247 |
| H | 9.682233 | -1.734746 | 0.286007 |

| | | | |
|---|---|---|---|
| C | 3.574170 | -0.572766 | 2.511350 |
| C | 0.267621 | 3.650002 | -0.234049 |
| C | 1.008735 | 4.830507 | -0.376826 |
| C | -1.110870 | 3.646565 | -0.515559 |
| C | 0.343754 | 5.972055 | -0.807441 |
| H | 2.067708 | 4.866232 | -0.169205 |
| C | -1.712700 | 4.823672 | -0.926474 |
| H | -1.703151 | 2.739985 | -0.448789 |
| C | -1.016980 | 6.015554 | -1.088709 |
| H | -1.503895 | 6.925247 | -1.417247 |
| F | 1.057279 | 7.108266 | -0.943946 |
| F | -3.041429 | 4.809979 | -1.185873 |
| C | 4.700933 | -1.622218 | 2.510129 |
| H | 5.585376 | -1.267651 | 1.971572 |
| H | 5.002791 | -1.844466 | 3.540313 |
| H | 4.379085 | -2.564553 | 2.051649 |
| C | 2.374241 | -1.127239 | 3.304148 |
| H | 2.013618 | -2.067529 | 2.880085 |
| H | 2.679147 | -1.312735 | 4.340975 |
| H | 1.538986 | -0.418360 | 3.334335 |
| C | 4.082028 | 0.711101 | 3.198119 |
| H | 3.314406 | 1.491379 | 3.204594 |
| H | 4.356968 | 0.497297 | 4.237735 |
| H | 4.965257 | 1.114080 | 2.690042 |

TS Conformation 14

B3LYP/6-31G(d) Energy = -3411.893048
M06-2X/def2tzvpp/IEFPCM(chloroform) Energy = -3411.899811
M06-2X/def2tzvpp/IEFPCM(chloroform)//B3LYP/6-31G(d) Quasiharmonic Free Energy = -3411.169231

Frequencies (Top 3 out of 291)

1. -22.8454 cm-1
2. 6.3571 cm-1
3. 9.7245 cm-1

B3LYP/6-31G(d) Molecular Geometry in Cartesian Coordinates

| | | | |
|---|---|---|---|
| C | 2.726129 | -1.486590 | 0.408644 |
| C | 3.142555 | -0.157191 | 1.068483 |
| H | 4.008507 | 0.268575 | 0.564617 |
| C | 2.067593 | 1.882027 | 0.042236 |
| N | 0.808215 | 2.395586 | -0.139973 |
| H | 0.055277 | 1.796786 | 0.186712 |
| N | 2.035190 | 0.764289 | 0.831950 |
| H | 1.122027 | 0.407403 | 1.099430 |
| C | 4.486748 | -1.233650 | -1.414845 |
| C | 2.940524 | -3.151326 | -1.360256 |
| C | 4.332823 | -1.852791 | -2.827639 |
| H | 4.245472 | -0.168105 | -1.452762 |
| C | 3.890422 | -3.297143 | -2.555004 |
| H | 2.933572 | -4.014851 | -0.688553 |
| H | 1.906511 | -2.981813 | -1.684891 |
| H | 5.257855 | -1.780659 | -3.404779 |
| H | 3.549022 | -1.312523 | -3.371614 |
| H | 4.753178 | -3.917421 | -2.289770 |
| H | 3.398454 | -3.761936 | -3.414095 |
| N | 3.430426 | -1.950727 | -0.650056 |
| O | 1.714080 | -2.093115 | 0.810489 |
| S | 3.493684 | 2.510913 | -0.607048 |

| | | | |
|---|---|---|---|
| C | 5.890022 | -1.393831 | -0.846548 |
| C | 6.798007 | -0.336419 | -0.996327 |
| C | 6.327325 | -2.571922 | -0.226769 |
| C | 8.114914 | -0.457791 | -0.551029 |
| H | 6.465775 | 0.591503 | -1.455759 |
| C | 7.644439 | -2.695285 | 0.218546 |
| C | 8.543533 | -1.639965 | 0.054992 |
| H | 8.802404 | 0.375197 | -0.670670 |
| O | -3.723794 | -3.198735 | 1.107157 |
| O | -0.951011 | 0.170988 | 1.061419 |
| C | -4.251718 | -2.098360 | 1.180432 |
| C | -3.517057 | -0.793572 | 0.993320 |
| O | -5.550127 | -1.892444 | 1.385237 |
| C | -6.383362 | -3.073564 | 1.379129 |
| H | -6.381074 | -3.510974 | 0.378481 |
| H | -6.019780 | -3.799893 | 2.109039 |
| H | -7.378448 | -2.718364 | 1.644431 |
| S | -1.677888 | -1.125350 | 1.007862 |
| C | -1.193817 | -2.134450 | 2.419630 |
| H | -1.764135 | -3.062576 | 2.393915 |
| H | -0.118912 | -2.293547 | 2.293596 |
| H | -1.404690 | -1.546134 | 3.315071 |
| C | -1.269529 | -2.084079 | -0.452435 |
| H | -0.190009 | -2.252690 | -0.394758 |
| H | -1.850070 | -3.006812 | -0.409155 |
| H | -1.589253 | -1.451330 | -1.298555 |
| C | -3.847561 | 0.365599 | 1.891127 |
| C | -4.010364 | 0.209086 | 3.276502 |
| C | -3.985408 | 1.637424 | 1.317680 |
| C | -4.301532 | 1.311709 | 4.076630 |
| H | -3.934217 | -0.777960 | 3.727890 |
| C | -4.278508 | 2.738451 | 2.122229 |
| H | -3.891115 | 1.744146 | 0.239931 |
| C | -4.430989 | 2.578710 | 3.500335 |
| H | -4.432376 | 1.183090 | 5.147423 |
| H | -4.386681 | 3.718813 | 1.667236 |
| H | -4.657554 | 3.437435 | 4.126322 |
| C | -5.728413 | -1.397995 | -2.070303 |
| C | -6.521218 | -2.509492 | -2.363686 |
| C | -6.014699 | -3.566180 | -3.125262 |
| C | -4.701960 | -3.485200 | -3.597752 |
| C | -3.909437 | -2.375000 | -3.308989 |
| C | -4.397568 | -1.300446 | -2.533056 |
| H | -6.141137 | -0.575860 | -1.491771 |
| H | -7.550154 | -2.540279 | -2.008630 |
| H | -6.634461 | -4.428251 | -3.357962 |
| H | -4.292608 | -4.291906 | -4.202835 |
| H | -2.896381 | -2.317199 | -3.698992 |
| S | -3.382675 | 0.106972 | -2.157868 |
| H | -3.606213 | -0.460023 | -0.107617 |
| H | 5.631067 | -3.390606 | -0.068834 |
| H | 7.965957 | -3.615666 | 0.699021 |
| H | 9.567710 | -1.735242 | 0.405215 |
| C | 3.515254 | -0.270440 | 2.589868 |
| C | 0.325251 | 3.595899 | -0.696596 |
| C | 1.116523 | 4.700662 | -1.038032 |
| C | -1.072191 | 3.660952 | -0.851364 |
| C | 0.479803 | 5.834511 | -1.528016 |
| H | 2.190889 | 4.684151 | -0.936652 |
| C | -1.643767 | 4.826725 | -1.333200 |
| H | -1.708542 | 2.815584 | -0.611837 |
| C | -0.895580 | 5.943427 | -1.688051 |

| | | | |
|---|---|---|---|
| H | -1.358281 | 6.844957 | -2.069611 |
| F | 1.244677 | 6.895969 | -1.855718 |
| F | -2.985147 | 4.881398 | -1.454562 |
| C | 4.621235 | -1.328538 | 2.760215 |
| H | 5.506043 | -1.090194 | 2.161377 |
| H | 4.931473 | -1.377496 | 3.810577 |
| H | 4.275060 | -2.328351 | 2.472514 |
| C | 4.055781 | 1.102307 | 3.038629 |
| H | 3.302186 | 1.887215 | 2.920181 |
| H | 4.342702 | 1.064095 | 4.096256 |
| H | 4.936223 | 1.395992 | 2.457259 |
| C | 2.316174 | -0.649456 | 3.479591 |
| H | 1.485551 | 0.056664 | 3.363740 |
| H | 1.954629 | -1.656163 | 3.256383 |
| H | 2.620591 | -0.622118 | 4.532668 |

TS Conformation 15

B3LYP/6-31G(d) Energy = -3411.891378
M06-2X/def2tzvpp/IEFPCM(chloroform) Energy = -3411.894894
M06-2X/def2tzvpp/IEFPCM(chloroform)//B3LYP/6-31G(d) Quasiharmonic Free Energy = -3411.169056

Frequencies (Top 3 out of 291)

1. -983.1389 cm-1
2. 8.2282 cm-1
3. 10.4468 cm-1

B3LYP/6-31G(d) Molecular Geometry in Cartesian Coordinates

| | | | |
|---|---|---|---|
| C | -3.201232 | -1.457799 | -0.231805 |
| C | -3.083780 | -0.946257 | -1.684468 |
| H | -3.967559 | -0.372811 | -1.961286 |
| C | -2.121141 | 1.367864 | -1.665111 |
| N | -0.907905 | 1.984578 | -1.481408 |
| H | -0.132255 | 1.339218 | -1.344229 |
| N | -1.979889 | 0.006934 | -1.697044 |
| H | -1.083945 | -0.376567 | -1.408551 |
| C | -4.466513 | -1.647787 | 1.864922 |
| C | -5.663096 | -0.827709 | -0.118348 |
| C | -5.951766 | -1.357814 | 2.202020 |
| H | -4.259353 | -2.722489 | 1.941058 |
| C | -6.675457 | -1.445630 | 0.853030 |
| H | -5.728971 | 0.266965 | -0.137995 |
| H | -5.792446 | -1.192135 | -1.138219 |
| H | -6.039259 | -0.345017 | 2.611153 |
| H | -6.336096 | -2.052580 | 2.954256 |
| H | -7.634181 | -0.918723 | 0.843930 |
| H | -6.860956 | -2.492102 | 0.582495 |
| N | -4.364994 | -1.257457 | 0.439368 |
| O | -2.224213 | -1.974531 | 0.323639 |
| S | -3.607080 | 2.144996 | -1.865611 |
| C | -3.512328 | -0.914018 | 2.794413 |
| C | -3.035688 | -1.565580 | 3.938012 |
| C | -3.161794 | 0.426061 | 2.586195 |
| C | -2.237550 | -0.891374 | 4.865079 |
| H | -3.291214 | -2.609718 | 4.105582 |
| C | -2.351092 | 1.098602 | 3.501286 |
| C | -1.893061 | 0.445423 | 4.649289 |
| H | -1.886909 | -1.411133 | 5.753296 |
| O | 1.008413 | -0.316685 | -0.968831 |

| | | | |
|---|---|---|---|
| O | 0.744244 | 1.475814 | 1.750447 |
| C | 2.212362 | -0.243987 | -0.694369 |
| C | 2.774350 | -0.165563 | 0.668445 |
| O | 3.173797 | -0.276404 | -1.620381 |
| C | 2.759740 | -0.529193 | -2.976613 |
| H | 2.014546 | 0.202555 | -3.297667 |
| H | 3.668090 | -0.437945 | -3.571280 |
| H | 2.351444 | -1.539593 | -3.055064 |
| S | 1.470904 | 0.185634 | 1.843734 |
| C | 0.360945 | -1.230622 | 1.901559 |
| H | -0.269777 | -1.226331 | 1.013169 |
| H | 0.984978 | -2.130157 | 1.948844 |
| H | -0.252798 | -1.103591 | 2.796482 |
| C | 2.333046 | 0.059163 | 3.424765 |
| H | 2.820380 | -0.917056 | 3.489675 |
| H | 3.064631 | 0.866399 | 3.469791 |
| H | 1.571719 | 0.178676 | 4.198394 |
| C | 3.988092 | 0.720559 | 0.868387 |
| C | 3.892331 | 2.114604 | 0.711389 |
| C | 5.233993 | 0.156978 | 1.180292 |
| C | 5.016199 | 2.926001 | 0.859452 |
| H | 2.935423 | 2.569818 | 0.478990 |
| C | 6.357688 | 0.972791 | 1.324808 |
| H | 5.319202 | -0.919268 | 1.295628 |
| C | 6.253355 | 2.355677 | 1.165985 |
| H | 4.918169 | 3.998754 | 0.719629 |
| H | 7.318449 | 0.522427 | 1.559655 |
| H | 7.132165 | 2.985596 | 1.275902 |
| C | 4.625912 | -4.311624 | -0.722712 |
| C | 4.575639 | -4.899505 | -1.986609 |
| C | 3.371438 | -4.961735 | -2.691512 |
| C | 2.214786 | -4.435483 | -2.112355 |
| C | 2.259666 | -3.854580 | -0.842550 |
| C | 3.468968 | -3.777456 | -0.129950 |
| H | 5.565770 | -4.261160 | -0.181078 |
| H | 5.483734 | -5.308600 | -2.423316 |
| H | 3.333506 | -5.421133 | -3.675761 |
| H | 1.265438 | -4.490573 | -2.640468 |
| H | 1.347185 | -3.469898 | -0.396473 |
| S | 3.557555 | -3.051789 | 1.502273 |
| H | 3.129885 | -1.500877 | 1.036699 |
| H | -3.508897 | 0.943055 | 1.695259 |
| H | -2.072974 | 2.131452 | 3.313654 |
| H | -1.278491 | 0.976919 | 5.371832 |
| C | -2.889725 | -2.081877 | -2.746103 |
| C | -0.510354 | 3.327043 | -1.374507 |
| C | -1.368589 | 4.434091 | -1.317119 |
| C | 0.882089 | 3.529722 | -1.298944 |
| C | -0.805300 | 5.696371 | -1.171785 |
| H | -2.440124 | 4.321576 | -1.380554 |
| C | 1.376370 | 4.811536 | -1.135754 |
| H | 1.575858 | 2.699821 | -1.363524 |
| C | 0.560213 | 5.933814 | -1.070506 |
| H | 0.961847 | 6.931708 | -0.947383 |
| F | -1.638107 | 6.755104 | -1.114160 |
| F | 2.716128 | 4.972524 | -1.030562 |
| C | -1.555497 | -2.835176 | -2.572027 |
| H | -1.487118 | -3.313048 | -1.591909 |
| H | -1.469941 | -3.608254 | -3.345511 |
| H | -0.694326 | -2.166541 | -2.683434 |
| C | -2.931034 | -1.436423 | -4.145259 |
| H | -2.131220 | -0.698996 | -4.265760 |

| | | | |
|---|---|---|---|
| H | -2.806096 | -2.202594 | -4.919641 |
| H | -3.884834 | -0.926508 | -4.324410 |
| C | -4.052472 | -3.086267 | -2.620076 |
| H | -5.024080 | -2.602529 | -2.780332 |
| H | -3.953081 | -3.872769 | -3.376789 |
| H | -4.066453 | -3.575335 | -1.639077 |

TS Conformation 16

B3LYP/6-31G(d) Energy = -3411.891029
M06-2X/def2tzvpp/IEFPCM(chloroform) Energy = -3411.895545
M06-2X/def2tzvpp/IEFPCM(chloroform)//B3LYP/6-31G(d) Quasiharmonic Free Energy = -3411.16893

Frequencies (Top 3 out of 291)

1. -716.1669 cm-1
2. 7.2800 cm-1
3. 13.0879 cm-1

B3LYP/6-31G(d) Molecular Geometry in Cartesian Coordinates

| | | | |
|---|---|---|---|
| C | -2.704717 | -1.309260 | 0.407028 |
| C | -2.928150 | -0.621985 | -0.956418 |
| H | -3.767444 | 0.068916 | -0.901318 |
| C | -1.682275 | 1.555490 | -1.083020 |
| N | -0.395680 | 2.021269 | -1.194201 |
| H | 0.329875 | 1.308720 | -1.193021 |
| N | -1.744086 | 0.195814 | -1.203159 |
| H | -0.863958 | -0.314112 | -1.211236 |
| C | -4.648807 | -0.065328 | 1.493209 |
| C | -3.238385 | -1.639376 | 2.757765 |
| C | -4.662301 | 0.284460 | 3.002887 |
| H | -4.348272 | 0.813735 | 0.917219 |
| C | -4.293145 | -1.035832 | 3.693583 |
| H | -3.245649 | -2.733218 | 2.728160 |
| H | -2.222598 | -1.330280 | 3.032428 |
| H | -5.627991 | 0.687193 | 3.318282 |
| H | -3.895883 | 1.043751 | 3.198628 |
| H | -5.169706 | -1.688832 | 3.761501 |
| H | -3.906475 | -0.893742 | 4.706836 |
| N | -3.567608 | -1.084577 | 1.426644 |
| O | -1.701110 | -2.025513 | 0.584181 |
| S | -3.041477 | 2.528259 | -0.828496 |
| C | -6.000351 | -0.547478 | 0.985532 |
| C | -6.855016 | 0.380555 | 0.374534 |
| C | -6.447430 | -1.865620 | 1.146896 |
| C | -8.129229 | 0.005223 | -0.053389 |
| H | -6.514358 | 1.402735 | 0.226222 |
| C | -7.721632 | -2.242916 | 0.720343 |
| C | -8.568120 | -1.308272 | 0.121345 |
| H | -8.774782 | 0.738058 | -0.530005 |
| O | 3.558312 | -3.321329 | 1.486593 |
| O | 1.144793 | -0.770442 | -1.098161 |
| C | 4.162172 | -2.729736 | 0.595267 |
| C | 3.565205 | -1.738941 | -0.337783 |
| O | 5.482785 | -2.843632 | 0.400075 |
| C | 6.182656 | -3.705455 | 1.317242 |
| H | 6.081150 | -3.335697 | 2.340271 |
| H | 7.224187 | -3.673309 | 0.999068 |
| H | 5.791087 | -4.724419 | 1.261744 |
| S | 1.768448 | -1.699141 | -0.113329 |

| | | | |
|---|---|---|---|
| C | 1.040287 | -3.342396 | -0.282485 |
| H | -0.028009 | -3.220470 | -0.084641 |
| H | 1.536599 | -3.998281 | 0.433432 |
| H | 1.227520 | -3.651585 | -1.313128 |
| C | 1.391842 | -1.187367 | 1.570561 |
| H | 0.301312 | -1.220650 | 1.644926 |
| H | 1.882214 | -1.880089 | 2.253762 |
| H | 1.792179 | -0.177090 | 1.685352 |
| C | 3.909429 | -1.810090 | -1.802500 |
| C | 3.905478 | -0.639062 | -2.576075 |
| C | 4.220056 | -3.032365 | -2.423332 |
| C | 4.213734 | -0.691069 | -3.935481 |
| H | 3.664570 | 0.310041 | -2.109976 |
| C | 4.534673 | -3.079875 | -3.779530 |
| H | 4.237548 | -3.948050 | -1.838057 |
| C | 4.529520 | -1.908006 | -4.540026 |
| H | 4.211572 | 0.224557 | -4.519953 |
| H | 4.781922 | -4.031285 | -4.242572 |
| H | 4.773587 | -1.944921 | -5.598230 |
| C | 4.522339 | 0.068265 | 3.039653 |
| C | 4.279771 | 0.091627 | 4.413795 |
| C | 3.646429 | 1.187814 | 5.003230 |
| C | 3.265336 | 2.264473 | 4.197940 |
| C | 3.494094 | 2.240620 | 2.822278 |
| C | 4.121531 | 1.135568 | 2.211702 |
| H | 5.051559 | -0.772364 | 2.598983 |
| H | 4.599585 | -0.747565 | 5.028072 |
| H | 3.463599 | 1.208385 | 6.074333 |
| H | 2.784035 | 3.132463 | 4.643071 |
| H | 3.206916 | 3.087656 | 2.206003 |
| S | 4.400196 | 1.123458 | 0.454108 |
| H | 3.935575 | -0.523659 | 0.085830 |
| H | -5.789200 | -2.609937 | 1.585870 |
| H | -8.050463 | -3.270605 | 0.851143 |
| H | -9.558568 | -1.603578 | -0.214055 |
| C | -3.221848 | -1.601349 | -2.148164 |
| C | 0.113040 | 3.338161 | -1.090124 |
| C | -0.563169 | 4.462386 | -1.581220 |
| C | 1.385075 | 3.473093 | -0.512696 |
| C | 0.045240 | 5.703226 | -1.445693 |
| H | -1.535848 | 4.385916 | -2.043293 |
| C | 1.935884 | 4.742141 | -0.407514 |
| H | 1.953485 | 2.621007 | -0.149124 |
| C | 1.291854 | 5.887444 | -0.859900 |
| H | 1.737551 | 6.869540 | -0.761460 |
| F | -0.606112 | 6.787143 | -1.915896 |
| F | 3.141052 | 4.872991 | 0.183159 |
| C | -2.038811 | -2.532600 | -2.474745 |
| H | -1.832348 | -3.221260 | -1.651683 |
| H | -2.280303 | -3.128179 | -3.363221 |
| H | -1.123239 | -1.973545 | -2.698759 |
| C | -3.539237 | -0.738586 | -3.386655 |
| H | -4.397853 | -0.082028 | -3.206986 |
| H | -2.688323 | -0.108770 | -3.664318 |
| H | -3.777952 | -1.382592 | -4.241231 |
| C | -4.453126 | -2.460247 | -1.806263 |
| H | -4.273168 | -3.104443 | -0.937700 |
| H | -5.331580 | -1.843272 | -1.592967 |
| H | -4.698781 | -3.111501 | -2.653247 |

TS Conformation 17

B3LYP/6-31G(d) Energy = -3411.891492
M06-2X/def2tzvpp/IEFPCM(chloroform) Energy = -3411.895116
M06-2X/def2tzvpp/IEFPCM(chloroform)//B3LYP/6-31G(d) Quasiharmonic Free Energy = -3411.168906

Frequencies (Top 3 out of 291)

1. -921.2525 cm-1
2. 6.6740 cm-1
3. 10.2773 cm-1

B3LYP/6-31G(d) Molecular Geometry in Cartesian Coordinates

| | | | |
|---|---|---|---|
| C | 2.739737 | -1.497859 | 0.034481 |
| C | 3.060963 | -0.333943 | 0.993881 |
| H | 3.911700 | 0.238721 | 0.628809 |
| C | 1.893235 | 1.799964 | 0.356359 |
| N | 0.624337 | 2.321075 | 0.304826 |
| H | -0.129549 | 1.675155 | 0.524775 |
| N | 1.913520 | 0.566579 | 0.941530 |
| H | 1.019190 | 0.146014 | 1.182309 |
| C | 4.592239 | -0.818797 | -1.581051 |
| C | 3.112759 | -2.737159 | -2.028110 |
| C | 4.516646 | -1.105702 | -3.102333 |
| H | 4.321068 | 0.223041 | -1.390685 |
| C | 4.110560 | -2.583967 | -3.182684 |
| H | 3.118388 | -3.728351 | -1.564722 |
| H | 2.083294 | -2.536257 | -2.349206 |
| H | 5.461384 | -0.884295 | -3.604933 |
| H | 3.738631 | -0.473156 | -3.546101 |
| H | 4.982770 | -3.227963 | -3.028227 |
| H | 3.666346 | -2.852945 | -4.145358 |
| N | 3.524435 | -1.707903 | -1.049740 |
| O | 1.731986 | -2.204550 | 0.225332 |
| S | 3.273214 | 2.573817 | -0.239857 |
| C | 5.974165 | -1.077943 | -0.997362 |
| C | 6.854000 | 0.004061 | -0.855334 |
| C | 6.421776 | -2.357673 | -0.643289 |
| C | 8.153444 | -0.188831 | -0.384373 |
| H | 6.512943 | 1.005645 | -1.106676 |
| C | 7.721230 | -2.552690 | -0.172811 |
| C | 8.592599 | -1.469525 | -0.044711 |
| H | 8.818678 | 0.663547 | -0.275590 |
| O | -3.618803 | -3.414228 | 0.034601 |
| O | -0.996538 | -0.188611 | 1.291684 |
| C | -4.166738 | -2.456233 | 0.575699 |
| C | -3.485483 | -1.203175 | 0.976789 |
| O | -5.486492 | -2.404008 | 0.814673 |
| C | -6.249795 | -3.521548 | 0.321849 |
| H | -6.192226 | -3.559386 | -0.768777 |
| H | -5.875027 | -4.459030 | 0.739594 |
| H | -7.272621 | -3.334056 | 0.648394 |
| S | -1.700827 | -1.408757 | 0.801919 |
| C | -1.076430 | -2.854801 | 1.685889 |
| H | -1.633165 | -3.723722 | 1.333514 |
| H | -0.009035 | -2.909445 | 1.455511 |
| H | -1.250409 | -2.662965 | 2.746854 |
| C | -1.314173 | -1.717773 | -0.928545 |
| H | -1.650069 | -0.824773 | -1.464334 |
| H | -0.230311 | -1.859224 | -0.963265 |
| H | -1.868368 | -2.601309 | -1.244224 |
| C | -3.829937 | -0.552961 | 2.289040 |

| | | | |
|---|---|---|---|
| C | -3.766564 | 0.842849 | 2.418586 |
| C | -4.200624 | -1.318941 | 3.408226 |
| C | -4.070974 | 1.455389 | 3.634172 |
| H | -3.490077 | 1.449943 | 1.564130 |
| C | -4.512680 | -0.704911 | 4.619078 |
| H | -4.266586 | -2.400520 | 3.324151 |
| C | -4.444903 | 0.685471 | 4.735828 |
| H | -4.020786 | 2.537445 | 3.715917 |
| H | -4.806270 | -1.311599 | 5.471453 |
| H | -4.686010 | 1.165058 | 5.680612 |
| C | -5.699864 | -0.864425 | -2.341942 |
| C | -6.192472 | -1.704056 | -3.342047 |
| C | -5.493193 | -1.866955 | -4.540416 |
| C | -4.295130 | -1.175212 | -4.728047 |
| C | -3.794907 | -0.340809 | -3.727305 |
| C | -4.485590 | -0.173697 | -2.513639 |
| H | -6.259310 | -0.724083 | -1.421685 |
| H | -7.138117 | -2.220630 | -3.190724 |
| H | -5.881964 | -2.516060 | -5.320479 |
| H | -3.744671 | -1.283310 | -5.659933 |
| H | -2.866223 | 0.201102 | -3.884725 |
| S | -3.855584 | 0.908830 | -1.242696 |
| H | -3.710616 | -0.266538 | -0.026957 |
| H | 5.745773 | -3.205217 | -0.711312 |
| H | 8.050504 | -3.552052 | 0.099651 |
| H | 9.602891 | -1.621465 | 0.325333 |
| C | 3.405270 | -0.769936 | 2.462622 |
| C | 0.179532 | 3.597288 | -0.114464 |
| C | 0.899443 | 4.770664 | 0.144615 |
| C | -1.075477 | 3.657849 | -0.739488 |
| C | 0.346581 | 5.979808 | -0.253751 |
| H | 1.863197 | 4.751516 | 0.631878 |
| C | -1.578830 | 4.901069 | -1.099968 |
| H | -1.651174 | 2.764657 | -0.966096 |
| C | -0.891242 | 6.088029 | -0.876239 |
| H | -1.298580 | 7.046645 | -1.172957 |
| F | 1.038532 | 7.111272 | -0.005972 |
| F | -2.780594 | 4.960586 | -1.702919 |
| C | 2.220340 | -1.428309 | 3.194672 |
| H | 1.940749 | -2.377409 | 2.730917 |
| H | 2.502133 | -1.628023 | 4.235438 |
| H | 1.338345 | -0.778053 | 3.216983 |
| C | 4.585264 | -1.758853 | 2.431182 |
| H | 5.463716 | -1.326776 | 1.941604 |
| H | 4.871118 | -2.029780 | 3.454302 |
| H | 4.325962 | -2.685647 | 1.905999 |
| C | 3.829967 | 0.496644 | 3.233359 |
| H | 4.692150 | 0.981633 | 2.762645 |
| H | 3.017693 | 1.228883 | 3.277751 |
| H | 4.106530 | 0.234605 | 4.261466 |

TS Conformation 18

B3LYP/6-31G(d) Energy = -3411.893112
M06-2X/def2tzvpp/IEFPCM(chloroform) Energy = -3411.895329
M06-2X/def2tzvpp/IEFPCM(chloroform)//B3LYP/6-31G(d) Quasiharmonic Free Energy = -3411.16886

Frequencies (Top 3 out of 291)

1. -986.5303 cm-1
2. 8.2296 cm-1

3. 12.9247 cm-1

B3LYP/6-31G(d) Molecular Geometry in Cartesian Coordinates

| | | | |
|---|---|---|---|
| C | -3.226715 | -1.090607 | -0.342327 |
| C | -3.072285 | -0.196101 | -1.590345 |
| H | -3.908689 | 0.497524 | -1.669956 |
| C | -1.951172 | 1.986137 | -1.093517 |
| N | -0.756507 | 2.443313 | -0.574172 |
| H | -0.202340 | 1.746609 | -0.080610 |
| N | -1.906796 | 0.647059 | -1.340958 |
| H | -1.017490 | 0.167606 | -1.218482 |
| C | -4.456111 | -1.705040 | 1.691602 |
| C | -5.582289 | -0.223663 | 0.084373 |
| C | -5.870285 | -1.324798 | 2.196926 |
| H | -4.411982 | -2.774193 | 1.448612 |
| C | -6.640058 | -0.949166 | 0.925089 |
| H | -5.507423 | 0.838117 | 0.350158 |
| H | -5.788545 | -0.289180 | -0.984963 |
| H | -5.797051 | -0.458069 | 2.863690 |
| H | -6.327617 | -2.140494 | 2.764199 |
| H | -7.515582 | -0.323024 | 1.120472 |
| H | -6.978617 | -1.850700 | 0.400513 |
| N | -4.335791 | -0.937014 | 0.430583 |
| O | -2.326763 | -1.869485 | -0.009815 |
| S | -3.310959 | 2.949939 | -1.356052 |
| C | -3.373332 | -1.408624 | 2.717211 |
| C | -2.883371 | -0.111540 | 2.914278 |
| C | -2.906563 | -2.437094 | 3.544214 |
| C | -1.949998 | 0.151735 | 3.917325 |
| H | -3.221818 | 0.695678 | 2.270095 |
| C | -1.980874 | -2.176934 | 4.557012 |
| C | -1.500561 | -0.878638 | 4.748559 |
| H | -1.574577 | 1.162614 | 4.048152 |
| O | 0.992923 | -0.457590 | -1.274706 |
| O | 0.680021 | 0.777048 | 1.607573 |
| C | 2.200496 | -0.406669 | -1.042407 |
| C | 2.810682 | -0.547244 | 0.298704 |
| O | 3.126516 | -0.254289 | -1.997236 |
| C | 2.633844 | -0.200293 | -3.349564 |
| H | 3.508904 | 0.012882 | -3.963244 |
| H | 1.886247 | 0.589090 | -3.454842 |
| H | 2.194900 | -1.162682 | -3.624656 |
| S | 1.491990 | -0.472500 | 1.507325 |
| C | 0.451756 | -1.933023 | 1.336354 |
| H | -0.197010 | -1.798966 | 0.471020 |
| H | -0.149169 | -1.987203 | 2.247404 |
| H | 1.122392 | -2.791555 | 1.230994 |
| C | 2.338313 | -0.777158 | 3.068022 |
| H | 2.857153 | -1.737155 | 2.993243 |
| H | 1.561468 | -0.797462 | 3.835230 |
| H | 3.038197 | 0.041570 | 3.237266 |
| C | 3.957951 | 0.375958 | 0.652005 |
| C | 5.212574 | -0.140569 | 1.008893 |
| C | 3.778991 | 1.769750 | 0.635823 |
| C | 6.256307 | 0.722681 | 1.349027 |
| H | 5.373937 | -1.214032 | 1.001130 |
| C | 4.815837 | 2.629487 | 0.993804 |
| H | 2.821741 | 2.185716 | 0.340023 |
| C | 6.059645 | 2.104905 | 1.352347 |
| H | 7.225690 | 0.310077 | 1.615324 |
| H | 4.639968 | 3.701684 | 0.997536 |

| | | | |
|---|---|---|---|
| H | 6.872875 | 2.770667 | 1.628806 |
| C | 2.161951 | -5.245126 | -0.599079 |
| C | 1.635982 | -5.829150 | -1.752282 |
| C | 2.010233 | -5.364886 | -3.014430 |
| C | 2.925628 | -4.314440 | -3.111150 |
| C | 3.460969 | -3.732884 | -1.960774 |
| C | 3.080187 | -4.185396 | -0.685138 |
| H | 1.866532 | -5.611562 | 0.380077 |
| H | 0.927895 | -6.649507 | -1.661842 |
| H | 1.598971 | -5.819775 | -3.911535 |
| H | 3.238437 | -3.954336 | -4.088922 |
| H | 4.186210 | -2.928910 | -2.043011 |
| S | 3.769612 | -3.456580 | 0.795508 |
| H | 3.269830 | -1.899826 | 0.474179 |
| H | -3.269885 | -3.451306 | 3.393723 |
| H | -1.638360 | -2.987006 | 5.195930 |
| H | -0.792227 | -0.669170 | 5.546740 |
| C | -2.987551 | -0.987305 | -2.939049 |
| C | -0.242878 | 3.747305 | -0.482683 |
| C | -0.580296 | 4.777256 | -1.372925 |
| C | 0.731757 | 3.972892 | 0.506454 |
| C | 0.068583 | 5.997467 | -1.244927 |
| H | -1.328562 | 4.643104 | -2.140367 |
| C | 1.363758 | 5.205846 | 0.559020 |
| H | 0.985860 | 3.203344 | 1.225804 |
| C | 1.055552 | 6.253470 | -0.298074 |
| H | 1.552916 | 7.213225 | -0.235907 |
| F | -0.257182 | 6.984466 | -2.103726 |
| F | 2.330506 | 5.390704 | 1.488506 |
| C | -4.262363 | -1.844049 | -3.078237 |
| H | -5.169379 | -1.226305 | -3.065231 |
| H | -4.250037 | -2.383683 | -4.032031 |
| H | -4.340606 | -2.592226 | -2.280458 |
| C | -1.752215 | -1.904703 | -3.027475 |
| H | -1.758979 | -2.671800 | -2.250124 |
| H | -1.739674 | -2.401677 | -4.005390 |
| H | -0.817771 | -1.342220 | -2.932473 |
| C | -2.942109 | 0.037264 | -4.090012 |
| H | -3.818193 | 0.695275 | -4.080987 |
| H | -2.051889 | 0.670656 | -4.022433 |
| H | -2.914575 | -0.482981 | -5.054940 |

TS Conformation 19

B3LYP/6-31G(d) Energy = -3411.892003
M06-2X/def2tzvpp/IEFPCM(chloroform) Energy = -3411.895276
M06-2X/def2tzvpp/IEFPCM(chloroform)//B3LYP/6-31G(d) Quasiharmonic Free Energy = -3411.168705

Frequencies (Top 3 out of 291)

1. -570.2279 cm-1
2. 7.6627 cm-1
3. 10.9636 cm-1

B3LYP/6-31G(d) Molecular Geometry in Cartesian Coordinates

| | | | |
|---|---|---|---|
| C | 3.826273 | -0.742009 | 0.552348 |
| C | 3.870701 | 0.599239 | 1.306673 |
| H | 4.546772 | 1.295620 | 0.812454 |
| C | 2.244706 | 2.314830 | 0.463360 |
| N | 0.889370 | 2.485753 | 0.342959 |

| | | | |
|---|---|---|---|
| H | 0.333340 | 1.720707 | 0.714605 |
| N | 2.539857 | 1.181569 | 1.177468 |
| H | 1.774845 | 0.539730 | 1.371207 |
| C | 4.657670 | -2.205511 | -1.233733 |
| C | 5.768354 | -0.029316 | -0.922826 |
| C | 6.031897 | -2.197272 | -1.944295 |
| H | 4.581603 | -3.036395 | -0.526144 |
| C | 6.296559 | -0.706203 | -2.196038 |
| H | 5.379201 | 0.975658 | -1.107902 |
| H | 6.552637 | 0.038317 | -0.159114 |
| H | 6.024187 | -2.797046 | -2.857746 |
| H | 6.793455 | -2.609878 | -1.271123 |
| H | 5.730120 | -0.363461 | -3.068335 |
| H | 7.351732 | -0.478832 | -2.371443 |
| N | 4.693378 | -0.941816 | -0.466034 |
| O | 2.970668 | -1.594893 | 0.850163 |
| S | 3.431524 | 3.345177 | -0.153331 |
| C | 3.480444 | -2.295888 | -2.199792 |
| C | 2.850690 | -1.164019 | -2.731583 |
| C | 3.031064 | -3.562931 | -2.597494 |
| C | 1.795316 | -1.296011 | -3.637648 |
| H | 3.164916 | -0.171705 | -2.420422 |
| C | 1.979478 | -3.697451 | -3.504209 |
| C | 1.355221 | -2.561954 | -4.026781 |
| H | 1.309413 | -0.407119 | -4.030017 |
| O | -1.895629 | -4.231467 | 1.097611 |
| O | -0.228177 | -0.209076 | 1.230164 |
| C | -2.702331 | -3.315294 | 1.225955 |
| C | -2.377550 | -1.874785 | 1.067652 |
| O | -4.015382 | -3.506273 | 1.413264 |
| C | -4.457624 | -4.877530 | 1.381344 |
| H | -3.966410 | -5.457015 | 2.166891 |
| H | -5.533362 | -4.833587 | 1.548473 |
| H | -4.235560 | -5.321921 | 0.408551 |
| S | -0.571478 | -1.652079 | 1.097363 |
| C | 0.235005 | -2.556535 | 2.438818 |
| H | -0.121716 | -2.108219 | 3.368683 |
| H | 1.306272 | -2.387919 | 2.298779 |
| H | -0.044174 | -3.606827 | 2.360811 |
| C | 0.099324 | -2.362748 | -0.410443 |
| H | -0.311235 | -1.773820 | -1.235077 |
| H | 1.186194 | -2.270446 | -0.339218 |
| H | -0.238081 | -3.398518 | -0.461296 |
| C | -3.065295 | -0.835539 | 1.906083 |
| C | -3.407784 | 0.393945 | 1.324447 |
| C | -3.358380 | -1.053220 | 3.262070 |
| C | -4.027175 | 1.387832 | 2.083349 |
| H | -3.203840 | 0.558206 | 0.271299 |
| C | -3.984936 | -0.063592 | 4.017295 |
| H | -3.114996 | -2.007936 | 3.722176 |
| C | -4.314432 | 1.160879 | 3.430348 |
| H | -4.276213 | 2.336303 | 1.616956 |
| H | -4.213800 | -0.247003 | 5.063634 |
| H | -4.798934 | 1.933447 | 4.021312 |
| C | -5.543711 | -1.030068 | -1.254314 |
| C | -6.774204 | -0.383563 | -1.370997 |
| C | -6.960048 | 0.626026 | -2.317983 |
| C | -5.893173 | 0.984233 | -3.145125 |
| C | -4.657731 | 0.348270 | -3.024477 |
| C | -4.458751 | -0.673256 | -2.077351 |
| H | -5.414920 | -1.820660 | -0.520280 |
| H | -7.595044 | -0.675749 | -0.719244 |

| | | | |
|---|---|---|---|
| H | -7.921022 | 1.125501 | -2.410078 |
| H | -6.019752 | 1.769819 | -3.887011 |
| H | -3.830392 | 0.638811 | -3.665623 |
| S | -2.883617 | -1.497839 | -1.967137 |
| H | -2.640033 | -1.661876 | -0.206497 |
| H | 3.504661 | -4.452580 | -2.186301 |
| H | 1.640211 | -4.688237 | -3.794699 |
| H | 0.525080 | -2.662350 | -4.719704 |
| C | 4.346164 | 0.479346 | 2.796969 |
| C | 0.083943 | 3.544990 | -0.119435 |
| C | 0.533553 | 4.644105 | -0.862806 |
| C | -1.281510 | 3.433420 | 0.205820 |
| C | -0.400457 | 5.598041 | -1.250523 |
| H | 1.572232 | 4.760358 | -1.131799 |
| C | -2.158423 | 4.417445 | -0.216041 |
| H | -1.655815 | 2.597562 | 0.786605 |
| C | -1.754892 | 5.525970 | -0.950674 |
| H | -2.454710 | 6.287173 | -1.271895 |
| F | 0.036542 | 6.653782 | -1.966057 |
| F | -3.465038 | 4.299500 | 0.110895 |
| C | 3.386662 | -0.356253 | 3.664641 |
| H | 3.342065 | -1.396355 | 3.331276 |
| H | 3.728735 | -0.345324 | 4.706378 |
| H | 2.371853 | 0.058008 | 3.650567 |
| C | 4.441196 | 1.902990 | 3.380225 |
| H | 5.133147 | 2.527934 | 2.805512 |
| H | 3.466129 | 2.399705 | 3.377436 |
| H | 4.797939 | 1.861047 | 4.416254 |
| C | 5.744331 | -0.169422 | 2.818853 |
| H | 5.730056 | -1.190337 | 2.418551 |
| H | 6.468271 | 0.417564 | 2.240543 |
| H | 6.117606 | -0.226045 | 3.847691 |

TS Conformation 20

B3LYP/6-31G(d) Energy = -3411.894609
M06-2X/def2tzvpp/IEFPCM(chloroform) Energy = -3411.895604
M06-2X/def2tzvpp/IEFPCM(chloroform)//B3LYP/6-31G(d) Quasiharmonic Free Energy = -3411.168692

Frequencies (Top 3 out of 291)

1. -997.5340 cm-1
2. 7.0188 cm-1
3. 9.8698 cm-1

B3LYP/6-31G(d) Molecular Geometry in Cartesian Coordinates

| | | | |
|---|---|---|---|
| C | 3.356571 | -1.325171 | 1.060175 |
| C | 3.597349 | 0.011296 | 1.793526 |
| H | 4.518185 | 0.488109 | 1.456076 |
| C | 2.653796 | 2.110379 | 0.765525 |
| N | 1.435491 | 2.585460 | 0.344569 |
| H | 0.672317 | 1.923445 | 0.452884 |
| N | 2.510745 | 0.887945 | 1.371674 |
| H | 1.629584 | 0.402057 | 1.223315 |
| C | 4.122778 | -3.094845 | -0.468644 |
| C | 5.730082 | -1.346661 | 0.151437 |
| C | 5.515538 | -3.385110 | -1.087554 |
| H | 3.863899 | -3.865371 | 0.267995 |
| C | 6.501905 | -2.624600 | -0.193797 |
| H | 5.819153 | -0.595624 | -0.644140 |

| | | | |
|---|---|---|---|
| H | 6.068170 | -0.893994 | 1.082926 |
| H | 5.548065 | -2.987548 | -2.108099 |
| H | 5.712168 | -4.459335 | -1.145738 |
| H | 7.455359 | -2.413696 | -0.686817 |
| H | 6.710755 | -3.190413 | 0.721978 |
| N | 4.335333 | -1.821085 | 0.260719 |
| O | 2.262346 | -1.897813 | 1.169520 |
| S | 4.136964 | 2.893746 | 0.601957 |
| C | 3.027673 | -3.057405 | -1.522547 |
| C | 2.728844 | -1.895024 | -2.243162 |
| C | 2.355044 | -4.242333 | -1.845943 |
| C | 1.776191 | -1.917660 | -3.262653 |
| H | 3.230352 | -0.963620 | -1.995165 |
| C | 1.409612 | -4.270772 | -2.872960 |
| C | 1.115503 | -3.105960 | -3.585482 |
| H | 1.544556 | -1.003794 | -3.802436 |
| O | -2.758690 | 1.371311 | 0.848431 |
| O | -0.021899 | 0.114860 | -0.025793 |
| C | -3.189981 | 0.755093 | -0.121404 |
| C | -2.693421 | -0.571730 | -0.572666 |
| O | -4.198484 | 1.195936 | -0.887577 |
| C | -4.757792 | 2.469546 | -0.504055 |
| H | -5.580113 | 2.637820 | -1.199426 |
| H | -5.121791 | 2.427532 | 0.525041 |
| H | -4.007703 | 3.258582 | -0.598242 |
| S | -1.126088 | -0.860332 | 0.227116 |
| C | -1.407553 | -1.053490 | 1.998412 |
| H | -2.199320 | -1.798607 | 2.127091 |
| H | -0.453088 | -1.380959 | 2.417896 |
| H | -1.713828 | -0.077148 | 2.371000 |
| C | -0.621198 | -2.503943 | -0.288969 |
| H | 0.348836 | -2.676172 | 0.185653 |
| H | -1.389807 | -3.210812 | 0.035437 |
| H | -0.515238 | -2.495874 | -1.374242 |
| C | -2.628614 | -0.821986 | -2.064233 |
| C | -3.338987 | -1.887457 | -2.636386 |
| C | -1.876186 | 0.020593 | -2.901806 |
| C | -3.296234 | -2.104664 | -4.015043 |
| H | -3.942730 | -2.530187 | -2.003312 |
| C | -1.832705 | -0.201066 | -4.277424 |
| H | -1.327270 | 0.854035 | -2.472177 |
| C | -2.542805 | -1.266166 | -4.837683 |
| H | -3.859516 | -2.928433 | -4.445105 |
| H | -1.252914 | 0.463829 | -4.912084 |
| H | -2.516128 | -1.434142 | -5.911189 |
| C | -5.329588 | -2.212796 | 3.209516 |
| C | -6.114714 | -1.460093 | 4.083703 |
| C | -6.868162 | -0.384126 | 3.610558 |
| C | -6.836123 | -0.075881 | 2.248931 |
| C | -6.061833 | -0.832948 | 1.368539 |
| C | -5.288866 | -1.909372 | 1.838168 |
| H | -4.744228 | -3.047761 | 3.584655 |
| H | -6.133981 | -1.715170 | 5.140748 |
| H | -7.476639 | 0.203170 | 4.292958 |
| H | -7.428566 | 0.751652 | 1.864895 |
| H | -6.057679 | -0.597920 | 0.308292 |
| S | -4.297915 | -2.894962 | 0.723208 |
| H | -3.492390 | -1.620629 | 0.021493 |
| H | 2.572897 | -5.151428 | -1.288965 |
| H | 0.899681 | -5.200578 | -3.111315 |
| H | 0.371280 | -3.118828 | -4.376806 |
| C | 3.682528 | -0.148412 | 3.356094 |

| | | | |
|---|---|---|---|
| C | 0.998985 | 3.786756 | -0.236903 |
| C | 1.827126 | 4.821571 | -0.692550 |
| C | -0.401473 | 3.903955 | -0.361626 |
| C | 1.225701 | 5.939911 | -1.258238 |
| H | 2.902068 | 4.762541 | -0.610116 |
| C | -0.930430 | 5.043511 | -0.940264 |
| H | -1.069690 | 3.132636 | 0.009310 |
| C | -0.147430 | 6.093210 | -1.406455 |
| H | -0.580328 | 6.978906 | -1.854240 |
| F | 2.025517 | 6.932809 | -1.697357 |
| F | -2.276598 | 5.142125 | -1.054883 |
| C | 4.045479 | 1.220375 | 3.963827 |
| H | 3.285737 | 1.973164 | 3.733512 |
| H | 4.119745 | 1.136289 | 5.054851 |
| H | 5.002526 | 1.591256 | 3.582471 |
| C | 4.792668 | -1.160650 | 3.702608 |
| H | 4.584941 | -2.157438 | 3.295237 |
| H | 5.771163 | -0.831157 | 3.331735 |
| H | 4.878994 | -1.264223 | 4.790203 |
| C | 2.352536 | -0.628963 | 3.967506 |
| H | 2.069841 | -1.618690 | 3.599674 |
| H | 2.446011 | -0.679247 | 5.058814 |
| H | 1.540971 | 0.072064 | 3.741297 |

TS Conformation 21

B3LYP/6-31G(d) Energy = -3411.887628
M06-2X/def2tzvpp/IEFPCM(chloroform) Energy = -3411.894225
M06-2X/def2tzvpp/IEFPCM(chloroform)//B3LYP/6-31G(d) Quasiharmonic Free Energy = -3411.168434

Frequencies (Top 3 out of 291)

1. -928.2426 cm-1
2. 7.2033 cm-1
3. 9.1122 cm-1

B3LYP/6-31G(d) Molecular Geometry in Cartesian Coordinates

| | | | |
|---|---|---|---|
| C | -2.622226 | -1.499150 | 0.449023 |
| C | -2.740063 | -0.726120 | -0.879269 |
| H | -3.626021 | -0.092834 | -0.876687 |
| C | -1.640064 | 1.542031 | -0.969710 |
| N | -0.383741 | 2.090570 | -0.900859 |
| H | 0.378845 | 1.419695 | -0.878400 |
| N | -1.587989 | 0.174553 | -0.913327 |
| H | -0.724230 | -0.229527 | -0.559789 |
| C | -4.576678 | -0.210294 | 1.454494 |
| C | -3.348811 | -1.913933 | 2.739766 |
| C | -4.729984 | 0.038470 | 2.976985 |
| H | -4.189183 | 0.690190 | 0.970488 |
| C | -4.478267 | -1.339884 | 3.603784 |
| H | -3.373960 | -3.003611 | 2.643329 |
| H | -2.360361 | -1.643160 | 3.132006 |
| H | -5.708011 | 0.458982 | 3.223385 |
| H | -3.961267 | 0.749281 | 3.303334 |
| H | -5.376285 | -1.962140 | 3.527745 |
| H | -4.200068 | -1.285313 | 4.660271 |
| N | -3.539661 | -1.275144 | 1.422276 |
| O | -1.670324 | -2.276140 | 0.643389 |
| S | -3.078147 | 2.409121 | -1.146309 |
| C | -5.889620 | -0.592749 | 0.784120 |

| | | | |
|---|---|---|---|
| C | -6.384688 | -1.903250 | 0.779988 |
| C | -6.658954 | 0.418418 | 0.191858 |
| C | -7.626575 | -2.193127 | 0.211954 |
| H | -5.788209 | -2.707627 | 1.200876 |
| C | -7.899992 | 0.130302 | -0.376835 |
| C | -8.389902 | -1.176871 | -0.365000 |
| H | -7.994036 | -3.216016 | 0.215188 |
| O | 3.867156 | -3.271699 | 1.506099 |
| O | 0.883973 | -0.190462 | 0.736527 |
| C | 4.271871 | -2.111868 | 1.478256 |
| C | 3.436814 | -0.909676 | 1.241958 |
| O | 5.567885 | -1.785080 | 1.610560 |
| C | 6.474477 | -2.887467 | 1.794339 |
| H | 7.459468 | -2.432857 | 1.897338 |
| H | 6.214280 | -3.455039 | 2.691599 |
| H | 6.445445 | -3.553078 | 0.928189 |
| S | 1.713971 | -1.388526 | 1.030214 |
| C | 1.577979 | -2.596328 | -0.296019 |
| H | 0.508575 | -2.806532 | -0.372675 |
| H | 1.965408 | -2.119002 | -1.199449 |
| H | 2.168713 | -3.471817 | -0.030910 |
| C | 1.046702 | -2.236980 | 2.479252 |
| H | 1.112867 | -1.527492 | 3.307111 |
| H | 0.006526 | -2.474165 | 2.235052 |
| H | 1.658135 | -3.122181 | 2.660459 |
| C | 3.556076 | 0.248518 | 2.199096 |
| C | 3.946752 | 0.050285 | 3.535694 |
| C | 3.261104 | 1.552446 | 1.771139 |
| C | 4.056864 | 1.127175 | 4.412492 |
| H | 4.184458 | -0.950410 | 3.887116 |
| C | 3.360919 | 2.627739 | 2.653975 |
| H | 2.952714 | 1.726438 | 0.747374 |
| C | 3.760759 | 2.420190 | 3.974050 |
| H | 4.370360 | 0.956024 | 5.438828 |
| H | 3.129478 | 3.628960 | 2.302120 |
| H | 3.843720 | 3.260061 | 4.658485 |
| C | 3.185134 | -1.355744 | -3.596193 |
| C | 3.286485 | -2.531435 | -4.341743 |
| C | 4.210396 | -3.517781 | -3.989063 |
| C | 5.042636 | -3.307360 | -2.887131 |
| C | 4.953910 | -2.128268 | -2.146496 |
| C | 4.016742 | -1.131465 | -2.481747 |
| H | 2.463015 | -0.593492 | -3.875800 |
| H | 2.636918 | -2.676779 | -5.201871 |
| H | 4.285478 | -4.433986 | -4.568481 |
| H | 5.774828 | -4.061827 | -2.607757 |
| H | 5.625673 | -1.957405 | -1.309698 |
| S | 3.884230 | 0.366427 | -1.528081 |
| H | 3.704131 | -0.384909 | -0.023891 |
| H | -6.274829 | 1.435311 | 0.165538 |
| H | -8.478645 | 0.926316 | -0.837659 |
| H | -9.354444 | -1.403779 | -0.810945 |
| C | -2.849037 | -1.638090 | -2.153974 |
| C | 0.074694 | 3.424597 | -0.915575 |
| C | -0.695411 | 4.527511 | -0.523173 |
| C | 1.416768 | 3.603607 | -1.300549 |
| C | -0.100406 | 5.782357 | -0.541917 |
| H | -1.726831 | 4.422257 | -0.223596 |
| C | 1.950859 | 4.883694 | -1.287433 |
| H | 2.033507 | 2.768829 | -1.623510 |
| C | 1.218910 | 6.005515 | -0.914430 |
| H | 1.652243 | 6.997939 | -0.919683 |

| | | | |
|---|---|---|---|
| F | -0.843890 | 6.842311 | -0.162240 |
| F | 3.234227 | 5.050910 | -1.660856 |
| C | -4.025272 | -2.615567 | -1.968669 |
| H | -4.965431 | -2.087899 | -1.777015 |
| H | -4.157925 | -3.215087 | -2.876850 |
| H | -3.848270 | -3.310482 | -1.139203 |
| C | -1.566503 | -2.440034 | -2.438348 |
| H | -0.696202 | -1.782265 | -2.544336 |
| H | -1.365538 | -3.168633 | -1.649279 |
| H | -1.677289 | -2.984661 | -3.383558 |
| C | -3.139860 | -0.722262 | -3.360094 |
| H | -4.068132 | -0.157846 | -3.222734 |
| H | -2.332703 | -0.000265 | -3.518941 |
| H | -3.237798 | -1.325220 | -4.270775 |

TS Conformation 22

B3LYP/6-31G(d) Energy = -3411.897975
M06-2X/def2tzvpp/IEFPCM(chloroform) Energy = -3411.895822
M06-2X/def2tzvpp/IEFPCM(chloroform)//B3LYP/6-31G(d) Quasiharmonic Free Energy = -3411.168377

Frequencies (Top 3 out of 291)

1. -981.2284 cm-1
2. 10.2554 cm-1
3. 12.2737 cm-1

B3LYP/6-31G(d) Molecular Geometry in Cartesian Coordinates

| | | | |
|---|---|---|---|
| C | 2.897775 | -1.477470 | -0.033780 |
| C | 3.323431 | -0.356363 | 0.937934 |
| H | 4.192795 | 0.175977 | 0.555957 |
| C | 2.215644 | 1.837022 | 0.371912 |
| N | 0.958545 | 2.391574 | 0.401115 |
| H | 0.221738 | 1.762955 | 0.712619 |
| N | 2.224798 | 0.603697 | 0.958532 |
| H | 1.326736 | 0.210171 | 1.228817 |
| C | 4.741358 | -0.889574 | -1.693850 |
| C | 3.139864 | -2.713098 | -2.118929 |
| C | 4.607528 | -1.154598 | -3.215295 |
| H | 4.534534 | 0.163597 | -1.486002 |
| C | 4.113710 | -2.605688 | -3.298694 |
| H | 3.097954 | -3.708298 | -1.666299 |
| H | 2.116437 | -2.447368 | -2.410461 |
| H | 5.549427 | -0.982552 | -3.741985 |
| H | 3.855750 | -0.473091 | -3.631031 |
| H | 4.950570 | -3.301044 | -3.174539 |
| H | 3.628850 | -2.837597 | -4.251291 |
| N | 3.639509 | -1.722479 | -1.141153 |
| O | 1.850405 | -2.116966 | 0.177364 |
| S | 3.584517 | 2.560697 | -0.303197 |
| C | 6.122740 | -1.231329 | -1.152985 |
| C | 6.512354 | -2.539617 | -0.836390 |
| C | 7.061613 | -0.199637 | -1.015294 |
| C | 7.812204 | -2.811193 | -0.406840 |
| H | 5.791441 | -3.349529 | -0.901301 |
| C | 8.361621 | -0.469023 | -0.585059 |
| C | 8.742245 | -1.777417 | -0.282643 |
| H | 8.096042 | -3.831654 | -0.163100 |
| O | -2.915249 | 1.025270 | -0.801040 |
| O | -0.688544 | 0.043939 | 1.376614 |

| | | | |
|---|---|---|---|
| C | -3.640612 | 0.512038 | 0.047751 |
| C | -3.315001 | -0.747968 | 0.767522 |
| O | -4.840103 | 0.991288 | 0.386395 |
| C | -5.299635 | 2.130612 | -0.372937 |
| H | -5.523437 | 1.819953 | -1.396543 |
| H | -6.207515 | 2.459076 | 0.132409 |
| H | -4.546881 | 2.921747 | -0.381859 |
| S | -1.547621 | -0.979202 | 0.696172 |
| C | -1.260633 | -2.590538 | 1.441749 |
| H | -0.191708 | -2.782306 | 1.318111 |
| H | -1.541118 | -2.522548 | 2.493332 |
| H | -1.873053 | -3.329876 | 0.918881 |
| C | -1.040674 | -1.243463 | -1.013428 |
| H | -1.754578 | -1.952368 | -1.450607 |
| H | -1.098590 | -0.275616 | -1.507763 |
| H | -0.022147 | -1.638797 | -0.958437 |
| C | -3.854221 | -0.950475 | 2.164765 |
| C | -3.564737 | -0.033269 | 3.190339 |
| C | -4.663774 | -2.059417 | 2.452647 |
| C | -4.070754 | -0.223999 | 4.474837 |
| H | -2.942028 | 0.830998 | 2.977428 |
| C | -5.174017 | -2.243296 | 3.739035 |
| H | -4.902597 | -2.764350 | 1.661581 |
| C | -4.877454 | -1.330441 | 4.751768 |
| H | -3.838916 | 0.493212 | 5.257432 |
| H | -5.806670 | -3.101961 | 3.946825 |
| H | -5.275579 | -1.476715 | 5.752338 |
| C | -5.119934 | -1.106381 | -2.735798 |
| C | -6.091239 | -0.476943 | -3.517099 |
| C | -7.378362 | -1.009482 | -3.613104 |
| C | -7.684541 | -2.179310 | -2.914776 |
| C | -6.720409 | -2.805495 | -2.124729 |
| C | -5.421672 | -2.279203 | -2.021799 |
| H | -4.120045 | -0.686042 | -2.683569 |
| H | -5.832361 | 0.426394 | -4.065272 |
| H | -8.130969 | -0.522065 | -4.227097 |
| H | -8.682179 | -2.607218 | -2.980609 |
| H | -6.966966 | -3.710979 | -1.578216 |
| S | -4.201908 | -3.124597 | -1.024976 |
| H | -3.747925 | -1.816156 | -0.064641 |
| H | 6.767189 | 0.823205 | -1.238760 |
| H | 9.073340 | 0.345324 | -0.479121 |
| H | 9.752936 | -1.989119 | 0.055537 |
| C | 3.684550 | -0.856508 | 2.382750 |
| C | 0.465079 | 3.651962 | 0.008772 |
| C | -0.914585 | 3.702964 | -0.261783 |
| C | 1.234635 | 4.821164 | -0.052843 |
| C | -1.489196 | 4.921483 | -0.581336 |
| H | -1.529779 | 2.809312 | -0.256864 |
| C | 0.596201 | 6.007186 | -0.394213 |
| H | 2.295234 | 4.815734 | 0.149383 |
| C | -0.764585 | 6.104407 | -0.661522 |
| H | -1.230413 | 7.047349 | -0.919264 |
| F | -2.818975 | 4.959333 | -0.831630 |
| F | 1.337089 | 7.132359 | -0.452644 |
| C | 4.234860 | 0.352196 | 3.166468 |
| H | 3.491290 | 1.151881 | 3.242651 |
| H | 4.509663 | 0.047050 | 4.183060 |
| H | 5.126891 | 0.768444 | 2.685074 |
| C | 4.779632 | -1.934800 | 2.287369 |
| H | 4.427152 | -2.827087 | 1.756886 |
| H | 5.671230 | -1.563857 | 1.772240 |

| | | | |
|---|---|---|---|
| H | 5.080468 | -2.248393 | 3.293819 |
| C | 2.473936 | -1.438566 | 3.138685 |
| H | 1.661956 | -0.709306 | 3.238302 |
| H | 2.080027 | -2.327834 | 2.640592 |
| H | 2.781271 | -1.720881 | 4.152658 |

TS Conformation 23

B3LYP/6-31G(d) Energy = -3411.891164
M06-2X/def2tzvpp/IEFPCM(chloroform) Energy = -3411.893658
M06-2X/def2tzvpp/IEFPCM(chloroform)//B3LYP/6-31G(d) Quasiharmonic Free Energy = -3411.168377

Frequencies (Top 3 out of 291)

1. -991.2333 cm-1
2. 7.7107 cm-1
3. 9.6316 cm-1

B3LYP/6-31G(d) Molecular Geometry in Cartesian Coordinates

| | | | |
|---|---|---|---|
| C | 2.736699 | -2.204342 | 0.693371 |
| C | 3.320540 | -1.166013 | 1.676911 |
| H | 4.368440 | -0.971504 | 1.451271 |
| C | 3.121302 | 1.103317 | 0.639871 |
| N | 2.143202 | 2.010978 | 0.310971 |
| H | 1.189252 | 1.703229 | 0.485761 |
| N | 2.626721 | 0.089788 | 1.411081 |
| H | 1.621881 | 0.067369 | 1.561911 |
| C | 3.013687 | -3.700143 | -1.236029 |
| C | 5.044108 | -2.747304 | -0.228709 |
| C | 4.277057 | -4.173644 | -1.997779 |
| H | 2.540887 | -4.545252 | -0.719629 |
| C | 5.412317 | -4.034065 | -0.976819 |
| H | 5.400379 | -1.851335 | -0.751089 |
| H | 5.447098 | -2.729675 | 0.785131 |
| H | 4.454458 | -3.510694 | -2.852189 |
| H | 4.155386 | -5.188854 | -2.386229 |
| H | 6.402217 | -3.975726 | -1.438879 |
| H | 5.411906 | -4.883145 | -0.282679 |
| N | 3.567228 | -2.782873 | -0.212559 |
| O | 1.523019 | -2.442781 | 0.689441 |
| S | 4.734672 | 1.208866 | 0.153321 |
| C | 1.981798 | -3.074172 | -2.160879 |
| C | 2.084159 | -1.744622 | -2.589989 |
| C | 0.945097 | -3.867301 | -2.665879 |
| C | 1.166130 | -1.219611 | -3.499869 |
| H | 2.875240 | -1.110303 | -2.198059 |
| C | 0.034058 | -3.350650 | -3.590359 |
| C | 0.142049 | -2.022620 | -4.010299 |
| H | 1.248911 | -0.181301 | -3.807289 |
| O | -0.362659 | 0.789040 | 1.511761 |
| O | -0.970068 | 0.927041 | -1.604299 |
| C | -1.529658 | 1.173711 | 1.622511 |
| C | -2.624749 | 0.849902 | 0.682841 |
| O | -1.968198 | 1.883322 | 2.665091 |
| C | -1.000057 | 2.187801 | 3.687341 |
| H | -0.152927 | 2.732170 | 3.263241 |
| H | -1.533317 | 2.806611 | 4.408201 |
| H | -0.645758 | 1.268251 | 4.159471 |
| S | -1.922749 | 0.121982 | -0.794619 |
| C | -1.249581 | -1.487139 | -0.349129 |

| | | | |
|---|---|---|---|
| H | -0.298790 | -1.342270 | 0.163861 |
| H | -1.975511 | -1.979288 | 0.305831 |
| H | -1.092801 | -2.028079 | -1.285319 |
| C | -3.364720 | -0.292687 | -1.794479 |
| H | -4.046780 | -0.923486 | -1.219499 |
| H | -3.842409 | 0.645073 | -2.080489 |
| H | -2.977600 | -0.813607 | -2.672819 |
| C | -3.642488 | 1.935803 | 0.400671 |
| C | -3.341127 | 3.031113 | -0.428119 |
| C | -4.902278 | 1.875294 | 1.014261 |
| C | -4.290496 | 4.027654 | -0.656669 |
| H | -2.363557 | 3.108372 | -0.895579 |
| C | -5.841897 | 2.883855 | 0.796531 |
| H | -5.131418 | 1.042545 | 1.671891 |
| C | -5.543076 | 3.957125 | -0.044069 |
| H | -4.043045 | 4.865044 | -1.303269 |
| H | -6.810757 | 2.827446 | 1.285511 |
| H | -6.278485 | 4.738586 | -0.215559 |
| C | -4.491593 | -3.680606 | 0.486201 |
| C | -5.186203 | -4.388785 | -0.495419 |
| C | -6.301243 | -3.825714 | -1.119999 |
| C | -6.721202 | -2.548794 | -0.740499 |
| C | -6.034941 | -1.842025 | 0.249231 |
| C | -4.897951 | -2.389736 | 0.875581 |
| H | -3.629173 | -4.127687 | 0.972781 |
| H | -4.853174 | -5.386406 | -0.772409 |
| H | -6.841103 | -4.377424 | -1.884869 |
| H | -7.596241 | -2.102413 | -1.207519 |
| H | -6.384410 | -0.859034 | 0.551421 |
| S | -4.006801 | -1.511526 | 2.153551 |
| H | -3.294100 | -0.242467 | 1.289291 |
| H | 0.849256 | -4.898791 | -2.333379 |
| H | -0.756903 | -3.986299 | -3.980899 |
| H | -0.560861 | -1.616870 | -4.733699 |
| C | 3.218279 | -1.587903 | 3.181421 |
| C | 2.226934 | 3.240948 | -0.372049 |
| C | 3.301234 | 4.129427 | -0.230539 |
| C | 1.118504 | 3.594689 | -1.159139 |
| C | 3.236685 | 5.346507 | -0.895559 |
| H | 4.166164 | 3.884106 | 0.368291 |
| C | 1.109455 | 4.833159 | -1.782479 |
| H | 0.299313 | 2.901220 | -1.315199 |
| C | 2.155766 | 5.740728 | -1.677059 |
| H | 2.134387 | 6.700678 | -2.177819 |
| F | 4.268866 | 6.203786 | -0.759439 |
| F | 0.036385 | 5.166110 | -2.533189 |
| C | 1.760999 | -1.672502 | 3.678181 |
| H | 1.177839 | -2.395401 | 3.103501 |
| H | 1.749939 | -1.972792 | 4.732831 |
| H | 1.254790 | -0.702451 | 3.611991 |
| C | 3.982310 | -0.544093 | 4.019731 |
| H | 3.553831 | 0.455377 | 3.894181 |
| H | 3.933820 | -0.803613 | 5.083991 |
| H | 5.038800 | -0.492364 | 3.731541 |
| C | 3.888718 | -2.965043 | 3.357451 |
| H | 4.940078 | -2.946484 | 3.043891 |
| H | 3.869778 | -3.259993 | 4.412771 |
| H | 3.371277 | -3.746613 | 2.789171 |

TS Conformation 24

B3LYP/6-31G(d) Energy = -3411.8969
M06-2X/def2tzvpp/IEFPCM(chloroform) Energy = -3411.895506
M06-2X/def2tzvpp/IEFPCM(chloroform)//B3LYP/6-31G(d) Quasiharmonic Free Energy = -3411.168357

Frequencies (Top 3 out of 291)

1. -980.3774 cm-1
2. 9.8299 cm-1
3. 11.4873 cm-1

B3LYP/6-31G(d) Molecular Geometry in Cartesian Coordinates

| | | | |
|---|---|---|---|
| C | 2.803617 | -1.642077 | 0.012000 |
| C | 3.286785 | -0.562514 | 1.003790 |
| H | 4.243362 | -0.148988 | 0.688995 |
| C | 2.509201 | 1.740152 | 0.329858 |
| N | 1.330575 | 2.441924 | 0.243766 |
| H | 0.498545 | 1.910602 | 0.489464 |
| N | 2.317461 | 0.525315 | 0.923583 |
| H | 1.358689 | 0.249361 | 1.120683 |
| C | 4.827872 | -1.312016 | -1.502564 |
| C | 3.053651 | -2.935767 | -2.036997 |
| C | 4.781793 | -1.587889 | -3.027277 |
| H | 4.736104 | -0.237769 | -1.321570 |
| C | 4.122987 | -2.970163 | -3.135649 |
| H | 2.854312 | -3.909538 | -1.579791 |
| H | 2.096868 | -2.553063 | -2.412735 |
| H | 5.775842 | -1.540857 | -3.478736 |
| H | 4.153303 | -0.828831 | -3.508224 |
| H | 4.856442 | -3.758802 | -2.937308 |
| H | 3.689945 | -3.160752 | -4.121776 |
| N | 3.592787 | -1.994627 | -1.031762 |
| O | 1.673010 | -2.146073 | 0.146424 |
| S | 4.008764 | 2.282410 | -0.226698 |
| C | 6.110705 | -1.808803 | -0.850532 |
| C | 6.308161 | -3.147126 | -0.485294 |
| C | 7.158388 | -0.898230 | -0.655300 |
| C | 7.527584 | -3.567078 | 0.048217 |
| H | 5.498242 | -3.862701 | -0.594129 |
| C | 8.378137 | -1.316012 | -0.121212 |
| C | 8.567818 | -2.653752 | 0.229200 |
| H | 7.662001 | -4.608580 | 0.328413 |
| O | -2.514699 | 1.503244 | -1.311326 |
| O | -0.670842 | 0.336439 | 1.087986 |
| C | -3.401253 | 1.102463 | -0.564364 |
| C | -3.317433 | -0.135592 | 0.254393 |
| O | -4.592537 | 1.705158 | -0.449719 |
| C | -4.778578 | 2.874894 | -1.277763 |
| H | -4.778121 | 2.591077 | -2.332874 |
| H | -5.749862 | 3.274488 | -0.987730 |
| H | -3.985356 | 3.604428 | -1.099937 |
| S | -1.593638 | -0.599190 | 0.368278 |
| C | -1.599519 | -2.196797 | 1.192580 |
| H | -1.930923 | -2.033400 | 2.218802 |
| H | -0.566396 | -2.550988 | 1.153575 |
| H | -2.292195 | -2.852354 | 0.653972 |
| C | -0.976301 | -0.995394 | -1.280950 |
| H | -0.038452 | -1.535727 | -1.121562 |
| H | -1.733816 | -1.598576 | -1.790374 |
| H | -0.838480 | -0.045013 | -1.792996 |
| C | -3.987670 | -0.157996 | 1.608081 |
| C | -4.885081 | -1.184835 | 1.937069 |

| | | | |
|---|---|---|---|
| C | -3.724267 | 0.846383 | 2.555930 |
| C | -5.510708 | -1.199930 | 3.184909 |
| H | -5.103277 | -1.960529 | 1.210851 |
| C | -4.347316 | 0.825791 | 3.802149 |
| H | -3.028919 | 1.644894 | 2.312715 |
| C | -5.242950 | -0.198566 | 4.118889 |
| H | -6.212013 | -1.995017 | 3.422106 |
| H | -4.135021 | 1.609478 | 4.524132 |
| H | -5.731867 | -0.212944 | 5.089304 |
| C | -6.718070 | -1.346912 | -1.255593 |
| C | -8.113389 | -1.369566 | -1.229776 |
| C | -8.808633 | -2.571412 | -1.371740 |
| C | -8.088726 | -3.757401 | -1.534391 |
| C | -6.694805 | -3.743552 | -1.548834 |
| C | -5.985498 | -2.536767 | -1.412194 |
| H | -6.195434 | -0.400512 | -1.153672 |
| H | -8.658510 | -0.436860 | -1.103319 |
| H | -9.895157 | -2.584295 | -1.356728 |
| H | -8.614643 | -4.702902 | -1.645650 |
| H | -6.142003 | -4.671104 | -1.667387 |
| S | -4.202870 | -2.565042 | -1.468168 |
| H | -3.803575 | -1.191402 | -0.542790 |
| H | 7.012239 | 0.147478 | -0.915985 |
| H | 9.176283 | -0.593758 | 0.027902 |
| H | 9.515561 | -2.980672 | 0.648220 |
| C | 3.465875 | -1.073615 | 2.478409 |
| C | 1.029065 | 3.746255 | -0.195399 |
| C | 1.935839 | 4.814306 | -0.194702 |
| C | -0.305888 | 3.957708 | -0.585971 |
| C | 1.476073 | 6.062813 | -0.595702 |
| H | 2.966775 | 4.685473 | 0.099303 |
| C | -0.700511 | 5.230952 | -0.959791 |
| H | -1.020337 | 3.142787 | -0.632490 |
| C | 0.164873 | 6.317812 | -0.981293 |
| H | -0.160189 | 7.305709 | -1.282872 |
| F | 2.348821 | 7.090604 | -0.594121 |
| F | -1.990029 | 5.422792 | -1.325668 |
| C | 4.420541 | -2.281751 | 2.483770 |
| H | 5.388721 | -2.036369 | 2.036147 |
| H | 4.601087 | -2.607659 | 3.514761 |
| H | 4.000425 | -3.134926 | 1.938437 |
| C | 2.135142 | -1.484130 | 3.139122 |
| H | 1.671580 | -2.325531 | 2.618432 |
| H | 2.323013 | -1.783785 | 4.176999 |
| H | 1.417510 | -0.656235 | 3.166733 |
| C | 4.099768 | 0.073104 | 3.291500 |
| H | 5.073760 | 0.363196 | 2.881788 |
| H | 3.460187 | 0.961435 | 3.295940 |
| H | 4.249612 | -0.241746 | 4.330967 |

TS Conformation 25

B3LYP/6-31G(d) Energy = -3411.885406
M06-2X/def2tzvpp/IEFPCM(chloroform) Energy = -3411.893711
M06-2X/def2tzvpp/IEFPCM(chloroform)//B3LYP/6-31G(d) Quasiharmonic Free Energy = -3411.167839

Frequencies (Top 3 out of 291)

1. -704.3096 cm-1
2. 6.4927 cm-1
3. 11.5374 cm-1

B3LYP/6-31G(d) Molecular Geometry in Cartesian Coordinates

| | | | |
|---|---|---|---|
| C | 3.593761 | -1.222926 | 0.857305 |
| C | 3.550625 | -0.026752 | 1.827358 |
| H | 4.399887 | 0.632769 | 1.655015 |
| C | 2.387566 | 1.948441 | 0.813226 |
| N | 1.127663 | 2.357584 | 0.461497 |
| H | 0.403548 | 1.646120 | 0.526110 |
| N | 2.370361 | 0.757267 | 1.484220 |
| H | 1.479294 | 0.274676 | 1.562554 |
| C | 4.664700 | -2.423653 | -0.987654 |
| C | 5.852231 | -0.479096 | -0.057639 |
| C | 6.143363 | -2.425475 | -1.446134 |
| H | 4.406152 | -3.355698 | -0.475762 |
| C | 6.554169 | -0.950145 | -1.338263 |
| H | 5.591147 | 0.582134 | -0.081666 |
| H | 6.478426 | -0.667099 | 0.824482 |
| H | 6.262408 | -2.838469 | -2.451140 |
| H | 6.737292 | -3.035791 | -0.754760 |
| H | 6.179299 | -0.387660 | -2.200341 |
| H | 7.637248 | -0.806410 | -1.289541 |
| N | 4.636370 | -1.323089 | -0.005009 |
| O | 2.666204 | -2.045584 | 0.833482 |
| S | 3.811939 | 2.793353 | 0.461752 |
| C | 3.682184 | -2.233260 | -2.141195 |
| C | 3.174456 | -0.980404 | -2.503290 |
| C | 3.298749 | -3.354943 | -2.891201 |
| C | 2.307967 | -0.849598 | -3.592212 |
| H | 3.438116 | -0.100282 | -1.924435 |
| C | 2.438664 | -3.228565 | -3.982703 |
| C | 1.939734 | -1.971074 | -4.338436 |
| H | 1.920183 | 0.131667 | -3.850107 |
| O | -0.539517 | -0.056083 | 1.577597 |
| O | -0.403339 | 0.067604 | -1.335695 |
| C | -1.650296 | -0.576523 | 1.512665 |
| C | -2.278082 | -1.162786 | 0.301588 |
| O | -2.505549 | -0.598871 | 2.544178 |
| C | -2.072408 | 0.070299 | 3.745155 |
| H | -2.914653 | -0.006490 | 4.431843 |
| H | -1.190682 | -0.424192 | 4.160443 |
| H | -1.840052 | 1.116323 | 3.534075 |
| S | -1.070331 | -1.219906 | -1.047945 |
| C | 0.123777 | -2.550724 | -0.816622 |
| H | -0.420427 | -3.455689 | -0.538405 |
| H | 0.651533 | -2.676038 | -1.765613 |
| H | 0.830493 | -2.240988 | -0.039563 |
| C | -2.038980 | -1.737073 | -2.477805 |
| H | -1.347057 | -1.706451 | -3.322633 |
| H | -2.443796 | -2.737551 | -2.323300 |
| H | -2.839279 | -0.994631 | -2.583853 |
| C | -3.051943 | -2.450426 | 0.460785 |
| C | -4.290839 | -2.616545 | -0.177684 |
| C | -2.554312 | -3.498803 | 1.255834 |
| C | -5.005852 | -3.806459 | -0.032278 |
| H | -4.701986 | -1.802626 | -0.765790 |
| C | -3.271794 | -4.684623 | 1.401611 |
| H | -1.608422 | -3.377667 | 1.778780 |
| C | -4.498487 | -4.842971 | 0.751994 |
| H | -5.968689 | -3.913956 | -0.523526 |
| H | -2.875267 | -5.481812 | 2.024337 |
| H | -5.059501 | -5.766498 | 0.865982 |

| | | | |
|---|---|---|---|
| C | -6.812678 | 0.952217 | -1.191204 |
| C | -8.101945 | 0.799113 | -0.682770 |
| C | -8.301077 | 0.430209 | 0.649898 |
| C | -7.189147 | 0.224044 | 1.468848 |
| C | -5.897099 | 0.386816 | 0.967844 |
| C | -5.683104 | 0.750346 | -0.375381 |
| H | -6.666974 | 1.235293 | -2.229886 |
| H | -8.956730 | 0.966129 | -1.334658 |
| H | -9.306602 | 0.308671 | 1.044162 |
| H | -7.325854 | -0.059921 | 2.510253 |
| H | -5.042138 | 0.234456 | 1.620530 |
| S | -4.050256 | 0.988758 | -1.047384 |
| H | -3.086770 | -0.221130 | -0.187164 |
| H | 3.677900 | -4.337557 | -2.616640 |
| H | 2.159774 | -4.109537 | -4.555509 |
| H | 1.278322 | -1.866768 | -5.194986 |
| C | 3.583662 | -0.439852 | 3.338066 |
| C | 0.685481 | 3.565192 | -0.123855 |
| C | 1.284698 | 4.805076 | 0.136692 |
| C | -0.458726 | 3.490924 | -0.934650 |
| C | 0.730277 | 5.936603 | -0.445245 |
| H | 2.159709 | 4.894633 | 0.762984 |
| C | -0.968986 | 4.661476 | -1.478165 |
| H | -0.941362 | 2.544639 | -1.151486 |
| C | -0.395844 | 5.908721 | -1.258605 |
| H | -0.806300 | 6.809863 | -1.697071 |
| F | 1.308203 | 7.130689 | -0.194856 |
| F | -2.058407 | 4.586211 | -2.265101 |
| C | 4.818379 | -1.332773 | 3.572880 |
| H | 5.743674 | -0.827719 | 3.268148 |
| H | 4.912616 | -1.574415 | 4.637778 |
| H | 4.746190 | -2.279935 | 3.025746 |
| C | 2.316923 | -1.198542 | 3.780466 |
| H | 2.392891 | -1.449297 | 4.845620 |
| H | 1.418261 | -0.584697 | 3.650823 |
| H | 2.182175 | -2.121801 | 3.212292 |
| C | 3.722290 | 0.846360 | 4.176154 |
| H | 4.639842 | 1.391510 | 3.926993 |
| H | 2.877896 | 1.522156 | 4.007655 |
| H | 3.752969 | 0.600414 | 5.244374 |

TS Conformation 26

B3LYP/6-31G(d) Energy = -3411.890165
M06-2X/def2tzvpp/IEFPCM(chloroform) Energy = -3411.894127
M06-2X/def2tzvpp/IEFPCM(chloroform)//B3LYP/6-31G(d) Quasiharmonic Free Energy = -3411.167715

Frequencies (Top 3 out of 291)

1. -962.5185 cm-1
2. 9.0598 cm-1
3. 9.8699 cm-1

B3LYP/6-31G(d) Molecular Geometry in Cartesian Coordinates

| | | | |
|---|---|---|---|
| C | 2.837351 | -1.390079 | -0.136178 |
| C | 2.801287 | -0.237042 | 0.885922 |
| H | 3.592680 | 0.481844 | 0.681308 |
| C | 1.407530 | 1.748468 | 0.209185 |
| N | 0.085426 | 2.086161 | 0.055837 |
| H | -0.572970 | 1.373716 | 0.356491 |

| | | | |
|---|---|---|---|
| N | 1.540750 | 0.462289 | 0.651050 |
| H | 0.720012 | -0.136918 | 0.595468 |
| C | 4.754191 | -0.312164 | -1.426373 |
| C | 3.740726 | -2.440296 | -2.140994 |
| C | 4.976668 | -0.540380 | -2.943425 |
| H | 4.268278 | 0.654156 | -1.267211 |
| C | 4.867636 | -2.062470 | -3.110144 |
| H | 3.847936 | -3.436444 | -1.701330 |
| H | 2.757865 | -2.404210 | -2.627584 |
| H | 5.932432 | -0.131155 | -3.279785 |
| H | 4.176606 | -0.039397 | -3.501679 |
| H | 5.806125 | -2.545685 | -2.818767 |
| H | 4.643767 | -2.365156 | -4.137170 |
| N | 3.800683 | -1.403316 | -1.090272 |
| O | 1.956789 | -2.269148 | -0.115901 |
| S | 2.718361 | 2.778307 | -0.066216 |
| C | 6.050206 | -0.340887 | -0.627755 |
| C | 6.667294 | -1.532929 | -0.225821 |
| C | 6.678487 | 0.875417 | -0.326613 |
| C | 7.889808 | -1.509868 | 0.447175 |
| H | 6.180346 | -2.485119 | -0.415949 |
| C | 7.900282 | 0.900471 | 0.347217 |
| C | 8.512039 | -0.293330 | 0.733132 |
| H | 8.352611 | -2.444096 | 0.754269 |
| O | -3.117476 | -4.553983 | -0.823949 |
| O | -1.169882 | -0.734447 | -0.036351 |
| C | -3.842671 | -3.561849 | -0.808848 |
| C | -3.405369 | -2.177234 | -0.506119 |
| O | -5.169617 | -3.619967 | -0.992493 |
| C | -5.719151 | -4.936053 | -1.183139 |
| H | -6.789508 | -4.781517 | -1.316857 |
| H | -5.283495 | -5.411533 | -2.065830 |
| H | -5.526808 | -5.559265 | -0.306554 |
| S | -1.613293 | -2.120670 | -0.343986 |
| C | -0.761507 | -2.714483 | -1.822988 |
| H | 0.307437 | -2.697601 | -1.587207 |
| H | -1.011063 | -2.013948 | -2.622984 |
| H | -1.131413 | -3.718647 | -2.036255 |
| C | -1.111267 | -3.253224 | 0.959964 |
| H | -0.036874 | -3.095618 | 1.076981 |
| H | -1.362315 | -4.265715 | 0.646883 |
| H | -1.689499 | -2.960089 | 1.843947 |
| C | -3.910431 | -1.057712 | -1.375656 |
| C | -4.134781 | -1.252644 | -2.750456 |
| C | -4.151711 | 0.210293 | -0.827421 |
| C | -4.599783 | -0.211223 | -3.550395 |
| H | -3.964019 | -2.231102 | -3.192926 |
| C | -4.606454 | 1.256678 | -1.631412 |
| H | -3.992351 | 0.372809 | 0.232278 |
| C | -4.832155 | 1.048226 | -2.992279 |
| H | -4.776966 | -0.381797 | -4.608941 |
| H | -4.776690 | 2.235012 | -1.191984 |
| H | -5.189505 | 1.862613 | -3.616655 |
| C | -4.560438 | 0.879102 | 2.765163 |
| C | -4.171616 | 2.181587 | 3.085191 |
| C | -2.875554 | 2.442649 | 3.537156 |
| C | -1.974648 | 1.383008 | 3.671033 |
| C | -2.361922 | 0.081286 | 3.348115 |
| C | -3.660041 | -0.196993 | 2.880949 |
| H | -5.573014 | 0.679844 | 2.426135 |
| H | -4.886370 | 2.994890 | 2.983949 |
| H | -2.573999 | 3.455791 | 3.788599 |

| | | | |
|---|---|---|---|
| H | -0.966731 | 1.569068 | 4.035569 |
| H | -1.661123 | -0.739006 | 3.476442 |
| S | -4.169084 | -1.862325 | 2.479507 |
| H | -3.774003 | -1.955363 | 0.827850 |
| H | 6.199603 | 1.808898 | -0.612766 |
| H | 8.368459 | 1.853640 | 0.577718 |
| H | 9.461549 | -0.275896 | 1.261121 |
| C | 2.972021 | -0.682679 | 2.382800 |
| C | -0.560089 | 3.248444 | -0.408738 |
| C | 0.042392 | 4.242460 | -1.192126 |
| C | -1.926281 | 3.345193 | -0.086858 |
| C | -0.743221 | 5.303769 | -1.624064 |
| H | 1.088921 | 4.203124 | -1.452776 |
| C | -2.653877 | 4.421798 | -0.564097 |
| H | -2.413995 | 2.607259 | 0.540347 |
| C | -2.097441 | 5.431326 | -1.339530 |
| H | -2.682350 | 6.268964 | -1.698324 |
| F | -0.159500 | 6.257019 | -2.378816 |
| F | -3.972810 | 4.486448 | -0.266562 |
| C | 1.809200 | -1.554880 | 2.890258 |
| H | 1.773461 | -2.516454 | 2.372475 |
| H | 1.938928 | -1.750551 | 3.961189 |
| H | 0.842100 | -1.053777 | 2.767175 |
| C | 3.050574 | 0.595916 | 3.241522 |
| H | 3.884820 | 1.235915 | 2.934446 |
| H | 2.131751 | 1.186343 | 3.166156 |
| H | 3.195116 | 0.329900 | 4.295218 |
| C | 4.289298 | -1.467205 | 2.527381 |
| H | 4.275104 | -2.396746 | 1.946120 |
| H | 5.151525 | -0.876360 | 2.202147 |
| H | 4.446637 | -1.739192 | 3.577598 |

TS Conformation 27

B3LYP/6-31G(d) Energy = -3411.890249
M06-2X/def2tzvpp/IEFPCM(chloroform) Energy = -3411.894173
M06-2X/def2tzvpp/IEFPCM(chloroform)//B3LYP/6-31G(d) Quasiharmonic Free Energy = -3411.167706

Frequencies (Top 3 out of 291)

1. -566.5060 cm-1
2. 7.8873 cm-1
3. 10.4492 cm-1

B3LYP/6-31G(d) Molecular Geometry in Cartesian Coordinates

| | | | |
|---|---|---|---|
| C | 3.867441 | -0.702284 | 0.458114 |
| C | 3.864797 | 0.623690 | 1.246191 |
| H | 4.529759 | 1.350203 | 0.779282 |
| C | 2.218898 | 2.335196 | 0.431096 |
| N | 0.861822 | 2.480026 | 0.287661 |
| H | 0.314164 | 1.698036 | 0.635816 |
| N | 2.523756 | 1.173449 | 1.092744 |
| H | 1.769200 | 0.509179 | 1.246930 |
| C | 4.748516 | -2.090574 | -1.376967 |
| C | 5.946704 | -0.018718 | -0.828209 |
| C | 6.033156 | -1.955057 | -2.236884 |
| H | 4.820835 | -2.959028 | -0.711636 |
| C | 6.941058 | -1.011868 | -1.438296 |
| H | 5.677802 | 0.773287 | -1.538644 |
| H | 6.329112 | 0.452903 | 0.076160 |

| | | | |
|---|---|---|---|
| H | 5.776887 | -1.503589 | -3.201520 |
| H | 6.483414 | -2.930829 | -2.439599 |
| H | 7.696037 | -0.516575 | -2.055766 |
| H | 7.459105 | -1.555606 | -0.639218 |
| N | 4.774232 | -0.868033 | -0.533712 |
| O | 3.003259 | -1.564501 | 0.693030 |
| S | 3.392389 | 3.424264 | -0.100781 |
| C | 3.497435 | -2.239845 | -2.227344 |
| C | 2.993524 | -3.518317 | -2.493269 |
| C | 2.875923 | -1.132932 | -2.820536 |
| C | 1.891697 | -3.690886 | -3.334015 |
| H | 3.464215 | -4.385865 | -2.034972 |
| C | 1.768935 | -1.301968 | -3.653005 |
| C | 1.272949 | -2.581954 | -3.914425 |
| H | 1.512152 | -4.690606 | -3.528297 |
| O | -1.875163 | -4.252213 | 1.037137 |
| O | -0.227813 | -0.223158 | 1.192345 |
| C | -2.684768 | -3.342627 | 1.191347 |
| C | -2.370964 | -1.898691 | 1.042702 |
| O | -3.992863 | -3.543574 | 1.400935 |
| C | -4.427961 | -4.916894 | 1.360147 |
| H | -5.500482 | -4.881348 | 1.548574 |
| H | -4.222351 | -5.347559 | 0.377612 |
| H | -3.918357 | -5.503377 | 2.128593 |
| S | -0.565576 | -1.666196 | 1.043826 |
| C | 0.273183 | -2.586166 | 2.354714 |
| H | 1.340557 | -2.412788 | 2.192880 |
| H | -0.004478 | -3.636185 | 2.268599 |
| H | -0.062270 | -2.151619 | 3.298866 |
| C | 0.082882 | -2.347372 | -0.485853 |
| H | 1.169554 | -2.238459 | -0.438699 |
| H | -0.238738 | -3.387654 | -0.543177 |
| H | -0.355765 | -1.755189 | -1.293333 |
| C | -3.050267 | -0.870485 | 1.901126 |
| C | -3.404294 | 0.363699 | 1.336505 |
| C | -3.323469 | -1.102358 | 3.258870 |
| C | -4.015382 | 1.348426 | 2.113805 |
| H | -3.215590 | 0.539082 | 0.282314 |
| C | -3.942030 | -0.121982 | 4.032495 |
| H | -3.070785 | -2.060771 | 3.706040 |
| C | -4.282945 | 1.107334 | 3.462410 |
| H | -4.273269 | 2.300914 | 1.660507 |
| H | -4.155564 | -0.316268 | 5.080111 |
| H | -4.760933 | 1.872735 | 4.067818 |
| C | -5.580754 | -1.043890 | -1.219108 |
| C | -6.812765 | -0.395958 | -1.308475 |
| C | -7.013833 | 0.623670 | -2.241510 |
| C | -5.960732 | 0.990413 | -3.082422 |
| C | -4.723693 | 0.352898 | -2.989106 |
| C | -4.509534 | -0.678544 | -2.056222 |
| H | -5.439886 | -1.842176 | -0.495690 |
| H | -7.622788 | -0.694814 | -0.646307 |
| H | -7.975985 | 1.124346 | -2.312311 |
| H | -6.099309 | 1.783871 | -3.813698 |
| H | -3.906982 | 0.650030 | -3.640761 |
| S | -2.933197 | -1.504854 | -1.980157 |
| H | -2.656019 | -1.677050 | -0.224689 |
| H | 3.246735 | -0.131398 | -2.617210 |
| H | 1.287756 | -0.432956 | -4.092929 |
| H | 0.405329 | -2.710327 | -4.554899 |
| C | 4.295269 | 0.483577 | 2.749483 |
| C | 0.046504 | 3.545561 | -0.141495 |

| | | | |
|---|---|---|---|
| C | 0.479825 | 4.645590 | -0.893098 |
| C | -1.310506 | 3.434928 | 0.216785 |
| C | -0.461165 | 5.603269 | -1.253446 |
| H | 1.512099 | 4.760442 | -1.186506 |
| C | -2.195814 | 4.421869 | -0.180350 |
| H | -1.671046 | 2.598370 | 0.805223 |
| C | -1.808033 | 5.532434 | -0.920288 |
| H | -2.513915 | 6.296395 | -1.221079 |
| F | -0.040526 | 6.660425 | -1.976863 |
| F | -3.494381 | 4.305142 | 0.178300 |
| C | 3.338361 | -0.413181 | 3.557453 |
| H | 3.355972 | -1.446988 | 3.201939 |
| H | 3.634824 | -0.410042 | 4.613133 |
| H | 2.307336 | -0.044935 | 3.506726 |
| C | 4.311623 | 1.892198 | 3.375173 |
| H | 4.988489 | 2.564042 | 2.836846 |
| H | 3.315833 | 2.345363 | 3.360964 |
| H | 4.643595 | 1.834834 | 4.418737 |
| C | 5.715835 | -0.111613 | 2.816358 |
| H | 5.769253 | -1.106241 | 2.356961 |
| H | 6.449144 | 0.537788 | 2.323009 |
| H | 6.029976 | -0.217187 | 3.861019 |

TS Conformation 28

B3LYP/6-31G(d) Energy = -3411.88994
M06-2X/def2tzvpp/IEFPCM(chloroform) Energy = -3411.894047
M06-2X/def2tzvpp/IEFPCM(chloroform)//B3LYP/6-31G(d) Quasiharmonic Free Energy = -3411.167671

Frequencies (Top 3 out of 291)

1. -918.9963 cm-1
2. 6.8938 cm-1
3. 10.6783 cm-1

B3LYP/6-31G(d) Molecular Geometry in Cartesian Coordinates

| | | | |
|---|---|---|---|
| C | 2.745793 | -1.605181 | -0.135963 |
| C | 3.106866 | -0.474587 | 0.850486 |
| H | 3.961553 | 0.093434 | 0.489254 |
| C | 1.962146 | 1.702248 | 0.310860 |
| N | 0.702966 | 2.249252 | 0.317103 |
| H | -0.056948 | 1.611203 | 0.539349 |
| N | 1.974005 | 0.446260 | 0.844152 |
| H | 1.079014 | 0.035390 | 1.097881 |
| C | 4.614333 | -0.916092 | -1.736191 |
| C | 3.053334 | -2.722871 | -2.291561 |
| C | 4.703023 | -1.280965 | -3.245557 |
| H | 4.281411 | 0.120824 | -1.630880 |
| C | 3.365514 | -1.954905 | -3.577253 |
| H | 3.609632 | -3.669165 | -2.245484 |
| H | 1.998377 | -2.946686 | -2.135593 |
| H | 5.527584 | -1.983852 | -3.406427 |
| H | 4.907273 | -0.392220 | -3.849024 |
| H | 2.587085 | -1.204794 | -3.759762 |
| H | 3.422106 | -2.605960 | -4.455051 |
| N | 3.520997 | -1.809104 | -1.232286 |
| O | 1.716504 | -2.278902 | 0.058848 |
| S | 3.337929 | 2.476244 | -0.295327 |
| C | 5.946000 | -1.077961 | -1.022624 |
| C | 6.636137 | 0.055191 | -0.576789 |

| | | | |
|---|---|---|---|
| C | 6.531073 | -2.339859 | -0.842852 |
| C | 7.886220 | -0.068541 | 0.036203 |
| H | 6.184953 | 1.036843 | -0.698038 |
| C | 7.775526 | -2.465353 | -0.228517 |
| C | 8.458716 | -1.327928 | 0.212190 |
| H | 8.407271 | 0.821438 | 0.378719 |
| O | -3.671285 | -3.355623 | -0.050842 |
| O | -0.950328 | -0.242385 | 1.274016 |
| C | -4.184353 | -2.416596 | 0.553983 |
| C | -3.467913 | -1.195137 | 0.989516 |
| O | -5.493986 | -2.355464 | 0.840900 |
| C | -6.293243 | -3.434828 | 0.320512 |
| H | -6.276329 | -3.416619 | -0.771993 |
| H | -5.918914 | -4.397965 | 0.675502 |
| H | -7.300164 | -3.249463 | 0.694307 |
| S | -1.693596 | -1.425778 | 0.752476 |
| C | -1.073005 | -2.923158 | 1.549741 |
| H | -0.015127 | -2.990663 | 1.281723 |
| H | -1.660699 | -3.763120 | 1.177938 |
| H | -1.207686 | -2.775843 | 2.623456 |
| C | -1.362219 | -1.660001 | -1.000967 |
| H | -0.280545 | -1.801751 | -1.073392 |
| H | -1.929027 | -2.527082 | -1.338931 |
| H | -1.709691 | -0.742918 | -1.486715 |
| C | -3.760939 | -0.596185 | 2.338055 |
| C | -4.110049 | -1.403110 | 3.435154 |
| C | -3.669965 | 0.791721 | 2.523824 |
| C | -4.374134 | -0.836196 | 4.679911 |
| H | -4.197224 | -2.478929 | 3.307761 |
| C | -3.926329 | 1.356813 | 3.773006 |
| H | -3.410133 | 1.430094 | 1.687086 |
| C | -4.279087 | 0.546754 | 4.852639 |
| H | -4.651664 | -1.473786 | 5.514930 |
| H | -3.855293 | 2.433505 | 3.898440 |
| H | -4.482747 | 0.989533 | 5.823837 |
| C | -5.808897 | -0.659316 | -2.219164 |
| C | -6.368043 | -1.444096 | -3.228838 |
| C | -5.721768 | -1.583812 | -4.459467 |
| C | -4.509253 | -0.924482 | -4.669528 |
| C | -3.942748 | -0.145154 | -3.659770 |
| C | -4.579714 | -0.002036 | -2.414131 |
| H | -6.326987 | -0.536399 | -1.272586 |
| H | -7.324033 | -1.935157 | -3.058753 |
| H | -6.162259 | -2.189936 | -5.246589 |
| H | -3.999347 | -1.014916 | -5.626034 |
| H | -3.003166 | 0.372120 | -3.834393 |
| S | -3.865123 | 1.011806 | -1.131117 |
| H | -3.704503 | -0.213188 | 0.032749 |
| H | 6.004535 | -3.231613 | -1.174737 |
| H | 8.213373 | -3.450714 | -0.091792 |
| H | 9.429335 | -1.425964 | 0.690838 |
| C | 3.458079 | -0.961609 | 2.303397 |
| C | 0.271813 | 3.549032 | -0.039965 |
| C | 1.021291 | 4.698104 | 0.243211 |
| C | -0.998916 | 3.657129 | -0.625497 |
| C | 0.481080 | 5.931801 | -0.092255 |
| H | 1.997710 | 4.642383 | 0.701531 |
| C | -1.488081 | 4.922458 | -0.923149 |
| H | -1.598556 | 2.784424 | -0.868957 |
| C | -0.771439 | 6.086958 | -0.674084 |
| H | -1.168225 | 7.063729 | -0.921854 |
| F | 1.201644 | 7.039859 | 0.178816 |

| | | | |
|---|---|---|---|
| F | -2.705026 | 5.027385 | -1.488205 |
| C | 3.937839 | 0.269291 | 3.099283 |
| H | 3.155030 | 1.031103 | 3.169866 |
| H | 4.214154 | -0.027496 | 4.118003 |
| H | 4.813998 | 0.730831 | 2.630941 |
| C | 2.260046 | -1.595910 | 3.036021 |
| H | 1.942244 | -2.523611 | 2.554027 |
| H | 2.550034 | -1.830775 | 4.067124 |
| H | 1.400914 | -0.917595 | 3.088288 |
| C | 4.599998 | -1.992956 | 2.237264 |
| H | 4.309525 | -2.887378 | 1.673692 |
| H | 5.498354 | -1.575423 | 1.773731 |
| H | 4.865466 | -2.315344 | 3.251035 |

TS Conformation 29

B3LYP/6-31G(d) Energy = -3411.887602
M06-2X/def2tzvpp/IEFPCM(chloroform) Energy = -3411.894193
M06-2X/def2tzvpp/IEFPCM(chloroform)//B3LYP/6-31G(d) Quasiharmonic Free Energy = -3411.167659

Frequencies (Top 3 out of 291)

1. -1003.3582 cm-1
2. 12.0144 cm-1
3. 14.7794 cm-1

B3LYP/6-31G(d) Molecular Geometry in Cartesian Coordinates

| | | | |
|---|---|---|---|
| C | -3.026146 | -1.782304 | -0.593619 |
| C | -2.953764 | -1.036132 | -1.944476 |
| H | -3.854887 | -0.443325 | -2.096309 |
| C | -2.036073 | 1.271031 | -1.607459 |
| N | -0.841183 | 1.939419 | -1.525301 |
| H | 0.008225 | 1.403940 | -1.700534 |
| N | -1.869643 | -0.062765 | -1.843503 |
| H | -0.939281 | -0.452494 | -1.725593 |
| C | -4.233145 | -2.308358 | 1.481777 |
| C | -5.477609 | -1.162392 | -0.301746 |
| C | -5.688026 | -2.020338 | 1.927928 |
| H | -4.076102 | -3.389233 | 1.371737 |
| C | -6.469063 | -1.888335 | 0.615456 |
| H | -5.514833 | -0.074393 | -0.171349 |
| H | -5.656407 | -1.381717 | -1.355800 |
| H | -5.716466 | -1.074607 | 2.480899 |
| H | -6.067884 | -2.802961 | 2.590986 |
| H | -7.408727 | -1.339105 | 0.726503 |
| H | -6.702055 | -2.878586 | 0.205549 |
| N | -4.169426 | -1.690563 | 0.136659 |
| O | -2.039314 | -2.392268 | -0.168812 |
| S | -3.540193 | 2.028727 | -1.479371 |
| C | -3.205603 | -1.779583 | 2.468978 |
| C | -2.832361 | -0.429334 | 2.490412 |
| C | -2.666581 | -2.643326 | 3.429059 |
| C | -1.929411 | 0.041373 | 3.444517 |
| H | -3.233230 | 0.251691 | 1.743390 |
| C | -1.774371 | -2.172087 | 4.394427 |
| C | -1.399915 | -0.827615 | 4.402565 |
| H | -1.628644 | 1.085419 | 3.435622 |
| O | 1.427915 | 0.044105 | -1.377634 |
| O | 1.171607 | -3.187820 | -0.911159 |
| C | 2.611503 | -0.195962 | -1.125350 |

| | | | |
|---|---|---|---|
| C | 3.111569 | -1.262673 | -0.230991 |
| O | 3.624104 | 0.536011 | -1.612602 |
| C | 3.273924 | 1.633241 | -2.472403 |
| H | 4.224209 | 2.046990 | -2.809013 |
| H | 2.686883 | 1.280285 | -3.324502 |
| H | 2.706079 | 2.386583 | -1.920391 |
| S | 1.784890 | -2.392300 | 0.178368 |
| C | 0.581098 | -1.466071 | 1.153448 |
| H | 1.076763 | -1.150123 | 2.074025 |
| H | 0.275828 | -0.608838 | 0.559033 |
| H | -0.270509 | -2.129265 | 1.315487 |
| C | 2.533335 | -3.480620 | 1.407817 |
| H | 3.304646 | -4.063876 | 0.903740 |
| H | 2.951681 | -2.864197 | 2.209252 |
| H | 1.731139 | -4.127689 | 1.769296 |
| C | 4.371356 | -1.984201 | -0.672512 |
| C | 5.594229 | -1.703060 | -0.045347 |
| C | 4.346644 | -2.911380 | -1.729880 |
| C | 6.763844 | -2.339302 | -0.463923 |
| H | 5.628870 | -0.977786 | 0.761065 |
| C | 5.516254 | -3.554921 | -2.134475 |
| H | 3.408963 | -3.131244 | -2.231887 |
| C | 6.728568 | -3.269515 | -1.503402 |
| H | 7.704312 | -2.106399 | 0.028080 |
| H | 5.479778 | -4.271771 | -2.950407 |
| H | 7.640588 | -3.766014 | -1.823964 |
| C | 2.784895 | 2.184252 | 1.644817 |
| C | 1.981827 | 3.323737 | 1.699843 |
| C | 1.146729 | 3.552353 | 2.796075 |
| C | 1.131218 | 2.626899 | 3.842233 |
| C | 1.921060 | 1.477194 | 3.784216 |
| C | 2.756982 | 1.233285 | 2.680508 |
| H | 3.453982 | 2.029717 | 0.802732 |
| H | 2.012917 | 4.042573 | 0.884929 |
| H | 0.516716 | 4.436868 | 2.829791 |
| H | 0.499596 | 2.799234 | 4.711046 |
| H | 1.900289 | 0.758931 | 4.599162 |
| S | 3.774683 | -0.232427 | 2.625228 |
| H | 3.402034 | -0.705219 | 1.052321 |
| H | -2.944523 | -3.695383 | 3.418048 |
| H | -1.367996 | -2.856998 | 5.134478 |
| H | -0.697393 | -0.458487 | 5.144607 |
| C | -2.777758 | -1.980725 | -3.182436 |
| C | -0.632750 | 3.313235 | -1.251766 |
| C | -1.183395 | 3.933154 | -0.124860 |
| C | 0.229491 | 4.024630 | -2.096811 |
| C | -0.851623 | 5.255924 | 0.126683 |
| H | -1.840332 | 3.398752 | 0.546840 |
| C | 0.528826 | 5.347540 | -1.791278 |
| H | 0.634918 | 3.572012 | -2.994581 |
| C | 0.004114 | 5.997936 | -0.682303 |
| H | 0.246741 | 7.029480 | -0.458419 |
| F | -1.346754 | 5.842902 | 1.236771 |
| F | 1.355213 | 6.027733 | -2.611451 |
| C | -2.960580 | -1.132765 | -4.456812 |
| H | -2.227405 | -0.320816 | -4.501857 |
| H | -2.829031 | -1.756169 | -5.349231 |
| H | -3.959958 | -0.683775 | -4.502517 |
| C | -3.871498 | -3.066640 | -3.138985 |
| H | -4.878072 | -2.629803 | -3.127390 |
| H | -3.803691 | -3.701684 | -4.029647 |
| H | -3.764946 | -3.716272 | -2.262954 |

| | | | |
|---|---|---|---|
| C | -1.395336 | -2.661866 | -3.217563 |
| H | -1.193484 | -3.227644 | -2.306059 |
| H | -1.346242 | -3.344375 | -4.074849 |
| H | -0.586759 | -1.932505 | -3.344307 |

TS Conformation 30

B3LYP/6-31G(d) Energy = -3411.893816
M06-2X/def2tzvpp/IEFPCM(chloroform) Energy = -3411.894619
M06-2X/def2tzvpp/IEFPCM(chloroform)//B3LYP/6-31G(d) Quasiharmonic Free Energy = -3411.167608

Frequencies (Top 3 out of 291)

1. -992.0244 cm-1
2. 9.2326 cm-1
3. 11.7161 cm-1

B3LYP/6-31G(d) Molecular Geometry in Cartesian Coordinates

| | | | |
|---|---|---|---|
| C | 3.486227 | -1.324853 | 1.063116 |
| C | 3.798171 | 0.054587 | 1.680574 |
| H | 4.707705 | 0.482207 | 1.257330 |
| C | 2.840971 | 2.101341 | 0.562522 |
| N | 1.611730 | 2.570497 | 0.167477 |
| H | 0.844816 | 1.929226 | 0.349040 |
| N | 2.707449 | 0.925506 | 1.257030 |
| H | 1.810650 | 0.450866 | 1.188728 |
| C | 4.124831 | -3.224010 | -0.366000 |
| C | 5.801112 | -1.474267 | 0.025026 |
| C | 5.472039 | -3.593620 | -1.040786 |
| H | 3.895854 | -3.930369 | 0.441490 |
| C | 6.524940 | -2.793095 | -0.265811 |
| H | 5.857725 | -0.787539 | -0.829448 |
| H | 6.203009 | -0.961429 | 0.898117 |
| H | 5.451646 | -3.274091 | -2.088695 |
| H | 5.643180 | -4.673685 | -1.028635 |
| H | 7.451524 | -2.643238 | -0.827565 |
| H | 6.776238 | -3.293826 | 0.676798 |
| N | 4.405830 | -1.904447 | 0.250375 |
| O | 2.387117 | -1.858351 | 1.275767 |
| S | 4.327010 | 2.841778 | 0.275216 |
| C | 2.969268 | -3.240332 | -1.353736 |
| C | 2.646924 | -2.128080 | -2.140247 |
| C | 2.258467 | -4.431327 | -1.547303 |
| C | 1.633822 | -2.205352 | -3.096912 |
| H | 3.178105 | -1.191817 | -1.992762 |
| C | 1.252142 | -4.515131 | -2.511495 |
| C | 0.934860 | -3.399883 | -3.290356 |
| H | 1.385035 | -1.328822 | -3.688485 |
| O | -2.615124 | 1.476951 | 1.004758 |
| O | 0.082735 | 0.112827 | 0.033129 |
| C | -3.072104 | 0.816258 | 0.074674 |
| C | -2.628991 | -0.559994 | -0.274720 |
| O | -4.065786 | 1.233862 | -0.715530 |
| C | -4.619056 | 2.528898 | -0.402405 |
| H | -3.858838 | 3.305646 | -0.514401 |
| H | -5.424292 | 2.672077 | -1.122582 |
| H | -5.011147 | 2.532822 | 0.616897 |
| S | -1.017131 | -0.811416 | 0.445743 |
| C | -1.172938 | -0.844372 | 2.242016 |
| H | -2.014278 | -1.501499 | 2.492363 |

| | | | |
|---|---|---|---|
| H | -1.371164 | 0.181032 | 2.550476 |
| H | -0.217360 | -1.215991 | 2.620038 |
| C | -0.575746 | -2.505167 | 0.044084 |
| H | -1.333393 | -3.169975 | 0.467007 |
| H | 0.417222 | -2.660653 | 0.475045 |
| H | -0.534960 | -2.591528 | -1.042070 |
| C | -2.670698 | -0.972220 | -1.729122 |
| C | -3.381620 | -2.115866 | -2.120918 |
| C | -2.011844 | -0.210762 | -2.710557 |
| C | -3.433275 | -2.487296 | -3.465977 |
| H | -3.908306 | -2.698698 | -1.370862 |
| C | -2.063733 | -0.585240 | -4.052113 |
| H | -1.462543 | 0.680335 | -2.419516 |
| C | -2.775177 | -1.726268 | -4.433079 |
| H | -3.996305 | -3.370098 | -3.756758 |
| H | -1.556596 | 0.018230 | -4.800194 |
| H | -2.823184 | -2.013705 | -5.480223 |
| C | -6.975795 | -2.012248 | 1.582207 |
| C | -8.081235 | -1.230403 | 1.917489 |
| C | -7.909629 | 0.033519 | 2.485821 |
| C | -6.616269 | 0.502979 | 2.722461 |
| C | -5.507291 | -0.281222 | 2.399401 |
| C | -5.669820 | -1.550461 | 1.817393 |
| H | -7.115793 | -2.990574 | 1.132012 |
| H | -9.082364 | -1.610693 | 1.728066 |
| H | -8.771841 | 0.642423 | 2.744339 |
| H | -6.465321 | 1.480502 | 3.175385 |
| H | -4.509248 | 0.094472 | 2.604530 |
| S | -4.277515 | -2.591354 | 1.400881 |
| H | -3.416862 | -1.472690 | 0.499062 |
| H | 2.494528 | -5.301454 | -0.937938 |
| H | 0.713267 | -5.448960 | -2.649221 |
| H | 0.143299 | -3.454877 | -4.032330 |
| C | 3.971535 | 0.005402 | 3.243527 |
| C | 1.165437 | 3.748434 | -0.453423 |
| C | 1.985950 | 4.738080 | -1.011503 |
| C | -0.237202 | 3.890503 | -0.504739 |
| C | 1.374854 | 5.838172 | -1.602120 |
| H | 3.062496 | 4.658904 | -0.987719 |
| C | -0.776545 | 5.009271 | -1.113705 |
| H | -0.897977 | 3.155915 | -0.054435 |
| C | -0.001280 | 6.014501 | -1.680041 |
| H | -0.441986 | 6.884656 | -2.150128 |
| F | 2.167184 | 6.787578 | -2.139673 |
| F | -2.124616 | 5.132383 | -1.156119 |
| C | 5.080308 | -1.005251 | 3.599008 |
| H | 6.041841 | -0.725574 | 3.150921 |
| H | 5.227328 | -1.033309 | 4.684644 |
| H | 4.830488 | -2.023421 | 3.277109 |
| C | 2.670632 | -0.398777 | 3.963163 |
| H | 2.348734 | -1.406269 | 3.687370 |
| H | 2.826311 | -0.370592 | 5.048121 |
| H | 1.860379 | 0.301866 | 3.731244 |
| C | 4.395807 | 1.404943 | 3.729099 |
| H | 5.335559 | 1.725513 | 3.267735 |
| H | 3.638502 | 2.157168 | 3.489272 |
| H | 4.532499 | 1.396195 | 4.817280 |

TS Conformation 31

B3LYP/6-31G(d) Energy = -3411.889596

M06-2X/def2tzvpp/IEFPCM(chloroform) Energy = -3411.893631
M06-2X/def2tzvpp/IEFPCM(chloroform)//B3LYP/6-31G(d) Quasiharmonic Free Energy = -3411.167404

Frequencies (Top 3 out of 291)

1. -542.0293 cm-1
2. 6.8036 cm-1
3. 7.9778 cm-1

B3LYP/6-31G(d) Molecular Geometry in Cartesian Coordinates

| | | | |
|---|---|---|---|
| C | 3.045752 | -1.296258 | -0.410734 |
| C | 2.995476 | -0.553409 | 0.941080 |
| H | 3.882439 | 0.063988 | 1.070441 |
| C | 1.942386 | 1.712148 | 0.662636 |
| N | 0.696780 | 2.272503 | 0.497302 |
| H | -0.068064 | 1.606556 | 0.421848 |
| N | 1.863594 | 0.365814 | 0.865088 |
| H | 0.959555 | -0.078454 | 0.724663 |
| C | 5.335095 | -0.338290 | -1.005744 |
| C | 4.113923 | -1.840396 | -2.527333 |
| C | 5.757997 | -0.084396 | -2.474899 |
| H | 5.004860 | 0.600424 | -0.553670 |
| C | 5.427117 | -1.402695 | -3.188312 |
| H | 3.993958 | -2.925614 | -2.454794 |
| H | 3.237881 | -1.454097 | -3.062291 |
| H | 6.809859 | 0.201752 | -2.551821 |
| H | 5.151241 | 0.732699 | -2.883214 |
| H | 6.215798 | -2.140968 | -3.009432 |
| H | 5.320942 | -1.287518 | -4.270883 |
| N | 4.160436 | -1.234033 | -1.178779 |
| O | 2.040585 | -1.910174 | -0.815261 |
| S | 3.402779 | 2.561284 | 0.614138 |
| C | 6.458546 | -0.924581 | -0.162446 |
| C | 6.777971 | -2.288859 | -0.163123 |
| C | 7.240691 | -0.053113 | 0.608006 |
| C | 7.859259 | -2.767519 | 0.578108 |
| H | 6.164855 | -2.988080 | -0.724457 |
| C | 8.322092 | -0.529620 | 1.350397 |
| C | 8.636818 | -1.889416 | 1.335186 |
| H | 8.089791 | -3.829589 | 0.568702 |
| O | -2.939303 | -2.706623 | -2.994070 |
| O | -1.006498 | -0.301262 | 0.084091 |
| C | -3.661105 | -1.915520 | -2.393871 |
| C | -3.255496 | -1.151027 | -1.185730 |
| O | -4.953644 | -1.723205 | -2.683573 |
| C | -5.496708 | -2.571616 | -3.714537 |
| H | -6.538987 | -2.269606 | -3.811746 |
| H | -4.960439 | -2.423527 | -4.655040 |
| H | -5.424496 | -3.619546 | -3.414920 |
| S | -1.451771 | -1.245342 | -0.980588 |
| C | -0.543710 | -0.900651 | -2.506410 |
| H | 0.508607 | -1.092940 | -2.274458 |
| H | -0.929914 | -1.560248 | -3.283574 |
| H | -0.721353 | 0.151229 | -2.741189 |
| C | -1.021650 | -2.940757 | -0.561668 |
| H | -1.522069 | -3.148894 | 0.389384 |
| H | -1.401575 | -3.582429 | -1.357559 |
| H | 0.067586 | -2.956685 | -0.479128 |
| C | -3.748985 | 0.250919 | -0.969306 |
| C | -3.882358 | 1.156702 | -2.036137 |
| C | -4.071558 | 0.673339 | 0.328400 |

| | | | |
|---|---|---|---|
| C | -4.335588 | 2.454495 | -1.809569 |
| H | -3.650913 | 0.837083 | -3.049446 |
| C | -4.526081 | 1.973412 | 0.551779 |
| H | -3.981994 | -0.024065 | 1.154136 |
| C | -4.656485 | 2.865683 | -0.512582 |
| H | -4.431591 | 3.148145 | -2.639802 |
| H | -4.781793 | 2.284810 | 1.560349 |
| H | -4.996583 | 3.881570 | -0.336694 |
| C | -6.554661 | -1.793169 | 1.131172 |
| C | -7.637129 | -1.212558 | 1.791784 |
| C | -7.696605 | -1.204591 | 3.187448 |
| C | -6.653608 | -1.783012 | 3.914235 |
| C | -5.563828 | -2.355297 | 3.257631 |
| C | -5.493366 | -2.372679 | 1.852375 |
| H | -6.522559 | -1.802626 | 0.045131 |
| H | -8.442444 | -0.768427 | 1.210406 |
| H | -8.543706 | -0.756486 | 3.700372 |
| H | -6.684279 | -1.785665 | 5.001748 |
| H | -4.753018 | -2.797562 | 3.829407 |
| S | -4.112835 | -3.139342 | 1.028751 |
| H | -3.661412 | -1.929828 | -0.207087 |
| H | 6.994795 | 1.006209 | 0.631154 |
| H | 8.912757 | 0.161222 | 1.946017 |
| H | 9.475918 | -2.263464 | 1.915335 |
| C | 2.896503 | -1.485893 | 2.201915 |
| C | 0.286875 | 3.607165 | 0.301025 |
| C | -0.957675 | 3.781168 | -0.331305 |
| C | 1.006588 | 4.725144 | 0.743551 |
| C | -1.442285 | 5.066785 | -0.516337 |
| H | -1.543117 | 2.935677 | -0.675522 |
| C | 0.467281 | 5.985272 | 0.519209 |
| H | 1.959932 | 4.627123 | 1.239821 |
| C | -0.753900 | 6.201870 | -0.107376 |
| H | -1.147029 | 7.198237 | -0.266262 |
| F | -2.635140 | 5.226356 | -1.131927 |
| F | 1.163070 | 7.057977 | 0.944909 |
| C | 1.594925 | -2.308767 | 2.248885 |
| H | 0.699954 | -1.676768 | 2.227401 |
| H | 1.537690 | -3.018322 | 1.419767 |
| H | 1.560279 | -2.879298 | 3.184183 |
| C | 2.968698 | -0.583434 | 3.450773 |
| H | 2.129634 | 0.118267 | 3.488406 |
| H | 3.896556 | -0.000445 | 3.470236 |
| H | 2.935942 | -1.197458 | 4.358146 |
| C | 4.095819 | -2.451210 | 2.212445 |
| H | 4.062247 | -3.073237 | 3.114291 |
| H | 5.049619 | -1.914461 | 2.211221 |
| H | 4.085490 | -3.125391 | 1.348285 |

TS Conformation 32 (**TS-1'**)

B3LYP/6-31G(d) Energy = -3411.889981
M06-2X/def2tzvpp/IEFPCM(chloroform) Energy = -3411.894204
M06-2X/def2tzvpp/IEFPCM(chloroform)//B3LYP/6-31G(d) Quasiharmonic Free Energy = -3411.167199

Frequencies (Top 3 out of 291)

1. -840.1967 cm-1
2. 8.5004 cm-1
3. 11.3797 cm-1

B3LYP/6-31G(d) Molecular Geometry in Cartesian Coordinates

| | | | |
|---|---|---|---|
| C | 2.778698 | -1.755633 | 0.703718 |
| C | 2.958353 | -0.687740 | 1.800473 |
| H | 3.955535 | -0.253547 | 1.745025 |
| C | 2.402989 | 1.576997 | 0.886774 |
| N | 1.308965 | 2.304550 | 0.495901 |
| H | 0.425393 | 1.800408 | 0.517631 |
| N | 2.039645 | 0.399052 | 1.483555 |
| H | 1.061501 | 0.128265 | 1.415784 |
| C | 3.712284 | -3.120100 | -1.106707 |
| C | 5.266782 | -1.708727 | 0.177529 |
| C | 5.169548 | -3.591300 | -1.325970 |
| H | 3.077268 | -3.937719 | -0.753156 |
| C | 5.999784 | -2.337514 | -1.016459 |
| H | 5.342805 | -0.618012 | 0.195984 |
| H | 5.651193 | -2.103300 | 1.125638 |
| H | 5.328768 | -3.980066 | -2.334944 |
| H | 5.405197 | -4.389395 | -0.611277 |
| H | 5.985050 | -1.653115 | -1.871320 |
| H | 7.044946 | -2.559569 | -0.783498 |
| N | 3.860751 | -2.136455 | -0.015260 |
| O | 1.648880 | -2.213599 | 0.456896 |
| S | 4.012828 | 2.044912 | 0.662382 |
| C | 3.079767 | -2.536585 | -2.367388 |
| C | 3.123271 | -1.170331 | -2.671275 |
| C | 2.469414 | -3.408497 | -3.280166 |
| C | 2.575296 | -0.689558 | -3.863678 |
| H | 3.568788 | -0.470352 | -1.969812 |
| C | 1.923497 | -2.931412 | -4.471684 |
| C | 1.976028 | -1.567018 | -4.768468 |
| H | 2.618953 | 0.374287 | -4.081102 |
| O | -1.915360 | 1.442791 | -1.935932 |
| O | -0.869302 | 0.237240 | 0.606562 |
| C | -3.005016 | 0.985305 | -1.608570 |
| C | -3.207426 | -0.229960 | -0.764632 |
| O | -4.173614 | 1.547912 | -1.924885 |
| C | -4.097736 | 2.775100 | -2.681442 |
| H | -3.733187 | 2.571229 | -3.692171 |
| H | -5.119107 | 3.152689 | -2.711473 |
| H | -3.430130 | 3.489503 | -2.195567 |
| S | -1.574316 | -0.768090 | -0.233909 |
| C | -1.807868 | -2.275773 | 0.718929 |
| H | -0.798677 | -2.578293 | 1.010268 |
| H | -2.414078 | -2.015412 | 1.591038 |
| H | -2.298873 | -3.031826 | 0.105237 |
| C | -0.535381 | -1.302191 | -1.616940 |
| H | -1.096835 | -2.037382 | -2.197584 |
| H | -0.330204 | -0.408044 | -2.204520 |
| H | 0.373449 | -1.730013 | -1.182925 |
| C | -4.041106 | -1.341298 | -1.359686 |
| C | -3.933381 | -1.672780 | -2.722490 |
| C | -4.949198 | -2.053515 | -0.560924 |
| C | -4.703298 | -2.697686 | -3.269951 |
| H | -3.253147 | -1.113791 | -3.361375 |
| C | -5.713489 | -3.084227 | -1.110160 |
| H | -5.079390 | -1.762526 | 0.477050 |
| C | -5.591096 | -3.411419 | -2.461360 |
| H | -4.612280 | -2.936446 | -4.326075 |
| H | -6.417190 | -3.622435 | -0.481115 |
| H | -6.191981 | -4.210827 | -2.886387 |
| C | -4.237574 | -1.259850 | 3.552911 |

| | | | |
|---|---|---|---|
| C | -3.509826 | -1.856769 | 4.585515 |
| C | -2.370718 | -1.233027 | 5.098746 |
| C | -1.969818 | -0.004249 | 4.566138 |
| C | -2.690944 | 0.589560 | 3.529703 |
| C | -3.842688 | -0.024681 | 2.998745 |
| H | -5.140760 | -1.735601 | 3.179708 |
| H | -3.843337 | -2.806666 | 4.998090 |
| H | -1.809940 | -1.691171 | 5.909260 |
| H | -1.093247 | 0.500536 | 4.966410 |
| H | -2.377783 | 1.547082 | 3.125301 |
| S | -4.784871 | 0.746395 | 1.696319 |
| H | -3.827677 | 0.205142 | 0.371271 |
| H | 2.420615 | -4.472023 | -3.053651 |
| H | 1.455619 | -3.624009 | -5.166717 |
| H | 1.552381 | -1.191910 | -5.696198 |
| C | 2.768236 | -1.231108 | 3.258368 |
| C | 1.179236 | 3.604803 | -0.034755 |
| C | 2.094820 | 4.643046 | 0.181941 |
| C | 0.000030 | 3.846272 | -0.760934 |
| C | 1.802524 | 5.893428 | -0.348616 |
| H | 3.008267 | 4.491655 | 0.737315 |
| C | -0.234835 | 5.119673 | -1.250571 |
| H | -0.712375 | 3.054958 | -0.965706 |
| C | 0.647692 | 6.177492 | -1.068578 |
| H | 0.449106 | 7.166307 | -1.462806 |
| F | 2.683807 | 6.893245 | -0.140152 |
| F | -1.375766 | 5.340499 | -1.944942 |
| C | 3.799471 | -2.350883 | 3.503537 |
| H | 4.826536 | -1.986905 | 3.378128 |
| H | 3.708481 | -2.727273 | 4.528672 |
| H | 3.650112 | -3.202145 | 2.828320 |
| C | 1.354394 | -1.785304 | 3.515218 |
| H | 1.136930 | -2.647610 | 2.879348 |
| H | 1.270726 | -2.103970 | 4.561063 |
| H | 0.577471 | -1.031363 | 3.349157 |
| C | 3.045671 | -0.071033 | 4.234962 |
| H | 2.329954 | 0.744994 | 4.096769 |
| H | 2.963658 | -0.423085 | 5.269991 |
| H | 4.052182 | 0.339757 | 4.094759 |

TS Conformation 33

B3LYP/6-31G(d) Energy = -3411.891446
M06-2X/def2tzvpp/IEFPCM(chloroform) Energy = -3411.893987
M06-2X/def2tzvpp/IEFPCM(chloroform)//B3LYP/6-31G(d) Quasiharmonic Free Energy = -3411.16717

Frequencies (Top 3 out of 291)

1. -732.5786 cm-1
2. 8.5218 cm-1
3. 14.5177 cm-1

B3LYP/6-31G(d) Molecular Geometry in Cartesian Coordinates

| | | | |
|---|---|---|---|
| C | 3.150272 | -1.273175 | -0.329109 |
| C | 3.180623 | -0.331366 | 0.891753 |
| H | 3.953845 | 0.425062 | 0.770009 |
| C | 1.739546 | 1.703471 | 0.576753 |
| N | 0.412811 | 2.052209 | 0.510027 |
| H | -0.253038 | 1.283816 | 0.518813 |
| N | 1.909327 | 0.386141 | 0.890648 |

| | | | |
|---|---|---|---|
| H | 1.078302 | -0.198095 | 0.941709 |
| C | 5.021470 | 0.002870 | -1.501670 |
| C | 3.920717 | -1.941292 | -2.539862 |
| C | 5.142139 | 0.068687 | -3.045365 |
| H | 4.569024 | 0.925911 | -1.129770 |
| C | 4.988526 | -1.391464 | -3.493617 |
| H | 4.041570 | -3.002454 | -2.301989 |
| H | 2.908750 | -1.813491 | -2.943348 |
| H | 6.083744 | 0.525264 | -3.360085 |
| H | 4.319944 | 0.676539 | -3.441353 |
| H | 5.933621 | -1.930319 | -3.368331 |
| H | 4.688925 | -1.487061 | -4.541223 |
| N | 4.061908 | -1.119476 | -1.318632 |
| O | 2.256442 | -2.135564 | -0.429544 |
| S | 3.023426 | 2.767388 | 0.294444 |
| C | 6.362560 | -0.197691 | -0.809963 |
| C | 7.034992 | 0.928490 | -0.315501 |
| C | 6.976627 | -1.451651 | -0.690790 |
| C | 8.295393 | 0.807667 | 0.271494 |
| H | 6.561451 | 1.905136 | -0.383373 |
| C | 8.237310 | -1.574425 | -0.104789 |
| C | 8.902470 | -0.444894 | 0.375611 |
| H | 8.798086 | 1.691901 | 0.654033 |
| O | -2.746774 | -4.043553 | -1.540399 |
| O | -0.823699 | -0.891464 | 0.769254 |
| C | -3.486438 | -3.363984 | -0.832918 |
| C | -3.069307 | -2.160271 | -0.072526 |
| O | -4.806938 | -3.576103 | -0.734210 |
| C | -5.333245 | -4.637684 | -1.554253 |
| H | -5.155483 | -4.423637 | -2.610573 |
| H | -4.865849 | -5.590536 | -1.293781 |
| H | -6.401251 | -4.659723 | -1.340027 |
| S | -1.261619 | -2.032176 | -0.083008 |
| C | -0.464759 | -3.552350 | 0.476926 |
| H | -0.833212 | -4.368385 | -0.145541 |
| H | 0.610055 | -3.383794 | 0.369084 |
| H | -0.750585 | -3.677795 | 1.523509 |
| C | -0.701211 | -1.816315 | -1.781902 |
| H | -1.109673 | -0.861510 | -2.121569 |
| H | 0.391203 | -1.808226 | -1.727987 |
| H | -1.090610 | -2.646296 | -2.371074 |
| C | -3.610203 | -1.920522 | 1.310544 |
| C | -3.852901 | -2.986534 | 2.193852 |
| C | -3.859703 | -0.609329 | 1.741273 |
| C | -4.345257 | -2.747196 | 3.474823 |
| H | -3.677376 | -4.009303 | 1.869053 |
| C | -4.347568 | -0.372566 | 3.026298 |
| H | -3.686382 | 0.216638 | 1.059934 |
| C | -4.590200 | -1.437336 | 3.894692 |
| H | -4.537396 | -3.581288 | 4.144256 |
| H | -4.545533 | 0.647063 | 3.344011 |
| H | -4.973907 | -1.249732 | 4.893904 |
| C | -5.534949 | 2.169883 | -1.306699 |
| C | -6.810370 | 2.600264 | -0.940042 |
| C | -7.819229 | 1.677551 | -0.655543 |
| C | -7.535675 | 0.313415 | -0.747001 |
| C | -6.265632 | -0.123870 | -1.123805 |
| C | -5.241854 | 0.798188 | -1.407212 |
| H | -4.755725 | 2.896560 | -1.510815 |
| H | -7.012757 | 3.666872 | -0.871223 |
| H | -8.810974 | 2.016106 | -0.366889 |
| H | -8.309575 | -0.419391 | -0.528120 |

| | | | |
|---|---|---|---|
| H | -6.061278 | -1.187950 | -1.197707 |
| S | -3.614859 | 0.263878 | -1.911919 |
| H | -3.370334 | -1.117790 | -0.858782 |
| H | 6.459156 | -2.342613 | -1.034899 |
| H | 8.697268 | -2.555415 | -0.018371 |
| H | 9.882325 | -0.541710 | 0.835173 |
| C | 3.456010 | -1.043422 | 2.264256 |
| C | -0.175037 | 3.323187 | 0.297456 |
| C | 0.342032 | 4.490561 | 0.873746 |
| C | -1.354982 | 3.366525 | -0.461280 |
| C | -0.324459 | 5.686358 | 0.647653 |
| H | 1.241231 | 4.479177 | 1.471938 |
| C | -1.976134 | 4.595351 | -0.640766 |
| H | -1.785750 | 2.476672 | -0.914424 |
| C | -1.488733 | 5.780647 | -0.105000 |
| H | -1.989437 | 6.727162 | -0.266976 |
| F | 0.173124 | 6.811534 | 1.200666 |
| F | -3.102479 | 4.652243 | -1.383623 |
| C | 3.583535 | 0.056524 | 3.337982 |
| H | 2.656422 | 0.629954 | 3.435867 |
| H | 3.806947 | -0.394751 | 4.311889 |
| H | 4.388356 | 0.759701 | 3.097069 |
| C | 2.339702 | -2.020987 | 2.678392 |
| H | 2.273594 | -2.867434 | 1.990435 |
| H | 2.554085 | -2.413110 | 3.679757 |
| H | 1.359802 | -1.532508 | 2.724805 |
| C | 4.786207 | -1.813962 | 2.174219 |
| H | 4.741092 | -2.619508 | 1.431914 |
| H | 5.619237 | -1.155653 | 1.908605 |
| H | 5.016843 | -2.272256 | 3.143039 |

TS Conformation 34

B3LYP/6-31G(d) Energy = -3411.890729
M06-2X/def2tzvpp/IEFPCM(chloroform) Energy = -3411.893922
M06-2X/def2tzvpp/IEFPCM(chloroform)//B3LYP/6-31G(d) Quasiharmonic Free Energy = -3411.167142

Frequencies (Top 3 out of 291)

1. -794.3787 cm-1
2. 12.2095 cm-1
3. 13.6714 cm-1

B3LYP/6-31G(d) Molecular Geometry in Cartesian Coordinates

| | | | |
|---|---|---|---|
| C | -2.530715 | -1.464792 | -0.520098 |
| C | -2.699980 | -0.247164 | -1.449513 |
| H | -3.527312 | 0.378912 | -1.119562 |
| C | -1.413095 | 1.814074 | -0.765993 |
| N | -0.108384 | 2.192501 | -0.560327 |
| H | 0.574990 | 1.461154 | -0.734106 |
| N | -1.484418 | 0.545409 | -1.271711 |
| H | -0.620519 | 0.010531 | -1.317492 |
| C | -4.259348 | -0.554824 | 1.112138 |
| C | -3.054082 | -2.659046 | 1.542765 |
| C | -4.249823 | -0.884097 | 2.626927 |
| H | -3.844543 | 0.443500 | 0.950118 |
| C | -4.058829 | -2.406018 | 2.672248 |
| H | -3.159666 | -3.635401 | 1.060931 |
| H | -2.020988 | -2.575490 | 1.901499 |
| H | -5.160539 | -0.540330 | 3.123407 |

| | | | |
|---|---|---|---|
| H | -3.392681 | -0.384927 | 3.094589 |
| H | -5.006430 | -2.916754 | 2.469904 |
| H | -3.686962 | -2.761331 | 3.637387 |
| N | -3.321070 | -1.574869 | 0.573234 |
| O | -1.633587 | -2.298930 | -0.743777 |
| S | -2.775049 | 2.765300 | -0.466297 |
| C | -5.654185 | -0.604151 | 0.502833 |
| C | -6.247403 | -1.794183 | 0.061905 |
| C | -6.394413 | 0.584053 | 0.427427 |
| C | -7.554817 | -1.798708 | -0.428001 |
| H | -5.679524 | -2.719979 | 0.080142 |
| C | -7.700765 | 0.581304 | -0.062232 |
| C | -8.286893 | -0.611894 | -0.488553 |
| H | -7.998721 | -2.730913 | -0.767542 |
| O | 3.628473 | -4.133441 | -0.137221 |
| O | 1.383942 | -0.467036 | -0.882329 |
| C | 4.281171 | -3.095412 | -0.197396 |
| C | 3.709874 | -1.724980 | -0.289780 |
| O | 5.614062 | -3.050938 | -0.093694 |
| C | 6.266155 | -4.307503 | 0.171620 |
| H | 7.328783 | -4.071914 | 0.220339 |
| H | 5.918459 | -4.718690 | 1.122038 |
| H | 6.063350 | -5.022149 | -0.629988 |
| S | 1.937552 | -1.823683 | -0.618914 |
| C | 1.133910 | -2.623893 | 0.775389 |
| H | 1.292874 | -1.980752 | 1.644412 |
| H | 1.610353 | -3.595582 | 0.908231 |
| H | 0.076212 | -2.696827 | 0.503283 |
| C | 1.535800 | -2.890170 | -2.023697 |
| H | 1.961082 | -2.412050 | -2.908489 |
| H | 1.974164 | -3.871488 | -1.840530 |
| H | 0.444026 | -2.918668 | -2.073542 |
| C | 4.373194 | -0.713740 | -1.187471 |
| C | 4.851639 | -1.066383 | -2.460455 |
| C | 4.506134 | 0.613482 | -0.755994 |
| C | 5.446680 | -0.111922 | -3.283360 |
| H | 4.775778 | -2.096616 | -2.801283 |
| C | 5.094649 | 1.571217 | -1.583883 |
| H | 4.177697 | 0.877699 | 0.244311 |
| C | 5.562063 | 1.210948 | -2.848722 |
| H | 5.818237 | -0.399781 | -4.263085 |
| H | 5.175749 | 2.597389 | -1.238022 |
| H | 6.020948 | 1.956356 | -3.492606 |
| C | 1.722681 | 0.673226 | 2.713010 |
| C | 0.429214 | 0.952195 | 3.156807 |
| C | -0.184126 | 0.134929 | 4.110282 |
| C | 0.514712 | -0.966335 | 4.612386 |
| C | 1.804764 | -1.252414 | 4.162016 |
| C | 2.439573 | -0.439618 | 3.199432 |
| H | 2.198281 | 1.334533 | 1.994513 |
| H | -0.094160 | 1.821376 | 2.765323 |
| H | -1.183031 | 0.366119 | 4.472278 |
| H | 0.056832 | -1.605420 | 5.364601 |
| H | 2.343753 | -2.108630 | 4.557850 |
| S | 4.089321 | -0.816715 | 2.641964 |
| H | 3.776603 | -1.280781 | 0.983042 |
| H | -5.937716 | 1.518551 | 0.744537 |
| H | -8.256361 | 1.513489 | -0.118830 |
| H | -9.303005 | -0.615423 | -0.873517 |
| C | -2.978364 | -0.608289 | -2.951700 |
| C | 0.501589 | 3.413117 | -0.213141 |
| C | -0.160330 | 4.534213 | 0.305401 |

| | | | |
|---|---|---|---|
| C | 1.897646 | 3.452384 | -0.399803 |
| C | 0.597321 | 5.658078 | 0.614977 |
| H | -1.228369 | 4.540083 | 0.460331 |
| C | 2.591458 | 4.602333 | -0.063577 |
| H | 2.434879 | 2.606001 | -0.813853 |
| C | 1.974255 | 5.736816 | 0.448754 |
| H | 2.532975 | 6.628151 | 0.704955 |
| F | -0.044688 | 6.732907 | 1.113301 |
| F | 3.930533 | 4.625459 | -0.252240 |
| C | -3.306915 | 0.702711 | -3.694135 |
| H | -2.471483 | 1.408640 | -3.650239 |
| H | -3.517203 | 0.491849 | -4.749495 |
| H | -4.183452 | 1.198966 | -3.264390 |
| C | -4.197499 | -1.547428 | -3.021368 |
| H | -3.995536 | -2.509420 | -2.535897 |
| H | -5.078678 | -1.103815 | -2.546041 |
| H | -4.450530 | -1.753311 | -4.068000 |
| C | -1.779567 | -1.284621 | -3.643434 |
| H | -0.877770 | -0.663808 | -3.584615 |
| H | -1.563122 | -2.261079 | -3.204081 |
| H | -2.005521 | -1.429215 | -4.706668 |

TS Conformation 35

B3LYP/6-31G(d) Energy = -3411.887554
M06-2X/def2tzvpp/IEFPCM(chloroform) Energy = -3411.893967
M06-2X/def2tzvpp/IEFPCM(chloroform)//B3LYP/6-31G(d) Quasiharmonic Free Energy = -3411.166815

Frequencies (Top 3 out of 291)

1. -892.2038 cm-1
2. 12.6383 cm-1
3. 15.6737 cm-1

B3LYP/6-31G(d) Molecular Geometry in Cartesian Coordinates

| | | | |
|---|---|---|---|
| C | 2.614019 | -1.482461 | -0.212610 |
| C | 2.737150 | -0.330991 | 0.808990 |
| H | 3.606640 | 0.285068 | 0.586440 |
| C | 1.550791 | 1.750379 | 0.026310 |
| N | 0.267851 | 2.210970 | -0.131270 |
| H | -0.461239 | 1.544330 | 0.108820 |
| N | 1.568480 | 0.518699 | 0.614030 |
| H | 0.676360 | 0.046290 | 0.733920 |
| C | 4.667600 | -0.696002 | -1.506930 |
| C | 3.359299 | -2.659431 | -2.210480 |
| C | 4.837830 | -0.947332 | -3.026580 |
| H | 4.326340 | 0.329358 | -1.341500 |
| C | 4.515139 | -2.438702 | -3.194270 |
| H | 3.336909 | -3.660611 | -1.769260 |
| H | 2.384309 | -2.493191 | -2.685500 |
| H | 5.837260 | -0.674872 | -3.374580 |
| H | 4.108710 | -0.336582 | -3.572420 |
| H | 5.380849 | -3.049462 | -2.917110 |
| H | 4.237219 | -2.703582 | -4.218640 |
| N | 3.568819 | -1.637872 | -1.163450 |
| O | 1.619959 | -2.232401 | -0.194390 |
| S | 2.947581 | 2.578559 | -0.448070 |
| C | 5.954930 | -0.916202 | -0.724720 |
| C | 6.395689 | -2.185273 | -0.326690 |
| C | 6.759280 | 0.194727 | -0.435080 |

| | | | |
|---|---|---|---|
| C | 7.616529 | -2.341213 | 0.331530 |
| H | 5.771759 | -3.055842 | -0.507710 |
| C | 7.979890 | 0.041087 | 0.223700 |
| C | 8.414370 | -1.229083 | 0.606230 |
| H | 7.940819 | -3.332933 | 0.636000 |
| O | -3.130130 | 0.432211 | -1.803780 |
| O | -1.244530 | -0.378970 | 0.353250 |
| C | -3.965560 | -0.183599 | -1.149800 |
| C | -3.685960 | -1.400019 | -0.330760 |
| O | -5.250900 | 0.174092 | -1.062670 |
| C | -5.634459 | 1.335322 | -1.830020 |
| H | -6.670919 | 1.526622 | -1.554240 |
| H | -5.554860 | 1.122492 | -2.899800 |
| H | -5.000979 | 2.190982 | -1.587650 |
| S | -1.897381 | -1.565810 | -0.252030 |
| C | -1.571711 | -3.012760 | 0.761970 |
| H | -1.999101 | -3.901420 | 0.295910 |
| H | -2.040701 | -2.807729 | 1.730500 |
| H | -0.482281 | -3.067490 | 0.831590 |
| C | -1.136811 | -1.971200 | -1.844760 |
| H | -1.653981 | -2.837330 | -2.264400 |
| H | -0.083121 | -2.179010 | -1.631600 |
| H | -1.271700 | -1.087840 | -2.469370 |
| C | -4.403111 | -2.664839 | -0.739210 |
| C | -4.692021 | -2.920469 | -2.092150 |
| C | -4.812071 | -3.601578 | 0.224410 |
| C | -5.369101 | -4.078098 | -2.471530 |
| H | -4.394511 | -2.200329 | -2.851020 |
| C | -5.483052 | -4.763618 | -0.159010 |
| H | -4.616941 | -3.410859 | 1.274970 |
| C | -5.762422 | -5.006168 | -1.504650 |
| H | -5.588962 | -4.254978 | -3.520820 |
| H | -5.798022 | -5.474348 | 0.599940 |
| H | -6.289642 | -5.909488 | -1.798860 |
| C | -3.432019 | 1.493781 | 2.304590 |
| C | -2.609679 | 2.595491 | 2.545290 |
| C | -1.584819 | 2.520690 | 3.491630 |
| C | -1.396159 | 1.329820 | 4.197660 |
| C | -2.209170 | 0.222811 | 3.949800 |
| C | -3.240000 | 0.283281 | 2.994920 |
| H | -4.243279 | 1.566871 | 1.585130 |
| H | -2.775579 | 3.518691 | 1.995250 |
| H | -0.948649 | 3.380820 | 3.682020 |
| H | -0.609890 | 1.260200 | 4.946230 |
| H | -2.054050 | -0.701299 | 4.499840 |
| S | -4.279300 | -1.131309 | 2.679030 |
| H | -3.988540 | -1.184919 | 0.997740 |
| H | 6.419940 | 1.187907 | -0.720050 |
| H | 8.586140 | 0.915417 | 0.444920 |
| H | 9.362650 | -1.350684 | 1.122620 |
| C | 2.874960 | -0.797601 | 2.302850 |
| C | -0.256259 | 3.411070 | -0.649770 |
| C | -1.585109 | 3.337070 | -1.106460 |
| C | 0.424312 | 4.635570 | -0.672700 |
| C | -2.199968 | 4.488381 | -1.566540 |
| H | -2.122569 | 2.395501 | -1.131130 |
| C | -0.248448 | 5.748690 | -1.162120 |
| H | 1.443382 | 4.726289 | -0.327870 |
| C | -1.562588 | 5.722400 | -1.614870 |
| H | -2.058228 | 6.611931 | -1.983180 |
| F | -3.482348 | 4.404131 | -1.995670 |
| F | 0.407053 | 6.927530 | -1.183460 |

| | | | |
|---|---|---|---|
| C | 1.623569 | -1.526371 | 2.829850 |
| H | 1.440029 | -2.454091 | 2.281950 |
| H | 1.772059 | -1.779751 | 3.886390 |
| H | 0.725110 | -0.901571 | 2.774180 |
| C | 3.118280 | 0.461829 | 3.159270 |
| H | 2.271650 | 1.153009 | 3.101420 |
| H | 3.252640 | 0.179079 | 4.210030 |
| H | 4.016680 | 0.999718 | 2.835830 |
| C | 4.090649 | -1.734352 | 2.427490 |
| H | 3.956019 | -2.656002 | 1.849030 |
| H | 5.012290 | -1.252062 | 2.086590 |
| H | 4.231269 | -2.022542 | 3.475740 |

TS Conformation 36

B3LYP/6-31G(d) Energy = -3411.890433
M06-2X/def2tzvpp/IEFPCM(chloroform) Energy = -3411.892799
M06-2X/def2tzvpp/IEFPCM(chloroform)//B3LYP/6-31G(d) Quasiharmonic Free Energy = -3411.166723

Frequencies (Top 3 out of 291)

1. -970.6350 cm-1
2. 9.7781 cm-1
3. 11.4278 cm-1

B3LYP/6-31G(d) Molecular Geometry in Cartesian Coordinates

| | | | |
|---|---|---|---|
| C | -2.793389 | -1.246405 | 0.771848 |
| C | -2.752883 | -0.650207 | -0.651093 |
| H | -3.634461 | -0.039756 | -0.839607 |
| C | -1.687825 | 1.624931 | -0.717406 |
| N | -0.478877 | 2.204509 | -0.385030 |
| H | 0.129964 | 1.632471 | 0.194981 |
| N | -1.614869 | 0.267523 | -0.674142 |
| H | -0.698667 | -0.152165 | -0.534241 |
| C | -4.933391 | 0.020561 | 1.349329 |
| C | -3.802799 | -1.439044 | 2.977069 |
| C | -5.271586 | 0.457901 | 2.797997 |
| H | -4.531700 | 0.872868 | 0.794824 |
| C | -5.040486 | -0.811164 | 3.630231 |
| H | -3.788041 | -2.532473 | 3.019369 |
| H | -2.869702 | -1.086677 | 3.433758 |
| H | -4.572794 | 1.245438 | 3.104770 |
| H | -6.286839 | 0.854719 | 2.877411 |
| H | -5.902704 | -1.481645 | 3.547705 |
| H | -4.883296 | -0.602636 | 4.692603 |
| N | -3.857813 | -0.977171 | 1.574809 |
| O | -1.847069 | -1.924880 | 1.194463 |
| S | -3.087755 | 2.488965 | -1.095314 |
| C | -6.144581 | -0.517624 | 0.598716 |
| C | -6.884846 | 0.364260 | -0.200951 |
| C | -6.578869 | -1.844567 | 0.713255 |
| C | -8.038525 | -0.064343 | -0.858346 |
| H | -6.546734 | 1.391479 | -0.316521 |
| C | -7.733332 | -2.275155 | 0.057097 |
| C | -8.468936 | -1.385966 | -0.728187 |
| H | -8.595145 | 0.632722 | -1.479116 |
| O | 1.316727 | -0.676440 | -0.655693 |
| O | 1.217960 | 1.004933 | 2.028455 |
| C | 2.532047 | -0.487604 | -0.582603 |
| C | 3.285449 | -0.404467 | 0.689988 |

| | | | |
|---|---|---|---|
| O | 3.334173 | -0.387947 | -1.642264 |
| C | 2.723880 | -0.595793 | -2.931702 |
| H | 1.858481 | 0.058615 | -3.056464 |
| H | 3.501363 | -0.353840 | -3.655611 |
| H | 2.421889 | -1.641185 | -3.029927 |
| S | 2.097515 | -0.200989 | 2.014364 |
| C | 1.135985 | -1.716735 | 2.190708 |
| H | 1.827739 | -2.557661 | 2.059960 |
| H | 0.709570 | -1.692635 | 3.196464 |
| H | 0.336718 | -1.710370 | 1.447815 |
| C | 3.133444 | -0.221319 | 3.490174 |
| H | 3.707663 | -1.151539 | 3.503816 |
| H | 3.794532 | 0.644519 | 3.446978 |
| H | 2.459295 | -0.154187 | 4.346390 |
| C | 4.420624 | 0.590207 | 0.788173 |
| C | 5.711169 | 0.171001 | 1.144405 |
| C | 4.193838 | 1.954112 | 0.537823 |
| C | 6.745726 | 1.102691 | 1.252506 |
| H | 5.901320 | -0.884074 | 1.318908 |
| C | 5.223463 | 2.884727 | 0.662873 |
| H | 3.206061 | 2.290559 | 0.240395 |
| C | 6.504367 | 2.458418 | 1.021781 |
| H | 7.743343 | 0.765323 | 1.520429 |
| H | 5.013606 | 3.936219 | 0.487848 |
| H | 7.311595 | 3.179681 | 1.117446 |
| C | 5.344302 | -4.405462 | -0.980050 |
| C | 5.233803 | -4.966394 | -2.252438 |
| C | 3.990848 | -5.053559 | -2.883214 |
| C | 2.856297 | -4.579510 | -2.221425 |
| C | 2.961834 | -4.026393 | -0.943194 |
| C | 4.209944 | -3.924657 | -0.304574 |
| H | 6.314274 | -4.334395 | -0.497028 |
| H | 6.125717 | -5.334170 | -2.754297 |
| H | 3.906539 | -5.491119 | -3.874417 |
| H | 1.878203 | -4.653954 | -2.691651 |
| H | 2.066813 | -3.680376 | -0.434373 |
| S | 4.377339 | -3.233906 | 1.336712 |
| H | 3.784772 | -1.702534 | 0.965359 |
| H | -5.999787 | -2.553141 | 1.298400 |
| H | -8.053735 | -3.309169 | 0.155050 |
| H | -9.365005 | -1.722925 | -1.242331 |
| C | -2.691642 | -1.722183 | -1.795206 |
| C | -0.000079 | 3.508125 | -0.595068 |
| C | -0.424329 | 4.329939 | -1.649157 |
| C | 1.027676 | 3.945400 | 0.260285 |
| C | 0.193607 | 5.562093 | -1.812860 |
| H | -1.214470 | 4.029900 | -2.321829 |
| C | 1.623885 | 5.174223 | 0.021176 |
| H | 1.349252 | 3.342378 | 1.101610 |
| C | 1.229991 | 6.021093 | -1.005897 |
| H | 1.700725 | 6.981986 | -1.171600 |
| F | -0.215654 | 6.348901 | -2.827951 |
| F | 2.641495 | 5.559862 | 0.826073 |
| C | -1.397616 | -2.559437 | -1.779014 |
| H | -1.293744 | -3.120683 | -0.847643 |
| H | -1.421388 | -3.272174 | -2.612767 |
| H | -0.504120 | -1.939161 | -1.902662 |
| C | -2.794478 | -0.976001 | -3.140762 |
| H | -3.724581 | -0.401432 | -3.212493 |
| H | -1.961184 | -0.278768 | -3.275928 |
| H | -2.772639 | -1.693858 | -3.969388 |
| C | -3.900916 | -2.665780 | -1.650671 |

| | | | |
|---|---|---|---|
| H | -4.849161 | -2.118349 | -1.665237 |
| H | -3.914136 | -3.381335 | -2.481062 |
| H | -3.854981 | -3.243319 | -0.720244 |

TS Conformation 37

B3LYP/6-31G(d) Energy = -3411.888079
M06-2X/def2tzvpp/IEFPCM(chloroform) Energy = -3411.892034
M06-2X/def2tzvpp/IEFPCM(chloroform)//B3LYP/6-31G(d) Quasiharmonic Free Energy = -3411.16639

Frequencies (Top 3 out of 291)

1. -963.6463 cm-1
2. 5.3916 cm-1
3. 11.3004 cm-1

B3LYP/6-31G(d) Molecular Geometry in Cartesian Coordinates

| | | | |
|---|---|---|---|
| C | 2.435591 | -0.855733 | 1.015411 |
| C | 2.842188 | 0.595883 | 1.338979 |
| H | 3.521372 | 0.989376 | 0.584101 |
| C | 1.370126 | 2.396378 | 0.358993 |
| N | 0.047011 | 2.780169 | 0.372190 |
| H | -0.600541 | 2.129486 | 0.811274 |
| N | 1.610727 | 1.381306 | 1.233275 |
| H | 0.819191 | 1.000940 | 1.743984 |
| C | 3.452269 | -0.711750 | -1.310770 |
| C | 2.269028 | -2.727297 | -0.533360 |
| C | 2.933684 | -1.542027 | -2.513618 |
| H | 3.078685 | 0.313335 | -1.382714 |
| C | 2.802561 | -2.964074 | -1.950456 |
| H | 2.587204 | -3.481746 | 0.191854 |
| H | 1.174912 | -2.685475 | -0.512192 |
| H | 3.599742 | -1.468881 | -3.377239 |
| H | 1.947417 | -1.161541 | -2.805236 |
| H | 3.782937 | -3.452650 | -1.921409 |
| H | 2.129655 | -3.598048 | -2.534931 |
| N | 2.809417 | -1.400166 | -0.166556 |
| O | 1.732433 | -1.491963 | 1.815100 |
| S | 2.549231 | 3.086367 | -0.631240 |
| C | 4.973553 | -0.665782 | -1.234575 |
| C | 5.648418 | 0.375234 | -1.887745 |
| C | 5.727476 | -1.645664 | -0.576483 |
| C | 7.042257 | 0.427327 | -1.897178 |
| H | 5.074669 | 1.156447 | -2.380776 |
| C | 7.123132 | -1.595033 | -0.584891 |
| C | 7.785108 | -0.560780 | -1.247935 |
| H | 7.547185 | 1.245974 | -2.403138 |
| O | -0.816014 | -0.852225 | -0.493198 |
| O | -1.664103 | 0.766940 | 2.100191 |
| C | -2.016748 | -0.871228 | -0.733921 |
| C | -3.082271 | -0.922071 | 0.304082 |
| O | -2.531365 | -0.888029 | -1.972447 |
| C | -1.571209 | -0.913356 | -3.044957 |
| H | -0.884912 | -0.067156 | -2.964453 |
| H | -1.006365 | -1.848877 | -3.018868 |
| H | -2.161312 | -0.845832 | -3.958803 |
| S | -2.331487 | -0.555183 | 1.879320 |
| C | -1.240378 | -1.913021 | 2.332740 |
| H | -1.777029 | -2.842131 | 2.116978 |
| H | -0.310092 | -1.816473 | 1.768714 |

| | | | |
|---|---|---|---|
| H | -1.045360 | -1.794935 | 3.401674 |
| C | -3.701269 | -0.759142 | 3.035484 |
| H | -4.458791 | -0.011062 | 2.802022 |
| H | -4.093431 | -1.773388 | 2.913004 |
| H | -3.291992 | -0.602977 | 4.035591 |
| C | -4.372779 | -0.176865 | 0.040103 |
| C | -4.504188 | 1.203481 | 0.270545 |
| C | -5.458485 | -0.874685 | -0.510264 |
| C | -5.685230 | 1.872737 | -0.050387 |
| H | -3.678871 | 1.762123 | 0.700843 |
| C | -6.637271 | -0.201437 | -0.834209 |
| H | -5.373211 | -1.941983 | -0.685863 |
| C | -6.755127 | 1.170689 | -0.608023 |
| H | -5.752585 | 2.944770 | 0.111221 |
| H | -7.466464 | -0.755615 | -1.265535 |
| H | -7.672626 | 1.691252 | -0.868248 |
| C | -2.357208 | -4.391583 | -1.484669 |
| C | -1.294528 | -4.977402 | -2.174546 |
| C | -0.386640 | -5.804596 | -1.508140 |
| C | -0.556526 | -6.041659 | -0.142428 |
| C | -1.614769 | -5.453001 | 0.551489 |
| C | -2.530349 | -4.613575 | -0.106433 |
| H | -3.069744 | -3.765313 | -2.013276 |
| H | -1.187392 | -4.801950 | -3.242926 |
| H | 0.432876 | -6.270254 | -2.049152 |
| H | 0.136405 | -6.690530 | 0.387933 |
| H | -1.743574 | -5.647596 | 1.612619 |
| S | -3.897105 | -3.875793 | 0.781437 |
| H | -3.442499 | -2.279911 | 0.486341 |
| H | 5.221918 | -2.442174 | -0.038247 |
| H | 7.692203 | -2.363265 | -0.067676 |
| H | 8.871013 | -0.519495 | -1.250620 |
| C | 3.547369 | 0.766100 | 2.730828 |
| C | -0.566438 | 3.911651 | -0.201187 |
| C | 0.050390 | 5.168799 | -0.259672 |
| C | -1.889758 | 3.762901 | -0.644097 |
| C | -0.677715 | 6.236105 | -0.766910 |
| H | 1.067477 | 5.318119 | 0.072785 |
| C | -2.573719 | 4.874092 | -1.115740 |
| H | -2.378319 | 2.796694 | -0.645923 |
| C | -1.996809 | 6.133840 | -1.198569 |
| H | -2.540261 | 6.989730 | -1.578988 |
| F | -0.084515 | 7.445020 | -0.820902 |
| F | -3.854228 | 4.716483 | -1.517167 |
| C | 4.699418 | -0.252547 | 2.832012 |
| H | 5.394107 | -0.162686 | 1.989129 |
| H | 5.269123 | -0.080810 | 3.752822 |
| H | 4.324260 | -1.281360 | 2.860542 |
| C | 2.583116 | 0.566849 | 3.917012 |
| H | 3.130004 | 0.698167 | 4.858497 |
| H | 1.777055 | 1.310435 | 3.905784 |
| H | 2.138562 | -0.430435 | 3.906985 |
| C | 4.128080 | 2.192959 | 2.794242 |
| H | 3.345837 | 2.949866 | 2.676711 |
| H | 4.614415 | 2.356601 | 3.763567 |
| H | 4.871807 | 2.360197 | 2.007517 |

TS Conformation 38

B3LYP/6-31G(d) Energy = -3411.889286
M06-2X/def2tzvpp/IEFPCM(chloroform) Energy = -3411.891289

M06-2X/def2tzvpp/IEFPCM(chloroform)//B3LYP/6-31G(d) Quasiharmonic Free Energy = -3411.166186

Frequencies (Top 3 out of 291)

1. -973.9569 cm-1
2. 5.8201 cm-1
3. 10.9521 cm-1

B3LYP/6-31G(d) Molecular Geometry in Cartesian Coordinates

| | | | |
|---|---|---|---|
| C | -2.849797 | -1.029445 | 0.792958 |
| C | -2.827742 | -0.626589 | -0.697197 |
| H | -3.692179 | -0.014925 | -0.949158 |
| C | -1.651497 | 1.575577 | -1.040508 |
| N | -0.370138 | 2.076019 | -0.991530 |
| H | 0.339550 | 1.366739 | -0.819065 |
| N | -1.651775 | 0.222031 | -0.870601 |
| H | -0.775995 | -0.217629 | -0.602283 |
| C | -4.983782 | 0.296156 | 1.242664 |
| C | -3.817460 | -0.942120 | 3.021803 |
| C | -5.285097 | 0.923516 | 2.628092 |
| H | -4.594192 | 1.065456 | 0.570793 |
| C | -5.036888 | -0.225478 | 3.615438 |
| H | -3.807781 | -2.021067 | 3.205136 |
| H | -2.872313 | -0.540728 | 3.406881 |
| H | -6.297080 | 1.333277 | 2.679053 |
| H | -4.576315 | 1.740230 | 2.809863 |
| H | -5.902966 | -0.895530 | 3.644124 |
| H | -4.852054 | 0.121433 | 4.636345 |
| N | -3.902022 | -0.665163 | 1.572296 |
| O | -1.892519 | -1.648006 | 1.279477 |
| S | -3.052700 | 2.478258 | -1.312759 |
| C | -6.213339 | -0.333832 | 0.601683 |
| C | -6.641313 | -1.634374 | 0.898292 |
| C | -6.978091 | 0.438138 | -0.284065 |
| C | -7.812151 | -2.144995 | 0.335002 |
| H | -6.044569 | -2.261616 | 1.554224 |
| C | -8.148277 | -0.070354 | -0.848778 |
| C | -8.571366 | -1.363898 | -0.537862 |
| H | -8.127216 | -3.157329 | 0.574508 |
| O | 1.322344 | -0.376786 | -0.408140 |
| O | 1.639117 | 1.063398 | 2.566298 |
| C | 2.556440 | -0.344930 | -0.343819 |
| C | 3.345689 | -0.464282 | 0.897907 |
| O | 3.342594 | -0.245080 | -1.418289 |
| C | 2.700507 | -0.328820 | -2.706599 |
| H | 2.248042 | -1.315476 | -2.829071 |
| H | 1.942014 | 0.450079 | -2.814961 |
| H | 3.501905 | -0.183773 | -3.430059 |
| S | 2.284212 | -0.249405 | 2.323683 |
| C | 1.098453 | -1.607691 | 2.365187 |
| H | 0.296576 | -1.401857 | 1.655214 |
| H | 1.642817 | -2.526574 | 2.118616 |
| H | 0.701885 | -1.632908 | 3.383167 |
| C | 3.395797 | -0.667071 | 3.683733 |
| H | 2.802589 | -0.614288 | 4.598945 |
| H | 3.797588 | -1.670868 | 3.522877 |
| H | 4.194206 | 0.075490 | 3.697769 |
| C | 4.610079 | 0.363966 | 0.996882 |
| C | 4.547807 | 1.767850 | 0.954007 |
| C | 5.862704 | -0.258963 | 1.096859 |
| C | 5.712264 | 2.532336 | 1.000195 |

| | | | |
|---|---|---|---|
| H | 3.585107 | 2.264823 | 0.886787 |
| C | 7.026662 | 0.510426 | 1.145842 |
| H | 5.919452 | -1.343270 | 1.117855 |
| C | 6.955570 | 1.903699 | 1.096828 |
| H | 5.640776 | 3.614692 | 0.945054 |
| H | 7.992007 | 0.016359 | 1.215975 |
| H | 7.865217 | 2.497521 | 1.128169 |
| C | 2.482544 | -3.935194 | -1.041276 |
| C | 2.263088 | -4.302948 | -2.371139 |
| C | 3.334178 | -4.666712 | -3.190111 |
| C | 4.628299 | -4.658536 | -2.664658 |
| C | 4.851379 | -4.284466 | -1.339454 |
| C | 3.782100 | -3.914142 | -0.506257 |
| H | 1.638330 | -3.675110 | -0.409365 |
| H | 1.247403 | -4.319002 | -2.759932 |
| H | 3.161773 | -4.960494 | -4.222158 |
| H | 5.471237 | -4.943831 | -3.289695 |
| H | 5.859686 | -4.277322 | -0.936438 |
| S | 4.088819 | -3.461036 | 1.197233 |
| H | 3.688487 | -1.837929 | 1.021475 |
| H | -6.647596 | 1.442717 | -0.538347 |
| H | -8.724083 | 0.541723 | -1.537934 |
| H | -9.480431 | -1.763379 | -0.979247 |
| C | -2.814007 | -1.845755 | -1.686364 |
| C | 0.168059 | 3.372301 | -1.035330 |
| C | -0.569065 | 4.562990 | -1.101814 |
| C | 1.575644 | 3.435377 | -0.981723 |
| C | 0.124248 | 5.767586 | -1.098109 |
| H | -1.647210 | 4.557152 | -1.151871 |
| C | 2.202708 | 4.669159 | -0.965724 |
| H | 2.182500 | 2.537792 | -0.953621 |
| C | 1.508255 | 5.870736 | -1.025709 |
| H | 2.012800 | 6.828630 | -1.016154 |
| F | -0.593074 | 6.907033 | -1.159403 |
| F | 3.552357 | 4.699890 | -0.883313 |
| C | -2.981809 | -1.288075 | -3.113949 |
| H | -2.174410 | -0.593808 | -3.368806 |
| H | -2.966810 | -2.107210 | -3.842730 |
| H | -3.931664 | -0.752982 | -3.226106 |
| C | -4.002223 | -2.770697 | -1.363556 |
| H | -4.049625 | -3.584317 | -2.096773 |
| H | -3.902250 | -3.226930 | -0.372091 |
| H | -4.956098 | -2.234240 | -1.397031 |
| C | -1.507574 | -2.661229 | -1.611003 |
| H | -1.350261 | -3.075624 | -0.612480 |
| H | -1.556338 | -3.489652 | -2.328294 |
| H | -0.632070 | -2.055550 | -1.871385 |

TS Conformation 39

B3LYP/6-31G(d) Energy = -3411.887963
M06-2X/def2tzvpp/IEFPCM(chloroform) Energy = -3411.89198
M06-2X/def2tzvpp/IEFPCM(chloroform)//B3LYP/6-31G(d) Quasiharmonic Free Energy = -3411.166157

Frequencies (Top 3 out of 291)

1. -971.5744 cm-1
2. 6.9131 cm-1
3. 11.7693 cm-1

B3LYP/6-31G(d) Molecular Geometry in Cartesian Coordinates

| | | | |
|---|---|---|---|
| C | -3.781602 | -0.476901 | -1.039049 |
| C | -3.550554 | 0.908379 | -1.675422 |
| H | -4.235193 | 1.645635 | -1.256575 |
| C | -1.950020 | 2.434196 | -0.482587 |
| N | -0.626370 | 2.479812 | -0.123352 |
| H | -0.064205 | 1.705998 | -0.465214 |
| N | -2.219655 | 1.323446 | -1.242946 |
| H | -1.529962 | 0.575529 | -1.222831 |
| C | -4.979875 | -1.897084 | 0.576990 |
| C | -5.894196 | 0.333888 | 0.114890 |
| C | -6.304681 | -1.675502 | 1.353785 |
| H | -5.103532 | -2.689540 | -0.171621 |
| C | -7.036049 | -0.583477 | 0.565072 |
| H | -5.615819 | 1.050830 | 0.897281 |
| H | -6.143627 | 0.896343 | -0.784836 |
| H | -6.075880 | -1.320846 | 2.364924 |
| H | -6.873341 | -2.604672 | 1.450673 |
| H | -7.785219 | -0.052184 | 1.159248 |
| H | -7.537968 | -1.009702 | -0.311794 |
| N | -4.788249 | -0.614025 | -0.138399 |
| O | -3.011995 | -1.415238 | -1.294995 |
| S | -3.116706 | 3.588801 | -0.091944 |
| C | -3.838471 | -2.288341 | 1.503459 |
| C | -3.648550 | -3.641567 | 1.810935 |
| C | -3.024180 | -1.335348 | 2.126986 |
| C | -2.674400 | -4.036237 | 2.730041 |
| H | -4.270288 | -4.393404 | 1.328700 |
| C | -2.039741 | -1.727204 | 3.035692 |
| C | -1.864922 | -3.078518 | 3.345820 |
| H | -2.546668 | -5.090729 | 2.961277 |
| O | 2.298662 | -3.997018 | -1.452153 |
| O | -0.019397 | -0.484416 | -0.332123 |
| C | 2.811994 | -3.228369 | -0.641459 |
| C | 2.214207 | -1.963803 | -0.159773 |
| O | 4.040887 | -3.411311 | -0.133825 |
| C | 4.758767 | -4.556297 | -0.624643 |
| H | 4.210885 | -5.477455 | -0.409336 |
| H | 4.914309 | -4.475058 | -1.703303 |
| H | 5.712905 | -4.541946 | -0.098487 |
| S | 0.526701 | -1.799812 | -0.762023 |
| C | 0.533288 | -1.905407 | -2.561854 |
| H | -0.486100 | -1.664223 | -2.871983 |
| H | 1.256075 | -1.159760 | -2.909075 |
| H | 0.840883 | -2.908776 | -2.851011 |
| C | -0.552172 | -3.135028 | -0.213523 |
| H | -0.143595 | -4.068512 | -0.604197 |
| H | -0.542964 | -3.113393 | 0.878363 |
| H | -1.553052 | -2.899906 | -0.587333 |
| C | 2.295076 | -1.625618 | 1.305570 |
| C | 2.249086 | -2.633115 | 2.285958 |
| C | 2.391604 | -0.288337 | 1.717476 |
| C | 2.309128 | -2.311675 | 3.640227 |
| H | 2.185455 | -3.676062 | 1.986218 |
| C | 2.439341 | 0.032642 | 3.074380 |
| H | 2.445185 | 0.494578 | 0.969839 |
| C | 2.398478 | -0.975011 | 4.038480 |
| H | 2.287465 | -3.103810 | 4.384160 |
| H | 2.515641 | 1.074154 | 3.373620 |
| H | 2.443525 | -0.723198 | 5.094734 |
| C | 5.621360 | 1.401243 | -0.815641 |
| C | 7.004675 | 1.379974 | -0.632858 |

| | | | |
|---|---|---|---|
| C | 7.779486 | 0.363335 | -1.194792 |
| C | 7.156238 | -0.632328 | -1.950023 |
| C | 5.774551 | -0.611551 | -2.141674 |
| C | 4.986410 | 0.402103 | -1.572177 |
| H | 5.025142 | 2.192945 | -0.371529 |
| H | 7.477823 | 2.163149 | -0.045024 |
| H | 8.856662 | 0.349421 | -1.049981 |
| H | 7.749175 | -1.425355 | -2.400700 |
| H | 5.296516 | -1.377509 | -2.746308 |
| S | 3.216018 | 0.446480 | -1.818665 |
| H | 2.761638 | -0.900067 | -0.904076 |
| H | -3.148160 | -0.282528 | 1.890000 |
| H | -1.403942 | -0.976738 | 3.496937 |
| H | -1.099758 | -3.379847 | 4.055876 |
| C | -3.732087 | 0.931446 | -3.233736 |
| C | 0.146133 | 3.431283 | 0.574488 |
| C | 1.536553 | 3.347794 | 0.366585 |
| C | -0.373266 | 4.380724 | 1.464765 |
| C | 2.367504 | 4.216191 | 1.055598 |
| H | 1.970324 | 2.636308 | -0.332470 |
| C | 0.517840 | 5.226432 | 2.113844 |
| H | -1.433933 | 4.470846 | 1.642857 |
| C | 1.895480 | 5.177226 | 1.942504 |
| H | 2.562331 | 5.851882 | 2.464814 |
| F | 3.701173 | 4.132472 | 0.852554 |
| F | 0.015084 | 6.138502 | 2.971691 |
| C | -2.676358 | 0.083417 | -3.967417 |
| H | -2.748655 | -0.973237 | -3.696491 |
| H | -2.818208 | 0.172647 | -5.051033 |
| H | -1.663670 | 0.437340 | -3.742175 |
| C | -5.137712 | 0.399155 | -3.577364 |
| H | -5.924144 | 1.002739 | -3.107111 |
| H | -5.302203 | 0.441116 | -4.660098 |
| H | -5.269428 | -0.644264 | -3.266299 |
| C | -3.615829 | 2.393053 | -3.708584 |
| H | -4.371561 | 3.031467 | -3.238820 |
| H | -2.634031 | 2.812237 | -3.468364 |
| H | -3.749924 | 2.445948 | -4.795716 |

TS Conformation 40 (**TS-2’**)

B3LYP/6-31G(d) Energy = -3411.887092
M06-2X/def2tzvpp/IEFPCM(chloroform) Energy = -3411.89306
M06-2X/def2tzvpp/IEFPCM(chloroform)//B3LYP/6-31G(d) Quasiharmonic Free Energy = -3411.166076

Frequencies (Top 3 out of 291)

1. -719.6427 cm-1
2. 9.1771 cm-1
3. 13.6612 cm-1

B3LYP/6-31G(d) Molecular Geometry in Cartesian Coordinates

| | | | |
|---|---|---|---|
| C | -3.489275 | -1.204973 | -0.206800 |
| C | -3.651419 | -0.224163 | -1.384561 |
| H | -4.448856 | 0.488232 | -1.178700 |
| C | -2.292138 | 1.836575 | -0.970161 |
| N | -0.983789 | 2.246214 | -0.958319 |
| H | -0.290379 | 1.512412 | -1.088771 |
| N | -2.427024 | 0.565245 | -1.454166 |
| H | -1.574339 | 0.040439 | -1.630936 |

| | | | |
|---|---|---|---|
| C | -4.191685 | -2.022501 | 1.991258 |
| C | -5.580533 | -0.292482 | 0.926127 |
| C | -5.571399 | -1.936486 | 2.687404 |
| H | -3.988693 | -3.036562 | 1.633771 |
| C | -6.042481 | -0.509363 | 2.373702 |
| H | -5.345963 | 0.752779 | 0.708158 |
| H | -6.342331 | -0.638572 | 0.215626 |
| H | -5.506256 | -2.147959 | 3.757787 |
| H | -6.252553 | -2.668513 | 2.236333 |
| H | -5.547491 | 0.208011 | 3.037232 |
| H | -7.122597 | -0.377709 | 2.483844 |
| N | -4.369369 | -1.138152 | 0.822932 |
| O | -2.540152 | -2.002953 | -0.186198 |
| S | -3.599118 | 2.773338 | -0.442200 |
| C | -3.037602 | -1.603477 | 2.899083 |
| C | -2.531229 | -0.298639 | 2.925354 |
| C | -2.486097 | -2.555155 | 3.768882 |
| C | -1.499309 | 0.047160 | 3.802074 |
| H | -2.926734 | 0.451632 | 2.246625 |
| C | -1.460287 | -2.212305 | 4.650366 |
| C | -0.961799 | -0.905949 | 4.669652 |
| H | -1.115603 | 1.063512 | 3.800977 |
| O | 0.444372 | -0.316984 | -1.867647 |
| O | 1.336941 | 0.326472 | 0.852035 |
| C | 1.455765 | -0.998016 | -2.025623 |
| C | 2.349371 | -1.524737 | -0.958138 |
| O | 1.954359 | -1.304793 | -3.226153 |
| C | 1.285363 | -0.739171 | -4.369068 |
| H | 1.907102 | -1.007115 | -5.222548 |
| H | 0.284540 | -1.165933 | -4.475010 |
| H | 1.214200 | 0.345908 | -4.269137 |
| S | 1.672980 | -1.099564 | 0.662169 |
| C | 0.271170 | -2.151662 | 1.092164 |
| H | 0.570040 | -3.195398 | 0.978736 |
| H | 0.008814 | -1.920652 | 2.127871 |
| H | -0.564740 | -1.906686 | 0.429814 |
| C | 2.948054 | -1.604211 | 1.832815 |
| H | 2.544990 | -1.378933 | 2.822800 |
| H | 3.161995 | -2.667473 | 1.718920 |
| H | 3.834827 | -0.995779 | 1.631426 |
| C | 2.750249 | -2.981539 | -1.067331 |
| C | 1.800126 | -3.965516 | -1.395885 |
| C | 4.082093 | -3.369629 | -0.856426 |
| C | 2.169540 | -5.305550 | -1.496531 |
| H | 0.768152 | -3.679098 | -1.586376 |
| C | 4.447438 | -4.713669 | -0.953923 |
| H | 4.829859 | -2.605261 | -0.662148 |
| C | 3.495161 | -5.683673 | -1.268617 |
| H | 1.424122 | -6.052308 | -1.755908 |
| H | 5.483977 | -4.998612 | -0.795872 |
| H | 3.784298 | -6.728209 | -1.347189 |
| C | 6.141134 | 0.141092 | 1.192849 |
| C | 6.336302 | 0.673728 | 2.469752 |
| C | 5.640643 | 1.815662 | 2.873754 |
| C | 4.756258 | 2.425800 | 1.980456 |
| C | 4.563029 | 1.897182 | 0.704047 |
| C | 5.242286 | 0.736920 | 0.283586 |
| H | 6.698001 | -0.737757 | 0.878195 |
| H | 7.041275 | 0.196688 | 3.147660 |
| H | 5.794141 | 2.230608 | 3.866686 |
| H | 4.211917 | 3.321259 | 2.267433 |
| H | 3.891429 | 2.394916 | 0.012821 |

| | | | |
|---|---|---|---|
| S | 4.987227 | 0.049835 | -1.342485 |
| H | 3.460192 | -0.788623 | -1.053123 |
| H | -2.863480 | -3.575967 | 3.754131 |
| H | -1.050286 | -2.963888 | 5.320453 |
| H | -0.165816 | -0.633135 | 5.357716 |
| C | -3.989854 | -0.924223 | -2.744370 |
| C | -0.427355 | 3.508586 | -0.656679 |
| C | -1.051540 | 4.719106 | -0.986157 |
| C | 0.849598 | 3.509263 | -0.072628 |
| C | -0.382725 | 5.901698 | -0.701239 |
| H | -2.029857 | 4.749579 | -1.442549 |
| C | 1.467833 | 4.725961 | 0.171588 |
| H | 1.341325 | 2.584215 | 0.205568 |
| C | 0.881543 | 5.949602 | -0.124990 |
| H | 1.381556 | 6.888201 | 0.079161 |
| F | -0.983512 | 7.067149 | -1.018240 |
| F | 2.699541 | 4.723626 | 0.730925 |
| C | -2.840095 | -1.811147 | -3.260987 |
| H | -2.590598 | -2.602146 | -2.549772 |
| H | -3.130504 | -2.272687 | -4.212557 |
| H | -1.934773 | -1.221717 | -3.445904 |
| C | -4.288643 | 0.175636 | -3.782066 |
| H | -4.534529 | -0.274638 | -4.751180 |
| H | -5.136010 | 0.798628 | -3.473419 |
| H | -3.425218 | 0.833525 | -3.922437 |
| C | -5.249478 | -1.791616 | -2.550227 |
| H | -6.096300 | -1.195343 | -2.188361 |
| H | -5.548890 | -2.240160 | -3.504332 |
| H | -5.076606 | -2.610254 | -1.841913 |

TS Conformation 41

B3LYP/6-31G(d) Energy = -3411.891098
M06-2X/def2tzvpp/IEFPCM(chloroform) Energy = -3411.892608
M06-2X/def2tzvpp/IEFPCM(chloroform)//B3LYP/6-31G(d) Quasiharmonic Free Energy = -3411.166043

Frequencies (Top 3 out of 291)

1. -988.6686 cm-1
2. 8.2840 cm-1
3. 10.1758 cm-1

B3LYP/6-31G(d) Molecular Geometry in Cartesian Coordinates

| | | | |
|---|---|---|---|
| C | 2.537209 | -0.521718 | 1.187566 |
| C | 2.773298 | 0.988161 | 1.390742 |
| H | 3.389869 | 1.399235 | 0.593043 |
| C | 1.080504 | 2.534829 | 0.328375 |
| N | -0.269811 | 2.797431 | 0.399189 |
| H | -0.780466 | 2.211475 | 1.055985 |
| N | 1.454286 | 1.611314 | 1.259372 |
| H | 0.721682 | 1.147644 | 1.788516 |
| C | 3.531925 | -0.455360 | -1.150161 |
| C | 2.600028 | -2.524831 | -0.194635 |
| C | 3.106783 | -1.436063 | -2.273754 |
| H | 3.039722 | 0.509566 | -1.298299 |
| C | 3.146877 | -2.813238 | -1.596963 |
| H | 3.011948 | -3.173957 | 0.583644 |
| H | 1.509017 | -2.610258 | -0.159467 |
| H | 3.753681 | -1.356340 | -3.151302 |
| H | 2.080383 | -1.198861 | -2.577400 |

| | | | |
|---|---|---|---|
| H | 4.177592 | -3.181851 | -1.546022 |
| H | 2.545177 | -3.564368 | -2.116371 |
| N | 2.980981 | -1.117319 | 0.055972 |
| O | 1.906164 | -1.164039 | 2.041116 |
| S | 2.170734 | 3.258279 | -0.738170 |
| C | 5.037635 | -0.226350 | -1.102434 |
| C | 5.905541 | -1.053711 | -0.378730 |
| C | 5.581889 | 0.827444 | -1.850124 |
| C | 7.285574 | -0.842270 | -0.414099 |
| H | 5.500111 | -1.855797 | 0.231123 |
| C | 6.959918 | 1.040221 | -1.886675 |
| C | 7.817601 | 0.202844 | -1.170527 |
| H | 7.944059 | -1.493424 | 0.155031 |
| O | -0.580879 | -0.916663 | -0.359387 |
| O | -1.753602 | 0.709516 | 2.154828 |
| C | -1.766228 | -1.017338 | -0.652396 |
| C | -2.864156 | -1.188261 | 0.334196 |
| O | -2.228314 | -1.033618 | -1.911787 |
| C | -1.228600 | -0.963822 | -2.944797 |
| H | -0.589275 | -0.089819 | -2.800540 |
| H | -0.621675 | -1.873010 | -2.937504 |
| H | -1.786398 | -0.883638 | -3.877898 |
| S | -2.244537 | -0.689297 | 1.934131 |
| C | -3.644611 | -1.001851 | 3.026877 |
| H | -3.931682 | -2.052365 | 2.931305 |
| H | -3.306367 | -0.769995 | 4.038741 |
| H | -4.460194 | -0.343205 | 2.727928 |
| C | -1.030463 | -1.890077 | 2.502297 |
| H | -1.434053 | -2.883817 | 2.277138 |
| H | -0.083278 | -1.694984 | 1.993778 |
| H | -0.923095 | -1.725679 | 3.577512 |
| C | -4.220683 | -0.594186 | 0.028713 |
| C | -5.358666 | -1.413023 | -0.033623 |
| C | -4.367569 | 0.785862 | -0.187222 |
| C | -6.611828 | -0.856393 | -0.297477 |
| H | -5.257354 | -2.484989 | 0.105281 |
| C | -5.621613 | 1.343658 | -0.431115 |
| H | -3.492792 | 1.427206 | -0.169847 |
| C | -6.748457 | 0.519970 | -0.486181 |
| H | -7.483537 | -1.502965 | -0.351601 |
| H | -5.710263 | 2.417547 | -0.567366 |
| H | -7.727838 | 0.949293 | -0.679823 |
| C | -1.999063 | -4.506424 | -1.681305 |
| C | -0.995329 | -4.943730 | -2.547428 |
| C | 0.098519 | -5.663456 | -2.059358 |
| C | 0.177810 | -5.939870 | -0.692403 |
| C | -0.819847 | -5.497299 | 0.177632 |
| C | -1.923315 | -4.771994 | -0.302572 |
| H | -2.855105 | -3.963414 | -2.070376 |
| H | -1.079545 | -4.737592 | -3.612219 |
| H | 0.870271 | -6.017985 | -2.737670 |
| H | 1.018590 | -6.506493 | -0.299175 |
| H | -0.753368 | -5.718935 | 1.238922 |
| S | -3.217007 | -4.238239 | 0.811899 |
| H | -3.033153 | -2.604751 | 0.528474 |
| H | 4.917576 | 1.492145 | -2.397453 |
| H | 7.362223 | 1.866573 | -2.466708 |
| H | 8.891158 | 0.369529 | -1.194365 |
| C | 3.474056 | 1.344732 | 2.750056 |
| C | -1.104225 | 3.724534 | -0.247999 |
| C | -0.743567 | 4.516559 | -1.347756 |
| C | -2.414769 | 3.812893 | 0.266368 |

| | | | |
|---|---|---|---|
| C | -1.701583 | 5.360831 | -1.895550 |
| H | 0.250455 | 4.479752 | -1.766380 |
| C | -3.325588 | 4.659573 | -0.343066 |
| H | -2.720404 | 3.232995 | 1.130615 |
| C | -3.007995 | 5.460068 | -1.432393 |
| H | -3.732447 | 6.120045 | -1.892688 |
| F | -1.344951 | 6.116877 | -2.952392 |
| F | -4.589435 | 4.696550 | 0.141432 |
| C | 3.885954 | 2.829374 | 2.693561 |
| H | 3.020296 | 3.480545 | 2.534468 |
| H | 4.360480 | 3.122493 | 3.637895 |
| H | 4.597264 | 3.019088 | 1.882281 |
| C | 4.737699 | 0.475829 | 2.901413 |
| H | 4.485061 | -0.583886 | 3.016798 |
| H | 5.405416 | 0.578450 | 2.038553 |
| H | 5.296361 | 0.782996 | 3.793305 |
| C | 2.555556 | 1.127386 | 3.968754 |
| H | 2.227463 | 0.088448 | 4.041965 |
| H | 3.096783 | 1.390152 | 4.885655 |
| H | 1.669627 | 1.771903 | 3.920668 |

TS Conformation 42

B3LYP/6-31G(d) Energy = -3411.893537
M06-2X/def2tzvpp/IEFPCM(chloroform) Energy = -3411.892414
M06-2X/def2tzvpp/IEFPCM(chloroform)//B3LYP/6-31G(d) Quasiharmonic Free Energy = -3411.16597

Frequencies (Top 3 out of 291)

1. -993.4960 cm-1
2. 6.2720 cm-1
3. 9.9635 cm-1

B3LYP/6-31G(d) Molecular Geometry in Cartesian Coordinates

| | | | |
|---|---|---|---|
| C | -2.775105 | -1.618802 | -0.030923 |
| C | -3.063153 | -0.651368 | -1.197595 |
| H | -3.992653 | -0.107351 | -1.035411 |
| C | -2.103125 | 1.679764 | -0.948954 |
| N | -0.868131 | 2.253736 | -0.764333 |
| H | -0.107409 | 1.584626 | -0.688681 |
| N | -1.981404 | 0.331144 | -1.151505 |
| H | -1.075246 | -0.077781 | -0.940805 |
| C | -4.714099 | -0.675726 | 1.323063 |
| C | -3.260223 | -2.457058 | 2.206316 |
| C | -4.756011 | -0.697225 | 2.873090 |
| H | -4.429964 | 0.321818 | 0.976961 |
| C | -4.349857 | -2.132694 | 3.235293 |
| H | -3.213262 | -3.514477 | 1.929162 |
| H | -2.262975 | -2.168332 | 2.561627 |
| H | -5.738054 | -0.410210 | 3.256946 |
| H | -4.017282 | 0.014302 | 3.261249 |
| H | -5.202758 | -2.810100 | 3.119817 |
| H | -3.986677 | -2.227845 | 4.262673 |
| N | -3.615192 | -1.632841 | 1.033847 |
| O | -1.757136 | -2.330385 | -0.048688 |
| S | -3.583833 | 2.487397 | -0.953256 |
| C | -6.049424 | -1.043716 | 0.689465 |
| C | -6.450424 | -2.370584 | 0.487034 |
| C | -6.933875 | -0.013344 | 0.341381 |
| C | -7.712093 | -2.660679 | -0.035740 |

| | | | |
|---|---|---|---|
| H | -5.766456 | -3.182502 | 0.716921 |
| C | -8.194785 | -0.301514 | -0.181595 |
| C | -8.589632 | -1.627449 | -0.368361 |
| H | -8.005994 | -3.695858 | -0.188483 |
| O | 3.238482 | 0.870040 | -0.999345 |
| O | 0.570284 | -0.047862 | 0.335198 |
| C | 3.737381 | 0.564601 | 0.079774 |
| C | 3.274665 | -0.558903 | 0.937906 |
| O | 4.792809 | 1.195377 | 0.612341 |
| C | 5.318049 | 2.292862 | -0.164273 |
| H | 4.573769 | 3.088156 | -0.251980 |
| H | 6.187889 | 2.641584 | 0.392117 |
| H | 5.606123 | 1.947582 | -1.159778 |
| S | 1.670363 | -1.060384 | 0.340698 |
| C | 1.865864 | -1.796131 | -1.294380 |
| H | 2.651826 | -2.554435 | -1.223233 |
| H | 2.151960 | -0.986203 | -1.963940 |
| H | 0.893388 | -2.224464 | -1.548866 |
| C | 1.202119 | -2.460387 | 1.367287 |
| H | 0.201285 | -2.748175 | 1.029903 |
| H | 1.186831 | -2.114856 | 2.401816 |
| H | 1.947626 | -3.248212 | 1.225722 |
| C | 3.291177 | -0.346806 | 2.437021 |
| C | 4.123215 | -1.131082 | 3.249634 |
| C | 2.500156 | 0.650860 | 3.034630 |
| C | 4.161054 | -0.921377 | 4.629325 |
| H | 4.752995 | -1.890442 | 2.796913 |
| C | 2.533708 | 0.850085 | 4.414102 |
| H | 1.859086 | 1.272461 | 2.415591 |
| C | 3.365317 | 0.063917 | 5.214931 |
| H | 4.816412 | -1.530886 | 5.245519 |
| H | 1.916966 | 1.625066 | 4.860990 |
| H | 3.396264 | 0.223572 | 6.289433 |
| C | 5.520635 | -3.458958 | -2.304691 |
| C | 6.170717 | -3.070374 | -3.476770 |
| C | 6.926863 | -1.897186 | -3.511330 |
| C | 7.035106 | -1.121522 | -2.355179 |
| C | 6.396521 | -1.511705 | -1.177317 |
| C | 5.621246 | -2.684111 | -1.136506 |
| H | 4.933301 | -4.372906 | -2.284880 |
| H | 6.082411 | -3.687599 | -4.367816 |
| H | 7.429912 | -1.594166 | -4.425587 |
| H | 7.631388 | -0.211820 | -2.365060 |
| H | 6.502804 | -0.913305 | -0.277124 |
| S | 4.800717 | -3.200656 | 0.365438 |
| H | 4.037111 | -1.743162 | 0.661501 |
| H | -6.625532 | 1.021518 | 0.470036 |
| H | -8.863910 | 0.510894 | -0.451997 |
| H | -9.569736 | -1.853774 | -0.779274 |
| C | -3.183343 | -1.356126 | -2.596199 |
| C | -0.409924 | 3.573521 | -0.617452 |
| C | -1.219691 | 4.711349 | -0.501599 |
| C | 0.993833 | 3.705650 | -0.578210 |
| C | -0.596559 | 5.944570 | -0.348220 |
| H | -2.296906 | 4.643806 | -0.529421 |
| C | 1.545131 | 4.963934 | -0.417593 |
| H | 1.647259 | 2.846431 | -0.696026 |
| C | 0.781121 | 6.118830 | -0.296729 |
| H | 1.231055 | 7.096002 | -0.173999 |
| F | -1.378555 | 7.037020 | -0.234877 |
| F | 2.894544 | 5.074836 | -0.378958 |
| C | -1.842369 | -1.929157 | -3.093354 |

| | | | |
|---|---|---|---|
| H | -1.465617 | -2.705145 | -2.422506 |
| H | -1.977716 | -2.368678 | -4.088700 |
| H | -1.084940 | -1.141902 | -3.187082 |
| C | -3.682529 | -0.306207 | -3.608656 |
| H | -2.992844 | 0.540740 | -3.680274 |
| H | -3.769643 | -0.757901 | -4.604151 |
| H | -4.664508 | 0.088654 | -3.326686 |
| C | -4.216517 | -2.494584 | -2.496959 |
| H | -3.877279 | -3.291139 | -1.824624 |
| H | -5.185488 | -2.132374 | -2.137023 |
| H | -4.373944 | -2.942264 | -3.485166 |

TS Conformation 43

B3LYP/6-31G(d) Energy = -3411.893537
M06-2X/def2tzvpp/IEFPCM(chloroform) Energy = -3411.892412
M06-2X/def2tzvpp/IEFPCM(chloroform)//B3LYP/6-31G(d) Quasiharmonic Free Energy = -3411.165967

Frequencies (Top 3 out of 291)

1. -993.5024 cm-1
2. 6.2821 cm-1
3. 9.9630 cm-1

B3LYP/6-31G(d) Molecular Geometry in Cartesian Coordinates

| | | | |
|---|---|---|---|
| C | -2.775287 | -1.618843 | -0.031017 |
| C | -3.063269 | -0.651380 | -1.197682 |
| H | -3.992792 | -0.107385 | -1.035552 |
| C | -2.103273 | 1.679752 | -0.948898 |
| N | -0.868287 | 2.253710 | -0.764200 |
| H | -0.107572 | 1.584593 | -0.688520 |
| N | -1.981539 | 0.331146 | -1.151490 |
| H | -1.075366 | -0.077782 | -0.940841 |
| C | -4.714310 | -0.675745 | 1.322912 |
| C | -3.260488 | -2.457101 | 2.206203 |
| C | -4.756260 | -0.697234 | 2.872937 |
| H | -4.430165 | 0.321797 | 0.976809 |
| C | -4.350138 | -2.132708 | 3.235156 |
| H | -3.213558 | -3.514520 | 1.929045 |
| H | -2.263236 | -2.168416 | 2.561536 |
| H | -5.738307 | -0.410202 | 3.256769 |
| H | -4.017528 | 0.014284 | 3.261110 |
| H | -5.203048 | -2.810100 | 3.119667 |
| H | -3.986980 | -2.227860 | 4.262544 |
| N | -3.615401 | -1.632864 | 1.033732 |
| O | -1.757347 | -2.330464 | -0.048766 |
| S | -3.583982 | 2.487386 | -0.953234 |
| C | -6.049618 | -1.043741 | 0.689283 |
| C | -6.450577 | -2.370610 | 0.486779 |
| C | -6.934092 | -0.013376 | 0.341234 |
| C | -7.712230 | -2.660714 | -0.036032 |
| H | -5.766592 | -3.182520 | 0.716643 |
| C | -8.194984 | -0.301555 | -0.181777 |
| C | -8.589791 | -1.627493 | -0.368617 |
| H | -8.006100 | -3.695894 | -0.188830 |
| O | 3.238850 | 0.870239 | -0.999220 |
| O | 0.570399 | -0.047973 | 0.334602 |
| C | 3.737520 | 0.564650 | 0.079960 |
| C | 3.274669 | -0.559031 | 0.937796 |
| O | 4.792778 | 1.195408 | 0.612884 |

| | | | |
|---|---|---|---|
| C | 5.318119 | 2.293060 | -0.163428 |
| H | 6.187784 | 2.641775 | 0.393239 |
| H | 5.606476 | 1.947956 | -1.158912 |
| H | 4.573786 | 3.088298 | -0.251202 |
| S | 1.670511 | -1.060462 | 0.340159 |
| C | 1.866399 | -1.795889 | -1.295017 |
| H | 2.652370 | -2.554182 | -1.223836 |
| H | 2.152623 | -0.985817 | -1.964348 |
| H | 0.894001 | -2.224204 | -1.549830 |
| C | 1.202075 | -2.460647 | 1.366414 |
| H | 1.186693 | -2.115311 | 2.401008 |
| H | 1.947560 | -3.248480 | 1.224786 |
| H | 0.201253 | -2.748312 | 1.028881 |
| C | 3.290870 | -0.347212 | 2.436953 |
| C | 4.122837 | -1.131562 | 3.249568 |
| C | 2.499647 | 0.650267 | 3.034606 |
| C | 4.160411 | -0.922111 | 4.629304 |
| H | 4.752764 | -1.890783 | 2.796816 |
| C | 2.532933 | 0.849234 | 4.414123 |
| H | 1.858630 | 1.271925 | 2.415571 |
| C | 3.364475 | 0.062998 | 5.214953 |
| H | 4.815716 | -1.531673 | 5.245501 |
| H | 1.916036 | 1.624073 | 4.861045 |
| H | 3.395214 | 0.222454 | 6.289490 |
| C | 5.521567 | -3.458158 | -2.304893 |
| C | 6.171995 | -3.069253 | -3.476674 |
| C | 6.928212 | -1.896094 | -3.510669 |
| C | 7.036174 | -1.120786 | -2.354253 |
| C | 6.397238 | -1.511290 | -1.176687 |
| C | 5.621890 | -2.683668 | -1.136447 |
| H | 4.934184 | -4.372084 | -2.285521 |
| H | 6.083906 | -3.686205 | -4.367930 |
| H | 7.431532 | -1.592825 | -4.424694 |
| H | 7.632511 | -0.211115 | -2.363691 |
| H | 6.503299 | -0.913165 | -0.276285 |
| S | 4.800921 | -3.200622 | 0.365117 |
| H | 4.037207 | -1.743199 | 0.661334 |
| H | -6.625779 | 1.021488 | 0.469947 |
| H | -8.864127 | 0.510847 | -0.452151 |
| H | -9.569881 | -1.853824 | -0.779559 |
| C | -3.183332 | -1.356105 | -2.596312 |
| C | -0.410080 | 3.573483 | -0.617212 |
| C | 0.993675 | 3.705589 | -0.577792 |
| C | -1.219843 | 4.711320 | -0.501423 |
| C | 1.544975 | 4.963860 | -0.417062 |
| H | 1.647105 | 2.846368 | -0.695563 |
| C | -0.596710 | 5.944526 | -0.347920 |
| H | -2.297056 | 4.643794 | -0.529380 |
| C | 0.780967 | 6.118762 | -0.296250 |
| H | 1.230900 | 7.095922 | -0.173427 |
| F | 2.894384 | 5.074740 | -0.378255 |
| F | -1.378702 | 7.036984 | -0.234637 |
| C | -1.842283 | -1.929023 | -3.093402 |
| H | -1.465482 | -2.704946 | -2.422505 |
| H | -1.977551 | -2.368594 | -4.088736 |
| H | -1.084930 | -1.141693 | -3.187133 |
| C | -3.682534 | -0.306193 | -3.608769 |
| H | -2.992923 | 0.540822 | -3.680299 |
| H | -3.769528 | -0.757856 | -4.604289 |
| H | -4.664573 | 0.088564 | -3.326861 |
| C | -4.216429 | -2.494642 | -2.497166 |
| H | -3.877151 | -3.291208 | -1.824865 |

| | | | |
|---|---|---|---|
| H | -5.185436 | -2.132519 | -2.137239 |
| H | -4.373796 | -2.942277 | -3.485402 |

TS Conformation 44

B3LYP/6-31G(d) Energy = -3411.889569
M06-2X/def2tzvpp/IEFPCM(chloroform) Energy = -3411.891869
M06-2X/def2tzvpp/IEFPCM(chloroform)//B3LYP/6-31G(d) Quasiharmonic Free Energy = -3411.165956

Frequencies (Top 3 out of 291)

1. -1017.6357 cm-1
2. 8.2121 cm-1
3. 9.2390 cm-1

B3LYP/6-31G(d) Molecular Geometry in Cartesian Coordinates

| | | | |
|---|---|---|---|
| C | -2.861194 | -1.821034 | -0.712240 |
| C | -3.102692 | -0.855320 | -1.890728 |
| H | -4.148262 | -0.553217 | -1.931187 |
| C | -2.907714 | 1.495064 | -1.054124 |
| N | -1.944612 | 2.339541 | -0.559039 |
| H | -1.014990 | 1.926243 | -0.495151 |
| N | -2.351410 | 0.357758 | -1.577262 |
| H | -1.367092 | 0.192437 | -1.381038 |
| C | -3.730737 | -3.246314 | 1.082261 |
| C | -5.363311 | -2.215090 | -0.442116 |
| C | -5.086502 | -3.985574 | 1.173889 |
| H | -2.911678 | -3.935690 | 0.858753 |
| C | -6.098801 | -2.931956 | 0.701365 |
| H | -5.637695 | -1.159158 | -0.523611 |
| H | -5.566398 | -2.701854 | -1.403123 |
| H | -5.286053 | -4.357597 | 2.182058 |
| H | -5.081178 | -4.841954 | 0.488218 |
| H | -6.316082 | -2.227691 | 1.511276 |
| H | -7.047577 | -3.363570 | 0.369955 |
| N | -3.935025 | -2.366303 | -0.085253 |
| O | -1.706539 | -2.038226 | -0.319365 |
| S | -4.573887 | 1.778774 | -1.038123 |
| C | -3.380313 | -2.490577 | 2.361162 |
| C | -3.757974 | -1.159727 | 2.578353 |
| C | -2.698248 | -3.168703 | 3.380220 |
| C | -3.468844 | -0.526733 | 3.790238 |
| H | -4.261786 | -0.602039 | 1.793664 |
| C | -2.408644 | -2.540020 | 4.590967 |
| C | -2.796377 | -1.214350 | 4.801634 |
| H | -3.770310 | 0.506779 | 3.939251 |
| O | 0.634140 | 0.881006 | -0.423491 |
| O | 1.967058 | -0.569044 | -3.085972 |
| C | 1.325782 | 0.045847 | 0.174164 |
| C | 2.621713 | -0.475664 | -0.319237 |
| O | 1.046642 | -0.427390 | 1.380551 |
| C | -0.194771 | -0.014174 | 1.993855 |
| H | -0.179305 | -0.456284 | 2.988524 |
| H | -0.243804 | 1.075103 | 2.059586 |
| H | -1.031849 | -0.411427 | 1.419477 |
| S | 2.849347 | -0.009608 | -2.032699 |
| C | 2.909134 | 1.795026 | -2.107540 |
| H | 3.547485 | 2.145459 | -1.287137 |
| H | 3.312431 | 2.053252 | -3.089455 |
| H | 1.885314 | 2.144861 | -1.991780 |

| | | | |
|---|---|---|---|
| C | 4.561156 | -0.468322 | -2.371459 |
| H | 4.761224 | -0.154309 | -3.398274 |
| H | 5.227406 | 0.026483 | -1.660300 |
| H | 4.633099 | -1.552890 | -2.282715 |
| C | 2.906173 | -1.940238 | -0.091369 |
| C | 1.908169 | -2.903326 | -0.325275 |
| C | 4.166878 | -2.363055 | 0.356327 |
| C | 2.172717 | -4.255778 | -0.117959 |
| H | 0.917482 | -2.592622 | -0.645812 |
| C | 4.429086 | -3.719599 | 0.555405 |
| H | 4.940733 | -1.631437 | 0.564341 |
| C | 3.434408 | -4.668444 | 0.317042 |
| H | 1.390203 | -4.987684 | -0.297997 |
| H | 5.409757 | -4.029906 | 0.905663 |
| H | 3.638278 | -5.724102 | 0.475790 |
| C | 7.050875 | 1.381856 | -0.118139 |
| C | 8.358257 | 0.920153 | -0.284143 |
| C | 8.869688 | -0.069683 | 0.557171 |
| C | 8.062517 | -0.584847 | 1.574068 |
| C | 6.756476 | -0.123088 | 1.744275 |
| C | 6.220818 | 0.861616 | 0.892873 |
| H | 6.668138 | 2.171784 | -0.759292 |
| H | 8.981029 | 1.342686 | -1.069301 |
| H | 9.887808 | -0.427204 | 0.429345 |
| H | 8.453793 | -1.344634 | 2.246592 |
| H | 6.140297 | -0.511329 | 2.550137 |
| S | 4.557760 | 1.475068 | 1.129354 |
| H | 3.602546 | 0.343974 | 0.352747 |
| H | -2.385009 | -4.198608 | 3.220733 |
| H | -1.875269 | -3.082751 | 5.367067 |
| H | -2.572280 | -0.721600 | 5.743908 |
| C | -2.727988 | -1.442999 | -3.294504 |
| C | -1.985552 | 3.651830 | -0.056071 |
| C | -3.084272 | 4.518082 | -0.145907 |
| C | -0.792320 | 4.102182 | 0.544989 |
| C | -2.956000 | 5.798945 | 0.376942 |
| H | -4.014058 | 4.208306 | -0.597895 |
| C | -0.731573 | 5.393334 | 1.043589 |
| H | 0.078652 | 3.459871 | 0.621774 |
| C | -1.800966 | 6.279096 | 0.980814 |
| H | -1.736637 | 7.284101 | 1.378586 |
| F | -4.017142 | 6.626221 | 0.287656 |
| F | 0.416787 | 5.807602 | 1.615760 |
| C | -1.241124 | -1.826873 | -3.402967 |
| H | -0.968205 | -2.601952 | -2.682025 |
| H | -1.034627 | -2.209047 | -4.410069 |
| H | -0.577443 | -0.971543 | -3.244127 |
| C | -3.060209 | -0.378502 | -4.358842 |
| H | -2.464338 | 0.527537 | -4.213148 |
| H | -2.845397 | -0.768721 | -5.360768 |
| H | -4.117950 | -0.092587 | -4.324795 |
| C | -3.590669 | -2.694211 | -3.554062 |
| H | -3.405441 | -3.481707 | -2.813677 |
| H | -4.661060 | -2.455337 | -3.544084 |
| H | -3.357643 | -3.111308 | -4.540366 |

TS Conformation 45

B3LYP/6-31G(d) Energy = -3411.889419
M06-2X/def2tzvpp/IEFPCM(chloroform) Energy = -3411.891188
M06-2X/def2tzvpp/IEFPCM(chloroform)//B3LYP/6-31G(d) Quasiharmonic Free Energy = -3411.165858

Frequencies (Top 3 out of 291)

1. -965.3703 cm-1
2. 3.9619 cm-1
3. 9.9165 cm-1

B3LYP/6-31G(d) Molecular Geometry in Cartesian Coordinates

| | | | |
|---|---|---|---|
| C | 3.081110 | -1.330170 | -0.022570 |
| C | 3.177060 | -0.214920 | 1.039850 |
| H | 4.007340 | 0.455650 | 0.824870 |
| C | 1.880130 | 1.873280 | 0.475030 |
| N | 0.576310 | 2.296600 | 0.365450 |
| H | -0.131150 | 1.577531 | 0.494710 |
| N | 1.960600 | 0.586280 | 0.916970 |
| H | 1.095460 | 0.070970 | 1.048110 |
| C | 4.929330 | -0.282840 | -1.436140 |
| C | 3.769860 | -2.341720 | -2.126420 |
| C | 4.996390 | -0.453000 | -2.975600 |
| H | 4.508910 | 0.696730 | -1.192950 |
| C | 4.809530 | -1.961820 | -3.188660 |
| H | 3.894819 | -3.355520 | -1.733050 |
| H | 2.742750 | -2.266680 | -2.503510 |
| H | 5.931020 | -0.065230 | -3.388760 |
| H | 4.166650 | 0.100530 | -3.431360 |
| H | 5.752880 | -2.489620 | -3.010910 |
| H | 4.474370 | -2.212530 | -4.199360 |
| N | 3.965850 | -1.344400 | -1.054140 |
| O | 2.183380 | -2.180050 | 0.051870 |
| S | 3.231540 | 2.816300 | 0.099270 |
| C | 6.293740 | -0.403200 | -0.769490 |
| C | 6.855760 | -1.634480 | -0.408260 |
| C | 7.039420 | 0.764150 | -0.552660 |
| C | 8.137670 | -1.698631 | 0.141570 |
| H | 6.281120 | -2.547300 | -0.536360 |
| C | 8.320220 | 0.702229 | -0.003100 |
| C | 8.875570 | -0.531121 | 0.342840 |
| H | 8.556960 | -2.662761 | 0.417590 |
| O | -2.959350 | 0.645831 | -1.025360 |
| O | -1.020170 | -0.122709 | 1.396660 |
| C | -3.087660 | -0.563929 | -1.177120 |
| C | -2.998320 | -1.568489 | -0.083270 |
| O | -3.379440 | -1.137139 | -2.351360 |
| C | -3.551650 | -0.230199 | -3.458500 |
| H | -4.376150 | 0.458241 | -3.258540 |
| H | -2.636190 | 0.341291 | -3.628720 |
| H | -3.775290 | -0.865559 | -4.315010 |
| S | -2.378310 | -0.747959 | 1.376090 |
| C | -3.612440 | 0.463911 | 1.895960 |
| H | -3.613120 | 1.248591 | 1.140960 |
| H | -3.284100 | 0.837521 | 2.868610 |
| H | -4.580170 | -0.042399 | 1.951190 |
| C | -2.484350 | -2.019849 | 2.646470 |
| H | -2.182710 | -1.550319 | 3.584950 |
| H | -1.794050 | -2.817619 | 2.369940 |
| H | -3.520020 | -2.374319 | 2.676910 |
| C | -2.305561 | -2.883909 | -0.371600 |
| C | -3.081971 | -4.026049 | -0.623240 |
| C | -0.905231 | -2.984159 | -0.424190 |
| C | -2.464281 | -5.241319 | -0.920380 |
| H | -4.164451 | -3.957729 | -0.591790 |

| | | | |
|---|---|---|---|
| C | -0.292661 | -4.207129 | -0.698470 |
| H | -0.272860 | -2.120639 | -0.245930 |
| C | -1.071551 | -5.337479 | -0.951550 |
| H | -3.076781 | -6.116429 | -1.120270 |
| H | 0.791639 | -4.249430 | -0.714780 |
| H | -0.596381 | -6.289439 | -1.173990 |
| C | -7.483780 | -0.179358 | 1.350450 |
| C | -8.217240 | 0.924202 | 0.912060 |
| C | -8.194850 | 1.300472 | -0.432210 |
| C | -7.439000 | 0.551922 | -1.337100 |
| C | -6.712200 | -0.558138 | -0.905530 |
| C | -6.715680 | -0.937588 | 0.448990 |
| H | -7.507830 | -0.468368 | 2.397730 |
| H | -8.807680 | 1.492872 | 1.626680 |
| H | -8.763390 | 2.161892 | -0.771660 |
| H | -7.423420 | 0.827202 | -2.389280 |
| H | -6.145330 | -1.149679 | -1.618880 |
| S | -5.791640 | -2.358679 | 1.015450 |
| H | -4.294730 | -1.896569 | 0.351570 |
| H | 6.606390 | 1.728530 | -0.807860 |
| H | 8.879980 | 1.618879 | 0.162840 |
| H | 9.871660 | -0.581231 | 0.774290 |
| C | 3.379480 | -0.752610 | 2.501660 |
| C | 0.038950 | 3.553691 | 0.013520 |
| C | 0.656921 | 4.772330 | 0.324930 |
| C | -1.219380 | 3.541881 | -0.613320 |
| C | 0.003911 | 5.947551 | -0.021610 |
| H | 1.619721 | 4.812980 | 0.811850 |
| C | -1.822409 | 4.752991 | -0.920820 |
| H | -1.717290 | 2.614621 | -0.877350 |
| C | -1.238369 | 5.982841 | -0.643800 |
| H | -1.722889 | 6.916921 | -0.899710 |
| F | 0.599721 | 7.119660 | 0.278600 |
| F | -3.027949 | 4.735981 | -1.526940 |
| C | 4.568790 | -1.731700 | 2.516800 |
| H | 5.476750 | -1.273870 | 2.110120 |
| H | 4.782830 | -2.041200 | 3.546560 |
| H | 4.352700 | -2.636680 | 1.937950 |
| C | 2.129950 | -1.468790 | 3.052740 |
| H | 1.845340 | -2.314700 | 2.422860 |
| H | 2.338630 | -1.837380 | 4.064500 |
| H | 1.274040 | -0.787470 | 3.125530 |
| C | 3.707360 | 0.454670 | 3.402710 |
| H | 4.632250 | 0.950740 | 3.087810 |
| H | 2.904870 | 1.199370 | 3.380660 |
| H | 3.835460 | 0.126280 | 4.441210 |

TS Conformation 46

B3LYP/6-31G(d) Energy = -3411.886351
M06-2X/def2tzvpp/IEFPCM(chloroform) Energy = -3411.891478
M06-2X/def2tzvpp/IEFPCM(chloroform)//B3LYP/6-31G(d) Quasiharmonic Free Energy = -3411.165707

Frequencies (Top 3 out of 291)

1. -981.6479 cm-1
2. 7.3042 cm-1
3. 10.0174 cm-1

B3LYP/6-31G(d) Molecular Geometry in Cartesian Coordinates

| | | | |
|---|---|---|---|
| C | 3.149059 | -0.756060 | 1.434601 |
| C | 2.780257 | 0.497725 | 2.256087 |
| H | 3.577889 | 1.236349 | 2.191676 |
| C | 1.658376 | 2.195276 | 0.789882 |
| N | 0.408444 | 2.535615 | 0.346779 |
| H | -0.346098 | 1.957910 | 0.718523 |
| N | 1.617292 | 1.116817 | 1.632998 |
| H | 0.797293 | 0.520521 | 1.578578 |
| C | 4.790876 | -2.044353 | 0.150256 |
| C | 5.533073 | 0.086461 | 1.125044 |
| C | 6.336559 | -1.992317 | 0.212197 |
| H | 4.399785 | -2.932304 | 0.656446 |
| C | 6.637167 | -0.486897 | 0.225384 |
| H | 5.241888 | 1.103373 | 0.848404 |
| H | 5.847645 | 0.083901 | 2.176148 |
| H | 6.801990 | -2.522326 | -0.622837 |
| H | 6.679216 | -2.456288 | 1.145394 |
| H | 6.550814 | -0.075589 | -0.786189 |
| H | 7.636840 | -0.249277 | 0.600283 |
| N | 4.406224 | -0.853158 | 0.928070 |
| O | 2.304250 | -1.636895 | 1.229957 |
| S | 3.095338 | 3.003914 | 0.405714 |
| C | 4.265432 | -2.041307 | -1.283625 |
| C | 3.829291 | -0.877303 | -1.926826 |
| C | 4.274201 | -3.244938 | -2.003900 |
| C | 3.433658 | -0.912780 | -3.266842 |
| H | 3.783469 | 0.060450 | -1.380991 |
| C | 3.878892 | -3.283929 | -3.340931 |
| C | 3.462470 | -2.112388 | -3.980812 |
| H | 3.101181 | 0.002844 | -3.748330 |
| O | -2.195237 | 0.096870 | -2.904541 |
| O | 0.106704 | -0.316185 | -0.900947 |
| C | -2.891926 | -0.446006 | -2.058010 |
| C | -2.407394 | -1.370013 | -1.004264 |
| O | -4.213616 | -0.212947 | -1.929513 |
| C | -4.760826 | 0.756016 | -2.848054 |
| H | -5.822465 | 0.808294 | -2.606556 |
| H | -4.281706 | 1.726865 | -2.707209 |
| H | -4.616698 | 0.426551 | -3.880255 |
| S | -0.643469 | -1.556326 | -1.188656 |
| C | -0.172920 | -2.870125 | -0.050794 |
| H | -0.585301 | -3.823564 | -0.382659 |
| H | 0.919086 | -2.853470 | -0.016992 |
| H | -0.559919 | -2.590657 | 0.930664 |
| C | -0.172018 | -2.213250 | -2.812096 |
| H | 0.912205 | -2.350177 | -2.812891 |
| H | -0.700689 | -3.155285 | -2.976208 |
| H | -0.477006 | -1.453530 | -3.531839 |
| C | -3.177361 | -2.638344 | -0.747070 |
| C | -3.461302 | -3.053005 | 0.563957 |
| C | -3.640691 | -3.428731 | -1.814677 |
| C | -4.175408 | -4.229001 | 0.798523 |
| H | -3.137367 | -2.440973 | 1.399951 |
| C | -4.357606 | -4.600851 | -1.580247 |
| H | -3.452089 | -3.110858 | -2.837808 |
| C | -4.622061 | -5.007196 | -0.270170 |
| H | -4.391150 | -4.529838 | 1.819998 |
| H | -4.711357 | -5.194751 | -2.418772 |
| H | -5.180993 | -5.920482 | -0.085110 |
| C | -3.711991 | 0.591283 | 3.791795 |
| C | -4.939437 | 0.706833 | 4.443285 |
| C | -6.133683 | 0.620671 | 3.724544 |

| | | | |
|---|---|---|---|
| C | -6.085036 | 0.423993 | 2.343441 |
| C | -4.860682 | 0.319718 | 1.681756 |
| C | -3.657061 | 0.398951 | 2.401626 |
| H | -2.787006 | 0.649385 | 4.358491 |
| H | -4.960659 | 0.859582 | 5.519733 |
| H | -7.089412 | 0.707098 | 4.234409 |
| H | -7.006901 | 0.356989 | 1.770353 |
| H | -4.837819 | 0.179332 | 0.605260 |
| S | -2.067341 | 0.272210 | 1.597909 |
| H | -2.365743 | -0.560867 | 0.238551 |
| H | 4.600686 | -4.160132 | -1.513041 |
| H | 3.900375 | -4.225588 | -3.883948 |
| H | 3.163909 | -2.135920 | -5.025798 |
| C | 2.539094 | 0.196520 | 3.777405 |
| C | -0.053851 | 3.425858 | -0.639980 |
| C | 0.702695 | 4.448834 | -1.229427 |
| C | -1.400380 | 3.250727 | -1.016571 |
| C | 0.092083 | 5.249891 | -2.186405 |
| H | 1.732888 | 4.619085 | -0.956816 |
| C | -1.944326 | 4.087451 | -1.973052 |
| H | -2.006684 | 2.469069 | -0.572544 |
| C | -1.228642 | 5.105833 | -2.591203 |
| H | -1.673567 | 5.749560 | -3.339580 |
| F | 0.826856 | 6.231054 | -2.752344 |
| F | -3.239306 | 3.903587 | -2.332215 |
| C | 2.314762 | 1.541695 | 4.496175 |
| H | 1.433067 | 2.058288 | 4.105063 |
| H | 2.162224 | 1.375310 | 5.569195 |
| H | 3.176448 | 2.209383 | 4.377034 |
| C | 1.323828 | -0.719319 | 4.022449 |
| H | 1.443783 | -1.687800 | 3.530700 |
| H | 1.207023 | -0.888954 | 5.099916 |
| H | 0.392036 | -0.269369 | 3.662025 |
| C | 3.800663 | -0.475805 | 4.355095 |
| H | 3.999724 | -1.446434 | 3.885803 |
| H | 4.688266 | 0.156633 | 4.230104 |
| H | 3.672987 | -0.651443 | 5.429376 |

TS Conformation 47

B3LYP/6-31G(d) Energy = -3411.889329
M06-2X/def2tzvpp/IEFPCM(chloroform) Energy = -3411.892822
M06-2X/def2tzvpp/IEFPCM(chloroform)//B3LYP/6-31G(d) Quasiharmonic Free Energy = -3411.165625

Frequencies (Top 3 out of 291)

1. -764.2688 cm-1
2. 13.9264 cm-1
3. 15.1930 cm-1

B3LYP/6-31G(d) Molecular Geometry in Cartesian Coordinates

| | | | |
|---|---|---|---|
| C | -2.599510 | -1.534818 | -0.330121 |
| C | -2.781314 | -0.331147 | -1.277284 |
| H | -3.600740 | 0.304543 | -0.949021 |
| C | -1.474215 | 1.742971 | -0.670146 |
| N | -0.163638 | 2.130307 | -0.527553 |
| H | 0.515405 | 1.399687 | -0.720180 |
| N | -1.561147 | 0.460019 | -1.132675 |
| H | -0.698763 | -0.074390 | -1.200545 |
| C | -4.405457 | -0.646530 | 1.236279 |

| | | | |
|---|---|---|---|
| C | -3.106385 | -2.647772 | 1.795588 |
| C | -4.588984 | -1.026240 | 2.733838 |
| H | -3.947070 | 0.344447 | 1.166692 |
| C | -3.358942 | -1.871820 | 3.089393 |
| H | -3.777488 | -3.513179 | 1.707162 |
| H | -2.084074 | -3.002502 | 1.671720 |
| H | -5.501354 | -1.620809 | 2.851718 |
| H | -4.699306 | -0.130814 | 3.351815 |
| H | -3.528766 | -2.528774 | 3.948008 |
| H | -2.494082 | -1.236081 | 3.312229 |
| N | -3.415939 | -1.658271 | 0.746710 |
| O | -1.668249 | -2.335187 | -0.536446 |
| S | -2.823472 | 2.704588 | -0.345398 |
| C | -5.720402 | -0.632262 | 0.474039 |
| C | -6.271603 | 0.586115 | 0.060922 |
| C | -6.427411 | -1.815929 | 0.216733 |
| C | -7.505378 | 0.622918 | -0.594689 |
| H | -5.724300 | 1.508055 | 0.239977 |
| C | -7.656133 | -1.781173 | -0.439881 |
| C | -8.200523 | -0.559424 | -0.846647 |
| H | -7.917091 | 1.577306 | -0.911595 |
| O | 3.634670 | -4.106286 | -0.061852 |
| O | 1.350839 | -0.488777 | -0.898145 |
| C | 4.284571 | -3.076819 | -0.219427 |
| C | 3.708123 | -1.711387 | -0.349258 |
| O | 5.620938 | -3.032545 | -0.199808 |
| C | 6.286701 | -4.274384 | 0.099792 |
| H | 6.034481 | -5.034331 | -0.644000 |
| H | 7.350545 | -4.041272 | 0.069690 |
| H | 5.996984 | -4.626222 | 1.092505 |
| S | 1.920270 | -1.827625 | -0.582139 |
| C | 1.448188 | -2.975150 | -1.898420 |
| H | 1.895635 | -3.945073 | -1.680494 |
| H | 0.355222 | -3.003019 | -1.890769 |
| H | 1.827449 | -2.550530 | -2.830205 |
| C | 1.185751 | -2.537557 | 0.896808 |
| H | 1.386849 | -1.843480 | 1.716640 |
| H | 0.116928 | -2.621357 | 0.677973 |
| H | 1.665123 | -3.502106 | 1.066306 |
| C | 4.319059 | -0.741271 | -1.325416 |
| C | 4.459336 | 0.605855 | -0.963288 |
| C | 4.738070 | -1.150019 | -2.602444 |
| C | 4.995796 | 1.528494 | -1.863047 |
| H | 4.177846 | 0.914217 | 0.038649 |
| C | 5.282294 | -0.230412 | -3.497103 |
| H | 4.656120 | -2.195684 | -2.890647 |
| C | 5.404596 | 1.112539 | -3.131098 |
| H | 5.082609 | 2.570394 | -1.569376 |
| H | 5.608615 | -0.560991 | -4.479443 |
| H | 5.823367 | 1.830364 | -3.831211 |
| C | 1.889145 | 0.800787 | 2.683444 |
| C | 0.629755 | 1.095438 | 3.207695 |
| C | 0.094405 | 0.322341 | 4.241777 |
| C | 0.835994 | -0.752238 | 4.740926 |
| C | 2.090664 | -1.055333 | 4.209662 |
| C | 2.648076 | -0.285323 | 3.167231 |
| H | 2.306433 | 1.430263 | 1.902759 |
| H | 0.073053 | 1.943405 | 2.815732 |
| H | -0.876547 | 0.566595 | 4.665467 |
| H | 0.438633 | -1.357307 | 5.553192 |
| H | 2.662723 | -1.890804 | 4.603344 |
| S | 4.257327 | -0.678094 | 2.512331 |

| | | | |
|---|---|---|---|
| H | 3.841438 | -1.211897 | 0.890999 |
| H | -6.008888 | -2.772145 | 0.521539 |
| H | -8.189678 | -2.707518 | -0.636122 |
| H | -9.158561 | -0.532639 | -1.358723 |
| C | -3.075277 | -0.720150 | -2.771321 |
| C | 0.454341 | 3.363302 | -0.244152 |
| C | -0.182341 | 4.483839 | 0.306187 |
| C | 1.833071 | 3.415080 | -0.529938 |
| C | 0.581937 | 5.620250 | 0.546104 |
| H | -1.236844 | 4.480481 | 0.535959 |
| C | 2.535545 | 4.576909 | -0.258676 |
| H | 2.349232 | 2.568934 | -0.970418 |
| C | 1.942621 | 5.711588 | 0.281190 |
| H | 2.507362 | 6.612522 | 0.485337 |
| F | -0.035542 | 6.694722 | 1.075296 |
| F | 3.857386 | 4.611653 | -0.542678 |
| C | -1.884885 | -1.416618 | -3.457881 |
| H | -1.672687 | -2.387123 | -3.003220 |
| H | -2.119367 | -1.578500 | -4.516750 |
| H | -0.977603 | -0.802534 | -3.416466 |
| C | -3.402117 | 0.578160 | -3.536409 |
| H | -4.272097 | 1.087059 | -3.108290 |
| H | -2.562278 | 1.279892 | -3.514801 |
| H | -3.623751 | 0.347849 | -4.585376 |
| C | -4.299095 | -1.654581 | -2.819577 |
| H | -4.109775 | -2.599632 | -2.297161 |
| H | -5.183035 | -1.188936 | -2.373045 |
| H | -4.539438 | -1.897037 | -3.861466 |

TS Conformation 48

B3LYP/6-31G(d) Energy = -3411.891063
M06-2X/def2tzvpp/IEFPCM(chloroform) Energy = -3411.893498
M06-2X/def2tzvpp/IEFPCM(chloroform)//B3LYP/6-31G(d) Quasiharmonic Free Energy = -3411.165614

Frequencies (Top 3 out of 291)

1. -992.5442 cm-1
2. 13.5703 cm-1
3. 15.2862 cm-1

B3LYP/6-31G(d) Molecular Geometry in Cartesian Coordinates

| | | | |
|---|---|---|---|
| C | 2.582073 | -1.094126 | 0.439211 |
| C | 2.758655 | 0.258314 | 1.162481 |
| H | 3.530261 | 0.863067 | 0.691421 |
| C | 1.313958 | 2.170606 | 0.356568 |
| N | -0.024651 | 2.497318 | 0.298082 |
| H | -0.639769 | 1.783082 | 0.677961 |
| N | 1.492347 | 0.966388 | 0.970803 |
| H | 0.669510 | 0.385046 | 1.113724 |
| C | 4.294081 | -0.435592 | -1.333124 |
| C | 3.045550 | -2.543265 | -1.478681 |
| C | 4.465187 | -1.083019 | -2.736637 |
| H | 3.789788 | 0.530425 | -1.438105 |
| C | 3.256153 | -2.012437 | -2.897849 |
| H | 3.724889 | -3.377566 | -1.253607 |
| H | 2.024794 | -2.861241 | -1.273709 |
| H | 5.393620 | -1.664199 | -2.760178 |
| H | 4.539900 | -0.316369 | -3.513444 |
| H | 3.429271 | -2.811434 | -3.626037 |

| | | | |
|---|---|---|---|
| H | 2.366888 | -1.453035 | -3.206917 |
| N | 3.371880 | -1.379685 | -0.632729 |
| O | 1.697052 | -1.868323 | 0.827318 |
| S | 2.583879 | 3.115539 | -0.229385 |
| C | 5.627833 | -0.214152 | -0.636967 |
| C | 6.408853 | -1.293774 | -0.199022 |
| C | 6.122603 | 1.084785 | -0.473491 |
| C | 7.654451 | -1.078513 | 0.387958 |
| H | 6.033165 | -2.308443 | -0.306693 |
| C | 7.372534 | 1.302599 | 0.112870 |
| C | 8.141798 | 0.222534 | 0.544287 |
| H | 8.245416 | -1.925750 | 0.726244 |
| O | -1.419329 | -0.053098 | 0.643718 |
| O | -0.076267 | -1.556300 | -2.169620 |
| C | -1.889965 | -1.190833 | 0.721074 |
| C | -2.282596 | -2.030530 | -0.432750 |
| O | -2.193557 | -1.784536 | 1.874503 |
| C | -2.022880 | -1.002100 | 3.072044 |
| H | -0.975359 | -0.721655 | 3.196756 |
| H | -2.655389 | -0.112655 | 3.031706 |
| H | -2.339002 | -1.657352 | 3.883190 |
| S | -1.538752 | -1.441651 | -1.949742 |
| C | -2.429817 | -2.379341 | -3.208524 |
| H | -2.046068 | -2.042866 | -4.173895 |
| H | -3.499645 | -2.177570 | -3.107777 |
| H | -2.212036 | -3.436209 | -3.051842 |
| C | -2.115442 | 0.241291 | -2.255612 |
| H | -3.194925 | 0.261712 | -2.068224 |
| H | -1.874673 | 0.463242 | -3.297988 |
| H | -1.573061 | 0.896026 | -1.577906 |
| C | -2.135414 | -3.528663 | -0.291342 |
| C | -3.241371 | -4.368611 | -0.491373 |
| C | -0.893240 | -4.092488 | 0.050775 |
| C | -3.106584 | -5.751623 | -0.358433 |
| H | -4.208870 | -3.933364 | -0.724539 |
| C | -0.766883 | -5.475271 | 0.180618 |
| H | -0.035372 | -3.448669 | 0.231334 |
| C | -1.869854 | -6.307128 | -0.026865 |
| H | -3.971238 | -6.392622 | -0.507934 |
| H | 0.195623 | -5.902117 | 0.449187 |
| H | -1.766970 | -7.384128 | 0.077109 |
| C | -5.369258 | 1.202636 | 0.294542 |
| C | -5.510875 | 2.022103 | 1.416674 |
| C | -5.692378 | 1.460335 | 2.682212 |
| C | -5.732841 | 0.069979 | 2.813864 |
| C | -5.589429 | -0.751273 | 1.693835 |
| C | -5.401588 | -0.197122 | 0.416011 |
| H | -5.238483 | 1.654736 | -0.684216 |
| H | -5.473417 | 3.100351 | 1.291755 |
| H | -5.808164 | 2.098345 | 3.554414 |
| H | -5.885682 | -0.380545 | 3.792072 |
| H | -5.629637 | -1.831183 | 1.800388 |
| S | -5.232181 | -1.257375 | -1.018521 |
| H | -3.647996 | -1.711258 | -0.682319 |
| H | 5.518394 | 1.930199 | -0.792874 |
| H | 7.738941 | 2.318323 | 0.235517 |
| H | 9.112649 | 0.390163 | 1.002841 |
| C | 3.134803 | 0.117615 | 2.683866 |
| C | -0.740052 | 3.582287 | -0.236054 |
| C | -0.173966 | 4.729886 | -0.809444 |
| C | -2.144701 | 3.460977 | -0.164908 |
| C | -1.028414 | 5.711543 | -1.296828 |

| | | | |
|---|---|---|---|
| H | 0.895778 | 4.857565 | -0.876301 |
| C | -2.935128 | 4.477470 | -0.673940 |
| H | -2.618520 | 2.594397 | 0.285347 |
| C | -2.414689 | 5.627965 | -1.253699 |
| H | -3.050239 | 6.412179 | -1.645347 |
| F | -0.476403 | 6.811092 | -1.847259 |
| F | -4.280312 | 4.341270 | -0.609362 |
| C | 1.955715 | -0.391776 | 3.534882 |
| H | 1.593787 | -1.359116 | 3.177764 |
| H | 2.275541 | -0.500655 | 4.578118 |
| H | 1.124517 | 0.323420 | 3.523789 |
| C | 3.557468 | 1.509058 | 3.195208 |
| H | 2.759123 | 2.246153 | 3.059980 |
| H | 3.795011 | 1.460568 | 4.264931 |
| H | 4.443967 | 1.876725 | 2.666912 |
| C | 4.319560 | -0.855683 | 2.832853 |
| H | 4.048508 | -1.871460 | 2.523893 |
| H | 5.184739 | -0.536428 | 2.243804 |
| H | 4.630037 | -0.901387 | 3.883465 |

TS Conformation 49

B3LYP/6-31G(d) Energy = -3411.890284
M06-2X/def2tzvpp/IEFPCM(chloroform) Energy = -3411.891532
M06-2X/def2tzvpp/IEFPCM(chloroform)//B3LYP/6-31G(d) Quasiharmonic Free Energy = -3411.165603

Frequencies (Top 3 out of 291)

1. -880.0122 cm-1
2. 4.5907 cm-1
3. 11.6138 cm-1

B3LYP/6-31G(d) Molecular Geometry in Cartesian Coordinates

| | | | |
|---|---|---|---|
| C | 2.951001 | -1.297250 | -0.292606 |
| C | 2.857905 | -0.310923 | 0.888425 |
| H | 3.693918 | 0.385628 | 0.872315 |
| C | 1.631327 | 1.807586 | 0.304257 |
| N | 0.350040 | 2.230944 | 0.042107 |
| H | -0.353197 | 1.498506 | 0.093746 |
| N | 1.657420 | 0.487720 | 0.654802 |
| H | 0.797400 | -0.046512 | 0.556660 |
| C | 5.102393 | -0.237650 | -1.162195 |
| C | 4.025974 | -2.131861 | -2.311000 |
| C | 5.481130 | -0.249439 | -2.665162 |
| H | 4.677906 | 0.734609 | -0.898825 |
| C | 5.280302 | -1.711641 | -3.087138 |
| H | 4.014606 | -3.187344 | -2.022959 |
| H | 3.109595 | -1.943486 | -2.883849 |
| H | 6.496934 | 0.117651 | -2.831020 |
| H | 4.789697 | 0.402485 | -3.212498 |
| H | 6.142162 | -2.316778 | -2.787053 |
| H | 5.152166 | -1.830751 | -4.166901 |
| N | 4.031632 | -1.268986 | -1.110193 |
| O | 2.009456 | -2.078468 | -0.523121 |
| S | 3.022790 | 2.763471 | 0.219484 |
| C | 6.290632 | -0.514777 | -0.251855 |
| C | 6.762617 | -1.808054 | 0.008810 |
| C | 6.972894 | 0.574212 | 0.307863 |
| C | 7.896205 | -2.006547 | 0.798292 |
| H | 6.229011 | -2.668559 | -0.384489 |

| | | | |
|---|---|---|---|
| C | 8.106165 | 0.377854 | 1.098291 |
| C | 8.573638 | -0.914316 | 1.343308 |
| H | 8.245925 | -3.016992 | 0.993008 |
| O | -2.876782 | -3.858930 | -2.251640 |
| O | -1.184018 | -0.509628 | -0.032326 |
| C | -3.662047 | -2.969617 | -1.931282 |
| C | -3.332812 | -1.805267 | -1.069341 |
| O | -4.961531 | -2.983300 | -2.252916 |
| C | -5.420119 | -4.148705 | -2.962650 |
| H | -4.887092 | -4.256529 | -3.910804 |
| H | -5.266873 | -5.045321 | -2.357516 |
| H | -6.482550 | -3.977952 | -3.133906 |
| S | -1.544468 | -1.724773 | -0.820442 |
| C | -1.016853 | -3.215299 | 0.035710 |
| H | -1.611266 | -3.253884 | 0.956362 |
| H | -1.242458 | -4.064049 | -0.609569 |
| H | 0.053354 | -3.085176 | 0.210563 |
| C | -0.619888 | -1.731564 | -2.374839 |
| H | -0.953524 | -2.594448 | -2.952706 |
| H | -0.853449 | -0.790698 | -2.877941 |
| H | 0.437422 | -1.796978 | -2.099214 |
| C | -3.901719 | -0.459254 | -1.424545 |
| C | -4.334931 | 0.411426 | -0.413328 |
| C | -4.003838 | -0.047226 | -2.764800 |
| C | -4.852760 | 1.665886 | -0.739281 |
| H | -4.271989 | 0.105088 | 0.624971 |
| C | -4.526120 | 1.203557 | -3.087322 |
| H | -3.690049 | -0.717570 | -3.561616 |
| C | -4.945302 | 2.067002 | -2.072907 |
| H | -5.177071 | 2.334518 | 0.052373 |
| H | -4.600768 | 1.505534 | -4.128492 |
| H | -5.334691 | 3.050363 | -2.318042 |
| C | -5.704041 | -1.295488 | 3.395132 |
| C | -5.905256 | -0.230898 | 4.272990 |
| C | -4.871510 | 0.667848 | 4.547834 |
| C | -3.629884 | 0.482288 | 3.937354 |
| C | -3.420886 | -0.590597 | 3.068513 |
| C | -4.456720 | -1.498105 | 2.775389 |
| H | -6.513588 | -1.986489 | 3.179482 |
| H | -6.878359 | -0.100075 | 4.741124 |
| H | -5.030797 | 1.499084 | 5.229655 |
| H | -2.813003 | 1.170146 | 4.144947 |
| H | -2.445679 | -0.731615 | 2.610528 |
| S | -4.223478 | -2.891537 | 1.686383 |
| H | -3.745799 | -2.182062 | 0.181797 |
| H | 6.606746 | 1.582723 | 0.129645 |
| H | 8.617678 | 1.234641 | 1.528554 |
| H | 9.453510 | -1.069882 | 1.961537 |
| C | 2.853210 | -0.981802 | 2.309305 |
| C | -0.215500 | 3.489828 | -0.241976 |
| C | 0.498018 | 4.688782 | -0.378208 |
| C | -1.616847 | 3.493202 | -0.387837 |
| C | -0.214086 | 5.849960 | -0.652989 |
| H | 1.571088 | 4.724963 | -0.272494 |
| C | -2.262953 | 4.687736 | -0.658930 |
| H | -2.200348 | 2.584084 | -0.288752 |
| C | -1.593813 | 5.897313 | -0.801755 |
| H | -2.117535 | 6.821288 | -1.012432 |
| F | 0.479557 | 6.998644 | -0.782886 |
| F | -3.608644 | 4.680446 | -0.788382 |
| C | 2.872571 | 0.150869 | 3.355965 |
| H | 1.980299 | 0.780525 | 3.282751 |

| | | | |
|---|---|---|---|
| H | 2.902665 | -0.274496 | 4.365760 |
| H | 3.749600 | 0.795891 | 3.232817 |
| C | 4.126274 | -1.833290 | 2.468430 |
| H | 4.155606 | -2.661671 | 1.750996 |
| H | 5.033922 | -1.236492 | 2.334216 |
| H | 4.157232 | -2.268887 | 3.473791 |
| C | 1.622302 | -1.873675 | 2.556505 |
| H | 0.681705 | -1.327209 | 2.422984 |
| H | 1.616022 | -2.743332 | 1.894860 |
| H | 1.637510 | -2.236636 | 3.590688 |

TS Conformation 50

B3LYP/6-31G(d) Energy = -3411.886702
M06-2X/def2tzvpp/IEFPCM(chloroform) Energy = -3411.890944
M06-2X/def2tzvpp/IEFPCM(chloroform)//B3LYP/6-31G(d) Quasiharmonic Free Energy = -3411.165574

Frequencies (Top 3 out of 291)

1. -936.5678 cm-1
2. 10.4934 cm-1
3. 10.8542 cm-1

B3LYP/6-31G(d) Molecular Geometry in Cartesian Coordinates

| | | | |
|---|---|---|---|
| C | 2.969698 | -1.219065 | 0.155366 |
| C | 2.975535 | -0.062173 | 1.181001 |
| H | 3.777274 | 0.643175 | 0.968853 |
| C | 1.566246 | 1.815302 | 0.276102 |
| N | 0.242355 | 2.090576 | 0.037024 |
| H | -0.427729 | 1.370108 | 0.320768 |
| N | 1.722427 | 0.666777 | 0.991608 |
| H | 0.889942 | 0.109906 | 1.163584 |
| C | 4.950916 | -0.253419 | -1.135558 |
| C | 3.819554 | -2.320443 | -1.839215 |
| C | 5.107223 | -0.462244 | -2.663547 |
| H | 4.528103 | 0.736680 | -0.946251 |
| C | 4.922905 | -1.974941 | -2.847901 |
| H | 3.916161 | -3.323089 | -1.410350 |
| H | 2.818135 | -2.253171 | -2.280786 |
| H | 6.066971 | -0.090181 | -3.031550 |
| H | 4.308169 | 0.084260 | -3.178498 |
| H | 5.850817 | -2.503078 | -2.602358 |
| H | 4.644901 | -2.250257 | -3.869628 |
| N | 3.951250 | -1.291518 | -0.786251 |
| O | 2.053046 | -2.049404 | 0.177037 |
| S | 2.846641 | 2.784668 | -0.250426 |
| C | 6.273724 | -0.373911 | -0.390068 |
| C | 6.799961 | -1.602371 | 0.029869 |
| C | 7.021568 | 0.789189 | -0.158516 |
| C | 8.047985 | -1.668103 | 0.652151 |
| H | 6.220749 | -2.510837 | -0.107731 |
| C | 8.268788 | 0.726248 | 0.463652 |
| C | 8.788305 | -0.504765 | 0.868429 |
| H | 8.438548 | -2.630148 | 0.973967 |
| O | -5.999701 | -1.606730 | -0.232940 |
| O | -2.435442 | -0.403328 | -2.402609 |
| C | -4.948855 | -2.141372 | 0.118493 |
| C | -3.614156 | -1.863152 | -0.448593 |
| O | -4.885547 | -3.025460 | 1.130225 |
| C | -6.135807 | -3.319328 | 1.778067 |

| | | | |
|---|---|---|---|
| H | -5.894598 | -4.056950 | 2.543291 |
| H | -6.553309 | -2.416095 | 2.230129 |
| H | -6.855071 | -3.726150 | 1.062503 |
| S | -3.756321 | -0.716825 | -1.813064 |
| C | -4.857567 | -1.345306 | -3.110354 |
| H | -5.825444 | -1.566009 | -2.658785 |
| H | -4.925427 | -0.581006 | -3.888263 |
| H | -4.379151 | -2.246649 | -3.499839 |
| C | -4.569216 | 0.787137 | -1.228201 |
| H | -4.423729 | 1.536128 | -2.010732 |
| H | -5.616062 | 0.568911 | -1.024933 |
| H | -4.053815 | 1.090901 | -0.313946 |
| C | -2.691776 | -3.006483 | -0.780048 |
| C | -3.201590 | -4.257691 | -1.173215 |
| C | -1.299890 | -2.843457 | -0.705195 |
| C | -2.343906 | -5.316759 | -1.460812 |
| H | -4.276576 | -4.409088 | -1.231820 |
| C | -0.439793 | -3.903501 | -0.993955 |
| H | -0.872643 | -1.890732 | -0.416571 |
| C | -0.960225 | -5.141308 | -1.371457 |
| H | -2.756068 | -6.278938 | -1.753577 |
| H | 0.629462 | -3.743251 | -0.898311 |
| H | -0.292783 | -5.970622 | -1.590962 |
| C | -4.324930 | 0.194863 | 2.930394 |
| C | -5.329264 | 0.974447 | 3.504212 |
| C | -5.251767 | 2.368585 | 3.466839 |
| C | -4.158611 | 2.976229 | 2.847141 |
| C | -3.156316 | 2.201369 | 2.259921 |
| C | -3.226551 | 0.798260 | 2.292813 |
| H | -4.374060 | -0.888839 | 2.984293 |
| H | -6.170923 | 0.489692 | 3.993393 |
| H | -6.031687 | 2.973866 | 3.920702 |
| H | -4.082437 | 4.060285 | 2.815660 |
| H | -2.311144 | 2.684767 | 1.778966 |
| S | -1.942613 | -0.211070 | 1.562927 |
| H | -2.866830 | -1.052957 | 0.536145 |
| H | 6.617170 | 1.752163 | -0.462480 |
| H | 8.830109 | 1.640216 | 0.639469 |
| H | 9.757794 | -0.556004 | 1.356728 |
| C | 3.142247 | -0.533304 | 2.668861 |
| C | -0.344566 | 3.144542 | -0.692024 |
| C | 0.051251 | 4.483127 | -0.572363 |
| C | -1.435791 | 2.802102 | -1.506955 |
| C | -0.658861 | 5.442381 | -1.282592 |
| H | 0.888559 | 4.772383 | 0.046477 |
| C | -2.117072 | 3.811102 | -2.167885 |
| H | -1.713360 | 1.764907 | -1.654712 |
| C | -1.758570 | 5.149457 | -2.084478 |
| H | -2.297994 | 5.923731 | -2.615788 |
| F | -0.283254 | 6.732248 | -1.169296 |
| F | -3.183922 | 3.470454 | -2.938547 |
| C | 3.340737 | 0.725962 | 3.536143 |
| H | 2.488908 | 1.408075 | 3.447955 |
| H | 3.441216 | 0.445034 | 4.591279 |
| H | 4.244191 | 1.274779 | 3.245057 |
| C | 1.921359 | -1.319997 | 3.187124 |
| H | 1.015411 | -0.703846 | 3.216101 |
| H | 1.715840 | -2.197791 | 2.570233 |
| H | 2.114407 | -1.652454 | 4.214282 |
| C | 4.395222 | -1.422152 | 2.777523 |
| H | 4.278190 | -2.356016 | 2.215985 |
| H | 5.290699 | -0.911819 | 2.407824 |

| | | | |
|---|---|---|---|
| H | 4.570478 | -1.688492 | 3.826442 |

TS Conformation 51

B3LYP/6-31G(d) Energy = -3411.889168
M06-2X/def2tzvpp/IEFPCM(chloroform) Energy = -3411.891915
M06-2X/def2tzvpp/IEFPCM(chloroform)//B3LYP/6-31G(d) Quasiharmonic Free Energy = -3411.165504

Frequencies (Top 3 out of 291)

1. -970.7813 cm-1
2. 10.5008 cm-1
3. 11.5446 cm-1

B3LYP/6-31G(d) Molecular Geometry in Cartesian Coordinates

| | | | |
|---|---|---|---|
| C | -2.814958 | -1.234311 | 0.923902 |
| C | -2.812395 | -0.692358 | -0.522664 |
| H | -3.698481 | -0.090942 | -0.715279 |
| C | -1.745293 | 1.581377 | -0.677504 |
| N | -0.529202 | 2.166097 | -0.382307 |
| H | 0.092993 | 1.604393 | 0.193220 |
| N | -1.675864 | 0.225947 | -0.596711 |
| H | -0.758580 | -0.190652 | -0.456215 |
| C | -4.979252 | 0.017973 | 1.468020 |
| C | -3.729727 | -1.201641 | 3.190104 |
| C | -5.498142 | 0.378164 | 2.888968 |
| H | -4.565823 | 0.914439 | 0.995228 |
| C | -4.336708 | 0.046729 | 3.834296 |
| H | -4.290760 | -2.106564 | 3.461811 |
| H | -2.679619 | -1.374724 | 3.426489 |
| H | -5.818896 | 1.422756 | 2.934642 |
| H | -6.367345 | -0.243431 | 3.130440 |
| H | -4.661877 | -0.124119 | 4.865390 |
| H | -3.598296 | 0.857264 | 3.839971 |
| N | -3.858992 | -0.931608 | 1.746656 |
| O | -1.843305 | -1.883064 | 1.335848 |
| S | -3.146589 | 2.442412 | -1.057725 |
| C | -6.077896 | -0.539717 | 0.577778 |
| C | -6.659083 | -1.790268 | 0.832879 |
| C | -6.562723 | 0.223994 | -0.490113 |
| C | -7.700003 | -2.265762 | 0.037767 |
| H | -6.285050 | -2.401533 | 1.650709 |
| C | -7.608640 | -0.250012 | -1.286961 |
| C | -8.179912 | -1.494986 | -1.025367 |
| H | -8.136276 | -3.239484 | 0.244930 |
| O | 1.269950 | -0.672621 | -0.611564 |
| O | 1.253557 | 1.041488 | 2.049482 |
| C | 2.487266 | -0.484888 | -0.579331 |
| C | 3.279796 | -0.386845 | 0.668045 |
| O | 3.255598 | -0.399635 | -1.664871 |
| C | 2.604616 | -0.622209 | -2.931817 |
| H | 3.358618 | -0.388668 | -3.682819 |
| H | 2.300002 | -1.668655 | -3.008327 |
| H | 1.735504 | 0.030788 | -3.036413 |
| S | 2.132587 | -0.164280 | 2.025028 |
| C | 1.176863 | -1.677503 | 2.250698 |
| H | 0.351027 | -1.676611 | 1.537387 |
| H | 1.862362 | -2.520739 | 2.102827 |
| H | 0.788748 | -1.643829 | 3.271617 |
| C | 3.213189 | -0.166305 | 3.468647 |

| | | | |
|---|---|---|---|
| H | 3.786310 | -1.097264 | 3.477493 |
| H | 3.873875 | 0.697641 | 3.393324 |
| H | 2.565774 | -0.086211 | 4.344163 |
| C | 4.419234 | 0.606485 | 0.718151 |
| C | 5.719160 | 0.189187 | 1.040993 |
| C | 4.187758 | 1.967201 | 0.455100 |
| C | 6.758563 | 1.119634 | 1.103743 |
| H | 5.912360 | -0.863751 | 1.224792 |
| C | 5.222872 | 2.896711 | 0.534403 |
| H | 3.192068 | 2.301774 | 0.183082 |
| C | 6.513253 | 2.472408 | 0.860172 |
| H | 7.763178 | 0.783618 | 1.345995 |
| H | 5.010041 | 3.945870 | 0.349346 |
| H | 7.324682 | 3.192824 | 0.920042 |
| C | 5.281634 | -4.407409 | -1.017937 |
| C | 5.131937 | -4.982782 | -2.279829 |
| C | 3.869922 | -5.077977 | -2.870321 |
| C | 2.756127 | -4.597282 | -2.178733 |
| C | 2.901088 | -4.029625 | -0.910787 |
| C | 4.168459 | -3.919818 | -0.312749 |
| H | 6.266165 | -4.330229 | -0.566332 |
| H | 6.008080 | -5.355599 | -2.805201 |
| H | 3.755088 | -5.526825 | -3.853379 |
| H | 1.763843 | -4.677724 | -2.617157 |
| H | 2.022050 | -3.678617 | -0.378089 |
| S | 4.386204 | -3.210693 | 1.314793 |
| H | 3.785198 | -1.682655 | 0.944010 |
| H | -6.108166 | 1.187484 | -0.706931 |
| H | -7.970500 | 0.354361 | -2.114481 |
| H | -8.991096 | -1.866338 | -1.645837 |
| C | -2.759409 | -1.803072 | -1.630454 |
| C | -0.049837 | 3.462223 | -0.634656 |
| C | -0.489002 | 4.258113 | -1.702379 |
| C | 0.993372 | 3.917563 | 0.192026 |
| C | 0.130001 | 5.483466 | -1.907972 |
| H | -1.291063 | 3.943667 | -2.353981 |
| C | 1.589472 | 5.137646 | -0.088582 |
| H | 1.326763 | 3.335088 | 1.043217 |
| C | 1.181137 | 5.959287 | -1.130414 |
| H | 1.652106 | 6.913897 | -1.328613 |
| F | -0.293598 | 6.245319 | -2.936135 |
| F | 2.621721 | 5.540149 | 0.688984 |
| C | -1.449296 | -2.615585 | -1.616175 |
| H | -1.315565 | -3.146030 | -0.670670 |
| H | -1.478465 | -3.354387 | -2.426719 |
| H | -0.569785 | -1.984617 | -1.779317 |
| C | -2.905839 | -1.104970 | -2.997625 |
| H | -3.849916 | -0.553468 | -3.067576 |
| H | -2.090810 | -0.395399 | -3.174128 |
| H | -2.886310 | -1.849583 | -3.802376 |
| C | -3.944677 | -2.766796 | -1.430010 |
| H | -4.905825 | -2.245438 | -1.471210 |
| H | -3.940703 | -3.525047 | -2.221848 |
| H | -3.881513 | -3.293569 | -0.470924 |

TS Conformation 52

B3LYP/6-31G(d) Energy = -3411.889212
M06-2X/def2tzvpp/IEFPCM(chloroform) Energy = -3411.891911
M06-2X/def2tzvpp/IEFPCM(chloroform)//B3LYP/6-31G(d) Quasiharmonic Free Energy = -3411.165358

Frequencies (Top 3 out of 291)

1. -482.0377 cm-1
2. 7.9047 cm-1
3. 10.3363 cm-1

B3LYP/6-31G(d) Molecular Geometry in Cartesian Coordinates

| | | | |
|---|---|---|---|
| C | 3.179260 | -1.331869 | -0.132404 |
| C | 3.311432 | -0.249341 | 0.956280 |
| H | 4.055452 | 0.493406 | 0.673646 |
| C | 1.808284 | 1.754154 | 0.652790 |
| N | 0.465824 | 2.040045 | 0.623334 |
| H | -0.139835 | 1.241453 | 0.789622 |
| N | 2.026117 | 0.443400 | 0.978712 |
| H | 1.214840 | -0.166385 | 1.040715 |
| C | 4.801441 | -0.108268 | -1.668450 |
| C | 3.716116 | -2.216051 | -2.340527 |
| C | 4.758288 | -0.233345 | -3.213140 |
| H | 4.330500 | 0.829588 | -1.362293 |
| C | 4.649657 | -1.744219 | -3.461621 |
| H | 3.913315 | -3.235623 | -1.996372 |
| H | 2.663273 | -2.172841 | -2.645570 |
| H | 5.632660 | 0.224956 | -3.681560 |
| H | 3.863795 | 0.277548 | -3.588912 |
| H | 5.633013 | -2.217569 | -3.370765 |
| H | 4.254503 | -1.986412 | -4.452223 |
| N | 3.945848 | -1.249910 | -1.245474 |
| O | 2.332414 | -2.237536 | -0.011760 |
| S | 3.057387 | 2.849389 | 0.351581 |
| C | 6.217231 | -0.144125 | -1.109503 |
| C | 6.919356 | -1.338431 | -0.899670 |
| C | 6.863235 | 1.070912 | -0.842640 |
| C | 8.240192 | -1.317631 | -0.448866 |
| H | 6.426710 | -2.292142 | -1.066084 |
| C | 8.183416 | 1.093468 | -0.391081 |
| C | 8.877747 | -0.101659 | -0.196222 |
| H | 8.769017 | -2.253699 | -0.289445 |
| O | -2.750423 | -4.475056 | -0.313090 |
| O | -0.788135 | -0.763586 | 0.838610 |
| C | -3.490055 | -3.572484 | 0.067793 |
| C | -3.058396 | -2.168308 | 0.293310 |
| O | -4.813003 | -3.709821 | 0.217362 |
| C | -5.357585 | -4.985293 | -0.176179 |
| H | -6.427317 | -4.909218 | 0.015803 |
| H | -5.166905 | -5.163716 | -1.236780 |
| H | -4.913521 | -5.790213 | 0.414546 |
| S | -1.239497 | -2.094767 | 0.347503 |
| C | -0.497381 | -3.375740 | 1.384438 |
| H | -0.804352 | -3.157647 | 2.409593 |
| H | -0.864641 | -4.344258 | 1.046156 |
| H | 0.582970 | -3.262014 | 1.259087 |
| C | -0.619789 | -2.431572 | -1.307464 |
| H | -1.022162 | -3.396781 | -1.618133 |
| H | -1.000810 | -1.623724 | -1.940461 |
| H | 0.471017 | -2.430149 | -1.222921 |
| C | -3.665354 | -1.343935 | 1.391987 |
| C | -3.913446 | 0.018135 | 1.166991 |
| C | -3.974657 | -1.895829 | 2.645726 |
| C | -4.457879 | 0.813865 | 2.175920 |
| H | -3.697260 | 0.444524 | 0.192935 |
| C | -4.525887 | -1.102426 | 3.650086 |

| | | | |
|---|---|---|---|
| H | -3.804038 | -2.954015 | 2.828766 |
| C | -4.762387 | 0.255069 | 3.418335 |
| H | -4.635792 | 1.868088 | 1.985708 |
| H | -4.768685 | -1.542649 | 4.613405 |
| H | -5.188385 | 0.873738 | 4.203533 |
| C | -4.910206 | 1.359012 | -2.976619 |
| C | -6.024073 | 2.194710 | -2.893098 |
| C | -7.173131 | 1.780667 | -2.215856 |
| C | -7.194647 | 0.514194 | -1.627050 |
| C | -6.087050 | -0.328687 | -1.716925 |
| C | -4.920612 | 0.079681 | -2.391146 |
| H | -4.017261 | 1.690746 | -3.498571 |
| H | -5.990127 | 3.178511 | -3.356172 |
| H | -8.038819 | 2.434452 | -2.148420 |
| H | -8.082480 | 0.175692 | -1.096968 |
| H | -6.117321 | -1.315450 | -1.262980 |
| S | -3.503895 | -0.990278 | -2.538048 |
| H | -3.293776 | -1.610570 | -0.867129 |
| H | 6.322567 | 2.004415 | -0.979515 |
| H | 8.665097 | 2.045202 | -0.183727 |
| H | 9.904693 | -0.086334 | 0.158456 |
| C | 3.729863 | -0.792227 | 2.369661 |
| C | -0.264434 | 3.239570 | 0.508918 |
| C | 0.243600 | 4.456204 | 0.035572 |
| C | -1.616936 | 3.147616 | 0.888476 |
| C | -0.620354 | 5.543034 | -0.034583 |
| H | 1.274425 | 4.561477 | -0.266128 |
| C | -2.424053 | 4.267457 | 0.787135 |
| H | -2.034305 | 2.220666 | 1.266295 |
| C | -1.960278 | 5.494706 | 0.329235 |
| H | -2.605213 | 6.361517 | 0.258135 |
| F | -0.127798 | 6.711920 | -0.490929 |
| F | -3.719128 | 4.164146 | 1.161725 |
| C | 2.657543 | -1.691193 | 3.013451 |
| H | 2.516414 | -2.614763 | 2.446977 |
| H | 2.968780 | -1.958823 | 4.030228 |
| H | 1.692533 | -1.177228 | 3.094160 |
| C | 3.973699 | 0.426408 | 3.282623 |
| H | 4.759200 | 1.076485 | 2.882982 |
| H | 3.067907 | 1.030450 | 3.395472 |
| H | 4.283207 | 0.090579 | 4.279492 |
| C | 5.039795 | -1.590766 | 2.233872 |
| H | 4.908557 | -2.483988 | 1.611704 |
| H | 5.839911 | -0.985821 | 1.795477 |
| H | 5.376340 | -1.925079 | 3.222210 |

TS Conformation 53

B3LYP/6-31G(d) Energy = -3411.886833
M06-2X/def2tzvpp/IEFPCM(chloroform) Energy = -3411.891947
M06-2X/def2tzvpp/IEFPCM(chloroform)//B3LYP/6-31G(d) Quasiharmonic Free Energy = -3411.165321

Frequencies (Top 3 out of 291)

1. -750.7109 cm-1
2. 6.5300 cm-1
3. 12.8893 cm-1

B3LYP/6-31G(d) Molecular Geometry in Cartesian Coordinates

| | | | |
|---|---|---|---|
| C | -3.367006 | -1.068859 | -0.322619 |

| | | | |
|---|---|---|---|
| C | -3.453678 | -0.074893 | -1.497151 |
| H | -4.251364 | 0.645833 | -1.323613 |
| C | -2.094729 | 1.954395 | -0.967676 |
| N | -0.786989 | 2.363040 | -0.898130 |
| H | -0.083715 | 1.635394 | -1.010768 |
| N | -2.218633 | 0.700375 | -1.494163 |
| H | -1.362440 | 0.176059 | -1.658207 |
| C | -4.242125 | -1.934838 | 1.795974 |
| C | -5.562660 | -0.210932 | 0.639617 |
| C | -5.688056 | -1.908472 | 2.346464 |
| H | -3.965138 | -2.933976 | 1.446845 |
| C | -6.164939 | -0.484904 | 2.025203 |
| H | -5.323685 | 0.844341 | 0.481615 |
| H | -6.243387 | -0.540805 | -0.154873 |
| H | -5.728036 | -2.150750 | 3.411528 |
| H | -6.296312 | -2.644175 | 1.805998 |
| H | -5.763048 | 0.223478 | 2.757504 |
| H | -7.254007 | -0.384876 | 2.025370 |
| N | -4.332865 | -1.036904 | 0.628334 |
| O | -2.403240 | -1.845782 | -0.234147 |
| S | -3.414817 | 2.880185 | -0.449704 |
| C | -3.207970 | -1.487662 | 2.826551 |
| C | -2.762742 | -0.163945 | 2.924923 |
| C | -2.717143 | -2.431153 | 3.740629 |
| C | -1.853656 | 0.208505 | 3.919101 |
| H | -3.108359 | 0.581343 | 2.213974 |
| C | -1.812301 | -2.062143 | 4.736121 |
| C | -1.377077 | -0.736753 | 4.829690 |
| H | -1.519197 | 1.240707 | 3.976884 |
| O | 0.622306 | -0.215177 | -1.871202 |
| O | 1.519386 | 0.433782 | 0.820203 |
| C | 1.485811 | -1.085978 | -1.940881 |
| C | 2.254531 | -1.693614 | -0.818467 |
| O | 1.943312 | -1.575314 | -3.097653 |
| C | 1.448721 | -0.966907 | -4.304868 |
| H | 2.067668 | -1.376583 | -5.102393 |
| H | 0.399727 | -1.229850 | -4.462822 |
| H | 1.553329 | 0.118718 | -4.253786 |
| S | 1.713817 | -1.030057 | 0.769416 |
| C | 0.249776 | -1.881918 | 1.395864 |
| H | 0.455712 | -2.952285 | 1.443295 |
| H | 0.047940 | -1.467697 | 2.387321 |
| H | -0.587333 | -1.680347 | 0.719591 |
| C | 3.010806 | -1.534307 | 1.918251 |
| H | 2.688562 | -1.194659 | 2.905133 |
| H | 3.132613 | -2.617775 | 1.886251 |
| H | 3.927875 | -1.022338 | 1.613008 |
| C | 2.345267 | -3.207028 | -0.809622 |
| C | 3.572285 | -3.846925 | -0.577684 |
| C | 1.201864 | -3.991582 | -1.047782 |
| C | 3.648173 | -5.241309 | -0.571854 |
| H | 4.467082 | -3.243902 | -0.445242 |
| C | 1.283509 | -5.382766 | -1.044174 |
| H | 0.244764 | -3.510715 | -1.238534 |
| C | 2.507639 | -6.011373 | -0.800983 |
| H | 4.606348 | -5.723796 | -0.399413 |
| H | 0.392285 | -5.974898 | -1.233109 |
| H | 2.571392 | -7.096206 | -0.799082 |
| C | 4.746198 | 1.503842 | 0.232258 |
| C | 4.978095 | 2.326046 | 1.335708 |
| C | 5.935863 | 1.973897 | 2.290084 |
| C | 6.657493 | 0.788990 | 2.127247 |

| | | | |
|---|---|---|---|
| C | 6.418647 | -0.040302 | 1.029463 |
| C | 5.455406 | 0.299058 | 0.057257 |
| H | 4.012028 | 1.794482 | -0.513257 |
| H | 4.407756 | 3.244418 | 1.441863 |
| H | 6.121114 | 2.617821 | 3.145957 |
| H | 7.412732 | 0.506248 | 2.857713 |
| H | 6.985197 | -0.959274 | 0.905447 |
| S | 5.158292 | -0.764324 | -1.342126 |
| H | 3.497975 | -1.215535 | -0.984074 |
| H | -3.047768 | -3.465850 | 3.671260 |
| H | -1.446987 | -2.808066 | 5.437543 |
| H | -0.676156 | -0.443946 | 5.607032 |
| C | -3.731759 | -0.753417 | -2.881736 |
| C | -0.266136 | 3.632360 | -0.558207 |
| C | 0.940265 | 3.662795 | 0.159039 |
| C | -0.867634 | 4.826312 | -0.977623 |
| C | 1.506414 | 4.895998 | 0.449163 |
| H | 1.415132 | 2.750359 | 0.500741 |
| C | -0.254184 | 6.025552 | -0.644924 |
| H | -1.790428 | 4.830798 | -1.539235 |
| C | 0.937179 | 6.103638 | 0.067293 |
| H | 1.395929 | 7.053813 | 0.311053 |
| F | 2.662060 | 4.931165 | 1.151517 |
| F | -0.833187 | 7.174856 | -1.048588 |
| C | -3.910275 | 0.362468 | -3.930189 |
| H | -3.004796 | 0.970216 | -4.022039 |
| H | -4.129020 | -0.073109 | -4.912397 |
| H | -4.736350 | 1.032456 | -3.665320 |
| C | -5.039929 | -1.562994 | -2.781180 |
| H | -5.279750 | -2.012104 | -3.751676 |
| H | -4.962443 | -2.378488 | -2.052414 |
| H | -5.886176 | -0.925725 | -2.497158 |
| C | -2.593080 | -1.691271 | -3.325622 |
| H | -2.441222 | -2.507308 | -2.614894 |
| H | -2.831420 | -2.121363 | -4.305986 |
| H | -1.647048 | -1.148460 | -3.426214 |

TS Conformation 54

B3LYP/6-31G(d) Energy = -3411.889725
M06-2X/def2tzvpp/IEFPCM(chloroform) Energy = -3411.891501
M06-2X/def2tzvpp/IEFPCM(chloroform)//B3LYP/6-31G(d) Quasiharmonic Free Energy = -3411.16532

Frequencies (Top 3 out of 291)

1. -975.1880 cm-1
2. 10.9439 cm-1
3. 12.5966 cm-1

B3LYP/6-31G(d) Molecular Geometry in Cartesian Coordinates

| | | | |
|---|---|---|---|
| C | 2.984118 | -1.623374 | -0.476917 |
| C | 3.021850 | -0.814237 | 0.836549 |
| H | 3.947640 | -0.246787 | 0.914690 |
| C | 2.116799 | 1.512969 | 0.552480 |
| N | 0.938884 | 2.117484 | 0.158342 |
| H | 0.273398 | 1.505298 | -0.307647 |
| N | 1.950273 | 0.175501 | 0.735003 |
| H | 1.003737 | -0.193062 | 0.686258 |
| C | 5.197100 | -0.630755 | -1.273767 |
| C | 3.919289 | -2.231859 | -2.638536 |

| | | | |
|---|---|---|---|
| C | 5.525206 | -0.445670 | -2.777851 |
| H | 4.875081 | 0.324224 | -0.850093 |
| C | 5.180235 | -1.805549 | -3.400898 |
| H | 3.826358 | -3.314661 | -2.509978 |
| H | 3.001230 | -1.885880 | -3.129012 |
| H | 4.876492 | 0.336427 | -3.190275 |
| H | 6.563755 | -0.143248 | -2.934252 |
| H | 5.994083 | -2.518609 | -3.230717 |
| H | 5.008586 | -1.749789 | -4.479882 |
| N | 4.044682 | -1.564886 | -1.325807 |
| O | 1.979445 | -2.283932 | -0.776137 |
| S | 3.584980 | 2.321426 | 0.750790 |
| C | 6.386030 | -1.141058 | -0.470139 |
| C | 7.213879 | -0.207838 | 0.169367 |
| C | 6.715868 | -2.499854 | -0.384676 |
| C | 8.351104 | -0.619883 | 0.864871 |
| H | 6.957806 | 0.848441 | 0.129297 |
| C | 7.853685 | -2.914132 | 0.309940 |
| C | 8.676966 | -1.975579 | 0.934220 |
| H | 8.976833 | 0.118237 | 1.359564 |
| O | -1.066639 | -0.517550 | 0.979993 |
| O | -0.912538 | 0.610779 | -1.953528 |
| C | -2.273390 | -0.292449 | 0.898919 |
| C | -3.056890 | -0.352471 | -0.355304 |
| O | -3.044271 | -0.027494 | 1.959661 |
| C | -2.378999 | -0.009355 | 3.239114 |
| H | -3.150784 | 0.267165 | 3.956522 |
| H | -1.974293 | -0.997535 | 3.469474 |
| H | -1.570823 | 0.725591 | 3.236912 |
| S | -1.886574 | -0.493317 | -1.710223 |
| C | -1.048292 | -2.090197 | -1.584602 |
| H | -1.743233 | -2.814498 | -1.149619 |
| H | -0.762527 | -2.373081 | -2.600314 |
| H | -0.155864 | -1.961956 | -0.968802 |
| C | -2.956549 | -0.702758 | -3.144721 |
| H | -3.517948 | 0.222797 | -3.276131 |
| H | -2.303008 | -0.886212 | -3.999917 |
| H | -3.628544 | -1.545448 | -2.948285 |
| C | -4.085522 | 0.724895 | -0.611844 |
| C | -3.728650 | 2.081011 | -0.522828 |
| C | -5.401224 | 0.391718 | -0.967377 |
| C | -4.655117 | 3.084022 | -0.801010 |
| H | -2.718381 | 2.354915 | -0.236027 |
| C | -6.333559 | 1.397968 | -1.227513 |
| H | -5.695376 | -0.650540 | -1.029061 |
| C | -5.962676 | 2.741860 | -1.153763 |
| H | -4.344418 | 4.123869 | -0.755369 |
| H | -7.352262 | 1.126817 | -1.490356 |
| H | -6.689915 | 3.520191 | -1.368774 |
| C | -6.894000 | -3.947861 | -0.095648 |
| C | -8.065345 | -3.969408 | 0.660018 |
| C | -8.187255 | -3.175332 | 1.802713 |
| C | -7.117495 | -2.363334 | 2.182519 |
| C | -5.937379 | -2.345853 | 1.437432 |
| C | -5.808936 | -3.136982 | 0.282425 |
| H | -6.809632 | -4.562519 | -0.987312 |
| H | -8.889556 | -4.607547 | 0.349675 |
| H | -9.102467 | -3.190135 | 2.388425 |
| H | -7.195906 | -1.739725 | 3.070246 |
| H | -5.111489 | -1.715850 | 1.753579 |
| S | -4.318665 | -3.168579 | -0.697911 |
| H | -3.671562 | -1.618376 | -0.417670 |

| | | | |
|---|---|---|---|
| H | 6.067980 | -3.241916 | -0.842261 |
| H | 8.092517 | -3.972894 | 0.368254 |
| H | 9.560128 | -2.299039 | 1.478469 |
| C | 2.916524 | -1.689742 | 2.134360 |
| C | 0.559423 | 3.469764 | 0.163390 |
| C | 1.080538 | 4.417694 | 1.055919 |
| C | -0.468339 | 3.837960 | -0.723917 |
| C | 0.558073 | 5.703377 | 1.030295 |
| H | 1.873289 | 4.172348 | 1.747355 |
| C | -0.965799 | 5.131538 | -0.675632 |
| H | -0.867045 | 3.131565 | -1.443103 |
| C | -0.473584 | 6.102122 | 0.186258 |
| H | -0.868367 | 7.110217 | 0.202604 |
| F | 1.060733 | 6.611339 | 1.890324 |
| F | -1.984805 | 5.457575 | -1.504598 |
| C | 4.053538 | -2.729272 | 2.120237 |
| H | 5.037240 | -2.256370 | 2.032317 |
| H | 4.040525 | -3.305458 | 3.052711 |
| H | 3.941951 | -3.440189 | 1.293515 |
| C | 1.568204 | -2.424016 | 2.268559 |
| H | 0.722733 | -1.729689 | 2.309515 |
| H | 1.399944 | -3.112849 | 1.437769 |
| H | 1.564458 | -3.000478 | 3.201836 |
| C | 3.106268 | -0.754044 | 3.345338 |
| H | 2.327353 | 0.014554 | 3.382622 |
| H | 3.056284 | -1.331689 | 4.276023 |
| H | 4.075756 | -0.244656 | 3.312658 |

TS Conformation 55

B3LYP/6-31G(d) Energy = -3411.891588
M06-2X/def2tzvpp/IEFPCM(chloroform) Energy = -3411.89148
M06-2X/def2tzvpp/IEFPCM(chloroform)//B3LYP/6-31G(d) Quasiharmonic Free Energy = -3411.165162

Frequencies (Top 3 out of 291)

1. -889.9470 cm-1
2. 6.7991 cm-1
3. 9.2228 cm-1

B3LYP/6-31G(d) Molecular Geometry in Cartesian Coordinates

| | | | |
|---|---|---|---|
| C | 2.793863 | -1.527263 | 0.437708 |
| C | 3.221749 | -0.177839 | 1.046456 |
| H | 4.085142 | 0.226939 | 0.521267 |
| C | 2.157832 | 1.855774 | -0.001272 |
| N | 0.905505 | 2.394939 | -0.162751 |
| H | 0.156814 | 1.855163 | 0.261860 |
| N | 2.114152 | 0.737434 | 0.785387 |
| H | 1.197487 | 0.380354 | 1.043402 |
| C | 4.529403 | -1.338228 | -1.417448 |
| C | 2.983448 | -3.251529 | -1.275708 |
| C | 4.356281 | -2.005500 | -2.806130 |
| H | 4.288431 | -0.274253 | -1.488541 |
| C | 3.916482 | -3.439142 | -2.478004 |
| H | 2.986004 | -4.091703 | -0.574987 |
| H | 1.944511 | -3.092605 | -1.589986 |
| H | 5.273437 | -1.954117 | -3.397923 |
| H | 3.565354 | -1.483642 | -3.357897 |
| H | 4.782600 | -4.050694 | -2.203633 |
| H | 3.412749 | -3.932770 | -3.313928 |

| | | | |
|---|---|---|---|
| N | 3.484410 | -2.028218 | -0.614440 |
| O | 1.787724 | -2.117170 | 0.873704 |
| S | 3.588874 | 2.469045 | -0.654589 |
| C | 5.940775 | -1.479060 | -0.863760 |
| C | 6.386121 | -2.635384 | -0.209711 |
| C | 6.846864 | -0.428131 | -1.062300 |
| C | 7.709222 | -2.744326 | 0.221391 |
| H | 5.691669 | -3.447342 | -0.013825 |
| C | 8.169726 | -0.535031 | -0.631211 |
| C | 8.606289 | -1.695968 | 0.009189 |
| H | 8.036965 | -3.647721 | 0.729106 |
| O | -3.694785 | -3.099032 | 1.294298 |
| O | -0.894548 | 0.202521 | 0.988554 |
| C | -4.185933 | -1.972781 | 1.315904 |
| C | -3.441247 | -0.726254 | 1.016055 |
| O | -5.484188 | -1.736447 | 1.551842 |
| C | -6.319811 | -2.904218 | 1.673566 |
| H | -7.297460 | -2.524581 | 1.970511 |
| H | -6.382495 | -3.414473 | 0.709472 |
| H | -5.921670 | -3.585832 | 2.428493 |
| S | -1.664041 | -1.070485 | 1.071999 |
| C | -1.152296 | -1.959749 | 2.560857 |
| H | -0.086487 | -2.165473 | 2.429687 |
| H | -1.326307 | -1.283675 | 3.400600 |
| H | -1.751289 | -2.867328 | 2.636834 |
| C | -1.246292 | -2.180686 | -0.279424 |
| H | -1.821962 | -3.095862 | -0.137151 |
| H | -1.559389 | -1.663739 | -1.194193 |
| H | -0.165985 | -2.337968 | -0.208009 |
| C | -3.773265 | 0.545852 | 1.747696 |
| C | -3.769053 | 1.765977 | 1.055253 |
| C | -4.071370 | 0.548428 | 3.120770 |
| C | -4.048180 | 2.961283 | 1.719944 |
| H | -3.572502 | 1.772840 | -0.011737 |
| C | -4.359499 | 1.740561 | 3.782544 |
| H | -4.099521 | -0.389972 | 3.669235 |
| C | -4.341393 | 2.950682 | 3.084364 |
| H | -4.025486 | 3.895656 | 1.166665 |
| H | -4.595171 | 1.725334 | 4.843242 |
| H | -4.559953 | 3.880625 | 3.602277 |
| C | -4.460350 | -2.546182 | -3.330935 |
| C | -5.355086 | -3.566165 | -3.655445 |
| C | -6.621194 | -3.611686 | -3.068161 |
| C | -6.983795 | -2.616359 | -2.157432 |
| C | -6.093825 | -1.589527 | -1.836337 |
| C | -4.811370 | -1.539206 | -2.413623 |
| H | -3.478028 | -2.516438 | -3.794133 |
| H | -5.059772 | -4.330213 | -4.371178 |
| H | -7.318204 | -4.405833 | -3.322557 |
| H | -7.973803 | -2.627131 | -1.705879 |
| H | -6.391649 | -0.808899 | -1.142526 |
| S | -3.671416 | -0.220567 | -2.032241 |
| H | -3.589901 | -0.496686 | -0.332163 |
| H | 6.508517 | 0.484002 | -1.548158 |
| H | 8.855641 | 0.292978 | -0.788531 |
| H | 9.635093 | -1.779805 | 0.348607 |
| C | 3.607880 | -0.241903 | 2.567715 |
| C | 0.434113 | 3.599790 | -0.718820 |
| C | 1.165455 | 4.448471 | -1.559960 |
| C | -0.896259 | 3.921549 | -0.388316 |
| C | 0.540122 | 5.594846 | -2.036832 |
| H | 2.186133 | 4.231670 | -1.835656 |

| | | | |
|---|---|---|---|
| C | -1.459524 | 5.076014 | -0.902865 |
| H | -1.481952 | 3.287949 | 0.268620 |
| C | -0.769337 | 5.948299 | -1.735676 |
| H | -1.224919 | 6.848524 | -2.128574 |
| F | 1.247268 | 6.408629 | -2.846102 |
| F | -2.736925 | 5.372745 | -0.570345 |
| C | 2.414474 | -0.583540 | 3.479506 |
| H | 2.043276 | -1.593798 | 3.291686 |
| H | 2.728169 | -0.524136 | 4.528540 |
| H | 1.588426 | 0.124980 | 3.347000 |
| C | 4.161147 | 1.140973 | 2.966949 |
| H | 5.038247 | 1.410364 | 2.369127 |
| H | 3.411913 | 1.927140 | 2.830383 |
| H | 4.456698 | 1.134499 | 4.022884 |
| C | 4.709150 | -1.300884 | 2.762312 |
| H | 4.353114 | -2.307726 | 2.514043 |
| H | 5.588503 | -1.089549 | 2.145425 |
| H | 5.031251 | -1.315112 | 3.810129 |

TS Conformation 56

B3LYP/6-31G(d) Energy = -3411.889688
M06-2X/def2tzvpp/IEFPCM(chloroform) Energy = -3411.891341
M06-2X/def2tzvpp/IEFPCM(chloroform)//B3LYP/6-31G(d) Quasiharmonic Free Energy = -3411.165038

Frequencies (Top 3 out of 291)

1. -828.6552 cm-1
2. 6.3894 cm-1
3. 9.7435 cm-1

B3LYP/6-31G(d) Molecular Geometry in Cartesian Coordinates

| | | | |
|---|---|---|---|
| C | 3.124477 | -1.315807 | -0.134344 |
| C | 3.224308 | -0.261525 | 0.984958 |
| H | 3.954445 | 0.504504 | 0.729727 |
| C | 1.683055 | 1.718416 | 0.725306 |
| N | 0.335261 | 1.976816 | 0.686225 |
| H | -0.253270 | 1.155780 | 0.795542 |
| N | 1.923727 | 0.402850 | 1.014655 |
| H | 1.125151 | -0.226431 | 1.036979 |
| C | 4.738395 | -0.019980 | -1.619475 |
| C | 3.698827 | -2.127544 | -2.360532 |
| C | 4.710774 | -0.101700 | -3.167419 |
| H | 4.247877 | 0.900224 | -1.291301 |
| C | 4.632634 | -1.606662 | -3.459509 |
| H | 3.912738 | -3.152511 | -2.043447 |
| H | 2.647880 | -2.095824 | -2.673369 |
| H | 5.580268 | 0.385950 | -3.614841 |
| H | 3.809985 | 0.402952 | -3.536539 |
| H | 5.623980 | -2.063998 | -3.373442 |
| H | 4.250387 | -1.827819 | -4.460049 |
| N | 3.900651 | -1.188439 | -1.236819 |
| O | 2.294629 | -2.240186 | -0.047229 |
| S | 2.914310 | 2.845007 | 0.468894 |
| C | 6.149915 | -0.046019 | -1.049132 |
| C | 6.871452 | -1.233044 | -0.865604 |
| C | 6.771929 | 1.172419 | -0.743601 |
| C | 8.187808 | -1.201355 | -0.402506 |
| H | 6.397312 | -2.190331 | -1.062380 |
| C | 8.087579 | 1.205871 | -0.279672 |

| | | | |
|---|---|---|---|
| C | 8.801402 | 0.018254 | -0.111077 |
| H | 8.731839 | -2.131998 | -0.263844 |
| O | -2.722343 | -4.614541 | -0.435525 |
| O | -0.886281 | -0.841144 | 0.713692 |
| C | -3.487966 | -3.741961 | -0.032463 |
| C | -3.105357 | -2.326613 | 0.194271 |
| O | -4.799323 | -3.936648 | 0.152255 |
| C | -5.299636 | -5.230570 | -0.234104 |
| H | -6.366349 | -5.200854 | -0.013635 |
| H | -5.130817 | -5.396841 | -1.300564 |
| H | -4.807392 | -6.020471 | 0.338800 |
| S | -1.297381 | -2.195570 | 0.249410 |
| C | -0.524077 | -3.432757 | 1.317789 |
| H | 0.553793 | -3.296841 | 1.194444 |
| H | -0.841112 | -3.201183 | 2.336879 |
| H | -0.865364 | -4.417572 | 0.999010 |
| C | -0.649620 | -2.550260 | -1.390118 |
| H | -0.968699 | -3.559367 | -1.654057 |
| H | -1.104553 | -1.810951 | -2.059356 |
| H | 0.436918 | -2.456810 | -1.305225 |
| C | -3.741998 | -1.533415 | 1.299822 |
| C | -4.054408 | -0.182196 | 1.090125 |
| C | -4.017646 | -2.106194 | 2.552840 |
| C | -4.624669 | 0.581386 | 2.109803 |
| H | -3.869067 | 0.267542 | 0.120813 |
| C | -4.595725 | -1.346213 | 3.567905 |
| H | -3.799430 | -3.156960 | 2.727197 |
| C | -4.894476 | 0.001289 | 3.350060 |
| H | -4.848185 | 1.628211 | 1.926431 |
| H | -4.810529 | -1.804915 | 4.529332 |
| H | -5.340802 | 0.594183 | 4.143859 |
| C | -5.945001 | 0.314391 | -2.377111 |
| C | -6.648837 | 1.509685 | -2.233048 |
| C | -5.969088 | 2.728081 | -2.160105 |
| C | -4.575415 | 2.737127 | -2.239104 |
| C | -3.868228 | 1.542127 | -2.387868 |
| C | -4.540203 | 0.307864 | -2.452229 |
| H | -6.477648 | -0.630291 | -2.434950 |
| H | -7.734981 | 1.488642 | -2.176822 |
| H | -6.518803 | 3.658594 | -2.045375 |
| H | -4.032586 | 3.677884 | -2.189476 |
| H | -2.784359 | 1.556896 | -2.459415 |
| S | -3.647316 | -1.226461 | -2.645299 |
| H | -3.378841 | -1.753708 | -1.004738 |
| H | 6.216023 | 2.099728 | -0.860191 |
| H | 8.550451 | 2.159909 | -0.042218 |
| H | 9.824829 | 0.041927 | 0.253190 |
| C | 3.642622 | -0.834162 | 2.386611 |
| C | -0.425659 | 3.157268 | 0.587061 |
| C | 0.080708 | 4.433307 | 0.305594 |
| C | -1.808791 | 2.984284 | 0.787853 |
| C | -0.816208 | 5.493161 | 0.238538 |
| H | 1.134240 | 4.602532 | 0.144186 |
| C | -2.646399 | 4.082713 | 0.703762 |
| H | -2.228270 | 2.010406 | 1.014441 |
| C | -2.186449 | 5.365366 | 0.429194 |
| H | -2.855471 | 6.214632 | 0.369267 |
| F | -0.324387 | 6.718768 | -0.032357 |
| F | -3.970646 | 3.899446 | 0.901766 |
| C | 2.584439 | -1.772640 | 2.996087 |
| H | 2.466190 | -2.682301 | 2.402613 |
| H | 2.893507 | -2.062833 | 4.007320 |

| | | | |
|---|---|---|---|
| H | 1.608849 | -1.280196 | 3.083825 |
| C | 3.853186 | 0.363611 | 3.334765 |
| H | 4.628860 | 1.040099 | 2.960603 |
| H | 2.934227 | 0.945898 | 3.455349 |
| H | 4.160391 | 0.006843 | 4.325054 |
| C | 4.970235 | -1.601025 | 2.240452 |
| H | 4.863063 | -2.479152 | 1.592736 |
| H | 5.761021 | -0.967548 | 1.826076 |
| H | 5.305534 | -1.955500 | 3.222168 |

TS Conformation 57

B3LYP/6-31G(d) Energy = -3411.889688
M06-2X/def2tzvpp/IEFPCM(chloroform) Energy = -3411.891342
M06-2X/def2tzvpp/IEFPCM(chloroform)//B3LYP/6-31G(d) Quasiharmonic Free Energy = -3411.165033

Frequencies (Top 3 out of 291)

1. -828.8279 cm-1
2. 6.4078 cm-1
3. 9.7726 cm-1

B3LYP/6-31G(d) Molecular Geometry in Cartesian Coordinates

| | | | |
|---|---|---|---|
| C | 3.124510 | -1.315730 | -0.134321 |
| C | 3.224310 | -0.261470 | 0.984999 |
| H | 3.954430 | 0.504580 | 0.729779 |
| C | 1.683000 | 1.718430 | 0.725289 |
| N | 0.335190 | 1.976780 | 0.686239 |
| H | -0.253310 | 1.155720 | 0.795519 |
| N | 1.923720 | 0.402890 | 1.014729 |
| H | 1.125140 | -0.226380 | 1.037279 |
| C | 4.738390 | -0.019820 | -1.619431 |
| C | 3.698870 | -2.127390 | -2.360541 |
| C | 4.710770 | -0.101500 | -3.167371 |
| H | 4.247860 | 0.900360 | -1.291231 |
| C | 4.632660 | -1.606460 | -3.459511 |
| H | 3.912821 | -3.152360 | -2.043471 |
| H | 2.647930 | -2.095710 | -2.673371 |
| H | 5.580250 | 0.386180 | -3.614791 |
| H | 3.809960 | 0.403140 | -3.536481 |
| H | 5.624020 | -2.063770 | -3.373461 |
| H | 4.250410 | -1.827600 | -4.460051 |
| N | 3.900670 | -1.188300 | -1.236801 |
| O | 2.294710 | -2.240150 | -0.047221 |
| S | 2.914190 | 2.845050 | 0.468749 |
| C | 6.149910 | -0.045849 | -1.049091 |
| C | 6.871470 | -1.232869 | -0.865601 |
| C | 6.771900 | 1.172591 | -0.743511 |
| C | 8.187830 | -1.201169 | -0.402501 |
| H | 6.397350 | -2.190159 | -1.062421 |
| C | 8.087540 | 1.206051 | -0.279571 |
| C | 8.801390 | 0.018441 | -0.111021 |
| H | 8.731880 | -2.131809 | -0.263871 |
| O | -2.722159 | -4.614540 | -0.435901 |
| O | -0.886150 | -0.841220 | 0.713609 |
| C | -3.487789 | -3.742010 | -0.032751 |
| C | -3.105200 | -2.326680 | 0.194129 |
| O | -4.799149 | -3.936730 | 0.151949 |
| C | -5.299449 | -5.230621 | -0.234511 |
| H | -4.807189 | -6.020570 | 0.338329 |

| | | | |
|---|---|---|---|
| H | -6.366159 | -5.200941 | -0.014041 |
| H | -5.130619 | -5.396811 | -1.300991 |
| S | -1.297230 | -2.195620 | 0.249219 |
| C | -0.523829 | -3.432850 | 1.317469 |
| H | 0.554031 | -3.296730 | 1.194189 |
| H | -0.840959 | -3.201470 | 2.336569 |
| H | -0.864949 | -4.417680 | 0.998529 |
| C | -0.649520 | -2.550140 | -1.390361 |
| H | -1.104270 | -1.810600 | -2.059461 |
| H | 0.437040 | -2.456940 | -1.305441 |
| H | -0.968809 | -3.559130 | -1.654501 |
| C | -3.741810 | -1.533630 | 1.299789 |
| C | -4.017410 | -2.106560 | 2.552749 |
| C | -4.054270 | -0.182390 | 1.090259 |
| C | -4.595480 | -1.346710 | 3.567929 |
| H | -3.799159 | -3.157340 | 2.726979 |
| C | -4.624530 | 0.581060 | 2.110039 |
| H | -3.868980 | 0.267460 | 0.120999 |
| C | -4.894280 | 0.000810 | 3.350239 |
| H | -4.810230 | -1.805530 | 4.529309 |
| H | -4.848090 | 1.627890 | 1.926789 |
| H | -5.340600 | 0.593599 | 4.144119 |
| C | -3.868510 | 1.542450 | -2.387431 |
| C | -4.575910 | 2.737300 | -2.238511 |
| C | -5.969590 | 2.728009 | -2.159581 |
| C | -6.649120 | 1.509499 | -2.232741 |
| C | -5.945080 | 0.314349 | -2.376941 |
| C | -4.540280 | 0.308080 | -2.452001 |
| H | -2.784650 | 1.557410 | -2.458921 |
| H | -4.033240 | 3.678150 | -2.188711 |
| H | -6.519470 | 3.658409 | -2.044741 |
| H | -7.735270 | 1.488269 | -2.176571 |
| H | -6.477560 | -0.630411 | -2.434951 |
| S | -3.647090 | -1.226050 | -2.645271 |
| H | -3.378710 | -1.753590 | -1.004831 |
| H | 6.215970 | 2.099891 | -0.860061 |
| H | 8.550390 | 2.160091 | -0.042081 |
| H | 9.824820 | 0.042121 | 0.253249 |
| C | 3.642640 | -0.834130 | 2.386639 |
| C | -0.425780 | 3.157180 | 0.587009 |
| C | 0.080490 | 4.433230 | 0.305409 |
| C | -1.808890 | 2.984140 | 0.787949 |
| C | -0.816490 | 5.493030 | 0.238369 |
| H | 1.134000 | 4.602500 | 0.143909 |
| C | -2.646570 | 4.082520 | 0.703879 |
| H | -2.228290 | 2.010260 | 1.014669 |
| C | -2.186710 | 5.365170 | 0.429169 |
| H | -2.855780 | 6.214410 | 0.369259 |
| F | -0.324770 | 6.718650 | -0.032661 |
| F | -3.970780 | 3.899180 | 0.902059 |
| C | 3.853150 | 0.363640 | 3.334819 |
| H | 2.934150 | 0.945860 | 3.455439 |
| H | 4.160400 | 0.006870 | 4.325089 |
| H | 4.628770 | 1.040190 | 2.960659 |
| C | 4.970300 | -1.600910 | 2.240459 |
| H | 4.863180 | -2.479010 | 1.592699 |
| H | 5.761050 | -0.967369 | 1.826119 |
| H | 5.305600 | -1.955420 | 3.222159 |
| C | 2.584520 | -1.772680 | 2.996109 |
| H | 1.608890 | -1.280310 | 3.083829 |
| H | 2.466340 | -2.682380 | 2.402669 |
| H | 2.893590 | -2.062810 | 4.007349 |

TS Conformation 58

B3LYP/6-31G(d) Energy = -3411.886926
M06-2X/def2tzvpp/IEFPCM(chloroform) Energy = -3411.891714
M06-2X/def2tzvpp/IEFPCM(chloroform)//B3LYP/6-31G(d) Quasiharmonic Free Energy = -3411.164881

Frequencies (Top 3 out of 291)

1. -898.7004 cm-1
2. 5.0166 cm-1
3. 10.9067 cm-1

B3LYP/6-31G(d) Molecular Geometry in Cartesian Coordinates

| | | | |
|---|---|---|---|
| C | 3.270760 | -0.389529 | 1.089100 |
| C | 2.773660 | 0.671071 | 2.091070 |
| H | 3.436310 | 1.535211 | 2.084170 |
| C | 1.313990 | 2.308681 | 0.886330 |
| N | 0.033720 | 2.424390 | 0.413760 |
| H | -0.537060 | 1.586410 | 0.512210 |
| N | 1.488430 | 1.156521 | 1.604230 |
| H | 0.739100 | 0.469250 | 1.571390 |
| C | 4.957030 | -1.185939 | -0.501140 |
| C | 5.477970 | 0.842691 | 0.788660 |
| C | 6.477170 | -0.901029 | -0.543140 |
| H | 4.748780 | -2.197159 | -0.139420 |
| C | 6.562730 | 0.607911 | -0.272690 |
| H | 5.019410 | 1.832591 | 0.715080 |
| H | 5.885770 | 0.716801 | 1.799000 |
| H | 6.926320 | -1.198649 | -1.494110 |
| H | 6.974310 | -1.460069 | 0.259170 |
| H | 6.327140 | 1.169481 | -1.183000 |
| H | 7.549650 | 0.928611 | 0.072640 |
| N | 4.483580 | -0.218749 | 0.507890 |
| O | 2.546270 | -1.353389 | 0.794250 |
| S | 2.562490 | 3.425491 | 0.630920 |
| C | 4.287070 | -1.019269 | -1.863160 |
| C | 3.686140 | 0.178731 | -2.268030 |
| C | 4.314840 | -2.094779 | -2.762770 |
| C | 3.133420 | 0.300051 | -3.546120 |
| H | 3.627380 | 1.018931 | -1.581650 |
| C | 3.766830 | -1.975879 | -4.039980 |
| C | 3.173790 | -0.773849 | -4.437640 |
| H | 2.669860 | 1.237871 | -3.839810 |
| O | -1.142130 | -0.285800 | 1.206340 |
| O | -1.124700 | 0.008600 | -1.684670 |
| C | -1.755110 | -1.345160 | 1.088570 |
| C | -1.933180 | -2.157410 | -0.142220 |
| O | -2.440330 | -1.906370 | 2.093410 |
| C | -2.558790 | -1.136480 | 3.304240 |
| H | -3.148060 | -1.759250 | 3.977020 |
| H | -1.575330 | -0.935320 | 3.734460 |
| H | -3.079110 | -0.198110 | 3.099410 |
| S | -1.037910 | -1.459000 | -1.538680 |
| C | 0.681490 | -2.005240 | -1.607570 |
| H | 0.706761 | -3.096020 | -1.610610 |
| H | 1.103630 | -1.592010 | -2.527520 |
| H | 1.208550 | -1.610770 | -0.732650 |
| C | -1.793520 | -2.265610 | -2.965190 |
| H | -1.266470 | -1.889130 | -3.844530 |

| | | | |
|---|---|---|---|
| H | -1.705129 | -3.349350 | -2.875890 |
| H | -2.843070 | -1.950960 | -2.961710 |
| C | -1.732809 | -3.652310 | -0.002350 |
| C | -2.695159 | -4.553750 | -0.482340 |
| C | -0.584119 | -4.161220 | 0.631150 |
| C | -2.504199 | -5.930140 | -0.345630 |
| H | -3.610499 | -4.174210 | -0.925140 |
| C | -0.398009 | -5.535720 | 0.768120 |
| H | 0.164841 | -3.476150 | 1.022660 |
| C | -1.356309 | -6.424480 | 0.274350 |
| H | -3.262389 | -6.614860 | -0.715580 |
| H | 0.493351 | -5.911620 | 1.262930 |
| H | -1.211919 | -7.496170 | 0.381590 |
| C | -5.677440 | -0.539190 | 1.225170 |
| C | -6.002250 | 0.548250 | 2.038140 |
| C | -5.880740 | 1.853770 | 1.554890 |
| C | -5.431140 | 2.059400 | 0.249390 |
| C | -5.104420 | 0.973710 | -0.565580 |
| C | -5.221160 | -0.343760 | -0.090940 |
| H | -5.784310 | -1.553570 | 1.598630 |
| H | -6.362710 | 0.373250 | 3.049830 |
| H | -6.137760 | 2.699780 | 2.187130 |
| H | -5.328390 | 3.065550 | -0.146320 |
| H | -4.758440 | 1.147270 | -1.579820 |
| S | -4.829380 | -1.742790 | -1.132680 |
| H | -3.233650 | -1.953790 | -0.552780 |
| H | 4.771141 | -3.035099 | -2.458850 |
| H | 3.802121 | -2.820319 | -4.723750 |
| H | 2.750280 | -0.675359 | -5.433760 |
| C | 2.693010 | 0.155881 | 3.569210 |
| C | -0.642070 | 3.468010 | -0.249950 |
| C | -0.215220 | 4.803390 | -0.281030 |
| C | -1.859250 | 3.109350 | -0.858780 |
| C | -1.012450 | 5.733730 | -0.935360 |
| H | 0.709350 | 5.116130 | 0.179090 |
| C | -2.607420 | 4.090080 | -1.486940 |
| H | -2.212510 | 2.085100 | -0.854670 |
| C | -2.217800 | 5.422500 | -1.551280 |
| H | -2.817810 | 6.172670 | -2.051060 |
| F | -0.595340 | 7.017070 | -0.963270 |
| F | -3.779870 | 3.736290 | -2.064150 |
| C | 1.671750 | -0.983489 | 3.748550 |
| H | 1.927280 | -1.855669 | 3.141800 |
| H | 1.640930 | -1.287859 | 4.801710 |
| H | 0.662170 | -0.661910 | 3.470880 |
| C | 2.293090 | 1.344501 | 4.465640 |
| H | 2.249990 | 1.028831 | 5.514830 |
| H | 3.016110 | 2.164841 | 4.388680 |
| H | 1.310590 | 1.739001 | 4.188860 |
| C | 4.086470 | -0.347629 | 3.995350 |
| H | 4.840940 | 0.444661 | 3.918780 |
| H | 4.063330 | -0.677389 | 5.040240 |
| H | 4.417950 | -1.199479 | 3.389850 |

TS Conformation 59

B3LYP/6-31G(d) Energy = -3411.885982
M06-2X/def2tzvpp/IEFPCM(chloroform) Energy = -3411.891434
M06-2X/def2tzvpp/IEFPCM(chloroform)//B3LYP/6-31G(d) Quasiharmonic Free Energy = -3411.164848

Frequencies (Top 3 out of 291)

1. -855.7279 cm-1
2. 10.5445 cm-1
3. 13.5394 cm-1

B3LYP/6-31G(d) Molecular Geometry in Cartesian Coordinates

| | | | |
|---|---|---|---|
| C | 3.020608 | -1.336405 | 0.035168 |
| C | 3.000361 | -0.132848 | 1.000360 |
| H | 3.831690 | 0.538763 | 0.793288 |
| C | 1.707146 | 1.822610 | 0.070259 |
| N | 0.404670 | 2.161718 | -0.203311 |
| H | -0.272858 | 1.420288 | -0.043210 |
| N | 1.783549 | 0.612550 | 0.698454 |
| H | 0.921964 | 0.083837 | 0.804829 |
| C | 5.068320 | -0.447313 | -1.198272 |
| C | 3.959417 | -2.544695 | -1.860040 |
| C | 5.344037 | -0.762390 | -2.690559 |
| H | 4.636689 | 0.553109 | -1.108463 |
| C | 5.148764 | -2.281614 | -2.791912 |
| H | 3.988735 | -3.521063 | -1.366907 |
| H | 3.002792 | -2.480151 | -2.393513 |
| H | 6.337464 | -0.428166 | -2.999584 |
| H | 4.602354 | -0.241720 | -3.308131 |
| H | 6.042177 | -2.805178 | -2.435688 |
| H | 4.951537 | -2.620102 | -3.813262 |
| N | 4.031260 | -1.459112 | -0.859801 |
| O | 2.086375 | -2.160616 | 0.044435 |
| S | 3.062774 | 2.752969 | -0.323079 |
| C | 6.322187 | -0.517349 | -0.337866 |
| C | 7.010527 | 0.671958 | -0.061098 |
| C | 6.845796 | -1.723805 | 0.145558 |
| C | 8.199885 | 0.655779 | 0.668773 |
| H | 6.604452 | 1.617774 | -0.412191 |
| C | 8.035529 | -1.742140 | 0.875005 |
| C | 8.718367 | -0.552870 | 1.136381 |
| H | 8.715526 | 1.589034 | 0.878574 |
| O | -2.817487 | 0.181028 | -1.743414 |
| O | -0.990676 | -0.472292 | 0.473058 |
| C | -3.634651 | -0.544989 | -1.184502 |
| C | -3.281910 | -1.742588 | -0.364447 |
| O | -4.952889 | -0.354982 | -1.220992 |
| C | -5.399788 | 0.826313 | -1.920696 |
| H | -4.936955 | 1.719232 | -1.495835 |
| H | -6.479459 | 0.846085 | -1.778552 |
| H | -5.148793 | 0.755137 | -2.982388 |
| S | -1.495336 | -1.718652 | -0.164424 |
| C | -1.107741 | -3.117244 | 0.895063 |
| H | -1.604627 | -2.908585 | 1.847489 |
| H | -0.019454 | -3.111289 | 0.995308 |
| H | -1.476617 | -4.040971 | 0.447411 |
| C | -0.584692 | -2.054462 | -1.694015 |
| H | -0.984087 | -2.969148 | -2.137639 |
| H | 0.466991 | -2.159488 | -1.407266 |
| H | -0.762534 | -1.191634 | -2.336929 |
| C | -3.819922 | -3.070914 | -0.847326 |
| C | -4.329400 | -4.010366 | 0.063050 |
| C | -3.839996 | -3.379789 | -2.219705 |
| C | -4.826816 | -5.233410 | -0.389683 |
| H | -4.374164 | -3.761261 | 1.118420 |
| C | -4.342434 | -4.599705 | -2.669560 |
| H | -3.475952 | -2.650881 | -2.940514 |

| | | | |
|---|---|---|---|
| C | -4.830671 | -5.533519 | -1.752666 |
| H | -5.224927 | -5.946875 | 0.326457 |
| H | -4.356578 | -4.818638 | -3.733741 |
| H | -5.222898 | -6.485014 | -2.101123 |
| C | -3.842241 | 1.233682 | 1.984561 |
| C | -4.289977 | 2.550435 | 1.856516 |
| C | -5.600954 | 2.888438 | 2.198758 |
| C | -6.460108 | 1.892274 | 2.669801 |
| C | -6.021209 | 0.573343 | 2.783591 |
| C | -4.704630 | 0.219587 | 2.439283 |
| H | -2.812968 | 0.984826 | 1.744188 |
| H | -3.604923 | 3.315503 | 1.497852 |
| H | -5.946132 | 3.914992 | 2.106646 |
| H | -7.482548 | 2.141773 | 2.945351 |
| H | -6.697605 | -0.199776 | 3.136279 |
| S | -4.162591 | -1.475137 | 2.575696 |
| H | -3.676527 | -1.583776 | 0.935675 |
| H | 6.310083 | -2.653216 | -0.025314 |
| H | 8.425230 | -2.686823 | 1.245162 |
| H | 9.642265 | -0.567349 | 1.708085 |
| C | 3.088828 | -0.517242 | 2.521300 |
| C | -0.194743 | 3.336407 | -0.700518 |
| C | 0.402284 | 4.604076 | -0.691919 |
| C | -1.512191 | 3.183515 | -1.171249 |
| C | -0.339923 | 5.679745 | -1.164325 |
| H | 1.410208 | 4.756952 | -0.336935 |
| C | -2.198581 | 4.300948 | -1.615428 |
| H | -1.992317 | 2.211662 | -1.215238 |
| C | -1.644469 | 5.575473 | -1.632095 |
| H | -2.195251 | 6.437339 | -1.987580 |
| F | 0.236506 | 6.899182 | -1.155422 |
| F | -3.466441 | 4.140058 | -2.058256 |
| C | 4.357221 | -1.358428 | 2.755581 |
| H | 5.260897 | -0.828108 | 2.439006 |
| H | 4.459135 | -1.586332 | 3.822938 |
| H | 4.317815 | -2.313409 | 2.218349 |
| C | 1.864295 | -1.309588 | 3.018963 |
| H | 1.785838 | -2.279564 | 2.521539 |
| H | 1.960406 | -1.486188 | 4.096652 |
| H | 0.926721 | -0.763300 | 2.866629 |
| C | 3.199482 | 0.795020 | 3.323853 |
| H | 4.080807 | 1.373787 | 3.025501 |
| H | 2.318076 | 1.427800 | 3.180636 |
| H | 3.285210 | 0.573309 | 4.394002 |

TS Conformation 60

B3LYP/6-31G(d) Energy = -3411.885982
M06-2X/def2tzvpp/IEFPCM(chloroform) Energy = -3411.891431
M06-2X/def2tzvpp/IEFPCM(chloroform)//B3LYP/6-31G(d) Quasiharmonic Free Energy = -3411.164844

Frequencies (Top 3 out of 291)

1. -855.5838 cm-1
2. 10.5440 cm-1
3. 13.5402 cm-1

B3LYP/6-31G(d) Molecular Geometry in Cartesian Coordinates

| | | | |
|---|---|---|---|
| C | 3.020730 | -1.336160 | 0.035271 |
| C | 3.000454 | -0.132625 | 1.000482 |

| | | | |
|---|---|---|---|
| H | 3.831692 | 0.539081 | 0.793355 |
| C | 1.707007 | 1.822716 | 0.070498 |
| N | 0.404489 | 2.161785 | -0.202887 |
| H | -0.273004 | 1.420363 | -0.042601 |
| N | 1.783540 | 0.612655 | 0.698687 |
| H | 0.922033 | 0.083791 | 0.804964 |
| C | 5.068421 | -0.447061 | -1.198182 |
| C | 3.959539 | -2.544447 | -1.859940 |
| C | 5.343887 | -0.761966 | -2.690548 |
| H | 4.636832 | 0.553361 | -1.108180 |
| C | 5.148715 | -2.281196 | -2.791990 |
| H | 3.989068 | -3.520826 | -1.366838 |
| H | 3.002818 | -2.480014 | -2.393247 |
| H | 6.337228 | -0.427632 | -2.999733 |
| H | 4.602038 | -0.241301 | -3.307925 |
| H | 6.042229 | -2.804717 | -2.435955 |
| H | 4.951344 | -2.619618 | -3.813334 |
| N | 4.031397 | -1.458878 | -0.859678 |
| O | 2.086485 | -2.160356 | 0.044530 |
| S | 3.062545 | 2.753149 | -0.322974 |
| C | 6.322431 | -0.517255 | -0.337996 |
| C | 6.845916 | -1.723763 | 0.145426 |
| C | 7.011027 | 0.671954 | -0.061440 |
| C | 8.035773 | -1.742249 | 0.874669 |
| H | 6.310015 | -2.653094 | -0.025291 |
| C | 8.200508 | 0.655625 | 0.668226 |
| C | 8.718861 | -0.553079 | 1.135837 |
| H | 8.425374 | -2.686972 | 1.244830 |
| O | -2.817218 | 0.180645 | -1.743621 |
| O | -0.990930 | -0.472276 | 0.473454 |
| C | -3.634517 | -0.545199 | -1.184680 |
| C | -3.281974 | -1.742711 | -0.364401 |
| O | -4.952732 | -0.355097 | -1.221368 |
| C | -5.399478 | 0.826098 | -1.921346 |
| H | -4.936459 | 1.719061 | -1.496784 |
| H | -5.148587 | 0.754571 | -2.983042 |
| H | -6.479135 | 0.846091 | -1.779127 |
| S | -1.495434 | -1.718738 | -0.163964 |
| C | -1.108100 | -3.117155 | 0.895848 |
| H | -1.605327 | -2.908400 | 1.848078 |
| H | -0.019849 | -3.111123 | 0.996461 |
| H | -1.476754 | -4.040969 | 0.448193 |
| C | -0.584459 | -2.054789 | -1.693305 |
| H | -0.762176 | -1.192058 | -2.336385 |
| H | -0.983764 | -2.969541 | -2.136873 |
| H | 0.467174 | -2.159746 | -1.406335 |
| C | -3.819841 | -3.071077 | -0.847335 |
| C | -3.839698 | -3.379978 | -2.219711 |
| C | -4.329403 | -4.010538 | 0.062985 |
| C | -4.342013 | -4.599926 | -2.669616 |
| H | -3.475570 | -2.651071 | -2.940478 |
| C | -4.826691 | -5.233615 | -0.389796 |
| H | -4.374339 | -3.761419 | 1.118344 |
| C | -4.830336 | -5.533748 | -1.752776 |
| H | -4.355990 | -4.818881 | -3.733795 |
| H | -5.224870 | -5.947087 | 0.326299 |
| H | -5.222467 | -6.485269 | -2.101272 |
| C | -6.021500 | 0.573691 | 2.783363 |
| C | -6.460115 | 1.892719 | 2.669607 |
| C | -5.600717 | 2.888729 | 2.198681 |
| C | -4.289786 | 2.550468 | 1.856519 |
| C | -3.842334 | 1.233615 | 1.984530 |

| | | | |
|---|---|---|---|
| C | -4.704967 | 0.219677 | 2.439141 |
| H | -6.698085 | -0.199304 | 3.135961 |
| H | -7.482524 | 2.142417 | 2.945091 |
| H | -5.945674 | 3.915360 | 2.106595 |
| H | -3.604547 | 3.315408 | 1.497935 |
| H | -2.813097 | 0.984563 | 1.744214 |
| S | -4.163296 | -1.475165 | 2.575542 |
| H | -3.676901 | -1.583837 | 0.935570 |
| H | 6.605061 | 1.617815 | -0.412540 |
| H | 8.716348 | 1.588806 | 0.877865 |
| H | 9.642855 | -0.567674 | 1.707384 |
| C | 3.089090 | -0.517034 | 2.521408 |
| C | -0.195000 | 3.336340 | -0.700323 |
| C | 0.401902 | 4.604069 | -0.691868 |
| C | -1.512401 | 3.183242 | -1.171114 |
| C | -0.340381 | 5.679592 | -1.164488 |
| H | 1.409784 | 4.757097 | -0.336832 |
| C | -2.198869 | 4.300540 | -1.615510 |
| H | -1.992421 | 2.211331 | -1.214990 |
| C | -1.644885 | 5.575118 | -1.632332 |
| H | -2.195727 | 6.436875 | -1.987988 |
| F | 0.235922 | 6.899089 | -1.155726 |
| F | -3.466682 | 4.139457 | -2.058421 |
| C | 1.864876 | -1.309870 | 3.019073 |
| H | 1.786827 | -2.279902 | 2.521692 |
| H | 1.961026 | -1.486375 | 4.096775 |
| H | 0.927086 | -0.763971 | 2.866680 |
| C | 3.199254 | 0.795237 | 3.324015 |
| H | 2.317465 | 1.427549 | 3.181093 |
| H | 3.285404 | 0.573495 | 4.394124 |
| H | 4.080179 | 1.374508 | 3.025459 |
| C | 4.357819 | -1.357730 | 2.755628 |
| H | 4.318837 | -2.312652 | 2.218262 |
| H | 5.261290 | -0.826994 | 2.439158 |
| H | 4.459784 | -1.585738 | 3.822957 |

TS Conformation 61

B3LYP/6-31G(d) Energy = -3411.883743
M06-2X/def2tzvpp/IEFPCM(chloroform) Energy = -3411.88955
M06-2X/def2tzvpp/IEFPCM(chloroform)//B3LYP/6-31G(d) Quasiharmonic Free Energy = -3411.164787

Frequencies (Top 3 out of 291)

1. -994.0259 cm-1
2. 4.7184 cm-1
3. 9.6264 cm-1

B3LYP/6-31G(d) Molecular Geometry in Cartesian Coordinates

| | | | |
|---|---|---|---|
| C | 2.118591 | -1.354379 | 0.625290 |
| C | 2.488491 | -0.031589 | 1.329170 |
| H | 3.326360 | 0.450232 | 0.828120 |
| C | 1.351659 | 2.095591 | 0.584390 |
| N | 0.110839 | 2.695640 | 0.650470 |
| H | -0.602101 | 2.133500 | 1.118160 |
| N | 1.342810 | 0.867491 | 1.174020 |
| H | 0.444210 | 0.471850 | 1.447990 |
| C | 3.649211 | -0.829078 | -1.340990 |
| C | 2.345472 | -2.911609 | -1.235790 |
| C | 3.374641 | -1.373118 | -2.766460 |

| | | | |
|---|---|---|---|
| H | 3.312790 | 0.208762 | -1.270100 |
| C | 3.147862 | -2.874438 | -2.542630 |
| H | 2.536513 | -3.802298 | -0.628550 |
| H | 1.267432 | -2.856989 | -1.414910 |
| H | 4.192971 | -1.148438 | -3.455850 |
| H | 2.457191 | -0.911059 | -3.149350 |
| H | 4.107592 | -3.390248 | -2.425820 |
| H | 2.609762 | -3.352698 | -3.366450 |
| N | 2.778412 | -1.695818 | -0.514080 |
| O | 1.230432 | -2.079549 | 1.087690 |
| S | 2.724719 | 2.765042 | -0.139340 |
| C | 5.125691 | -0.882477 | -0.965770 |
| C | 5.939730 | 0.214813 | -1.281040 |
| C | 5.713012 | -2.000097 | -0.359890 |
| C | 7.308090 | 0.190424 | -1.012320 |
| H | 5.492430 | 1.099033 | -1.729060 |
| C | 7.083242 | -2.026806 | -0.090140 |
| C | 7.885891 | -0.933596 | -0.418390 |
| H | 7.920890 | 1.053925 | -1.257580 |
| O | -4.541920 | 0.237838 | -2.434530 |
| O | -0.379349 | -1.028500 | -2.387140 |
| C | -4.268909 | -0.673312 | -1.655260 |
| C | -2.905559 | -1.159242 | -1.357930 |
| O | -5.192409 | -1.279573 | -0.891360 |
| C | -6.529889 | -0.751544 | -0.987680 |
| H | -6.902989 | -0.837834 | -2.011050 |
| H | -7.125169 | -1.358984 | -0.306650 |
| H | -6.545380 | 0.297226 | -0.682970 |
| S | -1.741859 | -0.475321 | -2.544250 |
| C | -2.300199 | -0.740421 | -4.251480 |
| H | -2.326038 | -1.823221 | -4.393470 |
| H | -3.288799 | -0.295522 | -4.365760 |
| H | -1.558489 | -0.286661 | -4.913230 |
| C | -1.721100 | 1.318439 | -2.355200 |
| H | -1.288190 | 1.518569 | -1.373150 |
| H | -1.068600 | 1.714510 | -3.136830 |
| H | -2.741130 | 1.693349 | -2.431660 |
| C | -2.635298 | -2.618731 | -1.121960 |
| C | -1.598858 | -2.998081 | -0.256160 |
| C | -3.381327 | -3.619582 | -1.770350 |
| C | -1.316787 | -4.347741 | -0.042690 |
| H | -1.001798 | -2.250010 | 0.252400 |
| C | -3.107497 | -4.966312 | -1.542270 |
| H | -4.194818 | -3.343242 | -2.436220 |
| C | -2.070266 | -5.333521 | -0.679990 |
| H | -0.504757 | -4.611680 | 0.627940 |
| H | -3.700446 | -5.728772 | -2.040490 |
| H | -1.855276 | -6.384661 | -0.506190 |
| C | -2.323839 | -0.408251 | 3.866800 |
| C | -2.710809 | -1.198991 | 4.948370 |
| C | -3.653238 | -2.214962 | 4.780470 |
| C | -4.210468 | -2.424692 | 3.518120 |
| C | -3.837069 | -1.630292 | 2.433390 |
| C | -2.881389 | -0.615422 | 2.595570 |
| H | -1.585810 | 0.376079 | 4.007330 |
| H | -2.266399 | -1.021771 | 5.924670 |
| H | -3.949578 | -2.834472 | 5.622570 |
| H | -4.947038 | -3.211323 | 3.371970 |
| H | -4.290118 | -1.796932 | 1.461800 |
| S | -2.396900 | 0.449329 | 1.242360 |
| H | -2.619209 | -0.450881 | -0.117780 |
| H | 5.092812 | -2.846257 | -0.078960 |

| | | | |
|---|---|---|---|
| H | 7.521262 | -2.902346 | 0.382330 |
| H | 8.951131 | -0.952875 | -0.204210 |
| C | 2.896431 | -0.221438 | 2.835540 |
| C | -0.384262 | 3.946680 | 0.231750 |
| C | 0.384208 | 4.978300 | -0.327410 |
| C | -1.770592 | 4.135739 | 0.416260 |
| C | -0.256843 | 6.158610 | -0.683880 |
| H | 1.447528 | 4.872301 | -0.477670 |
| C | -2.343402 | 5.339539 | 0.040090 |
| H | -2.394741 | 3.361109 | 0.850980 |
| C | -1.617233 | 6.383829 | -0.521220 |
| H | -2.083503 | 7.317959 | -0.808460 |
| F | 0.491057 | 7.144290 | -1.221180 |
| F | -3.670032 | 5.503588 | 0.221750 |
| C | 3.521750 | 1.099632 | 3.326480 |
| H | 3.796980 | 1.013192 | 4.384620 |
| H | 4.424880 | 1.353293 | 2.760160 |
| H | 2.821930 | 1.936342 | 3.229930 |
| C | 1.695861 | -0.573739 | 3.734750 |
| H | 2.042861 | -0.733999 | 4.762770 |
| H | 0.967100 | 0.244681 | 3.764750 |
| H | 1.186001 | -1.476119 | 3.391370 |
| C | 3.951031 | -1.341448 | 2.930270 |
| H | 3.526542 | -2.315228 | 2.663270 |
| H | 4.808901 | -1.147287 | 2.276490 |
| H | 4.325061 | -1.412857 | 3.958390 |

TS Conformation 62

B3LYP/6-31G(d) Energy = -3411.891646
M06-2X/def2tzvpp/IEFPCM(chloroform) Energy = -3411.891037
M06-2X/def2tzvpp/IEFPCM(chloroform)//B3LYP/6-31G(d) Quasiharmonic Free Energy = -3411.164704

Frequencies (Top 3 out of 291)

1. -983.6528 cm-1
2. 8.7543 cm-1
3. 11.2358 cm-1

B3LYP/6-31G(d) Molecular Geometry in Cartesian Coordinates

| | | | |
|---|---|---|---|
| C | -2.784015 | -1.758647 | -0.278728 |
| C | -3.356772 | -0.644661 | -1.179255 |
| H | -4.285357 | -0.248372 | -0.770652 |
| C | -2.568380 | 1.703823 | -0.647201 |
| N | -1.371063 | 2.370687 | -0.543485 |
| H | -0.556220 | 1.785208 | -0.701600 |
| N | -2.371101 | 0.433717 | -1.119718 |
| H | -1.411754 | 0.099716 | -1.129309 |
| C | -4.534535 | -1.308358 | 1.513885 |
| C | -2.797206 | -3.030681 | 1.800930 |
| C | -4.303502 | -1.607074 | 3.018011 |
| H | -4.403197 | -0.238983 | 1.327511 |
| C | -3.715777 | -3.025140 | 3.028603 |
| H | -2.704289 | -4.009626 | 1.321210 |
| H | -1.783675 | -2.690196 | 2.047657 |
| H | -5.225094 | -1.510965 | 3.597252 |
| H | -3.573978 | -0.892254 | 3.417331 |
| H | -4.512611 | -3.768232 | 2.917132 |
| H | -3.172578 | -3.255876 | 3.949530 |
| N | -3.423020 | -2.062534 | 0.878119 |

| | | | |
|---|---|---|---|
| O | -1.722349 | -2.325226 | -0.588407 |
| S | -4.089656 | 2.328839 | -0.276098 |
| C | -5.922466 | -1.714243 | 1.035985 |
| C | -6.938803 | -0.748843 | 1.028912 |
| C | -6.237216 | -3.024273 | 0.652250 |
| C | -8.241811 | -1.087070 | 0.662423 |
| H | -6.702865 | 0.277275 | 1.300991 |
| C | -7.540677 | -3.364469 | 0.285670 |
| C | -8.547836 | -2.398059 | 0.293000 |
| H | -9.014775 | -0.323318 | 0.656475 |
| O | 2.720852 | 1.472333 | -1.697533 |
| O | 0.411745 | 0.100493 | -0.075501 |
| C | 3.419943 | 1.077631 | -0.769319 |
| C | 3.213814 | -0.201927 | -0.039397 |
| O | 4.501897 | 1.733674 | -0.328546 |
| C | 4.787567 | 2.986801 | -0.987624 |
| H | 4.887645 | 2.836633 | -2.064512 |
| H | 5.728191 | 3.325483 | -0.554366 |
| H | 3.990803 | 3.708035 | -0.789152 |
| S | 1.566403 | -0.778710 | -0.429225 |
| C | 1.501220 | -1.224765 | -2.177137 |
| H | 1.518696 | -0.285700 | -2.728521 |
| H | 0.563814 | -1.769373 | -2.313027 |
| H | 2.377268 | -1.831945 | -2.421985 |
| C | 1.412893 | -2.359944 | 0.412040 |
| H | 0.414829 | -2.730341 | 0.158814 |
| H | 1.502376 | -2.165017 | 1.481644 |
| H | 2.223415 | -3.009965 | 0.067263 |
| C | 3.470704 | -0.219366 | 1.450291 |
| C | 4.312989 | -1.189010 | 2.014176 |
| C | 2.871900 | 0.735633 | 2.290974 |
| C | 4.556384 | -1.196191 | 3.388864 |
| H | 4.786758 | -1.926059 | 1.374670 |
| C | 3.113810 | 0.722524 | 3.663346 |
| H | 2.216683 | 1.491339 | 1.866091 |
| C | 3.957867 | -0.244383 | 4.215150 |
| H | 5.220123 | -1.945853 | 3.810505 |
| H | 2.645910 | 1.468304 | 4.300036 |
| H | 4.150343 | -0.252173 | 5.284681 |
| C | 6.980673 | -1.131715 | -0.538571 |
| C | 8.313827 | -1.050220 | -0.133283 |
| C | 9.115209 | -2.191956 | -0.087290 |
| C | 8.563659 | -3.423814 | -0.447337 |
| C | 7.229571 | -3.514538 | -0.841476 |
| C | 6.415975 | -2.368671 | -0.896124 |
| H | 6.376307 | -0.230393 | -0.578103 |
| H | 8.727892 | -0.082960 | 0.142518 |
| H | 10.154289 | -2.123465 | 0.223429 |
| H | 9.173866 | -4.323621 | -0.416772 |
| H | 6.805866 | -4.477418 | -1.112298 |
| S | 4.723293 | -2.529262 | -1.435014 |
| H | 3.985441 | -1.201349 | -0.671276 |
| H | -5.456960 | -3.779157 | 0.616888 |
| H | -7.766746 | -4.385120 | -0.011714 |
| H | -9.561171 | -2.662526 | 0.003410 |
| C | -3.663788 | -1.105889 | -2.649148 |
| C | -1.003971 | 3.688779 | -0.226919 |
| C | -1.870745 | 4.692518 | 0.225425 |
| C | 0.369618 | 3.968124 | -0.384838 |
| C | -1.332506 | 5.943199 | 0.506601 |
| H | -2.927083 | 4.510678 | 0.355612 |
| C | 0.836863 | 5.233853 | -0.080431 |

| | | | |
|---|---|---|---|
| H | 1.058639 | 3.220266 | -0.765463 |
| C | 0.014363 | 6.258566 | 0.372738 |
| H | 0.397672 | 7.244511 | 0.603680 |
| F | -2.168439 | 6.906097 | 0.943789 |
| F | 2.158831 | 5.485608 | -0.235185 |
| C | -2.389891 | -1.432331 | -3.451587 |
| H | -1.844039 | -2.271226 | -3.013043 |
| H | -2.662504 | -1.698054 | -4.479793 |
| H | -1.722037 | -0.564537 | -3.507550 |
| C | -4.415872 | 0.041294 | -3.352721 |
| H | -3.818924 | 0.958531 | -3.373360 |
| H | -4.642206 | -0.239581 | -4.388309 |
| H | -5.360278 | 0.271553 | -2.848034 |
| C | -4.568715 | -2.351853 | -2.605230 |
| H | -4.055713 | -3.208904 | -2.153510 |
| H | -5.487741 | -2.165932 | -2.039252 |
| H | -4.856272 | -2.639459 | -3.623216 |

TS Conformation 63

B3LYP/6-31G(d) Energy = -3411.881293
M06-2X/def2tzvpp/IEFPCM(chloroform) Energy = -3411.891581
M06-2X/def2tzvpp/IEFPCM(chloroform)//B3LYP/6-31G(d) Quasiharmonic Free Energy = -3411.164625

Frequencies (Top 3 out of 291)

1. -1002.1848 cm-1
2. 3.7195 cm-1
3. 7.4727 cm-1

B3LYP/6-31G(d) Molecular Geometry in Cartesian Coordinates

| | | | |
|---|---|---|---|
| C | -2.583189 | -1.530968 | 0.915986 |
| C | -1.629723 | -2.568630 | 1.540897 |
| H | -1.756142 | -3.544580 | 1.071310 |
| C | 0.685093 | -2.945514 | 0.650163 |
| N | 1.875760 | -2.273247 | 0.512775 |
| H | 1.852714 | -1.294711 | 0.800504 |
| N | -0.267466 | -2.159076 | 1.237113 |
| H | -0.047802 | -1.183645 | 1.415394 |
| C | -4.775568 | -1.065688 | -0.110183 |
| C | -3.991769 | -3.395294 | -0.082000 |
| C | -5.977275 | -2.038654 | -0.191943 |
| H | -4.889544 | -0.350980 | 0.710389 |
| C | -5.353126 | -3.307371 | -0.789258 |
| H | -3.212241 | -3.841757 | -0.706737 |
| H | -4.072798 | -3.986104 | 0.834719 |
| H | -6.797395 | -1.634470 | -0.790526 |
| H | -6.355158 | -2.238887 | 0.818256 |
| H | -5.216645 | -3.187254 | -1.869126 |
| H | -5.956371 | -4.204544 | -0.624839 |
| N | -3.670202 | -1.983420 | 0.235247 |
| O | -2.369452 | -0.319454 | 1.066267 |
| S | 0.388341 | -4.539096 | 0.175012 |
| C | -4.597510 | -0.282309 | -1.406013 |
| C | -3.714838 | -0.671845 | -2.416478 |
| C | -5.409979 | 0.842356 | -1.618882 |
| C | -3.659753 | 0.031594 | -3.623415 |
| H | -3.044729 | -1.510228 | -2.256120 |
| C | -5.356800 | 1.548036 | -2.819960 |
| C | -4.482756 | 1.138459 | -3.832787 |

| | | | |
|---|---|---|---|
| H | -2.961216 | -0.285723 | -4.391998 |
| O | 1.367617 | 0.605437 | 1.174656 |
| O | -0.639688 | 0.380305 | -2.419347 |
| C | 0.738574 | 1.489850 | 0.600653 |
| C | 0.979257 | 1.980000 | -0.793799 |
| O | -0.202866 | 2.225319 | 1.203612 |
| C | -0.483126 | 1.967619 | 2.594693 |
| H | 0.446884 | 1.788093 | 3.135587 |
| H | -0.965609 | 2.874951 | 2.956957 |
| H | -1.149542 | 1.108441 | 2.668373 |
| S | -0.401098 | 1.737999 | -1.867954 |
| C | -1.892924 | 2.380919 | -1.085884 |
| H | -2.646402 | 2.417273 | -1.876450 |
| H | -2.185196 | 1.661889 | -0.317350 |
| H | -1.669758 | 3.365232 | -0.669568 |
| C | -0.108491 | 2.888483 | -3.229454 |
| H | -0.924515 | 2.742698 | -3.940122 |
| H | -0.086118 | 3.907446 | -2.834638 |
| H | 0.848526 | 2.618084 | -3.678419 |
| C | 2.257543 | 1.531302 | -1.467391 |
| C | 3.291283 | 2.469211 | -1.632571 |
| C | 2.452407 | 0.220217 | -1.934055 |
| C | 4.490810 | 2.101152 | -2.242725 |
| H | 3.149184 | 3.486705 | -1.281096 |
| C | 3.649876 | -0.140530 | -2.552551 |
| H | 1.665334 | -0.516061 | -1.817519 |
| C | 4.671841 | 0.797417 | -2.706931 |
| H | 5.282557 | 2.836771 | -2.356488 |
| H | 3.785838 | -1.160477 | -2.900365 |
| H | 5.606235 | 0.511688 | -3.182614 |
| C | -0.324574 | 5.907250 | 1.771572 |
| C | -0.392173 | 6.097495 | 3.153077 |
| C | 0.657461 | 5.683550 | 3.976474 |
| C | 1.781694 | 5.086666 | 3.400729 |
| C | 1.857120 | 4.905812 | 2.019056 |
| C | 0.799446 | 5.304182 | 1.183757 |
| H | -1.144362 | 6.229563 | 1.135899 |
| H | -1.269561 | 6.572350 | 3.586413 |
| H | 0.604482 | 5.832797 | 5.051670 |
| H | 2.611576 | 4.769829 | 4.028183 |
| H | 2.744344 | 4.460303 | 1.578086 |
| S | 0.892586 | 5.092418 | -0.589008 |
| H | 0.969018 | 3.459652 | -0.660647 |
| H | -6.093893 | 1.164963 | -0.836022 |
| H | -5.997565 | 2.413617 | -2.967415 |
| H | -4.446712 | 1.679217 | -4.775076 |
| C | -1.863746 | -2.757186 | 3.094799 |
| C | 3.175801 | -2.702277 | 0.181332 |
| C | 3.484413 | -3.883482 | -0.508277 |
| C | 4.211892 | -1.828370 | 0.564806 |
| C | 4.820193 | -4.157757 | -0.776289 |
| H | 2.715169 | -4.573405 | -0.819777 |
| C | 5.521312 | -2.158109 | 0.258130 |
| H | 4.002322 | -0.900295 | 1.085433 |
| C | 5.870448 | -3.324858 | -0.412734 |
| H | 6.900955 | -3.569852 | -0.637760 |
| F | 5.111368 | -5.296741 | -1.438439 |
| F | 6.503231 | -1.312239 | 0.632658 |
| C | -3.302487 | -3.242996 | 3.359754 |
| H | -3.461183 | -3.356423 | 4.438236 |
| H | -4.056171 | -2.537014 | 2.991962 |
| H | -3.488196 | -4.221562 | 2.902238 |

| | | | |
|---|---|---|---|
| C | -1.628629 | -1.454823 | 3.881482 |
| H | -1.736108 | -1.650653 | 4.954975 |
| H | -0.617078 | -1.065768 | 3.722342 |
| H | -2.344580 | -0.675677 | 3.605335 |
| C | -0.884705 | -3.832365 | 3.607462 |
| H | 0.156276 | -3.519071 | 3.486329 |
| H | -1.059552 | -4.010417 | 4.675435 |
| H | -1.009380 | -4.780246 | 3.075002 |

TS Conformation 64

B3LYP/6-31G(d) Energy = -3411.892091
M06-2X/def2tzvpp/IEFPCM(chloroform) Energy = -3411.891029
M06-2X/def2tzvpp/IEFPCM(chloroform)//B3LYP/6-31G(d) Quasiharmonic Free Energy = -3411.164607

Frequencies (Top 3 out of 291)

1. -993.7449 cm-1
2. 6.8230 cm-1
3. 9.6252 cm-1

B3LYP/6-31G(d) Molecular Geometry in Cartesian Coordinates

| | | | |
|---|---|---|---|
| C | -2.869015 | -1.643750 | 0.174628 |
| C | -3.145830 | -0.723961 | -1.034329 |
| H | -4.074409 | -0.171132 | -0.907098 |
| C | -2.174310 | 1.612462 | -0.875631 |
| N | -0.934262 | 2.191018 | -0.742240 |
| H | -0.170428 | 1.524086 | -0.680999 |
| N | -2.060916 | 0.256129 | -1.010428 |
| H | -1.153677 | -0.150631 | -0.798436 |
| C | -4.890304 | -0.705649 | 1.421047 |
| C | -3.330192 | -2.266495 | 2.495830 |
| C | -5.152968 | -0.786905 | 2.950984 |
| H | -4.577430 | 0.310252 | 1.158930 |
| C | -3.835048 | -1.285746 | 3.556722 |
| H | -3.811896 | -3.249035 | 2.594468 |
| H | -2.250946 | -2.420539 | 2.496537 |
| H | -5.953276 | -1.508793 | 3.146958 |
| H | -5.480029 | 0.179894 | 3.343790 |
| H | -3.966646 | -1.754318 | 4.536925 |
| H | -3.122047 | -0.460219 | 3.667423 |
| N | -3.726907 | -1.625025 | 1.229531 |
| O | -1.834281 | -2.329286 | 0.192291 |
| S | -3.650371 | 2.429661 | -0.897826 |
| C | -6.120787 | -1.063162 | 0.603036 |
| C | -6.625105 | -2.371570 | 0.580182 |
| C | -6.802147 | -0.064698 | -0.102370 |
| C | -7.784526 | -2.673594 | -0.131766 |
| H | -6.100067 | -3.161356 | 1.112152 |
| C | -7.966490 | -0.365420 | -0.814473 |
| C | -8.460833 | -1.669280 | -0.830669 |
| H | -8.160349 | -3.693348 | -0.143828 |
| O | 3.278060 | 0.872794 | -1.049600 |
| O | 0.604835 | -0.077962 | 0.276542 |
| C | 3.768792 | 0.580968 | 0.036647 |
| C | 3.313368 | -0.545544 | 0.895817 |
| O | 4.807934 | 1.230550 | 0.577957 |
| C | 5.325701 | 2.330167 | -0.201038 |
| H | 4.564094 | 3.104567 | -0.319679 |
| H | 6.172364 | 2.707934 | 0.371872 |

| | | | |
|---|---|---|---|
| H | 5.647611 | 1.977576 | -1.183603 |
| S | 1.721550 | -1.071762 | 0.286732 |
| C | 1.943888 | -1.799989 | -1.348673 |
| H | 2.219959 | -0.982579 | -2.013227 |
| H | 0.983860 | -2.247310 | -1.617096 |
| H | 2.743995 | -2.542988 | -1.271546 |
| C | 1.267384 | -2.479005 | 1.309930 |
| H | 0.262184 | -2.767048 | 0.986748 |
| H | 1.262250 | -2.138564 | 2.346278 |
| H | 2.013353 | -3.264259 | 1.157474 |
| C | 3.313063 | -0.327285 | 2.393955 |
| C | 4.135938 | -1.108073 | 3.219237 |
| C | 2.514742 | 0.672429 | 2.978181 |
| C | 4.157840 | -0.892652 | 4.598403 |
| H | 4.771460 | -1.868722 | 2.776739 |
| C | 2.532794 | 0.877724 | 4.356983 |
| H | 1.880403 | 1.290971 | 2.349243 |
| C | 3.355367 | 0.095089 | 5.170549 |
| H | 4.806212 | -1.499344 | 5.224667 |
| H | 1.910760 | 1.654317 | 4.793586 |
| H | 3.374004 | 0.259336 | 6.244638 |
| C | 6.490186 | -1.449969 | -1.159792 |
| C | 7.147068 | -1.043131 | -2.321886 |
| C | 7.065694 | -1.807787 | -3.487544 |
| C | 6.318113 | -2.986899 | -3.478478 |
| C | 5.650023 | -3.392271 | -2.322317 |
| C | 5.723346 | -2.628528 | -1.144859 |
| H | 6.575333 | -0.859764 | -0.251990 |
| H | 7.736705 | -0.129103 | -2.311699 |
| H | 7.582905 | -1.491761 | -4.389408 |
| H | 6.250763 | -3.595729 | -4.377094 |
| H | 5.069778 | -4.310954 | -2.322276 |
| S | 4.879873 | -3.166422 | 0.336850 |
| H | 4.092849 | -1.718633 | 0.628637 |
| H | -6.409343 | 0.948939 | -0.107227 |
| H | -8.480823 | 0.421071 | -1.360068 |
| H | -9.364352 | -1.905193 | -1.386341 |
| C | -3.239004 | -1.482095 | -2.407802 |
| C | -0.472969 | 3.513504 | -0.633826 |
| C | -1.280070 | 4.653040 | -0.514973 |
| C | 0.931449 | 3.647461 | -0.635404 |
| C | -0.654562 | 5.889111 | -0.399868 |
| H | -2.357526 | 4.584807 | -0.513366 |
| C | 1.485281 | 4.908932 | -0.511552 |
| H | 1.583544 | 2.787370 | -0.754278 |
| C | 0.723711 | 6.065269 | -0.389506 |
| H | 1.175871 | 7.044653 | -0.296145 |
| F | -1.434594 | 6.982718 | -0.283670 |
| F | 2.835084 | 5.022343 | -0.510928 |
| C | -1.882035 | -2.052602 | -2.864298 |
| H | -1.498039 | -2.786720 | -2.151452 |
| H | -1.999436 | -2.543050 | -3.837848 |
| H | -1.139038 | -1.255994 | -2.989967 |
| C | -3.738077 | -0.477151 | -3.464924 |
| H | -3.062898 | 0.379874 | -3.554115 |
| H | -3.799598 | -0.963894 | -4.445750 |
| H | -4.732325 | -0.092657 | -3.213169 |
| C | -4.255475 | -2.633236 | -2.285737 |
| H | -3.924438 | -3.391752 | -1.567097 |
| H | -5.240936 | -2.272839 | -1.974236 |
| H | -4.373332 | -3.128452 | -3.256778 |

TS Conformation 65

B3LYP/6-31G(d) Energy = -3411.883567
M06-2X/def2tzvpp/IEFPCM(chloroform) Energy = -3411.889989
M06-2X/def2tzvpp/IEFPCM(chloroform)//B3LYP/6-31G(d) Quasiharmonic Free Energy = -3411.164559

Frequencies (Top 3 out of 291)

1. -1006.4992 cm-1
2. 9.7337 cm-1
3. 11.8350 cm-1

B3LYP/6-31G(d) Molecular Geometry in Cartesian Coordinates

| | | | |
|---|---|---|---|
| C | -2.637070 | -1.545131 | -0.319944 |
| C | -2.912923 | -0.361258 | -1.271284 |
| H | -3.716926 | 0.266786 | -0.888850 |
| C | -1.597442 | 1.747292 | -0.802896 |
| N | -0.286325 | 2.073134 | -0.519536 |
| H | 0.285326 | 1.288164 | -0.196693 |
| N | -1.698235 | 0.454506 | -1.223947 |
| H | -0.830022 | -0.057579 | -1.344093 |
| C | -3.985175 | -0.445583 | 1.534831 |
| C | -2.792278 | -2.572494 | 1.882711 |
| C | -3.701282 | -0.673079 | 3.042508 |
| H | -3.578835 | 0.521768 | 1.227377 |
| C | -3.547108 | -2.195652 | 3.162959 |
| H | -3.036278 | -3.569765 | 1.504273 |
| H | -1.704635 | -2.521215 | 2.010938 |
| H | -4.493361 | -0.260443 | 3.672659 |
| H | -2.759264 | -0.178139 | 3.306045 |
| H | -4.531653 | -2.676322 | 3.187469 |
| H | -3.004368 | -2.498091 | 4.063108 |
| N | -3.206909 | -1.539951 | 0.908862 |
| O | -1.858924 | -2.450927 | -0.653579 |
| S | -2.915805 | 2.780122 | -0.619782 |
| C | -5.471517 | -0.471331 | 1.198381 |
| C | -6.194511 | 0.728836 | 1.251581 |
| C | -6.152996 | -1.652171 | 0.878568 |
| C | -7.567575 | 0.746178 | 1.007506 |
| H | -5.672796 | 1.656969 | 1.472854 |
| C | -7.527961 | -1.636512 | 0.633665 |
| C | -8.240269 | -0.438453 | 0.700682 |
| H | -8.109526 | 1.687384 | 1.046580 |
| O | 3.352678 | -4.423452 | -0.336848 |
| O | 1.786044 | -0.990415 | -2.558901 |
| C | 3.656837 | -3.307497 | 0.077184 |
| C | 3.238564 | -2.013657 | -0.507796 |
| O | 4.387354 | -3.123553 | 1.192297 |
| C | 4.780822 | -4.320871 | 1.887958 |
| H | 5.385086 | -4.960615 | 1.239880 |
| H | 5.363789 | -3.976438 | 2.741709 |
| H | 3.900047 | -4.874958 | 2.220908 |
| S | 2.281303 | -2.270292 | -2.002739 |
| C | 3.272153 | -3.143258 | -3.244131 |
| H | 2.653920 | -3.274257 | -4.135350 |
| H | 4.128376 | -2.501232 | -3.461781 |
| H | 3.585166 | -4.096759 | -2.815237 |
| C | 0.913102 | -3.390453 | -1.663536 |
| H | 0.408622 | -3.539923 | -2.621202 |
| H | 1.312449 | -4.315957 | -1.252625 |

| | | | |
|---|---|---|---|
| H | 0.222950 | -2.893993 | -0.974477 |
| C | 4.290751 | -0.952567 | -0.715626 |
| C | 5.623280 | -1.298061 | -1.006246 |
| C | 3.956887 | 0.405713 | -0.617360 |
| C | 6.594191 | -0.312276 | -1.169577 |
| H | 5.907452 | -2.344982 | -1.080985 |
| C | 4.929002 | 1.393463 | -0.777164 |
| H | 2.932621 | 0.682774 | -0.402340 |
| C | 6.249213 | 1.036570 | -1.052361 |
| H | 7.620667 | -0.597513 | -1.384040 |
| H | 4.655437 | 2.437819 | -0.658186 |
| H | 7.008566 | 1.804752 | -1.170307 |
| C | 3.095862 | -0.316056 | 3.021622 |
| C | 3.702915 | 0.356126 | 4.083230 |
| C | 3.060485 | 1.423351 | 4.713175 |
| C | 1.798499 | 1.817399 | 4.264939 |
| C | 1.188194 | 1.156052 | 3.199070 |
| C | 1.827961 | 0.077780 | 2.562536 |
| H | 3.606129 | -1.149248 | 2.549530 |
| H | 4.687370 | 0.039045 | 4.419827 |
| H | 3.537341 | 1.942527 | 5.540045 |
| H | 1.284846 | 2.649150 | 4.740880 |
| H | 0.208655 | 1.475806 | 2.853786 |
| S | 0.987097 | -0.764784 | 1.228107 |
| H | 2.224671 | -1.452017 | 0.363966 |
| H | -5.603795 | -2.586192 | 0.802694 |
| H | -8.040607 | -2.562429 | 0.385672 |
| H | -9.309310 | -0.425960 | 0.505868 |
| C | -3.319997 | -0.782961 | -2.725485 |
| C | 0.364160 | 3.315067 | -0.512630 |
| C | -0.122436 | 4.449687 | -1.179341 |
| C | 1.615816 | 3.366803 | 0.127206 |
| C | 0.658776 | 5.596504 | -1.185305 |
| H | -1.082164 | 4.450355 | -1.675061 |
| C | 2.356344 | 4.535958 | 0.058288 |
| H | 2.001916 | 2.519747 | 0.683161 |
| C | 1.911320 | 5.682329 | -0.586714 |
| H | 2.502967 | 6.588367 | -0.622821 |
| F | 0.186799 | 6.684157 | -1.828673 |
| F | 3.580992 | 4.551799 | 0.634326 |
| C | -3.758474 | 0.485969 | -3.483624 |
| H | -2.947089 | 1.218084 | -3.542402 |
| H | -4.055516 | 0.224464 | -4.506628 |
| H | -4.607873 | 0.974649 | -2.995002 |
| C | -2.171428 | -1.453370 | -3.505083 |
| H | -1.871806 | -2.392574 | -3.036051 |
| H | -2.501022 | -1.662235 | -4.530297 |
| H | -1.292883 | -0.800701 | -3.572348 |
| C | -4.512941 | -1.755122 | -2.636370 |
| H | -4.228352 | -2.697598 | -2.155674 |
| H | -5.346618 | -1.320528 | -2.072730 |
| H | -4.878749 | -1.991913 | -3.642465 |

TS Conformation 66

B3LYP/6-31G(d) Energy = -3411.883463
M06-2X/def2tzvpp/IEFPCM(chloroform) Energy = -3411.891864
M06-2X/def2tzvpp/IEFPCM(chloroform)//B3LYP/6-31G(d) Quasiharmonic Free Energy = -3411.164523

Frequencies (Top 3 out of 291)

1. -888.8428 cm-1
2. 10.5824 cm-1
3. 14.2978 cm-1

B3LYP/6-31G(d) Molecular Geometry in Cartesian Coordinates

| | | | |
|---|---|---|---|
| C | -3.555910 | -0.919167 | -0.390005 |
| C | -3.732044 | 0.377312 | -1.208119 |
| H | -4.378079 | 1.085822 | -0.688786 |
| C | -2.118730 | 2.267252 | -0.806402 |
| N | -0.763628 | 2.479025 | -0.790577 |
| H | -0.204251 | 1.663204 | -1.024088 |
| N | -2.410874 | 0.997793 | -1.247184 |
| H | -1.644139 | 0.330192 | -1.244629 |
| C | -4.145544 | -2.314053 | 1.555548 |
| C | -5.462023 | -0.280855 | 1.160571 |
| C | -5.304398 | -2.200705 | 2.581531 |
| H | -4.292840 | -3.187049 | 0.907856 |
| C | -6.337922 | -1.293883 | 1.904294 |
| H | -5.126438 | 0.525256 | 1.825541 |
| H | -5.974735 | 0.170820 | 0.312377 |
| H | -4.930601 | -1.728069 | 3.496432 |
| H | -5.694473 | -3.185146 | 2.854878 |
| H | -7.018548 | -0.812430 | 2.612507 |
| H | -6.941088 | -1.860886 | 1.185009 |
| N | -4.312159 | -1.098036 | 0.720098 |
| O | -2.694337 | -1.748536 | -0.727474 |
| S | -3.302855 | 3.388549 | -0.381811 |
| C | -2.793670 | -2.446333 | 2.237953 |
| C | -2.040150 | -1.330927 | 2.624378 |
| C | -2.326297 | -3.723888 | 2.570109 |
| C | -0.841063 | -1.489972 | 3.320249 |
| H | -2.381454 | -0.332876 | 2.362988 |
| C | -1.134594 | -3.885200 | 3.280744 |
| C | -0.385520 | -2.767952 | 3.655444 |
| H | -0.249523 | -0.618977 | 3.586478 |
| O | 2.375307 | 0.453322 | -2.264245 |
| O | 0.221890 | -0.307119 | -0.466579 |
| C | 3.091900 | -0.301029 | -1.613343 |
| C | 2.634121 | -1.546853 | -0.924914 |
| O | 4.394813 | -0.101497 | -1.406145 |
| C | 4.953255 | 1.102295 | -1.973317 |
| H | 4.794844 | 1.123414 | -3.054637 |
| H | 4.497899 | 1.985810 | -1.520707 |
| H | 6.016259 | 1.057237 | -1.738909 |
| S | 0.838562 | -1.558106 | -0.979604 |
| C | 0.182529 | -1.925658 | -2.628242 |
| H | -0.900687 | -2.013959 | -2.512441 |
| H | 0.634848 | -2.849448 | -2.994271 |
| H | 0.459047 | -1.075656 | -3.254047 |
| C | 0.312722 | -2.948629 | 0.025236 |
| H | -0.779463 | -2.892741 | 0.044508 |
| H | 0.679312 | -3.879180 | -0.410363 |
| H | 0.734240 | -2.777145 | 1.020685 |
| C | 3.249549 | -2.841203 | -1.407245 |
| C | 3.518142 | -3.891262 | -0.514064 |
| C | 3.578196 | -3.012979 | -2.764455 |
| C | 4.087030 | -5.081097 | -0.970832 |
| H | 3.306559 | -3.769576 | 0.542543 |
| C | 4.154475 | -4.198666 | -3.216859 |
| H | 3.392435 | -2.205153 | -3.468595 |
| C | 4.405178 | -5.239660 | -2.320188 |

| | | | |
|---|---|---|---|
| H | 4.294413 | -5.879883 | -0.264286 |
| H | 4.407830 | -4.308562 | -4.267749 |
| H | 4.853884 | -6.165055 | -2.670633 |
| C | 2.207704 | 1.003779 | 2.246463 |
| C | 2.244573 | 2.336347 | 2.663104 |
| C | 3.302585 | 2.806578 | 3.443012 |
| C | 4.330076 | 1.928658 | 3.796604 |
| C | 4.304398 | 0.600783 | 3.371678 |
| C | 3.241037 | 0.113288 | 2.590533 |
| H | 1.371424 | 0.648872 | 1.651656 |
| H | 1.437646 | 3.007574 | 2.378922 |
| H | 3.325785 | 3.842601 | 3.770698 |
| H | 5.162091 | 2.280372 | 4.402919 |
| H | 5.111587 | -0.074656 | 3.639993 |
| S | 3.227921 | -1.601918 | 2.099267 |
| H | 2.885260 | -1.461073 | 0.423811 |
| H | -2.900738 | -4.599429 | 2.272578 |
| H | -0.788549 | -4.884378 | 3.533434 |
| H | 0.556677 | -2.884732 | 4.181632 |
| C | -4.348590 | 0.135646 | -2.634974 |
| C | 0.040488 | 3.607990 | -0.542010 |
| C | 1.389307 | 3.464585 | -0.921169 |
| C | -0.392652 | 4.797553 | 0.059616 |
| C | 2.267199 | 4.506124 | -0.682514 |
| H | 1.748903 | 2.559048 | -1.399035 |
| C | 0.542416 | 5.807268 | 0.258622 |
| H | -1.419415 | 4.939340 | 0.360978 |
| C | 1.882540 | 5.704836 | -0.093573 |
| H | 2.584609 | 6.510027 | 0.082980 |
| F | 3.561813 | 4.350914 | -1.047296 |
| F | 0.123127 | 6.951324 | 0.837066 |
| C | -4.563782 | 1.503357 | -3.311594 |
| H | -3.617909 | 2.037777 | -3.440313 |
| H | -5.011648 | 1.361662 | -4.302814 |
| H | -5.226302 | 2.145889 | -2.723254 |
| C | -5.712947 | -0.566603 | -2.483070 |
| H | -6.418189 | 0.041972 | -1.903941 |
| H | -6.162698 | -0.729410 | -3.469188 |
| H | -5.622595 | -1.546708 | -1.999170 |
| C | -3.438512 | -0.723032 | -3.532442 |
| H | -2.452388 | -0.258873 | -3.647223 |
| H | -3.306885 | -1.731753 | -3.131878 |
| H | -3.879612 | -0.805717 | -4.532962 |

TS Conformation 67

B3LYP/6-31G(d) Energy = -3411.882419
M06-2X/def2tzvpp/IEFPCM(chloroform) Energy = -3411.890847
M06-2X/def2tzvpp/IEFPCM(chloroform)//B3LYP/6-31G(d) Quasiharmonic Free Energy = -3411.164348

Frequencies (Top 3 out of 291)

1. -670.2504 cm-1
2. 11.3173 cm-1
3. 13.5691 cm-1

B3LYP/6-31G(d) Molecular Geometry in Cartesian Coordinates

| | | | |
|---|---|---|---|
| C | -2.494606 | -1.508203 | -0.396361 |
| C | -2.913954 | -0.353020 | -1.330002 |
| H | -3.632992 | 0.301431 | -0.839548 |

| | | | |
|---|---|---|---|
| C | -1.507192 | 1.737061 | -1.165658 |
| N | -0.189307 | 2.099660 | -1.315627 |
| H | 0.428994 | 1.327143 | -1.545231 |
| N | -1.705832 | 0.440529 | -1.547671 |
| H | -0.882314 | -0.113151 | -1.765793 |
| C | -3.642945 | -0.427091 | 1.590977 |
| C | -2.443002 | -2.567089 | 1.795721 |
| C | -3.171867 | -0.655287 | 3.050101 |
| H | -3.265126 | 0.533036 | 1.230898 |
| C | -3.032623 | -2.179988 | 3.156791 |
| H | -2.740984 | -3.562169 | 1.450049 |
| H | -1.348990 | -2.518294 | 1.802936 |
| H | -3.868699 | -0.225761 | 3.774686 |
| H | -2.192706 | -0.181868 | 3.181066 |
| H | -4.013467 | -2.646258 | 3.305126 |
| H | -2.385019 | -2.488543 | 3.982459 |
| N | -2.958609 | -1.531397 | 0.873229 |
| O | -1.715899 | -2.385235 | -0.809188 |
| S | -2.751921 | 2.737300 | -0.618194 |
| C | -5.159388 | -0.438010 | 1.446788 |
| C | -5.892883 | -1.613895 | 1.244098 |
| C | -5.854625 | 0.773553 | 1.569026 |
| C | -7.287721 | -1.582067 | 1.180900 |
| H | -5.370443 | -2.557508 | 1.115884 |
| C | -7.247641 | 0.807424 | 1.506270 |
| C | -7.970057 | -0.372154 | 1.315178 |
| H | -7.840219 | -2.504732 | 1.021953 |
| O | 3.937788 | 0.316200 | -1.779654 |
| O | 1.111347 | -0.557871 | -2.394817 |
| C | 3.896758 | -0.374412 | -0.768101 |
| C | 2.964374 | -1.530123 | -0.564931 |
| O | 4.623703 | -0.160962 | 0.325193 |
| C | 5.454830 | 1.022139 | 0.335633 |
| H | 4.894133 | 1.891041 | -0.011366 |
| H | 5.750956 | 1.143065 | 1.376486 |
| H | 6.327823 | 0.867683 | -0.304762 |
| S | 1.998583 | -1.706079 | -2.078234 |
| C | 3.053745 | -2.051334 | -3.514468 |
| H | 3.674872 | -1.164998 | -3.644729 |
| H | 2.392993 | -2.205469 | -4.370837 |
| H | 3.653540 | -2.939856 | -3.305855 |
| C | 1.021765 | -3.203642 | -1.885297 |
| H | 1.663817 | -4.033298 | -1.590095 |
| H | 0.238323 | -2.989766 | -1.148105 |
| H | 0.550938 | -3.379927 | -2.855130 |
| C | 3.554954 | -2.827486 | -0.067613 |
| C | 4.790663 | -3.293976 | -0.549502 |
| C | 2.874416 | -3.583146 | 0.900288 |
| C | 5.328628 | -4.492526 | -0.083633 |
| H | 5.345170 | -2.704489 | -1.276509 |
| C | 3.413721 | -4.785386 | 1.360865 |
| H | 1.942316 | -3.201240 | 1.308117 |
| C | 4.636673 | -5.244568 | 0.868998 |
| H | 6.287951 | -4.836500 | -0.460256 |
| H | 2.879858 | -5.358090 | 2.114117 |
| H | 5.055168 | -6.179018 | 1.232596 |
| C | 2.943675 | -0.178470 | 3.288457 |
| C | 3.770317 | 0.607577 | 4.091982 |
| C | 3.583717 | 1.990865 | 4.161262 |
| C | 2.558053 | 2.576716 | 3.416067 |
| C | 1.734754 | 1.793697 | 2.605495 |
| C | 1.915373 | 0.401347 | 2.523669 |

| | | | |
|---|---|---|---|
| H | 3.092075 | -1.253781 | 3.248289 |
| H | 4.557873 | 0.134358 | 4.675016 |
| H | 4.223720 | 2.601778 | 4.792894 |
| H | 2.396968 | 3.651393 | 3.460643 |
| H | 0.942854 | 2.257969 | 2.025648 |
| S | 0.848492 | -0.587047 | 1.484085 |
| H | 2.045555 | -1.119836 | 0.300204 |
| H | -5.296170 | 1.696962 | 1.702353 |
| H | -7.767714 | 1.757457 | 1.596949 |
| H | -9.055255 | -0.347082 | 1.262191 |
| C | -3.571193 | -0.830390 | -2.673701 |
| C | 0.525321 | 3.279077 | -1.049163 |
| C | -0.042871 | 4.521450 | -0.731443 |
| C | 1.927033 | 3.158006 | -1.135867 |
| C | 0.809913 | 5.592309 | -0.492869 |
| H | -1.111833 | 4.651773 | -0.659261 |
| C | 2.715292 | 4.263873 | -0.875604 |
| H | 2.401458 | 2.218486 | -1.398314 |
| C | 2.195833 | 5.510305 | -0.547755 |
| H | 2.830428 | 6.364111 | -0.346210 |
| F | 0.256771 | 6.784104 | -0.185791 |
| F | 4.061985 | 4.119683 | -0.933030 |
| C | -4.143570 | 0.413025 | -3.383134 |
| H | -3.361213 | 1.149925 | -3.590886 |
| H | -4.600947 | 0.123927 | -4.337356 |
| H | -4.908860 | 0.905763 | -2.773873 |
| C | -4.721987 | -1.802780 | -2.350827 |
| H | -5.450177 | -1.354402 | -1.665908 |
| H | -5.252423 | -2.073604 | -3.271547 |
| H | -4.351028 | -2.728868 | -1.897195 |
| C | -2.568437 | -1.524107 | -3.616930 |
| H | -2.162729 | -2.432026 | -3.164867 |
| H | -3.072792 | -1.793386 | -4.553123 |
| H | -1.735724 | -0.858499 | -3.872943 |

TS Conformation 68

B3LYP/6-31G(d) Energy = -3411.881686
M06-2X/def2tzvpp/IEFPCM(chloroform) Energy = -3411.889546
M06-2X/def2tzvpp/IEFPCM(chloroform)//B3LYP/6-31G(d) Quasiharmonic Free Energy = -3411.1643

Frequencies (Top 3 out of 291)

1. -1019.3711 cm-1
2. 8.7276 cm-1
3. 14.7653 cm-1

B3LYP/6-31G(d) Molecular Geometry in Cartesian Coordinates

| | | | |
|---|---|---|---|
| C | -2.492070 | -1.583315 | 0.169853 |
| C | -2.756238 | -0.615127 | -1.005629 |
| H | -3.599622 | 0.037487 | -0.782514 |
| C | -1.559038 | 1.605308 | -0.919176 |
| N | -0.269164 | 2.067792 | -0.696102 |
| H | 0.319465 | 1.440586 | -0.141150 |
| N | -1.577556 | 0.254411 | -1.069605 |
| H | -0.681873 | -0.217533 | -1.162361 |
| C | -3.919336 | -0.161727 | 1.732538 |
| C | -2.706010 | -2.149208 | 2.529324 |
| C | -3.675710 | -0.073845 | 3.261542 |
| H | -3.529332 | 0.736396 | 1.246019 |

| | | | |
|---|---|---|---|
| C | -3.501579 | -1.537934 | 3.689172 |
| H | -2.928985 | -3.205518 | 2.350890 |
| H | -1.622618 | -2.063918 | 2.676535 |
| H | -4.491894 | 0.439946 | 3.776059 |
| H | -2.749900 | 0.484701 | 3.443320 |
| H | -4.479366 | -2.023568 | 3.784104 |
| H | -2.981307 | -1.643943 | 4.645469 |
| N | -3.099057 | -1.335911 | 1.360104 |
| O | -1.703645 | -2.528115 | 0.049898 |
| S | -2.929914 | 2.583525 | -0.954145 |
| C | -5.395494 | -0.297228 | 1.375182 |
| C | -6.156595 | 0.868196 | 1.205537 |
| C | -6.031400 | -1.538294 | 1.249320 |
| C | -7.522221 | 0.795409 | 0.932643 |
| H | -5.669979 | 1.838216 | 1.275231 |
| C | -7.399215 | -1.613092 | 0.975749 |
| C | -8.149634 | -0.447192 | 0.819893 |
| H | -8.093905 | 1.709815 | 0.797505 |
| O | 5.491404 | -0.738205 | -0.876619 |
| O | 1.361443 | -0.753519 | -2.219030 |
| C | 4.742630 | -1.521238 | -0.295398 |
| C | 3.294902 | -1.677187 | -0.553815 |
| O | 5.156987 | -2.312831 | 0.709615 |
| C | 6.537317 | -2.162458 | 1.091918 |
| H | 6.712544 | -1.158400 | 1.486371 |
| H | 6.702500 | -2.913687 | 1.863969 |
| H | 7.194868 | -2.334638 | 0.236515 |
| S | 2.819186 | -0.673921 | -1.966513 |
| C | 3.308581 | 1.039109 | -1.674163 |
| H | 2.906091 | 1.304994 | -0.694229 |
| H | 4.394138 | 1.111222 | -1.686658 |
| H | 2.825034 | 1.627866 | -2.457104 |
| C | 3.721993 | -1.160895 | -3.459599 |
| H | 4.789394 | -1.064194 | -3.255184 |
| H | 3.443059 | -2.198112 | -3.657845 |
| H | 3.395810 | -0.512096 | -4.275920 |
| C | 2.717005 | -3.064097 | -0.667885 |
| C | 1.376127 | -3.305304 | -0.334638 |
| C | 3.498375 | -4.136216 | -1.140296 |
| C | 0.832126 | -4.583942 | -0.463382 |
| H | 0.737796 | -2.509578 | 0.026729 |
| C | 2.957812 | -5.414618 | -1.253536 |
| H | 4.541652 | -3.973992 | -1.399171 |
| C | 1.619791 | -5.640903 | -0.917836 |
| H | -0.211096 | -4.729982 | -0.202721 |
| H | 3.579398 | -6.232543 | -1.608280 |
| H | 1.197266 | -6.637847 | -1.011817 |
| C | 3.826212 | -0.061160 | 3.026925 |
| C | 4.689538 | 0.528944 | 3.950884 |
| C | 4.391127 | 1.772331 | 4.511735 |
| C | 3.210088 | 2.418199 | 4.142669 |
| C | 2.345327 | 1.836730 | 3.214866 |
| C | 2.645294 | 0.592237 | 2.634496 |
| H | 4.055613 | -1.040685 | 2.619596 |
| H | 5.597107 | 0.005276 | 4.243345 |
| H | 5.064844 | 2.227199 | 5.232587 |
| H | 2.956643 | 3.382262 | 4.576567 |
| H | 1.422633 | 2.343656 | 2.946506 |
| S | 1.508400 | -0.148446 | 1.472695 |
| H | 2.541943 | -0.969295 | 0.471274 |
| H | -5.451576 | -2.451318 | 1.348547 |
| H | -7.876036 | -2.585303 | 0.880300 |

| | | | |
|---|---|---|---|
| H | -9.212881 | -0.505675 | 0.602837 |
| C | -3.081006 | -1.328390 | -2.363516 |
| C | 0.232074 | 3.370389 | -0.837359 |
| C | -0.235796 | 4.257078 | -1.820140 |
| C | 1.309913 | 3.748053 | -0.016312 |
| C | 0.371967 | 5.498364 | -1.934835 |
| H | -1.061182 | 4.000733 | -2.468790 |
| C | 1.881937 | 5.001115 | -0.191403 |
| H | 1.684583 | 3.094740 | 0.763912 |
| C | 1.438800 | 5.910656 | -1.142435 |
| H | 1.892387 | 6.887159 | -1.256931 |
| F | -0.081755 | 6.346222 | -2.880583 |
| F | 2.913690 | 5.351868 | 0.604262 |
| C | -1.863473 | -2.049439 | -2.973970 |
| H | -1.467241 | -2.810686 | -2.299906 |
| H | -2.161770 | -2.531614 | -3.913394 |
| H | -1.056263 | -1.347494 | -3.210682 |
| C | -3.575169 | -0.258789 | -3.358284 |
| H | -2.817678 | 0.513533 | -3.525768 |
| H | -3.802465 | -0.726174 | -4.324344 |
| H | -4.481073 | 0.240992 | -2.998837 |
| C | -4.212718 | -2.346958 | -2.119586 |
| H | -5.086482 | -1.876596 | -1.652944 |
| H | -4.537384 | -2.778686 | -3.073682 |
| H | -3.879516 | -3.169097 | -1.477661 |

TS Conformation 69

B3LYP/6-31G(d) Energy = -3411.892584
M06-2X/def2tzvpp/IEFPCM(chloroform) Energy = -3411.89092
M06-2X/def2tzvpp/IEFPCM(chloroform)//B3LYP/6-31G(d) Quasiharmonic Free Energy = -3411.164256

Frequencies (Top 3 out of 291)

1. -983.7953 cm-1
2. 8.6335 cm-1
3. 10.8670 cm-1

B3LYP/6-31G(d) Molecular Geometry in Cartesian Coordinates

| | | | |
|---|---|---|---|
| C | -2.916443 | -1.631980 | -0.281857 |
| C | -3.241423 | -0.499763 | -1.277736 |
| H | -4.160832 | 0.014121 | -1.000540 |
| C | -2.258694 | 1.768607 | -0.722846 |
| N | -1.014476 | 2.306931 | -0.496797 |
| H | -0.256822 | 1.631510 | -0.539052 |
| N | -2.152576 | 0.463714 | -1.123766 |
| H | -1.241697 | 0.027697 | -1.011123 |
| C | -4.795173 | -0.900748 | 1.272817 |
| C | -3.321106 | -2.796394 | 1.821281 |
| C | -4.776392 | -1.153939 | 2.802663 |
| H | -4.518052 | 0.137101 | 1.068656 |
| C | -4.367503 | -2.628164 | 2.929420 |
| H | -3.293099 | -3.800288 | 1.387031 |
| H | -2.308653 | -2.565852 | 2.176081 |
| H | -5.740434 | -0.925990 | 3.263926 |
| H | -4.017556 | -0.509865 | 3.263077 |
| H | -5.229417 | -3.278971 | 2.747334 |
| H | -3.965541 | -2.877029 | 3.915787 |
| N | -3.714949 | -1.804857 | 0.800456 |
| O | -1.904679 | -2.333950 | -0.445834 |

| | | | |
|---|---|---|---|
| S | -3.733318 | 2.570409 | -0.560034 |
| C | -6.156676 | -1.168951 | 0.644974 |
| C | -6.572302 | -2.449707 | 0.258717 |
| C | -7.048969 | -0.098337 | 0.494401 |
| C | -7.855378 | -2.657748 | -0.250937 |
| H | -5.883932 | -3.286548 | 0.334372 |
| C | -8.331311 | -0.304478 | -0.014980 |
| C | -8.740155 | -1.586706 | -0.386041 |
| H | -8.160528 | -3.657708 | -0.548009 |
| O | 3.149541 | 0.950704 | -1.092518 |
| O | 0.465369 | -0.128598 | 0.189200 |
| C | 3.624319 | 0.510874 | -0.047755 |
| C | 3.179760 | -0.749549 | 0.607317 |
| O | 4.635384 | 1.082812 | 0.610560 |
| C | 5.196404 | 2.265892 | 0.003430 |
| H | 5.571114 | 2.031934 | -0.995339 |
| H | 6.015097 | 2.557374 | 0.660818 |
| H | 4.446437 | 3.058357 | -0.053399 |
| S | 1.552907 | -1.133511 | -0.017035 |
| C | 1.678959 | -1.584710 | -1.758000 |
| H | 2.515201 | -2.287309 | -1.859686 |
| H | 1.876759 | -0.659585 | -2.297648 |
| H | 0.716931 | -2.026354 | -2.028887 |
| C | 1.100345 | -2.683985 | 0.774775 |
| H | 1.823319 | -3.450188 | 0.482658 |
| H | 0.084182 | -2.902688 | 0.431054 |
| H | 1.125986 | -2.518935 | 1.852538 |
| C | 3.242842 | -0.831351 | 2.115860 |
| C | 3.991395 | -1.842150 | 2.736719 |
| C | 2.565993 | 0.105765 | 2.916543 |
| C | 4.062257 | -1.910502 | 4.129427 |
| H | 4.528208 | -2.560381 | 2.123786 |
| C | 2.633935 | 0.029851 | 4.306605 |
| H | 1.987974 | 0.896676 | 2.446368 |
| C | 3.383389 | -0.979356 | 4.916254 |
| H | 4.652747 | -2.693169 | 4.597946 |
| H | 2.106919 | 0.761816 | 4.912486 |
| H | 3.440730 | -1.035358 | 6.000065 |
| C | 7.473477 | -2.613251 | -0.956221 |
| C | 8.573446 | -1.934469 | -1.480578 |
| C | 8.392947 | -0.834727 | -2.321996 |
| C | 7.096055 | -0.426837 | -2.639468 |
| C | 5.992326 | -1.110640 | -2.126098 |
| C | 6.163842 | -2.212303 | -1.269891 |
| H | 7.620633 | -3.462458 | -0.295491 |
| H | 9.577417 | -2.265419 | -1.225178 |
| H | 9.250974 | -0.305674 | -2.728021 |
| H | 6.937937 | 0.420364 | -3.303327 |
| H | 4.991237 | -0.788012 | -2.396423 |
| S | 4.778398 | -3.122236 | -0.600187 |
| H | 3.939712 | -1.813714 | 0.047778 |
| H | -6.730415 | 0.905023 | 0.767132 |
| H | -9.006544 | 0.539011 | -0.130827 |
| H | -9.737086 | -1.748534 | -0.786791 |
| C | -3.417510 | -0.985161 | -2.761244 |
| C | -0.541871 | 3.593781 | -0.190012 |
| C | -1.339239 | 4.705361 | 0.113643 |
| C | 0.863052 | 3.718590 | -0.191253 |
| C | -0.702911 | 5.905931 | 0.407545 |
| H | -2.417073 | 4.642478 | 0.121902 |
| C | 1.428312 | 4.943043 | 0.116283 |
| H | 1.506189 | 2.883506 | -0.452369 |

| | | | |
|---|---|---|---|
| C | 0.676748 | 6.070864 | 0.424903 |
| H | 1.137365 | 7.021960 | 0.660728 |
| F | -1.472878 | 6.972876 | 0.701248 |
| F | 2.778429 | 5.047254 | 0.112951 |
| C | -2.099727 | -1.478878 | -3.388558 |
| H | -1.704331 | -2.347788 | -2.856821 |
| H | -2.274602 | -1.762903 | -4.433014 |
| H | -1.340387 | -0.687854 | -3.390696 |
| C | -3.945783 | 0.206152 | -3.584879 |
| H | -3.253513 | 1.053421 | -3.553485 |
| H | -4.071924 | -0.090332 | -4.633111 |
| H | -4.914166 | 0.555289 | -3.210740 |
| C | -4.454565 | -2.123879 | -2.796897 |
| H | -4.097705 | -3.013059 | -2.264338 |
| H | -5.407848 | -1.818143 | -2.352555 |
| H | -4.649602 | -2.417825 | -3.834817 |

TS Conformation 70

B3LYP/6-31G(d) Energy = -3411.887188
M06-2X/def2tzvpp/IEFPCM(chloroform) Energy = -3411.890359
M06-2X/def2tzvpp/IEFPCM(chloroform)//B3LYP/6-31G(d) Quasiharmonic Free Energy = -3411.164233

Frequencies (Top 3 out of 291)

1. -994.4659 cm-1
2. 5.8633 cm-1
3. 10.4370 cm-1

B3LYP/6-31G(d) Molecular Geometry in Cartesian Coordinates

| | | | |
|---|---|---|---|
| C | 3.112079 | -1.502147 | 0.509514 |
| C | 3.179669 | -0.104340 | 1.155789 |
| H | 3.997487 | 0.479586 | 0.736923 |
| C | 1.868141 | 1.695808 | -0.022668 |
| N | 0.574336 | 1.961366 | -0.403937 |
| H | -0.104582 | 1.215776 | -0.260252 |
| N | 1.948983 | 0.581283 | 0.757932 |
| H | 1.092351 | 0.063635 | 0.930134 |
| C | 4.914854 | -0.899932 | -1.191011 |
| C | 3.822820 | -3.107104 | -1.176178 |
| C | 4.988322 | -1.552926 | -2.595750 |
| H | 4.459253 | 0.090966 | -1.270511 |
| C | 4.852266 | -3.055584 | -2.311996 |
| H | 3.972114 | -3.940202 | -0.482590 |
| H | 2.794045 | -3.180223 | -1.549308 |
| H | 5.909585 | -1.287778 | -3.120637 |
| H | 4.140666 | -1.202641 | -3.196974 |
| H | 5.812085 | -3.467629 | -1.981772 |
| H | 4.528668 | -3.627679 | -3.186447 |
| N | 3.993529 | -1.817073 | -0.476341 |
| O | 2.237960 | -2.307949 | 0.853792 |
| S | 3.203305 | 2.620952 | -0.480963 |
| C | 6.283198 | -0.749694 | -0.538231 |
| C | 6.984815 | 0.450716 | -0.717487 |
| C | 6.890245 | -1.779384 | 0.192072 |
| C | 8.267621 | 0.613187 | -0.193812 |
| H | 6.515644 | 1.266833 | -1.262021 |
| C | 8.174311 | -1.618762 | 0.716541 |
| C | 8.868615 | -0.423635 | 0.522448 |
| H | 8.792911 | 1.553627 | -0.337684 |

| | | | |
|---|---|---|---|
| O | -1.551348 | -0.218585 | 0.306293 |
| O | -2.655393 | 0.038198 | -2.761727 |
| C | -2.258612 | -1.227712 | 0.315497 |
| C | -3.490191 | -1.440286 | -0.491620 |
| O | -2.046097 | -2.276886 | 1.112289 |
| C | -0.916202 | -2.207112 | 2.008340 |
| H | 0.022551 | -2.234693 | 1.450935 |
| H | -0.975011 | -1.299832 | 2.614257 |
| H | -1.004900 | -3.091626 | 2.639028 |
| S | -3.678181 | -0.146310 | -1.699570 |
| C | -5.289960 | -0.488560 | -2.432842 |
| H | -5.212322 | -1.434006 | -2.970600 |
| H | -6.029345 | -0.542124 | -1.627775 |
| H | -5.494228 | 0.334093 | -3.121578 |
| C | -3.975401 | 1.386682 | -0.790655 |
| H | -4.077937 | 2.173079 | -1.542132 |
| H | -3.104421 | 1.558741 | -0.160724 |
| H | -4.876755 | 1.247650 | -0.188012 |
| C | -3.679526 | -2.820523 | -1.091867 |
| C | -4.610122 | -3.704988 | -0.526806 |
| C | -2.913163 | -3.248085 | -2.190618 |
| C | -4.772532 | -4.986978 | -1.053760 |
| H | -5.194945 | -3.391077 | 0.331701 |
| C | -3.090443 | -4.525371 | -2.722614 |
| H | -2.178604 | -2.578596 | -2.629289 |
| C | -4.019808 | -5.398686 | -2.154408 |
| H | -5.492582 | -5.663549 | -0.601607 |
| H | -2.493390 | -4.840145 | -3.574207 |
| H | -4.152093 | -6.396303 | -2.564481 |
| C | -5.910939 | 1.425345 | 2.665063 |
| C | -5.372934 | 2.253247 | 3.651377 |
| C | -4.271674 | 1.836755 | 4.401884 |
| C | -3.721166 | 0.575725 | 4.163060 |
| C | -4.262248 | -0.259941 | 3.185236 |
| C | -5.361831 | 0.155854 | 2.413530 |
| H | -6.768866 | 1.755151 | 2.085425 |
| H | -5.816525 | 3.229665 | 3.831482 |
| H | -3.851710 | 2.483743 | 5.167162 |
| H | -2.871323 | 0.234092 | 4.749725 |
| H | -3.844541 | -1.248951 | 3.020635 |
| S | -6.066802 | -0.904443 | 1.159231 |
| H | -4.634627 | -1.199645 | 0.329861 |
| H | 6.349277 | -2.704040 | 0.371444 |
| H | 8.629206 | -2.427671 | 1.282411 |
| H | 9.866231 | -0.297702 | 0.934441 |
| C | 3.386249 | -0.126674 | 2.711799 |
| C | 0.037018 | 3.106948 | -1.025744 |
| C | 0.470810 | 4.409542 | -0.744374 |
| C | -1.051668 | 2.899368 | -1.886036 |
| C | -0.203582 | 5.468514 | -1.336584 |
| H | 1.307337 | 4.601126 | -0.087945 |
| C | -1.705914 | 4.002577 | -2.412618 |
| H | -1.371698 | 1.900144 | -2.158550 |
| C | -1.308971 | 5.309471 | -2.167743 |
| H | -1.826866 | 6.158338 | -2.596548 |
| F | 0.211161 | 6.721838 | -1.065876 |
| F | -2.795643 | 3.787174 | -3.191131 |
| C | 3.664195 | 1.318781 | 3.170555 |
| H | 2.832333 | 1.985152 | 2.920394 |
| H | 3.804463 | 1.346376 | 4.257852 |
| H | 4.566871 | 1.723335 | 2.700129 |
| C | 4.609407 | -1.004686 | 3.038753 |

| | | | |
|---|---|---|---|
| H | 5.505392 | -0.666350 | 2.507362 |
| H | 4.821930 | -0.961178 | 4.113362 |
| H | 4.432939 | -2.054700 | 2.779219 |
| C | 2.160149 | -0.665929 | 3.472565 |
| H | 1.273933 | -0.044505 | 3.295742 |
| H | 1.931352 | -1.694505 | 3.184960 |
| H | 2.359537 | -0.642164 | 4.550588 |

TS Conformation 71

B3LYP/6-31G(d) Energy = -3411.89169
M06-2X/def2tzvpp/IEFPCM(chloroform) Energy = -3411.890475
M06-2X/def2tzvpp/IEFPCM(chloroform)//B3LYP/6-31G(d) Quasiharmonic Free Energy = -3411.164163

Frequencies (Top 3 out of 291)

1. -998.0998 cm-1
2. 7.9904 cm-1
3. 11.8592 cm-1

B3LYP/6-31G(d) Molecular Geometry in Cartesian Coordinates

| | | | |
|---|---|---|---|
| C | -2.361293 | -1.612066 | -0.087721 |
| C | -2.944448 | -0.589931 | -1.084709 |
| H | -3.902412 | -0.206075 | -0.736703 |
| C | -2.276786 | 1.823204 | -0.704848 |
| N | -1.117388 | 2.557042 | -0.628753 |
| H | -0.269968 | 2.007407 | -0.738927 |
| N | -2.006770 | 0.531727 | -1.070299 |
| H | -1.032590 | 0.244313 | -1.033383 |
| C | -4.186804 | -1.103004 | 1.612552 |
| C | -2.394366 | -2.726476 | 2.080241 |
| C | -3.994873 | -1.280026 | 3.141171 |
| H | -4.092453 | -0.045674 | 1.350327 |
| C | -3.352400 | -2.667723 | 3.276093 |
| H | -2.249113 | -3.733481 | 1.677794 |
| H | -1.402528 | -2.329391 | 2.330644 |
| H | -4.938412 | -1.180020 | 3.683262 |
| H | -3.307958 | -0.507727 | 3.507776 |
| H | -4.115451 | -3.449459 | 3.197195 |
| H | -2.831324 | -2.806916 | 4.227715 |
| N | -3.025534 | -1.854205 | 1.069817 |
| O | -1.271261 | -2.159198 | -0.322959 |
| S | -3.836454 | 2.396777 | -0.415404 |
| C | -5.540414 | -1.601992 | 1.124169 |
| C | -5.787866 | -2.948426 | 0.826940 |
| C | -6.594962 | -0.684737 | 1.016450 |
| C | -7.063280 | -3.370329 | 0.446798 |
| H | -4.976224 | -3.669056 | 0.869836 |
| C | -7.869973 | -1.104648 | 0.636289 |
| C | -8.109182 | -2.450680 | 0.353750 |
| H | -7.236991 | -4.418428 | 0.217415 |
| O | 3.023053 | 1.810766 | -1.643422 |
| O | 0.787803 | 0.402072 | -0.027656 |
| C | 3.726704 | 1.497951 | -0.688319 |
| C | 3.595087 | 0.223239 | 0.073296 |
| O | 4.745174 | 2.238740 | -0.240091 |
| C | 4.977980 | 3.478377 | -0.942550 |
| H | 4.135711 | 4.159864 | -0.799895 |
| H | 5.883653 | 3.889281 | -0.497200 |
| H | 5.121166 | 3.290033 | -2.008683 |

| | | | |
|---|---|---|---|
| S | 1.987332 | -0.450867 | -0.296157 |
| C | 1.989728 | -1.003314 | -2.012229 |
| H | 1.088241 | -1.607060 | -2.139153 |
| H | 2.910993 | -1.566945 | -2.189408 |
| H | 1.970868 | -0.097659 | -2.617452 |
| C | 1.849261 | -1.973660 | 0.651005 |
| H | 2.646302 | -2.659396 | 0.353510 |
| H | 0.847454 | -2.356125 | 0.429969 |
| H | 1.943130 | -1.702731 | 1.703614 |
| C | 3.912925 | 0.253334 | 1.551595 |
| C | 5.123240 | -0.291393 | 2.004472 |
| C | 3.049308 | 0.859212 | 2.480999 |
| C | 5.457277 | -0.238937 | 3.358542 |
| H | 5.804038 | -0.738912 | 1.286928 |
| C | 3.378882 | 0.893073 | 3.836097 |
| H | 2.120234 | 1.309463 | 2.141448 |
| C | 4.584600 | 0.344682 | 4.278118 |
| H | 6.402562 | -0.657230 | 3.693674 |
| H | 2.699569 | 1.361902 | 4.542886 |
| H | 4.845307 | 0.380130 | 5.332465 |
| C | 5.026632 | -3.802653 | 0.269236 |
| C | 4.570324 | -5.092310 | 0.550121 |
| C | 4.074854 | -5.907489 | -0.470156 |
| C | 4.043357 | -5.417205 | -1.777304 |
| C | 4.487746 | -4.124729 | -2.059442 |
| C | 4.983704 | -3.287557 | -1.041411 |
| H | 5.431409 | -3.188147 | 1.068056 |
| H | 4.615515 | -5.465140 | 1.570967 |
| H | 3.726870 | -6.913197 | -0.250739 |
| H | 3.669672 | -6.042937 | -2.584447 |
| H | 4.466233 | -3.752628 | -3.080093 |
| S | 5.568003 | -1.643958 | -1.436215 |
| H | 4.479679 | -0.685689 | -0.576384 |
| H | -6.410589 | 0.367466 | 1.220042 |
| H | -8.673182 | -0.377650 | 0.551588 |
| H | -9.100560 | -2.778957 | 0.053614 |
| C | -3.179821 | -1.166585 | -2.526758 |
| C | -0.827614 | 3.913925 | -0.407030 |
| C | 0.531950 | 4.255793 | -0.560035 |
| C | -1.757068 | 4.899537 | -0.048512 |
| C | 0.923086 | 5.564680 | -0.344137 |
| H | 1.269663 | 3.519822 | -0.864995 |
| C | -1.293636 | 6.195598 | 0.146512 |
| H | -2.804894 | 4.670455 | 0.074851 |
| C | 0.036859 | 6.573822 | 0.013978 |
| H | 0.361814 | 7.593809 | 0.176436 |
| F | 2.232828 | 5.877424 | -0.491482 |
| F | -2.190244 | 7.140974 | 0.493269 |
| C | -1.865751 | -1.492333 | -3.261969 |
| H | -1.299120 | -2.269480 | -2.743284 |
| H | -2.090453 | -1.846403 | -4.275071 |
| H | -1.235523 | -0.600758 | -3.362637 |
| C | -3.953656 | -0.106299 | -3.335244 |
| H | -3.395282 | 0.832751 | -3.402300 |
| H | -4.130803 | -0.469103 | -4.354855 |
| H | -4.924291 | 0.116931 | -2.879536 |
| C | -4.033183 | -2.444994 | -2.422598 |
| H | -3.500528 | -3.245190 | -1.895705 |
| H | -4.977796 | -2.260592 | -1.899811 |
| H | -4.274062 | -2.814877 | -3.426052 |

TS Conformation 72

B3LYP/6-31G(d) Energy = -3411.884103
M06-2X/def2tzvpp/IEFPCM(chloroform) Energy = -3411.891132
M06-2X/def2tzvpp/IEFPCM(chloroform)//B3LYP/6-31G(d) Quasiharmonic Free Energy = -3411.164098

Frequencies (Top 3 out of 291)

1. -867.3602 cm-1
2. 10.5625 cm-1
3. 12.0052 cm-1

B3LYP/6-31G(d) Molecular Geometry in Cartesian Coordinates

| | | | |
|---|---|---|---|
| C | 3.491401 | -1.623995 | 0.423109 |
| C | 4.129632 | -0.285624 | 0.851189 |
| H | 4.895260 | 0.030887 | 0.142077 |
| C | 3.109987 | 1.853744 | -0.000652 |
| N | 1.880562 | 2.459306 | -0.042981 |
| H | 1.132191 | 1.926447 | 0.391610 |
| N | 3.063554 | 0.706243 | 0.756121 |
| H | 2.139776 | 0.336561 | 0.963811 |
| C | 3.441704 | -3.587652 | -1.066100 |
| C | 5.323948 | -2.028730 | -1.287281 |
| C | 4.465825 | -4.106175 | -2.110455 |
| H | 3.401181 | -4.263642 | -0.203050 |
| C | 5.784002 | -3.418504 | -1.738217 |
| H | 5.167352 | -1.359555 | -2.143030 |
| H | 6.033194 | -1.553516 | -0.611021 |
| H | 4.148327 | -3.795124 | -3.111784 |
| H | 4.522660 | -5.198317 | -2.107112 |
| H | 6.494628 | -3.371008 | -2.568491 |
| H | 6.270812 | -3.936791 | -0.903324 |
| N | 4.040333 | -2.308098 | -0.610240 |
| O | 2.464219 | -2.028681 | 0.993261 |
| S | 4.518982 | 2.413557 | -0.736347 |
| C | 2.043359 | -3.474604 | -1.651201 |
| C | 1.601400 | -2.322469 | -2.313247 |
| C | 1.199876 | -4.591594 | -1.604309 |
| C | 0.340233 | -2.285786 | -2.909341 |
| H | 2.236846 | -1.441683 | -2.345909 |
| C | -0.056126 | -4.563453 | -2.214731 |
| C | -0.490772 | -3.408340 | -2.867549 |
| H | -0.006163 | -1.376680 | -3.391364 |
| O | -1.488068 | 1.924475 | 2.142312 |
| O | 0.099899 | 0.149346 | 0.446597 |
| C | -2.481035 | 1.328169 | 1.738502 |
| C | -2.505629 | -0.088486 | 1.271941 |
| O | -3.686564 | 1.895042 | 1.609362 |
| C | -3.761679 | 3.302088 | 1.926640 |
| H | -3.155412 | 3.882426 | 1.227245 |
| H | -4.815551 | 3.558711 | 1.824412 |
| H | -3.415401 | 3.481431 | 2.947254 |
| S | -0.807610 | -0.681415 | 1.276840 |
| C | -0.129115 | -0.867994 | 2.945796 |
| H | -0.066831 | 0.139984 | 3.358223 |
| H | -0.800863 | -1.497170 | 3.533498 |
| H | 0.857845 | -1.321447 | 2.821895 |
| C | -0.856589 | -2.356645 | 0.635594 |
| H | -1.438518 | -2.992694 | 1.303698 |
| H | -1.317015 | -2.290956 | -0.354787 |
| H | 0.190208 | -2.665671 | 0.563865 |

| | | | |
|---|---|---|---|
| C | -3.473609 | -1.028743 | 1.949439 |
| C | -3.719002 | -0.933323 | 3.331311 |
| C | -4.152096 | -2.009502 | 1.208959 |
| C | -4.614769 | -1.798195 | 3.957678 |
| H | -3.217503 | -0.165143 | 3.915988 |
| C | -5.044029 | -2.877808 | 1.839830 |
| H | -3.996796 | -2.069732 | 0.136641 |
| C | -5.275612 | -2.777577 | 3.212417 |
| H | -4.798167 | -1.705775 | 5.024687 |
| H | -5.570143 | -3.623632 | 1.250772 |
| H | -5.975119 | -3.451968 | 3.698777 |
| C | -5.223484 | -1.187099 | -3.022102 |
| C | -6.599390 | -1.297772 | -3.219738 |
| C | -7.492616 | -0.617637 | -2.388575 |
| C | -6.986831 | 0.182030 | -1.361652 |
| C | -5.610736 | 0.305713 | -1.167214 |
| C | -4.700915 | -0.382053 | -1.992350 |
| H | -4.536384 | -1.724153 | -3.670418 |
| H | -6.975178 | -1.923450 | -4.026567 |
| H | -8.565036 | -0.706780 | -2.541263 |
| H | -7.668313 | 0.722330 | -0.707803 |
| H | -5.232173 | 0.942703 | -0.373367 |
| S | -2.936414 | -0.235927 | -1.788814 |
| H | -2.754116 | -0.085951 | -0.072316 |
| H | 1.529881 | -5.491757 | -1.088797 |
| H | -0.695755 | -5.441576 | -2.173001 |
| H | -1.475168 | -3.368214 | -3.323787 |
| C | 4.798977 | -0.344001 | 2.274145 |
| C | 1.419309 | 3.694360 | -0.535410 |
| C | 2.130567 | 4.550026 | -1.386877 |
| C | 0.118737 | 4.041059 | -0.120015 |
| C | 1.513711 | 5.728015 | -1.792742 |
| H | 3.127781 | 4.312620 | -1.725227 |
| C | -0.436183 | 5.224990 | -0.568949 |
| H | -0.444702 | 3.405897 | 0.555461 |
| C | 0.231993 | 6.105591 | -1.411557 |
| H | -0.218304 | 7.029375 | -1.752511 |
| F | 2.199882 | 6.550059 | -2.613127 |
| F | -1.687767 | 5.543834 | -0.158960 |
| C | 5.480557 | 1.010485 | 2.548781 |
| H | 4.754756 | 1.828968 | 2.550015 |
| H | 5.969892 | 0.988350 | 3.530253 |
| H | 6.236429 | 1.243773 | 1.792404 |
| C | 5.869829 | -1.453380 | 2.284652 |
| H | 6.656964 | -1.262123 | 1.545094 |
| H | 6.354740 | -1.497122 | 3.266575 |
| H | 5.442103 | -2.443804 | 2.087052 |
| C | 3.777995 | -0.620470 | 3.393323 |
| H | 3.313069 | -1.604149 | 3.285975 |
| H | 4.279978 | -0.582457 | 4.367502 |
| H | 2.990367 | 0.141526 | 3.401247 |

TS Conformation 73

B3LYP/6-31G(d) Energy = -3411.883314
M06-2X/def2tzvpp/IEFPCM(chloroform) Energy = -3411.888721
M06-2X/def2tzvpp/IEFPCM(chloroform)//B3LYP/6-31G(d) Quasiharmonic Free Energy = -3411.163936

Frequencies (Top 3 out of 291)

1. -587.6275 cm-1

2. 5.7026 cm-1
3. 10.3614 cm-1

B3LYP/6-31G(d) Molecular Geometry in Cartesian Coordinates

| | | | |
|---|---|---|---|
| C | -2.537109 | -1.225991 | -1.012100 |
| C | -3.074730 | 0.193659 | -1.283020 |
| H | -3.807170 | 0.486659 | -0.532430 |
| C | -1.822910 | 2.126709 | -0.240790 |
| N | -0.550150 | 2.655880 | -0.238670 |
| H | 0.140210 | 2.099370 | -0.733550 |
| N | -1.923510 | 1.082809 | -1.113900 |
| H | -1.072940 | 0.751500 | -1.560960 |
| C | -3.645149 | -1.295711 | 1.273350 |
| C | -2.276549 | -3.169481 | 0.439670 |
| C | -3.103709 | -2.145561 | 2.452400 |
| H | -3.361050 | -0.248981 | 1.412710 |
| C | -2.833209 | -3.520161 | 1.824420 |
| H | -2.523918 | -3.903891 | -0.333040 |
| H | -1.186259 | -3.055520 | 0.453800 |
| H | -3.806819 | -2.174641 | 3.288860 |
| H | -2.165599 | -1.703571 | 2.808320 |
| H | -3.765768 | -4.089221 | 1.738910 |
| H | -2.126738 | -4.120711 | 2.404320 |
| N | -2.904699 | -1.865651 | 0.122120 |
| O | -1.738879 | -1.741840 | -1.810050 |
| S | -3.111000 | 2.666259 | 0.704890 |
| C | -5.161169 | -1.368072 | 1.137830 |
| C | -5.807709 | -2.383172 | 0.421130 |
| C | -5.942819 | -0.407952 | 1.795630 |
| C | -7.202049 | -2.445792 | 0.376210 |
| H | -5.219269 | -3.118422 | -0.119990 |
| C | -7.335769 | -0.468812 | 1.751690 |
| C | -7.970699 | -1.491122 | 1.043780 |
| H | -7.686779 | -3.240242 | -0.185450 |
| O | 4.074911 | -2.419559 | -3.080940 |
| O | 1.144400 | 0.505790 | -1.729760 |
| C | 4.283141 | -1.935939 | -1.971610 |
| C | 3.333081 | -1.062319 | -1.231420 |
| O | 5.362671 | -2.217948 | -1.233040 |
| C | 6.264641 | -3.196938 | -1.787930 |
| H | 5.749482 | -4.151118 | -1.918480 |
| H | 6.652251 | -2.856698 | -2.751160 |
| H | 7.067861 | -3.288838 | -1.057840 |
| S | 2.003631 | -0.535419 | -2.351370 |
| C | 2.655640 | 0.119921 | -3.910200 |
| H | 3.240980 | 1.004471 | -3.649620 |
| H | 3.270231 | -0.649059 | -4.377780 |
| H | 1.795720 | 0.398181 | -4.524510 |
| C | 1.050321 | -1.989720 | -2.810830 |
| H | 0.319231 | -1.672400 | -3.556060 |
| H | 1.749921 | -2.736439 | -3.186080 |
| H | 0.519991 | -2.313670 | -1.912040 |
| C | 3.854210 | 0.097281 | -0.429200 |
| C | 4.911000 | 0.896262 | -0.898750 |
| C | 3.272510 | 0.394551 | 0.811560 |
| C | 5.377320 | 1.968652 | -0.141720 |
| H | 5.385570 | 0.663142 | -1.849380 |
| C | 3.744070 | 1.467721 | 1.568330 |
| H | 2.465730 | -0.227479 | 1.184560 |
| C | 4.790770 | 2.258992 | 1.092990 |
| H | 6.195850 | 2.578262 | -0.514110 |

| | | | |
|---|---|---|---|
| H | 3.289980 | 1.684481 | 2.530560 |
| H | 5.143709 | 3.103532 | 1.676550 |
| C | 1.803801 | -3.061389 | 3.299790 |
| C | 2.397501 | -3.006509 | 4.560540 |
| C | 3.781351 | -2.867469 | 4.687460 |
| C | 4.563011 | -2.790769 | 3.532770 |
| C | 3.974341 | -2.856949 | 2.269830 |
| C | 2.580101 | -2.989769 | 2.128190 |
| H | 0.725491 | -3.161140 | 3.210640 |
| H | 1.772741 | -3.066809 | 5.449080 |
| H | 4.243021 | -2.820439 | 5.670230 |
| H | 5.642711 | -2.683488 | 3.613320 |
| H | 4.596221 | -2.805729 | 1.380790 |
| S | 1.799431 | -3.099009 | 0.530160 |
| H | 2.694571 | -1.899759 | -0.436330 |
| H | -5.453740 | 0.399058 | 2.336140 |
| H | -7.924290 | 0.288448 | 2.262920 |
| H | -9.055709 | -1.538173 | 1.005370 |
| C | -3.763290 | 0.354799 | -2.685810 |
| C | 0.008009 | 3.808560 | 0.341400 |
| C | -0.721311 | 4.840870 | 0.948530 |
| C | 1.409749 | 3.912450 | 0.244360 |
| C | -0.023851 | 5.934010 | 1.447010 |
| H | -1.795361 | 4.795329 | 1.043470 |
| C | 2.041219 | 5.030871 | 0.762140 |
| H | 2.003949 | 3.129141 | -0.212540 |
| C | 1.356949 | 6.071630 | 1.377730 |
| H | 1.868298 | 6.937441 | 1.779310 |
| F | -0.731732 | 6.922530 | 2.029360 |
| F | 3.387879 | 5.111331 | 0.667650 |
| C | -4.819029 | -0.755241 | -2.852770 |
| H | -5.540369 | -0.754072 | -2.028120 |
| H | -5.377159 | -0.602712 | -3.784110 |
| H | -4.355669 | -1.746811 | -2.901670 |
| C | -2.761590 | 0.284289 | -3.855300 |
| H | -2.005980 | 1.075909 | -3.779630 |
| H | -2.256209 | -0.683371 | -3.886750 |
| H | -3.293470 | 0.432869 | -4.802820 |
| C | -4.465280 | 1.727149 | -2.716420 |
| H | -5.232260 | 1.803778 | -1.938480 |
| H | -3.755160 | 2.545809 | -2.561640 |
| H | -4.949500 | 1.877939 | -3.689020 |

TS Conformation 74

B3LYP/6-31G(d) Energy = -3411.884918
M06-2X/def2tzvpp/IEFPCM(chloroform) Energy = -3411.890521
M06-2X/def2tzvpp/IEFPCM(chloroform)//B3LYP/6-31G(d) Quasiharmonic Free Energy = -3411.163781

Frequencies (Top 3 out of 291)

1. -861.7669 cm-1
2. 7.6260 cm-1
3. 12.9721 cm-1

B3LYP/6-31G(d) Molecular Geometry in Cartesian Coordinates

| | | | |
|---|---|---|---|
| C | 3.451957 | -0.577658 | 0.917164 |
| C | 2.953879 | 0.413058 | 1.991707 |
| H | 3.622563 | 1.271427 | 2.046140 |
| C | 1.532118 | 2.142504 | 0.872928 |

| | | | |
|---|---|---|---|
| N | 0.251384 | 2.321826 | 0.419297 |
| H | -0.346553 | 1.498875 | 0.466884 |
| N | 1.680873 | 0.949316 | 1.525264 |
| H | 0.901730 | 0.297199 | 1.491721 |
| C | 5.053086 | -1.162249 | -0.851514 |
| C | 5.666588 | 0.651229 | 0.686068 |
| C | 6.467044 | -0.617134 | -1.183530 |
| H | 5.113028 | -2.202021 | -0.507097 |
| C | 6.947832 | 0.027088 | 0.122264 |
| H | 5.458215 | 1.634638 | 0.248817 |
| H | 5.702738 | 0.763402 | 1.770243 |
| H | 6.387027 | 0.141694 | -1.969727 |
| H | 7.125950 | -1.407983 | -1.553331 |
| H | 7.737728 | 0.768445 | -0.030240 |
| H | 7.327259 | -0.735947 | 0.812688 |
| N | 4.626142 | -0.319232 | 0.288495 |
| O | 2.754213 | -1.548212 | 0.590082 |
| S | 2.806547 | 3.236842 | 0.660913 |
| C | 4.123361 | -1.109590 | -2.054591 |
| C | 3.424382 | 0.055573 | -2.396639 |
| C | 4.028571 | -2.224892 | -2.895555 |
| C | 2.647122 | 0.101236 | -3.555048 |
| H | 3.473597 | 0.927902 | -1.749658 |
| C | 3.262727 | -2.177944 | -4.062634 |
| C | 2.567969 | -1.012434 | -4.396572 |
| H | 2.104210 | 1.010106 | -3.799009 |
| O | -1.042629 | -0.362340 | 1.274444 |
| O | -0.979206 | -0.103820 | -1.610183 |
| C | -1.829773 | -1.302685 | 1.178173 |
| C | -2.139808 | -2.088698 | -0.042552 |
| O | -2.592941 | -1.721559 | 2.196091 |
| C | -2.558184 | -0.924804 | 3.395305 |
| H | -3.215940 | -1.439805 | 4.095168 |
| H | -1.543207 | -0.868009 | 3.794863 |
| H | -2.935094 | 0.078763 | 3.184656 |
| S | -1.097746 | -1.564297 | -1.419446 |
| C | 0.528745 | -2.340727 | -1.376127 |
| H | 0.400226 | -3.418912 | -1.265463 |
| H | 1.024184 | -2.090137 | -2.317848 |
| H | 1.092894 | -1.921771 | -0.536507 |
| C | -1.891157 | -2.304640 | -2.860228 |
| H | -1.278368 | -2.024580 | -3.719770 |
| H | -1.953265 | -3.387561 | -2.745192 |
| H | -2.886318 | -1.849987 | -2.915266 |
| C | -2.208432 | -3.593554 | 0.112723 |
| C | -3.279095 | -4.322757 | -0.427706 |
| C | -1.208701 | -4.287184 | 0.819034 |
| C | -3.336245 | -5.710026 | -0.280164 |
| H | -4.087071 | -3.797236 | -0.926431 |
| C | -1.270017 | -5.671421 | 0.968175 |
| H | -0.383291 | -3.736852 | 1.264871 |
| C | -2.332527 | -6.388020 | 0.411967 |
| H | -4.176199 | -6.257850 | -0.698314 |
| H | -0.490575 | -6.188895 | 1.520698 |
| H | -2.381477 | -7.467372 | 0.527429 |
| C | -5.511479 | 0.067689 | 1.213850 |
| C | -5.726879 | 1.188460 | 2.017912 |
| C | -5.572144 | 2.474992 | 1.494950 |
| C | -5.196858 | 2.627297 | 0.159070 |
| C | -4.976985 | 1.508838 | -0.646737 |
| C | -5.131129 | 0.210402 | -0.133021 |
| H | -5.647744 | -0.930967 | 1.618847 |

| | | | |
|---|---|---|---|
| H | -6.030671 | 1.055001 | 3.054318 |
| H | -5.744487 | 3.346966 | 2.120572 |
| H | -5.066871 | 3.618296 | -0.266561 |
| H | -4.680573 | 1.640764 | -1.682327 |
| S | -4.881800 | -1.224303 | -1.168153 |
| H | -3.363441 | -1.675580 | -0.495716 |
| H | 4.561177 | -3.137833 | -2.636803 |
| H | 3.210329 | -3.050482 | -4.709220 |
| H | 1.975503 | -0.969268 | -5.307019 |
| C | 2.847449 | -0.202723 | 3.427571 |
| C | -0.374009 | 3.427536 | -0.194125 |
| C | -1.522389 | 3.142614 | -0.954024 |
| C | 0.039943 | 4.756397 | -0.024306 |
| C | -2.219528 | 4.193221 | -1.527943 |
| H | -1.862820 | 2.124559 | -1.102411 |
| C | -0.698519 | 5.759073 | -0.638204 |
| H | 0.912241 | 5.010526 | 0.558133 |
| C | -1.837345 | 5.522657 | -1.397938 |
| H | -2.395206 | 6.327328 | -1.860481 |
| F | -3.330267 | 3.915049 | -2.249392 |
| F | -0.295689 | 7.036692 | -0.473698 |
| C | 1.803640 | -1.333466 | 3.520382 |
| H | 2.046243 | -2.160941 | 2.849235 |
| H | 1.766778 | -1.715056 | 4.548027 |
| H | 0.799456 | -0.975130 | 3.268644 |
| C | 2.461630 | 0.926365 | 4.403764 |
| H | 2.400814 | 0.537759 | 5.427318 |
| H | 3.201423 | 1.735306 | 4.392821 |
| H | 1.490285 | 1.360166 | 4.146760 |
| C | 4.226470 | -0.764093 | 3.828581 |
| H | 4.996482 | 0.017168 | 3.827350 |
| H | 4.183870 | -1.176188 | 4.843231 |
| H | 4.550653 | -1.570591 | 3.160315 |

TS Conformation 75

B3LYP/6-31G(d) Energy = -3411.878029
M06-2X/def2tzvpp/IEFPCM(chloroform) Energy = -3411.888937
M06-2X/def2tzvpp/IEFPCM(chloroform)//B3LYP/6-31G(d) Quasiharmonic Free Energy = -3411.1637

Frequencies (Top 3 out of 291)

1. -805.0857 cm-1
2. 7.7910 cm-1
3. 12.9603 cm-1

B3LYP/6-31G(d) Molecular Geometry in Cartesian Coordinates

| | | | |
|---|---|---|---|
| C | 2.790241 | -1.491902 | 0.426088 |
| C | 2.921814 | -0.156320 | 1.189126 |
| H | 3.741802 | 0.436579 | 0.788098 |
| C | 1.621586 | 1.771832 | 0.210489 |
| N | 0.320311 | 2.144709 | -0.020962 |
| H | -0.376934 | 1.427872 | 0.161084 |
| N | 1.700315 | 0.592570 | 0.892667 |
| H | 0.830764 | 0.097289 | 1.068220 |
| C | 4.507480 | -0.746686 | -1.316522 |
| C | 3.135515 | -2.759784 | -1.630519 |
| C | 4.686000 | -1.319640 | -2.749956 |
| H | 4.059618 | 0.250350 | -1.373911 |
| C | 3.420127 | -2.147690 | -3.004344 |

| | | | |
|---|---|---|---|
| H | 3.741660 | -3.659634 | -1.454873 |
| H | 2.089833 | -3.017925 | -1.458994 |
| H | 4.840585 | -0.517117 | -3.476659 |
| H | 5.570065 | -1.966357 | -2.779655 |
| H | 3.553663 | -2.902853 | -3.785470 |
| H | 2.586632 | -1.496807 | -3.294139 |
| N | 3.520354 | -1.682146 | -0.703375 |
| O | 1.970309 | -2.344431 | 0.799944 |
| S | 2.978464 | 2.651587 | -0.285313 |
| C | 5.824950 | -0.650717 | -0.563211 |
| C | 6.496872 | -1.799454 | -0.120689 |
| C | 6.414340 | 0.600406 | -0.347708 |
| C | 7.730216 | -1.698915 | 0.520752 |
| H | 6.045388 | -2.777595 | -0.268345 |
| C | 7.652176 | 0.702640 | 0.292861 |
| C | 8.313530 | -0.445520 | 0.728043 |
| H | 8.236396 | -2.598418 | 0.861665 |
| O | -0.398370 | -1.032856 | -1.152281 |
| O | -1.379755 | -0.178167 | 1.467204 |
| C | -1.520847 | -1.406284 | -1.462303 |
| C | -2.649880 | -1.657764 | -0.517454 |
| O | -1.937156 | -1.562743 | -2.726854 |
| C | -0.990699 | -1.198966 | -3.749524 |
| H | -1.520255 | -1.339278 | -4.691311 |
| H | -0.684406 | -0.157357 | -3.629672 |
| H | -0.109527 | -1.844585 | -3.702549 |
| S | -2.028214 | -1.479763 | 1.161332 |
| C | -3.465210 | -1.713105 | 2.226676 |
| H | -3.863925 | -2.719567 | 2.095626 |
| H | -3.104190 | -1.551535 | 3.244773 |
| H | -4.202919 | -0.952904 | 1.958513 |
| C | -0.938154 | -2.843418 | 1.631679 |
| H | -1.420307 | -3.783292 | 1.355741 |
| H | -0.791401 | -2.775666 | 2.712690 |
| H | 0.017682 | -2.692964 | 1.116585 |
| C | -3.464637 | -2.915000 | -0.715236 |
| C | -4.863293 | -2.876365 | -0.606187 |
| C | -2.843648 | -4.138025 | -1.027287 |
| C | -5.617302 | -4.037549 | -0.783824 |
| H | -5.353305 | -1.925661 | -0.420549 |
| C | -3.599285 | -5.294752 | -1.209897 |
| H | -1.763396 | -4.179796 | -1.147035 |
| C | -4.989652 | -5.247853 | -1.081011 |
| H | -6.699690 | -3.990606 | -0.702558 |
| H | -3.103943 | -6.229838 | -1.456388 |
| H | -5.580059 | -6.148860 | -1.223158 |
| C | -4.276059 | 1.840656 | 1.474267 |
| C | -4.682310 | 2.098267 | 2.784536 |
| C | -5.895751 | 1.598156 | 3.263993 |
| C | -6.704470 | 0.843327 | 2.410985 |
| C | -6.300105 | 0.583687 | 1.099528 |
| C | -5.071874 | 1.068349 | 0.603887 |
| H | -3.337061 | 2.244785 | 1.109749 |
| H | -4.047751 | 2.699328 | 3.431845 |
| H | -6.211968 | 1.803748 | 4.283296 |
| H | -7.659488 | 0.459891 | 2.763687 |
| H | -6.943497 | 0.013053 | 0.435081 |
| S | -4.554333 | 0.708211 | -1.065202 |
| H | -3.468500 | -0.593183 | -0.685904 |
| H | 5.892011 | 1.497896 | -0.668005 |
| H | 8.093389 | 1.682301 | 0.455915 |
| H | 9.274597 | -0.367033 | 1.229211 |

| | | | |
|---|---|---|---|
| C | 3.175853 | -0.306504 | 2.729914 |
| C | -0.218308 | 3.329770 | -0.570106 |
| C | 0.401873 | 4.581910 | -0.456958 |
| C | -1.474560 | 3.220842 | -1.191030 |
| C | -0.244453 | 5.686514 | -0.994050 |
| H | 1.361351 | 4.700997 | 0.022750 |
| C | -2.071460 | 4.367589 | -1.698540 |
| H | -2.001077 | 2.274651 | -1.277896 |
| C | -1.480602 | 5.623538 | -1.624465 |
| H | -1.960207 | 6.504228 | -2.033238 |
| F | 0.356326 | 6.890282 | -0.884878 |
| F | -3.269541 | 4.259784 | -2.300610 |
| C | 3.490541 | 1.094977 | 3.291215 |
| H | 2.656510 | 1.786316 | 3.133501 |
| H | 3.679523 | 1.033209 | 4.369777 |
| H | 4.376596 | 1.527898 | 2.814620 |
| C | 4.395983 | -1.221821 | 2.948370 |
| H | 5.285315 | -0.839352 | 2.436563 |
| H | 4.627808 | -1.284457 | 4.018298 |
| H | 4.205945 | -2.239919 | 2.590496 |
| C | 1.965868 | -0.885526 | 3.489649 |
| H | 1.070427 | -0.267594 | 3.353055 |
| H | 1.742787 | -1.903281 | 3.162010 |
| H | 2.186190 | -0.905854 | 4.563950 |

TS Conformation 76

B3LYP/6-31G(d) Energy = -3411.875438
M06-2X/def2tzvpp/IEFPCM(chloroform) Energy = -3411.888523
M06-2X/def2tzvpp/IEFPCM(chloroform)//B3LYP/6-31G(d) Quasiharmonic Free Energy = -3411.163491

Frequencies (Top 3 out of 291)

1. -926.0079 cm-1
2. 8.3727 cm-1
3. 10.6313 cm-1

B3LYP/6-31G(d) Molecular Geometry in Cartesian Coordinates

| | | | |
|---|---|---|---|
| C | -2.517049 | -2.018792 | -0.213834 |
| C | -2.585307 | -1.517393 | -1.674247 |
| H | -3.587609 | -1.153712 | -1.896607 |
| C | -2.171880 | 0.930381 | -1.581742 |
| N | -1.168066 | 1.852229 | -1.425558 |
| H | -0.228894 | 1.505840 | -1.248640 |
| N | -1.721302 | -0.341065 | -1.764085 |
| H | -0.723224 | -0.519523 | -1.657736 |
| C | -3.657073 | -2.669135 | 1.859120 |
| C | -5.062754 | -2.035349 | -0.053027 |
| C | -5.097248 | -3.198226 | 2.056982 |
| H | -2.918052 | -3.471230 | 1.946708 |
| C | -5.938753 | -2.250581 | 1.190498 |
| H | -5.190891 | -1.043175 | -0.495218 |
| H | -5.281010 | -2.791041 | -0.816723 |
| H | -5.394374 | -3.205610 | 3.108972 |
| H | -5.164794 | -4.224353 | 1.674497 |
| H | -6.092917 | -1.300242 | 1.712813 |
| H | -6.921729 | -2.656675 | 0.934444 |
| N | -3.685341 | -2.208397 | 0.459340 |
| O | -1.423023 | -2.200663 | 0.331409 |
| S | -3.813544 | 1.353248 | -1.550726 |

| | | | |
|---|---|---|---|
| C | -3.295480 | -1.575470 | 2.862162 |
| C | -3.392895 | -0.211937 | 2.562921 |
| C | -2.892371 | -1.953497 | 4.151238 |
| C | -3.102569 | 0.750384 | 3.533727 |
| H | -3.676716 | 0.107011 | 1.563874 |
| C | -2.604318 | -0.994926 | 5.122445 |
| C | -2.711285 | 0.364542 | 4.816164 |
| H | -3.179539 | 1.803971 | 3.279041 |
| O | 1.040763 | 1.460177 | 1.622857 |
| O | 2.009891 | 2.561835 | -1.020658 |
| C | 1.838924 | 0.537575 | 1.492472 |
| C | 3.034872 | 0.585116 | 0.604313 |
| O | 1.724095 | -0.657436 | 2.069108 |
| C | 0.510024 | -0.917728 | 2.821217 |
| H | 0.771776 | -1.718694 | 3.512682 |
| H | 0.193300 | -0.023715 | 3.358669 |
| H | -0.269151 | -1.247068 | 2.131014 |
| S | 3.125969 | 2.223729 | -0.113038 |
| C | 3.342990 | 3.530694 | 1.129404 |
| H | 4.203628 | 3.286639 | 1.755985 |
| H | 3.487603 | 4.472331 | 0.594432 |
| H | 2.417616 | 3.548131 | 1.706055 |
| C | 4.672965 | 2.237324 | -1.039208 |
| H | 4.717507 | 3.209845 | -1.534518 |
| H | 5.527073 | 2.074595 | -0.381861 |
| H | 4.579258 | 1.435333 | -1.777320 |
| C | 4.344800 | 0.058127 | 1.131259 |
| C | 5.152593 | -0.771720 | 0.338076 |
| C | 4.777360 | 0.378672 | 2.430845 |
| C | 6.365406 | -1.256499 | 0.829958 |
| H | 4.817180 | -1.058064 | -0.653098 |
| C | 5.988252 | -0.107516 | 2.920821 |
| H | 4.148069 | 0.992507 | 3.071588 |
| C | 6.788697 | -0.923007 | 2.117263 |
| H | 6.972223 | -1.907562 | 0.207128 |
| H | 6.303163 | 0.144659 | 3.929742 |
| H | 7.731722 | -1.305316 | 2.498277 |
| C | 2.347101 | -3.150720 | -0.753753 |
| C | 2.779890 | -4.466021 | -0.582249 |
| C | 3.648855 | -5.055184 | -1.503747 |
| C | 4.082044 | -4.312424 | -2.603783 |
| C | 3.659491 | -2.993256 | -2.776030 |
| C | 2.787457 | -2.390785 | -1.852295 |
| H | 1.654495 | -2.708713 | -0.044009 |
| H | 2.428679 | -5.035255 | 0.275380 |
| H | 3.979992 | -6.081624 | -1.368722 |
| H | 4.753901 | -4.759534 | -3.333181 |
| H | 4.000814 | -2.418165 | -3.632357 |
| S | 2.260906 | -0.698027 | -2.089179 |
| H | 2.668785 | -0.070825 | -0.561267 |
| H | -2.802776 | -3.010658 | 4.395398 |
| H | -2.294834 | -1.308714 | 6.116398 |
| H | -2.488029 | 1.114672 | 5.570409 |
| C | -2.236087 | -2.603348 | -2.747843 |
| C | -1.325110 | 3.246438 | -1.210100 |
| C | -2.000008 | 4.055887 | -2.130674 |
| C | -0.721947 | 3.806967 | -0.078540 |
| C | -2.060532 | 5.420146 | -1.884893 |
| H | -2.471891 | 3.637639 | -3.009393 |
| C | -0.794098 | 5.181005 | 0.097752 |
| H | -0.215954 | 3.181342 | 0.646097 |
| C | -1.460992 | 6.022471 | -0.782421 |

| | | | |
|---|---|---|---|
| H | -1.514998 | 7.092301 | -0.621655 |
| F | -2.709238 | 6.208756 | -2.767221 |
| F | -0.189116 | 5.724014 | 1.182151 |
| C | -0.811501 | -3.163703 | -2.594041 |
| H | -0.657598 | -3.624893 | -1.615654 |
| H | -0.631573 | -3.923001 | -3.364934 |
| H | -0.049374 | -2.388556 | -2.719825 |
| C | -2.387146 | -1.962003 | -4.141946 |
| H | -1.676447 | -1.142105 | -4.282146 |
| H | -2.198144 | -2.710047 | -4.921115 |
| H | -3.397182 | -1.562202 | -4.293025 |
| C | -3.246481 | -3.761708 | -2.622915 |
| H | -3.195851 | -4.246403 | -1.640717 |
| H | -4.275816 | -3.420755 | -2.788065 |
| H | -3.029390 | -4.526984 | -3.376774 |

TS Conformation 77

B3LYP/6-31G(d) Energy = -3411.887023
M06-2X/def2tzvpp/IEFPCM(chloroform) Energy = -3411.88946
M06-2X/def2tzvpp/IEFPCM(chloroform)//B3LYP/6-31G(d) Quasiharmonic Free Energy = -3411.16345

Frequencies (Top 3 out of 291)

1. -963.4434 cm-1
2. 9.9616 cm-1
3. 14.5723 cm-1

B3LYP/6-31G(d) Molecular Geometry in Cartesian Coordinates

| | | | |
|---|---|---|---|
| C | -3.139141 | -1.431798 | -0.270978 |
| C | -3.148785 | -0.215683 | -1.217992 |
| H | -3.938802 | 0.481474 | -0.942522 |
| C | -1.731295 | 1.762088 | -0.538988 |
| N | -0.408474 | 2.060338 | -0.325886 |
| H | 0.236923 | 1.302708 | -0.527420 |
| N | -1.882214 | 0.474550 | -0.980086 |
| H | -1.083015 | -0.143549 | -0.866195 |
| C | -4.872413 | -0.347779 | 1.248008 |
| C | -3.905691 | -2.555263 | 1.753897 |
| C | -4.984198 | -0.659081 | 2.762667 |
| H | -4.355928 | 0.605106 | 1.104272 |
| C | -4.936422 | -2.191893 | 2.829628 |
| H | -4.090823 | -3.519055 | 1.269926 |
| H | -2.886846 | -2.583930 | 2.160633 |
| H | -5.889215 | -0.230248 | 3.199834 |
| H | -4.118654 | -0.230043 | 3.281724 |
| H | -5.916279 | -2.614181 | 2.582583 |
| H | -4.651153 | -2.568540 | 3.816189 |
| N | -4.005882 | -1.457700 | 0.771622 |
| O | -2.312784 | -2.346926 | -0.433243 |
| S | -3.025663 | 2.825454 | -0.328130 |
| C | -6.228581 | -0.271017 | 0.559015 |
| C | -6.910525 | -1.405543 | 0.100530 |
| C | -6.841911 | 0.982337 | 0.424514 |
| C | -8.182427 | -1.291680 | -0.463817 |
| H | -6.437880 | -2.381650 | 0.162154 |
| C | -8.112648 | 1.097877 | -0.139700 |
| C | -8.789144 | -0.040243 | -0.582358 |
| H | -8.696105 | -2.182162 | -0.816687 |
| O | 3.171389 | -4.243546 | -0.236376 |

| | | | |
|---|---|---|---|
| O | 0.529306 | -0.814982 | 0.219046 |
| C | 3.656455 | -3.236523 | 0.275804 |
| C | 2.945038 | -1.956008 | 0.491371 |
| O | 4.940590 | -3.148812 | 0.651640 |
| C | 5.752959 | -4.306630 | 0.390465 |
| H | 5.355395 | -5.182237 | 0.910308 |
| H | 5.790095 | -4.511810 | -0.682232 |
| H | 6.743262 | -4.050615 | 0.765971 |
| S | 1.207125 | -2.129400 | 0.061235 |
| C | 1.072074 | -2.702237 | -1.641585 |
| H | 1.488722 | -3.706065 | -1.704696 |
| H | 0.005717 | -2.663908 | -1.875461 |
| H | 1.654332 | -1.998263 | -2.245388 |
| C | 0.351484 | -3.380687 | 1.042583 |
| H | 0.449038 | -3.068369 | 2.084904 |
| H | -0.693761 | -3.360603 | 0.716091 |
| H | 0.844559 | -4.338036 | 0.865627 |
| C | 3.104116 | -1.246858 | 1.810727 |
| C | 3.250971 | -1.970831 | 3.007858 |
| C | 3.085881 | 0.154361 | 1.866737 |
| C | 3.388467 | -1.309724 | 4.226299 |
| H | 3.279297 | -3.057579 | 2.982576 |
| C | 3.214505 | 0.814053 | 3.089161 |
| H | 2.987401 | 0.720732 | 0.947677 |
| C | 3.366036 | 0.086690 | 4.269820 |
| H | 3.511836 | -1.883684 | 5.140805 |
| H | 3.200058 | 1.899966 | 3.113314 |
| H | 3.470409 | 0.603215 | 5.220194 |
| C | 5.788588 | 1.503109 | -1.353885 |
| C | 7.171650 | 1.688284 | -1.334340 |
| C | 8.031665 | 0.656598 | -1.715743 |
| C | 7.493148 | -0.565131 | -2.125041 |
| C | 6.111132 | -0.752922 | -2.153224 |
| C | 5.238831 | 0.276333 | -1.762297 |
| H | 5.127803 | 2.310104 | -1.050942 |
| H | 7.577659 | 2.645768 | -1.016345 |
| H | 9.108414 | 0.804058 | -1.698747 |
| H | 8.151533 | -1.374580 | -2.433044 |
| H | 5.696496 | -1.698888 | -2.490800 |
| S | 3.465511 | 0.050554 | -1.803569 |
| H | 3.280513 | -1.070745 | -0.541248 |
| H | -6.312511 | 1.873695 | 0.752410 |
| H | -8.568822 | 2.078813 | -0.242152 |
| H | -9.777206 | 0.048361 | -1.025724 |
| C | -3.377132 | -0.578042 | -2.729572 |
| C | 0.265303 | 3.240508 | 0.050379 |
| C | -0.327424 | 4.322240 | 0.715084 |
| C | 1.642323 | 3.263585 | -0.243827 |
| C | 0.478661 | 5.401172 | 1.057287 |
| H | -1.380313 | 4.335017 | 0.951972 |
| C | 2.387596 | 4.367641 | 0.135707 |
| H | 2.127012 | 2.448491 | -0.776578 |
| C | 1.840541 | 5.465224 | 0.790727 |
| H | 2.439630 | 6.322361 | 1.071873 |
| F | -0.094046 | 6.440908 | 1.698578 |
| F | 3.708810 | 4.383498 | -0.149365 |
| C | -2.204360 | -1.362810 | -3.345706 |
| H | -2.085309 | -2.340536 | -2.872174 |
| H | -2.389722 | -1.520387 | -4.414844 |
| H | -1.263242 | -0.807300 | -3.259388 |
| C | -3.554066 | 0.742062 | -3.506361 |
| H | -4.408048 | 1.317115 | -3.133139 |

| | | | |
|---|---|---|---|
| H | -2.665877 | 1.376533 | -3.426062 |
| H | -3.722910 | 0.529935 | -4.568896 |
| C | -4.665906 | -1.413071 | -2.853611 |
| H | -4.571700 | -2.381502 | -2.348273 |
| H | -5.530332 | -0.889970 | -2.431143 |
| H | -4.880230 | -1.612811 | -3.910008 |

TS Conformation 78

B3LYP/6-31G(d) Energy = -3411.891631
M06-2X/def2tzvpp/IEFPCM(chloroform) Energy = -3411.889893
M06-2X/def2tzvpp/IEFPCM(chloroform)//B3LYP/6-31G(d) Quasiharmonic Free Energy = -3411.163443

Frequencies (Top 3 out of 291)

1. -1017.7270 cm-1
2. 10.0894 cm-1
3. 11.6063 cm-1

B3LYP/6-31G(d) Molecular Geometry in Cartesian Coordinates

| | | | |
|---|---|---|---|
| C | -2.369518 | -1.776292 | -0.253384 |
| C | -3.053766 | -0.720316 | -1.145750 |
| H | -4.017275 | -0.423988 | -0.733289 |
| C | -2.512492 | 1.694301 | -0.605145 |
| N | -1.394288 | 2.488361 | -0.515166 |
| H | -0.522731 | 1.998702 | -0.696704 |
| N | -2.183917 | 0.452898 | -1.079503 |
| H | -1.193921 | 0.223817 | -1.102493 |
| C | -4.140825 | -1.499182 | 1.554427 |
| C | -2.241438 | -3.043793 | 1.825062 |
| C | -3.870237 | -1.775291 | 3.056242 |
| H | -4.117571 | -0.421874 | 1.368682 |
| C | -3.146909 | -3.129297 | 3.059557 |
| H | -2.058110 | -4.008431 | 1.342341 |
| H | -1.263389 | -2.607608 | 2.064308 |
| H | -4.792255 | -1.770177 | 3.642755 |
| H | -3.210793 | -0.993109 | 3.451160 |
| H | -3.868520 | -3.946221 | 2.952028 |
| H | -2.577060 | -3.307418 | 3.976065 |
| N | -2.965465 | -2.139429 | 0.909602 |
| O | -1.263044 | -2.240817 | -0.574107 |
| S | -4.089114 | 2.150522 | -0.216518 |
| C | -5.485356 | -2.040456 | 1.086156 |
| C | -5.668374 | -3.371380 | 0.689432 |
| C | -6.595332 | -1.184377 | 1.100920 |
| C | -6.934166 | -3.839453 | 0.331583 |
| H | -4.815049 | -4.041450 | 0.637258 |
| C | -7.860728 | -1.650400 | 0.743244 |
| C | -8.034872 | -2.981681 | 0.360712 |
| H | -7.057732 | -4.874542 | 0.023954 |
| O | 2.809974 | 2.074171 | -1.757040 |
| O | 0.653204 | 0.432630 | -0.167100 |
| C | 3.522050 | 1.749315 | -0.811254 |
| C | 3.465611 | 0.426586 | -0.130989 |
| O | 4.487625 | 2.526373 | -0.308578 |
| C | 4.648567 | 3.815458 | -0.937328 |
| H | 4.802229 | 3.698272 | -2.012093 |
| H | 3.768351 | 4.437911 | -0.757790 |
| H | 5.529013 | 4.251550 | -0.465994 |
| S | 1.895025 | -0.313243 | -0.540094 |

| | | | |
|---|---|---|---|
| C | 1.881454 | -0.728966 | -2.294066 |
| H | 2.839259 | -1.208106 | -2.531558 |
| H | 1.780745 | 0.215822 | -2.826789 |
| H | 1.021488 | -1.385260 | -2.446895 |
| C | 1.884034 | -1.915893 | 0.276265 |
| H | 2.714716 | -2.518808 | -0.099571 |
| H | 0.903431 | -2.347618 | 0.051063 |
| H | 1.994898 | -1.732390 | 1.345774 |
| C | 3.739452 | 0.380665 | 1.352696 |
| C | 4.669812 | -0.528535 | 1.876509 |
| C | 3.066010 | 1.245061 | 2.234359 |
| C | 4.923349 | -0.569461 | 3.248814 |
| H | 5.201586 | -1.199457 | 1.210812 |
| C | 3.320578 | 1.202155 | 3.603765 |
| H | 2.342053 | 1.954390 | 1.842879 |
| C | 4.251037 | 0.293660 | 4.114483 |
| H | 5.650457 | -1.277142 | 3.637409 |
| H | 2.793438 | 1.878543 | 4.270978 |
| H | 4.451432 | 0.261611 | 5.182098 |
| C | 4.839491 | -3.889029 | -1.249980 |
| C | 5.031692 | -5.100920 | -0.583461 |
| C | 6.065752 | -5.239841 | 0.344472 |
| C | 6.911459 | -4.156095 | 0.590565 |
| C | 6.720545 | -2.943468 | -0.073997 |
| C | 5.672217 | -2.782133 | -0.998957 |
| H | 4.054032 | -3.800299 | -1.996384 |
| H | 4.376757 | -5.941988 | -0.799186 |
| H | 6.217738 | -6.184042 | 0.860367 |
| H | 7.730736 | -4.255523 | 1.298804 |
| H | 7.391770 | -2.109013 | 0.108129 |
| S | 5.446820 | -1.232439 | -1.862842 |
| H | 4.406161 | -0.402027 | -0.856231 |
| H | -6.462576 | -0.142508 | 1.382781 |
| H | -8.707914 | -0.969954 | 0.754144 |
| H | -9.018925 | -3.345432 | 0.077860 |
| C | -3.314370 | -1.200715 | -2.618378 |
| C | -1.170733 | 3.836374 | -0.190672 |
| C | 0.159208 | 4.270668 | -0.371321 |
| C | -2.135499 | 4.731338 | 0.291034 |
| C | 0.486418 | 5.578061 | -0.059837 |
| H | 0.920793 | 3.609304 | -0.773419 |
| C | -1.736410 | 6.031764 | 0.577636 |
| H | -3.161828 | 4.430267 | 0.438490 |
| C | -0.436853 | 6.498615 | 0.421915 |
| H | -0.162170 | 7.519057 | 0.657713 |
| F | 1.767809 | 5.980301 | -0.235856 |
| F | -2.666693 | 6.889360 | 1.043066 |
| C | -2.014985 | -1.384258 | -3.425543 |
| H | -1.378358 | -2.157265 | -2.988319 |
| H | -2.260000 | -1.678019 | -4.453105 |
| H | -1.447712 | -0.447715 | -3.482486 |
| C | -4.185387 | -0.134697 | -3.312341 |
| H | -3.689373 | 0.840956 | -3.326222 |
| H | -4.382195 | -0.429894 | -4.350012 |
| H | -5.148141 | -0.010065 | -2.805159 |
| C | -4.082424 | -2.535696 | -2.581848 |
| H | -3.478908 | -3.337668 | -2.141124 |
| H | -5.012385 | -2.452805 | -2.009154 |
| H | -4.344666 | -2.842809 | -3.601006 |

TS Conformation 79

B3LYP/6-31G(d) Energy = -3411.890474
M06-2X/def2tzvpp/IEFPCM(chloroform) Energy = -3411.889335
M06-2X/def2tzvpp/IEFPCM(chloroform)//B3LYP/6-31G(d) Quasiharmonic Free Energy = -3411.163383

Frequencies (Top 3 out of 291)

1. -976.5847 cm-1
2. 7.4630 cm-1
3. 8.3810 cm-1

B3LYP/6-31G(d) Molecular Geometry in Cartesian Coordinates

| | | | |
|---|---|---|---|
| C | 2.393488 | -1.509050 | 0.862207 |
| C | 3.134950 | -0.237175 | 1.323124 |
| H | 3.977028 | -0.018355 | 0.668234 |
| C | 2.321110 | 1.939314 | 0.329935 |
| N | 1.172776 | 2.700011 | 0.301010 |
| H | 0.403834 | 2.319739 | 0.846431 |
| N | 2.173629 | 0.853510 | 1.142711 |
| H | 1.249762 | 0.667405 | 1.525196 |
| C | 3.666014 | -1.552600 | -1.343295 |
| C | 1.875308 | -3.169829 | -0.843281 |
| C | 3.086687 | -2.148660 | -2.652391 |
| H | 3.611194 | -0.461127 | -1.379258 |
| C | 2.487235 | -3.491636 | -2.211986 |
| H | 1.885290 | -4.013232 | -0.146559 |
| H | 0.840370 | -2.817413 | -0.923407 |
| H | 3.849281 | -2.241751 | -3.429831 |
| H | 2.296224 | -1.486687 | -3.026098 |
| H | 3.276594 | -4.245301 | -2.115262 |
| H | 1.742653 | -3.877510 | -2.914343 |
| N | 2.718497 | -2.069041 | -0.327770 |
| O | 1.470226 | -1.969141 | 1.549558 |
| S | 3.750386 | 2.282545 | -0.499523 |
| C | 5.116272 | -1.954064 | -1.101580 |
| C | 5.473416 | -3.130824 | -0.431539 |
| C | 6.134111 | -1.134645 | -1.609939 |
| C | 6.815817 | -3.487837 | -0.286374 |
| H | 4.700200 | -3.762713 | -0.004247 |
| C | 7.475248 | -1.489703 | -1.466018 |
| C | 7.820703 | -2.670594 | -0.805710 |
| H | 7.074826 | -4.404128 | 0.237818 |
| O | -1.304596 | -1.245915 | -0.640119 |
| O | -0.948462 | 1.030447 | 1.814033 |
| C | -2.381109 | -0.668677 | -0.727237 |
| C | -3.154266 | -0.144512 | 0.431797 |
| O | -3.050998 | -0.492371 | -1.873313 |
| C | -2.478310 | -1.111579 | -3.039787 |
| H | -1.437397 | -0.804895 | -3.165468 |
| H | -2.537873 | -2.198990 | -2.947464 |
| H | -3.087848 | -0.766569 | -3.874704 |
| S | -2.063187 | 0.029113 | 1.834128 |
| C | -3.197913 | 0.455366 | 3.169258 |
| H | -3.957973 | -0.326352 | 3.248199 |
| H | -2.597113 | 0.523853 | 4.078322 |
| H | -3.651903 | 1.416968 | 2.928556 |
| C | -1.464915 | -1.597946 | 2.319662 |
| H | -2.313283 | -2.287598 | 2.236928 |
| H | -0.634190 | -1.858798 | 1.661858 |
| H | -1.116051 | -1.498336 | 3.350426 |
| C | -4.030177 | 1.071961 | 0.238014 |

| | | | |
|---|---|---|---|
| C | -5.405437 | 1.003121 | 0.507906 |
| C | -3.485208 | 2.288577 | -0.206020 |
| C | -6.211720 | 2.130652 | 0.341137 |
| H | -5.839020 | 0.061875 | 0.833041 |
| C | -4.288512 | 3.418418 | -0.355081 |
| H | -2.426712 | 2.348576 | -0.440749 |
| C | -5.656042 | 3.339315 | -0.080540 |
| H | -7.276823 | 2.061776 | 0.545276 |
| H | -3.843158 | 4.356932 | -0.670138 |
| H | -6.285310 | 4.217408 | -0.198637 |
| C | -3.742991 | -3.957377 | -0.626294 |
| C | -3.783451 | -4.767731 | -1.762936 |
| C | -5.006246 | -5.154689 | -2.315136 |
| C | -6.190626 | -4.718323 | -1.717969 |
| C | -6.154809 | -3.900897 | -0.588174 |
| C | -4.930204 | -3.506449 | -0.023241 |
| H | -2.783647 | -3.676634 | -0.201772 |
| H | -2.851640 | -5.110992 | -2.207518 |
| H | -5.035700 | -5.791550 | -3.195216 |
| H | -7.150954 | -5.012365 | -2.135038 |
| H | -7.079444 | -3.560142 | -0.131901 |
| S | -4.921797 | -2.490845 | 1.447739 |
| H | -3.959527 | -1.226352 | 0.867788 |
| H | 5.871334 | -0.206364 | -2.111805 |
| H | 8.250313 | -0.838383 | -1.861179 |
| H | 8.865181 | -2.946767 | -0.688966 |
| C | 3.695505 | -0.313635 | 2.786586 |
| C | 0.832061 | 3.908862 | -0.329826 |
| C | 1.619674 | 4.586639 | -1.271392 |
| C | -0.426758 | 4.436140 | 0.025733 |
| C | 1.123363 | 5.760833 | -1.824228 |
| H | 2.589628 | 4.216618 | -1.565916 |
| C | -0.864573 | 5.604586 | -0.574471 |
| H | -1.055193 | 3.948220 | 0.762996 |
| C | -0.115947 | 6.305336 | -1.510803 |
| H | -0.474478 | 7.219528 | -1.966813 |
| F | 1.887557 | 6.403434 | -2.728797 |
| F | -2.086261 | 6.080871 | -0.236202 |
| C | 4.608915 | 0.909156 | 3.006001 |
| H | 4.062760 | 1.848251 | 2.869635 |
| H | 5.011403 | 0.897517 | 4.026115 |
| H | 5.452719 | 0.911899 | 2.307488 |
| C | 4.531390 | -1.600274 | 2.930616 |
| H | 3.907149 | -2.496401 | 2.845208 |
| H | 5.320312 | -1.657119 | 2.172036 |
| H | 5.013662 | -1.623823 | 3.914884 |
| C | 2.584551 | -0.303485 | 3.855200 |
| H | 1.919192 | -1.162245 | 3.745566 |
| H | 3.037438 | -0.334959 | 4.853487 |
| H | 1.985503 | 0.613519 | 3.799786 |

TS Conformation 80

B3LYP/6-31G(d) Energy = -3411.883395
M06-2X/def2tzvpp/IEFPCM(chloroform) Energy = -3411.890034
M06-2X/def2tzvpp/IEFPCM(chloroform)//B3LYP/6-31G(d) Quasiharmonic Free Energy = -3411.163043

Frequencies (Top 3 out of 291)

1. -970.0473 cm-1
2. 7.3754 cm-1

3. 12.4821 cm-1

B3LYP/6-31G(d) Molecular Geometry in Cartesian Coordinates

| | | | |
|---|---|---|---|
| C | -2.685929 | -1.424130 | -0.143525 |
| C | -2.881574 | -0.268623 | -1.145232 |
| H | -3.706449 | 0.370552 | -0.835673 |
| C | -1.603843 | 1.814602 | -0.544373 |
| N | -0.305496 | 2.254187 | -0.457655 |
| H | 0.407524 | 1.543987 | -0.603631 |
| N | -1.677123 | 0.552081 | -1.059593 |
| H | -0.802721 | 0.058805 | -1.223861 |
| C | -4.536207 | -0.552787 | 1.378709 |
| C | -3.231093 | -2.566151 | 1.938184 |
| C | -4.548113 | -0.802817 | 2.908316 |
| H | -4.175862 | 0.459037 | 1.174831 |
| C | -4.263006 | -2.305326 | 3.042306 |
| H | -3.292054 | -3.566880 | 1.500138 |
| H | -2.204520 | -2.433322 | 2.301188 |
| H | -5.493102 | -0.495120 | 3.362699 |
| H | -3.742349 | -0.220787 | 3.371334 |
| H | -5.175392 | -2.884135 | 2.863746 |
| H | -3.883272 | -2.581344 | 4.030211 |
| N | -3.521471 | -1.537482 | 0.916617 |
| O | -1.731877 | -2.213079 | -0.279041 |
| S | -2.959819 | 2.703167 | -0.064632 |
| C | -5.908456 | -0.718658 | 0.740578 |
| C | -6.440815 | -1.969017 | 0.399025 |
| C | -6.692694 | 0.424456 | 0.532642 |
| C | -7.730734 | -2.075086 | -0.123384 |
| H | -5.837595 | -2.864508 | 0.518007 |
| C | -7.982272 | 0.320463 | 0.009489 |
| C | -8.507082 | -0.931088 | -0.317011 |
| H | -8.126513 | -3.052957 | -0.385064 |
| O | 3.455025 | -3.814747 | 0.158298 |
| O | 1.196158 | -0.402488 | -1.141122 |
| C | 4.155757 | -2.871801 | -0.197189 |
| C | 3.589458 | -1.579631 | -0.676140 |
| O | 5.488876 | -3.010131 | -0.096488 |
| C | 6.429118 | -1.929159 | -0.281469 |
| H | 6.647129 | -1.793394 | -1.343190 |
| H | 6.062843 | -0.999115 | 0.158985 |
| H | 7.329918 | -2.253716 | 0.241810 |
| S | 1.785383 | -1.673534 | -0.626755 |
| C | 1.126084 | -3.049839 | -1.595392 |
| H | 1.592855 | -3.964918 | -1.231753 |
| H | 0.043758 | -3.027116 | -1.443778 |
| H | 1.390394 | -2.842781 | -2.634587 |
| C | 1.237462 | -1.988535 | 1.058288 |
| H | 1.728680 | -2.893783 | 1.412683 |
| H | 1.534148 | -1.120713 | 1.651368 |
| H | 0.150776 | -2.095153 | 0.989264 |
| C | 4.029365 | -1.031348 | -2.007735 |
| C | 3.996487 | 0.351891 | -2.249816 |
| C | 4.470579 | -1.887157 | -3.033964 |
| C | 4.402104 | 0.863255 | -3.482456 |
| H | 3.654351 | 1.024112 | -1.470607 |
| C | 4.887825 | -1.371648 | -4.260135 |
| H | 4.503867 | -2.960942 | -2.864088 |
| C | 4.850793 | 0.006030 | -4.488305 |
| H | 4.372391 | 1.935688 | -3.652966 |
| H | 5.235454 | -2.045962 | -5.038024 |

| | | | |
|---|---|---|---|
| H | 5.171599 | 0.408254 | -5.445290 |
| C | 4.190159 | -1.402086 | 3.205403 |
| C | 3.782925 | -2.040037 | 4.378082 |
| C | 2.862254 | -1.431301 | 5.233757 |
| C | 2.350922 | -0.174853 | 4.900998 |
| C | 2.744284 | 0.460740 | 3.722323 |
| C | 3.671116 | -0.142486 | 2.851114 |
| H | 4.925328 | -1.879933 | 2.564210 |
| H | 4.196967 | -3.014387 | 4.626604 |
| H | 2.552781 | -1.926317 | 6.150324 |
| H | 1.638852 | 0.315903 | 5.560470 |
| H | 2.339477 | 1.436270 | 3.468151 |
| S | 4.156344 | 0.687680 | 1.347246 |
| H | 3.843485 | -0.573016 | 0.292893 |
| H | -6.283952 | 1.403037 | 0.773290 |
| H | -8.572473 | 1.218843 | -0.150145 |
| H | -9.509741 | -1.013940 | -0.727512 |
| C | -3.188501 | -0.722526 | -2.617119 |
| C | 0.224452 | 3.516912 | -0.109117 |
| C | -0.440390 | 4.725437 | -0.356155 |
| C | 1.517940 | 3.521503 | 0.439656 |
| C | 0.204816 | 5.908735 | -0.023050 |
| H | -1.431153 | 4.753899 | -0.784098 |
| C | 2.110574 | 4.740667 | 0.738473 |
| H | 2.062131 | 2.603918 | 0.648909 |
| C | 1.480472 | 5.961088 | 0.524894 |
| H | 1.957938 | 6.900979 | 0.772740 |
| F | -0.436493 | 7.071788 | -0.260251 |
| F | 3.348976 | 4.742845 | 1.267296 |
| C | -2.015578 | -1.470095 | -3.279231 |
| H | -1.815615 | -2.422122 | -2.781847 |
| H | -2.262608 | -1.678645 | -4.327059 |
| H | -1.095599 | -0.874227 | -3.275681 |
| C | -3.501012 | 0.545799 | -3.437066 |
| H | -4.352971 | 1.093201 | -3.019373 |
| H | -2.645281 | 1.227885 | -3.461213 |
| H | -3.745411 | 0.272849 | -4.470487 |
| C | -4.427225 | -1.637478 | -2.613984 |
| H | -4.246872 | -2.564314 | -2.056784 |
| H | -5.297463 | -1.140798 | -2.173378 |
| H | -4.685930 | -1.917015 | -3.641998 |

TS Conformation 81

B3LYP/6-31G(d) Energy = -3411.883528
M06-2X/def2tzvpp/IEFPCM(chloroform) Energy = -3411.888889
M06-2X/def2tzvpp/IEFPCM(chloroform)//B3LYP/6-31G(d) Quasiharmonic Free Energy = -3411.163033

Frequencies (Top 3 out of 291)

1. -982.9323 cm-1
2. 5.8465 cm-1
3. 10.8360 cm-1

B3LYP/6-31G(d) Molecular Geometry in Cartesian Coordinates

| | | | |
|---|---|---|---|
| C | -2.632686 | -1.741413 | 0.737512 |
| C | -2.754637 | -0.945170 | -0.579313 |
| H | -3.706849 | -0.421216 | -0.627971 |
| C | -1.971881 | 1.438704 | -0.551228 |
| N | -0.839872 | 2.197249 | -0.296181 |

| | | | |
|---|---|---|---|
| H | -0.125421 | 1.773019 | 0.291146 |
| N | -1.729821 | 0.102977 | -0.502863 |
| H | -0.772591 | -0.219109 | -0.364600 |
| C | -4.821234 | -0.741470 | 1.614822 |
| C | -3.377941 | -2.126892 | 3.033689 |
| C | -5.266040 | -0.660046 | 3.103552 |
| H | -4.509746 | 0.251044 | 1.273403 |
| C | -4.024410 | -1.054519 | 3.913769 |
| H | -3.854750 | -3.106292 | 3.179768 |
| H | -2.304414 | -2.252985 | 3.178146 |
| H | -6.074100 | -1.377723 | 3.282772 |
| H | -5.651802 | 0.334795 | 3.343982 |
| H | -4.268381 | -1.419595 | 4.916384 |
| H | -3.343301 | -0.201671 | 4.019542 |
| N | -3.624791 | -1.630481 | 1.668329 |
| O | -1.617850 | -2.408340 | 0.966378 |
| S | -3.483663 | 2.123504 | -0.859728 |
| C | -5.936158 | -1.240130 | 0.708660 |
| C | -6.372154 | -2.572179 | 0.757378 |
| C | -6.583132 | -0.350165 | -0.156111 |
| C | -7.430638 | -3.003210 | -0.040017 |
| H | -5.870648 | -3.279160 | 1.413800 |
| C | -7.646470 | -0.780136 | -0.954695 |
| C | -8.073378 | -2.106560 | -0.898678 |
| H | -7.753317 | -4.040161 | 0.005468 |
| O | 1.402667 | -0.662180 | -0.351930 |
| O | 1.770023 | 1.921665 | 1.461473 |
| C | 2.335008 | -1.086115 | 0.335176 |
| C | 3.464210 | -0.241207 | 0.808473 |
| O | 2.497142 | -2.352414 | 0.697274 |
| C | 1.526348 | -3.307654 | 0.206930 |
| H | 0.515608 | -2.993327 | 0.469567 |
| H | 1.631084 | -3.403726 | -0.876343 |
| H | 1.790004 | -4.244952 | 0.696580 |
| S | 2.959387 | 1.461860 | 0.689154 |
| C | 4.425830 | 2.377408 | 1.200138 |
| H | 4.194996 | 3.437028 | 1.072317 |
| H | 4.611446 | 2.144021 | 2.248982 |
| H | 5.269203 | 2.070183 | 0.576086 |
| C | 2.809327 | 1.899717 | -1.056698 |
| H | 2.713965 | 2.987094 | -1.100370 |
| H | 3.705185 | 1.527917 | -1.569269 |
| H | 1.911194 | 1.405942 | -1.423609 |
| C | 4.076117 | -0.558673 | 2.155283 |
| C | 5.434274 | -0.895494 | 2.251665 |
| C | 3.294652 | -0.533119 | 3.323996 |
| C | 5.996246 | -1.202183 | 3.492295 |
| H | 6.040391 | -0.929835 | 1.351114 |
| C | 3.862011 | -0.831873 | 4.561655 |
| H | 2.240169 | -0.279185 | 3.259467 |
| C | 5.215186 | -1.167935 | 4.648063 |
| H | 7.047608 | -1.469975 | 3.552415 |
| H | 3.246278 | -0.807937 | 5.456571 |
| H | 5.656309 | -1.405961 | 5.612284 |
| C | 3.939126 | -1.931777 | -2.718385 |
| C | 3.604185 | -3.092075 | -3.419186 |
| C | 4.562981 | -4.079354 | -3.653844 |
| C | 5.861778 | -3.894204 | -3.175580 |
| C | 6.197457 | -2.741879 | -2.464846 |
| C | 5.241022 | -1.741335 | -2.224180 |
| H | 3.184012 | -1.167611 | -2.559368 |
| H | 2.590800 | -3.215033 | -3.794868 |

| | | | |
|---|---|---|---|
| H | 4.302611 | -4.978693 | -4.205430 |
| H | 6.620096 | -4.653515 | -3.351716 |
| H | 7.207114 | -2.607867 | -2.088072 |
| S | 5.714649 | -0.265540 | -1.332691 |
| H | 4.480443 | -0.289027 | -0.183820 |
| H | -6.240399 | 0.679460 | -0.216850 |
| H | -8.134250 | -0.076791 | -1.624309 |
| H | -8.897780 | -2.443155 | -1.521592 |
| C | -2.655091 | -1.814387 | -1.882712 |
| C | -0.690284 | 3.589468 | -0.489720 |
| C | -1.112720 | 4.212043 | -1.673176 |
| C | -0.025680 | 4.336080 | 0.494681 |
| C | -0.879648 | 5.570471 | -1.827191 |
| H | -1.623724 | 3.661815 | -2.451010 |
| C | 0.196149 | 5.689383 | 0.271643 |
| H | 0.309696 | 3.879856 | 1.417151 |
| C | -0.221002 | 6.346215 | -0.877686 |
| H | -0.047624 | 7.404686 | -1.027254 |
| F | -1.288134 | 6.167033 | -2.964672 |
| F | 0.842957 | 6.397778 | 1.222482 |
| C | -2.900233 | -0.889365 | -3.092354 |
| H | -2.127812 | -0.117327 | -3.171115 |
| H | -2.881785 | -1.477341 | -4.017828 |
| H | -3.869430 | -0.384697 | -3.025966 |
| C | -3.762378 | -2.885714 | -1.842091 |
| H | -3.630923 | -3.572288 | -0.997945 |
| H | -4.758785 | -2.438657 | -1.766901 |
| H | -3.731850 | -3.483886 | -2.760474 |
| C | -1.292558 | -2.510626 | -2.056435 |
| H | -1.126217 | -3.260178 | -1.280886 |
| H | -1.266902 | -3.010795 | -3.032585 |
| H | -0.458575 | -1.801511 | -2.026924 |

TS Conformation 82

B3LYP/6-31G(d) Energy = -3411.889155
M06-2X/def2tzvpp/IEFPCM(chloroform) Energy = -3411.888798
M06-2X/def2tzvpp/IEFPCM(chloroform)//B3LYP/6-31G(d) Quasiharmonic Free Energy = -3411.16298

Frequencies (Top 3 out of 291)

1. -978.7410 cm-1
2. 6.9686 cm-1
3. 7.5777 cm-1

B3LYP/6-31G(d) Molecular Geometry in Cartesian Coordinates

| | | | |
|---|---|---|---|
| C | 2.406202 | -1.604898 | 0.655366 |
| C | 3.172202 | -0.370081 | 1.177433 |
| H | 4.018060 | -0.130836 | 0.536055 |
| C | 2.372335 | 1.847785 | 0.263732 |
| N | 1.229527 | 2.617367 | 0.267083 |
| H | 0.466343 | 2.233677 | 0.818251 |
| N | 2.221987 | 0.737530 | 1.041323 |
| H | 1.298079 | 0.544332 | 1.420019 |
| C | 3.793554 | -1.654067 | -1.488071 |
| C | 1.788069 | -3.052217 | -1.214092 |
| C | 3.423188 | -2.358474 | -2.822635 |
| H | 3.698920 | -0.569977 | -1.609733 |
| C | 1.931298 | -2.689615 | -2.693944 |
| H | 2.074903 | -4.095346 | -1.022009 |

| | | | |
|---|---|---|---|
| H | 0.786827 | -2.891973 | -0.814031 |
| H | 4.005695 | -3.281135 | -2.922580 |
| H | 3.663280 | -1.724089 | -3.680736 |
| H | 1.615791 | -3.499694 | -3.358838 |
| H | 1.315992 | -1.809296 | -2.913996 |
| N | 2.731138 | -2.127889 | -0.554801 |
| O | 1.448296 | -2.041632 | 1.308811 |
| S | 3.798829 | 2.214079 | -0.560804 |
| C | 5.210848 | -1.972669 | -1.038702 |
| C | 5.557890 | -3.250411 | -0.576259 |
| C | 6.209022 | -0.996272 | -1.134851 |
| C | 6.873012 | -3.544193 | -0.220019 |
| H | 4.791166 | -4.015707 | -0.483176 |
| C | 7.528531 | -1.289883 | -0.780416 |
| C | 7.864263 | -2.563810 | -0.322924 |
| H | 7.125188 | -4.538051 | 0.140611 |
| O | -1.386805 | -1.237285 | -0.695219 |
| O | -0.888981 | 0.977106 | 1.800679 |
| C | -2.450913 | -0.632169 | -0.725795 |
| C | -3.167756 | -0.119349 | 0.474186 |
| O | -3.159512 | -0.410072 | -1.840446 |
| C | -2.649071 | -1.014035 | -3.043086 |
| H | -1.609500 | -0.721670 | -3.208504 |
| H | -2.722841 | -2.102011 | -2.970742 |
| H | -3.288265 | -0.641331 | -3.843218 |
| S | -2.023107 | -0.001515 | 1.838469 |
| C | -3.099088 | 0.414273 | 3.224554 |
| H | -3.871495 | -0.354218 | 3.313123 |
| H | -2.463988 | 0.448940 | 4.111961 |
| H | -3.542048 | 1.390060 | 3.023761 |
| C | -1.439836 | -1.650821 | 2.263072 |
| H | -1.055899 | -1.583696 | 3.283998 |
| H | -2.302375 | -2.323822 | 2.191303 |
| H | -0.636023 | -1.906881 | 1.571133 |
| C | -4.023237 | 1.119851 | 0.341992 |
| C | -5.390088 | 1.071855 | 0.655511 |
| C | -3.467137 | 2.336636 | -0.087532 |
| C | -6.177137 | 2.219731 | 0.545159 |
| H | -5.832606 | 0.131359 | 0.970641 |
| C | -4.250572 | 3.486302 | -0.180057 |
| H | -2.415732 | 2.381539 | -0.355148 |
| C | -5.609843 | 3.427744 | 0.137223 |
| H | -7.236242 | 2.167212 | 0.782661 |
| H | -3.795509 | 4.423491 | -0.485027 |
| H | -6.223809 | 4.321354 | 0.063094 |
| C | -6.293864 | -3.773126 | -0.520892 |
| C | -6.394985 | -4.557494 | -1.670009 |
| C | -5.246535 | -5.007539 | -2.324455 |
| C | -3.993454 | -4.668011 | -1.810244 |
| C | -3.887672 | -3.891005 | -0.654688 |
| C | -5.038301 | -3.426430 | 0.006105 |
| H | -7.190794 | -3.421433 | -0.019812 |
| H | -7.378167 | -4.814824 | -2.057025 |
| H | -5.326862 | -5.618592 | -3.219538 |
| H | -3.088939 | -5.022613 | -2.299970 |
| H | -2.905605 | -3.646919 | -0.260567 |
| S | -4.945765 | -2.452524 | 1.502248 |
| H | -3.979461 | -1.194117 | 0.915632 |
| H | 5.947449 | 0.002589 | -1.474308 |
| H | 8.290126 | -0.518491 | -0.857288 |
| H | 8.889260 | -2.792791 | -0.043909 |
| C | 3.719171 | -0.513303 | 2.641103 |

| | | | |
|---|---|---|---|
| C | 0.893082 | 3.845913 | -0.327113 |
| C | 1.657024 | 4.520340 | -1.290338 |
| C | -0.338503 | 4.394621 | 0.086378 |
| C | 1.165984 | 5.713910 | -1.804957 |
| H | 2.606048 | 4.134184 | -1.628933 |
| C | -0.773535 | 5.581675 | -0.478450 |
| H | -0.947302 | 3.908992 | 0.841395 |
| C | -0.047094 | 6.280573 | -1.433438 |
| H | -0.402590 | 7.209746 | -1.860640 |
| F | 1.907226 | 6.353311 | -2.730722 |
| F | -1.969482 | 6.079711 | -0.084016 |
| C | 2.598795 | -0.532693 | 3.700054 |
| H | 1.928091 | -1.382051 | 3.554585 |
| H | 3.043705 | -0.604258 | 4.699840 |
| H | 2.006308 | 0.389810 | 3.673953 |
| C | 4.644704 | 0.688905 | 2.916496 |
| H | 5.494530 | 0.707160 | 2.225548 |
| H | 4.110973 | 1.639285 | 2.812356 |
| H | 5.038461 | 0.632684 | 3.938537 |
| C | 4.538154 | -1.814247 | 2.744892 |
| H | 5.350199 | -1.841496 | 2.010709 |
| H | 4.987456 | -1.891885 | 3.742036 |
| H | 3.909419 | -2.698859 | 2.594999 |

TS Conformation 83

B3LYP/6-31G(d) Energy = -3411.883827
M06-2X/def2tzvpp/IEFPCM(chloroform) Energy = -3411.887417
M06-2X/def2tzvpp/IEFPCM(chloroform)//B3LYP/6-31G(d) Quasiharmonic Free Energy = -3411.162707

Frequencies (Top 3 out of 291)

1. -978.4111 cm-1
2. 5.6938 cm-1
3. 7.2000 cm-1

B3LYP/6-31G(d) Molecular Geometry in Cartesian Coordinates

| | | | |
|---|---|---|---|
| C | -3.364925 | -1.578078 | 0.281753 |
| C | -3.146244 | -0.557869 | -0.858022 |
| H | -3.944633 | 0.180804 | -0.883806 |
| C | -1.829143 | 1.486133 | -0.175994 |
| N | -0.569579 | 1.802675 | 0.284356 |
| H | 0.040350 | 1.000112 | 0.464202 |
| N | -1.923997 | 0.167656 | -0.503712 |
| H | -1.118286 | -0.429182 | -0.323282 |
| C | -5.396465 | -0.284091 | 1.144960 |
| C | -4.471085 | -2.196855 | 2.371121 |
| C | -6.013725 | -0.379362 | 2.569397 |
| H | -4.839124 | 0.653697 | 1.052858 |
| C | -4.997027 | -1.183363 | 3.390160 |
| H | -5.150825 | -3.054718 | 2.270947 |
| H | -3.473219 | -2.582495 | 2.582438 |
| H | -6.966649 | -0.918025 | 2.520320 |
| H | -6.219693 | 0.615556 | 2.974600 |
| H | -5.440863 | -1.659090 | 4.270395 |
| H | -4.176830 | -0.539708 | 3.729332 |
| N | -4.431878 | -1.420957 | 1.118415 |
| O | -2.540693 | -2.481304 | 0.456916 |
| S | -3.109913 | 2.580045 | -0.320502 |
| C | -6.455362 | -0.329063 | 0.054323 |

| | | | |
|---|---|---|---|
| C | -7.170693 | -1.503999 | -0.219116 |
| C | -6.768270 | 0.832057 | -0.661783 |
| C | -8.176083 | -1.516076 | -1.184268 |
| H | -6.928774 | -2.417976 | 0.317789 |
| C | -7.777683 | 0.822455 | -1.628360 |
| C | -8.484654 | -0.350406 | -1.891702 |
| H | -8.717998 | -2.436155 | -1.387607 |
| O | 4.602062 | 2.091649 | -0.275546 |
| O | 3.729526 | -0.820673 | -3.382602 |
| C | 4.723761 | 0.911821 | 0.049390 |
| C | 4.340122 | -0.240802 | -0.791032 |
| O | 5.143051 | 0.530116 | 1.266392 |
| C | 5.442222 | 1.598193 | 2.191485 |
| H | 5.700161 | 1.098293 | 3.124733 |
| H | 6.287112 | 2.187701 | 1.825714 |
| H | 4.577144 | 2.249486 | 2.322285 |
| S | 3.985610 | 0.305561 | -2.462448 |
| C | 2.561725 | 1.414324 | -2.397367 |
| H | 1.712602 | 0.799068 | -2.092098 |
| H | 2.408004 | 1.794317 | -3.410062 |
| H | 2.761545 | 2.209138 | -1.678991 |
| C | 5.322731 | 1.336950 | -3.129351 |
| H | 5.478666 | 2.181487 | -2.458072 |
| H | 6.202404 | 0.691129 | -3.178196 |
| H | 5.024876 | 1.649077 | -4.133253 |
| C | 5.158996 | -1.500950 | -0.798921 |
| C | 6.550653 | -1.473629 | -0.599889 |
| C | 4.534168 | -2.735959 | -1.031794 |
| C | 7.293926 | -2.651681 | -0.616521 |
| H | 7.050129 | -0.527898 | -0.407548 |
| C | 5.282344 | -3.912686 | -1.059633 |
| H | 3.460936 | -2.770983 | -1.185162 |
| C | 6.661103 | -3.874931 | -0.850745 |
| H | 8.367207 | -2.614292 | -0.450380 |
| H | 4.782475 | -4.860544 | -1.237343 |
| H | 7.241190 | -4.793671 | -0.866398 |
| C | 2.854209 | -2.151724 | 2.327203 |
| C | 2.963453 | -3.085555 | 3.358818 |
| C | 1.906398 | -3.949487 | 3.649097 |
| C | 0.735050 | -3.874166 | 2.892591 |
| C | 0.617998 | -2.954168 | 1.850138 |
| C | 1.681090 | -2.084136 | 1.560766 |
| H | 3.678531 | -1.476496 | 2.120133 |
| H | 3.881155 | -3.131403 | 3.940604 |
| H | 1.994293 | -4.672522 | 4.455776 |
| H | -0.096780 | -4.540960 | 3.106646 |
| H | -0.296696 | -2.907657 | 1.266073 |
| S | 1.489408 | -0.900324 | 0.230227 |
| H | 3.006625 | -0.582795 | -0.278473 |
| H | -6.207024 | 1.743244 | -0.471254 |
| H | -8.004564 | 1.732311 | -2.177804 |
| H | -9.267750 | -0.360413 | -2.645186 |
| C | -3.057435 | -1.203880 | -2.286105 |
| C | 0.019231 | 3.019280 | 0.667766 |
| C | -0.414847 | 4.286838 | 0.251160 |
| C | 1.166723 | 2.909060 | 1.479001 |
| C | 0.300115 | 5.399967 | 0.674026 |
| H | -1.291001 | 4.409323 | -0.367442 |
| C | 1.835322 | 4.060719 | 1.856609 |
| H | 1.521583 | 1.940667 | 1.814924 |
| C | 1.433383 | 5.334760 | 1.476158 |
| H | 1.966872 | 6.223423 | 1.789225 |

| | | | |
|---|---|---|---|
| F | -0.122351 | 6.615809 | 0.270254 |
| F | 2.931334 | 3.941527 | 2.645020 |
| C | -3.068860 | -0.058977 | -3.318782 |
| H | -2.229073 | 0.626982 | -3.164397 |
| H | -2.989604 | -0.467334 | -4.333603 |
| H | -3.992949 | 0.526780 | -3.258972 |
| C | -4.293648 | -2.095644 | -2.511391 |
| H | -5.227012 | -1.537156 | -2.386607 |
| H | -4.278518 | -2.498359 | -3.531155 |
| H | -4.306996 | -2.946439 | -1.821143 |
| C | -1.783779 | -2.049235 | -2.483371 |
| H | -1.728234 | -2.867550 | -1.762915 |
| H | -1.781467 | -2.471290 | -3.495832 |
| H | -0.873974 | -1.446650 | -2.381268 |

TS Conformation 84

B3LYP/6-31G(d) Energy = -3411.883977
M06-2X/def2tzvpp/IEFPCM(chloroform) Energy = -3411.889537
M06-2X/def2tzvpp/IEFPCM(chloroform)//B3LYP/6-31G(d) Quasiharmonic Free Energy = -3411.162303

Frequencies (Top 3 out of 291)

1. -847.6879 cm-1
2. 11.2052 cm-1
3. 13.3042 cm-1

B3LYP/6-31G(d) Molecular Geometry in Cartesian Coordinates

| | | | |
|---|---|---|---|
| C | -2.389184 | -1.400006 | -0.700472 |
| C | -2.774195 | -0.121666 | -1.473172 |
| H | -3.625516 | 0.373208 | -1.008083 |
| C | -1.631091 | 2.003030 | -0.705810 |
| N | -0.349376 | 2.460197 | -0.530924 |
| H | 0.372058 | 1.782232 | -0.759528 |
| N | -1.628617 | 0.774355 | -1.316331 |
| H | -0.733455 | 0.295739 | -1.352925 |
| C | -4.066674 | -0.872147 | 1.137934 |
| C | -2.592566 | -2.842917 | 1.257104 |
| C | -3.915118 | -1.358320 | 2.602441 |
| H | -3.773938 | 0.178920 | 1.068371 |
| C | -3.532498 | -2.838514 | 2.467428 |
| H | -2.614728 | -3.771874 | 0.679772 |
| H | -1.554775 | -2.657962 | 1.559488 |
| H | -4.826630 | -1.191349 | 3.181562 |
| H | -3.099020 | -0.803286 | 3.079317 |
| H | -4.421441 | -3.448092 | 2.272798 |
| H | -3.044366 | -3.234832 | 3.362255 |
| N | -3.065448 | -1.713050 | 0.429376 |
| O | -1.428386 | -2.093883 | -1.083279 |
| S | -3.044438 | 2.813191 | -0.269097 |
| C | -5.488486 | -1.012811 | 0.610926 |
| C | -5.998059 | -2.223373 | 0.122970 |
| C | -6.338007 | 0.100827 | 0.665132 |
| C | -7.329068 | -2.321870 | -0.285884 |
| H | -5.347407 | -3.089152 | 0.039603 |
| C | -7.668594 | 0.004273 | 0.255970 |
| C | -8.169680 | -1.209393 | -0.217656 |
| H | -7.707161 | -3.268254 | -0.663747 |
| O | 3.264503 | 1.090889 | -1.822003 |
| O | 1.075310 | -0.128037 | -0.343577 |

| | | | |
|---|---|---|---|
| C | 4.033932 | 0.429224 | -1.133232 |
| C | 3.715752 | -0.887377 | -0.498685 |
| O | 5.266717 | 0.819612 | -0.801851 |
| C | 5.658102 | 2.135472 | -1.248316 |
| H | 5.032528 | 2.897386 | -0.777191 |
| H | 6.695254 | 2.243617 | -0.932907 |
| H | 5.571934 | 2.211362 | -2.334980 |
| S | 1.977313 | -1.215346 | -0.798718 |
| C | 1.625825 | -1.609542 | -2.532244 |
| H | 0.567858 | -1.881912 | -2.570749 |
| H | 2.274216 | -2.427546 | -2.852198 |
| H | 1.832896 | -0.696334 | -3.092216 |
| C | 1.580667 | -2.742043 | 0.064814 |
| H | 0.515763 | -2.900739 | -0.133227 |
| H | 1.763600 | -2.560951 | 1.127894 |
| H | 2.203532 | -3.554076 | -0.312877 |
| C | 4.611365 | -2.053192 | -0.854073 |
| C | 4.987220 | -2.988453 | 0.122772 |
| C | 5.091631 | -2.214171 | -2.166610 |
| C | 5.808077 | -4.066474 | -0.213316 |
| H | 4.668591 | -2.835803 | 1.150201 |
| C | 5.916352 | -3.287942 | -2.497222 |
| H | 4.830272 | -1.484424 | -2.929903 |
| C | 6.270805 | -4.221958 | -1.520606 |
| H | 6.097311 | -4.778196 | 0.555021 |
| H | 6.283977 | -3.393365 | -3.514256 |
| H | 6.913659 | -5.059716 | -1.776708 |
| C | 2.246746 | -2.117137 | 3.830438 |
| C | 0.984679 | -2.415377 | 4.347789 |
| C | -0.069695 | -1.509547 | 4.204100 |
| C | 0.155044 | -0.303205 | 3.533908 |
| C | 1.415812 | -0.002652 | 3.017792 |
| C | 2.491242 | -0.902629 | 3.156029 |
| H | 3.068640 | -2.815628 | 3.962737 |
| H | 0.829161 | -3.353593 | 4.876249 |
| H | -1.046115 | -1.730927 | 4.628338 |
| H | -0.650997 | 0.419197 | 3.424523 |
| H | 1.583917 | 0.943068 | 2.512325 |
| S | 4.116833 | -0.513076 | 2.537588 |
| H | 3.801137 | -0.689507 | 0.849054 |
| H | -5.948888 | 1.052513 | 1.019209 |
| H | -8.309864 | 0.880479 | 0.299320 |
| H | -9.204575 | -1.285715 | -0.539972 |
| C | -3.160662 | -0.364004 | -2.976240 |
| C | 0.207751 | 3.674017 | -0.088853 |
| C | -0.504563 | 4.751084 | 0.454736 |
| C | 1.608426 | 3.754932 | -0.221142 |
| C | 0.212656 | 5.874768 | 0.850007 |
| H | -1.577521 | 4.720866 | 0.569994 |
| C | 2.257534 | 4.901822 | 0.197455 |
| H | 2.181241 | 2.944948 | -0.661099 |
| C | 1.592902 | 5.993949 | 0.743062 |
| H | 2.117716 | 6.884270 | 1.065953 |
| F | -0.474937 | 6.909710 | 1.374130 |
| F | 3.603292 | 4.963325 | 0.061130 |
| C | -4.274712 | -1.425709 | -3.043203 |
| H | -5.148438 | -1.134435 | -2.451124 |
| H | -4.603236 | -1.556427 | -4.080983 |
| H | -3.927444 | -2.400985 | -2.682222 |
| C | -1.970508 | -0.825910 | -3.837315 |
| H | -1.603787 | -1.805743 | -3.521745 |
| H | -2.283374 | -0.898888 | -4.885769 |

| | | | |
|---|---|---|---|
| H | -1.144997 | -0.105707 | -3.794029 |
| C | -3.696038 | 0.966324 | -3.542663 |
| H | -4.576820 | 1.311958 | -2.991466 |
| H | -2.941289 | 1.757144 | -3.489329 |
| H | -3.979806 | 0.837438 | -4.594164 |

TS Conformation 85

B3LYP/6-31G(d) Energy = -3411.883977
M06-2X/def2tzvpp/IEFPCM(chloroform) Energy = -3411.889536
M06-2X/def2tzvpp/IEFPCM(chloroform)//B3LYP/6-31G(d) Quasiharmonic Free Energy = -3411.162301

Frequencies (Top 3 out of 291)

1. -847.8756 cm-1
2. 11.2097 cm-1
3. 13.3016 cm-1

B3LYP/6-31G(d) Molecular Geometry in Cartesian Coordinates

| | | | |
|---|---|---|---|
| C | -2.389162 | -1.399895 | -0.700471 |
| C | -2.774268 | -0.121610 | -1.473214 |
| H | -3.625582 | 0.373261 | -1.008111 |
| C | -1.631163 | 2.003086 | -0.705841 |
| N | -0.349433 | 2.460140 | -0.530827 |
| H | 0.371961 | 1.782074 | -0.759277 |
| N | -1.628722 | 0.774464 | -1.316458 |
| H | -0.733541 | 0.295913 | -1.353272 |
| C | -4.066644 | -0.872060 | 1.137945 |
| C | -2.592405 | -2.842731 | 1.257173 |
| C | -3.915031 | -1.358158 | 2.602470 |
| H | -3.773968 | 0.179020 | 1.068336 |
| C | -3.532301 | -2.838329 | 2.467527 |
| H | -2.614526 | -3.771719 | 0.679888 |
| H | -1.554617 | -2.657695 | 1.559516 |
| H | -4.826547 | -1.191227 | 3.181597 |
| H | -3.098968 | -0.803040 | 3.079307 |
| H | -4.421201 | -3.447991 | 2.272965 |
| H | -3.044106 | -3.234553 | 3.362362 |
| N | -3.065384 | -1.712936 | 0.429402 |
| O | -1.428322 | -2.093719 | -1.083272 |
| S | -3.044495 | 2.813292 | -0.269159 |
| C | -5.488456 | -1.012817 | 0.610960 |
| C | -5.998011 | -2.223455 | 0.123170 |
| C | -6.337993 | 0.100815 | 0.665012 |
| C | -7.329017 | -2.322027 | -0.285672 |
| H | -5.347348 | -3.089237 | 0.039919 |
| C | -7.668579 | 0.004186 | 0.255861 |
| C | -8.169646 | -1.209553 | -0.217599 |
| H | -7.707095 | -3.268469 | -0.663406 |
| O | 3.264483 | 1.090934 | -1.821947 |
| O | 1.075374 | -0.127927 | -0.343365 |
| C | 4.033893 | 0.429276 | -1.133151 |
| C | 3.715738 | -0.887373 | -0.498686 |
| O | 5.266648 | 0.819698 | -0.801692 |
| C | 5.658016 | 2.135578 | -1.248107 |
| H | 5.032358 | 2.897459 | -0.777038 |
| H | 6.695132 | 2.243770 | -0.932593 |
| H | 5.571953 | 2.211474 | -2.334778 |
| S | 1.977281 | -1.215265 | -0.798628 |
| C | 1.625633 | -1.609358 | -2.532146 |

| | | | |
|---|---|---|---|
| H | 2.274011 | -2.427328 | -2.852216 |
| H | 0.567669 | -1.881749 | -2.570564 |
| H | 1.832639 | -0.696111 | -3.092080 |
| C | 1.580629 | -2.741994 | 0.064840 |
| H | 0.515724 | -2.900682 | -0.133202 |
| H | 2.203493 | -3.554010 | -0.312889 |
| H | 1.763572 | -2.560946 | 1.127925 |
| C | 4.611320 | -2.053157 | -0.854250 |
| C | 5.091533 | -2.213982 | -2.166826 |
| C | 4.987200 | -2.988546 | 0.122465 |
| C | 5.916228 | -3.287723 | -2.497602 |
| H | 4.830145 | -1.484142 | -2.930020 |
| C | 5.808029 | -4.066537 | -0.213788 |
| H | 4.668621 | -2.836015 | 1.149926 |
| C | 6.270706 | -4.221865 | -1.521115 |
| H | 6.283810 | -3.393026 | -3.514663 |
| H | 6.097282 | -4.778358 | 0.554450 |
| H | 6.913538 | -5.059600 | -1.777345 |
| C | 1.416011 | -0.002729 | 3.017954 |
| C | 0.155230 | -0.303192 | 3.534093 |
| C | -0.069612 | -1.509566 | 4.204192 |
| C | 0.984669 | -2.415521 | 4.347764 |
| C | 2.246747 | -2.117372 | 3.830391 |
| C | 2.491346 | -0.902833 | 3.156075 |
| H | 1.584196 | 0.943014 | 2.512557 |
| H | -0.650743 | 0.419300 | 3.424796 |
| H | -1.046040 | -1.730876 | 4.628450 |
| H | 0.829071 | -3.353765 | 4.876151 |
| H | 3.068571 | -2.815962 | 3.962596 |
| S | 4.116965 | -0.513415 | 2.537612 |
| H | 3.801224 | -0.689646 | 0.849120 |
| H | -5.948888 | 1.052555 | 1.018959 |
| H | -8.309862 | 0.880388 | 0.299089 |
| H | -9.204539 | -1.285935 | -0.539906 |
| C | -3.160792 | -0.364026 | -2.976254 |
| C | 0.207748 | 3.673957 | -0.088808 |
| C | -0.504534 | 4.751076 | 0.454719 |
| C | 1.608431 | 3.754795 | -0.221054 |
| C | 0.212722 | 5.874746 | 0.849959 |
| H | -1.577496 | 4.720902 | 0.569957 |
| C | 2.257577 | 4.901673 | 0.197518 |
| H | 2.181226 | 2.944757 | -0.660937 |
| C | 1.592977 | 5.993856 | 0.743050 |
| H | 2.117823 | 6.884168 | 1.065918 |
| F | -0.474838 | 6.909743 | 1.374020 |
| F | 3.603343 | 4.963108 | 0.061239 |
| C | -1.970647 | -0.825877 | -3.837372 |
| H | -1.603799 | -1.805644 | -3.521746 |
| H | -2.283582 | -0.898980 | -4.885797 |
| H | -1.145210 | -0.105582 | -3.794212 |
| C | -3.696289 | 0.966249 | -3.542689 |
| H | -3.980039 | 0.837332 | -4.594190 |
| H | -4.577109 | 1.311800 | -2.991499 |
| H | -2.941616 | 1.757140 | -3.489349 |
| C | -4.274768 | -1.425813 | -3.043126 |
| H | -5.148478 | -1.134593 | -2.450997 |
| H | -4.603350 | -1.556572 | -4.080884 |
| H | -3.927406 | -2.401057 | -2.682151 |

TS Conformation 86

B3LYP/6-31G(d) Energy = -3411.884195
M06-2X/def2tzvpp/IEFPCM(chloroform) Energy = -3411.889519
M06-2X/def2tzvpp/IEFPCM(chloroform)//B3LYP/6-31G(d) Quasiharmonic Free Energy = -3411.162167

Frequencies (Top 3 out of 291)

1. -903.0099 cm-1
2. 11.5962 cm-1
3. 17.0157 cm-1

B3LYP/6-31G(d) Molecular Geometry in Cartesian Coordinates

| | | | |
|---|---|---|---|
| C | -2.307810 | -1.622837 | -0.563898 |
| C | -2.700875 | -0.480823 | -1.524279 |
| H | -3.567243 | 0.062493 | -1.149608 |
| C | -1.598501 | 1.750817 | -1.060688 |
| N | -0.324807 | 2.239669 | -0.902928 |
| H | 0.406958 | 1.540225 | -0.991272 |
| N | -1.569214 | 0.446836 | -1.483684 |
| H | -0.668456 | -0.020389 | -1.431724 |
| C | -3.945817 | -0.788032 | 1.193283 |
| C | -2.473293 | -2.717763 | 1.610758 |
| C | -3.765394 | -1.024730 | 2.714763 |
| H | -3.653319 | 0.235738 | 0.945452 |
| C | -3.393025 | -2.509816 | 2.818761 |
| H | -2.504726 | -3.729473 | 1.195618 |
| H | -1.430035 | -2.487949 | 1.859432 |
| H | -4.664283 | -0.757505 | 3.276011 |
| H | -2.934363 | -0.407243 | 3.073597 |
| H | -4.287029 | -3.137922 | 2.738422 |
| H | -2.892614 | -2.757151 | 3.759248 |
| N | -2.960281 | -1.739487 | 0.615616 |
| O | -1.359851 | -2.377820 | -0.849597 |
| S | -3.029654 | 2.606625 | -0.810477 |
| C | -5.376677 | -1.010064 | 0.721399 |
| C | -5.881590 | -2.278377 | 0.406723 |
| C | -6.238338 | 0.093345 | 0.648209 |
| C | -7.219692 | -2.441740 | 0.043290 |
| H | -5.222187 | -3.141364 | 0.424674 |
| C | -7.575670 | -0.068114 | 0.284969 |
| C | -8.072013 | -1.337914 | -0.015371 |
| H | -7.594101 | -3.432942 | -0.199183 |
| O | 3.414943 | 0.803907 | -1.729466 |
| O | 1.103679 | -0.335183 | -0.391756 |
| C | 4.107521 | 0.216482 | -0.904581 |
| C | 3.745439 | -1.065387 | -0.227086 |
| O | 5.285209 | 0.665702 | -0.462990 |
| C | 5.725591 | 1.930401 | -1.001083 |
| H | 6.707222 | 2.098311 | -0.559278 |
| H | 5.794525 | 1.877949 | -2.090642 |
| H | 5.035663 | 2.728824 | -0.718931 |
| S | 2.048700 | -1.445124 | -0.667529 |
| C | 1.867637 | -1.990809 | -2.385471 |
| H | 2.566107 | -2.807654 | -2.577814 |
| H | 2.099687 | -1.118749 | -2.998956 |
| H | 0.827046 | -2.303862 | -2.502169 |
| C | 1.598900 | -2.888416 | 0.303967 |
| H | 0.531315 | -3.034597 | 0.108136 |
| H | 1.794317 | -2.620648 | 1.347585 |
| H | 2.205103 | -3.743195 | 0.000860 |
| C | 4.692529 | -2.226320 | -0.435387 |
| C | 4.929281 | -3.159537 | 0.587114 |

| | | | |
|---|---|---|---|
| C | 5.365210 | -2.384466 | -1.661425 |
| C | 5.808617 | -4.224124 | 0.381525 |
| H | 4.441237 | -3.033190 | 1.549289 |
| C | 6.247049 | -3.444782 | -1.860972 |
| H | 5.206566 | -1.663263 | -2.460011 |
| C | 6.467192 | -4.371986 | -0.839542 |
| H | 5.986511 | -4.932372 | 1.186010 |
| H | 6.762167 | -3.546082 | -2.812296 |
| H | 7.154219 | -5.199522 | -0.993604 |
| C | 1.151050 | -0.782509 | 4.098896 |
| C | -0.073846 | -0.204138 | 4.435189 |
| C | -0.462251 | 1.012577 | 3.867763 |
| C | 0.394165 | 1.646194 | 2.963480 |
| C | 1.626885 | 1.079541 | 2.640693 |
| C | 2.029646 | -0.147601 | 3.200155 |
| H | 1.450490 | -1.725404 | 4.548519 |
| H | -0.722736 | -0.703049 | 5.152427 |
| H | -1.409960 | 1.470748 | 4.139049 |
| H | 0.108166 | 2.595390 | 2.517114 |
| H | 2.295454 | 1.598445 | 1.960123 |
| S | 3.623075 | -0.869543 | 2.852071 |
| H | 3.643659 | -0.856845 | 1.131916 |
| H | -5.853164 | 1.086635 | 0.866007 |
| H | -8.226438 | 0.800300 | 0.227672 |
| H | -9.112406 | -1.465127 | -0.302036 |
| C | -3.058485 | -0.952496 | -2.978028 |
| C | 0.205551 | 3.506052 | -0.600089 |
| C | -0.535869 | 4.632859 | -0.218648 |
| C | 1.610073 | 3.590759 | -0.690952 |
| C | 0.154939 | 5.806859 | 0.061057 |
| H | -1.612219 | 4.602671 | -0.141363 |
| C | 2.231111 | 4.789308 | -0.390154 |
| H | 2.207510 | 2.743051 | -1.011457 |
| C | 1.537084 | 5.931080 | -0.007546 |
| H | 2.041366 | 6.860926 | 0.223550 |
| F | -0.561942 | 6.887944 | 0.428523 |
| F | 3.581616 | 4.852096 | -0.477924 |
| C | -1.844760 | -1.507788 | -3.746875 |
| H | -1.456840 | -2.415531 | -3.278416 |
| H | -2.140715 | -1.749770 | -4.774704 |
| H | -1.040146 | -0.765184 | -3.806530 |
| C | -3.615238 | 0.264351 | -3.743754 |
| H | -2.880768 | 1.074053 | -3.797446 |
| H | -3.876803 | -0.026571 | -4.768382 |
| H | -4.513721 | 0.664926 | -3.262806 |
| C | -4.147402 | -2.039401 | -2.895923 |
| H | -3.779516 | -2.943207 | -2.396408 |
| H | -5.031923 | -1.686308 | -2.355033 |
| H | -4.465845 | -2.326218 | -3.905059 |

TS Conformation 87

B3LYP/6-31G(d) Energy = -3411.883839
M06-2X/def2tzvpp/IEFPCM(chloroform) Energy = -3411.886488
M06-2X/def2tzvpp/IEFPCM(chloroform)//B3LYP/6-31G(d) Quasiharmonic Free Energy = -3411.161849

Frequencies (Top 3 out of 291)

1. -987.3465 cm-1
2. 7.6517 cm-1
3. 9.9158 cm-1

B3LYP/6-31G(d) Molecular Geometry in Cartesian Coordinates

| | | | |
|---|---|---|---|
| C | 2.595361 | -1.403010 | 0.232140 |
| C | 2.811800 | -0.366000 | 1.353550 |
| H | 3.713130 | 0.219341 | 1.179900 |
| C | 1.762780 | 1.844410 | 0.767160 |
| N | 0.531740 | 2.333710 | 0.411700 |
| H | -0.229570 | 1.659700 | 0.347300 |
| N | 1.687300 | 0.566320 | 1.241980 |
| H | 0.783270 | 0.106480 | 1.181200 |
| C | 4.588610 | -0.528339 | -1.095440 |
| C | 3.181771 | -2.372240 | -1.922130 |
| C | 4.668470 | -0.623849 | -2.641130 |
| H | 4.288820 | 0.482831 | -0.807350 |
| C | 4.289701 | -2.080739 | -2.941830 |
| H | 3.152331 | -3.414760 | -1.590140 |
| H | 2.186181 | -2.126850 | -2.311390 |
| H | 5.655460 | -0.341609 | -3.016310 |
| H | 3.929260 | 0.057731 | -3.078600 |
| H | 5.149781 | -2.738809 | -2.776370 |
| H | 3.950181 | -2.229959 | -3.970990 |
| N | 3.491481 | -1.479229 | -0.786630 |
| O | 1.583251 | -2.117280 | 0.243960 |
| S | 3.198640 | 2.721680 | 0.639970 |
| C | 5.911850 | -0.852759 | -0.414390 |
| C | 6.785980 | 0.200231 | -0.110150 |
| C | 6.313451 | -2.163109 | -0.124350 |
| C | 8.036570 | -0.050118 | 0.455350 |
| H | 6.477550 | 1.223981 | -0.309180 |
| C | 7.564801 | -2.415709 | 0.441190 |
| C | 8.431891 | -1.360658 | 0.729720 |
| H | 8.697550 | 0.780072 | 0.689860 |
| O | -1.872530 | 0.403189 | 0.709030 |
| O | -1.174210 | 0.417409 | -2.457500 |
| C | -2.433110 | -0.671271 | 0.478300 |
| C | -2.960749 | -1.112811 | -0.837200 |
| O | -2.713409 | -1.571701 | 1.419600 |
| C | -2.424869 | -1.198591 | 2.781050 |
| H | -1.351080 | -1.058341 | 2.919350 |
| H | -2.961350 | -0.282991 | 3.038330 |
| H | -2.783959 | -2.031871 | 3.383270 |
| S | -2.577340 | 0.108719 | -2.075520 |
| C | -3.493030 | -0.470021 | -3.518190 |
| H | -3.058499 | -1.422221 | -3.824710 |
| H | -4.546440 | -0.587951 | -3.252360 |
| H | -3.356880 | 0.286179 | -4.294150 |
| C | -3.485000 | 1.603089 | -1.613720 |
| H | -3.034840 | 1.972349 | -0.693470 |
| H | -4.531510 | 1.317539 | -1.454460 |
| H | -3.357140 | 2.322689 | -2.425980 |
| C | -2.625779 | -2.521361 | -1.275030 |
| C | -3.632989 | -3.386261 | -1.730570 |
| C | -1.299779 | -2.983771 | -1.215640 |
| C | -3.316669 | -4.686091 | -2.128950 |
| H | -4.665309 | -3.049361 | -1.739590 |
| C | -0.991789 | -4.285471 | -1.608540 |
| H | -0.504439 | -2.342940 | -0.845380 |
| C | -1.996879 | -5.137411 | -2.071560 |
| H | -4.106088 | -5.348701 | -2.473560 |
| H | 0.036691 | -4.628650 | -1.542370 |
| H | -1.754618 | -6.152021 | -2.376980 |

| | | | |
|---|---|---|---|
| C | -6.300159 | -1.745662 | 1.925460 |
| C | -6.379499 | -1.606482 | 3.311180 |
| C | -6.294920 | -0.342812 | 3.901810 |
| C | -6.134250 | 0.781738 | 3.090190 |
| C | -6.057260 | 0.644838 | 1.702090 |
| C | -6.133870 | -0.621332 | 1.099480 |
| H | -6.368219 | -2.728842 | 1.469930 |
| H | -6.512709 | -2.489562 | 3.931830 |
| H | -6.361550 | -0.235502 | 4.981290 |
| H | -6.076620 | 1.772208 | 3.535510 |
| H | -5.950070 | 1.526038 | 1.075930 |
| S | -6.048720 | -0.809172 | -0.680740 |
| H | -4.396150 | -0.986451 | -0.758110 |
| H | 5.636471 | -2.989739 | -0.319910 |
| H | 7.858881 | -3.438658 | 0.661650 |
| H | 9.403981 | -1.557558 | 1.173710 |
| C | 2.929840 | -0.988000 | 2.788200 |
| C | 0.220559 | 3.608450 | -0.116400 |
| C | 0.590259 | 4.796740 | 0.523370 |
| C | -0.559971 | 3.642670 | -1.279000 |
| C | 0.161409 | 5.997030 | -0.027610 |
| H | 1.195239 | 4.792500 | 1.419680 |
| C | -0.983091 | 4.871879 | -1.763660 |
| H | -0.790320 | 2.730039 | -1.815820 |
| C | -0.639231 | 6.075660 | -1.164510 |
| H | -0.970421 | 7.027989 | -1.559970 |
| F | 0.510429 | 7.146170 | 0.583320 |
| F | -1.761111 | 4.889379 | -2.871200 |
| C | 3.423270 | 0.121460 | 3.737820 |
| H | 2.748610 | 0.983850 | 3.726150 |
| H | 3.475390 | -0.256389 | 4.766080 |
| H | 4.421180 | 0.475301 | 3.455400 |
| C | 3.961651 | -2.131379 | 2.762390 |
| H | 4.928851 | -1.796729 | 2.372510 |
| H | 4.124101 | -2.508669 | 3.778830 |
| H | 3.616381 | -2.971439 | 2.149060 |
| C | 1.583941 | -1.531000 | 3.305330 |
| H | 0.858530 | -0.718750 | 3.434310 |
| H | 1.163241 | -2.275570 | 2.624730 |
| H | 1.726681 | -1.995690 | 4.288230 |

TS Conformation 88

B3LYP/6-31G(d) Energy = -3411.883075
M06-2X/def2tzvpp/IEFPCM(chloroform) Energy = -3411.887073
M06-2X/def2tzvpp/IEFPCM(chloroform)//B3LYP/6-31G(d) Quasiharmonic Free Energy = -3411.161781

Frequencies (Top 3 out of 291)

1. -914.8372 cm-1
2. 6.2207 cm-1
3. 14.0113 cm-1

B3LYP/6-31G(d) Molecular Geometry in Cartesian Coordinates

| | | | |
|---|---|---|---|
| C | -3.053025 | -1.279463 | 0.569976 |
| C | -3.151693 | -0.489069 | -0.752218 |
| H | -3.955614 | 0.243882 | -0.710612 |
| C | -1.776938 | 1.623901 | -0.756905 |
| N | -0.458228 | 2.008102 | -0.692589 |
| H | 0.216392 | 1.254016 | -0.599495 |

| | | | |
|---|---|---|---|
| N | -1.906890 | 0.271792 | -0.863567 |
| H | -1.065934 | -0.295590 | -0.814699 |
| C | -4.820542 | 0.177999 | 1.693592 |
| C | -3.691811 | -1.655466 | 2.887047 |
| C | -4.840308 | 0.431411 | 3.223354 |
| H | -4.380535 | 1.040166 | 1.185108 |
| C | -4.687207 | -0.969894 | 3.831253 |
| H | -3.849393 | -2.734121 | 2.789341 |
| H | -2.651482 | -1.508659 | 3.201236 |
| H | -5.749491 | 0.949240 | 3.539541 |
| H | -3.980565 | 1.056891 | 3.491548 |
| H | -5.649456 | -1.493796 | 3.824189 |
| H | -4.326774 | -0.950269 | 4.863853 |
| N | -3.904759 | -0.983459 | 1.588149 |
| O | -2.178113 | -2.142388 | 0.707165 |
| S | -3.085794 | 2.690282 | -0.706503 |
| C | -6.210417 | -0.064418 | 1.118480 |
| C | -6.814302 | -1.328178 | 1.102713 |
| C | -6.935051 | 1.031513 | 0.628941 |
| C | -8.116126 | -1.489871 | 0.624054 |
| H | -6.257104 | -2.194774 | 1.446719 |
| C | -8.235528 | 0.872116 | 0.149936 |
| C | -8.832382 | -0.390120 | 0.149221 |
| H | -8.568125 | -2.478450 | 0.618165 |
| O | 5.421463 | -1.432247 | -1.652245 |
| O | 1.206358 | -0.440005 | -1.559986 |
| C | 4.770908 | -2.021141 | -0.790902 |
| C | 3.325460 | -1.812406 | -0.527172 |
| O | 5.308640 | -2.900367 | 0.064525 |
| C | 6.738388 | -3.060236 | -0.019824 |
| H | 7.231575 | -2.134537 | 0.286441 |
| H | 6.975217 | -3.869118 | 0.671097 |
| H | 7.038245 | -3.318242 | -1.038027 |
| S | 2.653855 | -0.697069 | -1.779506 |
| C | 3.568572 | 0.858062 | -1.735768 |
| H | 3.533059 | 1.185249 | -0.691718 |
| H | 4.590892 | 0.676178 | -2.061942 |
| H | 3.023159 | 1.549196 | -2.382772 |
| C | 2.889103 | -1.348419 | -3.451925 |
| H | 2.335488 | -2.289245 | -3.490944 |
| H | 2.467340 | -0.624555 | -4.153273 |
| H | 3.957757 | -1.503104 | -3.605687 |
| C | 2.429627 | -3.005805 | -0.338525 |
| C | 1.253186 | -2.892466 | 0.417575 |
| C | 2.734088 | -4.243471 | -0.935830 |
| C | 0.402134 | -3.987348 | 0.575363 |
| H | 0.997477 | -1.948568 | 0.885190 |
| C | 1.895129 | -5.340653 | -0.760416 |
| H | 3.644236 | -4.354645 | -1.519012 |
| C | 0.724394 | -5.212624 | -0.007976 |
| H | -0.517054 | -3.860497 | 1.135892 |
| H | 2.151451 | -6.292906 | -1.217162 |
| H | 0.064585 | -6.066689 | 0.119438 |
| C | 5.126111 | 1.909548 | 1.865994 |
| C | 6.469955 | 2.242510 | 2.046004 |
| C | 7.404146 | 1.265520 | 2.394140 |
| C | 6.975846 | -0.052394 | 2.571456 |
| C | 5.632565 | -0.390372 | 2.399027 |
| C | 4.684915 | 0.584413 | 2.034955 |
| H | 4.411171 | 2.683903 | 1.603715 |
| H | 6.785636 | 3.274844 | 1.913765 |
| H | 8.449229 | 1.528211 | 2.535200 |

| | | | |
|---|---|---|---|
| H | 7.688442 | -0.820957 | 2.864051 |
| H | 5.302586 | -1.412641 | 2.558417 |
| S | 2.962615 | 0.154619 | 1.824313 |
| H | 3.196578 | -0.983401 | 0.573071 |
| H | -6.470300 | 2.014673 | 0.615262 |
| H | -8.778661 | 1.733085 | -0.230817 |
| H | -9.844105 | -0.517056 | -0.226626 |
| C | -3.404008 | -1.388613 | -2.013499 |
| C | 0.121685 | 3.292286 | -0.616516 |
| C | -0.381663 | 4.404575 | -1.304515 |
| C | 1.306994 | 3.399320 | 0.130958 |
| C | 0.310773 | 5.604129 | -1.207621 |
| H | -1.289492 | 4.348381 | -1.886988 |
| C | 1.957343 | 4.623874 | 0.171104 |
| H | 1.696986 | 2.558543 | 0.700030 |
| C | 1.489049 | 5.755331 | -0.484756 |
| H | 2.008282 | 6.704163 | -0.430785 |
| F | -0.171493 | 6.675197 | -1.870019 |
| F | 3.099580 | 4.725831 | 0.888468 |
| C | -4.607593 | -2.310594 | -1.740569 |
| H | -5.496291 | -1.742246 | -1.445557 |
| H | -4.857346 | -2.875078 | -2.646750 |
| H | -4.386312 | -3.035769 | -0.949507 |
| C | -2.180492 | -2.249713 | -2.385479 |
| H | -1.316781 | -1.629036 | -2.651576 |
| H | -1.887216 | -2.908655 | -1.565516 |
| H | -2.421608 | -2.864614 | -3.261429 |
| C | -3.739883 | -0.454120 | -3.192976 |
| H | -4.650564 | 0.124207 | -3.000632 |
| H | -2.928022 | 0.255929 | -3.382671 |
| H | -3.897213 | -1.041002 | -4.106110 |

TS Conformation 89

B3LYP/6-31G(d) Energy = -3411.88267
M06-2X/def2tzvpp/IEFPCM(chloroform) Energy = -3411.888487
M06-2X/def2tzvpp/IEFPCM(chloroform)//B3LYP/6-31G(d) Quasiharmonic Free Energy = -3411.161722

Frequencies (Top 3 out of 291)

1. -942.0537 cm-1
2. 5.8656 cm-1
3. 12.6163 cm-1

B3LYP/6-31G(d) Molecular Geometry in Cartesian Coordinates

| | | | |
|---|---|---|---|
| C | -2.590442 | -1.254696 | 0.655249 |
| C | -2.750155 | -0.576995 | -0.720077 |
| H | -3.698042 | -0.045324 | -0.776571 |
| C | -1.905752 | 1.792314 | -0.753435 |
| N | -0.712998 | 2.475511 | -0.724346 |
| H | 0.110704 | 1.889674 | -0.612648 |
| N | -1.707098 | 0.442852 | -0.794884 |
| H | -0.756965 | 0.099889 | -0.675205 |
| C | -4.842161 | -0.383137 | 1.478177 |
| C | -3.371097 | -1.747085 | 2.907629 |
| C | -5.122217 | -0.099042 | 2.975931 |
| H | -4.618448 | 0.554634 | 0.962963 |
| C | -4.632489 | -1.365341 | 3.692235 |
| H | -3.184196 | -2.824213 | 2.863617 |
| H | -2.471910 | -1.280221 | 3.328052 |

| | | | |
|---|---|---|---|
| H | -6.176324 | 0.125310 | 3.156704 |
| H | -4.530427 | 0.768784 | 3.291008 |
| H | -5.385535 | -2.157382 | 3.623791 |
| H | -4.420342 | -1.201114 | 4.752644 |
| N | -3.606397 | -1.208009 | 1.550829 |
| O | -1.510978 | -1.795398 | 0.958224 |
| S | -3.439139 | 2.502351 | -0.728821 |
| C | -6.011381 | -1.059417 | 0.776056 |
| C | -6.910739 | -0.262282 | 0.054629 |
| C | -6.255437 | -2.436552 | 0.862110 |
| C | -8.033144 | -0.824050 | -0.555548 |
| H | -6.723764 | 0.805250 | -0.036528 |
| C | -7.377644 | -3.000173 | 0.253287 |
| C | -8.271944 | -2.195593 | -0.455141 |
| H | -8.715373 | -0.190301 | -1.115733 |
| O | 3.618487 | -2.285506 | 2.559758 |
| O | 1.327092 | 0.166657 | -0.125273 |
| C | 4.307696 | -1.496024 | 1.920978 |
| C | 3.737723 | -0.555157 | 0.912414 |
| O | 5.635991 | -1.524733 | 2.117368 |
| C | 6.591645 | -0.806410 | 1.305411 |
| H | 6.319835 | -0.835090 | 0.247698 |
| H | 6.685526 | 0.224472 | 1.654271 |
| H | 7.534187 | -1.334745 | 1.457413 |
| S | 1.944403 | -0.798239 | 0.831551 |
| C | 1.137366 | -0.616435 | 2.441406 |
| H | 1.641966 | -1.280340 | 3.142803 |
| H | 1.249440 | 0.433501 | 2.720900 |
| H | 0.088762 | -0.885785 | 2.282465 |
| C | 1.602422 | -2.489567 | 0.325704 |
| H | 2.100613 | -3.159504 | 1.025306 |
| H | 0.513948 | -2.581247 | 0.348367 |
| H | 2.006647 | -2.599088 | -0.682989 |
| C | 4.050553 | 0.912783 | 1.028992 |
| C | 4.091504 | 1.717931 | -0.121550 |
| C | 4.303176 | 1.503899 | 2.280256 |
| C | 4.380092 | 3.079119 | -0.018474 |
| H | 3.894856 | 1.273312 | -1.091716 |
| C | 4.601154 | 2.862595 | 2.377605 |
| H | 4.281351 | 0.892938 | 3.179910 |
| C | 4.632143 | 3.655234 | 1.228263 |
| H | 4.398323 | 3.695799 | -0.911847 |
| H | 4.798351 | 3.302851 | 3.351224 |
| H | 4.842162 | 4.717875 | 1.301686 |
| C | 4.819399 | -3.909630 | -0.847004 |
| C | 4.519264 | -5.272173 | -0.809544 |
| C | 3.724725 | -5.851306 | -1.801402 |
| C | 3.231109 | -5.048352 | -2.832275 |
| C | 3.517058 | -3.682777 | -2.865721 |
| C | 4.316595 | -3.085296 | -1.872115 |
| H | 5.458845 | -3.481514 | -0.080278 |
| H | 4.918192 | -5.884442 | -0.004086 |
| H | 3.497944 | -6.913744 | -1.774243 |
| H | 2.615764 | -5.485301 | -3.615534 |
| H | 3.125193 | -3.062296 | -3.666709 |
| S | 4.660226 | -1.336220 | -1.933340 |
| H | 4.139945 | -0.958871 | -0.365659 |
| H | -5.553261 | -3.079038 | 1.385576 |
| H | -7.549113 | -4.070840 | 0.327466 |
| H | -9.143100 | -2.635788 | -0.932550 |
| C | -2.704599 | -1.556785 | -1.947583 |
| C | -0.398799 | 3.847393 | -0.780514 |

| | | | |
|---|---|---|---|
| C | -1.274126 | 4.854213 | -1.210312 |
| C | 0.920415 | 4.178423 | -0.418358 |
| C | -0.802922 | 6.160503 | -1.252852 |
| H | -2.291337 | 4.638170 | -1.498382 |
| C | 1.328464 | 5.499916 | -0.495593 |
| H | 1.621994 | 3.421493 | -0.086085 |
| C | 0.490854 | 6.529378 | -0.905947 |
| H | 0.826829 | 7.557510 | -0.955725 |
| F | -1.649388 | 7.124447 | -1.665986 |
| F | 2.602115 | 5.803865 | -0.155066 |
| C | -3.794359 | -2.630950 | -1.775473 |
| H | -4.790194 | -2.187595 | -1.676757 |
| H | -3.806315 | -3.290169 | -2.651157 |
| H | -3.613244 | -3.258430 | -0.895011 |
| C | -1.340956 | -2.251348 | -2.120220 |
| H | -0.519572 | -1.532283 | -2.218272 |
| H | -1.120876 | -2.919359 | -1.284161 |
| H | -1.352464 | -2.851357 | -3.037592 |
| C | -3.010918 | -0.728299 | -3.212016 |
| H | -2.256995 | 0.048166 | -3.375646 |
| H | -3.988541 | -0.238365 | -3.143437 |
| H | -3.019164 | -1.380584 | -4.092979 |

TS Conformation 90

B3LYP/6-31G(d) Energy = -3411.885347
M06-2X/def2tzvpp/IEFPCM(chloroform) Energy = -3411.886479
M06-2X/def2tzvpp/IEFPCM(chloroform)//B3LYP/6-31G(d) Quasiharmonic Free Energy = -3411.161614

Frequencies (Top 3 out of 291)

1. -966.3227 cm-1
2. 10.0941 cm-1
3. 12.3232 cm-1

B3LYP/6-31G(d) Molecular Geometry in Cartesian Coordinates

| | | | |
|---|---|---|---|
| C | 2.669940 | -1.181964 | -0.470805 |
| C | 2.814789 | -0.472103 | 0.894308 |
| H | 3.695629 | 0.167706 | 0.909336 |
| C | 1.648214 | 1.758367 | 0.817068 |
| N | 0.370388 | 2.258350 | 0.815220 |
| H | -0.375390 | 1.570038 | 0.927401 |
| N | 1.658710 | 0.412075 | 1.028692 |
| H | 0.763688 | -0.069900 | 0.996190 |
| C | 4.737636 | -0.030328 | -1.430194 |
| C | 3.378829 | -1.589288 | -2.761415 |
| C | 4.867723 | 0.291818 | -2.940883 |
| H | 4.426943 | 0.869180 | -0.892271 |
| C | 4.514148 | -1.033131 | -3.630613 |
| H | 3.358600 | -2.682799 | -2.713178 |
| H | 2.391987 | -1.262743 | -3.110803 |
| H | 5.862837 | 0.665888 | -3.195359 |
| H | 4.134716 | 1.064303 | -3.202284 |
| H | 5.376430 | -1.708871 | -3.619269 |
| H | 4.207161 | -0.904116 | -4.672782 |
| N | 3.629911 | -1.016422 | -1.422432 |
| O | 1.666182 | -1.868392 | -0.698747 |
| S | 3.036436 | 2.695401 | 0.592872 |
| C | 6.040227 | -0.538451 | -0.825898 |
| C | 6.428174 | -1.883515 | -0.877543 |

| | | | |
|---|---|---|---|
| C | 6.912632 | 0.388011 | -0.237372 |
| C | 7.662745 | -2.289614 | -0.367432 |
| H | 5.751629 | -2.622639 | -1.296733 |
| C | 8.146585 | -0.015389 | 0.273657 |
| C | 8.527685 | -1.356842 | 0.207009 |
| H | 7.945214 | -3.338287 | -0.413756 |
| O | -6.285347 | -0.535899 | -0.397062 |
| O | -2.770199 | 1.151027 | -2.308242 |
| C | -5.283998 | -1.249921 | -0.416436 |
| C | -3.968909 | -0.857880 | -0.960193 |
| O | -5.246130 | -2.473315 | 0.135148 |
| C | -6.443774 | -2.894586 | 0.811702 |
| H | -6.678243 | -2.211272 | 1.631258 |
| H | -7.287173 | -2.928663 | 0.117100 |
| H | -6.219329 | -3.889233 | 1.195892 |
| S | -4.086239 | 0.723679 | -1.782156 |
| C | -5.306151 | 0.706947 | -3.124543 |
| H | -6.263087 | 0.387378 | -2.709320 |
| H | -5.353318 | 1.714377 | -3.545225 |
| H | -4.931317 | -0.001450 | -3.866483 |
| C | -4.726196 | 1.945660 | -0.612368 |
| H | -5.794150 | 1.787986 | -0.474894 |
| H | -4.476790 | 2.922941 | -1.033869 |
| H | -4.189429 | 1.785201 | 0.327838 |
| C | -3.164265 | -1.846116 | -1.761269 |
| C | -1.765016 | -1.873969 | -1.660936 |
| C | -3.796181 | -2.757996 | -2.627200 |
| C | -1.015863 | -2.797412 | -2.392335 |
| H | -1.253956 | -1.187393 | -0.996301 |
| C | -3.049484 | -3.678964 | -3.358084 |
| H | -4.880566 | -2.761016 | -2.710062 |
| C | -1.656582 | -3.699199 | -3.242060 |
| H | 0.062465 | -2.803926 | -2.267832 |
| H | -3.554442 | -4.381905 | -4.015576 |
| H | -1.075871 | -4.422178 | -3.809181 |
| C | -2.514114 | -2.521091 | 2.328950 |
| C | -2.685210 | -3.498900 | 3.309649 |
| C | -2.833347 | -3.141498 | 4.651480 |
| C | -2.814539 | -1.791236 | 5.005753 |
| C | -2.657622 | -0.806965 | 4.029370 |
| C | -2.503946 | -1.161582 | 2.680010 |
| H | -2.385370 | -2.811400 | 1.291167 |
| H | -2.691145 | -4.547308 | 3.021019 |
| H | -2.959067 | -3.906832 | 5.412694 |
| H | -2.928541 | -1.498497 | 6.046692 |
| H | -2.655998 | 0.242930 | 4.307376 |
| S | -2.283116 | 0.122758 | 1.454286 |
| H | -3.157549 | -0.452966 | 0.211515 |
| H | 6.616368 | 1.432649 | -0.173175 |
| H | 8.805886 | 0.717460 | 0.731454 |
| H | 9.486672 | -1.674153 | 0.607800 |
| C | 2.923757 | -1.457614 | 2.111826 |
| C | -0.104386 | 3.554240 | 0.527012 |
| C | 0.483287 | 4.724107 | 1.024115 |
| C | -1.278526 | 3.626116 | -0.240150 |
| C | -0.125887 | 5.937897 | 0.732527 |
| H | 1.386928 | 4.695416 | 1.615759 |
| C | -1.849257 | 4.866333 | -0.472958 |
| H | -1.708153 | 2.736416 | -0.685469 |
| C | -1.300968 | 6.052568 | -0.004620 |
| H | -1.756454 | 7.014689 | -0.203693 |
| F | 0.434285 | 7.067072 | 1.211213 |

| | | | |
|---|---|---|---|
| F | -2.995051 | 4.923022 | -1.202298 |
| C | 3.296226 | -0.622995 | 3.353483 |
| H | 2.547489 | 0.150912 | 3.551677 |
| H | 3.356738 | -1.268560 | 4.237753 |
| H | 4.267177 | -0.129343 | 3.228218 |
| C | 4.043262 | -2.478717 | 1.836323 |
| H | 3.794836 | -3.131743 | 0.991926 |
| H | 4.998262 | -1.988738 | 1.619638 |
| H | 4.186619 | -3.117422 | 2.715775 |
| C | 1.608330 | -2.214254 | 2.385212 |
| H | 0.799102 | -1.537809 | 2.682285 |
| H | 1.279093 | -2.778645 | 1.509720 |
| H | 1.757563 | -2.914546 | 3.215750 |

TS Conformation 91

B3LYP/6-31G(d) Energy = -3411.87388
M06-2X/def2tzvpp/IEFPCM(chloroform) Energy = -3411.885605
M06-2X/def2tzvpp/IEFPCM(chloroform)//B3LYP/6-31G(d) Quasiharmonic Free Energy = -3411.161519

Frequencies (Top 3 out of 291)

1. -911.6058 cm-1
2. 4.8501 cm-1
3. 7.8472 cm-1

B3LYP/6-31G(d) Molecular Geometry in Cartesian Coordinates

| | | | |
|---|---|---|---|
| C | -4.262703 | 0.030886 | -1.194133 |
| C | -2.862408 | -0.306333 | -1.774198 |
| H | -2.909398 | 0.160845 | -2.761159 |
| C | -1.345245 | 1.593176 | -1.184541 |
| N | -0.196399 | 1.846812 | -0.456512 |
| H | 0.178979 | 1.038330 | 0.047193 |
| N | -1.723529 | 0.286742 | -1.080720 |
| H | -1.161453 | -0.325266 | -0.497382 |
| C | -5.841031 | 0.653890 | 0.546982 |
| C | -3.494823 | 0.433596 | 1.230718 |
| C | -5.606785 | 1.342059 | 1.914079 |
| H | -6.215359 | 1.366227 | -0.193288 |
| C | -4.371781 | 0.626618 | 2.475418 |
| H | -2.828357 | -0.428481 | 1.323822 |
| H | -2.884100 | 1.326929 | 1.054949 |
| H | -6.482572 | 1.273407 | 2.564511 |
| H | -5.383692 | 2.403687 | 1.753070 |
| H | -4.652673 | -0.343178 | 2.901008 |
| H | -3.856425 | 1.197716 | 3.253878 |
| N | -4.474661 | 0.246235 | 0.144253 |
| O | -5.204175 | 0.054338 | -1.982827 |
| S | -2.220097 | 2.709324 | -2.086700 |
| C | -6.834559 | -0.499596 | 0.622120 |
| C | -6.468089 | -1.786469 | 1.033609 |
| C | -8.181844 | -0.253410 | 0.328584 |
| C | -7.422536 | -2.798459 | 1.154025 |
| H | -5.426024 | -2.005749 | 1.247558 |
| C | -9.139366 | -1.259920 | 0.450351 |
| C | -8.762728 | -2.538554 | 0.865933 |
| H | -7.115796 | -3.792423 | 1.470157 |
| O | 4.726387 | 1.284248 | -0.573639 |
| O | 2.993754 | -1.908609 | -2.971368 |
| C | 4.593334 | 0.199227 | -0.010800 |

| | | | |
|---|---|---|---|
| C | 3.958961 | -0.997878 | -0.605263 |
| O | 4.947783 | -0.011098 | 1.264611 |
| C | 5.472251 | 1.130677 | 1.974345 |
| H | 4.760903 | 1.957505 | 1.952339 |
| H | 5.625800 | 0.779781 | 2.994360 |
| H | 6.417844 | 1.452150 | 1.529398 |
| S | 3.611850 | -0.725523 | -2.335532 |
| C | 2.535965 | 0.712231 | -2.501835 |
| H | 1.607205 | 0.471496 | -1.981939 |
| H | 3.020897 | 1.579431 | -2.056284 |
| H | 2.348259 | 0.834674 | -3.571208 |
| C | 5.111790 | -0.255261 | -3.243133 |
| H | 5.790923 | -1.107343 | -3.166860 |
| H | 4.831562 | -0.082382 | -4.284979 |
| H | 5.529530 | 0.635712 | -2.772482 |
| C | 4.564782 | -2.358461 | -0.389673 |
| C | 3.753551 | -3.505130 | -0.417067 |
| C | 5.947053 | -2.518955 | -0.180722 |
| C | 4.309531 | -4.772271 | -0.237330 |
| H | 2.686334 | -3.401221 | -0.575431 |
| C | 6.497305 | -3.784078 | 0.012291 |
| H | 6.592517 | -1.645766 | -0.147175 |
| C | 5.679721 | -4.916347 | -0.019622 |
| H | 3.665723 | -5.646973 | -0.262399 |
| H | 7.565750 | -3.885951 | 0.182392 |
| H | 6.109453 | -5.903544 | 0.126853 |
| C | 0.754732 | -0.050823 | 3.235091 |
| C | 1.034465 | 0.110002 | 4.593451 |
| C | 2.101038 | -0.572597 | 5.178814 |
| C | 2.881136 | -1.425402 | 4.394105 |
| C | 2.600127 | -1.599785 | 3.039186 |
| C | 1.535379 | -0.906532 | 2.442581 |
| H | -0.076077 | 0.488185 | 2.787752 |
| H | 0.417561 | 0.776246 | 5.191092 |
| H | 2.320380 | -0.444907 | 6.235252 |
| H | 3.710437 | -1.969038 | 4.840092 |
| H | 3.204435 | -2.275195 | 2.442657 |
| S | 1.131404 | -1.141973 | 0.712341 |
| H | 2.576227 | -1.042497 | 0.032455 |
| H | -8.480601 | 0.736235 | -0.010267 |
| H | -10.178235 | -1.048028 | 0.210503 |
| H | -9.505625 | -3.326677 | 0.956021 |
| C | -2.637697 | -1.845750 | -2.000396 |
| C | 0.561441 | 3.011096 | -0.241029 |
| C | 0.428605 | 4.207748 | -0.961928 |
| C | 1.552127 | 2.910135 | 0.758379 |
| C | 1.280194 | 5.261560 | -0.653163 |
| H | -0.321951 | 4.321601 | -1.729567 |
| C | 2.371478 | 3.997563 | 1.006086 |
| H | 1.671837 | 2.006631 | 1.346655 |
| C | 2.270268 | 5.201042 | 0.319738 |
| H | 2.919431 | 6.041049 | 0.532460 |
| F | 1.145169 | 6.407698 | -1.350235 |
| F | 3.317750 | 3.883309 | 1.968640 |
| C | -2.580973 | -2.630118 | -0.673265 |
| H | -3.507891 | -2.511254 | -0.102171 |
| H | -2.456246 | -3.699328 | -0.880497 |
| H | -1.737774 | -2.336011 | -0.036014 |
| C | -1.316614 | -2.044055 | -2.775165 |
| H | -0.435978 | -1.737845 | -2.200834 |
| H | -1.186793 | -3.103446 | -3.023983 |
| H | -1.323360 | -1.478024 | -3.714190 |

| | | | |
|---|---|---|---|
| C | -3.784010 | -2.414136 | -2.862827 |
| H | -3.875629 | -1.872569 | -3.810123 |
| H | -3.576112 | -3.466997 | -3.088492 |
| H | -4.750781 | -2.352700 | -2.359240 |

TS Conformation 92

B3LYP/6-31G(d) Energy = -3411.87388
M06-2X/def2tzvpp/IEFPCM(chloroform) Energy = -3411.885606
M06-2X/def2tzvpp/IEFPCM(chloroform)//B3LYP/6-31G(d) Quasiharmonic Free Energy = -3411.161515

Frequencies (Top 3 out of 291)

1. -911.6738 cm-1
2. 4.8673 cm-1
3. 7.8618 cm-1

B3LYP/6-31G(d) Molecular Geometry in Cartesian Coordinates

| | | | |
|---|---|---|---|
| C | -4.263072 | 0.032148 | -1.194273 |
| C | -2.862880 | -0.304483 | -1.774920 |
| H | -2.910044 | 0.163671 | -2.761412 |
| C | -1.345421 | 1.594350 | -1.183866 |
| N | -0.196285 | 1.847165 | -0.456006 |
| H | 0.178981 | 1.038276 | 0.047127 |
| N | -1.723865 | 0.287889 | -1.081070 |
| H | -1.161614 | -0.324735 | -0.498550 |
| C | -5.841091 | 0.653593 | 0.547669 |
| C | -3.494732 | 0.432890 | 1.230758 |
| C | -5.606607 | 1.340728 | 1.915244 |
| H | -6.215632 | 1.366470 | -0.191973 |
| C | -4.371449 | 0.624925 | 2.475783 |
| H | -2.828233 | -0.429246 | 1.323064 |
| H | -2.884060 | 1.326371 | 1.055558 |
| H | -6.482258 | 1.271532 | 2.565801 |
| H | -5.383603 | 2.402490 | 1.754999 |
| H | -4.652200 | -0.345205 | 2.900702 |
| H | -3.855961 | 1.195462 | 3.254567 |
| N | -4.474776 | 0.246357 | 0.144334 |
| O | -5.204705 | 0.056214 | -1.982760 |
| S | -2.220436 | 2.711470 | -2.084657 |
| C | -6.834508 | -0.500031 | 0.622128 |
| C | -6.467848 | -1.787186 | 1.032570 |
| C | -8.181871 | -0.253738 | 0.329042 |
| C | -7.422186 | -2.799347 | 1.152406 |
| H | -5.425724 | -2.006540 | 1.246148 |
| C | -9.139284 | -1.260421 | 0.450234 |
| C | -8.762457 | -2.539338 | 0.864773 |
| H | -7.115301 | -3.793525 | 1.467726 |
| O | 4.726858 | 1.284454 | -0.571152 |
| O | 2.995528 | -1.906337 | -2.972579 |
| C | 4.593465 | 0.198960 | -0.009307 |
| C | 3.959375 | -0.997620 | -0.605131 |
| O | 4.947196 | -0.012461 | 1.266121 |
| C | 5.471241 | 1.128694 | 1.977163 |
| H | 4.760090 | 1.955689 | 1.955107 |
| H | 5.623789 | 0.777019 | 2.997060 |
| H | 6.417310 | 1.450292 | 1.533317 |
| S | 3.613257 | -0.723799 | -2.335370 |
| C | 2.537455 | 0.714090 | -2.501022 |
| H | 1.608397 | 0.472847 | -1.981897 |

| | | | |
|---|---|---|---|
| H | 3.022103 | 1.580889 | -2.054384 |
| H | 2.350395 | 0.837511 | -3.570396 |
| C | 5.113706 | -0.252734 | -3.241713 |
| H | 5.531181 | 0.637819 | -2.770033 |
| H | 5.792795 | -1.104886 | -3.165823 |
| H | 4.834061 | -0.078925 | -4.283562 |
| C | 4.564992 | -2.358416 | -0.390324 |
| C | 5.947134 | -2.519169 | -0.180721 |
| C | 3.753704 | -3.505008 | -0.419129 |
| C | 6.497196 | -3.784488 | 0.011553 |
| H | 6.592633 | -1.646049 | -0.146081 |
| C | 4.309500 | -4.772334 | -0.240128 |
| H | 2.686583 | -3.400897 | -0.578013 |
| C | 5.679558 | -4.916678 | -0.021763 |
| H | 7.565538 | -3.886571 | 0.182177 |
| H | 3.665651 | -5.646974 | -0.266290 |
| H | 6.109144 | -5.904023 | 0.124135 |
| C | 0.753295 | -0.053171 | 3.234166 |
| C | 1.032363 | 0.106649 | 4.592783 |
| C | 2.098538 | -0.576521 | 5.178202 |
| C | 2.878908 | -1.428885 | 4.393281 |
| C | 2.598560 | -1.602264 | 3.038097 |
| C | 1.534217 | -0.908430 | 2.441441 |
| H | -0.077209 | 0.486278 | 2.786794 |
| H | 0.415253 | 0.772558 | 5.190585 |
| H | 2.317363 | -0.449615 | 6.234841 |
| H | 3.707901 | -1.972962 | 4.839303 |
| H | 3.203072 | -2.277336 | 2.441393 |
| S | 1.131102 | -1.142578 | 0.710825 |
| H | 2.576295 | -1.042669 | 0.031777 |
| H | -8.480779 | 0.736137 | -0.009001 |
| H | -10.178218 | -1.048437 | 0.210748 |
| H | -9.505270 | -3.327592 | 0.954410 |
| C | -2.638200 | -1.843680 | -2.002660 |
| C | 0.561821 | 3.011157 | -0.239894 |
| C | 0.429769 | 4.207940 | -0.960721 |
| C | 1.552035 | 2.909678 | 0.759921 |
| C | 1.281617 | 5.261392 | -0.651448 |
| H | -0.320431 | 4.322188 | -1.728650 |
| C | 2.371715 | 3.996754 | 1.008104 |
| H | 1.671136 | 2.006049 | 1.348129 |
| C | 2.271251 | 5.200360 | 0.321870 |
| H | 2.920643 | 6.040092 | 0.534983 |
| F | 1.147353 | 6.407665 | -1.348445 |
| F | 3.317538 | 3.882019 | 1.971040 |
| C | -2.581420 | -2.629348 | -0.676301 |
| H | -3.508340 | -2.511097 | -0.105083 |
| H | -2.456626 | -3.698346 | -0.884578 |
| H | -1.738238 | -2.335804 | -0.038768 |
| C | -1.317153 | -2.041240 | -2.777687 |
| H | -0.436477 | -1.735704 | -2.203053 |
| H | -1.187416 | -3.100375 | -3.027640 |
| H | -1.323887 | -1.474202 | -3.716104 |
| C | -3.784555 | -2.411206 | -2.865599 |
| H | -3.876166 | -1.868747 | -3.812386 |
| H | -3.576710 | -3.463864 | -3.092259 |
| H | -4.751311 | -2.350192 | -2.361933 |

TS Conformation 93

B3LYP/6-31G(d) Energy = -3411.880146

M06-2X/def2tzvpp/IEFPCM(chloroform) Energy = -3411.888074
M06-2X/def2tzvpp/IEFPCM(chloroform)//B3LYP/6-31G(d) Quasiharmonic Free Energy = -3411.161489

Frequencies (Top 3 out of 291)

1. -990.0063 cm-1
2. 8.8377 cm-1
3. 10.7984 cm-1

B3LYP/6-31G(d) Molecular Geometry in Cartesian Coordinates

| | | | |
|---|---|---|---|
| C | -2.682433 | -1.373996 | 0.545664 |
| C | -2.757170 | -0.653326 | -0.814617 |
| H | -3.610345 | 0.022605 | -0.845333 |
| C | -1.541772 | 1.550708 | -0.979412 |
| N | -0.258855 | 2.035454 | -0.909603 |
| H | 0.462449 | 1.325996 | -0.819779 |
| N | -1.561433 | 0.185990 | -0.877707 |
| H | -0.714998 | -0.257467 | -0.529008 |
| C | -4.593601 | 0.029223 | 1.477669 |
| C | -3.446467 | -1.669272 | 2.842697 |
| C | -4.749371 | 0.345271 | 2.987419 |
| H | -4.164814 | 0.892961 | 0.962479 |
| C | -4.558437 | -1.015301 | 3.671923 |
| H | -3.515063 | -2.760022 | 2.790036 |
| H | -2.450902 | -1.423381 | 3.233218 |
| H | -5.711636 | 0.814200 | 3.207042 |
| H | -3.955430 | 1.037605 | 3.292479 |
| H | -5.480341 | -1.603523 | 3.613177 |
| H | -4.286322 | -0.928653 | 4.727831 |
| N | -3.600708 | -1.077400 | 1.498451 |
| O | -1.760360 | -2.174835 | 0.781913 |
| S | -2.931362 | 2.483863 | -1.202087 |
| C | -5.915218 | -0.325350 | 0.809221 |
| C | -6.468709 | -1.611350 | 0.859874 |
| C | -6.631882 | 0.689947 | 0.160714 |
| C | -7.716125 | -1.872165 | 0.290044 |
| H | -5.913622 | -2.420816 | 1.325251 |
| C | -7.878443 | 0.430780 | -0.409984 |
| C | -8.426675 | -0.851310 | -0.343557 |
| H | -8.129261 | -2.876404 | 0.336205 |
| O | 3.641433 | -3.427872 | 1.793220 |
| O | 0.985972 | -0.250838 | 0.648443 |
| C | 4.203766 | -2.350734 | 1.619436 |
| C | 3.471900 | -1.098832 | 1.278617 |
| O | 5.544737 | -2.323162 | 1.708886 |
| C | 6.346721 | -1.171425 | 1.370080 |
| H | 5.972296 | -0.668564 | 0.475601 |
| H | 6.386028 | -0.476022 | 2.211843 |
| H | 7.342657 | -1.573255 | 1.177036 |
| S | 1.719764 | -1.475048 | 1.072944 |
| C | 1.488902 | -2.783794 | -0.140672 |
| H | 2.018983 | -3.672411 | 0.196921 |
| H | 0.406312 | -2.923186 | -0.192165 |
| H | 1.893707 | -2.412948 | -1.084962 |
| C | 0.963409 | -2.119894 | 2.582751 |
| H | -0.087954 | -2.300029 | 2.337506 |
| H | 1.504021 | -3.023930 | 2.864726 |
| H | 1.070817 | -1.336185 | 3.335903 |
| C | 3.626929 | 0.090641 | 2.191817 |
| C | 3.411073 | 1.392597 | 1.710518 |
| C | 3.981359 | -0.077831 | 3.544056 |

| | | | |
|---|---|---|---|
| C | 3.551687 | 2.493001 | 2.555994 |
| H | 3.132708 | 1.542579 | 0.674160 |
| C | 4.137088 | 1.025201 | 4.382137 |
| H | 4.151805 | -1.077362 | 3.937380 |
| C | 3.918130 | 2.314469 | 3.890538 |
| H | 3.378970 | 3.491217 | 2.164059 |
| H | 4.422398 | 0.876465 | 5.420063 |
| H | 4.033994 | 3.174052 | 4.544942 |
| C | 4.566266 | -2.657316 | -2.170674 |
| C | 4.350450 | -3.822223 | -2.908803 |
| C | 3.453526 | -3.827349 | -3.979062 |
| C | 2.771195 | -2.652017 | -4.302209 |
| C | 2.973410 | -1.488428 | -3.558307 |
| C | 3.874927 | -1.469776 | -2.477194 |
| H | 5.283902 | -2.663909 | -1.355046 |
| H | 4.894856 | -4.727087 | -2.649365 |
| H | 3.291958 | -4.733418 | -4.556647 |
| H | 2.073928 | -2.638762 | -5.136569 |
| H | 2.436760 | -0.578684 | -3.812300 |
| S | 4.118088 | 0.024507 | -1.532556 |
| H | 3.794905 | -0.635375 | -0.047546 |
| H | -6.202352 | 1.686547 | 0.092231 |
| H | -8.415761 | 1.229137 | -0.914710 |
| H | -9.395564 | -1.055949 | -0.790832 |
| C | -2.902457 | -1.611887 | -2.050915 |
| C | 0.276247 | 3.337304 | -0.991157 |
| C | -0.438431 | 4.506910 | -0.699997 |
| C | 1.638842 | 3.413647 | -1.338234 |
| C | 0.231441 | 5.721309 | -0.777674 |
| H | -1.483626 | 4.481045 | -0.432371 |
| C | 2.248312 | 4.658529 | -1.387446 |
| H | 2.214557 | 2.526651 | -1.590239 |
| C | 1.573532 | 5.843077 | -1.114183 |
| H | 2.065489 | 6.806341 | -1.166939 |
| F | -0.459122 | 6.845049 | -0.495100 |
| F | 3.551663 | 4.725266 | -1.722962 |
| C | -3.139843 | -0.734663 | -3.296724 |
| H | -2.297926 | -0.059004 | -3.477560 |
| H | -3.259656 | -1.369879 | -4.182497 |
| H | -4.041227 | -0.121711 | -3.191856 |
| C | -4.126493 | -2.522050 | -1.835177 |
| H | -4.283143 | -3.151176 | -2.719165 |
| H | -5.040518 | -1.941504 | -1.672425 |
| H | -3.988959 | -3.190216 | -0.976758 |
| C | -1.659140 | -2.487396 | -2.289682 |
| H | -1.497500 | -3.187675 | -1.466693 |
| H | -0.756287 | -1.879864 | -2.420796 |
| H | -1.792937 | -3.069234 | -3.209384 |

TS Conformation 94

B3LYP/6-31G(d) Energy = -3411.87898
M06-2X/def2tzvpp/IEFPCM(chloroform) Energy = -3411.886319
M06-2X/def2tzvpp/IEFPCM(chloroform)//B3LYP/6-31G(d) Quasiharmonic Free Energy = -3411.161269

Frequencies (Top 3 out of 291)

1. -756.7083 cm-1
2. 5.4301 cm-1
3. 9.2530 cm-1

B3LYP/6-31G(d) Molecular Geometry in Cartesian Coordinates

| | | | |
|---|---|---|---|
| C | -3.453362 | -1.604227 | -0.717904 |
| C | -3.607545 | -0.371395 | -1.628596 |
| H | -4.553761 | 0.133639 | -1.440087 |
| C | -2.786305 | 1.759435 | -0.586311 |
| N | -1.605127 | 2.352881 | -0.218104 |
| H | -0.770154 | 1.852865 | -0.515571 |
| N | -2.566406 | 0.570686 | -1.231822 |
| H | -1.631221 | 0.173677 | -1.172142 |
| C | -4.360456 | -3.118573 | 0.980097 |
| C | -5.845328 | -1.353991 | 0.118416 |
| C | -5.834922 | -3.442143 | 1.322106 |
| H | -3.891230 | -3.936233 | 0.424926 |
| C | -6.518503 | -2.067364 | 1.299975 |
| H | -5.789205 | -0.270107 | 0.252744 |
| H | -6.379698 | -1.559523 | -0.818263 |
| H | -5.930935 | -3.959816 | 2.279866 |
| H | -6.256665 | -4.087594 | 0.541678 |
| H | -6.314001 | -1.528375 | 2.231282 |
| H | -7.603882 | -2.126471 | 1.178698 |
| N | -4.494552 | -1.957115 | 0.078152 |
| O | -2.380798 | -2.222307 | -0.689113 |
| S | -4.331636 | 2.393502 | -0.328898 |
| C | -3.508444 | -2.837555 | 2.214352 |
| C | -3.338395 | -1.549954 | 2.736820 |
| C | -2.909592 | -3.915522 | 2.879902 |
| C | -2.592543 | -1.346797 | 3.901026 |
| H | -3.774944 | -0.696560 | 2.225539 |
| C | -2.165806 | -3.716515 | 4.042663 |
| C | -2.005340 | -2.428462 | 4.559216 |
| H | -2.471877 | -0.339757 | 4.291686 |
| O | 0.466368 | 0.299653 | -0.975617 |
| O | 2.370898 | 2.338059 | -1.986084 |
| C | 1.434542 | -0.314033 | -0.516405 |
| C | 2.813758 | -0.248429 | -1.073310 |
| O | 1.394638 | -1.084149 | 0.561267 |
| C | 0.122467 | -1.203241 | 1.250249 |
| H | 0.330500 | -1.827328 | 2.117444 |
| H | -0.217380 | -0.215119 | 1.567706 |
| H | -0.620625 | -1.670985 | 0.601977 |
| S | 2.829842 | 0.993458 | -2.385197 |
| C | 1.933469 | 0.423257 | -3.855855 |
| H | 2.297097 | -0.566669 | -4.138168 |
| H | 0.881174 | 0.391251 | -3.570986 |
| H | 2.102372 | 1.156346 | -4.648283 |
| C | 4.552375 | 1.060540 | -2.911027 |
| H | 5.124617 | 1.296455 | -2.004382 |
| H | 4.863336 | 0.106349 | -3.337235 |
| H | 4.612680 | 1.872635 | -3.639114 |
| C | 3.439198 | -1.562359 | -1.483298 |
| C | 2.659378 | -2.566920 | -2.085503 |
| C | 4.802467 | -1.808261 | -1.256222 |
| C | 3.230007 | -3.782529 | -2.458814 |
| H | 1.595188 | -2.405126 | -2.243034 |
| C | 5.371313 | -3.024591 | -1.636470 |
| H | 5.405536 | -1.057055 | -0.757173 |
| C | 4.590693 | -4.011153 | -2.240637 |
| H | 2.611499 | -4.550839 | -2.914366 |
| H | 6.424848 | -3.204375 | -1.442504 |
| H | 5.036544 | -4.958889 | -2.529768 |
| C | 6.937484 | 0.097167 | 2.027319 |

| | | | |
|---|---|---|---|
| C | 7.568113 | -0.867653 | 2.812451 |
| C | 6.846286 | -1.947684 | 3.326140 |
| C | 5.480973 | -2.043809 | 3.048652 |
| C | 4.843058 | -1.075310 | 2.273072 |
| C | 5.561331 | 0.013356 | 1.742884 |
| H | 7.507040 | 0.931817 | 1.628067 |
| H | 8.631941 | -0.775609 | 3.020765 |
| H | 7.339423 | -2.700358 | 3.935789 |
| H | 4.903005 | -2.876481 | 3.444124 |
| H | 3.778013 | -1.155534 | 2.074996 |
| S | 4.773668 | 1.279661 | 0.767011 |
| H | 3.612039 | 0.364438 | -0.185871 |
| H | -3.021904 | -4.920907 | 2.478631 |
| H | -1.706301 | -4.565751 | 4.541724 |
| H | -1.425069 | -2.269507 | 5.464047 |
| C | -3.556644 | -0.714929 | -3.157611 |
| C | -1.305742 | 3.588098 | 0.394948 |
| C | -2.205673 | 4.329187 | 1.172895 |
| C | 0.016084 | 4.042195 | 0.226780 |
| C | -1.756788 | 5.512015 | 1.746598 |
| H | -3.225248 | 4.008572 | 1.321480 |
| C | 0.399399 | 5.229073 | 0.831853 |
| H | 0.737627 | 3.493887 | -0.370864 |
| C | -0.464545 | 5.999015 | 1.602528 |
| H | -0.146477 | 6.925539 | 2.064023 |
| F | -2.626692 | 6.222551 | 2.494479 |
| F | 1.662454 | 5.662496 | 0.657177 |
| C | -2.168479 | -1.215418 | -3.601624 |
| H | -1.868446 | -2.110697 | -3.051882 |
| H | -2.182772 | -1.445000 | -4.674124 |
| H | -1.406932 | -0.442918 | -3.439160 |
| C | -3.909991 | 0.562159 | -3.944420 |
| H | -3.207303 | 1.371794 | -3.722857 |
| H | -3.874218 | 0.365306 | -5.022866 |
| H | -4.915806 | 0.920269 | -3.698152 |
| C | -4.607435 | -1.801985 | -3.457891 |
| H | -5.612321 | -1.483586 | -3.153933 |
| H | -4.641616 | -2.007471 | -4.534054 |
| H | -4.375918 | -2.744949 | -2.949399 |

TS Conformation 95

B3LYP/6-31G(d) Energy = -3411.882364
M06-2X/def2tzvpp/IEFPCM(chloroform) Energy = -3411.886855
M06-2X/def2tzvpp/IEFPCM(chloroform)//B3LYP/6-31G(d) Quasiharmonic Free Energy = -3411.160997

Frequencies (Top 3 out of 291)

1. -853.0752 cm-1
2. 3.4613 cm-1
3. 9.9459 cm-1

B3LYP/6-31G(d) Molecular Geometry in Cartesian Coordinates

| | | | |
|---|---|---|---|
| C | 2.877888 | -1.120814 | -0.124607 |
| C | 2.749267 | -0.047379 | 0.974613 |
| H | 3.533139 | 0.701043 | 0.873026 |
| C | 1.349370 | 1.919451 | 0.259393 |
| N | 0.031871 | 2.242427 | 0.059331 |
| H | -0.620598 | 1.469810 | 0.178128 |
| N | 1.488019 | 0.648866 | 0.742473 |

| | | | |
|---|---|---|---|
| H | 0.648890 | 0.080850 | 0.824478 |
| C | 4.894644 | 0.033572 | -1.180453 |
| C | 3.949675 | -2.041875 | -2.106823 |
| C | 5.207768 | -0.077204 | -2.694232 |
| H | 4.396620 | 0.985389 | -0.977613 |
| C | 5.125236 | -1.583366 | -2.979285 |
| H | 4.050894 | -3.063766 | -1.728292 |
| H | 2.995937 | -1.991575 | -2.646443 |
| H | 6.177014 | 0.361962 | -2.943233 |
| H | 4.435404 | 0.460076 | -3.257619 |
| H | 6.051605 | -2.079899 | -2.671649 |
| H | 4.963691 | -1.810355 | -4.037239 |
| N | 3.924282 | -1.076145 | -0.988663 |
| O | 2.006954 | -2.000716 | -0.238111 |
| S | 2.669251 | 2.934305 | -0.048884 |
| C | 6.142515 | -0.060713 | -0.313273 |
| C | 6.730168 | -1.281432 | 0.044285 |
| C | 6.759695 | 1.127618 | 0.101591 |
| C | 7.912777 | -1.312957 | 0.785049 |
| H | 6.249443 | -2.214767 | -0.234527 |
| C | 7.941705 | 1.098374 | 0.842775 |
| C | 8.524537 | -0.123262 | 1.183989 |
| H | 8.352657 | -2.269286 | 1.055924 |
| O | -1.370485 | -0.353946 | 0.919097 |
| O | -1.846691 | 0.147044 | -1.896114 |
| C | -2.168741 | -1.288124 | 0.888413 |
| C | -2.697772 | -1.983213 | -0.315810 |
| O | -2.750030 | -1.792396 | 1.981781 |
| C | -2.509536 | -1.099459 | 3.222198 |
| H | -3.072284 | -1.658231 | 3.969469 |
| H | -1.444976 | -1.100521 | 3.464954 |
| H | -2.880463 | -0.074551 | 3.153252 |
| S | -1.973749 | -1.323053 | -1.825504 |
| C | -0.397761 | -2.109510 | -2.241342 |
| H | -0.541463 | -3.189062 | -2.307492 |
| H | -0.095031 | -1.687776 | -3.203702 |
| H | 0.334214 | -1.869604 | -1.462214 |
| C | -3.089189 | -1.909225 | -3.116898 |
| H | -2.681639 | -1.548855 | -4.063916 |
| H | -3.154890 | -2.997998 | -3.092501 |
| H | -4.059074 | -1.448011 | -2.897389 |
| C | -2.697940 | -3.497709 | -0.279050 |
| C | -3.855379 | -4.221618 | -0.603818 |
| C | -1.541215 | -4.201822 | 0.103202 |
| C | -3.849148 | -5.617234 | -0.562585 |
| H | -4.769228 | -3.688563 | -0.846777 |
| C | -1.539855 | -5.595017 | 0.145214 |
| H | -0.639855 | -3.656195 | 0.374459 |
| C | -2.693607 | -6.306546 | -0.193516 |
| H | -4.755297 | -6.163787 | -0.808838 |
| H | -0.639386 | -6.123380 | 0.445993 |
| H | -2.693047 | -7.392659 | -0.159812 |
| C | -5.873476 | -0.021124 | 1.814839 |
| C | -5.885012 | 1.018160 | 2.746631 |
| C | -5.647402 | 2.334244 | 2.341244 |
| C | -5.398885 | 2.598993 | 0.993354 |
| C | -5.385555 | 1.561173 | 0.059220 |
| C | -5.621470 | 0.233537 | 0.454619 |
| H | -6.069431 | -1.041819 | 2.130407 |
| H | -6.091473 | 0.799117 | 3.792303 |
| H | -5.660631 | 3.143545 | 3.066759 |
| H | -5.211613 | 3.614147 | 0.656214 |

| | | | |
|---|---|---|---|
| H | -5.194710 | 1.781433 | -0.986575 |
| S | -5.627914 | -1.102091 | -0.733889 |
| H | -3.993227 | -1.577102 | -0.466044 |
| H | 6.303714 | 2.081990 | -0.151456 |
| H | 8.401533 | 2.030506 | 1.160053 |
| H | 9.443050 | -0.148495 | 1.764012 |
| C | 2.844265 | -0.618066 | 2.435528 |
| C | -0.608503 | 3.431670 | -0.342158 |
| C | -1.955708 | 3.289994 | -0.725869 |
| C | -0.019383 | 4.704033 | -0.329347 |
| C | -2.669451 | 4.417847 | -1.092456 |
| H | -2.437642 | 2.319568 | -0.749091 |
| C | -0.792942 | 5.791024 | -0.716169 |
| H | 1.008670 | 4.851437 | -0.036784 |
| C | -2.123133 | 5.695597 | -1.103944 |
| H | -2.699601 | 6.563936 | -1.397517 |
| F | -3.965979 | 4.271339 | -1.453896 |
| F | -0.219343 | 7.012545 | -0.703145 |
| C | 2.883165 | 0.582567 | 3.402314 |
| H | 1.982693 | 1.198455 | 3.311954 |
| H | 2.949493 | 0.229318 | 4.438258 |
| H | 3.749390 | 1.224901 | 3.207820 |
| C | 4.148482 | -1.423456 | 2.582361 |
| H | 4.256711 | -1.768026 | 3.617474 |
| H | 4.153522 | -2.310642 | 1.938570 |
| H | 5.028366 | -0.820488 | 2.337107 |
| C | 1.652861 | -1.523509 | 2.801769 |
| H | 1.591194 | -2.393681 | 2.143765 |
| H | 1.765622 | -1.874854 | 3.834563 |
| H | 0.703686 | -0.980310 | 2.742543 |

TS Conformation 96

B3LYP/6-31G(d) Energy = -3411.883428
M06-2X/def2tzvpp/IEFPCM(chloroform) Energy = -3411.887575
M06-2X/def2tzvpp/IEFPCM(chloroform)//B3LYP/6-31G(d) Quasiharmonic Free Energy = -3411.160906

Frequencies (Top 3 out of 291)

1. -853.5744 cm-1
2. 7.9223 cm-1
3. 10.5669 cm-1

B3LYP/6-31G(d) Molecular Geometry in Cartesian Coordinates

| | | | |
|---|---|---|---|
| C | -2.772674 | -1.608847 | -0.471939 |
| C | -3.401196 | -0.357545 | -1.119545 |
| H | -4.223510 | 0.022314 | -0.514579 |
| C | -2.390118 | 1.855919 | -0.418827 |
| N | -1.145395 | 2.431840 | -0.370930 |
| H | -0.392695 | 1.833317 | -0.698982 |
| N | -2.346508 | 0.656145 | -1.083146 |
| H | -1.421485 | 0.283657 | -1.276944 |
| C | -4.111630 | -1.249624 | 1.661318 |
| C | -2.492770 | -3.093324 | 1.444940 |
| C | -3.639446 | -1.730861 | 3.057599 |
| H | -3.931637 | -0.175847 | 1.562733 |
| C | -3.175847 | -3.173865 | 2.815103 |
| H | -2.562841 | -4.014910 | 0.859285 |
| H | -1.429613 | -2.839666 | 1.537260 |
| H | -4.431949 | -1.645804 | 3.805111 |

| | | | |
|---|---|---|---|
| H | -2.794903 | -1.112385 | 3.383323 |
| H | -4.036755 | -3.850155 | 2.777679 |
| H | -2.496886 | -3.539550 | 3.590494 |
| N | -3.196252 | -1.992093 | 0.755613 |
| O | -1.848558 | -2.212421 | -1.047056 |
| S | -3.811816 | 2.504306 | 0.215408 |
| C | -5.589800 | -1.517112 | 1.409386 |
| C | -6.066377 | -2.747045 | 0.937827 |
| C | -6.515120 | -0.507379 | 1.708697 |
| C | -7.436521 | -2.967822 | 0.784370 |
| H | -5.364002 | -3.530679 | 0.668509 |
| C | -7.884369 | -0.726397 | 1.555765 |
| C | -8.350036 | -1.959672 | 1.096092 |
| H | -7.788750 | -3.927716 | 0.415655 |
| O | 2.505556 | 1.527201 | -2.094033 |
| O | 0.524873 | -0.041485 | -0.620562 |
| C | 3.364381 | 0.887195 | -1.496576 |
| C | 3.202432 | -0.507018 | -0.989422 |
| O | 4.565889 | 1.376053 | -1.169795 |
| C | 4.809488 | 2.752737 | -1.533348 |
| H | 4.127071 | 3.415692 | -0.996808 |
| H | 5.840890 | 2.943935 | -1.239185 |
| H | 4.681083 | 2.890067 | -2.609714 |
| S | 1.486652 | -0.977235 | -1.254878 |
| C | 1.069225 | -1.208857 | -3.002068 |
| H | 0.047920 | -1.597265 | -3.027698 |
| H | 1.782276 | -1.904899 | -3.448654 |
| H | 1.139428 | -0.220416 | -3.458451 |
| C | 1.307698 | -2.612346 | -0.532168 |
| H | 0.236001 | -2.829689 | -0.594496 |
| H | 1.919127 | -3.331589 | -1.078697 |
| H | 1.647344 | -2.510267 | 0.505605 |
| C | 4.190333 | -1.540536 | -1.475641 |
| C | 4.655076 | -1.517296 | -2.803356 |
| C | 4.669021 | -2.536098 | -0.609472 |
| C | 5.570373 | -2.466471 | -3.254955 |
| H | 4.310274 | -0.739799 | -3.481690 |
| C | 5.581367 | -3.488344 | -1.066094 |
| H | 4.345505 | -2.543990 | 0.426029 |
| C | 6.031414 | -3.458982 | -2.386787 |
| H | 5.924741 | -2.429342 | -4.281432 |
| H | 5.951519 | -4.244780 | -0.379998 |
| H | 6.745833 | -4.199106 | -2.736655 |
| C | 4.917030 | -1.836358 | 3.702727 |
| C | 6.210621 | -2.094113 | 4.155833 |
| C | 7.310403 | -1.485003 | 3.547061 |
| C | 7.095832 | -0.607221 | 2.482278 |
| C | 5.803079 | -0.337058 | 2.032608 |
| C | 4.687559 | -0.951344 | 2.632647 |
| H | 4.067393 | -2.318357 | 4.178442 |
| H | 6.359235 | -2.778880 | 4.988060 |
| H | 8.318282 | -1.688598 | 3.899277 |
| H | 7.941123 | -0.121143 | 1.999647 |
| H | 5.649217 | 0.357640 | 1.211872 |
| S | 3.021833 | -0.612560 | 2.096149 |
| H | 3.205605 | -0.479093 | 0.373033 |
| H | -6.157127 | 0.460345 | 2.051778 |
| H | -8.585933 | 0.070672 | 1.786743 |
| H | -9.416016 | -2.130626 | 0.972777 |
| C | -3.975182 | -0.597935 | -2.560719 |
| C | -0.661224 | 3.682327 | 0.055246 |
| C | -1.410575 | 4.668671 | 0.709973 |

| | | | |
|---|---|---|---|
| C | 0.702514 | 3.905722 | -0.219560 |
| C | -0.766292 | 5.846511 | 1.071045 |
| H | -2.456474 | 4.528247 | 0.937214 |
| C | 1.280005 | 5.098369 | 0.174608 |
| H | 1.300921 | 3.168520 | -0.745001 |
| C | 0.576969 | 6.104352 | 0.826848 |
| H | 1.046485 | 7.032295 | 1.128229 |
| F | -1.489765 | 6.793569 | 1.702303 |
| F | 2.593348 | 5.295936 | -0.094394 |
| C | -4.951102 | -1.789453 | -2.516677 |
| H | -5.746647 | -1.633896 | -1.779970 |
| H | -5.425119 | -1.920732 | -3.496498 |
| H | -4.435742 | -2.725276 | -2.271133 |
| C | -2.878730 | -0.877861 | -3.605808 |
| H | -2.346143 | -1.806369 | -3.386096 |
| H | -3.333033 | -0.967616 | -4.599834 |
| H | -2.156562 | -0.053940 | -3.651705 |
| C | -4.744745 | 0.671121 | -2.978136 |
| H | -5.572373 | 0.882725 | -2.293069 |
| H | -4.092303 | 1.549804 | -2.988503 |
| H | -5.159143 | 0.542566 | -3.985447 |

TS Conformation 97

B3LYP/6-31G(d) Energy = -3411.876478
M06-2X/def2tzvpp/IEFPCM(chloroform) Energy = -3411.88693
M06-2X/def2tzvpp/IEFPCM(chloroform)//B3LYP/6-31G(d) Quasiharmonic Free Energy = -3411.160873

Frequencies (Top 3 out of 291)

1. -771.4787 cm-1
2. 7.5911 cm-1
3. 13.5658 cm-1

B3LYP/6-31G(d) Molecular Geometry in Cartesian Coordinates

| | | | |
|---|---|---|---|
| C | 3.030930 | -0.349788 | 1.000264 |
| C | 3.405768 | 0.931813 | 0.214208 |
| H | 4.118707 | 0.693528 | -0.576734 |
| C | 1.525689 | 0.916592 | -1.442794 |
| N | 0.374926 | 1.549572 | -1.835818 |
| H | -0.276642 | 0.944246 | -2.327256 |
| N | 2.210798 | 1.484832 | -0.422761 |
| H | 1.687977 | 2.145015 | 0.140160 |
| C | 4.712670 | -1.814081 | -0.240398 |
| C | 3.348918 | -2.676740 | 1.610628 |
| C | 4.434661 | -3.326757 | -0.430058 |
| H | 4.383714 | -1.278205 | -1.133053 |
| C | 4.153364 | -3.827044 | 0.992768 |
| H | 3.505347 | -2.555600 | 2.687395 |
| H | 2.271831 | -2.805127 | 1.454490 |
| H | 5.266787 | -3.839871 | -0.919126 |
| H | 3.541779 | -3.440108 | -1.056212 |
| H | 5.091550 | -3.984649 | 1.535474 |
| H | 3.598516 | -4.769909 | 1.012528 |
| N | 3.801089 | -1.471582 | 0.881023 |
| O | 2.061427 | -0.328218 | 1.767870 |
| S | 2.001815 | -0.506980 | -2.230668 |
| C | 6.186427 | -1.525346 | 0.009669 |
| C | 6.993379 | -1.168896 | -1.079739 |
| C | 6.787619 | -1.662095 | 1.268231 |

| | | | |
|---|---|---|---|
| C | 8.364731 | -0.963796 | -0.921200 |
| H | 6.541436 | -1.051380 | -2.062572 |
| C | 8.158682 | -1.457422 | 1.430350 |
| C | 8.952972 | -1.110222 | 0.335986 |
| H | 8.970942 | -0.683913 | -1.778721 |
| O | -1.927740 | -0.307866 | -2.281056 |
| O | -1.980247 | 0.591964 | 0.570733 |
| C | -2.863438 | -1.064841 | -2.047167 |
| C | -3.336029 | -1.477392 | -0.696751 |
| O | -3.658159 | -1.570332 | -2.998998 |
| C | -3.383121 | -1.134226 | -4.343753 |
| H | -4.105311 | -1.660348 | -4.967316 |
| H | -3.520987 | -0.053422 | -4.425163 |
| H | -2.360607 | -1.393065 | -4.629574 |
| S | -2.162380 | -0.871250 | 0.533190 |
| C | -2.791163 | -1.458497 | 2.119121 |
| H | -2.871212 | -2.545563 | 2.122905 |
| H | -2.075641 | -1.103009 | 2.864181 |
| H | -3.765568 | -0.985002 | 2.271760 |
| C | -0.567538 | -1.705974 | 0.364209 |
| H | -0.715489 | -2.780242 | 0.493165 |
| H | -0.189283 | -1.473280 | -0.634745 |
| H | 0.109138 | -1.285711 | 1.116382 |
| C | -3.712837 | -2.928740 | -0.502636 |
| C | -2.901406 | -3.962295 | -1.004157 |
| C | -4.892207 | -3.266580 | 0.178683 |
| C | -3.250800 | -5.298327 | -0.815335 |
| H | -2.000651 | -3.718887 | -1.563015 |
| C | -5.237787 | -4.605554 | 0.369822 |
| H | -5.549990 | -2.471345 | 0.518281 |
| C | -4.418362 | -5.623200 | -0.120184 |
| H | -2.615226 | -6.084122 | -1.214593 |
| H | -6.157621 | -4.851277 | 0.893482 |
| H | -4.692140 | -6.664426 | 0.027508 |
| C | -4.902476 | 1.971755 | 1.521957 |
| C | -4.684770 | 2.464815 | 2.808893 |
| C | -5.213032 | 1.808738 | 3.923804 |
| C | -5.972748 | 0.652249 | 3.734461 |
| C | -6.193405 | 0.155877 | 2.447155 |
| C | -5.656731 | 0.799883 | 1.312755 |
| H | -4.493530 | 2.497400 | 0.664970 |
| H | -4.097928 | 3.371205 | 2.940156 |
| H | -5.043202 | 2.198406 | 4.924306 |
| H | -6.405067 | 0.137401 | 4.590130 |
| H | -6.805779 | -0.730746 | 2.304096 |
| S | -5.936947 | 0.153926 | -0.326259 |
| H | -4.432857 | -0.724566 | -0.490002 |
| H | 6.176943 | -1.902823 | 2.133442 |
| H | 8.605493 | -1.564025 | 2.415431 |
| H | 10.019745 | -0.947963 | 0.463691 |
| C | 4.058839 | 2.038012 | 1.128129 |
| C | -0.116631 | 2.825333 | -1.478734 |
| C | -1.501872 | 2.981283 | -1.327655 |
| C | 0.735222 | 3.935498 | -1.351411 |
| C | -1.998023 | 4.241131 | -1.027905 |
| H | -2.171514 | 2.134783 | -1.402741 |
| C | 0.174728 | 5.169329 | -1.043916 |
| H | 1.800168 | 3.863190 | -1.530649 |
| C | -1.189524 | 5.362469 | -0.870266 |
| H | -1.602596 | 6.333782 | -0.628302 |
| F | -3.326405 | 4.383512 | -0.857740 |
| F | 0.996916 | 6.231213 | -0.923862 |

| | | | |
|---|---|---|---|
| C | 5.224578 | 1.430248 | 1.928885 |
| H | 5.969859 | 0.968155 | 1.275986 |
| H | 5.724403 | 2.220443 | 2.500584 |
| H | 4.880081 | 0.674022 | 2.642049 |
| C | 3.049635 | 2.647899 | 2.126794 |
| H | 2.533038 | 1.872733 | 2.698229 |
| H | 3.584010 | 3.300687 | 2.826280 |
| H | 2.295203 | 3.278070 | 1.638539 |
| C | 4.621287 | 3.148501 | 0.217264 |
| H | 3.840594 | 3.611792 | -0.394292 |
| H | 5.078613 | 3.937822 | 0.824956 |
| H | 5.391421 | 2.756441 | -0.457634 |

TS Conformation 98

B3LYP/6-31G(d) Energy = -3411.877894
M06-2X/def2tzvpp/IEFPCM(chloroform) Energy = -3411.885631
M06-2X/def2tzvpp/IEFPCM(chloroform)//B3LYP/6-31G(d) Quasiharmonic Free Energy = -3411.160813

Frequencies (Top 3 out of 291)

1. -641.0795 cm-1
2. 5.3030 cm-1
3. 7.8325 cm-1

B3LYP/6-31G(d) Molecular Geometry in Cartesian Coordinates

| | | | |
|---|---|---|---|
| C | -2.803272 | -2.171354 | -0.186328 |
| C | -2.350763 | -1.729225 | -1.594032 |
| H | -3.210253 | -1.584564 | -2.246872 |
| C | -2.362091 | 0.776413 | -1.651243 |
| N | -1.618110 | 1.822269 | -1.150888 |
| H | -0.826363 | 1.535747 | -0.580825 |
| N | -1.734043 | -0.414921 | -1.441397 |
| H | -0.908939 | -0.408545 | -0.847634 |
| C | -4.557959 | -2.916384 | 1.349197 |
| C | -5.179275 | -2.582513 | -1.008309 |
| C | -5.906823 | -3.611776 | 1.045013 |
| H | -3.830459 | -3.620695 | 1.763627 |
| C | -6.440250 | -2.839815 | -0.170329 |
| H | -5.236828 | -1.654370 | -1.583983 |
| H | -4.994819 | -3.413279 | -1.701400 |
| H | -6.582922 | -3.591593 | 1.903555 |
| H | -5.724818 | -4.660210 | 0.778198 |
| H | -6.879348 | -1.888684 | 0.149658 |
| H | -7.201302 | -3.389376 | -0.731866 |
| N | -4.104270 | -2.511448 | 0.004080 |
| O | -1.996506 | -2.173565 | 0.749999 |
| S | -3.859934 | 0.920802 | -2.417458 |
| C | -4.696019 | -1.751292 | 2.325539 |
| C | -4.872784 | -0.428540 | 1.904521 |
| C | -4.692498 | -2.023261 | 3.700424 |
| C | -5.049966 | 0.596325 | 2.837343 |
| H | -4.854760 | -0.189409 | 0.844855 |
| C | -4.871235 | -1.002649 | 4.633419 |
| C | -5.052995 | 0.313952 | 4.203539 |
| H | -5.183995 | 1.617659 | 2.490295 |
| O | 0.726189 | 0.757535 | 0.282259 |
| O | 2.694824 | 0.050484 | -1.875326 |
| C | 1.547968 | 0.358415 | 1.111889 |
| C | 3.020898 | 0.296418 | 0.879867 |

| | | | |
|---|---|---|---|
| O | 1.238422 | -0.113211 | 2.308073 |
| C | -0.170576 | -0.196240 | 2.647955 |
| H | -0.184803 | -0.567889 | 3.671395 |
| H | -0.625588 | 0.796215 | 2.595209 |
| H | -0.684658 | -0.886272 | 1.976350 |
| S | 3.344440 | 0.842685 | -0.814682 |
| C | 2.956968 | 2.605253 | -1.031167 |
| H | 3.459037 | 3.186674 | -0.255684 |
| H | 3.301423 | 2.888825 | -2.028668 |
| H | 1.872959 | 2.694966 | -0.958754 |
| C | 5.141588 | 0.806375 | -0.982228 |
| H | 5.344504 | 1.074705 | -2.021408 |
| H | 5.599374 | 1.509442 | -0.285736 |
| H | 5.467758 | -0.218758 | -0.787241 |
| C | 3.895097 | 0.984734 | 1.905669 |
| C | 5.069262 | 0.368100 | 2.363741 |
| C | 3.541101 | 2.242485 | 2.426499 |
| C | 5.878300 | 1.007884 | 3.304268 |
| H | 5.314965 | -0.628486 | 2.008463 |
| C | 4.348684 | 2.876624 | 3.368971 |
| H | 2.616969 | 2.720600 | 2.108340 |
| C | 5.525149 | 2.261860 | 3.804622 |
| H | 6.781151 | 0.516054 | 3.655471 |
| H | 4.057053 | 3.845023 | 3.765849 |
| H | 6.155896 | 2.754160 | 4.539744 |
| C | 6.341208 | -2.707329 | -0.116347 |
| C | 7.249750 | -2.712724 | -1.177349 |
| C | 6.795248 | -2.744214 | -2.498085 |
| C | 5.419552 | -2.779895 | -2.741995 |
| C | 4.509693 | -2.777245 | -1.683761 |
| C | 4.948563 | -2.731510 | -0.343900 |
| H | 6.702561 | -2.700207 | 0.908609 |
| H | 8.317601 | -2.698798 | -0.969187 |
| H | 7.502089 | -2.751453 | -3.323794 |
| H | 5.049510 | -2.814681 | -3.764369 |
| H | 3.443354 | -2.807829 | -1.883248 |
| S | 3.794261 | -2.700402 | 1.012713 |
| H | 3.335355 | -0.989831 | 0.861551 |
| H | -4.542137 | -3.045298 | 4.043161 |
| H | -4.862467 | -1.234379 | 5.695483 |
| H | -5.190659 | 1.112215 | 4.928167 |
| C | -1.382269 | -2.745332 | -2.292536 |
| C | -1.753765 | 3.214573 | -1.231198 |
| C | -2.555694 | 3.895108 | -2.158664 |
| C | -0.963222 | 3.952406 | -0.325319 |
| C | -2.546253 | 5.284187 | -2.142340 |
| H | -3.176199 | 3.364242 | -2.864951 |
| C | -0.994794 | 5.336793 | -0.368661 |
| H | -0.353805 | 3.453141 | 0.420776 |
| C | -1.779843 | 6.046897 | -1.268056 |
| H | -1.798306 | 7.129338 | -1.285005 |
| F | -3.317698 | 5.932193 | -3.036932 |
| F | -0.228692 | 6.023001 | 0.507761 |
| C | -0.017909 | -2.850789 | -1.581958 |
| H | -0.123113 | -3.132758 | -0.531783 |
| H | 0.595209 | -3.611598 | -2.079273 |
| H | 0.543595 | -1.910795 | -1.631678 |
| C | -1.165865 | -2.271747 | -3.743438 |
| H | -0.725682 | -1.269901 | -3.771900 |
| H | -0.484863 | -2.954426 | -4.265164 |
| H | -2.109132 | -2.242084 | -4.302169 |
| C | -2.051736 | -4.133795 | -2.309997 |

| | | | |
|---|---|---|---|
| H | -2.196175 | -4.530353 | -1.298605 |
| H | -3.025825 | -4.107418 | -2.814448 |
| H | -1.420399 | -4.845450 | -2.853751 |

TS Conformation 99

B3LYP/6-31G(d) Energy = -3411.882005
M06-2X/def2tzvpp/IEFPCM(chloroform) Energy = -3411.886268
M06-2X/def2tzvpp/IEFPCM(chloroform)//B3LYP/6-31G(d) Quasiharmonic Free Energy = -3411.160793

Frequencies (Top 3 out of 291)

1. -919.6519 cm-1
2. 6.7732 cm-1
3. 16.2054 cm-1

B3LYP/6-31G(d) Molecular Geometry in Cartesian Coordinates

| | | | |
|---|---|---|---|
| C | -3.031769 | -1.256476 | 0.715510 |
| C | -3.199763 | -0.501341 | -0.621842 |
| H | -4.004302 | 0.228750 | -0.564908 |
| C | -1.827133 | 1.613700 | -0.704479 |
| N | -0.506090 | 1.995316 | -0.680793 |
| H | 0.169239 | 1.240954 | -0.595169 |
| N | -1.962386 | 0.261521 | -0.796280 |
| H | -1.120580 | -0.305657 | -0.759282 |
| C | -4.836292 | 0.165590 | 1.838353 |
| C | -3.483568 | -1.440622 | 3.109224 |
| C | -5.041402 | 0.356039 | 3.367503 |
| H | -4.382978 | 1.068968 | 1.417905 |
| C | -3.786620 | -0.244243 | 4.014051 |
| H | -4.101134 | -2.310602 | 3.373021 |
| H | -2.440059 | -1.756897 | 3.106564 |
| H | -5.931310 | -0.196070 | 3.690006 |
| H | -5.202121 | 1.409877 | 3.612165 |
| H | -3.941544 | -0.532759 | 5.058380 |
| H | -2.953512 | 0.467313 | 3.978514 |
| N | -3.841048 | -0.944973 | 1.766819 |
| O | -2.120343 | -2.085314 | 0.825781 |
| S | -3.129170 | 2.686834 | -0.626958 |
| C | -6.142178 | -0.108447 | 1.109705 |
| C | -6.842204 | -1.309786 | 1.293602 |
| C | -6.693392 | 0.871292 | 0.275793 |
| C | -8.064523 | -1.524983 | 0.659503 |
| H | -6.420452 | -2.086296 | 1.927189 |
| C | -7.920177 | 0.658303 | -0.359152 |
| C | -8.609152 | -0.539135 | -0.168822 |
| H | -8.592587 | -2.463218 | 0.809254 |
| O | 5.363577 | -1.428229 | -1.714553 |
| O | 1.143891 | -0.458657 | -1.570252 |
| C | 4.727161 | -2.017267 | -0.842843 |
| C | 3.283316 | -1.818100 | -0.563347 |
| O | 5.281149 | -2.888296 | 0.010705 |
| C | 6.710856 | -3.038685 | -0.089482 |
| H | 7.000974 | -3.297131 | -1.110385 |
| H | 7.201164 | -2.108977 | 0.209170 |
| H | 6.961014 | -3.844233 | 0.600655 |
| S | 2.588969 | -0.710798 | -1.809759 |
| C | 2.803484 | -1.368142 | -3.482618 |
| H | 2.253943 | -2.311764 | -3.509888 |
| H | 2.368581 | -0.649358 | -4.181155 |

| | | | |
|---|---|---|---|
| H | 3.870637 | -1.518460 | -3.650740 |
| C | 3.497757 | 0.848329 | -1.786001 |
| H | 3.479018 | 1.178378 | -0.742467 |
| H | 4.515021 | 0.670460 | -2.129574 |
| H | 2.937696 | 1.535244 | -2.424930 |
| C | 2.399343 | -3.018349 | -0.361609 |
| C | 2.709940 | -4.255765 | -0.956527 |
| C | 1.228886 | -2.913061 | 0.404873 |
| C | 1.883803 | -5.360484 | -0.768274 |
| H | 3.615344 | -4.360657 | -1.548162 |
| C | 0.390747 | -4.016001 | 0.575733 |
| H | 0.966108 | -1.969875 | 0.869788 |
| C | 0.719381 | -5.240875 | -0.004738 |
| H | 2.145228 | -6.312108 | -1.223437 |
| H | -0.523150 | -3.895319 | 1.146324 |
| H | 0.069648 | -6.100991 | 0.133394 |
| C | 5.611699 | -0.382885 | 2.344689 |
| C | 6.955729 | -0.042448 | 2.506061 |
| C | 7.381381 | 1.274830 | 2.317864 |
| C | 6.443859 | 2.248629 | 1.969827 |
| C | 5.099208 | 1.913110 | 1.800864 |
| C | 4.660556 | 0.588633 | 1.981091 |
| H | 5.283999 | -1.404569 | 2.512237 |
| H | 7.671095 | -0.808492 | 2.798477 |
| H | 8.427090 | 1.539498 | 2.450356 |
| H | 6.757515 | 3.280428 | 1.828900 |
| H | 4.382053 | 2.685219 | 1.538024 |
| S | 2.937169 | 0.155568 | 1.785425 |
| H | 3.161595 | -0.985513 | 0.537267 |
| H | -6.150998 | 1.799239 | 0.112965 |
| H | -8.331819 | 1.428779 | -1.005730 |
| H | -9.561985 | -0.707344 | -0.663644 |
| C | -3.487482 | -1.429885 | -1.854760 |
| C | 0.077042 | 3.279424 | -0.633569 |
| C | -0.437237 | 4.382110 | -1.328873 |
| C | 1.275488 | 3.395664 | 0.091001 |
| C | 0.258172 | 5.581965 | -1.262095 |
| H | -1.355374 | 4.318523 | -1.894234 |
| C | 1.928041 | 4.619697 | 0.101442 |
| H | 1.673868 | 2.562366 | 0.665193 |
| C | 1.449422 | 5.741986 | -0.562628 |
| H | 1.970643 | 6.690774 | -0.531981 |
| F | -0.234414 | 6.643917 | -1.931565 |
| F | 3.082978 | 4.730394 | 0.796903 |
| C | -4.661808 | -2.369607 | -1.522214 |
| H | -5.558284 | -1.813391 | -1.230951 |
| H | -4.914053 | -2.972719 | -2.402588 |
| H | -4.407160 | -3.060308 | -0.710569 |
| C | -2.264267 | -2.276823 | -2.259682 |
| H | -1.421252 | -1.647894 | -2.568879 |
| H | -1.928965 | -2.918460 | -1.442070 |
| H | -2.529205 | -2.909601 | -3.115744 |
| C | -3.885527 | -0.522750 | -3.036284 |
| H | -4.799638 | 0.040653 | -2.817730 |
| H | -4.066485 | -1.128094 | -3.932859 |
| H | -3.095277 | 0.198956 | -3.268817 |

TS Conformation 100

B3LYP/6-31G(d) Energy = -3411.882119
M06-2X/def2tzvpp/IEFPCM(chloroform) Energy = -3411.887871

M06-2X/def2tzvpp/IEFPCM(chloroform)//B3LYP/6-31G(d) Quasiharmonic Free Energy = -3411.160672

Frequencies (Top 3 out of 291)

1. -868.9333 cm-1
2. 8.4072 cm-1
3. 15.7723 cm-1

B3LYP/6-31G(d) Molecular Geometry in Cartesian Coordinates

| | | | |
|---|---|---|---|
| C | -2.410099 | -1.677252 | -0.312349 |
| C | -2.796569 | -0.560605 | -1.306188 |
| H | -3.653278 | 0.006923 | -0.948328 |
| C | -1.665285 | 1.678118 | -0.954454 |
| N | -0.386016 | 2.170567 | -0.869387 |
| H | 0.341470 | 1.468059 | -0.966872 |
| N | -1.653286 | 0.352487 | -1.299438 |
| H | -0.756059 | -0.121341 | -1.244472 |
| C | -4.153254 | -0.859414 | 1.360551 |
| C | -2.571581 | -2.648404 | 1.931230 |
| C | -4.202571 | -1.178325 | 2.880800 |
| H | -3.806908 | 0.168755 | 1.215819 |
| C | -2.853699 | -1.835720 | 3.196426 |
| H | -3.099047 | -3.612259 | 1.939769 |
| H | -1.514284 | -2.844006 | 1.754885 |
| H | -5.021309 | -1.878229 | 3.080761 |
| H | -4.396028 | -0.272672 | 3.462445 |
| H | -2.885660 | -2.458856 | 4.095657 |
| H | -2.071160 | -1.081339 | 3.332964 |
| N | -3.096721 | -1.789896 | 0.852664 |
| O | -1.433187 | -2.405116 | -0.567112 |
| S | -3.082842 | 2.558426 | -0.707500 |
| C | -5.510026 | -1.018766 | 0.693757 |
| C | -6.108683 | -2.279318 | 0.553659 |
| C | -6.209662 | 0.112381 | 0.258110 |
| C | -7.377062 | -2.404962 | -0.009909 |
| H | -5.574061 | -3.169244 | 0.877197 |
| C | -7.482964 | -0.011591 | -0.304674 |
| C | -8.069900 | -1.269334 | -0.439905 |
| H | -7.825718 | -3.389289 | -0.115665 |
| O | 3.331112 | 0.711035 | -1.879962 |
| O | 1.105501 | -0.350637 | -0.345687 |
| C | 4.072261 | 0.168778 | -1.066686 |
| C | 3.756370 | -1.080639 | -0.307823 |
| O | 5.268999 | 0.643196 | -0.713357 |
| C | 5.669913 | 1.883254 | -1.333844 |
| H | 6.672494 | 2.077129 | -0.954180 |
| H | 5.680343 | 1.779206 | -2.421815 |
| H | 4.989570 | 2.690097 | -1.052373 |
| S | 2.033395 | -1.474110 | -0.623398 |
| C | 1.751133 | -2.105646 | -2.297433 |
| H | 2.437277 | -2.933125 | -2.488480 |
| H | 1.947110 | -1.266214 | -2.966450 |
| H | 0.705551 | -2.420501 | -2.338638 |
| C | 1.637942 | -2.863443 | 0.445343 |
| H | 0.563674 | -3.023747 | 0.307341 |
| H | 1.879772 | -2.538370 | 1.462608 |
| H | 2.232824 | -3.732880 | 0.162313 |
| C | 4.685163 | -2.253665 | -0.531800 |
| C | 4.983630 | -3.147463 | 0.509629 |
| C | 5.277948 | -2.462529 | -1.790972 |
| C | 5.844952 | -4.223998 | 0.290406 |

| | | | |
|---|---|---|---|
| H | 4.559242 | -2.979710 | 1.495498 |
| C | 6.142239 | -3.534568 | -2.004252 |
| H | 5.070638 | -1.772184 | -2.605515 |
| C | 6.424035 | -4.422626 | -0.963325 |
| H | 6.071864 | -4.901261 | 1.109101 |
| H | 6.595718 | -3.675482 | -2.981547 |
| H | 7.097506 | -5.259167 | -1.128203 |
| C | 1.874244 | 1.196165 | 2.575839 |
| C | 0.673246 | 1.792569 | 2.959555 |
| C | -0.094957 | 1.241574 | 3.988303 |
| C | 0.352489 | 0.080055 | 4.624034 |
| C | 1.543880 | -0.529313 | 4.228555 |
| C | 2.331068 | 0.019244 | 3.198284 |
| H | 2.475224 | 1.652700 | 1.794870 |
| H | 0.343336 | 2.700379 | 2.460446 |
| H | -1.020516 | 1.719398 | 4.298971 |
| H | -0.226014 | -0.353393 | 5.437419 |
| H | 1.886185 | -1.431950 | 4.727403 |
| S | 3.880107 | -0.746666 | 2.758270 |
| H | 3.756095 | -0.814685 | 1.035857 |
| H | -5.747903 | 1.092652 | 0.345039 |
| H | -8.010352 | 0.876937 | -0.641099 |
| H | -9.058739 | -1.367537 | -0.879668 |
| C | -3.157671 | -1.074915 | -2.746822 |
| C | 0.156977 | 3.448851 | -0.650827 |
| C | -0.562234 | 4.586349 | -0.258800 |
| C | 1.551810 | 3.533717 | -0.839064 |
| C | 0.140196 | 5.771098 | -0.067344 |
| H | -1.630915 | 4.556618 | -0.109102 |
| C | 2.186134 | 4.743462 | -0.622305 |
| H | 2.130424 | 2.676706 | -1.169179 |
| C | 1.513961 | 5.896184 | -0.233633 |
| H | 2.028221 | 6.834749 | -0.069479 |
| F | -0.555315 | 6.862512 | 0.310586 |
| F | 3.527326 | 4.806527 | -0.803268 |
| C | -1.946376 | -1.663884 | -3.493881 |
| H | -1.568246 | -2.560229 | -2.995941 |
| H | -2.242249 | -1.935971 | -4.514142 |
| H | -1.134890 | -0.931046 | -3.574983 |
| C | -3.702050 | 0.122091 | -3.551715 |
| H | -4.598362 | 0.543768 | -3.085180 |
| H | -2.961224 | 0.923949 | -3.630071 |
| H | -3.964407 | -0.198836 | -4.567190 |
| C | -4.254751 | -2.151284 | -2.641485 |
| H | -3.906217 | -3.033684 | -2.092170 |
| H | -5.150615 | -1.769687 | -2.142229 |
| H | -4.547438 | -2.482178 | -3.645057 |

TS Conformation 101

B3LYP/6-31G(d) Energy = -3411.881449
M06-2X/def2tzvpp/IEFPCM(chloroform) Energy = -3411.887981
M06-2X/def2tzvpp/IEFPCM(chloroform)//B3LYP/6-31G(d) Quasiharmonic Free Energy = -3411.160624

Frequencies (Top 3 out of 291)

1. -902.8766 cm-1
2. 7.8861 cm-1
3. 14.9485 cm-1

B3LYP/6-31G(d) Molecular Geometry in Cartesian Coordinates

| | | | |
|---|---|---|---|
| C | 2.604198 | -1.113227 | 0.276836 |
| C | 2.775490 | 0.171629 | 1.108122 |
| H | 3.562541 | 0.796921 | 0.690286 |
| C | 1.416815 | 2.170726 | 0.401797 |
| N | 0.100546 | 2.537065 | 0.255535 |
| H | -0.568684 | 1.797513 | 0.466725 |
| N | 1.529280 | 0.916639 | 0.935988 |
| H | 0.677076 | 0.373346 | 1.053058 |
| C | 4.246640 | -0.281461 | -1.485777 |
| C | 3.086911 | -2.442474 | -1.710534 |
| C | 4.181204 | -0.713070 | -2.973369 |
| H | 3.816586 | 0.717329 | -1.372882 |
| C | 4.020708 | -2.238358 | -2.910057 |
| H | 3.263836 | -3.375544 | -1.166631 |
| H | 2.030905 | -2.433052 | -2.007674 |
| H | 5.064074 | -0.388829 | -3.529682 |
| H | 3.298952 | -0.258643 | -3.440115 |
| H | 4.990068 | -2.715400 | -2.730432 |
| H | 3.606595 | -2.662603 | -3.829258 |
| N | 3.357136 | -1.280763 | -0.838737 |
| O | 1.744775 | -1.954069 | 0.591222 |
| S | 2.756265 | 3.114414 | -0.012326 |
| C | 5.667707 | -0.259893 | -0.938180 |
| C | 6.313184 | -1.408744 | -0.462731 |
| C | 6.375987 | 0.949644 | -0.957764 |
| C | 7.639905 | -1.352968 | -0.032129 |
| H | 5.771378 | -2.348485 | -0.405684 |
| C | 7.701801 | 1.007368 | -0.526937 |
| C | 8.339650 | -0.145652 | -0.065979 |
| H | 8.124429 | -2.253569 | 0.335959 |
| O | -1.336052 | -0.117366 | 0.455535 |
| O | -2.638663 | -1.308568 | 2.791640 |
| C | -1.929145 | -0.921531 | -0.264197 |
| C | -2.541840 | -2.206832 | 0.160609 |
| O | -2.160798 | -0.727380 | -1.566679 |
| C | -1.772554 | 0.542267 | -2.121233 |
| H | -1.984592 | 0.463435 | -3.187067 |
| H | -2.369150 | 1.342866 | -1.677047 |
| H | -0.709562 | 0.731421 | -1.953916 |
| S | -2.326130 | -2.461560 | 1.925459 |
| C | -3.444456 | -3.830958 | 2.288468 |
| H | -3.327839 | -4.052094 | 3.351670 |
| H | -3.204823 | -4.694249 | 1.666374 |
| H | -4.448945 | -3.451950 | 2.070658 |
| C | -0.689748 | -3.130113 | 2.323100 |
| H | -0.643148 | -3.197594 | 3.412919 |
| H | 0.063094 | -2.440923 | 1.931980 |
| H | -0.579125 | -4.109561 | 1.855277 |
| C | -2.195044 | -3.432004 | -0.665570 |
| C | -3.207733 | -4.254305 | -1.183782 |
| C | -0.852662 | -3.751855 | -0.942120 |
| C | -2.884776 | -5.378026 | -1.946677 |
| H | -4.247596 | -3.989082 | -1.021902 |
| C | -0.536580 | -4.876524 | -1.704142 |
| H | -0.050099 | -3.121948 | -0.564498 |
| C | -1.551021 | -5.695680 | -2.204812 |
| H | -3.681297 | -5.999425 | -2.346759 |
| H | 0.505021 | -5.111200 | -1.907456 |
| H | -1.302705 | -6.571049 | -2.799090 |
| C | -6.224506 | 0.319291 | -1.373766 |
| C | -6.270840 | 1.698274 | -1.579295 |

| | | | |
|---|---|---|---|
| C | -5.766883 | 2.574911 | -0.615798 |
| C | -5.228460 | 2.054168 | 0.563508 |
| C | -5.184344 | 0.674700 | 0.776920 |
| C | -5.676308 | -0.215190 | -0.194630 |
| H | -6.609588 | -0.360195 | -2.128507 |
| H | -6.699304 | 2.088800 | -2.499839 |
| H | -5.788691 | 3.648496 | -0.775468 |
| H | -4.852955 | 2.731173 | 1.326433 |
| H | -4.772441 | 0.279719 | 1.701199 |
| S | -5.619082 | -1.982440 | 0.046439 |
| H | -3.914405 | -2.063290 | 0.105819 |
| H | 5.879332 | 1.853852 | -1.301518 |
| H | 8.232177 | 1.955657 | -0.543014 |
| H | 9.370944 | -0.101888 | 0.273402 |
| C | 3.152744 | -0.064850 | 2.614377 |
| C | -0.521842 | 3.771865 | -0.017317 |
| C | -1.899717 | 3.825577 | 0.272626 |
| C | 0.118156 | 4.897944 | -0.553792 |
| C | -2.591124 | 5.002934 | 0.036549 |
| H | -2.426274 | 2.971499 | 0.684795 |
| C | -0.636586 | 6.044967 | -0.766477 |
| H | 1.170430 | 4.890679 | -0.792194 |
| C | -1.992928 | 6.143235 | -0.485512 |
| H | -2.551174 | 7.054681 | -0.658829 |
| F | -3.906806 | 5.052947 | 0.341012 |
| F | -0.013639 | 7.123773 | -1.282046 |
| C | 3.423274 | 1.312199 | 3.253749 |
| H | 2.531980 | 1.946981 | 3.231682 |
| H | 3.719521 | 1.183039 | 4.301590 |
| H | 4.225018 | 1.847399 | 2.734881 |
| C | 4.440539 | -0.908369 | 2.674602 |
| H | 5.264921 | -0.429289 | 2.136159 |
| H | 4.752885 | -1.037832 | 3.717464 |
| H | 4.291395 | -1.907997 | 2.249364 |
| C | 2.047617 | -0.775344 | 3.416776 |
| H | 1.088084 | -0.250083 | 3.344535 |
| H | 1.909343 | -1.804868 | 3.080007 |
| H | 2.325558 | -0.798395 | 4.477371 |

TS Conformation 102

B3LYP/6-31G(d) Energy = -3411.872227
M06-2X/def2tzvpp/IEFPCM(chloroform) Energy = -3411.88603
M06-2X/def2tzvpp/IEFPCM(chloroform)//B3LYP/6-31G(d) Quasiharmonic Free Energy = -3411.160422

Frequencies (Top 3 out of 291)

1. -919.1026 cm-1
2. 8.6950 cm-1
3. 12.5662 cm-1

B3LYP/6-31G(d) Molecular Geometry in Cartesian Coordinates

| | | | |
|---|---|---|---|
| C | 5.015220 | -0.819982 | 0.660670 |
| C | 3.949130 | -0.310452 | 1.661680 |
| H | 4.442400 | 0.564808 | 2.089940 |
| C | 2.415600 | 1.494289 | 0.865940 |
| N | 1.061411 | 1.706870 | 0.697730 |
| H | 0.453130 | 0.918030 | 0.936700 |
| N | 2.693040 | 0.172549 | 1.091070 |
| H | 1.894360 | -0.451741 | 1.152060 |

| | | | |
|---|---|---|---|
| C | 3.427449 | -1.823741 | -1.128500 |
| C | 5.848399 | -2.044542 | -1.258900 |
| C | 3.781479 | -3.194082 | -1.756350 |
| H | 2.660379 | -1.942971 | -0.361640 |
| C | 5.192149 | -2.952362 | -2.313200 |
| H | 6.479219 | -1.263453 | -1.695260 |
| H | 6.472619 | -2.598513 | -0.550960 |
| H | 3.063988 | -3.512961 | -2.516250 |
| H | 3.803178 | -3.953742 | -0.965440 |
| H | 5.127939 | -2.439722 | -3.278750 |
| H | 5.755178 | -3.877992 | -2.464270 |
| N | 4.712169 | -1.450802 | -0.517470 |
| O | 6.193990 | -0.684693 | 0.986400 |
| S | 3.595801 | 2.687659 | 0.825300 |
| C | 2.944020 | -0.830881 | -2.192120 |
| C | 1.819909 | -1.149301 | -2.969830 |
| C | 3.615170 | 0.369539 | -2.449840 |
| C | 1.405930 | -0.312221 | -4.006420 |
| H | 1.261839 | -2.062271 | -2.775910 |
| C | 3.192510 | 1.217509 | -3.476850 |
| C | 2.096360 | 0.874339 | -4.268660 |
| H | 0.545370 | -0.597350 | -4.606030 |
| O | -3.881089 | 1.677571 | -0.842220 |
| O | -1.815251 | -1.415719 | -3.089730 |
| C | -3.928490 | 0.546591 | -0.362100 |
| C | -3.244730 | -0.643609 | -0.916480 |
| O | -4.553290 | 0.265892 | 0.789110 |
| C | -5.145740 | 1.385132 | 1.480750 |
| H | -5.402970 | 1.000902 | 2.467300 |
| H | -4.436859 | 2.211222 | 1.552040 |
| H | -6.042189 | 1.722302 | 0.952230 |
| S | -2.477180 | -0.241469 | -2.477700 |
| C | -1.298810 | 1.094460 | -2.204530 |
| H | -0.834270 | 1.308290 | -3.168750 |
| H | -0.545180 | 0.708260 | -1.516390 |
| H | -1.822739 | 1.950471 | -1.783090 |
| C | -3.673620 | 0.452731 | -3.653900 |
| H | -4.403440 | -0.337418 | -3.843850 |
| H | -4.136030 | 1.326832 | -3.194280 |
| H | -3.132640 | 0.701311 | -4.570090 |
| C | -3.966231 | -1.961199 | -0.987360 |
| C | -5.366821 | -2.023028 | -1.103930 |
| C | -3.242621 | -3.164969 | -0.957720 |
| C | -6.023911 | -3.249688 | -1.170850 |
| H | -5.947691 | -1.105088 | -1.121210 |
| C | -3.901942 | -4.391959 | -1.039460 |
| H | -2.163091 | -3.136249 | -0.864670 |
| C | -5.292332 | -4.439298 | -1.143030 |
| H | -7.107381 | -3.276097 | -1.250150 |
| H | -3.324852 | -5.312169 | -1.015330 |
| H | -5.804402 | -5.396018 | -1.199260 |
| C | -1.365460 | -0.088590 | 3.586770 |
| C | -2.051490 | -0.004309 | 4.800310 |
| C | -3.160130 | -0.816539 | 5.040070 |
| C | -3.571261 | -1.722029 | 4.058430 |
| C | -2.882261 | -1.819009 | 2.849680 |
| C | -1.774061 | -0.995849 | 2.597140 |
| H | -0.506460 | 0.552420 | 3.409910 |
| H | -1.717020 | 0.702541 | 5.555330 |
| H | -3.695160 | -0.749779 | 5.983480 |
| H | -4.429341 | -2.365598 | 4.236320 |
| H | -3.202431 | -2.532839 | 2.097450 |

| | | | |
|---|---|---|---|
| S | -0.870381 | -1.125690 | 1.052100 |
| H | -2.062420 | -0.855619 | 0.018170 |
| H | 4.469000 | 0.646938 | -1.841000 |
| H | 3.730091 | 2.144168 | -3.658960 |
| H | 1.784780 | 1.523659 | -5.083320 |
| C | 3.692559 | -1.318692 | 2.845380 |
| C | 0.351341 | 2.893340 | 0.439070 |
| C | 0.822661 | 3.914620 | -0.399020 |
| C | -0.935369 | 2.987670 | 1.000620 |
| C | -0.003228 | 5.005750 | -0.634160 |
| H | 1.805911 | 3.870579 | -0.843900 |
| C | -1.713859 | 4.098931 | 0.715910 |
| H | -1.317369 | 2.218390 | 1.662530 |
| C | -1.281858 | 5.136130 | -0.100850 |
| H | -1.899848 | 6.002081 | -0.302780 |
| F | 0.448802 | 5.985260 | -1.443590 |
| F | -2.943588 | 4.184561 | 1.272310 |
| C | 5.027559 | -1.659192 | 3.540280 |
| H | 5.537540 | -0.754092 | 3.885870 |
| H | 4.827999 | -2.294432 | 4.411740 |
| H | 5.717699 | -2.186812 | 2.878900 |
| C | 3.032919 | -2.629391 | 2.370090 |
| H | 3.663009 | -3.155092 | 1.643520 |
| H | 2.043139 | -2.473521 | 1.922000 |
| H | 2.885379 | -3.301231 | 3.223640 |
| C | 2.780170 | -0.631471 | 3.883790 |
| H | 2.670679 | -1.274051 | 4.764920 |
| H | 1.774340 | -0.438361 | 3.497720 |
| H | 3.206780 | 0.322979 | 4.213120 |

TS Conformation 103

B3LYP/6-31G(d) Energy = -3411.883286
M06-2X/def2tzvpp/IEFPCM(chloroform) Energy = -3411.885409
M06-2X/def2tzvpp/IEFPCM(chloroform)//B3LYP/6-31G(d) Quasiharmonic Free Energy = -3411.160209

Frequencies (Top 3 out of 291)

1. -950.6466 cm-1
2. 6.9138 cm-1
3. 9.5441 cm-1

B3LYP/6-31G(d) Molecular Geometry in Cartesian Coordinates

| | | | |
|---|---|---|---|
| C | 2.444210 | -1.238601 | 1.048486 |
| C | 3.107223 | 0.144728 | 1.214279 |
| H | 3.816758 | 0.343586 | 0.412697 |
| C | 1.955678 | 2.146172 | 0.176754 |
| N | 0.738642 | 2.792055 | 0.255217 |
| H | 0.064744 | 2.360653 | 0.882233 |
| N | 2.019982 | 1.114855 | 1.064099 |
| H | 1.181808 | 0.863290 | 1.579871 |
| C | 3.463521 | -1.545750 | -1.257144 |
| C | 2.039793 | -3.274281 | -0.230249 |
| C | 2.803730 | -2.431872 | -2.346011 |
| H | 3.232530 | -0.494448 | -1.450389 |
| C | 2.496618 | -3.746366 | -1.615370 |
| H | 2.284168 | -3.971264 | 0.577574 |
| H | 0.960791 | -3.090294 | -0.202156 |
| H | 3.450632 | -2.553611 | -3.218917 |
| H | 1.868355 | -1.960345 | -2.668346 |

| | | | |
|---|---|---|---|
| H | 3.399056 | -4.363960 | -1.541931 |
| H | 1.723947 | -4.336007 | -2.116270 |
| N | 2.758748 | -1.994452 | -0.032204 |
| O | 1.615113 | -1.622980 | 1.884555 |
| S | 3.220929 | 2.566363 | -0.853818 |
| C | 4.978095 | -1.702245 | -1.201878 |
| C | 5.606362 | -2.704660 | -0.451969 |
| C | 5.773889 | -0.840432 | -1.969587 |
| C | 6.994792 | -2.851725 | -0.481343 |
| H | 5.008973 | -3.361760 | 0.173187 |
| C | 7.160955 | -0.985359 | -2.000072 |
| C | 7.776500 | -1.995046 | -1.257759 |
| H | 7.465114 | -3.634734 | 0.107989 |
| O | -5.374690 | -0.078420 | -0.237072 |
| O | -1.633807 | 1.360269 | 1.392246 |
| C | -4.742630 | -0.895659 | 0.429992 |
| C | -3.362455 | -0.683765 | 0.926090 |
| O | -5.205930 | -2.115738 | 0.732455 |
| C | -6.476787 | -2.464054 | 0.148377 |
| H | -6.734196 | -3.429557 | 0.583728 |
| H | -7.231676 | -1.713616 | 0.393452 |
| H | -6.374787 | -2.545690 | -0.936306 |
| S | -2.936019 | 1.063066 | 0.737273 |
| C | -2.882933 | 1.450280 | -1.022298 |
| H | -2.529472 | 2.480932 | -1.104660 |
| H | -2.173789 | 0.735112 | -1.456813 |
| H | -3.883420 | 1.305458 | -1.429438 |
| C | -4.191375 | 2.182063 | 1.419563 |
| H | -5.152143 | 1.929465 | 0.970892 |
| H | -4.189947 | 2.016442 | 2.499159 |
| H | -3.882397 | 3.204189 | 1.183551 |
| C | -2.942106 | -1.197381 | 2.274203 |
| C | -3.844420 | -1.256661 | 3.351531 |
| C | -1.614761 | -1.602058 | 2.479066 |
| C | -3.430810 | -1.725120 | 4.596237 |
| H | -4.879775 | -0.958732 | 3.207805 |
| C | -1.199454 | -2.059618 | 3.729744 |
| H | -0.894707 | -1.570942 | 1.670050 |
| C | -2.104921 | -2.124509 | 4.788708 |
| H | -4.141857 | -1.775670 | 5.416595 |
| H | -0.164715 | -2.361823 | 3.856270 |
| H | -1.783087 | -2.485890 | 5.761929 |
| C | -2.797671 | -1.904843 | -3.687066 |
| C | -3.744066 | -2.381724 | -4.594870 |
| C | -4.635380 | -3.390210 | -4.223650 |
| C | -4.562847 | -3.922498 | -2.933993 |
| C | -3.612648 | -3.452872 | -2.025207 |
| C | -2.716579 | -2.429889 | -2.385213 |
| H | -2.104667 | -1.122778 | -3.984768 |
| H | -3.784403 | -1.961536 | -5.597204 |
| H | -5.371306 | -3.762053 | -4.931588 |
| H | -5.238953 | -4.721524 | -2.636891 |
| H | -3.549804 | -3.883721 | -1.030321 |
| S | -1.477152 | -1.840025 | -1.241865 |
| H | -2.521964 | -1.248091 | -0.039804 |
| H | 5.301354 | -0.043212 | -2.538817 |
| H | 7.760920 | -0.303009 | -2.596464 |
| H | 8.857241 | -2.107313 | -1.277042 |
| C | 3.877554 | 0.309948 | 2.575084 |
| C | 0.213939 | 3.937319 | -0.360381 |
| C | 0.738500 | 4.573684 | -1.494472 |
| C | -0.971511 | 4.435547 | 0.222712 |

| | | | |
|---|---|---|---|
| C | 0.061699 | 5.677010 | -2.002162 |
| H | 1.645324 | 4.225445 | -1.965856 |
| C | -1.606596 | 5.525919 | -0.347040 |
| H | -1.372460 | 3.995113 | 1.128369 |
| C | -1.119075 | 6.183649 | -1.468802 |
| H | -1.622352 | 7.039429 | -1.900775 |
| F | 0.570864 | 6.281173 | -3.092393 |
| F | -2.756737 | 5.957368 | 0.221620 |
| C | 4.818080 | -0.895776 | 2.766556 |
| H | 5.491731 | -1.024530 | 1.912112 |
| H | 5.436654 | -0.745924 | 3.659278 |
| H | 4.255958 | -1.825883 | 2.904773 |
| C | 2.926277 | 0.411507 | 3.783468 |
| H | 2.288722 | 1.301062 | 3.715671 |
| H | 2.285203 | -0.468715 | 3.864578 |
| H | 3.514204 | 0.504624 | 4.704525 |
| C | 4.720931 | 1.597833 | 2.493466 |
| H | 5.461463 | 1.543876 | 1.688017 |
| H | 4.096143 | 2.477830 | 2.309799 |
| H | 5.255793 | 1.754420 | 3.438225 |

TS Conformation 104

B3LYP/6-31G(d) Energy = -3411.88057
M06-2X/def2tzvpp/IEFPCM(chloroform) Energy = -3411.88615
M06-2X/def2tzvpp/IEFPCM(chloroform)//B3LYP/6-31G(d) Quasiharmonic Free Energy = -3411.159996

Frequencies (Top 3 out of 291)

1. -1008.5603 cm-1
2. 7.9748 cm-1
3. 12.8180 cm-1

B3LYP/6-31G(d) Molecular Geometry in Cartesian Coordinates

| | | | |
|---|---|---|---|
| C | 2.415085 | -1.251572 | 0.353095 |
| C | 2.649951 | -0.063611 | 1.310776 |
| H | 3.540582 | 0.495492 | 1.027201 |
| C | 1.557801 | 2.081320 | 0.547203 |
| N | 0.308842 | 2.481245 | 0.125770 |
| H | -0.332193 | 1.711477 | -0.077786 |
| N | 1.512674 | 0.832717 | 1.095648 |
| H | 0.603438 | 0.382389 | 1.156602 |
| C | 4.160022 | -0.375478 | -1.283456 |
| C | 2.825242 | -2.389377 | -1.763136 |
| C | 4.102756 | -0.639509 | -2.810279 |
| H | 3.818979 | 0.641887 | -1.073473 |
| C | 3.809321 | -2.142937 | -2.913623 |
| H | 2.911243 | -3.385863 | -1.318855 |
| H | 1.781413 | -2.259198 | -2.072602 |
| H | 5.026132 | -0.337212 | -3.310983 |
| H | 3.275912 | -0.062213 | -3.240964 |
| H | 4.729372 | -2.719101 | -2.764746 |
| H | 3.388749 | -2.430204 | -3.881673 |
| N | 3.172717 | -1.352795 | -0.768050 |
| O | 1.504617 | -2.060492 | 0.578406 |
| S | 2.976657 | 2.982774 | 0.397032 |
| C | 5.560640 | -0.541550 | -0.706011 |
| C | 6.406136 | 0.576101 | -0.661904 |
| C | 6.052805 | -1.773649 | -0.257758 |
| C | 7.716141 | 0.462216 | -0.196806 |

| | | | |
|---|---|---|---|
| H | 6.028298 | 1.543933 | -0.983047 |
| C | 7.364303 | -1.889505 | 0.208327 |
| C | 8.201227 | -0.773222 | 0.237107 |
| H | 8.354193 | 1.341376 | -0.163790 |
| O | -1.161636 | 0.087915 | -0.850594 |
| O | -0.892417 | -2.441266 | -2.844734 |
| C | -1.713781 | -0.712894 | -0.090701 |
| C | -2.530093 | -1.845036 | -0.587543 |
| O | -1.585832 | -0.476984 | 1.223523 |
| C | -2.396553 | -1.089911 | 2.250254 |
| H | -3.437051 | -1.163522 | 1.931924 |
| H | -1.995520 | -2.068966 | 2.514809 |
| H | -2.325426 | -0.409435 | 3.099174 |
| S | -2.224864 | -2.011194 | -2.360551 |
| C | -3.473524 | -3.211123 | -2.872889 |
| H | -3.243813 | -4.157712 | -2.383293 |
| H | -3.381647 | -3.306334 | -3.956832 |
| H | -4.464505 | -2.852047 | -2.584250 |
| C | -2.790498 | -0.509448 | -3.194839 |
| H | -2.877686 | -0.758401 | -4.255112 |
| H | -2.030147 | 0.248446 | -3.021092 |
| H | -3.755858 | -0.227564 | -2.758990 |
| C | -2.436951 | -3.199696 | 0.080198 |
| C | -3.605957 | -3.895616 | 0.434147 |
| C | -1.183964 | -3.780309 | 0.345313 |
| C | -3.522791 | -5.146894 | 1.044910 |
| H | -4.575635 | -3.441235 | 0.249596 |
| C | -1.110590 | -5.030687 | 0.961725 |
| H | -0.269060 | -3.250620 | 0.095551 |
| C | -2.275513 | -5.717321 | 1.309325 |
| H | -4.433434 | -5.672058 | 1.320216 |
| H | -0.136607 | -5.464200 | 1.170681 |
| H | -2.212546 | -6.690611 | 1.788863 |
| C | -6.225507 | 0.091788 | 1.728792 |
| C | -6.180706 | 1.035252 | 2.756313 |
| C | -5.347929 | 2.152084 | 2.656710 |
| C | -4.567030 | 2.323402 | 1.511857 |
| C | -4.619552 | 1.388871 | 0.476670 |
| C | -5.442546 | 0.253721 | 0.572698 |
| H | -6.870503 | -0.777983 | 1.812240 |
| H | -6.798109 | 0.893556 | 3.640181 |
| H | -5.312033 | 2.883621 | 3.459355 |
| H | -3.919121 | 3.190922 | 1.414625 |
| H | -4.024951 | 1.544911 | -0.418775 |
| S | -5.511318 | -0.965644 | -0.734053 |
| H | -3.904119 | -1.423437 | -0.623633 |
| H | 5.402498 | -2.643655 | -0.254718 |
| H | 7.729155 | -2.853419 | 0.553803 |
| H | 9.220239 | -0.862833 | 0.603858 |
| C | 2.823031 | -0.473760 | 2.813762 |
| C | -0.165508 | 3.733972 | -0.290478 |
| C | -1.312987 | 3.723104 | -1.105861 |
| C | 0.387864 | 4.958374 | 0.111020 |
| C | -1.869375 | 4.928001 | -1.505956 |
| H | -1.757293 | 2.789465 | -1.432506 |
| C | -0.214552 | 6.128927 | -0.329809 |
| H | 1.267468 | 5.006407 | 0.735516 |
| C | -1.344697 | 6.160978 | -1.139296 |
| H | -1.788875 | 7.093042 | -1.465345 |
| F | -2.965237 | 4.898853 | -2.294078 |
| F | 0.320765 | 7.304040 | 0.058994 |
| C | 3.313605 | 0.767493 | 3.585512 |

| | | | |
|---|---|---|---|
| H | 2.609774 | 1.600587 | 3.490337 |
| H | 3.418284 | 0.530034 | 4.651223 |
| H | 4.285826 | 1.112183 | 3.216424 |
| C | 3.889567 | -1.582270 | 2.907445 |
| H | 4.830631 | -1.278996 | 2.434351 |
| H | 4.103021 | -1.807269 | 3.959047 |
| H | 3.548359 | -2.508237 | 2.431744 |
| C | 1.510561 | -0.971408 | 3.451181 |
| H | 0.749315 | -0.182238 | 3.459236 |
| H | 1.110641 | -1.836265 | 2.917768 |
| H | 1.695055 | -1.254398 | 4.494664 |

TS Conformation 105

B3LYP/6-31G(d) Energy = -3411.86828
M06-2X/def2tzvpp/IEFPCM(chloroform) Energy = -3411.884696
M06-2X/def2tzvpp/IEFPCM(chloroform)//B3LYP/6-31G(d) Quasiharmonic Free Energy = -3411.159967

Frequencies (Top 3 out of 291)

1. -961.1099 cm-1
2. 5.1818 cm-1
3. 9.9296 cm-1

B3LYP/6-31G(d) Molecular Geometry in Cartesian Coordinates

| | | | |
|---|---|---|---|
| C | 2.979762 | -3.571441 | 0.616483 |
| C | 2.507275 | -2.526638 | 1.661058 |
| H | 3.407632 | -2.390611 | 2.263865 |
| C | 3.050954 | -0.142610 | 1.137989 |
| N | 2.409447 | 1.039472 | 0.834555 |
| H | 1.395845 | 1.008923 | 0.883200 |
| N | 2.184148 | -1.202046 | 1.143498 |
| H | 1.217723 | -1.013828 | 0.906039 |
| C | 1.497828 | -2.821267 | -1.372259 |
| C | 3.039864 | -4.710147 | -1.518726 |
| C | 0.935995 | -3.886387 | -2.346439 |
| H | 0.718568 | -2.530252 | -0.665082 |
| C | 2.200217 | -4.626825 | -2.801120 |
| H | 4.116915 | -4.627120 | -1.694053 |
| H | 2.877227 | -5.644267 | -0.970546 |
| H | 0.365249 | -3.443272 | -3.165396 |
| H | 0.270982 | -4.561796 | -1.794457 |
| H | 2.720751 | -4.041886 | -3.567150 |
| H | 1.991491 | -5.615875 | -3.219390 |
| N | 2.560104 | -3.581322 | -0.686842 |
| O | 3.765357 | -4.433242 | 1.010458 |
| S | 4.685114 | -0.302914 | 1.495381 |
| C | 1.990698 | -1.581472 | -2.121957 |
| C | 3.338473 | -1.212842 | -2.165184 |
| C | 1.055822 | -0.804212 | -2.824768 |
| C | 3.748002 | -0.096190 | -2.899562 |
| H | 4.070023 | -1.790434 | -1.609456 |
| C | 1.465792 | 0.305635 | -3.564272 |
| C | 2.816238 | 0.663763 | -3.605649 |
| H | 4.799808 | 0.176253 | -2.915960 |
| O | -4.921256 | 1.236353 | 1.759323 |
| O | -0.604780 | 0.855033 | 1.629528 |
| C | -4.399220 | 1.547276 | 0.690359 |
| C | -2.956198 | 1.408306 | 0.380642 |
| O | -5.089643 | 2.005788 | -0.361974 |

| | | | |
|---|---|---|---|
| C | -6.522278 | 2.037216 | -0.204956 |
| H | -6.799765 | 2.604576 | 0.686138 |
| H | -6.896474 | 2.522652 | -1.106050 |
| H | -6.907766 | 1.017690 | -0.127483 |
| S | -2.064505 | 0.970180 | 1.884251 |
| C | -2.341167 | 2.166146 | 3.216506 |
| H | -3.413647 | 2.214849 | 3.408877 |
| H | -1.776409 | 1.826427 | 4.088057 |
| H | -1.952216 | 3.121223 | 2.856438 |
| C | -2.708301 | -0.592166 | 2.514306 |
| H | -2.059658 | -0.877759 | 3.345648 |
| H | -3.747077 | -0.447828 | 2.807153 |
| H | -2.619873 | -1.295944 | 1.679696 |
| C | -2.246740 | 2.512810 | -0.354296 |
| C | -1.203130 | 2.213380 | -1.242555 |
| C | -2.589747 | 3.860938 | -0.142327 |
| C | -0.518315 | 3.236905 | -1.898611 |
| H | -0.927623 | 1.180735 | -1.425583 |
| C | -1.910138 | 4.880149 | -0.804885 |
| H | -3.406373 | 4.111478 | 0.530340 |
| C | -0.866886 | 4.569906 | -1.680665 |
| H | 0.296315 | 2.984848 | -2.570560 |
| H | -2.188060 | 5.915930 | -0.631544 |
| H | -0.325710 | 5.365413 | -2.184262 |
| C | -5.190279 | -1.213241 | -1.655653 |
| C | -6.491751 | -1.717509 | -1.629556 |
| C | -6.771560 | -2.927582 | -0.990161 |
| C | -5.732577 | -3.630180 | -0.377167 |
| C | -4.431498 | -3.125645 | -0.393285 |
| C | -4.138766 | -1.907117 | -1.030083 |
| H | -4.976479 | -0.282144 | -2.171949 |
| H | -7.288469 | -1.169471 | -2.128031 |
| H | -7.784224 | -3.321414 | -0.977444 |
| H | -5.933754 | -4.577347 | 0.117875 |
| H | -3.627267 | -3.680365 | 0.082342 |
| S | -2.466480 | -1.278463 | -1.065389 |
| H | -2.776798 | 0.209349 | -0.329363 |
| H | 0.001581 | -1.073611 | -2.799621 |
| H | 0.729137 | 0.883263 | -4.117800 |
| H | 3.137788 | 1.526503 | -4.183181 |
| C | 1.391027 | -3.071858 | 2.627373 |
| C | 2.899472 | 2.329244 | 0.567386 |
| C | 4.210862 | 2.615029 | 0.160888 |
| C | 1.962010 | 3.374047 | 0.669946 |
| C | 4.539799 | 3.934023 | -0.121081 |
| H | 4.958027 | 1.841104 | 0.075187 |
| C | 2.352553 | 4.666338 | 0.360588 |
| H | 0.939122 | 3.183269 | 0.972811 |
| C | 3.642632 | 4.992931 | -0.036745 |
| H | 3.933728 | 6.010257 | -0.266471 |
| F | 5.801961 | 4.202653 | -0.513306 |
| F | 1.431578 | 5.653344 | 0.448160 |
| C | 1.097351 | -1.992951 | 3.691831 |
| H | 0.671849 | -1.082426 | 3.256682 |
| H | 0.386223 | -2.384667 | 4.430209 |
| H | 2.010569 | -1.708541 | 4.226815 |
| C | 1.905526 | -4.331217 | 3.355489 |
| H | 2.125917 | -5.145808 | 2.663246 |
| H | 2.825230 | -4.118586 | 3.910656 |
| H | 1.146700 | -4.674417 | 4.069719 |
| C | 0.091602 | -3.430354 | 1.879952 |
| H | -0.355187 | -2.576092 | 1.356000 |

| | | | |
|---|---|---|---|
| H | 0.258634 | -4.222889 | 1.142117 |
| H | -0.658655 | -3.800232 | 2.590010 |

TS Conformation 106

B3LYP/6-31G(d) Energy = -3411.88258
M06-2X/def2tzvpp/IEFPCM(chloroform) Energy = -3411.887356
M06-2X/def2tzvpp/IEFPCM(chloroform)//B3LYP/6-31G(d) Quasiharmonic Free Energy = -3411.15996

Frequencies (Top 3 out of 291)

1. -898.9923 cm-1
2. 9.2797 cm-1
3. 16.1738 cm-1

B3LYP/6-31G(d) Molecular Geometry in Cartesian Coordinates

| | | | |
|---|---|---|---|
| C | -2.915715 | -1.501663 | -0.056386 |
| C | -3.287409 | -0.410831 | -1.080627 |
| H | -4.047093 | 0.257532 | -0.677571 |
| C | -1.932360 | 1.722737 | -0.964743 |
| N | -0.610963 | 2.092903 | -0.984963 |
| H | 0.043229 | 1.317822 | -1.053661 |
| N | -2.069958 | 0.385787 | -1.242967 |
| H | -1.216901 | -0.165374 | -1.223917 |
| C | -4.262893 | -0.340022 | 1.762411 |
| C | -2.987748 | -2.398907 | 2.212040 |
| C | -3.940631 | -0.465890 | 3.273750 |
| H | -3.892349 | 0.617242 | 1.386446 |
| C | -3.729638 | -1.971645 | 3.483845 |
| H | -3.195751 | -3.427079 | 1.900945 |
| H | -1.901199 | -2.300788 | 2.325684 |
| H | -4.735591 | -0.044912 | 3.894078 |
| H | -3.014265 | 0.079244 | 3.489630 |
| H | -4.694018 | -2.485581 | 3.559015 |
| H | -3.158076 | -2.200087 | 4.387758 |
| N | -3.455320 | -1.446426 | 1.183786 |
| O | -2.083956 | -2.379402 | -0.353411 |
| S | -3.246146 | 2.736509 | -0.664846 |
| C | -5.753322 | -0.432700 | 1.463321 |
| C | -6.494942 | 0.753721 | 1.375914 |
| C | -6.422326 | -1.655413 | 1.322521 |
| C | -7.874083 | 0.719264 | 1.168322 |
| H | -5.984269 | 1.710101 | 1.459108 |
| C | -7.802534 | -1.691523 | 1.115012 |
| C | -8.533344 | -0.504686 | 1.040704 |
| H | -8.430922 | 1.649662 | 1.096866 |
| O | 2.853317 | 0.186439 | -1.955075 |
| O | 0.639486 | -0.556301 | -0.261606 |
| C | 3.597438 | -0.472291 | -1.234682 |
| C | 3.177428 | -1.642917 | -0.407806 |
| O | 4.895963 | -0.213816 | -1.065107 |
| C | 5.396520 | 0.968601 | -1.723033 |
| H | 6.459078 | 0.995483 | -1.484710 |
| H | 5.240320 | 0.899537 | -2.802532 |
| H | 4.895441 | 1.858095 | -1.334666 |
| S | 1.393620 | -1.798782 | -0.565112 |
| C | 0.875877 | -2.446141 | -2.175595 |
| H | 1.421340 | -3.368484 | -2.383712 |
| H | 1.120802 | -1.668911 | -2.901575 |
| H | -0.201898 | -2.613079 | -2.105072 |

| | | | |
|---|---|---|---|
| C | 0.919885 | -3.088840 | 0.594083 |
| H | 1.436414 | -4.018431 | 0.351235 |
| H | -0.167734 | -3.169220 | 0.500977 |
| H | 1.211613 | -2.718219 | 1.581098 |
| C | 3.915777 | -2.942073 | -0.646709 |
| C | 4.252370 | -3.342424 | -1.953058 |
| C | 4.295327 | -3.761606 | 0.428011 |
| C | 4.937083 | -4.534738 | -2.180899 |
| H | 3.992307 | -2.705221 | -2.795539 |
| C | 4.976490 | -4.957975 | 0.196229 |
| H | 4.094027 | -3.433495 | 1.443626 |
| C | 5.294460 | -5.350358 | -1.104522 |
| H | 5.194838 | -4.823993 | -3.196053 |
| H | 5.270621 | -5.577132 | 1.039240 |
| H | 5.828385 | -6.280337 | -1.279852 |
| C | 3.818878 | 1.487367 | 1.849465 |
| C | 4.558059 | 2.671746 | 1.830396 |
| C | 5.710721 | 2.797726 | 2.609061 |
| C | 6.116438 | 1.724729 | 3.405626 |
| C | 5.391439 | 0.533135 | 3.410920 |
| C | 4.232000 | 0.392341 | 2.629009 |
| H | 2.904268 | 1.410813 | 1.267426 |
| H | 4.232283 | 3.495587 | 1.201276 |
| H | 6.281842 | 3.722528 | 2.598996 |
| H | 7.009276 | 1.810596 | 4.021214 |
| H | 5.721578 | -0.304864 | 4.017915 |
| S | 3.326706 | -1.144353 | 2.639134 |
| H | 3.260844 | -1.349532 | 0.932510 |
| H | -5.861081 | -2.584943 | 1.353327 |
| H | -8.305254 | -2.649004 | 1.006093 |
| H | -9.606895 | -0.533269 | 0.875260 |
| C | -3.853240 | -0.963930 | -2.436945 |
| C | 0.057095 | 3.327849 | -0.890968 |
| C | -0.551928 | 4.569145 | -0.663116 |
| C | 1.456000 | 3.251991 | -1.045069 |
| C | 0.261002 | 5.694473 | -0.591909 |
| H | -1.620060 | 4.662214 | -0.539344 |
| C | 2.202401 | 4.412463 | -0.953860 |
| H | 1.953850 | 2.309858 | -1.252491 |
| C | 1.643449 | 5.663807 | -0.725395 |
| H | 2.246796 | 6.560158 | -0.655558 |
| F | -0.329619 | 6.886386 | -0.371678 |
| F | 3.549426 | 4.320205 | -1.083364 |
| C | -2.801646 | -1.724844 | -3.265441 |
| H | -2.470037 | -2.632664 | -2.755627 |
| H | -3.233804 | -2.012659 | -4.231300 |
| H | -1.929750 | -1.093867 | -3.475161 |
| C | -4.357570 | 0.237307 | -3.261520 |
| H | -3.550102 | 0.944910 | -3.473957 |
| H | -4.764438 | -0.111728 | -4.218325 |
| H | -5.145825 | 0.784566 | -2.734333 |
| C | -5.037565 | -1.902503 | -2.136097 |
| H | -4.716738 | -2.796179 | -1.588130 |
| H | -5.812644 | -1.400673 | -1.547084 |
| H | -5.495845 | -2.237611 | -3.073947 |

TS Conformation 107

B3LYP/6-31G(d) Energy = -3411.873924
M06-2X/def2tzvpp/IEFPCM(chloroform) Energy = -3411.884313
M06-2X/def2tzvpp/IEFPCM(chloroform)//B3LYP/6-31G(d) Quasiharmonic Free Energy = -3411.158702

Frequencies (Top 3 out of 291)

1. -973.8802 cm-1
2. 7.0669 cm-1
3. 8.3654 cm-1

B3LYP/6-31G(d) Molecular Geometry in Cartesian Coordinates

| | | | |
|---|---|---|---|
| C | -3.790203 | -1.288333 | 0.973761 |
| C | -2.570720 | -1.167295 | 1.926936 |
| H | -2.762746 | -1.967932 | 2.645756 |
| C | -0.758008 | -2.687750 | 1.130968 |
| N | 0.594104 | -2.647771 | 0.832995 |
| H | 1.090208 | -1.825146 | 1.167338 |
| N | -1.257238 | -1.439429 | 1.351906 |
| H | -0.686542 | -0.652246 | 1.061457 |
| C | -4.933567 | -1.301436 | -1.168779 |
| C | -2.503629 | -0.964210 | -1.230814 |
| C | -4.385509 | -1.572272 | -2.590886 |
| H | -5.434783 | -2.185437 | -0.765874 |
| C | -3.082338 | -0.765244 | -2.638742 |
| H | -1.859043 | -0.136046 | -0.925920 |
| H | -1.919688 | -1.891203 | -1.188127 |
| H | -5.100527 | -1.291072 | -3.368336 |
| H | -4.167290 | -2.641521 | -2.698357 |
| H | -3.297193 | 0.294014 | -2.820629 |
| H | -2.392861 | -1.107543 | -3.417445 |
| N | -3.702352 | -1.068458 | -0.377241 |
| O | -4.875347 | -1.543575 | 1.491051 |
| S | -1.692457 | -4.076641 | 1.242214 |
| C | -5.924002 | -0.144139 | -1.119374 |
| C | -7.294939 | -0.419772 | -1.198378 |
| C | -5.517047 | 1.194212 | -1.058524 |
| C | -8.234510 | 0.610523 | -1.225110 |
| H | -7.629114 | -1.454572 | -1.227594 |
| C | -6.454110 | 2.229129 | -1.081971 |
| C | -7.816687 | 1.941551 | -1.168524 |
| H | -9.293863 | 0.373119 | -1.279589 |
| O | 0.658549 | 0.869978 | -0.188506 |
| O | 2.471647 | -0.364752 | 2.057297 |
| C | 1.712610 | 1.137115 | -0.765382 |
| C | 2.998208 | 1.440599 | -0.087243 |
| O | 1.828190 | 1.234652 | -2.093355 |
| C | 0.618283 | 1.051599 | -2.852873 |
| H | 0.917191 | 1.162430 | -3.894840 |
| H | 0.204244 | 0.056372 | -2.676029 |
| H | -0.115896 | 1.812649 | -2.577660 |
| S | 2.818357 | 1.026756 | 1.644595 |
| C | 1.676553 | 2.220060 | 2.372879 |
| H | 1.672499 | 2.030737 | 3.448590 |
| H | 0.698277 | 2.036106 | 1.933116 |
| H | 2.047598 | 3.219558 | 2.120802 |
| C | 4.400620 | 1.511647 | 2.357373 |
| H | 4.576490 | 2.563338 | 2.112911 |
| H | 4.318967 | 1.354249 | 3.434867 |
| H | 5.169115 | 0.868737 | 1.927113 |
| C | 4.272769 | 0.881704 | -0.685047 |
| C | 4.492732 | -0.504949 | -0.734076 |
| C | 5.254756 | 1.744059 | -1.195153 |
| C | 5.679582 | -1.022004 | -1.251463 |
| H | 3.731365 | -1.186269 | -0.369152 |

| | | | |
|---|---|---|---|
| C | 6.432314 | 1.222803 | -1.734613 |
| H | 5.088987 | 2.816648 | -1.181995 |
| C | 6.652685 | -0.155697 | -1.754567 |
| H | 5.835475 | -2.096846 | -1.250620 |
| H | 7.181875 | 1.900049 | -2.134719 |
| H | 7.577129 | -0.553970 | -2.163903 |
| C | 0.953961 | 5.730291 | 0.640974 |
| C | -0.334413 | 6.126382 | 0.278453 |
| C | -0.852639 | 5.788606 | -0.973018 |
| C | -0.062761 | 5.058628 | -1.864113 |
| C | 1.229038 | 4.667146 | -1.508952 |
| C | 1.755174 | 4.990513 | -0.245447 |
| H | 1.353093 | 6.002862 | 1.614219 |
| H | -0.934660 | 6.702525 | 0.978625 |
| H | -1.855580 | 6.097989 | -1.254057 |
| H | -0.447701 | 4.805897 | -2.849692 |
| H | 1.846079 | 4.118336 | -2.214295 |
| S | 3.412896 | 4.501247 | 0.214588 |
| H | 3.160366 | 2.841433 | -0.001954 |
| H | -4.460081 | 1.430736 | -0.975968 |
| H | -6.117392 | 3.261514 | -1.028552 |
| H | -8.546937 | 2.746283 | -1.183170 |
| C | -2.554467 | 0.185602 | 2.729248 |
| C | 1.432974 | -3.633118 | 0.287868 |
| C | 0.981950 | -4.652880 | -0.563344 |
| C | 2.808555 | -3.510847 | 0.556974 |
| C | 1.919163 | -5.514173 | -1.116451 |
| H | -0.066877 | -4.787006 | -0.782800 |
| C | 3.694026 | -4.386762 | -0.052909 |
| H | 3.180970 | -2.752042 | 1.235826 |
| C | 3.289237 | -5.411664 | -0.896295 |
| H | 3.995153 | -6.092557 | -1.354962 |
| F | 1.480069 | -6.490196 | -1.935208 |
| F | 5.018618 | -4.222723 | 0.178803 |
| C | -2.392778 | 1.402609 | 1.795148 |
| H | -3.246061 | 1.491201 | 1.114624 |
| H | -2.349033 | 2.325736 | 2.385664 |
| H | -1.482801 | 1.362969 | 1.184186 |
| C | -1.393893 | 0.137004 | 3.747142 |
| H | -1.505606 | -0.716749 | 4.425542 |
| H | -0.411836 | 0.048692 | 3.271280 |
| H | -1.395866 | 1.049244 | 4.356079 |
| C | -3.866305 | 0.347767 | 3.525013 |
| H | -4.738233 | 0.418223 | 2.873057 |
| H | -4.029075 | -0.500941 | 4.197788 |
| H | -3.808626 | 1.259094 | 4.133037 |

TS Conformation 108

B3LYP/6-31G(d) Energy = -3411.873924
M06-2X/def2tzvpp/IEFPCM(chloroform) Energy = -3411.884309
M06-2X/def2tzvpp/IEFPCM(chloroform)//B3LYP/6-31G(d) Quasiharmonic Free Energy = -3411.158695

Frequencies (Top 3 out of 291)

1. -973.9087 cm-1
2. 7.0756 cm-1
3. 8.3705 cm-1

B3LYP/6-31G(d) Molecular Geometry in Cartesian Coordinates

| | | | |
|---|---|---|---|
| C | -3.790084 | -1.288823 | 0.973849 |
| C | -2.570595 | -1.168046 | 1.927052 |
| H | -2.762587 | -1.968896 | 2.645644 |
| C | -0.757717 | -2.688224 | 1.130900 |
| N | 0.594381 | -2.648031 | 0.832904 |
| H | 1.090363 | -1.825348 | 1.167283 |
| N | -1.257109 | -1.439986 | 1.351942 |
| H | -0.686528 | -0.652706 | 1.061533 |
| C | -4.933433 | -1.301735 | -1.168697 |
| C | -2.503444 | -0.964914 | -1.230739 |
| C | -4.385397 | -1.572865 | -2.590752 |
| H | -5.434888 | -2.185557 | -0.765698 |
| C | -3.082130 | -0.766002 | -2.638686 |
| H | -1.858780 | -0.136785 | -0.925915 |
| H | -1.919600 | -1.891964 | -1.187973 |
| H | -5.100363 | -1.291693 | -3.368260 |
| H | -4.167305 | -2.642158 | -2.698050 |
| H | -3.296866 | 0.293267 | -2.820660 |
| H | -2.392692 | -1.108449 | -3.417358 |
| N | -3.702183 | -1.068976 | -0.377154 |
| O | -4.875280 | -1.543890 | 1.491123 |
| S | -1.691983 | -4.077248 | 1.242054 |
| C | -5.923573 | -0.144175 | -1.119486 |
| C | -7.294552 | -0.419439 | -1.199086 |
| C | -5.516293 | 1.194055 | -1.058240 |
| C | -8.233843 | 0.611104 | -1.226005 |
| H | -7.628987 | -1.454148 | -1.228626 |
| C | -6.453079 | 2.229221 | -1.081874 |
| C | -7.815695 | 1.942014 | -1.169016 |
| H | -9.293237 | 0.373986 | -1.280949 |
| O | 0.658261 | 0.870055 | -0.188381 |
| O | 2.471682 | -0.364782 | 2.057174 |
| C | 1.712276 | 1.137414 | -0.765240 |
| C | 2.997839 | 1.441010 | -0.087084 |
| O | 1.827829 | 1.235124 | -2.093202 |
| C | 0.617932 | 1.052057 | -2.852731 |
| H | 0.204011 | 0.056752 | -2.676046 |
| H | -0.116327 | 1.812981 | -2.577384 |
| H | 0.916812 | 1.163090 | -3.894685 |
| S | 2.818103 | 1.026862 | 1.644692 |
| C | 1.676104 | 2.219839 | 2.373201 |
| H | 2.046910 | 3.219440 | 2.121170 |
| H | 0.697826 | 2.035705 | 1.933518 |
| H | 1.672200 | 2.030424 | 3.448896 |
| C | 4.400293 | 1.511927 | 2.357511 |
| H | 5.168897 | 0.869239 | 1.927115 |
| H | 4.575965 | 2.563693 | 2.113238 |
| H | 4.318699 | 1.354319 | 3.434979 |
| C | 4.272479 | 0.882410 | -0.684987 |
| C | 4.492517 | -0.504215 | -0.734516 |
| C | 5.254484 | 1.745006 | -1.194655 |
| C | 5.679454 | -1.021012 | -1.251960 |
| H | 3.731146 | -1.185722 | -0.369938 |
| C | 6.432132 | 1.224016 | -1.734173 |
| H | 5.088653 | 2.817581 | -1.181122 |
| C | 6.652574 | -0.154466 | -1.754621 |
| H | 5.835414 | -2.095844 | -1.251490 |
| H | 7.181705 | 1.901450 | -2.133938 |
| H | 7.577087 | -0.552538 | -2.163995 |
| C | 1.228489 | 4.667482 | -1.508934 |
| C | -0.063281 | 5.058774 | -1.864408 |
| C | -0.853572 | 5.788423 | -0.973409 |

| | | | |
|---|---|---|---|
| C | -0.335790 | 6.126062 | 0.278282 |
| C | 0.952556 | 5.730160 | 0.641113 |
| C | 1.754181 | 4.990714 | -0.245211 |
| H | 1.845850 | 4.118928 | -2.214195 |
| H | -0.447872 | 4.806159 | -2.850152 |
| H | -1.856490 | 6.097663 | -1.254690 |
| H | -0.936363 | 6.701949 | 0.978386 |
| H | 1.351341 | 6.002625 | 1.614530 |
| S | 3.411874 | 4.501710 | 0.215215 |
| H | 3.159733 | 2.841876 | -0.001575 |
| H | -4.459297 | 1.430278 | -0.975222 |
| H | -6.116113 | 3.261508 | -1.028139 |
| H | -8.545731 | 2.746938 | -1.183806 |
| C | -2.554341 | 0.184630 | 2.729746 |
| C | 1.433396 | -3.633163 | 0.287613 |
| C | 0.982491 | -4.653022 | -0.563545 |
| C | 2.808991 | -3.510560 | 0.556511 |
| C | 1.919831 | -5.514078 | -1.116809 |
| H | -0.066338 | -4.787404 | -0.782837 |
| C | 3.694579 | -4.386256 | -0.053514 |
| H | 3.181326 | -2.751671 | 1.235314 |
| C | 3.289912 | -5.411243 | -0.896856 |
| H | 3.995924 | -6.091960 | -1.355636 |
| F | 1.480847 | -6.490196 | -1.935511 |
| F | 5.019166 | -4.221905 | 0.178004 |
| C | -1.393609 | 0.135853 | 3.747454 |
| H | -0.411601 | 0.047899 | 3.271428 |
| H | -1.395664 | 1.047865 | 4.356732 |
| H | -1.505073 | -0.718173 | 4.425550 |
| C | -3.866061 | 0.346479 | 3.525769 |
| H | -4.738107 | 0.416939 | 2.873976 |
| H | -4.028595 | -0.502378 | 4.198416 |
| H | -3.808389 | 1.257696 | 4.133960 |
| C | -2.392892 | 1.401893 | 1.795939 |
| H | -1.483089 | 1.362399 | 1.184706 |
| H | -3.246362 | 1.490685 | 1.115675 |
| H | -2.348967 | 2.324862 | 2.386690 |

TS Conformation 109

B3LYP/6-31G(d) Energy = -3411.863778
M06-2X/def2tzvpp/IEFPCM(chloroform) Energy = -3411.884056
M06-2X/def2tzvpp/IEFPCM(chloroform)//B3LYP/6-31G(d) Quasiharmonic Free Energy = -3411.157895

Frequencies (Top 3 out of 291)

1. -992.0002 cm-1
2. 9.5848 cm-1
3. 17.2400 cm-1

B3LYP/6-31G(d) Molecular Geometry in Cartesian Coordinates

| | | | |
|---|---|---|---|
| C | -4.542879 | -1.485319 | 0.028149 |
| C | -3.600038 | -1.297864 | -1.187555 |
| H | -4.262578 | -0.818917 | -1.911488 |
| C | -2.571555 | 0.958194 | -1.329570 |
| N | -1.326067 | 1.562701 | -1.415716 |
| H | -0.528812 | 0.951974 | -1.577233 |
| N | -2.489541 | -0.362588 | -1.007191 |
| H | -1.577562 | -0.738262 | -0.757038 |
| C | -2.778338 | -1.279601 | 1.909143 |

| | | | |
|---|---|---|---|
| C | -5.131235 | -1.739495 | 2.363298 |
| C | -2.901181 | -2.199497 | 3.149620 |
| H | -2.030054 | -1.680244 | 1.223940 |
| C | -4.317182 | -1.894771 | 3.656798 |
| H | -5.625543 | -2.669329 | 2.063795 |
| H | -5.909472 | -0.972405 | 2.428245 |
| H | -2.120396 | -2.012899 | 3.891213 |
| H | -2.823165 | -3.244621 | 2.826776 |
| H | -4.316653 | -0.960769 | 4.229400 |
| H | -4.720049 | -2.681129 | 4.301977 |
| N | -4.128941 | -1.383331 | 1.331720 |
| O | -5.717506 | -1.760128 | -0.213165 |
| S | -4.019429 | 1.754323 | -1.629942 |
| C | -2.376785 | 0.141122 | 2.311636 |
| C | -1.107796 | 0.349703 | 2.876611 |
| C | -3.238148 | 1.234453 | 2.183496 |
| C | -0.717666 | 1.616259 | 3.313781 |
| H | -0.427383 | -0.492546 | 2.981110 |
| C | -2.848895 | 2.504473 | 2.621201 |
| C | -1.590735 | 2.701483 | 3.189462 |
| H | 0.262921 | 1.752504 | 3.765459 |
| O | 4.465715 | -0.589391 | -1.923810 |
| O | 1.687227 | 0.739093 | -1.706942 |
| C | 4.331635 | -0.861777 | -0.738064 |
| C | 3.434254 | -0.140582 | 0.208893 |
| O | 4.946816 | -1.880826 | -0.124467 |
| C | 5.731331 | -2.740804 | -0.974008 |
| H | 6.484428 | -2.163449 | -1.515746 |
| H | 6.201579 | -3.455890 | -0.299447 |
| H | 5.078036 | -3.253641 | -1.683685 |
| S | 2.653993 | 1.196906 | -0.680638 |
| C | 1.833657 | 2.183828 | 0.586393 |
| H | 1.086514 | 1.548908 | 1.068881 |
| H | 2.565787 | 2.542560 | 1.310207 |
| H | 1.342208 | 3.005195 | 0.061703 |
| C | 3.813559 | 2.373442 | -1.441374 |
| H | 4.469548 | 2.776997 | -0.666914 |
| H | 3.216835 | 3.159078 | -1.913318 |
| H | 4.371193 | 1.796172 | -2.179073 |
| C | 3.995036 | 0.233559 | 1.559517 |
| C | 5.240593 | 0.877060 | 1.670421 |
| C | 3.286725 | -0.066624 | 2.733044 |
| C | 5.755149 | 1.228704 | 2.917416 |
| H | 5.820721 | 1.085216 | 0.773795 |
| C | 3.803255 | 0.284723 | 3.981467 |
| H | 2.347633 | -0.606513 | 2.663784 |
| C | 5.032676 | 0.938216 | 4.077166 |
| H | 6.720722 | 1.722457 | 2.984088 |
| H | 3.246516 | 0.037477 | 4.881170 |
| H | 5.432511 | 1.210070 | 5.050138 |
| C | 1.506516 | -4.568308 | 0.067627 |
| C | 1.901894 | -5.625596 | -0.752364 |
| C | 2.273990 | -5.392326 | -2.078274 |
| C | 2.238857 | -4.088497 | -2.578071 |
| C | 1.830785 | -3.028041 | -1.765299 |
| C | 1.465093 | -3.254004 | -0.426620 |
| H | 1.228418 | -4.752896 | 1.101059 |
| H | 1.923000 | -6.635943 | -0.350615 |
| H | 2.581844 | -6.217008 | -2.715675 |
| H | 2.515913 | -3.892275 | -3.611290 |
| H | 1.794050 | -2.020693 | -2.169134 |
| S | 0.932139 | -1.914137 | 0.633006 |

| | | | |
|---|---|---|---|
| H | 2.281059 | -0.954921 | 0.396594 |
| H | -4.211891 | 1.095381 | 1.725678 |
| H | -3.537111 | 3.339475 | 2.517845 |
| H | -1.294027 | 3.687790 | 3.536678 |
| C | -3.143133 | -2.663123 | -1.829082 |
| C | -1.031510 | 2.931615 | -1.545604 |
| C | -1.707918 | 3.924335 | -0.820315 |
| C | 0.057869 | 3.289262 | -2.359387 |
| C | -1.274409 | 5.237576 | -0.930829 |
| H | -2.544292 | 3.682631 | -0.180929 |
| C | 0.468237 | 4.614328 | -2.391325 |
| H | 0.581858 | 2.547573 | -2.948417 |
| C | -0.176298 | 5.626922 | -1.695491 |
| H | 0.149461 | 6.658495 | -1.744092 |
| F | -1.922229 | 6.186743 | -0.226608 |
| F | 1.570391 | 4.919948 | -3.122146 |
| C | -2.416544 | -3.577133 | -0.824487 |
| H | -3.067428 | -3.843845 | 0.016533 |
| H | -2.117038 | -4.509297 | -1.317135 |
| H | -1.502895 | -3.123215 | -0.426010 |
| C | -2.212143 | -2.367363 | -3.024304 |
| H | -2.674438 | -1.659467 | -3.722466 |
| H | -2.010159 | -3.295859 | -3.570395 |
| H | -1.244149 | -1.960152 | -2.714687 |
| C | -4.386554 | -3.403343 | -2.367168 |
| H | -4.073512 | -4.343133 | -2.837980 |
| H | -4.908198 | -2.803323 | -3.121463 |
| H | -5.103864 | -3.629593 | -1.576194 |

TS Conformation 110

B3LYP/6-31G(d) Energy = -3411.863115
M06-2X/def2tzvpp/IEFPCM(chloroform) Energy = -3411.883219
M06-2X/def2tzvpp/IEFPCM(chloroform)//B3LYP/6-31G(d) Quasiharmonic Free Energy = -3411.157786

Frequencies (Top 3 out of 291)

1. -709.5868 cm-1
2. 9.8390 cm-1
3. 14.1168 cm-1

B3LYP/6-31G(d) Molecular Geometry in Cartesian Coordinates

| | | | |
|---|---|---|---|
| C | -3.678945 | -2.809843 | -0.404716 |
| C | -3.149973 | -1.819233 | -1.471403 |
| H | -4.071695 | -1.431735 | -1.910177 |
| C | -2.983964 | 0.626701 | -0.996303 |
| N | -2.114282 | 1.553646 | -0.449392 |
| H | -1.397865 | 1.187162 | 0.171827 |
| N | -2.463183 | -0.637592 | -0.958385 |
| H | -1.473018 | -0.711485 | -0.751323 |
| C | -1.813711 | -2.501236 | 1.366521 |
| C | -3.669132 | -4.060140 | 1.670754 |
| C | -1.352512 | -3.708456 | 2.221374 |
| H | -1.094043 | -2.326223 | 0.563563 |
| C | -2.668607 | -4.216322 | 2.824379 |
| H | -4.675033 | -3.780886 | 1.998993 |
| H | -3.770587 | -4.974106 | 1.075748 |
| H | -0.603631 | -3.431881 | 2.966964 |
| H | -0.915055 | -4.468905 | 1.562616 |
| H | -2.956719 | -3.588763 | 3.674607 |

| | | | |
|---|---|---|---|
| H | -2.607006 | -5.249864 | 3.177085 |
| N | -3.087071 | -2.998477 | 0.816209 |
| O | -4.681045 | -3.462896 | -0.697534 |
| S | -4.501167 | 1.005603 | -1.611551 |
| C | -1.948815 | -1.234983 | 2.216267 |
| C | -3.184106 | -0.623909 | 2.458470 |
| C | -0.798523 | -0.693638 | 2.812275 |
| C | -3.275800 | 0.494866 | 3.290219 |
| H | -4.078067 | -1.021227 | 1.988469 |
| C | -0.893554 | 0.414615 | 3.654595 |
| C | -2.132671 | 1.012801 | 3.900060 |
| H | -4.244183 | 0.956666 | 3.462404 |
| O | 0.744765 | 1.871422 | 0.726276 |
| O | 0.746061 | -0.669728 | -0.802053 |
| C | 1.955201 | 1.805784 | 0.896884 |
| C | 2.862796 | 0.891813 | 0.142263 |
| O | 2.621010 | 2.494449 | 1.826150 |
| C | 1.825411 | 3.341485 | 2.684412 |
| H | 1.139401 | 2.732407 | 3.276431 |
| H | 2.544861 | 3.846562 | 3.327801 |
| H | 1.252423 | 4.063605 | 2.098133 |
| S | 1.887000 | 0.184568 | -1.205194 |
| C | 1.307503 | 1.472813 | -2.347148 |
| H | 2.159068 | 2.075900 | -2.667720 |
| H | 0.577324 | 2.064837 | -1.793615 |
| H | 0.833866 | 0.966700 | -3.191958 |
| C | 3.052951 | -0.767478 | -2.200784 |
| H | 2.451721 | -1.245365 | -2.977353 |
| H | 3.496431 | -1.522711 | -1.547062 |
| H | 3.812584 | -0.109253 | -2.622915 |
| C | 4.195501 | 1.433717 | -0.314588 |
| C | 5.351756 | 0.647818 | -0.195820 |
| C | 4.308057 | 2.728959 | -0.851934 |
| C | 6.585679 | 1.139907 | -0.624088 |
| H | 5.279789 | -0.334642 | 0.258756 |
| C | 5.541932 | 3.219250 | -1.275080 |
| H | 3.430254 | 3.368092 | -0.914928 |
| C | 6.683857 | 2.421170 | -1.167892 |
| H | 7.473328 | 0.522135 | -0.521167 |
| H | 5.612986 | 4.224259 | -1.681486 |
| H | 7.646555 | 2.802521 | -1.496804 |
| C | 4.861024 | -3.137638 | 0.406287 |
| C | 5.015375 | -4.129791 | -0.564680 |
| C | 3.898479 | -4.772441 | -1.104249 |
| C | 2.625399 | -4.416047 | -0.651353 |
| C | 2.469584 | -3.422672 | 0.316918 |
| C | 3.583159 | -2.751344 | 0.862666 |
| H | 5.736834 | -2.663167 | 0.840844 |
| H | 6.014529 | -4.407962 | -0.892735 |
| H | 4.018983 | -5.548306 | -1.855605 |
| H | 1.746219 | -4.917357 | -1.050136 |
| H | 1.477694 | -3.156236 | 0.667198 |
| S | 3.391525 | -1.464648 | 2.081993 |
| H | 3.062319 | -0.150500 | 0.949264 |
| H | 0.177815 | -1.139231 | 2.628504 |
| H | 0.004333 | 0.798133 | 4.132895 |
| H | -2.206607 | 1.872006 | 4.561748 |
| C | -2.368556 | -2.538093 | -2.638150 |
| C | -2.237400 | 2.958300 | -0.390955 |
| C | -1.764973 | 3.595209 | 0.766705 |
| C | -2.708179 | 3.726116 | -1.466596 |
| C | -1.750340 | 4.980029 | 0.812097 |

| | | | |
|---|---|---|---|
| H | -1.403038 | 3.021322 | 1.609842 |
| C | -2.698838 | 5.108818 | -1.344007 |
| H | -3.089794 | 3.266983 | -2.367142 |
| C | -2.217711 | 5.778624 | -0.223366 |
| H | -2.210088 | 6.859901 | -0.163866 |
| F | -1.244963 | 5.577462 | 1.917503 |
| F | -3.151399 | 5.845347 | -2.379926 |
| C | -1.871543 | -1.467456 | -3.631328 |
| H | -1.107337 | -0.821108 | -3.185221 |
| H | -1.431146 | -1.952885 | -4.510699 |
| H | -2.694264 | -0.828864 | -3.970764 |
| C | -3.333084 | -3.477835 | -3.392365 |
| H | -4.181879 | -2.921167 | -3.804752 |
| H | -2.802631 | -3.955413 | -4.225608 |
| H | -3.737157 | -4.255621 | -2.742013 |
| C | -1.169967 | -3.361984 | -2.126973 |
| H | -0.416972 | -2.742306 | -1.627635 |
| H | -1.490376 | -4.146116 | -1.431299 |
| H | -0.672713 | -3.858102 | -2.969821 |

TS Conformation 111

B3LYP/6-31G(d) Energy = -3411.870296
M06-2X/def2tzvpp/IEFPCM(chloroform) Energy = -3411.882446
M06-2X/def2tzvpp/IEFPCM(chloroform)//B3LYP/6-31G(d) Quasiharmonic Free Energy = -3411.157631

Frequencies (Top 3 out of 291)

1. -947.1538 cm-1
2. 6.1903 cm-1
3. 8.7622 cm-1

B3LYP/6-31G(d) Molecular Geometry in Cartesian Coordinates

| | | | |
|---|---|---|---|
| C | -4.429201 | -2.040109 | 0.530799 |
| C | -3.844181 | -1.418979 | -0.762441 |
| H | -4.722671 | -0.975199 | -1.234881 |
| C | -3.197330 | 0.996521 | -0.846691 |
| N | -2.165040 | 1.817881 | -0.402591 |
| H | -1.668130 | 1.459371 | 0.407289 |
| N | -2.924731 | -0.307389 | -0.551221 |
| H | -1.950131 | -0.517879 | -0.366241 |
| C | -2.356311 | -1.748589 | 2.056939 |
| C | -4.417741 | -2.811439 | 2.829369 |
| C | -2.033341 | -2.798219 | 3.151119 |
| H | -1.706401 | -1.914879 | 1.195309 |
| C | -3.351371 | -2.896179 | 3.929789 |
| H | -5.327811 | -2.289888 | 3.141019 |
| H | -4.725452 | -3.798249 | 2.466569 |
| H | -1.801822 | -3.756419 | 2.669759 |
| H | -1.177931 | -2.508190 | 3.766339 |
| H | -3.435792 | -3.817549 | 4.513079 |
| H | -3.441221 | -2.052649 | 4.622779 |
| N | -3.752281 | -2.082179 | 1.723949 |
| O | -5.557681 | -2.524958 | 0.460239 |
| S | -4.589970 | 1.545461 | -1.598881 |
| C | -2.150901 | -0.325009 | 2.578149 |
| C | -0.840630 | 0.128680 | 2.803529 |
| C | -3.218560 | 0.522611 | 2.895219 |
| C | -0.607070 | 1.389220 | 3.356209 |
| H | 0.007579 | -0.506920 | 2.560059 |

| | | | |
|---|---|---|---|
| C | -2.986320 | 1.786481 | 3.444259 |
| C | -1.681900 | 2.221411 | 3.685509 |
| H | 0.415670 | 1.708710 | 3.538809 |
| O | 4.042970 | 0.858029 | -2.083971 |
| O | 0.209789 | -0.421000 | -0.482331 |
| C | 3.978129 | -0.311921 | -1.713471 |
| C | 2.816319 | -0.917971 | -1.019291 |
| O | 4.988119 | -1.183801 | -1.841361 |
| C | 6.220859 | -0.642472 | -2.356131 |
| H | 6.651430 | 0.050098 | -1.628811 |
| H | 6.049180 | -0.124872 | -3.302371 |
| H | 6.872889 | -1.504262 | -2.497641 |
| S | 1.408490 | 0.206980 | -1.095971 |
| C | 1.852010 | 1.727219 | -0.236241 |
| H | 2.704660 | 2.169239 | -0.750861 |
| H | 0.968900 | 2.371310 | -0.250301 |
| H | 2.118750 | 1.413409 | 0.779609 |
| C | 1.023960 | 0.730670 | -2.787051 |
| H | 0.201110 | 1.448020 | -2.726241 |
| H | 1.924630 | 1.171149 | -3.216521 |
| H | 0.713200 | -0.167040 | -3.325821 |
| C | 2.410729 | -2.327941 | -1.358791 |
| C | 2.446939 | -2.794421 | -2.684631 |
| C | 1.984069 | -3.200651 | -0.346941 |
| C | 2.072868 | -4.101001 | -2.990351 |
| H | 2.793339 | -2.137451 | -3.478871 |
| C | 1.608038 | -4.508690 | -0.655191 |
| H | 1.975189 | -2.860801 | 0.682869 |
| C | 1.649078 | -4.961360 | -1.974431 |
| H | 2.114088 | -4.448001 | -4.019141 |
| H | 1.289418 | -5.176550 | 0.140239 |
| H | 1.359418 | -5.981580 | -2.210931 |
| C | 5.945619 | -0.638432 | 1.433639 |
| C | 7.221890 | -0.072712 | 1.450569 |
| C | 7.447990 | 1.150588 | 2.085859 |
| C | 6.380370 | 1.802608 | 2.705859 |
| C | 5.101510 | 1.245119 | 2.683459 |
| C | 4.860910 | 0.016009 | 2.045069 |
| H | 5.782589 | -1.598982 | 0.954049 |
| H | 8.047789 | -0.601192 | 0.978999 |
| H | 8.443660 | 1.585588 | 2.103849 |
| H | 6.541460 | 2.752988 | 3.209439 |
| H | 4.276280 | 1.758639 | 3.169039 |
| S | 3.225239 | -0.701851 | 2.042709 |
| H | 3.057959 | -0.852331 | 0.352929 |
| H | -4.235350 | 0.194901 | 2.702629 |
| H | -3.828640 | 2.429661 | 3.683829 |
| H | -1.503289 | 3.198720 | 4.126169 |
| C | -3.284911 | -2.493049 | -1.770721 |
| C | -1.890280 | 3.159521 | -0.709951 |
| C | -2.150419 | 3.719011 | -1.971331 |
| C | -1.236049 | 3.925970 | 0.271479 |
| C | -1.767899 | 5.033581 | -2.201511 |
| H | -2.666129 | 3.164741 | -2.742481 |
| C | -0.869579 | 5.232000 | -0.028201 |
| H | -1.056119 | 3.530620 | 1.265689 |
| C | -1.118599 | 5.824680 | -1.259011 |
| H | -0.833028 | 6.847450 | -1.470661 |
| F | -2.020279 | 5.565981 | -3.412711 |
| F | -0.249339 | 5.956120 | 0.924349 |
| C | -2.795811 | -1.762799 | -3.038551 |
| H | -1.932901 | -1.120759 | -2.830911 |

| | | | |
|---|---|---|---|
| H | -2.492271 | -2.496299 | -3.794871 |
| H | -3.585601 | -1.136329 | -3.466571 |
| C | -4.419511 | -3.456739 | -2.175431 |
| H | -4.810002 | -4.015169 | -1.322281 |
| H | -4.042922 | -4.171389 | -2.917451 |
| H | -5.259001 | -2.913288 | -2.622491 |
| C | -2.121951 | -3.308679 | -1.173481 |
| H | -2.431772 | -3.855409 | -0.274971 |
| H | -1.256221 | -2.685340 | -0.921551 |
| H | -1.771912 | -4.049669 | -1.901571 |

TS Conformation 112

B3LYP/6-31G(d) Energy = -3411.870846
M06-2X/def2tzvpp/IEFPCM(chloroform) Energy = -3411.883142
M06-2X/def2tzvpp/IEFPCM(chloroform)//B3LYP/6-31G(d) Quasiharmonic Free Energy = -3411.157446

Frequencies (Top 3 out of 291)

1. -924.3232 cm-1
2. 9.6136 cm-1
3. 12.4172 cm-1

B3LYP/6-31G(d) Molecular Geometry in Cartesian Coordinates

| | | | |
|---|---|---|---|
| C | -5.057877 | -0.667354 | -0.070676 |
| C | -4.133577 | -0.192353 | -1.220001 |
| H | -4.718679 | 0.608075 | -1.677866 |
| C | -2.625217 | 1.778660 | -0.892311 |
| N | -1.359233 | 2.065647 | -0.407638 |
| H | -0.991207 | 1.373438 | 0.238525 |
| N | -2.882865 | 0.436140 | -0.810026 |
| H | -2.074271 | -0.161410 | -0.678956 |
| C | -3.220176 | -1.310114 | 1.632565 |
| C | -5.603919 | -1.603844 | 2.098238 |
| C | -3.426082 | -2.471136 | 2.639433 |
| H | -2.578001 | -1.650216 | 0.816670 |
| C | -4.780401 | -2.137312 | 3.278085 |
| H | -6.306958 | -0.813100 | 2.376715 |
| H | -6.188185 | -2.389196 | 1.605333 |
| H | -2.606425 | -2.543407 | 3.359060 |
| H | -3.486514 | -3.418171 | 2.088646 |
| H | -4.653448 | -1.362797 | 4.042061 |
| H | -5.254352 | -3.001069 | 3.753245 |
| N | -4.592705 | -1.088477 | 1.147395 |
| O | -6.268623 | -0.676490 | -0.291251 |
| S | -3.719344 | 2.909873 | -1.474326 |
| C | -2.579506 | -0.095755 | 2.302300 |
| C | -1.207625 | -0.135203 | 2.595801 |
| C | -3.314093 | 1.032779 | 2.680785 |
| C | -0.586077 | 0.918585 | 3.268805 |
| H | -0.619062 | -0.999715 | 2.295291 |
| C | -2.694890 | 2.090882 | 3.351101 |
| C | -1.333786 | 2.035734 | 3.652411 |
| H | 0.478850 | 0.865479 | 3.478134 |
| O | 4.020247 | -1.257754 | -2.432561 |
| O | -0.004590 | -0.965147 | -0.827922 |
| C | 3.788886 | -1.954093 | -1.446718 |
| C | 2.531946 | -1.916715 | -0.660000 |
| O | 4.677606 | -2.805303 | -0.917098 |
| C | 5.990887 | -2.788736 | -1.511879 |

| | | | |
|---|---|---|---|
| H | 5.927395 | -2.948979 | -2.590370 |
| H | 6.475139 | -1.830531 | -1.308527 |
| H | 6.531747 | -3.602707 | -1.029550 |
| S | 1.295475 | -0.947509 | -1.548112 |
| C | 1.056598 | -1.546664 | -3.241077 |
| H | 0.662307 | -2.561392 | -3.154888 |
| H | 0.324580 | -0.893400 | -3.722052 |
| H | 2.023360 | -1.528186 | -3.745399 |
| C | 1.903218 | 0.738971 | -1.732673 |
| H | 2.129885 | 1.079309 | -0.716200 |
| H | 2.797650 | 0.715167 | -2.353886 |
| H | 1.086811 | 1.317393 | -2.170967 |
| C | 1.945177 | -3.206456 | -0.149386 |
| C | 1.384774 | -3.264826 | 1.134998 |
| C | 1.938286 | -4.366010 | -0.944161 |
| C | 0.830150 | -4.453740 | 1.610360 |
| H | 1.417403 | -2.386807 | 1.770969 |
| C | 1.385701 | -5.552906 | -0.467584 |
| H | 2.390249 | -4.345478 | -1.933114 |
| C | 0.825178 | -5.597444 | 0.811192 |
| H | 0.408791 | -4.486442 | 2.611240 |
| H | 1.395046 | -6.442166 | -1.091806 |
| H | 0.394581 | -6.522610 | 1.184420 |
| C | 5.592144 | -0.378634 | 1.400460 |
| C | 6.908438 | 0.034085 | 1.185073 |
| C | 7.206276 | 1.386498 | 1.003035 |
| C | 6.170923 | 2.322548 | 1.040674 |
| C | 4.851887 | 1.915773 | 1.246630 |
| C | 4.541565 | 0.556190 | 1.426587 |
| H | 5.370408 | -1.429489 | 1.562091 |
| H | 7.707309 | -0.704658 | 1.178118 |
| H | 8.232251 | 1.706908 | 0.842484 |
| H | 6.388515 | 3.379772 | 0.907750 |
| H | 4.055307 | 2.653387 | 1.276917 |
| S | 2.853269 | 0.043526 | 1.708996 |
| H | 2.731095 | -1.072367 | 0.417742 |
| H | -4.369528 | 1.093276 | 2.434008 |
| H | -3.280181 | 2.962691 | 3.630543 |
| H | -0.854290 | 2.860111 | 4.172827 |
| C | -3.919453 | -1.288781 | -2.331184 |
| C | -0.582281 | 3.236378 | -0.473141 |
| C | -0.749204 | 4.231838 | -1.448484 |
| C | 0.475452 | 3.337470 | 0.450777 |
| C | 0.131338 | 5.305833 | -1.452907 |
| H | -1.553361 | 4.193231 | -2.167674 |
| C | 1.323480 | 4.431993 | 0.382185 |
| H | 0.641335 | 2.583359 | 1.212743 |
| C | 1.185364 | 5.445508 | -0.557870 |
| H | 1.855550 | 6.295384 | -0.588443 |
| F | -0.037075 | 6.258885 | -2.391580 |
| F | 2.338756 | 4.514253 | 1.270668 |
| C | -3.075674 | -0.670622 | -3.465394 |
| H | -2.068955 | -0.404223 | -3.125205 |
| H | -2.977302 | -1.386802 | -4.290218 |
| H | -3.544710 | 0.237941 | -3.857927 |
| C | -5.282409 | -1.705028 | -2.922113 |
| H | -5.818587 | -0.839989 | -3.326517 |
| H | -5.930270 | -2.169062 | -2.176133 |
| H | -5.122142 | -2.419662 | -3.738932 |
| C | -3.208832 | -2.544009 | -1.785783 |
| H | -3.098886 | -3.287332 | -2.584972 |
| H | -3.786340 | -3.014989 | -0.981992 |

| | | | |
|---|---|---|---|
| H | -2.202457 | -2.328584 | -1.407016 |

TS Conformation 113

B3LYP/6-31G(d) Energy = -3411.868305
M06-2X/def2tzvpp/IEFPCM(chloroform) Energy = -3411.882464
M06-2X/def2tzvpp/IEFPCM(chloroform)//B3LYP/6-31G(d) Quasiharmonic Free Energy = -3411.157376

Frequencies (Top 3 out of 291)

1. -934.7423 cm-1
2. 8.5875 cm-1
3. 9.8080 cm-1

B3LYP/6-31G(d) Molecular Geometry in Cartesian Coordinates

| | | | |
|---|---|---|---|
| C | -2.100865 | 3.808831 | 0.917005 |
| C | -2.310489 | 2.516343 | 1.745658 |
| H | -3.354234 | 2.594234 | 2.056004 |
| C | -3.290494 | 0.455475 | 0.717796 |
| N | -2.902459 | -0.565589 | -0.145623 |
| H | -2.149831 | -0.310297 | -0.777866 |
| N | -2.233344 | 1.275997 | 0.983411 |
| H | -1.318141 | 0.878293 | 0.804272 |
| C | -0.368219 | 2.890962 | -0.776477 |
| C | -1.121744 | 5.215392 | -0.799317 |
| C | 0.757827 | 3.806412 | -1.320873 |
| H | 0.041391 | 2.242243 | 0.000428 |
| C | -0.016938 | 5.021181 | -1.847268 |
| H | -2.065872 | 5.574769 | -1.219952 |
| H | -0.834686 | 5.922273 | -0.013198 |
| H | 1.375058 | 3.309765 | -2.073646 |
| H | 1.408443 | 4.101681 | -0.488392 |
| H | -0.448670 | 4.795424 | -2.828443 |
| H | 0.607492 | 5.912738 | -1.954779 |
| N | -1.298269 | 3.874034 | -0.194013 |
| O | -2.684175 | 4.822169 | 1.300150 |
| S | -4.845373 | 0.660822 | 1.306070 |
| C | -0.973584 | 2.030481 | -1.887426 |
| C | -0.207091 | 0.982278 | -2.423614 |
| C | -2.239763 | 2.280307 | -2.428985 |
| C | -0.686869 | 0.220070 | -3.490730 |
| H | 0.777648 | 0.763353 | -2.017940 |
| C | -2.723119 | 1.515935 | -3.494559 |
| C | -1.946769 | 0.489674 | -4.035442 |
| H | -0.066411 | -0.573677 | -3.899443 |
| O | 2.315379 | -4.405409 | 0.868511 |
| O | 0.286633 | -0.561717 | 0.629125 |
| C | 3.023795 | -3.412549 | 1.019115 |
| C | 2.569587 | -2.011097 | 0.843486 |
| O | 4.336297 | -3.473338 | 1.273928 |
| C | 4.909119 | -4.795324 | 1.302933 |
| H | 4.430304 | -5.404773 | 2.073281 |
| H | 4.789794 | -5.279351 | 0.330933 |
| H | 5.963859 | -4.643438 | 1.530015 |
| S | 0.770925 | -1.963773 | 0.696948 |
| C | -0.062220 | -2.823421 | 2.057177 |
| H | -1.136445 | -2.779762 | 1.858547 |
| H | 0.183461 | -2.270652 | 2.966582 |
| H | 0.311744 | -3.847305 | 2.091295 |
| C | 0.297542 | -2.883461 | -0.778988 |

| | | | |
|---|---|---|---|
| H | 0.651099 | -3.908167 | -0.666093 |
| H | -0.790788 | -2.821165 | -0.860856 |
| H | 0.795986 | -2.377935 | -1.613015 |
| C | 3.060016 | -0.946963 | 1.785790 |
| C | 3.300907 | 0.350668 | 1.311421 |
| C | 3.274253 | -1.221400 | 3.147955 |
| C | 3.755500 | 1.346232 | 2.176902 |
| H | 3.152700 | 0.572631 | 0.260976 |
| C | 3.730509 | -0.226866 | 4.010242 |
| H | 3.106415 | -2.225179 | 3.530275 |
| C | 3.970140 | 1.061546 | 3.525962 |
| H | 3.951000 | 2.342317 | 1.790413 |
| H | 3.900020 | -0.457373 | 5.058403 |
| H | 4.328654 | 1.837072 | 4.197279 |
| C | 5.302824 | 0.283314 | -1.583177 |
| C | 5.979881 | 1.491611 | -1.750507 |
| C | 5.513572 | 2.447662 | -2.655983 |
| C | 4.358652 | 2.179304 | -3.393613 |
| C | 3.671515 | 0.976375 | -3.220721 |
| C | 4.129859 | 0.007334 | -2.309465 |
| H | 5.682671 | -0.460795 | -0.889129 |
| H | 6.883753 | 1.681419 | -1.176031 |
| H | 6.047335 | 3.384676 | -2.790466 |
| H | 3.988574 | 2.908228 | -4.111155 |
| H | 2.776222 | 0.774130 | -3.801336 |
| S | 3.266672 | -1.544838 | -2.135479 |
| H | 2.943996 | -1.705089 | -0.455064 |
| H | -2.853726 | 3.070543 | -2.008401 |
| H | -3.708736 | 1.726029 | -3.900797 |
| H | -2.318120 | -0.094819 | -4.873100 |
| C | -1.449481 | 2.479996 | 3.065698 |
| C | -3.561196 | -1.760902 | -0.472592 |
| C | -3.336652 | -2.293174 | -1.755120 |
| C | -4.338097 | -2.476890 | 0.452254 |
| C | -3.902215 | -3.518902 | -2.083513 |
| H | -2.771514 | -1.743668 | -2.500665 |
| C | -4.883559 | -3.690306 | 0.056085 |
| H | -4.543011 | -2.090635 | 1.440526 |
| C | -4.685128 | -4.251763 | -1.201485 |
| H | -5.124965 | -5.201431 | -1.479118 |
| F | -3.683641 | -4.013265 | -3.318098 |
| F | -5.628513 | -4.373384 | 0.946539 |
| C | -1.778603 | 1.179714 | 3.827699 |
| H | -2.854728 | 1.085163 | 4.007410 |
| H | -1.267671 | 1.179691 | 4.797878 |
| H | -1.450426 | 0.291033 | 3.277619 |
| C | -1.833517 | 3.676224 | 3.960712 |
| H | -2.901562 | 3.660594 | 4.203816 |
| H | -1.622092 | 4.633968 | 3.480988 |
| H | -1.270141 | 3.624097 | 4.900438 |
| C | 0.063676 | 2.531579 | 2.779250 |
| H | 0.623522 | 2.518315 | 3.721776 |
| H | 0.343251 | 3.448418 | 2.246921 |
| H | 0.411200 | 1.670831 | 2.196365 |

TS Conformation 114

B3LYP/6-31G(d) Energy = -3411.868581
M06-2X/def2tzvpp/IEFPCM(chloroform) Energy = -3411.882212
M06-2X/def2tzvpp/IEFPCM(chloroform)//B3LYP/6-31G(d) Quasiharmonic Free Energy = -3411.157229

Frequencies (Top 3 out of 291)

1. -911.5204 cm-1
2. 10.2268 cm-1
3. 11.1882 cm-1

B3LYP/6-31G(d) Molecular Geometry in Cartesian Coordinates

| | | | |
|---|---|---|---|
| C | -4.360460 | -1.805173 | -0.747660 |
| C | -3.561601 | -0.846052 | -1.667030 |
| H | -4.341761 | -0.180453 | -2.042120 |
| C | -2.797942 | 1.356338 | -0.747800 |
| N | -1.706402 | 1.898599 | -0.091590 |
| H | -1.088332 | 1.212189 | 0.330400 |
| N | -2.601441 | 0.024508 | -0.997600 |
| H | -1.654431 | -0.318971 | -0.882210 |
| C | -2.516920 | -2.241362 | 1.014050 |
| C | -4.717169 | -3.306243 | 1.123460 |
| C | -2.415449 | -3.597462 | 1.758570 |
| H | -1.752950 | -2.199351 | 0.234080 |
| C | -3.842729 | -3.813342 | 2.277860 |
| H | -5.646490 | -2.831883 | 1.452590 |
| H | -4.997029 | -4.104983 | 0.427310 |
| H | -1.656259 | -3.581671 | 2.544590 |
| H | -2.150109 | -4.382442 | 1.039200 |
| H | -4.008269 | -3.214582 | 3.180030 |
| H | -4.054109 | -4.857332 | 2.526050 |
| N | -3.858990 | -2.329242 | 0.414550 |
| O | -5.494840 | -2.112333 | -1.111270 |
| S | -4.193662 | 2.187478 | -1.168650 |
| C | -2.324290 | -1.062422 | 1.966360 |
| C | -1.028661 | -0.763191 | 2.416110 |
| C | -3.394781 | -0.298862 | 2.443160 |
| C | -0.814271 | 0.265259 | 3.335540 |
| H | -0.181600 | -1.338871 | 2.047650 |
| C | -3.180441 | 0.733068 | 3.360190 |
| C | -1.892131 | 1.015509 | 3.813620 |
| H | 0.196129 | 0.479860 | 3.672410 |
| O | 4.194568 | 1.308861 | -2.853250 |
| O | 0.647389 | -0.151900 | -0.799610 |
| C | 4.359049 | 0.963951 | -1.685090 |
| C | 3.336919 | 0.281031 | -0.851520 |
| O | 5.523809 | 1.077422 | -1.037980 |
| C | 6.626388 | 1.586212 | -1.813450 |
| H | 6.403578 | 2.589552 | -2.184800 |
| H | 7.471478 | 1.607133 | -1.126140 |
| H | 6.832009 | 0.924133 | -2.657670 |
| S | 1.728759 | 0.399930 | -1.661730 |
| C | 1.825389 | -0.504510 | -3.218260 |
| H | 0.810219 | -0.542870 | -3.619310 |
| H | 2.207110 | -1.501070 | -2.959390 |
| H | 2.521159 | 0.019441 | -3.873040 |
| C | 1.314548 | 2.099500 | -2.138330 |
| H | 2.128298 | 2.482670 | -2.754490 |
| H | 0.365778 | 2.066440 | -2.679590 |
| H | 1.198538 | 2.666620 | -1.211770 |
| C | 3.252119 | 0.605581 | 0.615330 |
| C | 3.090029 | -0.422969 | 1.554380 |
| C | 3.345338 | 1.932241 | 1.070850 |
| C | 3.044119 | -0.129819 | 2.918600 |
| H | 3.020310 | -1.451859 | 1.221030 |
| C | 3.286908 | 2.225841 | 2.431740 |

| | | | |
|---|---|---|---|
| H | 3.484688 | 2.741041 | 0.357520 |
| C | 3.140618 | 1.191161 | 3.359830 |
| H | 2.939569 | -0.940189 | 3.634640 |
| H | 3.344468 | 3.257651 | 2.762790 |
| H | 3.107628 | 1.415961 | 4.422640 |
| C | 2.032751 | -3.922250 | 0.013370 |
| C | 1.541141 | -4.680210 | 1.077840 |
| C | 2.405811 | -5.161349 | 2.063170 |
| C | 3.770121 | -4.876279 | 1.970380 |
| C | 4.263631 | -4.116319 | 0.909580 |
| C | 3.403851 | -3.622539 | -0.089270 |
| H | 1.355071 | -3.565600 | -0.757320 |
| H | 0.478331 | -4.906110 | 1.129850 |
| H | 2.022952 | -5.756180 | 2.888270 |
| H | 4.456571 | -5.248859 | 2.727220 |
| H | 5.325231 | -3.898338 | 0.840570 |
| S | 4.059170 | -2.668289 | -1.448630 |
| H | 3.623580 | -1.056739 | -1.024990 |
| H | -4.398361 | -0.498663 | 2.081070 |
| H | -4.024122 | 1.319758 | 3.713040 |
| H | -1.726162 | 1.818659 | 4.526290 |
| C | -2.936490 | -1.565232 | -2.920360 |
| C | -1.350492 | 3.219529 | 0.222020 |
| C | -1.918733 | 4.360669 | -0.364610 |
| C | -0.289523 | 3.367309 | 1.140110 |
| C | -1.421994 | 5.606329 | -0.001820 |
| H | -2.738213 | 4.288858 | -1.063550 |
| C | 0.160617 | 4.641130 | 1.451940 |
| H | 0.159398 | 2.509030 | 1.627550 |
| C | -0.380724 | 5.794969 | 0.899560 |
| H | -0.019814 | 6.781709 | 1.161100 |
| F | -1.974484 | 6.695899 | -0.569100 |
| F | 1.175357 | 4.765640 | 2.336860 |
| C | -1.848810 | -2.585471 | -2.527720 |
| H | -1.000950 | -2.122491 | -2.006900 |
| H | -1.446230 | -3.069301 | -3.425560 |
| H | -2.253639 | -3.376942 | -1.886890 |
| C | -2.326331 | -0.486712 | -3.839500 |
| H | -1.511851 | 0.057079 | -3.346910 |
| H | -3.078471 | 0.251188 | -4.138790 |
| H | -1.925851 | -0.950651 | -4.748900 |
| C | -4.040220 | -2.296242 | -3.712240 |
| H | -3.608550 | -2.726652 | -4.624060 |
| H | -4.840450 | -1.608323 | -4.003930 |
| H | -4.498920 | -3.100503 | -3.133830 |

TS Conformation 115

B3LYP/6-31G(d) Energy = -3411.873665
M06-2X/def2tzvpp/IEFPCM(chloroform) Energy = -3411.882446
M06-2X/def2tzvpp/IEFPCM(chloroform)//B3LYP/6-31G(d) Quasiharmonic Free Energy = -3411.157144

Frequencies (Top 3 out of 291)

1. -810.6879 cm-1
2. 5.9045 cm-1
3. 8.6574 cm-1

B3LYP/6-31G(d) Molecular Geometry in Cartesian Coordinates

| | | | |
|---|---|---|---|
| C | 2.640947 | -1.130671 | 0.623607 |

| | | | |
|---|---|---|---|
| C | 2.987577 | 0.233330 | 1.256274 |
| H | 3.625171 | 0.825004 | 0.601080 |
| C | 1.415415 | 2.173978 | 0.862429 |
| N | 0.086648 | 2.490600 | 1.080803 |
| H | -0.474968 | 1.706400 | 1.402696 |
| N | 1.709654 | 0.943477 | 1.360247 |
| H | 0.943153 | 0.355362 | 1.677991 |
| C | 3.609780 | -0.427765 | -1.618294 |
| C | 2.614023 | -2.653153 | -1.277960 |
| C | 3.112334 | -1.018825 | -2.963619 |
| H | 3.157039 | 0.555088 | -1.458932 |
| C | 3.118000 | -2.534372 | -2.720800 |
| H | 3.014348 | -3.516979 | -0.739178 |
| H | 1.521748 | -2.710657 | -1.228817 |
| H | 3.735620 | -0.703183 | -3.804575 |
| H | 2.085943 | -0.676152 | -3.140499 |
| H | 4.135518 | -2.930624 | -2.812486 |
| H | 2.481845 | -3.081814 | -3.422175 |
| N | 3.063194 | -1.396117 | -0.640544 |
| O | 1.960472 | -1.943762 | 1.256540 |
| S | 2.537366 | 3.157844 | 0.076259 |
| C | 5.125048 | -0.271396 | -1.566180 |
| C | 5.974965 | -1.300101 | -1.141247 |
| C | 5.694773 | 0.932422 | -2.004040 |
| C | 7.361554 | -1.135170 | -1.169027 |
| H | 5.550452 | -2.227459 | -0.768197 |
| C | 7.079249 | 1.099383 | -2.032261 |
| C | 7.918394 | 0.063185 | -1.617690 |
| H | 8.005855 | -1.943968 | -0.834101 |
| O | -1.410989 | -0.000928 | 1.008893 |
| O | -0.503169 | -0.945967 | -1.696076 |
| C | -2.454055 | -0.612035 | 0.778027 |
| C | -3.044760 | -0.853070 | -0.570221 |
| O | -3.214119 | -1.171415 | 1.716483 |
| C | -2.688614 | -1.174104 | 3.059100 |
| H | -1.778422 | -1.777243 | 3.091403 |
| H | -2.480669 | -0.155044 | 3.395227 |
| H | -3.469571 | -1.628231 | 3.667368 |
| S | -1.860758 | -0.383304 | -1.845459 |
| C | -2.629739 | -0.997650 | -3.356571 |
| H | -2.691567 | -2.083824 | -3.224912 |
| H | -1.956260 | -0.739574 | -4.176617 |
| H | -3.618445 | -0.556417 | -3.489186 |
| C | -1.748723 | 1.408504 | -2.111758 |
| H | -1.130545 | 1.561391 | -2.999885 |
| H | -1.258482 | 1.822510 | -1.230154 |
| H | -2.748992 | 1.824431 | -2.240742 |
| C | -4.432632 | -0.282965 | -0.792485 |
| C | -4.724673 | 1.045465 | -0.434656 |
| C | -5.458117 | -1.078427 | -1.326790 |
| C | -6.001182 | 1.573678 | -0.617526 |
| H | -3.954202 | 1.674049 | 0.001787 |
| C | -6.735595 | -0.546279 | -1.512549 |
| H | -5.261586 | -2.122110 | -1.552717 |
| C | -7.009981 | 0.777666 | -1.164689 |
| H | -6.197909 | 2.600826 | -0.323754 |
| H | -7.521826 | -1.176159 | -1.919453 |
| H | -8.007521 | 1.183908 | -1.308043 |
| C | -1.102848 | -3.860658 | 0.627593 |
| C | -0.420072 | -4.291298 | 1.768349 |
| C | -0.955731 | -5.302295 | 2.569759 |
| C | -2.181472 | -5.874857 | 2.219171 |

| | | | |
|---|---|---|---|
| C | -2.870944 | -5.437179 | 1.088520 |
| C | -2.343435 | -4.420554 | 0.272438 |
| H | -0.657682 | -3.090212 | 0.005112 |
| H | 0.537721 | -3.836175 | 2.004037 |
| H | -0.421317 | -5.646347 | 3.451895 |
| H | -2.608395 | -6.665665 | 2.832536 |
| H | -3.829118 | -5.877463 | 0.827858 |
| S | -3.245148 | -3.874352 | -1.167621 |
| H | -3.108850 | -2.184125 | -0.797594 |
| H | 5.045997 | 1.748809 | -2.313344 |
| H | 7.501689 | 2.042677 | -2.368371 |
| H | 8.997213 | 0.192567 | -1.634846 |
| C | 3.723117 | 0.111418 | 2.640267 |
| C | -0.691070 | 3.628364 | 0.824596 |
| C | -0.218064 | 4.858139 | 0.342811 |
| C | -2.069940 | 3.477282 | 1.089134 |
| C | -1.137237 | 5.879054 | 0.129108 |
| H | 0.829267 | 5.015384 | 0.133028 |
| C | -2.932572 | 4.530310 | 0.837443 |
| H | -2.461539 | 2.553865 | 1.500580 |
| C | -2.504840 | 5.759126 | 0.351487 |
| H | -3.190790 | 6.574984 | 0.161550 |
| F | -0.674185 | 7.055169 | -0.335169 |
| F | -4.253791 | 4.339056 | 1.060705 |
| C | 4.915711 | -0.852496 | 2.485414 |
| H | 5.583345 | -0.543887 | 1.672933 |
| H | 5.502911 | -0.868733 | 3.411042 |
| H | 4.581057 | -1.876348 | 2.286831 |
| C | 2.795547 | -0.397938 | 3.760777 |
| H | 2.377352 | -1.377463 | 3.520827 |
| H | 3.362078 | -0.476195 | 4.696487 |
| H | 1.968891 | 0.299939 | 3.940257 |
| C | 4.251921 | 1.507293 | 3.025952 |
| H | 4.966916 | 1.886323 | 2.287536 |
| H | 3.440555 | 2.237912 | 3.106940 |
| H | 4.759002 | 1.458413 | 3.997271 |

TS Conformation 116

B3LYP/6-31G(d) Energy = -3411.865856
M06-2X/def2tzvpp/IEFPCM(chloroform) Energy = -3411.881057
M06-2X/def2tzvpp/IEFPCM(chloroform)//B3LYP/6-31G(d) Quasiharmonic Free Energy = -3411.157125

Frequencies (Top 3 out of 291)

1. -829.0015 cm-1
2. 4.2967 cm-1
3. 9.4325 cm-1

B3LYP/6-31G(d) Molecular Geometry in Cartesian Coordinates

| | | | |
|---|---|---|---|
| C | 4.530021 | 0.174143 | 0.688075 |
| C | 3.238087 | 0.066254 | 1.542246 |
| H | 3.467075 | 0.719841 | 2.388612 |
| C | 1.706736 | 1.891696 | 0.791007 |
| N | 0.450364 | 2.072774 | 0.253433 |
| H | -0.041950 | 1.221967 | -0.001812 |
| N | 2.014723 | 0.569064 | 0.929279 |
| H | 1.308026 | -0.109343 | 0.670990 |
| C | 5.822267 | 0.325521 | -1.362097 |
| C | 3.382531 | 0.266834 | -1.616079 |

| | | | |
|---|---|---|---|
| C | 5.411923 | 0.811716 | -2.773632 |
| H | 6.371643 | 1.102641 | -0.824281 |
| C | 4.038151 | 0.168425 | -3.000037 |
| H | 2.633979 | -0.513941 | -1.454272 |
| H | 2.896417 | 1.241055 | -1.494905 |
| H | 6.149619 | 0.540059 | -3.533091 |
| H | 5.316197 | 1.903913 | -2.766994 |
| H | 4.152206 | -0.879621 | -3.299648 |
| H | 3.445548 | 0.674222 | -3.768564 |
| N | 4.518242 | 0.130617 | -0.683630 |
| O | 5.595315 | 0.239624 | 1.297131 |
| S | 2.796083 | 3.105816 | 1.196511 |
| C | 6.694096 | -0.924181 | -1.385364 |
| C | 6.165272 | -2.208826 | -1.562509 |
| C | 8.084190 | -0.786029 | -1.286610 |
| C | 7.002204 | -3.323295 | -1.644823 |
| H | 5.088847 | -2.342330 | -1.623825 |
| C | 8.924809 | -1.895765 | -1.371123 |
| C | 8.386166 | -3.170913 | -1.552411 |
| H | 6.571071 | -4.312447 | -1.778946 |
| O | -4.047238 | -3.508252 | 0.233478 |
| O | -0.594426 | -0.921981 | -0.100081 |
| C | -4.136274 | -2.450194 | 0.853300 |
| C | -3.160024 | -1.334910 | 0.751390 |
| O | -5.159776 | -2.144012 | 1.656843 |
| C | -6.238746 | -3.101116 | 1.688686 |
| H | -5.865601 | -4.090941 | 1.960871 |
| H | -6.928658 | -2.725294 | 2.443805 |
| H | -6.721342 | -3.143389 | 0.709545 |
| S | -1.679436 | -1.942165 | -0.101995 |
| C | -1.045460 | -3.474700 | 0.627866 |
| H | -0.739911 | -3.225347 | 1.646245 |
| H | -1.847188 | -4.212937 | 0.612101 |
| H | -0.180144 | -3.784949 | 0.037584 |
| C | -2.125207 | -2.363629 | -1.796294 |
| H | -2.845909 | -3.180470 | -1.768657 |
| H | -1.195542 | -2.626983 | -2.306285 |
| H | -2.575902 | -1.448610 | -2.203924 |
| C | -2.784777 | -0.551324 | 1.978901 |
| C | -2.614952 | -1.182104 | 3.224170 |
| C | -2.575484 | 0.832285 | 1.884818 |
| C | -2.245617 | -0.445229 | 4.347159 |
| H | -2.796584 | -2.250075 | 3.319290 |
| C | -2.197640 | 1.567349 | 3.009090 |
| H | -2.730271 | 1.336960 | 0.937093 |
| C | -2.029747 | 0.931454 | 4.239378 |
| H | -2.125367 | -0.943957 | 5.304872 |
| H | -2.033688 | 2.636932 | 2.917840 |
| H | -1.735414 | 1.505436 | 5.113549 |
| C | -5.607244 | -0.957759 | -3.273736 |
| C | -6.716795 | -1.704450 | -3.672016 |
| C | -7.778719 | -1.919787 | -2.791559 |
| C | -7.720970 | -1.368814 | -1.509020 |
| C | -6.614790 | -0.616906 | -1.108781 |
| C | -5.532137 | -0.403633 | -1.982801 |
| H | -4.791140 | -0.784565 | -3.970109 |
| H | -6.751757 | -2.117353 | -4.677667 |
| H | -8.643788 | -2.498807 | -3.103502 |
| H | -8.551515 | -1.508236 | -0.819955 |
| H | -6.586769 | -0.173602 | -0.117622 |
| S | -4.123468 | 0.572828 | -1.485156 |
| H | -3.624591 | -0.479331 | -0.190514 |

| | | | |
|---|---|---|---|
| H | 8.510551 | 0.202229 | -1.129904 |
| H | 10.000462 | -1.765194 | -1.284982 |
| H | 9.038445 | -4.038035 | -1.613228 |
| C | 3.012158 | -1.379999 | 2.116553 |
| C | -0.256298 | 3.241298 | -0.099507 |
| C | -0.062628 | 4.488318 | 0.511141 |
| C | -1.248826 | 3.090115 | -1.085107 |
| C | -0.858471 | 5.550291 | 0.099441 |
| H | 0.691847 | 4.637406 | 1.268624 |
| C | -2.017227 | 4.189008 | -1.443067 |
| H | -1.425346 | 2.140506 | -1.579706 |
| C | -1.846687 | 5.444503 | -0.871072 |
| H | -2.451461 | 6.292605 | -1.166959 |
| F | -0.669355 | 6.748648 | 0.688117 |
| F | -2.956529 | 4.036289 | -2.392323 |
| C | 2.778088 | -2.410556 | 0.992077 |
| H | 3.648212 | -2.479744 | 0.331525 |
| H | 2.611347 | -3.404973 | 1.424730 |
| H | 1.902353 | -2.169166 | 0.376182 |
| C | 1.795012 | -1.354413 | 3.067647 |
| H | 1.676999 | -2.335564 | 3.544197 |
| H | 1.935514 | -0.613989 | 3.863159 |
| H | 0.855577 | -1.112661 | 2.559563 |
| C | 4.241282 | -1.820373 | 2.938445 |
| H | 5.142659 | -1.891322 | 2.327739 |
| H | 4.450058 | -1.111356 | 3.746319 |
| H | 4.043343 | -2.801374 | 3.387989 |

TS Conformation 117

B3LYP/6-31G(d) Energy = -3411.870432
M06-2X/def2tzvpp/IEFPCM(chloroform) Energy = -3411.882276
M06-2X/def2tzvpp/IEFPCM(chloroform)//B3LYP/6-31G(d) Quasiharmonic Free Energy = -3411.157113

Frequencies (Top 3 out of 291)

1. -903.1151 cm-1
2. 9.8086 cm-1
3. 9.8838 cm-1

B3LYP/6-31G(d) Molecular Geometry in Cartesian Coordinates

| | | | |
|---|---|---|---|
| C | -4.048196 | -3.002144 | -0.347016 |
| C | -3.293701 | -2.205384 | -1.441792 |
| H | -4.098686 | -1.909252 | -2.117483 |
| C | -3.149800 | 0.293777 | -1.294087 |
| N | -2.341245 | 1.262920 | -0.725019 |
| H | -1.722661 | 0.914694 | 0.000960 |
| N | -2.674640 | -0.957916 | -1.008313 |
| H | -1.727891 | -1.005120 | -0.648396 |
| C | -2.572871 | -2.340929 | 1.675264 |
| C | -4.454755 | -3.891951 | 1.872202 |
| C | -2.341647 | -3.307012 | 2.865354 |
| H | -1.682336 | -2.331323 | 1.042178 |
| C | -3.764496 | -3.745764 | 3.234918 |
| H | -5.513546 | -3.616726 | 1.885280 |
| H | -4.396773 | -4.913540 | 1.479793 |
| H | -1.753087 | -4.166143 | 2.521157 |
| H | -1.800920 | -2.829192 | 3.686302 |
| H | -4.253291 | -2.969329 | 3.833368 |
| H | -3.788851 | -4.675740 | 3.810023 |

| | | | |
|---|---|---|---|
| N | -3.695247 | -2.989393 | 0.976557 |
| O | -4.994923 | -3.697014 | -0.714392 |
| S | -4.537583 | 0.584652 | -2.191313 |
| C | -2.865922 | -0.918974 | 2.152583 |
| C | -4.156253 | -0.379180 | 2.157264 |
| C | -1.808378 | -0.139238 | 2.646717 |
| C | -4.389554 | 0.905516 | 2.654265 |
| H | -4.980488 | -0.958255 | 1.752999 |
| C | -2.041123 | 1.142646 | 3.148146 |
| C | -3.335625 | 1.668909 | 3.155559 |
| H | -5.398114 | 1.309437 | 2.641318 |
| O | 4.114261 | 1.104521 | -1.979679 |
| O | 0.390482 | -0.311827 | -0.245580 |
| C | 4.029993 | 1.189760 | -0.756655 |
| C | 2.938478 | 0.595029 | 0.055073 |
| O | 4.951386 | 1.784735 | 0.010776 |
| C | 6.136507 | 2.241481 | -0.672013 |
| H | 6.700984 | 2.794957 | 0.078174 |
| H | 6.709837 | 1.382312 | -1.028143 |
| H | 5.871323 | 2.885634 | -1.513037 |
| S | 1.570475 | 0.140818 | -1.033320 |
| C | 1.077797 | 1.486970 | -2.143262 |
| H | 0.309802 | 1.090940 | -2.812378 |
| H | 0.657060 | 2.276766 | -1.516644 |
| H | 1.964470 | 1.818327 | -2.684236 |
| C | 2.145473 | -1.167736 | -2.131804 |
| H | 1.278520 | -1.493585 | -2.710868 |
| H | 2.943922 | -0.759898 | -2.751380 |
| H | 2.524182 | -1.958317 | -1.471057 |
| C | 2.461490 | 1.321027 | 1.286934 |
| C | 2.163959 | 2.693998 | 1.252021 |
| C | 2.324887 | 0.628532 | 2.498814 |
| C | 1.739889 | 3.362482 | 2.399057 |
| H | 2.282916 | 3.251027 | 0.325638 |
| C | 1.914081 | 1.301341 | 3.651211 |
| H | 2.575647 | -0.426477 | 2.541491 |
| C | 1.618144 | 2.665004 | 3.604076 |
| H | 1.496448 | 4.418754 | 2.346172 |
| H | 1.830808 | 0.757526 | 4.588173 |
| H | 1.300366 | 3.186037 | 4.503397 |
| C | 6.388105 | -1.262820 | 0.727603 |
| C | 7.703751 | -1.227987 | 0.261193 |
| C | 8.140458 | -2.142169 | -0.700454 |
| C | 7.243768 | -3.093913 | -1.190343 |
| C | 5.926207 | -3.126449 | -0.732896 |
| C | 5.473299 | -2.210204 | 0.232904 |
| H | 6.063224 | -0.560256 | 1.489369 |
| H | 8.395733 | -0.492369 | 0.666053 |
| H | 9.166887 | -2.118204 | -1.056660 |
| H | 7.570142 | -3.816321 | -1.935012 |
| H | 5.235079 | -3.871077 | -1.117855 |
| S | 3.792165 | -2.281519 | 0.831161 |
| H | 3.355069 | -0.665195 | 0.427129 |
| H | -0.794639 | -0.534764 | 2.639666 |
| H | -1.208366 | 1.727435 | 3.529185 |
| H | -3.517860 | 2.668203 | 3.541153 |
| C | -2.301058 | -3.100102 | -2.276030 |
| C | -2.283550 | 2.657198 | -0.865555 |
| C | -2.919148 | 3.388987 | -1.880105 |
| C | -1.456314 | 3.329722 | 0.059322 |
| C | -2.718012 | 4.762952 | -1.927643 |
| H | -3.568792 | 2.912397 | -2.598536 |

| | | | |
|---|---|---|---|
| C | -1.295006 | 4.702012 | -0.051773 |
| H | -0.969339 | 2.799679 | 0.870404 |
| C | -1.910938 | 5.462355 | -1.037564 |
| H | -1.777686 | 6.534748 | -1.104576 |
| F | -3.332734 | 5.457037 | -2.904482 |
| F | -0.497158 | 5.329939 | 0.842557 |
| C | -3.064983 | -4.277592 | -2.916132 |
| H | -3.891540 | -3.921091 | -3.539699 |
| H | -2.380177 | -4.852371 | -3.551607 |
| H | -3.489080 | -4.949191 | -2.167453 |
| C | -1.156701 | -3.661862 | -1.408606 |
| H | -1.539074 | -4.295963 | -0.600827 |
| H | -0.537361 | -2.875250 | -0.959795 |
| H | -0.490881 | -4.282274 | -2.020230 |
| C | -1.714846 | -2.239574 | -3.414534 |
| H | -1.063688 | -2.851828 | -4.049961 |
| H | -1.124066 | -1.399476 | -3.031031 |
| H | -2.507270 | -1.821256 | -4.044482 |

TS Conformation 118

B3LYP/6-31G(d) Energy = -3411.866311
M06-2X/def2tzvpp/IEFPCM(chloroform) Energy = -3411.881271
M06-2X/def2tzvpp/IEFPCM(chloroform)//B3LYP/6-31G(d) Quasiharmonic Free Energy = -3411.156616

Frequencies (Top 3 out of 291)

1. -898.7768 cm-1
2. 8.0936 cm-1
3. 9.9760 cm-1

B3LYP/6-31G(d) Molecular Geometry in Cartesian Coordinates

| | | | |
|---|---|---|---|
| C | -3.646379 | -3.005450 | -1.094570 |
| C | -2.675739 | -2.069140 | -1.858190 |
| H | -3.318710 | -1.687150 | -2.654470 |
| C | -2.808130 | 0.334560 | -1.184900 |
| N | -2.101490 | 1.268960 | -0.457590 |
| H | -1.365460 | 0.896130 | 0.134560 |
| N | -2.220920 | -0.898230 | -1.117820 |
| H | -1.297170 | -0.942220 | -0.703060 |
| C | -2.598249 | -2.803410 | 1.264270 |
| C | -4.552309 | -4.192000 | 0.814830 |
| C | -2.566169 | -4.062480 | 2.166770 |
| H | -1.630569 | -2.662230 | 0.779350 |
| C | -4.046859 | -4.458120 | 2.239940 |
| H | -5.581579 | -3.821741 | 0.780160 |
| H | -4.512379 | -5.084240 | 0.182040 |
| H | -2.117999 | -3.875300 | 3.146000 |
| H | -1.983579 | -4.846420 | 1.667450 |
| H | -4.567689 | -3.821090 | 2.963190 |
| H | -4.197739 | -5.498700 | 2.541310 |
| N | -3.614699 | -3.182030 | 0.268870 |
| O | -4.484619 | -3.608380 | -1.760740 |
| S | -4.238510 | 0.641940 | -2.009890 |
| C | -2.938040 | -1.542970 | 2.066650 |
| C | -4.069150 | -0.765570 | 1.802120 |
| C | -2.093180 | -1.154560 | 3.118780 |
| C | -4.351350 | 0.369730 | 2.567230 |
| H | -4.723880 | -1.039430 | 0.981450 |
| C | -2.376080 | -0.027310 | 3.891050 |

| | | | |
|---|---|---|---|
| C | -3.510870 | 0.742230 | 3.616040 |
| H | -5.231250 | 0.963169 | 2.335890 |
| O | 4.231130 | 1.090390 | 2.169390 |
| O | 0.374290 | -0.147380 | 0.643090 |
| C | 4.052710 | 1.305390 | 0.971970 |
| C | 2.885900 | 0.813620 | 0.198240 |
| O | 4.922680 | 1.963700 | 0.198030 |
| C | 6.170690 | 2.327340 | 0.822670 |
| H | 6.752610 | 1.426400 | 1.030850 |
| H | 6.681990 | 2.953780 | 0.092080 |
| H | 5.991200 | 2.876780 | 1.749180 |
| S | 1.621730 | 0.239280 | 1.357000 |
| C | 2.267150 | -1.173260 | 2.271200 |
| H | 3.110020 | -0.836560 | 2.873710 |
| H | 1.437040 | -1.558010 | 2.868110 |
| H | 2.589790 | -1.887870 | 1.501980 |
| C | 1.218150 | 1.482090 | 2.611330 |
| H | 2.128430 | 1.721730 | 3.161240 |
| H | 0.831380 | 2.349360 | 2.070980 |
| H | 0.438280 | 1.054660 | 3.246910 |
| C | 2.274950 | 1.680090 | -0.869160 |
| C | 2.106440 | 3.062670 | -0.677110 |
| C | 1.841350 | 1.104410 | -2.072450 |
| C | 1.515910 | 3.849740 | -1.663000 |
| H | 2.458850 | 3.528620 | 0.240170 |
| C | 1.249200 | 1.894780 | -3.058220 |
| H | 1.993410 | 0.043680 | -2.242380 |
| C | 1.080200 | 3.264740 | -2.854520 |
| H | 1.391600 | 4.916510 | -1.500480 |
| H | 0.920390 | 1.436740 | -3.986550 |
| H | 0.614220 | 3.876850 | -3.621490 |
| C | 6.215090 | -1.007020 | -0.961870 |
| C | 7.564800 | -1.040070 | -0.605630 |
| C | 8.057391 | -2.055760 | 0.217210 |
| C | 7.182031 | -3.040640 | 0.679420 |
| C | 5.831171 | -3.006290 | 0.332520 |
| C | 5.322051 | -1.987650 | -0.492590 |
| H | 5.845320 | -0.225030 | -1.618510 |
| H | 8.238320 | -0.276640 | -0.989600 |
| H | 9.109791 | -2.084080 | 0.486760 |
| H | 7.551521 | -3.841900 | 1.315380 |
| H | 5.157221 | -3.777910 | 0.694250 |
| S | 3.597221 | -1.970040 | -0.954340 |
| H | 3.239440 | -0.394070 | -0.354210 |
| H | -1.213980 | -1.753760 | 3.349760 |
| H | -1.725960 | 0.238410 | 4.722410 |
| H | -3.737800 | 1.618650 | 4.216900 |
| C | -1.481349 | -2.815940 | -2.559040 |
| C | -2.318170 | 2.647290 | -0.291560 |
| C | -2.764850 | 3.486980 | -1.320830 |
| C | -1.981640 | 3.194290 | 0.959050 |
| C | -2.866700 | 4.847180 | -1.062720 |
| H | -3.033320 | 3.096310 | -2.291400 |
| C | -2.090230 | 4.564310 | 1.148230 |
| H | -1.701100 | 2.556340 | 1.789760 |
| C | -2.531890 | 5.429310 | 0.156280 |
| H | -2.621060 | 6.495560 | 0.323060 |
| F | -3.289650 | 5.654980 | -2.055130 |
| F | -1.762420 | 5.073330 | 2.356840 |
| C | -0.477059 | -3.412920 | -1.552610 |
| H | -0.952029 | -4.159870 | -0.905360 |
| H | 0.336561 | -3.913880 | -2.088580 |

| | | | |
|---|---|---|---|
| H | -0.001429 | -2.653530 | -0.918910 |
| C | -0.752940 | -1.804410 | -3.469420 |
| H | -1.440610 | -1.375530 | -4.207380 |
| H | -0.312020 | -0.978240 | -2.902250 |
| H | 0.057491 | -2.305790 | -4.010530 |
| C | -2.027349 | -3.953720 | -3.445880 |
| H | -2.520339 | -4.734240 | -2.861810 |
| H | -2.758709 | -3.578580 | -4.168940 |
| H | -1.198079 | -4.409150 | -4.000120 |

TS Conformation 119

B3LYP/6-31G(d) Energy = -3411.866311
M06-2X/def2tzvpp/IEFPCM(chloroform) Energy = -3411.88127
M06-2X/def2tzvpp/IEFPCM(chloroform)//B3LYP/6-31G(d) Quasiharmonic Free Energy = -3411.156614

Frequencies (Top 3 out of 291)

1. -898.8251 cm-1
2. 8.1000 cm-1
3. 9.9809 cm-1

B3LYP/6-31G(d) Molecular Geometry in Cartesian Coordinates

| | | | |
|---|---|---|---|
| C | -3.646209 | -3.005550 | -1.094530 |
| C | -2.675679 | -2.069170 | -1.858210 |
| H | -3.318729 | -1.687220 | -2.654450 |
| C | -2.808240 | 0.334480 | -1.184810 |
| N | -2.101580 | 1.268930 | -0.457580 |
| H | -1.365430 | 0.896160 | 0.134430 |
| N | -2.220870 | -0.898250 | -1.117860 |
| H | -1.297020 | -0.942130 | -0.703310 |
| C | -2.597899 | -2.803500 | 1.264230 |
| C | -4.551899 | -4.192210 | 0.814910 |
| C | -2.565629 | -4.062620 | 2.166650 |
| H | -1.630289 | -2.662190 | 0.779220 |
| C | -4.046279 | -4.458380 | 2.239960 |
| H | -5.581199 | -3.822000 | 0.780380 |
| H | -4.511989 | -5.084410 | 0.182070 |
| H | -1.983019 | -4.846480 | 1.667210 |
| H | -2.117359 | -3.875470 | 3.145840 |
| H | -4.567069 | -3.821430 | 2.963300 |
| H | -4.197039 | -5.498980 | 2.541280 |
| N | -3.614409 | -3.182160 | 0.268900 |
| O | -4.484459 | -3.608520 | -1.760660 |
| S | -4.238790 | 0.641710 | -2.009550 |
| C | -2.937739 | -1.543130 | 2.066700 |
| C | -2.092880 | -1.154740 | 3.118840 |
| C | -4.068890 | -0.765770 | 1.802240 |
| C | -2.375810 | -0.027550 | 3.891180 |
| H | -1.213649 | -1.753920 | 3.349780 |
| C | -4.351120 | 0.369480 | 2.567410 |
| C | -3.510640 | 0.741960 | 3.616230 |
| H | -1.725690 | 0.238150 | 4.722550 |
| O | 4.231050 | 1.090671 | 2.169280 |
| O | 0.374280 | -0.147370 | 0.643000 |
| C | 4.052620 | 1.305620 | 0.971850 |
| C | 2.885850 | 0.813740 | 0.198150 |
| O | 4.922550 | 1.963961 | 0.197890 |
| C | 6.170550 | 2.327671 | 0.822510 |
| H | 6.681840 | 2.954071 | 0.091870 |

| | | | |
|---|---|---|---|
| H | 6.752490 | 1.426761 | 1.030760 |
| H | 5.991040 | 2.877181 | 1.748980 |
| S | 1.621690 | 0.239400 | 1.356920 |
| C | 2.267160 | -1.173050 | 2.271210 |
| H | 2.589981 | -1.887650 | 1.502070 |
| H | 3.109940 | -0.836240 | 2.873800 |
| H | 1.437031 | -1.557860 | 2.868040 |
| C | 1.218030 | 1.482270 | 2.611160 |
| H | 0.831220 | 2.349480 | 2.070750 |
| H | 0.438180 | 1.054840 | 3.246760 |
| H | 2.128290 | 1.721990 | 3.161060 |
| C | 2.274870 | 1.680080 | -0.869340 |
| C | 2.106310 | 3.062680 | -0.677420 |
| C | 1.841290 | 1.104270 | -2.072570 |
| C | 1.515750 | 3.849630 | -1.663390 |
| H | 2.458710 | 3.528730 | 0.239800 |
| C | 1.249100 | 1.894530 | -3.058420 |
| H | 1.993400 | 0.043540 | -2.242410 |
| C | 1.080050 | 3.264500 | -2.854850 |
| H | 1.391390 | 4.916410 | -1.500970 |
| H | 0.920310 | 1.436380 | -3.986710 |
| H | 0.614050 | 3.876520 | -3.621880 |
| C | 6.215050 | -1.006769 | -0.961850 |
| C | 7.564790 | -1.039669 | -0.605730 |
| C | 8.057541 | -2.055259 | 0.217140 |
| C | 7.182331 | -3.040209 | 0.679480 |
| C | 5.831431 | -3.006009 | 0.332690 |
| C | 5.322141 | -1.987469 | -0.492440 |
| H | 5.845140 | -0.224859 | -1.618510 |
| H | 8.238190 | -0.276199 | -0.989790 |
| H | 9.109971 | -2.083469 | 0.486600 |
| H | 7.551941 | -3.841389 | 1.315460 |
| H | 5.157581 | -3.777669 | 0.694540 |
| S | 3.597271 | -1.970060 | -0.954050 |
| H | 3.239430 | -0.394010 | -0.354160 |
| H | -4.723630 | -1.039620 | 0.981570 |
| H | -5.231050 | 0.962890 | 2.336130 |
| H | -3.737600 | 1.618340 | 4.217140 |
| C | -1.481299 | -2.815900 | -2.559160 |
| C | -2.318400 | 2.647240 | -0.291500 |
| C | -1.981780 | 3.194250 | 0.959090 |
| C | -2.765300 | 3.486900 | -1.320700 |
| C | -2.090490 | 4.564250 | 1.148310 |
| H | -1.701070 | 2.556310 | 1.789750 |
| C | -2.867260 | 4.847080 | -1.062550 |
| H | -3.033830 | 3.096210 | -2.291240 |
| C | -2.532370 | 5.429220 | 0.156430 |
| H | -2.621630 | 6.495460 | 0.323240 |
| F | -1.762600 | 5.073280 | 2.356890 |
| F | -3.290420 | 5.654860 | -2.054880 |
| C | -0.476909 | -3.412850 | -1.552820 |
| H | -0.951789 | -4.159840 | -0.905550 |
| H | 0.336711 | -3.913750 | -2.088860 |
| H | -0.001269 | -2.653450 | -0.919130 |
| C | -0.753009 | -1.804310 | -3.469580 |
| H | -1.440760 | -1.375440 | -4.207460 |
| H | 0.057391 | -2.305650 | -4.010770 |
| H | -0.312070 | -0.978140 | -2.902430 |
| C | -2.027299 | -3.953690 | -3.445990 |
| H | -2.758719 | -3.578560 | -4.168990 |
| H | -2.520219 | -4.734230 | -2.861900 |
| H | -1.198039 | -4.409070 | -4.000290 |

TS Conformation 120

B3LYP/6-31G(d) Energy = -3411.866024
M06-2X/def2tzvpp/IEFPCM(chloroform) Energy = -3411.880608
M06-2X/def2tzvpp/IEFPCM(chloroform)//B3LYP/6-31G(d) Quasiharmonic Free Energy = -3411.156426

Frequencies (Top 3 out of 291)

1. -735.5144 cm-1
2. 5.7373 cm-1
3. 8.1667 cm-1

B3LYP/6-31G(d) Molecular Geometry in Cartesian Coordinates

| | | | |
|---|---|---|---|
| C | 1.577922 | 4.019271 | -0.383027 |
| C | 1.384769 | 2.904357 | -1.440838 |
| H | 2.153295 | 3.144111 | -2.178571 |
| C | 2.835491 | 0.871243 | -1.292861 |
| N | 2.851146 | -0.363438 | -0.653614 |
| H | 2.286498 | -0.403688 | 0.188416 |
| N | 1.696620 | 1.555793 | -0.980295 |
| H | 0.939367 | 1.002191 | -0.594699 |
| C | 0.820557 | 2.683236 | 1.700612 |
| C | 1.507873 | 5.024663 | 1.822711 |
| C | 0.063089 | 3.398257 | 2.848889 |
| H | 0.107502 | 2.141922 | 1.076611 |
| C | 0.994241 | 4.567196 | 3.194840 |
| H | 2.540349 | 5.386776 | 1.841440 |
| H | 0.897624 | 5.826117 | 1.392611 |
| H | -0.141954 | 2.732938 | 3.691586 |
| H | -0.894093 | 3.772695 | 2.465383 |
| H | 1.821234 | 4.215133 | 3.821219 |
| H | 0.487659 | 5.373506 | 3.733042 |
| N | 1.391704 | 3.822605 | 0.964196 |
| O | 1.908738 | 5.131669 | -0.788520 |
| S | 4.065676 | 1.451930 | -2.269900 |
| C | 1.855583 | 1.698679 | 2.248077 |
| C | 3.231927 | 1.942173 | 2.190723 |
| C | 1.405152 | 0.527167 | 2.878371 |
| C | 4.139305 | 1.047443 | 2.763894 |
| H | 3.594460 | 2.830214 | 1.682824 |
| C | 2.310167 | -0.364785 | 3.456580 |
| C | 3.683276 | -0.104417 | 3.406164 |
| H | 5.204409 | 1.253412 | 2.705538 |
| O | -2.300744 | -4.544519 | -0.386798 |
| O | -0.207744 | -0.763635 | 0.091483 |
| C | -2.906653 | -3.676348 | 0.235581 |
| C | -2.471442 | -2.259230 | 0.334639 |
| O | -4.095619 | -3.868495 | 0.815062 |
| C | -4.699989 | -5.159125 | 0.593764 |
| H | -5.642951 | -5.128474 | 1.138536 |
| H | -4.875288 | -5.312253 | -0.473380 |
| H | -4.056015 | -5.954793 | 0.975960 |
| S | -0.740680 | -2.121885 | -0.191113 |
| C | 0.307899 | -3.354720 | 0.621658 |
| H | -0.101100 | -4.342900 | 0.411020 |
| H | 1.324443 | -3.232331 | 0.236421 |
| H | 0.275328 | -3.122250 | 1.688483 |
| C | -0.636640 | -2.500583 | -1.949853 |
| H | -0.949730 | -3.534792 | -2.094832 |

| | | | |
|---|---|---|---|
| H | -1.325973 | -1.806097 | -2.446007 |
| H | 0.401725 | -2.325463 | -2.242233 |
| C | -2.695892 | -1.476882 | 1.597145 |
| C | -3.016689 | -0.113822 | 1.516266 |
| C | -2.564279 | -2.071249 | 2.863932 |
| C | -3.205018 | 0.636442 | 2.677763 |
| H | -3.140154 | 0.350139 | 0.543815 |
| C | -2.754868 | -1.321064 | 4.022719 |
| H | -2.336518 | -3.131321 | 2.942932 |
| C | -3.071798 | 0.037059 | 3.931383 |
| H | -3.468115 | 1.687360 | 2.598928 |
| H | -2.659831 | -1.795847 | 4.995377 |
| H | -3.224029 | 0.621354 | 4.834809 |
| C | -4.705287 | 1.409182 | -2.635557 |
| C | -5.461128 | 2.553623 | -2.377895 |
| C | -6.344756 | 2.591343 | -1.296811 |
| C | -6.470235 | 1.463507 | -0.482608 |
| C | -5.724822 | 0.313209 | -0.743767 |
| C | -4.821042 | 0.266809 | -1.821599 |
| H | -4.021894 | 1.385234 | -3.479658 |
| H | -5.357292 | 3.420667 | -3.026452 |
| H | -6.931589 | 3.483774 | -1.096162 |
| H | -7.161690 | 1.472796 | 0.357219 |
| H | -5.844815 | -0.565322 | -0.116105 |
| S | -3.884728 | -1.201205 | -2.199173 |
| H | -3.117667 | -1.692588 | -0.690095 |
| H | 0.338572 | 0.318238 | 2.923983 |
| H | 1.942780 | -1.257123 | 3.958437 |
| H | 4.389049 | -0.793934 | 3.861505 |
| C | 0.000018 | 2.976081 | -2.186956 |
| C | 3.693726 | -1.469617 | -0.836407 |
| C | 3.904838 | -2.303737 | 0.278031 |
| C | 4.243507 | -1.819224 | -2.079340 |
| C | 4.663886 | -3.456540 | 0.123485 |
| H | 3.538114 | -2.028801 | 1.261471 |
| C | 5.002098 | -2.978947 | -2.162165 |
| H | 4.108438 | -1.199031 | -2.953546 |
| C | 5.231994 | -3.831440 | -1.086284 |
| H | 5.828544 | -4.729492 | -1.186407 |
| F | 4.860962 | -4.241169 | 1.203761 |
| F | 5.527136 | -3.310801 | -3.356560 |
| C | -1.192579 | 2.746571 | -1.237898 |
| H | -1.229029 | 3.504994 | -0.446598 |
| H | -2.135560 | 2.805578 | -1.792141 |
| H | -1.172488 | 1.753957 | -0.771112 |
| C | -0.010760 | 1.899774 | -3.292337 |
| H | 0.844316 | 2.016864 | -3.967399 |
| H | 0.020043 | 0.885617 | -2.880640 |
| H | -0.928601 | 1.983964 | -3.884748 |
| C | -0.152319 | 4.357584 | -2.856007 |
| H | -0.171440 | 5.170509 | -2.126931 |
| H | 0.674527 | 4.557092 | -3.546055 |
| H | -1.087718 | 4.381952 | -3.427533 |

TS Conformation 121

B3LYP/6-31G(d) Energy = -3411.867386
M06-2X/def2tzvpp/IEFPCM(chloroform) Energy = -3411.880461
M06-2X/def2tzvpp/IEFPCM(chloroform)//B3LYP/6-31G(d) Quasiharmonic Free Energy = -3411.15631

Frequencies (Top 3 out of 291)

1. -946.0856 cm-1
2. 4.8166 cm-1
3. 8.3191 cm-1

B3LYP/6-31G(d) Molecular Geometry in Cartesian Coordinates

| | | | |
|---|---|---|---|
| C | -3.424358 | -3.446473 | -0.322544 |
| C | -3.159328 | -2.366045 | -1.401736 |
| H | -4.168034 | -2.137694 | -1.753310 |
| C | -3.379476 | 0.037708 | -0.759936 |
| N | -2.603448 | 1.106745 | -0.363302 |
| H | -1.671528 | 0.873748 | -0.035877 |
| N | -2.629435 | -1.097096 | -0.917367 |
| H | -1.622528 | -0.979141 | -0.903820 |
| C | -1.486651 | -2.856540 | 1.293862 |
| C | -3.041835 | -4.690662 | 1.720537 |
| C | -0.768779 | -3.985699 | 2.074353 |
| H | -0.879317 | -2.561345 | 0.435472 |
| C | -1.930623 | -4.697060 | 2.779448 |
| H | -4.046100 | -4.577025 | 2.140024 |
| H | -3.050520 | -5.603611 | 1.115534 |
| H | -0.281910 | -4.661712 | 1.360625 |
| H | -0.005104 | -3.604299 | 2.755712 |
| H | -2.235489 | -4.128031 | 3.664348 |
| H | -1.678928 | -5.710898 | 3.103832 |
| N | -2.711965 | -3.541940 | 0.843727 |
| O | -4.323005 | -4.255704 | -0.552892 |
| S | -5.041787 | 0.096386 | -0.994718 |
| C | -1.736376 | -1.632245 | 2.177738 |
| C | -3.021806 | -1.204457 | 2.524810 |
| C | -0.631414 | -0.931452 | 2.687359 |
| C | -3.203640 | -0.105605 | 3.368625 |
| H | -3.885686 | -1.724435 | 2.123531 |
| C | -0.812635 | 0.159877 | 3.538004 |
| C | -2.101454 | 0.576802 | 3.883577 |
| H | -4.210895 | 0.214647 | 3.620110 |
| O | 4.421479 | 0.741827 | -2.634895 |
| O | 0.394051 | 0.104698 | -1.111131 |
| C | 4.179768 | 1.247685 | -1.541010 |
| C | 2.915474 | 1.076297 | -0.782189 |
| O | 5.063204 | 1.986750 | -0.857715 |
| C | 6.384714 | 2.076172 | -1.426476 |
| H | 6.904115 | 2.821098 | -0.824003 |
| H | 6.334304 | 2.386782 | -2.472097 |
| H | 6.883769 | 1.107001 | -1.350680 |
| S | 1.686370 | 0.268886 | -1.826479 |
| C | 1.409919 | 1.170722 | -3.372650 |
| H | 2.370320 | 1.271885 | -3.880181 |
| H | 0.687199 | 0.601780 | -3.962851 |
| H | 0.994640 | 2.141161 | -3.092261 |
| C | 2.336879 | -1.335821 | -2.328993 |
| H | 3.239303 | -1.172433 | -2.916221 |
| H | 2.558218 | -1.857849 | -1.389791 |
| H | 1.542537 | -1.832370 | -2.889928 |
| C | 2.336487 | 2.269689 | -0.069152 |
| C | 1.777633 | 2.126069 | 1.208842 |
| C | 2.347554 | 3.543054 | -0.665867 |
| C | 1.244688 | 3.232505 | 1.871184 |
| H | 1.783182 | 1.152799 | 1.687149 |
| C | 1.812155 | 4.644862 | -0.002724 |
| H | 2.798049 | 3.676220 | -1.646860 |

| | | | |
|---|---|---|---|
| C | 1.252914 | 4.490600 | 1.267747 |
| H | 0.815444 | 3.110047 | 2.861492 |
| H | 1.828788 | 5.621822 | -0.477770 |
| H | 0.812342 | 5.340559 | 1.778914 |
| C | 5.446178 | -2.855389 | 0.762272 |
| C | 6.819730 | -3.089188 | 0.691176 |
| C | 7.727469 | -2.053113 | 0.919966 |
| C | 7.242992 | -0.780528 | 1.231056 |
| C | 5.869438 | -0.544525 | 1.313874 |
| C | 4.946980 | -1.578556 | 1.073196 |
| H | 4.746285 | -3.666265 | 0.580752 |
| H | 7.181312 | -4.086840 | 0.452901 |
| H | 8.797167 | -2.236229 | 0.863841 |
| H | 7.938215 | 0.032674 | 1.428705 |
| H | 5.503281 | 0.443746 | 1.575251 |
| S | 3.186207 | -1.299738 | 1.181863 |
| H | 3.095779 | 0.046381 | 0.135833 |
| H | 0.378558 | -1.248046 | 2.433378 |
| H | 0.055979 | 0.673659 | 3.942798 |
| H | -2.243818 | 1.424699 | 4.548293 |
| C | -2.359655 | -2.916122 | -2.641257 |
| C | -2.965014 | 2.445635 | -0.111503 |
| C | -3.908209 | 3.145135 | -0.877225 |
| C | -2.264529 | 3.105304 | 0.910301 |
| C | -4.131868 | 4.483091 | -0.582266 |
| H | -4.464327 | 2.665571 | -1.669039 |
| C | -2.522093 | 4.447421 | 1.142385 |
| H | -1.545377 | 2.579586 | 1.526654 |
| C | -3.455665 | 5.175426 | 0.416970 |
| H | -3.651350 | 6.221040 | 0.619708 |
| F | -5.038375 | 5.156170 | -1.319663 |
| F | -1.835963 | 5.073943 | 2.127200 |
| C | -0.973117 | -3.455879 | -2.240578 |
| H | -1.052814 | -4.300855 | -1.547781 |
| H | -0.434779 | -3.811484 | -3.128363 |
| H | -0.351414 | -2.688268 | -1.763834 |
| C | -2.193798 | -1.773701 | -3.665704 |
| H | -3.163793 | -1.341523 | -3.934810 |
| H | -1.730344 | -2.159522 | -4.582007 |
| H | -1.566374 | -0.960804 | -3.282463 |
| C | -3.159054 | -4.050565 | -3.315014 |
| H | -4.142991 | -3.696911 | -3.641186 |
| H | -3.323065 | -4.892711 | -2.640558 |
| H | -2.615355 | -4.407291 | -4.198730 |

TS Conformation 122

B3LYP/6-31G(d) Energy = -3411.86808
M06-2X/def2tzvpp/IEFPCM(chloroform) Energy = -3411.879194
M06-2X/def2tzvpp/IEFPCM(chloroform)//B3LYP/6-31G(d) Quasiharmonic Free Energy = -3411.156206

Frequencies (Top 3 out of 291)

1. -805.0111 cm-1
2. 4.2645 cm-1
3. 5.6297 cm-1

B3LYP/6-31G(d) Molecular Geometry in Cartesian Coordinates

| | | | |
|---|---|---|---|
| C | 4.363780 | -0.241860 | 0.989638 |
| C | 3.095842 | 0.093323 | 1.818627 |

| | | | |
|---|---|---|---|
| H | 3.498480 | 0.747625 | 2.596419 |
| C | 2.091044 | 2.182040 | 0.873152 |
| N | 0.873491 | 2.636787 | 0.398428 |
| H | 0.112474 | 1.968489 | 0.470055 |
| N | 2.054113 | 0.844056 | 1.126548 |
| H | 1.213385 | 0.338675 | 0.873001 |
| C | 5.624445 | -0.677855 | -1.043928 |
| C | 3.241380 | -0.172056 | -1.324062 |
| C | 5.321020 | -0.288675 | -2.511394 |
| H | 6.359039 | 0.000483 | -0.601765 |
| C | 3.830182 | -0.610903 | -2.672142 |
| H | 2.329143 | -0.719753 | -1.068693 |
| H | 3.006346 | 0.898649 | -1.341732 |
| H | 5.959968 | -0.822264 | -3.219624 |
| H | 5.487347 | 0.787035 | -2.644507 |
| H | 3.688375 | -1.687331 | -2.821940 |
| H | 3.363712 | -0.088827 | -3.514008 |
| N | 4.325178 | -0.454493 | -0.366985 |
| O | 5.418486 | -0.366712 | 1.605405 |
| S | 3.462200 | 3.115193 | 1.125696 |
| C | 6.160186 | -2.097597 | -0.901247 |
| C | 7.546097 | -2.298916 | -0.875340 |
| C | 5.324205 | -3.219479 | -0.856013 |
| C | 8.084177 | -3.584028 | -0.814906 |
| H | 8.209840 | -1.437159 | -0.890734 |
| C | 5.857995 | -4.508140 | -0.792082 |
| C | 7.240402 | -4.695754 | -0.774559 |
| H | 9.162722 | -3.716456 | -0.788235 |
| O | -4.368624 | -0.802230 | -2.401512 |
| O | -0.810309 | 0.167459 | -0.141703 |
| C | -4.571153 | -0.473997 | -1.234428 |
| C | -3.497280 | -0.267912 | -0.228411 |
| O | -5.788170 | -0.320147 | -0.704346 |
| C | -6.896815 | -0.677488 | -1.556223 |
| H | -7.786395 | -0.365752 | -1.009520 |
| H | -6.826744 | -0.159574 | -2.514975 |
| H | -6.901876 | -1.758322 | -1.714838 |
| S | -1.905336 | -0.174828 | -1.090554 |
| C | -1.573794 | -1.767031 | -1.866158 |
| H | -2.322481 | -1.933649 | -2.639988 |
| H | -0.555045 | -1.713457 | -2.257479 |
| H | -1.668159 | -2.503634 | -1.055725 |
| C | -1.913784 | 1.030392 | -2.444032 |
| H | -0.944759 | 0.962183 | -2.944797 |
| H | -2.740394 | 0.785912 | -3.111550 |
| H | -2.032045 | 2.014863 | -1.984623 |
| C | -3.620884 | 0.832394 | 0.789455 |
| C | -3.155610 | 0.625541 | 2.097238 |
| C | -4.176582 | 2.079467 | 0.453301 |
| C | -3.248346 | 1.644660 | 3.046075 |
| H | -2.747361 | -0.341226 | 2.372510 |
| C | -4.268672 | 3.095555 | 1.402690 |
| H | -4.553806 | 2.251781 | -0.551571 |
| C | -3.800569 | 2.879326 | 2.701784 |
| H | -2.890000 | 1.470463 | 4.056573 |
| H | -4.685390 | 4.058179 | 1.122572 |
| H | -3.867485 | 3.671958 | 3.441739 |
| C | -5.660767 | -3.473151 | 0.660635 |
| C | -6.716356 | -4.195862 | 0.100933 |
| C | -6.468324 | -5.339867 | -0.661457 |
| C | -5.148877 | -5.753852 | -0.855709 |
| C | -4.091379 | -5.030137 | -0.304191 |

| | | | |
|---|---|---|---|
| C | -4.326641 | -3.874428 | 0.462409 |
| H | -5.864450 | -2.596637 | 1.268737 |
| H | -7.739905 | -3.871974 | 0.278232 |
| H | -7.291617 | -5.905366 | -1.089723 |
| H | -4.939427 | -6.646574 | -1.440676 |
| H | -3.067991 | -5.361708 | -0.456680 |
| S | -2.967887 | -2.970739 | 1.186411 |
| H | -3.318424 | -1.440321 | 0.418603 |
| H | 4.246153 | -3.086138 | -0.853039 |
| H | 5.191079 | -5.365710 | -0.750751 |
| H | 7.656736 | -5.698115 | -0.720503 |
| C | 2.473946 | -1.160094 | 2.537916 |
| C | 0.456147 | 3.885784 | -0.084642 |
| C | 1.310542 | 4.862429 | -0.616098 |
| C | -0.935335 | 4.109413 | -0.074959 |
| C | 0.745155 | 6.031278 | -1.110407 |
| H | 2.382216 | 4.730755 | -0.627986 |
| C | -1.433500 | 5.295783 | -0.591042 |
| H | -1.618166 | 3.396697 | 0.374451 |
| C | -0.621787 | 6.289032 | -1.123139 |
| H | -1.026034 | 7.213785 | -1.515279 |
| F | 1.569337 | 6.965106 | -1.622949 |
| F | -2.771570 | 5.493342 | -0.567809 |
| C | 3.555235 | -1.869615 | 3.378492 |
| H | 4.019739 | -1.180663 | 4.091506 |
| H | 3.092375 | -2.687360 | 3.943512 |
| H | 4.353959 | -2.284691 | 2.760551 |
| C | 1.883331 | -2.172036 | 1.534097 |
| H | 1.051291 | -1.759099 | 0.949560 |
| H | 2.648765 | -2.536514 | 0.840585 |
| H | 1.481480 | -3.038283 | 2.071213 |
| C | 1.362864 | -0.677667 | 3.496379 |
| H | 1.752505 | 0.055624 | 4.212072 |
| H | 0.518933 | -0.218565 | 2.970188 |
| H | 0.968707 | -1.527606 | 4.064612 |

TS Conformation 123

B3LYP/6-31G(d) Energy = -3411.866279
M06-2X/def2tzvpp/IEFPCM(chloroform) Energy = -3411.880716
M06-2X/def2tzvpp/IEFPCM(chloroform)//B3LYP/6-31G(d) Quasiharmonic Free Energy = -3411.156068

Frequencies (Top 3 out of 291)

1. -963.3000 cm-1
2. 5.3437 cm-1
3. 11.4458 cm-1

B3LYP/6-31G(d) Molecular Geometry in Cartesian Coordinates

| | | | |
|---|---|---|---|
| C | -4.477362 | -2.240373 | -0.511153 |
| C | -3.889186 | -1.104851 | -1.384832 |
| H | -4.755147 | -0.460936 | -1.555494 |
| C | -3.160876 | 1.034558 | -0.320371 |
| N | -2.058593 | 1.581445 | 0.308137 |
| H | -1.406897 | 0.907011 | 0.696991 |
| N | -2.907743 | -0.248869 | -0.729845 |
| H | -1.929286 | -0.505897 | -0.799785 |
| C | -2.402505 | -2.679532 | 0.974410 |
| C | -4.471413 | -3.962480 | 1.195168 |
| C | -2.088496 | -4.108343 | 1.488410 |

| | | | |
|---|---|---|---|
| H | -1.754811 | -2.445293 | 0.126361 |
| C | -3.408762 | -4.532287 | 2.144523 |
| H | -5.379024 | -3.626789 | 1.705760 |
| H | -4.784492 | -4.685323 | 0.433528 |
| H | -1.233709 | -4.125821 | 2.169489 |
| H | -1.863999 | -4.758627 | 0.632908 |
| H | -3.494109 | -4.079397 | 3.137988 |
| H | -3.500482 | -5.615913 | 2.261706 |
| N | -3.798468 | -2.823977 | 0.528088 |
| O | -5.609563 | -2.636264 | -0.787359 |
| S | -4.632128 | 1.816432 | -0.520449 |
| C | -2.187090 | -1.635990 | 2.070431 |
| C | -3.247546 | -1.029618 | 2.752043 |
| C | -0.873369 | -1.316583 | 2.448164 |
| C | -3.001451 | -0.140170 | 3.801118 |
| H | -4.268792 | -1.242270 | 2.451779 |
| C | -0.624207 | -0.431118 | 3.498400 |
| C | -1.692353 | 0.155393 | 4.183906 |
| H | -3.837995 | 0.324333 | 4.315914 |
| O | 4.372561 | -1.661511 | -2.337190 |
| O | 0.303093 | -0.651195 | -1.209275 |
| C | 4.217474 | -0.533257 | -1.873377 |
| C | 2.945523 | -0.012132 | -1.316156 |
| O | 5.208261 | 0.360844 | -1.761728 |
| C | 6.521807 | -0.106315 | -2.127648 |
| H | 6.865086 | -0.846569 | -1.400971 |
| H | 6.509874 | -0.545937 | -3.127268 |
| H | 7.156381 | 0.779305 | -2.100282 |
| S | 1.606977 | -1.152409 | -1.713937 |
| C | 1.493082 | -1.455505 | -3.496769 |
| H | 2.457655 | -1.835521 | -3.835749 |
| H | 0.682444 | -2.168846 | -3.662477 |
| H | 1.253724 | -0.492818 | -3.953832 |
| C | 1.977697 | -2.761923 | -0.987458 |
| H | 1.096295 | -3.385117 | -1.156815 |
| H | 2.877806 | -3.157412 | -1.455515 |
| H | 2.137468 | -2.562443 | 0.079177 |
| C | 2.544823 | 1.408666 | -1.609821 |
| C | 2.795791 | 1.983680 | -2.869037 |
| C | 1.898559 | 2.178074 | -0.631785 |
| C | 2.417887 | 3.296528 | -3.139169 |
| H | 3.313201 | 1.407860 | -3.632766 |
| C | 1.516053 | 3.491978 | -0.905640 |
| H | 1.716394 | 1.754319 | 0.349954 |
| C | 1.772766 | 4.052219 | -2.157058 |
| H | 2.626040 | 3.728986 | -4.114020 |
| H | 1.017509 | 4.075849 | -0.138594 |
| H | 1.473524 | 5.075401 | -2.366691 |
| C | 4.847182 | -2.408972 | 2.466241 |
| C | 6.154743 | -2.863740 | 2.639955 |
| C | 7.237436 | -2.065256 | 2.266249 |
| C | 6.995687 | -0.800326 | 1.725154 |
| C | 5.689059 | -0.337404 | 1.558777 |
| C | 4.591546 | -1.138896 | 1.920790 |
| H | 4.009982 | -3.036023 | 2.760150 |
| H | 6.326888 | -3.848450 | 3.068353 |
| H | 8.255547 | -2.420039 | 2.402310 |
| H | 7.829675 | -0.159685 | 1.446476 |
| H | 5.510938 | 0.655039 | 1.155790 |
| S | 2.914101 | -0.554768 | 1.729859 |
| H | 2.981422 | -0.206778 | 0.072030 |
| H | -0.027440 | -1.765566 | 1.932140 |

| | | | |
|---|---|---|---|
| H | 0.404062 | -0.207775 | 3.769906 |
| H | -1.504154 | 0.843220 | 5.003827 |
| C | -3.417413 | -1.607578 | -2.802302 |
| C | -1.829736 | 2.906005 | 0.725522 |
| C | -2.271514 | 4.017854 | -0.005672 |
| C | -1.052316 | 3.090960 | 1.880458 |
| C | -1.935629 | 5.283405 | 0.453319 |
| H | -2.867667 | 3.909086 | -0.899736 |
| C | -0.743236 | 4.383165 | 2.282556 |
| H | -0.716914 | 2.248667 | 2.474847 |
| C | -1.169320 | 5.511547 | 1.592197 |
| H | -0.928782 | 6.512600 | 1.928033 |
| F | -2.359148 | 6.351849 | -0.252355 |
| F | -0.009080 | 4.550239 | 3.400989 |
| C | -4.629169 | -2.150888 | -3.587764 |
| H | -5.391755 | -1.374842 | -3.714907 |
| H | -4.304996 | -2.473598 | -4.585108 |
| H | -5.101838 | -2.994120 | -3.081187 |
| C | -2.355484 | -2.720898 | -2.696591 |
| H | -1.458420 | -2.377454 | -2.167208 |
| H | -2.745031 | -3.604194 | -2.177587 |
| H | -2.047244 | -3.041114 | -3.700275 |
| C | -2.834817 | -0.410621 | -3.582505 |
| H | -3.547745 | 0.420141 | -3.616943 |
| H | -1.906767 | -0.035041 | -3.139068 |
| H | -2.617947 | -0.712146 | -4.614737 |

TS Conformation 124

B3LYP/6-31G(d) Energy = -3411.86873
M06-2X/def2tzvpp/IEFPCM(chloroform) Energy = -3411.8803
M06-2X/def2tzvpp/IEFPCM(chloroform)//B3LYP/6-31G(d) Quasiharmonic Free Energy = -3411.155826

Frequencies (Top 3 out of 291)

1. -974.5502 cm-1
2. 6.9228 cm-1
3. 8.2810 cm-1

B3LYP/6-31G(d) Molecular Geometry in Cartesian Coordinates

| | | | |
|---|---|---|---|
| C | 3.867021 | -2.509079 | 1.409167 |
| C | 3.093551 | -1.402073 | 2.170542 |
| H | 3.894327 | -0.896496 | 2.714872 |
| C | 3.025798 | 0.831500 | 1.044819 |
| N | 2.075205 | 1.728647 | 0.588548 |
| H | 1.114010 | 1.536275 | 0.859034 |
| N | 2.463310 | -0.373545 | 1.350642 |
| H | 1.484632 | -0.497130 | 1.121484 |
| C | 2.372312 | -2.622975 | -0.703192 |
| C | 4.290766 | -4.100031 | -0.366501 |
| C | 2.126348 | -3.977950 | -1.413997 |
| H | 1.498987 | -2.369401 | -0.099603 |
| C | 3.548429 | -4.503792 | -1.648295 |
| H | 5.336102 | -3.823308 | -0.534852 |
| H | 4.289960 | -4.894980 | 0.387297 |
| H | 1.541602 | -3.864991 | -2.330127 |
| H | 1.576095 | -4.642582 | -0.737068 |
| H | 3.990671 | -4.012484 | -2.521767 |
| H | 3.582947 | -5.583720 | -1.819200 |
| N | 3.524487 | -2.945527 | 0.157435 |

| | | | |
|---|---|---|---|
| O | 4.817602 | -3.026175 | 1.995070 |
| S | 4.652952 | 1.175640 | 1.245751 |
| C | 2.630488 | -1.504184 | -1.712857 |
| C | 3.902191 | -0.970528 | -1.940751 |
| C | 1.554339 | -1.034776 | -2.482562 |
| C | 4.099518 | 0.001000 | -2.926597 |
| H | 4.739200 | -1.300755 | -1.334349 |
| C | 1.750556 | -0.064671 | -3.464986 |
| C | 3.027532 | 0.455448 | -3.693969 |
| H | 5.096190 | 0.402838 | -3.089300 |
| O | -4.442737 | 1.663833 | -1.549741 |
| O | -0.987720 | 1.623047 | 1.081736 |
| C | -4.539047 | 0.962441 | -0.544956 |
| C | -3.439917 | 0.707956 | 0.414724 |
| O | -5.648117 | 0.282339 | -0.222600 |
| C | -6.716519 | 0.323950 | -1.189183 |
| H | -6.947599 | 1.355060 | -1.464483 |
| H | -6.430958 | -0.242542 | -2.078779 |
| H | -7.566336 | -0.146546 | -0.694985 |
| S | -2.079363 | 1.843563 | 0.095266 |
| C | -1.466266 | 1.594269 | -1.581423 |
| H | -2.257980 | 1.854942 | -2.282253 |
| H | -0.578573 | 2.224814 | -1.676618 |
| H | -1.213449 | 0.530257 | -1.642867 |
| C | -2.625535 | 3.571710 | 0.145279 |
| H | -2.967627 | 3.751337 | 1.167040 |
| H | -1.775469 | 4.215325 | -0.095513 |
| H | -3.438341 | 3.679690 | -0.574667 |
| C | -3.736448 | 0.632453 | 1.888492 |
| C | -3.022003 | -0.256895 | 2.705192 |
| C | -4.710155 | 1.458526 | 2.478038 |
| C | -3.277306 | -0.318784 | 4.075330 |
| H | -2.282656 | -0.913767 | 2.260779 |
| C | -4.968545 | 1.390009 | 3.845120 |
| H | -5.285646 | 2.141672 | 1.858530 |
| C | -4.248014 | 0.502862 | 4.648594 |
| H | -2.717943 | -1.015172 | 4.693568 |
| H | -5.730319 | 2.029106 | 4.283117 |
| H | -4.446828 | 0.450744 | 5.715532 |
| C | -4.475546 | -2.770490 | -1.195267 |
| C | -5.404693 | -3.307990 | -2.087789 |
| C | -5.030080 | -3.630738 | -3.394149 |
| C | -3.712878 | -3.407144 | -3.799430 |
| C | -2.783834 | -2.859443 | -2.914279 |
| C | -3.149954 | -2.531036 | -1.598009 |
| H | -4.772029 | -2.540028 | -0.176546 |
| H | -6.423481 | -3.490793 | -1.753029 |
| H | -5.753147 | -4.056776 | -4.084472 |
| H | -3.404792 | -3.656455 | -4.812102 |
| H | -1.762263 | -2.684862 | -3.240196 |
| S | -1.945759 | -1.856458 | -0.462638 |
| H | -2.809638 | -0.493547 | 0.020938 |
| H | 0.557823 | -1.435192 | -2.304073 |
| H | 0.907608 | 0.280424 | -4.059789 |
| H | 3.183523 | 1.208778 | -4.461605 |
| C | 2.091252 | -1.971497 | 3.244333 |
| C | 2.232094 | 2.973759 | -0.036207 |
| C | 3.289993 | 3.288733 | -0.900624 |
| C | 1.200840 | 3.908688 | 0.167612 |
| C | 3.275813 | 4.524319 | -1.533278 |
| H | 4.096470 | 2.594286 | -1.084080 |
| C | 1.230700 | 5.111001 | -0.520423 |

| | | | |
|---|---|---|---|
| H | 0.409642 | 3.707948 | 0.879069 |
| C | 2.257899 | 5.464284 | -1.382609 |
| H | 2.272936 | 6.411439 | -1.906984 |
| F | 4.288708 | 4.822385 | -2.369849 |
| F | 0.193079 | 5.967825 | -0.340240 |
| C | 2.861593 | -2.845281 | 4.256278 |
| H | 3.661573 | -2.275887 | 4.741560 |
| H | 2.171026 | -3.194349 | 5.033612 |
| H | 3.322500 | -3.714700 | 3.783994 |
| C | 0.970139 | -2.818287 | 2.610062 |
| H | 0.341651 | -2.253141 | 1.909452 |
| H | 1.372949 | -3.685811 | 2.075658 |
| H | 0.301475 | -3.197834 | 3.391558 |
| C | 1.468490 | -0.786720 | 4.014356 |
| H | 2.244060 | -0.136404 | 4.435171 |
| H | 0.816253 | -0.169293 | 3.387083 |
| H | 0.860742 | -1.164448 | 4.844985 |

TS Conformation 125

B3LYP/6-31G(d) Energy = -3411.86873
M06-2X/def2tzvpp/IEFPCM(chloroform) Energy = -3411.8803
M06-2X/def2tzvpp/IEFPCM(chloroform)//B3LYP/6-31G(d) Quasiharmonic Free Energy = -3411.155825

Frequencies (Top 3 out of 291)

1. -974.5463 cm-1
2. 6.9250 cm-1
3. 8.2909 cm-1

B3LYP/6-31G(d) Molecular Geometry in Cartesian Coordinates

| | | | |
|---|---|---|---|
| C | 3.866759 | -2.509396 | 1.409271 |
| C | 3.093288 | -1.402421 | 2.170693 |
| H | 3.894045 | -0.896956 | 2.715155 |
| C | 3.025791 | 0.831270 | 1.045186 |
| N | 2.075305 | 1.728516 | 0.588890 |
| H | 1.114064 | 1.536158 | 0.859220 |
| N | 2.463193 | -0.373765 | 1.350843 |
| H | 1.484530 | -0.497265 | 1.121576 |
| C | 2.372227 | -2.622984 | -0.703229 |
| C | 4.290576 | -4.100175 | -0.366534 |
| C | 2.126257 | -3.977868 | -1.414205 |
| H | 1.498864 | -2.369433 | -0.099686 |
| C | 3.548332 | -4.503751 | -1.648440 |
| H | 5.335942 | -3.823491 | -0.534760 |
| H | 4.289657 | -4.895208 | 0.387175 |
| H | 1.541594 | -3.864779 | -2.330372 |
| H | 1.575914 | -4.642548 | -0.737397 |
| H | 3.990674 | -4.012362 | -2.521816 |
| H | 3.582814 | -5.583662 | -1.819468 |
| N | 3.524314 | -2.945686 | 0.157459 |
| O | 4.817257 | -3.026610 | 1.995205 |
| S | 4.652947 | 1.175287 | 1.246325 |
| C | 2.630542 | -1.504094 | -1.712750 |
| C | 1.554480 | -1.034552 | -2.482496 |
| C | 3.902290 | -0.970473 | -1.940478 |
| C | 1.750827 | -0.064348 | -3.464796 |
| H | 0.557931 | -1.434945 | -2.304139 |
| C | 4.099746 | 0.001155 | -2.926199 |
| C | 3.027847 | 0.455738 | -3.693612 |

| | | | |
|---|---|---|---|
| H | 0.907947 | 0.280850 | -4.059636 |
| O | -4.442552 | 1.664134 | -1.550027 |
| O | -0.987702 | 1.622981 | 1.081662 |
| C | -4.538970 | 0.962693 | -0.545287 |
| C | -3.439913 | 0.708082 | 0.414444 |
| O | -5.648104 | 0.282648 | -0.223035 |
| C | -6.716444 | 0.324377 | -1.189683 |
| H | -6.947441 | 1.355517 | -1.464943 |
| H | -6.430864 | -0.242087 | -2.079290 |
| H | -7.566321 | -0.146090 | -0.695561 |
| S | -2.079270 | 1.843626 | 0.095138 |
| C | -2.625338 | 3.571804 | 0.145222 |
| H | -2.967486 | 3.751389 | 1.166972 |
| H | -1.775216 | 4.215382 | -0.095473 |
| H | -3.438090 | 3.679879 | -0.574771 |
| C | -1.466087 | 1.594392 | -1.581529 |
| H | -0.578333 | 2.224864 | -1.676628 |
| H | -1.213358 | 0.530361 | -1.643025 |
| H | -2.257734 | 1.855178 | -2.282392 |
| C | -3.736538 | 0.632506 | 1.888190 |
| C | -3.022190 | -0.256932 | 2.704877 |
| C | -4.710232 | 1.458598 | 2.477729 |
| C | -3.277575 | -0.318890 | 4.074996 |
| H | -2.282855 | -0.913819 | 2.260465 |
| C | -4.968707 | 1.390011 | 3.844791 |
| H | -5.285649 | 2.141813 | 1.858228 |
| C | -4.248271 | 0.502775 | 4.648254 |
| H | -2.718286 | -1.015346 | 4.693224 |
| H | -5.730470 | 2.029123 | 4.282784 |
| H | -4.447150 | 0.450604 | 5.715177 |
| C | -2.783897 | -2.859222 | -2.914694 |
| C | -3.712939 | -3.406844 | -3.799895 |
| C | -5.030168 | -3.630379 | -3.394668 |
| C | -5.404810 | -3.307653 | -2.088311 |
| C | -4.475665 | -2.770233 | -1.195739 |
| C | -3.150045 | -2.530838 | -1.598426 |
| H | -1.762304 | -2.684687 | -3.240567 |
| H | -3.404831 | -3.656139 | -4.812565 |
| H | -5.753234 | -4.056355 | -4.085031 |
| H | -6.423620 | -3.490411 | -1.753593 |
| H | -4.772172 | -2.539790 | -0.177020 |
| S | -1.945852 | -1.856364 | -0.462993 |
| H | -2.809684 | -0.493431 | 0.020621 |
| H | 4.739232 | -1.300807 | -1.334041 |
| H | 5.096450 | 0.402964 | -3.088772 |
| H | 3.183939 | 1.209145 | -4.461152 |
| C | 2.090849 | -1.971895 | 3.244327 |
| C | 2.232325 | 2.973671 | -0.035748 |
| C | 3.290368 | 3.288691 | -0.899973 |
| C | 1.201059 | 3.908605 | 0.167980 |
| C | 3.276310 | 4.524327 | -1.532534 |
| H | 4.096862 | 2.594244 | -1.083353 |
| C | 1.231051 | 5.110971 | -0.519954 |
| H | 0.409744 | 3.707824 | 0.879296 |
| C | 2.258393 | 5.464301 | -1.381952 |
| H | 2.273532 | 6.411497 | -1.906249 |
| F | 4.289344 | 4.822437 | -2.368921 |
| F | 0.193420 | 5.967803 | -0.339865 |
| C | 1.468103 | -0.787160 | 4.014426 |
| H | 0.815974 | -0.169612 | 3.387162 |
| H | 0.860248 | -1.164937 | 4.844955 |
| H | 2.243683 | -0.136949 | 4.435389 |

| | | | |
|---|---|---|---|
| C | 2.861032 | -2.845851 | 4.256243 |
| H | 3.661016 | -2.276574 | 4.741657 |
| H | 3.321911 | -3.715256 | 3.783906 |
| H | 2.170369 | -3.194949 | 5.033478 |
| C | 0.969732 | -2.818531 | 2.609857 |
| H | 0.300970 | -3.198118 | 3.391249 |
| H | 1.372527 | -3.686023 | 2.075391 |
| H | 0.341348 | -2.253259 | 1.909255 |

TS Conformation 126

B3LYP/6-31G(d) Energy = -3411.872665
M06-2X/def2tzvpp/IEFPCM(chloroform) Energy = -3411.880709
M06-2X/def2tzvpp/IEFPCM(chloroform)//B3LYP/6-31G(d) Quasiharmonic Free Energy = -3411.155547

Frequencies (Top 3 out of 291)

1. -809.2096 cm-1
2. 6.9169 cm-1
3. 8.2628 cm-1

B3LYP/6-31G(d) Molecular Geometry in Cartesian Coordinates

| | | | |
|---|---|---|---|
| C | 2.678474 | -1.236674 | 0.308581 |
| C | 3.048215 | 0.054607 | 1.069197 |
| H | 3.736814 | 0.675208 | 0.499409 |
| C | 1.553401 | 2.072009 | 0.756453 |
| N | 0.228937 | 2.416194 | 0.952705 |
| H | -0.356291 | 1.643503 | 1.260375 |
| N | 1.787797 | 0.797065 | 1.166527 |
| H | 0.990642 | 0.210572 | 1.403197 |
| C | 3.920434 | -0.444526 | -1.771230 |
| C | 2.589401 | -2.507914 | -1.777524 |
| C | 3.727056 | -0.975488 | -3.220182 |
| H | 3.438038 | 0.534516 | -1.677514 |
| C | 2.471731 | -1.855060 | -3.156174 |
| H | 3.262045 | -3.376421 | -1.797664 |
| H | 1.640026 | -2.823488 | -1.345348 |
| H | 4.595548 | -1.579571 | -3.505415 |
| H | 3.655325 | -0.149234 | -3.933915 |
| H | 2.424452 | -2.588496 | -3.967295 |
| H | 1.560936 | -1.246850 | -3.190570 |
| N | 3.167316 | -1.432469 | -0.948066 |
| O | 1.897733 | -2.035113 | 0.835641 |
| S | 2.737383 | 3.080163 | 0.102783 |
| C | 5.387872 | -0.298195 | -1.399931 |
| C | 5.962177 | 0.974946 | -1.310576 |
| C | 6.201805 | -1.421908 | -1.195905 |
| C | 7.322718 | 1.124723 | -1.028739 |
| H | 5.336728 | 1.853747 | -1.446119 |
| C | 7.558517 | -1.274368 | -0.912236 |
| C | 8.124536 | 0.001258 | -0.829960 |
| H | 7.750717 | 2.121159 | -0.958212 |
| O | -1.393726 | 0.000008 | 0.914759 |
| O | -0.823865 | -0.944038 | -1.877769 |
| C | -2.473596 | -0.578315 | 0.792702 |
| C | -3.220759 | -0.767865 | -0.484107 |
| O | -3.136279 | -1.140835 | 1.799925 |
| C | -2.461126 | -1.189231 | 3.073012 |
| H | -2.194110 | -0.183538 | 3.407737 |
| H | -3.177336 | -1.644495 | 3.755540 |

| | | | |
|---|---|---|---|
| H | -1.566463 | -1.809947 | 2.986271 |
| S | -2.163040 | -0.322106 | -1.873122 |
| C | -3.116157 | -0.873215 | -3.301181 |
| H | -4.093077 | -0.388217 | -3.318807 |
| H | -3.211055 | -1.958340 | -3.179785 |
| H | -2.524292 | -0.627607 | -4.185508 |
| C | -2.002137 | 1.468855 | -2.122965 |
| H | -1.470647 | 1.612541 | -3.067008 |
| H | -1.409593 | 1.845903 | -1.288841 |
| H | -2.991364 | 1.928362 | -2.144375 |
| C | -4.597142 | -0.133320 | -0.541695 |
| C | -5.710977 | -0.872363 | -0.969480 |
| C | -4.785725 | 1.200404 | -0.137161 |
| C | -6.974830 | -0.279969 | -1.006473 |
| H | -5.589227 | -1.919423 | -1.229663 |
| C | -6.048310 | 1.789133 | -0.172428 |
| H | -3.943272 | 1.785514 | 0.219360 |
| C | -7.147054 | 1.048973 | -0.614702 |
| H | -7.829544 | -0.866458 | -1.332017 |
| H | -6.162901 | 2.819364 | 0.152242 |
| H | -8.134113 | 1.502419 | -0.642792 |
| C | -3.029876 | -5.395175 | 1.050471 |
| C | -2.221564 | -5.887808 | 2.074737 |
| C | -0.938149 | -5.372907 | 2.276380 |
| C | -0.466145 | -4.364608 | 1.432642 |
| C | -1.268692 | -3.879828 | 0.396208 |
| C | -2.567439 | -4.380916 | 0.193055 |
| H | -4.031514 | -5.790004 | 0.906511 |
| H | -2.600345 | -6.675737 | 2.722478 |
| H | -0.311210 | -5.758619 | 3.076580 |
| H | 0.531438 | -3.952691 | 1.555495 |
| H | -0.874660 | -3.113622 | -0.265285 |
| S | -3.622705 | -3.763302 | -1.107052 |
| H | -3.370219 | -2.091170 | -0.724804 |
| H | 5.766833 | -2.416988 | -1.246555 |
| H | 8.174988 | -2.155161 | -0.752297 |
| H | 9.181968 | 0.115737 | -0.607113 |
| C | 3.698650 | -0.207209 | 2.477027 |
| C | -0.512155 | 3.586960 | 0.742481 |
| C | -0.028158 | 4.784483 | 0.194391 |
| C | -1.872998 | 3.499967 | 1.110937 |
| C | -0.918315 | 5.839120 | 0.026444 |
| H | 1.006951 | 4.893176 | -0.092845 |
| C | -2.708440 | 4.582946 | 0.898467 |
| H | -2.269291 | 2.603732 | 1.574717 |
| C | -2.268892 | 5.782473 | 0.353323 |
| H | -2.933006 | 6.623209 | 0.197268 |
| F | -0.444676 | 6.983541 | -0.502227 |
| F | -4.015731 | 4.453545 | 1.225933 |
| C | 4.852383 | -1.216363 | 2.318240 |
| H | 5.591406 | -0.876642 | 1.584964 |
| H | 5.368194 | -1.342362 | 3.277489 |
| H | 4.485813 | -2.200806 | 2.007688 |
| C | 2.685354 | -0.750744 | 3.503401 |
| H | 2.236640 | -1.687690 | 3.167140 |
| H | 3.193604 | -0.927370 | 4.458923 |
| H | 1.882757 | -0.027746 | 3.691869 |
| C | 4.267196 | 1.128933 | 2.994972 |
| H | 5.042396 | 1.521294 | 2.327638 |
| H | 3.487301 | 1.892341 | 3.083219 |
| H | 4.712502 | 0.985930 | 3.987084 |

TS Conformation 127

B3LYP/6-31G(d) Energy = -3411.863353
M06-2X/def2tzvpp/IEFPCM(chloroform) Energy = -3411.879087
M06-2X/def2tzvpp/IEFPCM(chloroform)//B3LYP/6-31G(d) Quasiharmonic Free Energy = -3411.155422

Frequencies (Top 3 out of 291)

1. -852.5862 cm-1
2. 6.4004 cm-1
3. 7.1135 cm-1

B3LYP/6-31G(d) Molecular Geometry in Cartesian Coordinates

| | | | |
|---|---|---|---|
| C | -4.543516 | -2.014188 | -1.071623 |
| C | -3.323303 | -1.584902 | -1.924486 |
| H | -3.791342 | -1.085482 | -2.776208 |
| C | -2.423754 | 0.734000 | -1.665006 |
| N | -1.304679 | 1.375659 | -1.182389 |
| H | -0.552677 | 0.783871 | -0.844434 |
| N | -2.433667 | -0.594949 | -1.331989 |
| H | -1.640117 | -0.936413 | -0.802209 |
| C | -3.553583 | -1.548460 | 1.280101 |
| C | -5.764105 | -2.540366 | 0.955204 |
| C | -3.921569 | -2.428397 | 2.502447 |
| H | -2.551044 | -1.806314 | 0.931921 |
| C | -5.454946 | -2.456823 | 2.456862 |
| H | -6.662207 | -1.987719 | 0.663638 |
| H | -5.901966 | -3.571713 | 0.610863 |
| H | -3.517875 | -2.028230 | 3.436509 |
| H | -3.518393 | -3.438892 | 2.354960 |
| H | -5.857477 | -1.531569 | 2.882172 |
| H | -5.884608 | -3.295075 | 3.012983 |
| N | -4.565720 | -1.971081 | 0.298352 |
| O | -5.522181 | -2.456511 | -1.673063 |
| S | -3.629849 | 1.466508 | -2.574471 |
| C | -3.593008 | -0.061891 | 1.630581 |
| C | -4.671532 | 0.760113 | 1.289710 |
| C | -2.536732 | 0.487446 | 2.371374 |
| C | -4.704713 | 2.095693 | 1.699262 |
| H | -5.479793 | 0.363387 | 0.683999 |
| C | -2.565920 | 1.820208 | 2.778115 |
| C | -3.656377 | 2.628987 | 2.448482 |
| H | -5.549923 | 2.719591 | 1.421514 |
| O | 4.165272 | -2.870337 | 1.248876 |
| O | 0.297071 | -1.156071 | 0.299255 |
| C | 4.030325 | -1.685874 | 1.548759 |
| C | 2.853799 | -0.855226 | 1.185774 |
| O | 4.959503 | -0.972065 | 2.193218 |
| C | 6.217761 | -1.638080 | 2.426135 |
| H | 6.781577 | -0.962966 | 3.069415 |
| H | 6.058472 | -2.601146 | 2.915512 |
| H | 6.735666 | -1.784102 | 1.475275 |
| S | 1.536090 | -1.934256 | 0.573641 |
| C | 1.153052 | -3.266777 | 1.739366 |
| H | 0.382778 | -3.892648 | 1.282148 |
| H | 2.072753 | -3.819582 | 1.933719 |
| H | 0.771260 | -2.784183 | 2.641623 |
| C | 2.109944 | -2.761127 | -0.922374 |
| H | 2.946677 | -3.406711 | -0.658271 |
| H | 1.252248 | -3.306970 | -1.321990 |

| | | | |
|---|---|---|---|
| H | 2.427365 | -1.949003 | -1.590623 |
| C | 2.323584 | 0.149209 | 2.173273 |
| C | 2.258162 | -0.145380 | 3.546967 |
| C | 1.883208 | 1.403200 | 1.726961 |
| C | 1.772061 | 0.795281 | 4.451916 |
| H | 2.617279 | -1.104664 | 3.912489 |
| C | 1.393412 | 2.343513 | 2.634187 |
| H | 1.953287 | 1.654349 | 0.674491 |
| C | 1.336894 | 2.042973 | 3.995713 |
| H | 1.737791 | 0.558386 | 5.511727 |
| H | 1.062315 | 3.311935 | 2.270961 |
| H | 0.963833 | 2.779470 | 4.702330 |
| C | 6.063816 | 0.455879 | -0.740856 |
| C | 7.410833 | 0.119873 | -0.890089 |
| C | 7.845366 | -0.590679 | -2.011479 |
| C | 6.914771 | -0.960775 | -2.984609 |
| C | 5.566537 | -0.634888 | -2.835238 |
| C | 5.116430 | 0.076998 | -1.709282 |
| H | 5.738762 | 1.027211 | 0.123562 |
| H | 8.127210 | 0.432924 | -0.133356 |
| H | 8.895522 | -0.844045 | -2.129612 |
| H | 7.238629 | -1.507703 | -3.867115 |
| H | 4.849188 | -0.924116 | -3.598112 |
| S | 3.391997 | 0.505562 | -1.538042 |
| H | 3.143527 | -0.215748 | 0.020236 |
| H | -1.676477 | -0.129909 | 2.623550 |
| H | -1.731936 | 2.227007 | 3.343039 |
| H | -3.680997 | 3.669140 | 2.761777 |
| C | -2.525345 | -2.821921 | -2.489572 |
| C | -1.000724 | 2.755784 | -1.177726 |
| C | -1.962934 | 3.726739 | -0.872074 |
| C | 0.332020 | 3.128304 | -1.412114 |
| C | -1.563852 | 5.054155 | -0.824396 |
| H | -2.990739 | 3.460316 | -0.673434 |
| C | 0.670657 | 4.474285 | -1.333854 |
| H | 1.093567 | 2.403461 | -1.683765 |
| C | -0.254658 | 5.469225 | -1.044311 |
| H | 0.027434 | 6.513942 | -1.000159 |
| F | -2.487331 | 5.991192 | -0.523365 |
| F | 1.948950 | 4.832962 | -1.560804 |
| C | -1.975623 | -3.709738 | -1.353641 |
| H | -1.307479 | -3.151910 | -0.684359 |
| H | -2.783536 | -4.134445 | -0.747149 |
| H | -1.403469 | -4.547207 | -1.772736 |
| C | -1.357231 | -2.302469 | -3.354136 |
| H | -0.609919 | -1.754602 | -2.770821 |
| H | -1.718320 | -1.629327 | -4.139296 |
| H | -0.851910 | -3.144794 | -3.842093 |
| C | -3.437437 | -3.679603 | -3.390782 |
| H | -2.856430 | -4.508433 | -3.814392 |
| H | -3.835395 | -3.086405 | -4.220974 |
| H | -4.288964 | -4.088720 | -2.845185 |

TS Conformation 128

B3LYP/6-31G(d) Energy = -3411.868268
M06-2X/def2tzvpp/IEFPCM(chloroform) Energy = -3411.880876
M06-2X/def2tzvpp/IEFPCM(chloroform)//B3LYP/6-31G(d) Quasiharmonic Free Energy = -3411.155355

Frequencies (Top 3 out of 291)

1. -921.6650 cm-1
2. 8.3176 cm-1
3. 12.0399 cm-1

B3LYP/6-31G(d) Molecular Geometry in Cartesian Coordinates

| | | | |
|---|---|---|---|
| C | -5.112175 | -0.211838 | -0.669457 |
| C | -3.969033 | 0.321302 | -1.571150 |
| H | -4.385927 | 1.254852 | -1.953898 |
| C | -2.318859 | 2.004003 | -0.738530 |
| N | -1.093733 | 2.054093 | -0.093576 |
| H | -0.880049 | 1.229103 | 0.459735 |
| N | -2.733283 | 0.706765 | -0.898648 |
| H | -2.018699 | -0.004659 | -0.785889 |
| C | -3.643911 | -1.301855 | 1.165188 |
| C | -6.051665 | -1.597383 | 1.110279 |
| C | -4.054094 | -2.540668 | 2.035040 |
| H | -2.906528 | -1.618995 | 0.421969 |
| C | -5.456999 | -2.935346 | 1.551611 |
| H | -6.423576 | -1.022356 | 1.970146 |
| H | -6.856429 | -1.670128 | 0.379869 |
| H | -4.087638 | -2.244993 | 3.088182 |
| H | -3.320893 | -3.348318 | 1.946855 |
| H | -6.048024 | -3.427421 | 2.330409 |
| H | -5.399033 | -3.613405 | 0.691728 |
| N | -4.898973 | -0.919830 | 0.490722 |
| O | -6.259408 | -0.018727 | -1.068915 |
| S | -3.209914 | 3.336432 | -1.235141 |
| C | -3.032014 | -0.216045 | 2.043559 |
| C | -3.775523 | 0.874254 | 2.507908 |
| C | -1.703185 | -0.355917 | 2.469082 |
| C | -3.206793 | 1.799012 | 3.385790 |
| H | -4.797085 | 1.010655 | 2.166595 |
| C | -1.135541 | 0.565500 | 3.352049 |
| C | -1.888171 | 1.645940 | 3.816638 |
| H | -3.795941 | 2.646098 | 3.726252 |
| O | 1.678182 | -0.323875 | 1.550954 |
| O | -0.188246 | -1.293481 | -0.836918 |
| C | 2.316654 | -1.350917 | 1.354094 |
| C | 2.406146 | -2.069087 | 0.052098 |
| O | 3.072206 | -1.948961 | 2.284599 |
| C | 3.097876 | -1.317475 | 3.579349 |
| H | 3.807416 | -1.899674 | 4.166403 |
| H | 2.105321 | -1.349253 | 4.036458 |
| H | 3.425817 | -0.279265 | 3.492518 |
| S | 1.280662 | -1.305216 | -1.109955 |
| C | 1.878571 | 0.363444 | -1.444001 |
| H | 1.208553 | 0.777543 | -2.201010 |
| H | 2.910564 | 0.295781 | -1.795387 |
| H | 1.817044 | 0.928539 | -0.514734 |
| C | 1.630846 | -2.177836 | -2.647689 |
| H | 1.055559 | -1.678453 | -3.430145 |
| H | 1.303809 | -3.210614 | -2.522647 |
| H | 2.711289 | -2.116871 | -2.823408 |
| C | 2.290205 | -3.579974 | 0.090409 |
| C | 3.457415 | -4.355652 | 0.025164 |
| C | 1.049774 | -4.225698 | 0.235183 |
| C | 3.381694 | -5.747120 | 0.096248 |
| H | 4.421471 | -3.866530 | -0.068005 |
| C | 0.978952 | -5.618002 | 0.287200 |
| H | 0.138429 | -3.638623 | 0.306711 |
| C | 2.145269 | -6.382309 | 0.219671 |

| | | | |
|---|---|---|---|
| H | 4.294455 | -6.334530 | 0.049913 |
| H | 0.012570 | -6.103329 | 0.393464 |
| H | 2.090161 | -7.466346 | 0.268659 |
| C | 5.128851 | 0.623584 | 0.307140 |
| C | 5.243315 | 1.983384 | 0.600882 |
| C | 5.581933 | 2.896133 | -0.400347 |
| C | 5.805501 | 2.433233 | -1.698949 |
| C | 5.666875 | 1.078183 | -2.001628 |
| C | 5.318678 | 0.148731 | -1.003812 |
| H | 4.901399 | -0.085374 | 1.098305 |
| H | 5.056432 | 2.333284 | 1.612545 |
| H | 5.672773 | 3.953989 | -0.170360 |
| H | 6.078751 | 3.131846 | -2.486280 |
| H | 5.825669 | 0.727067 | -3.017515 |
| S | 5.092708 | -1.573922 | -1.417758 |
| H | 3.626343 | -1.799268 | -0.541281 |
| H | -1.102214 | -1.185290 | 2.103147 |
| H | -0.100751 | 0.442204 | 3.658484 |
| H | -1.446591 | 2.369043 | 4.496577 |
| C | -3.712383 | -0.604751 | -2.824190 |
| C | -0.193201 | 3.112923 | 0.125805 |
| C | 0.800700 | 2.889425 | 1.099823 |
| C | -0.181406 | 4.307262 | -0.610341 |
| C | 1.766000 | 3.861225 | 1.314315 |
| H | 0.831360 | 1.975366 | 1.680846 |
| C | 0.808980 | 5.241718 | -0.336307 |
| H | -0.928113 | 4.520100 | -1.359839 |
| C | 1.804319 | 5.061286 | 0.616783 |
| H | 2.562734 | 5.811248 | 0.802723 |
| F | 2.713001 | 3.627629 | 2.250364 |
| F | 0.810537 | 6.387194 | -1.046817 |
| C | -4.987163 | -0.669353 | -3.691208 |
| H | -5.295438 | 0.330912 | -4.014023 |
| H | -4.787644 | -1.269653 | -4.587470 |
| H | -5.828099 | -1.112551 | -3.154967 |
| C | -3.309055 | -2.038071 | -2.423413 |
| H | -4.092080 | -2.525410 | -1.831424 |
| H | -3.154660 | -2.646187 | -3.323176 |
| H | -2.373568 | -2.066086 | -1.852855 |
| C | -2.589869 | 0.025682 | -3.675295 |
| H | -2.820483 | 1.066164 | -3.928396 |
| H | -1.624378 | 0.011708 | -3.158066 |
| H | -2.476226 | -0.534442 | -4.611160 |

TS Conformation 129

B3LYP/6-31G(d) Energy = -3411.869241
M06-2X/def2tzvpp/IEFPCM(chloroform) Energy = -3411.879125
M06-2X/def2tzvpp/IEFPCM(chloroform)//B3LYP/6-31G(d) Quasiharmonic Free Energy = -3411.155204

Frequencies (Top 3 out of 291)

1. -819.1912 cm-1
2. 5.1887 cm-1
3. 9.2450 cm-1

B3LYP/6-31G(d) Molecular Geometry in Cartesian Coordinates

| | | | |
|---|---|---|---|
| C | -4.404123 | -0.444637 | -0.893693 |
| C | -3.218841 | -0.135475 | -1.847308 |
| H | -3.705156 | 0.449790 | -2.632052 |

| | | | |
|---|---|---|---|
| C | -2.214281 | 2.049573 | -1.151768 |
| N | -1.035784 | 2.551049 | -0.626102 |
| H | -0.356896 | 1.846570 | -0.351990 |
| N | -2.153563 | 0.695398 | -1.297470 |
| H | -1.279536 | 0.234777 | -1.072622 |
| C | -5.480983 | -0.766940 | 1.261651 |
| C | -3.092869 | -0.200709 | 1.309679 |
| C | -5.067710 | -0.278546 | 2.671242 |
| H | -6.270382 | -0.137158 | 0.842666 |
| C | -3.560741 | -0.558375 | 2.727305 |
| H | -2.197613 | -0.756938 | 1.017823 |
| H | -2.872340 | 0.870788 | 1.241660 |
| H | -5.632351 | -0.779436 | 3.461761 |
| H | -5.252022 | 0.799616 | 2.751005 |
| H | -3.376687 | -1.618020 | 2.936115 |
| H | -3.039104 | 0.029142 | 3.489158 |
| N | -4.249825 | -0.552604 | 0.464522 |
| O | -5.500310 | -0.637614 | -1.413342 |
| S | -3.582993 | 2.941887 | -1.533649 |
| C | -5.981941 | -2.206373 | 1.249323 |
| C | -7.359689 | -2.444877 | 1.330954 |
| C | -5.116791 | -3.306774 | 1.221444 |
| C | -7.861249 | -3.744689 | 1.392304 |
| H | -8.046809 | -1.601517 | 1.335013 |
| C | -5.614251 | -4.610130 | 1.279494 |
| C | -6.988525 | -4.834248 | 1.368288 |
| H | -8.934678 | -3.906494 | 1.448426 |
| O | 5.185078 | 0.083885 | -1.443326 |
| O | 0.939098 | 0.252472 | -0.641904 |
| C | 4.876885 | 0.003273 | -0.256221 |
| C | 3.482306 | -0.144411 | 0.233493 |
| O | 5.763586 | -0.024227 | 0.743510 |
| C | 7.151910 | -0.070749 | 0.352870 |
| H | 7.382519 | 0.739385 | -0.342020 |
| H | 7.711622 | 0.041555 | 1.281080 |
| H | 7.368381 | -1.035009 | -0.112710 |
| S | 2.348266 | 0.238119 | -1.127365 |
| C | 2.715109 | 1.822379 | -1.928422 |
| H | 2.530430 | 2.600663 | -1.183791 |
| H | 2.021002 | 1.929324 | -2.765961 |
| H | 3.755285 | 1.804808 | -2.254277 |
| C | 2.550148 | -1.006574 | -2.414284 |
| H | 1.759532 | -0.827090 | -3.146751 |
| H | 3.552505 | -0.906851 | -2.829773 |
| H | 2.427108 | -1.970730 | -1.901227 |
| C | 3.051837 | 0.539184 | 1.502294 |
| C | 2.177424 | -0.116359 | 2.382329 |
| C | 3.493827 | 1.835131 | 1.820953 |
| C | 1.760450 | 0.511198 | 3.556664 |
| H | 1.849477 | -1.125350 | 2.156059 |
| C | 3.074657 | 2.460954 | 2.993445 |
| H | 4.183267 | 2.351086 | 1.157315 |
| C | 2.203728 | 1.798578 | 3.862856 |
| H | 1.090463 | -0.010373 | 4.234388 |
| H | 3.412730 | 3.467524 | 3.217596 |
| H | 1.874954 | 2.285964 | 4.776567 |
| C | 5.569388 | -3.458736 | 0.889025 |
| C | 6.847420 | -3.948940 | 0.612965 |
| C | 7.068735 | -4.770981 | -0.494604 |
| C | 5.993465 | -5.098824 | -1.323042 |
| C | 4.716661 | -4.604941 | -1.054532 |
| C | 4.480578 | -3.773366 | 0.055053 |

| | | | |
|---|---|---|---|
| H | 5.403214 | -2.836617 | 1.763608 |
| H | 7.671156 | -3.700760 | 1.279183 |
| H | 8.062651 | -5.157636 | -0.703497 |
| H | 6.147936 | -5.743463 | -2.185338 |
| H | 3.884892 | -4.868088 | -1.702112 |
| S | 2.837558 | -3.168566 | 0.404795 |
| H | 3.234830 | -1.470681 | 0.352381 |
| H | -4.045411 | -3.147368 | 1.139701 |
| H | -4.925271 | -5.450575 | 1.252008 |
| H | -7.376711 | -5.848443 | 1.410188 |
| C | -2.617056 | -1.419740 | -2.525097 |
| C | -0.606868 | 3.857370 | -0.343032 |
| C | -1.148934 | 5.016951 | -0.915557 |
| C | 0.489868 | 3.962736 | 0.537710 |
| C | -0.584767 | 6.241626 | -0.580366 |
| H | -1.992184 | 4.972838 | -1.588427 |
| C | 1.010282 | 5.216663 | 0.820233 |
| H | 0.905457 | 3.090487 | 1.031252 |
| C | 0.498717 | 6.388743 | 0.278668 |
| H | 0.913444 | 7.360273 | 0.516366 |
| F | -1.108194 | 7.351737 | -1.134215 |
| F | 2.061419 | 5.301922 | 1.667072 |
| C | -1.899341 | -2.333264 | -1.509719 |
| H | -2.584455 | -2.665409 | -0.723002 |
| H | -1.521364 | -3.229193 | -2.015334 |
| H | -1.033937 | -1.856248 | -1.032368 |
| C | -1.620233 | -0.973088 | -3.617034 |
| H | -2.109784 | -0.332103 | -4.359204 |
| H | -0.772919 | -0.414638 | -3.202602 |
| H | -1.221500 | -1.850355 | -4.139885 |
| C | -3.739572 | -2.226955 | -3.209170 |
| H | -4.457782 | -2.625566 | -2.490047 |
| H | -4.298800 | -1.608767 | -3.918803 |
| H | -3.296679 | -3.066651 | -3.758237 |

TS Conformation 130

B3LYP/6-31G(d) Energy = -3411.86887
M06-2X/def2tzvpp/IEFPCM(chloroform) Energy = -3411.880439
M06-2X/def2tzvpp/IEFPCM(chloroform)//B3LYP/6-31G(d) Quasiharmonic Free Energy = -3411.155164

Frequencies (Top 3 out of 291)

1. -862.2152 cm-1
2. 7.0084 cm-1
3. 8.8510 cm-1

B3LYP/6-31G(d) Molecular Geometry in Cartesian Coordinates

| | | | |
|---|---|---|---|
| C | 4.205059 | -2.575501 | 1.144681 |
| C | 3.220030 | -1.665851 | 1.922881 |
| H | 3.899250 | -1.149581 | 2.605161 |
| C | 3.038640 | 0.658259 | 1.034301 |
| N | 2.059380 | 1.524669 | 0.591901 |
| H | 1.111970 | 1.158510 | 0.627771 |
| N | 2.564860 | -0.620621 | 1.142401 |
| H | 1.598100 | -0.775681 | 0.878551 |
| C | 2.892289 | -2.757141 | -1.076199 |
| C | 5.039259 | -3.860111 | -0.733699 |
| C | 2.908289 | -4.115331 | -1.821609 |
| H | 1.979079 | -2.659171 | -0.485999 |

| | | | |
|---|---|---|---|
| C | 4.407749 | -4.354381 | -2.043079 |
| H | 6.011219 | -3.377172 | -0.874159 |
| H | 5.184129 | -4.665851 | -0.007459 |
| H | 2.329379 | -4.104161 | -2.748579 |
| H | 2.489029 | -4.888481 | -1.166109 |
| H | 4.758329 | -3.760101 | -2.893979 |
| H | 4.648739 | -5.401961 | -2.245029 |
| N | 4.053509 | -2.899101 | -0.183099 |
| O | 5.168889 | -3.014781 | 1.767081 |
| S | 4.613260 | 1.089659 | 1.423161 |
| C | 2.982150 | -1.575571 | -2.047839 |
| C | 4.017090 | -0.637751 | -1.993839 |
| C | 1.998740 | -1.426251 | -3.039249 |
| C | 4.067020 | 0.423859 | -2.901769 |
| H | 4.777990 | -0.727301 | -1.225909 |
| C | 2.047190 | -0.369281 | -3.950499 |
| C | 3.085510 | 0.564259 | -3.882379 |
| H | 4.879830 | 1.141929 | -2.837689 |
| O | -3.951040 | 0.619831 | -2.257499 |
| O | -0.468590 | -0.082480 | 0.217131 |
| C | -4.078840 | 0.929611 | -1.074869 |
| C | -3.084030 | 0.618881 | -0.017559 |
| O | -5.164390 | 1.533021 | -0.578249 |
| C | -6.260419 | 1.712851 | -1.498549 |
| H | -6.986719 | 2.320801 | -0.959589 |
| H | -5.922379 | 2.221031 | -2.404079 |
| H | -6.687340 | 0.740281 | -1.754289 |
| S | -1.534990 | 0.110980 | -0.806659 |
| C | -1.822730 | -1.428440 | -1.695209 |
| H | -2.537070 | -1.231580 | -2.494089 |
| H | -0.847120 | -1.753110 | -2.062919 |
| H | -2.241770 | -2.117180 | -0.949049 |
| C | -0.955590 | 1.284910 | -2.057969 |
| H | -0.047880 | 0.863070 | -2.500159 |
| H | -1.753520 | 1.417960 | -2.789049 |
| H | -0.724199 | 2.214990 | -1.532689 |
| C | -2.827480 | 1.605090 | 1.088671 |
| C | -2.818519 | 2.989900 | 0.845801 |
| C | -2.569510 | 1.142060 | 2.388111 |
| C | -2.562309 | 3.891210 | 1.877491 |
| H | -3.027689 | 3.366481 | -0.152199 |
| C | -2.312549 | 2.046040 | 3.419501 |
| H | -2.594600 | 0.076780 | 2.590791 |
| C | -2.305639 | 3.418650 | 3.167431 |
| H | -2.542629 | 4.956370 | 1.668971 |
| H | -2.119599 | 1.674760 | 4.421941 |
| H | -2.101399 | 4.119840 | 3.971811 |
| C | -6.413850 | -1.483009 | 0.506331 |
| C | -7.628580 | -1.687618 | -0.151129 |
| C | -7.809191 | -2.797088 | -0.980539 |
| C | -6.758131 | -3.701949 | -1.143209 |
| C | -5.540401 | -3.496569 | -0.494159 |
| C | -5.344030 | -2.381999 | 0.341031 |
| H | -6.289450 | -0.628349 | 1.164751 |
| H | -8.444590 | -0.984138 | 0.001281 |
| H | -8.757971 | -2.958628 | -1.485269 |
| H | -6.885171 | -4.574449 | -1.780119 |
| H | -4.728821 | -4.207279 | -0.623569 |
| S | -3.791300 | -2.143879 | 1.189251 |
| H | -3.442560 | -0.573719 | 0.538651 |
| H | 1.200760 | -2.162510 | -3.122189 |
| H | 1.289370 | -0.288331 | -4.726759 |

| | | | |
|---|---|---|---|
| H | 3.131380 | 1.385759 | -4.592039 |
| C | 2.191280 | -2.448681 | 2.821571 |
| C | 2.118961 | 2.879579 | 0.228781 |
| C | 3.248341 | 3.494229 | -0.329609 |
| C | 0.928851 | 3.615840 | 0.383621 |
| C | 3.152931 | 4.827299 | -0.705939 |
| H | 4.178701 | 2.960949 | -0.454679 |
| C | 0.898131 | 4.942060 | -0.023279 |
| H | 0.056031 | 3.183320 | 0.860351 |
| C | 1.995421 | 5.588559 | -0.575999 |
| H | 1.957302 | 6.627139 | -0.879869 |
| F | 4.238651 | 5.413469 | -1.247289 |
| F | -0.251849 | 5.636500 | 0.133951 |
| C | 1.427390 | -1.424271 | 3.688921 |
| H | 2.120400 | -0.802741 | 4.267551 |
| H | 0.775370 | -1.950190 | 4.395551 |
| H | 0.793620 | -0.758960 | 3.093061 |
| C | 2.954029 | -3.402591 | 3.764091 |
| H | 3.495249 | -4.178681 | 3.218461 |
| H | 3.687359 | -2.860231 | 4.369671 |
| H | 2.241669 | -3.888281 | 4.441501 |
| C | 1.178849 | -3.271180 | 1.999491 |
| H | 0.491539 | -3.795480 | 2.672901 |
| H | 1.678529 | -4.030871 | 1.387111 |
| H | 0.553779 | -2.648530 | 1.346521 |

TS Conformation 131

B3LYP/6-31G(d) Energy = -3411.864532
M06-2X/def2tzvpp/IEFPCM(chloroform) Energy = -3411.87955
M06-2X/def2tzvpp/IEFPCM(chloroform)//B3LYP/6-31G(d) Quasiharmonic Free Energy = -3411.15499

Frequencies (Top 3 out of 291)

1. -968.7612 cm-1
2. 6.1575 cm-1
3. 10.1151 cm-1

B3LYP/6-31G(d) Molecular Geometry in Cartesian Coordinates

| | | | |
|---|---|---|---|
| C | 2.833973 | -3.696033 | 0.515571 |
| C | 2.449049 | -2.629912 | 1.577502 |
| H | 3.374445 | -2.546960 | 2.151157 |
| C | 3.097507 | -0.266883 | 1.078534 |
| N | 2.507005 | 0.948006 | 0.804214 |
| H | 1.492965 | 0.957841 | 0.851870 |
| N | 2.181339 | -1.284592 | 1.082987 |
| H | 1.218793 | -1.042899 | 0.880658 |
| C | 1.335316 | -2.836927 | -1.413538 |
| C | 2.674110 | -4.814237 | -1.652513 |
| C | 0.593484 | -3.835262 | -2.374862 |
| H | 0.627917 | -2.493483 | -0.656822 |
| C | 1.290158 | -5.196451 | -2.183053 |
| H | 3.339600 | -4.499653 | -2.467929 |
| H | 3.174425 | -5.584994 | -1.069024 |
| H | 0.698486 | -3.491857 | -3.407431 |
| H | -0.476371 | -3.869738 | -2.150855 |
| H | 1.336457 | -5.778220 | -3.109154 |
| H | 0.767716 | -5.802059 | -1.433117 |
| N | 2.379514 | -3.665010 | -0.780405 |
| O | 3.579589 | -4.595589 | 0.900302 |

| | | | |
|---|---|---|---|
| S | 4.728407 | -0.511158 | 1.402433 |
| C | 1.875125 | -1.625372 | -2.175238 |
| C | 0.971218 | -0.823475 | -2.890913 |
| C | 3.234789 | -1.304768 | -2.215085 |
| C | 1.423202 | 0.265030 | -3.637848 |
| H | -0.091087 | -1.059632 | -2.873267 |
| C | 3.686412 | -0.210252 | -2.958057 |
| C | 2.785405 | 0.576132 | -3.675087 |
| H | 0.709834 | 0.861733 | -4.201589 |
| O | -4.798130 | 1.439809 | 1.828842 |
| O | -0.509995 | 0.840942 | 1.608377 |
| C | -4.282887 | 1.727210 | 0.750017 |
| C | -2.855543 | 1.515414 | 0.409998 |
| O | -4.970041 | 2.225315 | -0.286200 |
| C | -6.395503 | 2.331288 | -0.099326 |
| H | -6.624307 | 2.906998 | 0.800197 |
| H | -6.761818 | 2.841362 | -0.989992 |
| H | -6.832652 | 1.333062 | -0.019323 |
| S | -1.956396 | 1.030425 | 1.894192 |
| C | -2.143005 | 2.237143 | 3.232423 |
| H | -3.207298 | 2.339782 | 3.447814 |
| H | -1.577583 | 1.868288 | 4.091609 |
| H | -1.714193 | 3.171759 | 2.864044 |
| C | -2.665775 | -0.497604 | 2.537427 |
| H | -3.689280 | -0.300126 | 2.852150 |
| H | -2.631717 | -1.203660 | 1.700683 |
| H | -2.015338 | -0.817368 | 3.354786 |
| C | -2.106808 | 2.585302 | -0.337127 |
| C | -2.376163 | 3.948258 | -0.113193 |
| C | -1.099075 | 2.237223 | -1.248739 |
| C | -1.660231 | 4.934658 | -0.787007 |
| H | -3.164175 | 4.236970 | 0.578067 |
| C | -0.377582 | 3.227733 | -1.916205 |
| H | -0.879896 | 1.192823 | -1.441199 |
| C | -0.653458 | 4.575691 | -1.686553 |
| H | -1.881120 | 5.982423 | -0.603967 |
| H | 0.408659 | 2.937323 | -2.606156 |
| H | -0.083580 | 5.344998 | -2.199106 |
| C | -5.262950 | -0.962049 | -1.611603 |
| C | -6.593373 | -1.382858 | -1.569773 |
| C | -6.942967 | -2.567797 | -0.917764 |
| C | -5.944762 | -3.329811 | -0.308045 |
| C | -4.614639 | -2.908637 | -0.340074 |
| C | -4.251614 | -1.716086 | -0.989603 |
| H | -4.995835 | -0.050203 | -2.137291 |
| H | -7.358488 | -0.789461 | -2.065781 |
| H | -7.978298 | -2.896767 | -0.892810 |
| H | -6.200683 | -4.258396 | 0.196806 |
| H | -3.842752 | -3.509367 | 0.133290 |
| S | -2.543279 | -1.193960 | -1.045635 |
| H | -2.752693 | 0.306787 | -0.305583 |
| H | 3.944015 | -1.905436 | -1.654962 |
| H | 4.747441 | 0.023881 | -2.972527 |
| H | 3.139794 | 1.421326 | -4.259338 |
| C | 1.337858 | -3.123105 | 2.578730 |
| C | 3.045581 | 2.220366 | 0.547782 |
| C | 4.373380 | 2.466256 | 0.169422 |
| C | 2.140019 | 3.294703 | 0.636536 |
| C | 4.749127 | 3.775437 | -0.100493 |
| H | 5.097512 | 1.669506 | 0.096269 |
| C | 2.577538 | 4.575043 | 0.341690 |
| H | 1.105220 | 3.135794 | 0.916627 |

| | | | |
|---|---|---|---|
| C | 3.884955 | 4.862125 | -0.028919 |
| H | 4.212662 | 5.870544 | -0.248328 |
| F | 6.026945 | 4.005096 | -0.465489 |
| F | 1.686949 | 5.590622 | 0.416453 |
| C | 1.807199 | -4.411505 | 3.286227 |
| H | 2.756067 | -4.252186 | 3.809083 |
| H | 1.055683 | -4.714144 | 4.026105 |
| H | 1.957355 | -5.234966 | 2.585866 |
| C | -0.003056 | -3.408175 | 1.874410 |
| H | 0.095760 | -4.205575 | 1.129771 |
| H | -0.419316 | -2.528929 | 1.367241 |
| H | -0.748199 | -3.739483 | 2.608494 |
| C | 1.136901 | -2.035826 | 3.656427 |
| H | 0.741257 | -1.103324 | 3.240156 |
| H | 2.081738 | -1.799697 | 4.158950 |
| H | 0.434236 | -2.395527 | 4.418748 |

TS Conformation 132

B3LYP/6-31G(d) Energy = -3411.864454
M06-2X/def2tzvpp/IEFPCM(chloroform) Energy = -3411.879287
M06-2X/def2tzvpp/IEFPCM(chloroform)//B3LYP/6-31G(d) Quasiharmonic Free Energy = -3411.154989

Frequencies (Top 3 out of 291)

1. -716.6510 cm-1
2. 9.7539 cm-1
3. 11.4026 cm-1

B3LYP/6-31G(d) Molecular Geometry in Cartesian Coordinates

| | | | |
|---|---|---|---|
| C | -3.589142 | 0.955778 | -1.149561 |
| C | -2.411653 | 0.919860 | -2.161170 |
| H | -2.680121 | 1.721244 | -2.853800 |
| C | -0.654472 | 2.530661 | -1.411221 |
| N | 0.655123 | 2.546624 | -0.970120 |
| H | 1.139289 | 1.654364 | -1.006758 |
| N | -1.099143 | 1.259042 | -1.625132 |
| H | -0.470580 | 0.494023 | -1.410250 |
| C | -4.609076 | 0.903545 | 1.054979 |
| C | -2.176806 | 0.586867 | 0.977618 |
| C | -3.985860 | 1.144019 | 2.451447 |
| H | -5.152595 | 1.786067 | 0.707141 |
| C | -2.679398 | 0.342601 | 2.406751 |
| H | -1.559097 | -0.238715 | 0.613373 |
| H | -1.582337 | 1.505814 | 0.938308 |
| H | -4.656591 | 0.840414 | 3.259454 |
| H | -3.767418 | 2.211906 | 2.572271 |
| H | -2.885703 | -0.722624 | 2.564085 |
| H | -1.942382 | 0.655783 | 3.151810 |
| N | -3.418993 | 0.725316 | 0.190977 |
| O | -4.709868 | 1.172320 | -1.605751 |
| S | -1.615115 | 3.882456 | -1.672663 |
| C | -5.575520 | -0.275092 | 1.028370 |
| C | -6.941280 | -0.035794 | 1.227335 |
| C | -5.148909 | -1.599351 | 0.872577 |
| C | -7.855645 | -1.087421 | 1.278384 |
| H | -7.292330 | 0.988482 | 1.332332 |
| C | -6.061203 | -2.655498 | 0.919717 |
| C | -7.417983 | -2.404510 | 1.125963 |
| H | -8.911694 | -0.877261 | 1.427125 |

| | | | |
|---|---|---|---|
| O | 4.686796 | -3.520514 | -1.532216 |
| O | 1.645444 | -0.434580 | -1.270724 |
| C | 3.863740 | -3.678384 | -0.633372 |
| C | 2.813248 | -2.690148 | -0.263110 |
| O | 3.860892 | -4.724964 | 0.195144 |
| C | 4.936414 | -5.669977 | 0.022788 |
| H | 5.898454 | -5.179367 | 0.186407 |
| H | 4.762621 | -6.437717 | 0.775732 |
| H | 4.912183 | -6.098374 | -0.982176 |
| S | 2.751446 | -1.395448 | -1.530056 |
| C | 4.339888 | -0.543927 | -1.530924 |
| H | 4.229584 | 0.320624 | -2.189799 |
| H | 4.513289 | -0.253592 | -0.483791 |
| H | 5.103138 | -1.241668 | -1.874174 |
| C | 2.562383 | -2.073511 | -3.199442 |
| H | 3.377978 | -2.775314 | -3.375367 |
| H | 1.588007 | -2.566331 | -3.221690 |
| H | 2.579215 | -1.233061 | -3.897794 |
| C | 1.439693 | -3.184339 | 0.096901 |
| C | 0.858690 | -4.273910 | -0.575459 |
| C | 0.726366 | -2.566428 | 1.134494 |
| C | -0.401000 | -4.743704 | -0.210509 |
| H | 1.406789 | -4.775754 | -1.369403 |
| C | -0.534613 | -3.041069 | 1.499288 |
| H | 1.166034 | -1.732445 | 1.669932 |
| C | -1.100794 | -4.126933 | 0.829931 |
| H | -0.833088 | -5.592884 | -0.732456 |
| H | -1.065611 | -2.565837 | 2.318964 |
| H | -2.079412 | -4.498027 | 1.121760 |
| C | 2.499914 | 1.053386 | 2.425685 |
| C | 1.507954 | 1.754436 | 3.113331 |
| C | 0.917201 | 1.205553 | 4.254317 |
| C | 1.332158 | -0.052926 | 4.698883 |
| C | 2.323600 | -0.754732 | 4.012087 |
| C | 2.926037 | -0.215691 | 2.858870 |
| H | 2.968711 | 1.500617 | 1.554104 |
| H | 1.206385 | 2.737586 | 2.759565 |
| H | 0.156314 | 1.758206 | 4.799600 |
| H | 0.888866 | -0.488132 | 5.591767 |
| H | 2.649995 | -1.727772 | 4.367681 |
| S | 4.212685 | -1.112159 | 2.003318 |
| H | 3.330281 | -2.005744 | 0.757953 |
| H | -4.097018 | -1.807031 | 0.697761 |
| H | -5.710606 | -3.676730 | 0.790535 |
| H | -8.128908 | -3.225813 | 1.159330 |
| C | -2.346996 | -0.411820 | -2.993179 |
| C | 1.495015 | 3.619083 | -0.615607 |
| C | 1.040845 | 4.818963 | -0.050682 |
| C | 2.874915 | 3.416230 | -0.795424 |
| C | 1.981714 | 5.779230 | 0.299800 |
| H | -0.010747 | 5.012721 | 0.097713 |
| C | 3.764480 | 4.410046 | -0.414959 |
| H | 3.255945 | 2.503763 | -1.240151 |
| C | 3.352853 | 5.616048 | 0.136092 |
| H | 4.058635 | 6.385513 | 0.422679 |
| F | 1.540151 | 6.930137 | 0.842323 |
| F | 5.082479 | 4.196495 | -0.604648 |
| C | -1.236469 | -0.273027 | -4.057324 |
| H | -0.240991 | -0.173688 | -3.611100 |
| H | -1.230219 | -1.158157 | -4.705243 |
| H | -1.405784 | 0.604629 | -4.691551 |
| C | -3.684005 | -0.637052 | -3.728831 |

| | | | |
|---|---|---|---|
| H | -4.517341 | -0.768813 | -3.036782 |
| H | -3.928319 | 0.212924 | -4.374702 |
| H | -3.605097 | -1.532535 | -4.357739 |
| C | -2.059631 | -1.635436 | -2.100620 |
| H | -1.099762 | -1.567547 | -1.573673 |
| H | -2.846665 | -1.774462 | -1.352612 |
| H | -2.024155 | -2.545263 | -2.712025 |

TS Conformation 133

B3LYP/6-31G(d) Energy = -3411.86713
M06-2X/def2tzvpp/IEFPCM(chloroform) Energy = -3411.87961
M06-2X/def2tzvpp/IEFPCM(chloroform)//B3LYP/6-31G(d) Quasiharmonic Free Energy = -3411.154836

Frequencies (Top 3 out of 291)

1. -950.0738 cm-1
2. 6.4618 cm-1
3. 10.2343 cm-1

B3LYP/6-31G(d) Molecular Geometry in Cartesian Coordinates

| | | | |
|---|---|---|---|
| C | 3.899245 | -3.054691 | 0.450624 |
| C | 3.587481 | -1.897448 | 1.433296 |
| H | 4.583783 | -1.513666 | 1.665082 |
| C | 3.449243 | 0.431179 | 0.537831 |
| N | 2.517472 | 1.324537 | 0.052857 |
| H | 1.632374 | 0.916392 | -0.230588 |
| N | 2.866335 | -0.763324 | 0.870559 |
| H | 1.853478 | -0.773297 | 0.916620 |
| C | 1.807043 | -2.840787 | -1.062121 |
| C | 3.526425 | -4.537902 | -1.429115 |
| C | 1.176643 | -4.103759 | -1.702063 |
| H | 1.221628 | -2.540272 | -0.189850 |
| C | 2.370193 | -4.751678 | -2.415259 |
| H | 4.491451 | -4.367656 | -1.915825 |
| H | 3.658673 | -5.385217 | -0.747378 |
| H | 0.344261 | -3.861905 | -2.367270 |
| H | 0.803961 | -4.763753 | -0.908066 |
| H | 2.566134 | -4.233429 | -3.359954 |
| H | 2.212284 | -5.810363 | -2.640251 |
| N | 3.125194 | -3.348582 | -0.641458 |
| O | 4.896716 | -3.736099 | 0.686767 |
| S | 5.094341 | 0.737289 | 0.677641 |
| C | 1.857586 | -1.678469 | -2.055048 |
| C | 0.648781 | -1.098159 | -2.471099 |
| C | 3.054940 | -1.201970 | -2.598052 |
| C | 0.638238 | -0.077280 | -3.422690 |
| H | -0.297916 | -1.451362 | -2.067520 |
| C | 3.046261 | -0.175064 | -3.545833 |
| C | 1.840139 | 0.385990 | -3.966422 |
| H | -0.312103 | 0.342544 | -3.742346 |
| O | -4.472305 | -0.505609 | 2.566592 |
| O | -0.402981 | -0.163170 | 1.071373 |
| C | -4.277068 | 0.413476 | 1.773543 |
| C | -3.008069 | 0.652116 | 1.040974 |
| O | -5.219632 | 1.298161 | 1.424023 |
| C | -6.535726 | 1.059669 | 1.961384 |
| H | -7.121542 | 1.933427 | 1.676615 |
| H | -6.956383 | 0.154066 | 1.517735 |
| H | -6.495159 | 0.955568 | 3.047751 |

| | | | |
|---|---|---|---|
| S | -1.716658 | -0.405820 | 1.723038 |
| C | -1.543952 | -0.196548 | 3.515051 |
| H | -0.775661 | -0.894064 | 3.857151 |
| H | -1.219592 | 0.834677 | 3.670996 |
| H | -2.514571 | -0.390038 | 3.973236 |
| C | -2.224822 | -2.121748 | 1.495937 |
| H | -3.148421 | -2.283289 | 2.050096 |
| H | -2.384350 | -2.229088 | 0.416031 |
| H | -1.399515 | -2.742581 | 1.850938 |
| C | -2.533714 | 2.070476 | 0.864485 |
| C | -2.640149 | 3.000787 | 1.913836 |
| C | -1.988714 | 2.488267 | -0.357994 |
| C | -2.218402 | 4.316952 | 1.743019 |
| H | -3.081210 | 2.698814 | 2.861135 |
| C | -1.568256 | 3.808388 | -0.528823 |
| H | -1.921237 | 1.784394 | -1.180857 |
| C | -1.678759 | 4.721784 | 0.519962 |
| H | -2.312402 | 5.025466 | 2.561387 |
| H | -1.145881 | 4.123955 | -1.477504 |
| H | -1.346665 | 5.747261 | 0.384581 |
| C | -5.188908 | -2.673122 | -1.758033 |
| C | -6.513653 | -3.109618 | -1.721378 |
| C | -7.555711 | -2.195881 | -1.552318 |
| C | -7.256304 | -0.836945 | -1.430192 |
| C | -5.932746 | -0.394926 | -1.475603 |
| C | -4.875089 | -1.308551 | -1.633368 |
| H | -4.383734 | -3.390249 | -1.891731 |
| H | -6.731147 | -4.170228 | -1.824411 |
| H | -8.587207 | -2.536673 | -1.524380 |
| H | -8.059079 | -0.111455 | -1.316665 |
| H | -5.710433 | 0.665195 | -1.399131 |
| S | -3.177429 | -0.757609 | -1.708604 |
| H | -3.132784 | 0.056518 | -0.216359 |
| H | 3.998906 | -1.624327 | -2.268404 |
| H | 3.987243 | 0.186225 | -3.951395 |
| H | 1.835194 | 1.179419 | -4.708921 |
| C | 2.965358 | -2.389524 | 2.792963 |
| C | 2.658307 | 2.670178 | -0.336235 |
| C | 3.535620 | 3.569479 | 0.285868 |
| C | 1.796185 | 3.122003 | -1.347958 |
| C | 3.530701 | 4.891685 | -0.137080 |
| H | 4.212079 | 3.254889 | 1.066428 |
| C | 1.827068 | 4.459313 | -1.708539 |
| H | 1.127712 | 2.440135 | -1.860783 |
| C | 2.686184 | 5.382167 | -1.127840 |
| H | 2.702010 | 6.421989 | -1.429714 |
| F | 4.374596 | 5.755542 | 0.462625 |
| F | 0.968547 | 4.883631 | -2.666693 |
| C | 1.617857 | -3.107749 | 2.581611 |
| H | 1.728510 | -4.001903 | 1.957693 |
| H | 1.204446 | -3.428122 | 3.546291 |
| H | 0.879865 | -2.449390 | 2.105966 |
| C | 2.756522 | -1.165154 | 3.708666 |
| H | 3.689975 | -0.607073 | 3.838723 |
| H | 2.010032 | -0.469457 | 3.309870 |
| H | 2.419923 | -1.493930 | 4.699671 |
| C | 3.938867 | -3.357078 | 3.496794 |
| H | 4.132204 | -4.251118 | 2.901539 |
| H | 4.904000 | -2.874360 | 3.683280 |
| H | 3.518049 | -3.660827 | 4.463479 |

TS Conformation 134

B3LYP/6-31G(d) Energy = -3411.866644
M06-2X/def2tzvpp/IEFPCM(chloroform) Energy = -3411.879596
M06-2X/def2tzvpp/IEFPCM(chloroform)//B3LYP/6-31G(d) Quasiharmonic Free Energy = -3411.154545

Frequencies (Top 3 out of 291)

1. -972.3616 cm-1
2. 8.3696 cm-1
3. 12.2031 cm-1

B3LYP/6-31G(d) Molecular Geometry in Cartesian Coordinates

| | | | |
|---|---|---|---|
| C | 5.032063 | -0.868441 | -0.082551 |
| C | 4.051482 | -0.560418 | 1.075902 |
| H | 4.605159 | 0.172425 | 1.667152 |
| C | 2.576002 | 1.451998 | 0.923272 |
| N | 1.329131 | 1.819884 | 0.457112 |
| H | 0.937807 | 1.192988 | -0.239254 |
| N | 2.816928 | 0.119761 | 0.697831 |
| H | 2.007841 | -0.455612 | 0.492405 |
| C | 3.285214 | -1.194583 | -1.962398 |
| C | 5.688860 | -1.437976 | -2.345622 |
| C | 3.541654 | -2.177335 | -3.134432 |
| H | 2.601791 | -1.655045 | -1.245051 |
| C | 4.927768 | -1.756661 | -3.639137 |
| H | 6.413147 | -0.624961 | -2.452665 |
| H | 6.238529 | -2.303892 | -1.959090 |
| H | 2.761059 | -2.122912 | -3.898105 |
| H | 3.575077 | -3.202909 | -2.744619 |
| H | 4.841504 | -0.863539 | -4.267219 |
| H | 5.423879 | -2.533497 | -4.228177 |
| N | 4.631024 | -1.067283 | -1.378737 |
| O | 6.228698 | -0.947846 | 0.194745 |
| S | 3.692010 | 2.485842 | 1.635244 |
| C | 2.679502 | 0.118075 | -2.456186 |
| C | 3.433180 | 1.286347 | -2.607249 |
| C | 1.323241 | 0.142476 | -2.816246 |
| C | 2.848045 | 2.447894 | -3.117953 |
| H | 4.475176 | 1.295079 | -2.303205 |
| C | 0.735669 | 1.300484 | -3.328473 |
| C | 1.502036 | 2.458701 | -3.484013 |
| H | 3.447512 | 3.348515 | -3.218624 |
| O | -3.863607 | -3.758438 | -0.001295 |
| O | 0.111962 | -1.987975 | 0.111432 |
| C | -3.768043 | -2.687088 | 0.596196 |
| C | -2.538160 | -1.862912 | 0.667175 |
| O | -4.791131 | -2.084852 | 1.211617 |
| C | -6.084809 | -2.696853 | 1.049499 |
| H | -6.738265 | -2.170737 | 1.744850 |
| H | -6.430688 | -2.553601 | 0.022788 |
| H | -6.040665 | -3.762779 | 1.282884 |
| S | -1.130858 | -2.799385 | 0.055699 |
| C | -1.454263 | -3.318721 | -1.642304 |
| H | -2.259568 | -4.051333 | -1.637181 |
| H | -0.509950 | -3.718149 | -2.020309 |
| H | -1.755591 | -2.408997 | -2.176449 |
| C | -0.899327 | -4.344194 | 0.973200 |
| H | -0.053890 | -4.876007 | 0.530026 |
| H | -0.675259 | -4.058398 | 2.003105 |
| H | -1.826879 | -4.915126 | 0.905435 |

| | | | |
|---|---|---|---|
| C | -2.192796 | -1.161219 | 1.953031 |
| C | -2.381351 | -1.793244 | 3.195610 |
| C | -1.656056 | 0.133077 | 1.926418 |
| C | -2.042783 | -1.144197 | 4.380534 |
| H | -2.819321 | -2.788013 | 3.235717 |
| C | -1.312090 | 0.780609 | 3.114009 |
| H | -1.535355 | 0.639845 | 0.975475 |
| C | -1.501067 | 0.143405 | 4.340661 |
| H | -2.201738 | -1.641473 | 5.333502 |
| H | -0.899831 | 1.784513 | 3.075194 |
| H | -1.233286 | 0.649386 | 5.264100 |
| C | -4.890705 | 1.666937 | -1.565656 |
| C | -6.261519 | 1.910141 | -1.658933 |
| C | -7.142655 | 0.886768 | -2.015008 |
| C | -6.637377 | -0.386644 | -2.285576 |
| C | -5.265820 | -0.634242 | -2.199374 |
| C | -4.373926 | 0.387979 | -1.831868 |
| H | -4.209700 | 2.466292 | -1.289228 |
| H | -6.642148 | 2.907275 | -1.450851 |
| H | -8.209448 | 1.081047 | -2.088467 |
| H | -7.310404 | -1.188839 | -2.581142 |
| H | -4.877766 | -1.622884 | -2.430398 |
| S | -2.613293 | 0.094974 | -1.725878 |
| H | -2.615996 | -0.938521 | -0.383863 |
| H | 0.716411 | -0.752581 | -2.694609 |
| H | -0.319423 | 1.294053 | -3.586921 |
| H | 1.048008 | 3.364703 | -3.875387 |
| C | 3.809319 | -1.803841 | 2.015780 |
| C | 0.559390 | 2.983660 | 0.641944 |
| C | 0.799759 | 3.941803 | 1.639599 |
| C | -0.559995 | 3.124640 | -0.201266 |
| C | -0.077199 | 5.013758 | 1.749647 |
| H | 1.652953 | 3.875173 | 2.297070 |
| C | -1.398189 | 4.214454 | -0.028786 |
| H | -0.795978 | 2.395716 | -0.969402 |
| C | -1.190462 | 5.190785 | 0.937669 |
| H | -1.855179 | 6.037954 | 1.050851 |
| F | 0.163657 | 5.931115 | 2.708801 |
| F | -2.473144 | 4.334274 | -0.839851 |
| C | 3.304571 | -3.035390 | 1.237222 |
| H | 4.028290 | -3.355190 | 0.478426 |
| H | 3.160880 | -3.876919 | 1.926684 |
| H | 2.343192 | -2.848820 | 0.745548 |
| C | 2.779417 | -1.417905 | 3.098279 |
| H | 3.081752 | -0.503870 | 3.621199 |
| H | 1.776888 | -1.254124 | 2.689642 |
| H | 2.707476 | -2.222135 | 3.840605 |
| C | 5.131988 | -2.170821 | 2.721473 |
| H | 5.916654 | -2.431602 | 2.009192 |
| H | 5.499431 | -1.332789 | 3.324167 |
| H | 4.965195 | -3.022591 | 3.392648 |

TS Conformation 135

B3LYP/6-31G(d) Energy = -3411.871067
M06-2X/def2tzvpp/IEFPCM(chloroform) Energy = -3411.879346
M06-2X/def2tzvpp/IEFPCM(chloroform)//B3LYP/6-31G(d) Quasiharmonic Free Energy = -3411.154472

Frequencies (Top 3 out of 291)

1. -777.3310 cm-1

2. 11.7536 cm-1
3. 13.5956 cm-1

B3LYP/6-31G(d) Molecular Geometry in Cartesian Coordinates

| | | | |
|---|---|---|---|
| C | 3.991262 | 0.642552 | 0.524168 |
| C | 3.157980 | 1.552655 | 1.472119 |
| H | 3.612408 | 2.550461 | 1.455273 |
| C | 1.157220 | 2.922526 | 0.774547 |
| N | -0.163735 | 2.732957 | 0.424386 |
| H | -0.482909 | 1.768206 | 0.437582 |
| N | 1.811161 | 1.734606 | 0.928496 |
| H | 1.290381 | 0.883350 | 0.743716 |
| C | 4.744206 | 0.033198 | -1.717113 |
| C | 3.430031 | 2.089790 | -1.499754 |
| C | 4.635063 | 0.757854 | -3.079615 |
| H | 5.775715 | 0.089490 | -1.346026 |
| C | 4.378874 | 2.219403 | -2.697709 |
| H | 2.390478 | 1.975602 | -1.837610 |
| H | 3.464985 | 2.952534 | -0.832296 |
| H | 3.782951 | 0.355606 | -3.641524 |
| H | 5.531559 | 0.607998 | -3.688038 |
| H | 3.946988 | 2.814032 | -3.508771 |
| H | 5.311813 | 2.703507 | -2.384782 |
| N | 3.893438 | 0.860564 | -0.828312 |
| O | 4.712471 | -0.255564 | 0.948865 |
| S | 1.891905 | 4.423165 | 0.982932 |
| C | 4.362301 | -1.436035 | -1.794925 |
| C | 3.067104 | -1.896895 | -1.533884 |
| C | 5.333005 | -2.359941 | -2.199797 |
| C | 2.750516 | -3.251212 | -1.667670 |
| H | 2.310585 | -1.194956 | -1.191431 |
| C | 5.018582 | -3.710885 | -2.348322 |
| C | 3.725227 | -4.161531 | -2.080910 |
| H | 1.748666 | -3.604059 | -1.432460 |
| O | -3.509036 | -1.806608 | -3.204329 |
| O | -0.550085 | -0.097867 | -0.533201 |
| C | -3.753108 | -1.935070 | -2.006586 |
| C | -2.814152 | -1.569976 | -0.913727 |
| O | -4.859267 | -2.515293 | -1.533336 |
| C | -5.756237 | -3.060882 | -2.521185 |
| H | -6.105922 | -2.276334 | -3.196685 |
| H | -5.252108 | -3.839679 | -3.097779 |
| H | -6.585205 | -3.479341 | -1.951547 |
| S | -1.451150 | -0.595804 | -1.608435 |
| C | -0.538071 | -1.640421 | -2.756646 |
| H | 0.379068 | -1.103335 | -3.007982 |
| H | -0.321895 | -2.562314 | -2.201073 |
| H | -1.172520 | -1.838673 | -3.619847 |
| C | -2.032695 | 0.807090 | -2.597516 |
| H | -2.700796 | 0.422975 | -3.368586 |
| H | -2.545336 | 1.484587 | -1.910001 |
| H | -1.150130 | 1.294335 | -3.019742 |
| C | -3.333553 | -0.945893 | 0.350409 |
| C | -4.446776 | -0.087086 | 0.338486 |
| C | -2.688905 | -1.206223 | 1.569960 |
| C | -4.913712 | 0.486979 | 1.519322 |
| H | -4.962044 | 0.121231 | -0.595310 |
| C | -3.154773 | -0.624930 | 2.749929 |
| H | -1.833892 | -1.872396 | 1.596914 |
| C | -4.265499 | 0.220051 | 2.728459 |
| H | -5.769263 | 1.154573 | 1.491212 |

| | | | |
|---|---|---|---|
| H | -2.650692 | -0.843904 | 3.686711 |
| H | -4.627634 | 0.668812 | 3.649351 |
| C | -1.781286 | -4.926379 | 2.164885 |
| C | -1.450224 | -4.989963 | 3.518211 |
| C | -0.242549 | -4.458460 | 3.977991 |
| C | 0.633164 | -3.866710 | 3.065805 |
| C | 0.304579 | -3.801667 | 1.709791 |
| C | -0.911389 | -4.326525 | 1.235871 |
| H | -2.719229 | -5.344353 | 1.811301 |
| H | -2.139038 | -5.459966 | 4.216623 |
| H | 0.015549 | -4.511587 | 5.032461 |
| H | 1.581982 | -3.458076 | 3.405217 |
| H | 0.999884 | -3.351604 | 1.007320 |
| S | -1.347868 | -4.262994 | -0.496331 |
| H | -2.179223 | -2.721778 | -0.634970 |
| H | 6.348904 | -2.019339 | -2.389851 |
| H | 5.788404 | -4.412425 | -2.659409 |
| H | 3.479005 | -5.215128 | -2.179571 |
| C | 3.137940 | 1.075466 | 2.971116 |
| C | -1.192686 | 3.644998 | 0.126529 |
| C | -0.987713 | 4.911462 | -0.438398 |
| C | -2.504609 | 3.187918 | 0.359447 |
| C | -2.101272 | 5.687219 | -0.735901 |
| H | 0.004232 | 5.294256 | -0.625834 |
| C | -3.573073 | 4.008741 | 0.026609 |
| H | -2.694126 | 2.231683 | 0.834794 |
| C | -3.412000 | 5.273008 | -0.525604 |
| H | -4.257328 | 5.904117 | -0.769876 |
| F | -1.899892 | 6.902426 | -1.279935 |
| F | -4.826281 | 3.559628 | 0.257245 |
| C | 2.589598 | -0.359457 | 3.107775 |
| H | 3.220429 | -1.080332 | 2.584160 |
| H | 2.548974 | -0.639560 | 4.167148 |
| H | 1.564991 | -0.442074 | 2.720717 |
| C | 2.237801 | 2.026127 | 3.788876 |
| H | 2.565633 | 3.067300 | 3.709620 |
| H | 1.190523 | 1.979635 | 3.472444 |
| H | 2.274756 | 1.736434 | 4.845526 |
| C | 4.564010 | 1.161885 | 3.556777 |
| H | 5.247970 | 0.477277 | 3.055284 |
| H | 4.959912 | 2.182238 | 3.470057 |
| H | 4.538100 | 0.911703 | 4.624233 |

TS Conformation 136

B3LYP/6-31G(d) Energy = -3411.862375
M06-2X/def2tzvpp/IEFPCM(chloroform) Energy = -3411.87877
M06-2X/def2tzvpp/IEFPCM(chloroform)//B3LYP/6-31G(d) Quasiharmonic Free Energy = -3411.153982

Frequencies (Top 3 out of 291)

1. -807.3303 cm-1
2. 7.8343 cm-1
3. 11.1921 cm-1

B3LYP/6-31G(d) Molecular Geometry in Cartesian Coordinates

| | | | |
|---|---|---|---|
| C | 4.480406 | -1.613833 | 0.944814 |
| C | 3.366950 | -1.028960 | 1.851127 |
| H | 3.921991 | -0.334752 | 2.486487 |
| C | 2.377049 | 1.145644 | 1.116182 |

| | | | |
|---|---|---|---|
| N | 1.202531 | 1.646965 | 0.603702 |
| H | 0.450673 | 0.974412 | 0.490992 |
| N | 2.356063 | -0.223231 | 1.180320 |
| H | 1.504474 | -0.687184 | 0.885445 |
| C | 3.105792 | -1.828818 | -1.241784 |
| C | 5.435360 | -2.553926 | -1.075096 |
| C | 3.378300 | -2.975505 | -2.249859 |
| H | 2.210366 | -2.057823 | -0.658977 |
| C | 4.893485 | -2.884369 | -2.472507 |
| H | 6.310159 | -1.897089 | -1.089785 |
| H | 5.721600 | -3.450343 | -0.513273 |
| H | 2.793067 | -2.867466 | -3.167248 |
| H | 3.121360 | -3.936477 | -1.784713 |
| H | 5.118750 | -2.075675 | -3.175639 |
| H | 5.323822 | -3.806414 | -2.873789 |
| N | 4.302263 | -1.895407 | -0.386091 |
| O | 5.565285 | -1.858674 | 1.470525 |
| S | 3.686681 | 2.061356 | 1.633993 |
| C | 2.901020 | -0.493906 | -1.955342 |
| C | 1.664780 | -0.235224 | -2.564484 |
| C | 3.916018 | 0.460894 | -2.068441 |
| C | 1.449200 | 0.941513 | -3.279871 |
| H | 0.853861 | -0.954897 | -2.466796 |
| C | 3.704752 | 1.639377 | -2.788594 |
| C | 2.474662 | 1.882571 | -3.398851 |
| H | 0.477521 | 1.127208 | -3.729459 |
| O | -3.969378 | -4.066785 | -0.412134 |
| O | -0.692591 | -1.279816 | 0.218484 |
| C | -4.177107 | -2.918162 | -0.797038 |
| C | -3.278639 | -1.761108 | -0.539010 |
| O | -5.293874 | -2.537058 | -1.423362 |
| C | -6.305217 | -3.551688 | -1.582412 |
| H | -7.124556 | -3.056637 | -2.102361 |
| H | -6.631636 | -3.915464 | -0.605565 |
| H | -5.920023 | -4.387522 | -2.171718 |
| S | -1.693603 | -2.372279 | 0.086408 |
| C | -1.003967 | -3.677071 | -0.968589 |
| H | -0.777056 | -3.208264 | -1.928443 |
| H | -1.752285 | -4.463368 | -1.069246 |
| H | -0.089383 | -4.036370 | -0.490529 |
| C | -1.999800 | -3.167448 | 1.675209 |
| H | -2.561677 | -4.083511 | 1.494672 |
| H | -1.021562 | -3.346594 | 2.126244 |
| H | -2.604191 | -2.450842 | 2.251728 |
| C | -3.094210 | -0.723970 | -1.611680 |
| C | -3.096492 | 0.635648 | -1.271905 |
| C | -2.930253 | -1.088306 | -2.959563 |
| C | -2.944151 | 1.612344 | -2.257276 |
| H | -3.242192 | 0.928838 | -0.237773 |
| C | -2.777305 | -0.113657 | -3.943558 |
| H | -2.951796 | -2.137987 | -3.244361 |
| C | -2.782783 | 1.239605 | -3.592356 |
| H | -2.952909 | 2.659982 | -1.973268 |
| H | -2.661838 | -0.408558 | -4.982959 |
| H | -2.670760 | 2.000249 | -4.360300 |
| C | -4.313979 | 2.070778 | 1.945498 |
| C | -3.740334 | 3.306310 | 2.251478 |
| C | -2.613237 | 3.378139 | 3.072997 |
| C | -2.063096 | 2.199500 | 3.582132 |
| C | -2.630475 | 0.963074 | 3.270871 |
| C | -3.767582 | 0.873652 | 2.446202 |
| H | -5.202556 | 2.021254 | 1.322433 |

| | | | |
|---|---|---|---|
| H | -4.175951 | 4.215380 | 1.844954 |
| H | -2.168084 | 4.339573 | 3.313201 |
| H | -1.187336 | 2.242070 | 4.225348 |
| H | -2.206065 | 0.052755 | 3.685646 |
| S | -4.531529 | -0.700438 | 2.084509 |
| H | -3.790806 | -1.193990 | 0.573755 |
| H | 4.866442 | 0.296193 | -1.571099 |
| H | 4.503047 | 2.373030 | -2.859527 |
| H | 2.309994 | 2.804184 | -3.950039 |
| C | 2.725609 | -2.109983 | 2.800040 |
| C | 0.829739 | 2.943676 | 0.202259 |
| C | -0.552378 | 3.170702 | 0.087416 |
| C | 1.732660 | 3.959974 | -0.139535 |
| C | -0.995628 | 4.397393 | -0.377187 |
| H | -1.273277 | 2.415405 | 0.376621 |
| C | 1.219890 | 5.171927 | -0.582115 |
| H | 2.799831 | 3.820393 | -0.060377 |
| C | -0.138577 | 5.433752 | -0.722703 |
| H | -0.504850 | 6.389357 | -1.076512 |
| F | -2.331096 | 4.589698 | -0.509122 |
| F | 2.091585 | 6.146223 | -0.914961 |
| C | 2.083144 | -3.262492 | 2.001511 |
| H | 2.823833 | -3.797630 | 1.396338 |
| H | 1.629922 | -3.989766 | 2.686767 |
| H | 1.292249 | -2.899927 | 1.332209 |
| C | 1.653299 | -1.428001 | 3.676266 |
| H | 0.796498 | -1.069942 | 3.095565 |
| H | 2.072541 | -0.568994 | 4.211510 |
| H | 1.278103 | -2.137269 | 4.423714 |
| C | 3.804413 | -2.696363 | 3.733985 |
| H | 3.342698 | -3.420982 | 4.416081 |
| H | 4.266517 | -1.909105 | 4.339132 |
| H | 4.602838 | -3.192682 | 3.180102 |

TS Conformation 137

B3LYP/6-31G(d) Energy = -3411.866124
M06-2X/def2tzvpp/IEFPCM(chloroform) Energy = -3411.877366
M06-2X/def2tzvpp/IEFPCM(chloroform)//B3LYP/6-31G(d) Quasiharmonic Free Energy = -3411.153781

Frequencies (Top 3 out of 291)

1. -791.0649 cm-1
2. 6.1510 cm-1
3. 9.3121 cm-1

B3LYP/6-31G(d) Molecular Geometry in Cartesian Coordinates

| | | | |
|---|---|---|---|
| C | -3.980560 | 0.447651 | -1.007921 |
| C | -2.618730 | 0.196010 | -1.706631 |
| H | -2.777050 | 0.652830 | -2.687271 |
| C | -1.136019 | 2.164150 | -1.265711 |
| N | 0.140331 | 2.425279 | -0.796791 |
| H | 0.697631 | 1.599729 | -0.599691 |
| N | -1.458230 | 0.850420 | -1.112411 |
| H | -0.799630 | 0.258779 | -0.619741 |
| C | -5.441250 | 0.984971 | 0.860449 |
| C | -3.033280 | 0.977920 | 1.324169 |
| C | -5.140369 | 1.736391 | 2.180329 |
| H | -5.936479 | 1.640722 | 0.139339 |
| C | -3.804520 | 1.140581 | 2.641409 |

| | | | |
|---|---|---|---|
| H | -2.286960 | 0.179300 | 1.373179 |
| H | -2.521629 | 1.912170 | 1.064529 |
| H | -5.943699 | 1.621002 | 2.912399 |
| H | -5.020199 | 2.806081 | 1.970809 |
| H | -3.966800 | 0.165761 | 3.115339 |
| H | -3.270549 | 1.777300 | 3.354369 |
| N | -4.088700 | 0.669841 | 0.343259 |
| O | -4.987650 | 0.388171 | -1.707711 |
| S | -2.183549 | 3.283720 | -1.945821 |
| C | -6.327990 | -0.238628 | 1.059119 |
| C | -7.712460 | -0.105918 | 0.893059 |
| C | -5.822471 | -1.480309 | 1.461879 |
| C | -8.570511 | -1.179227 | 1.130689 |
| H | -8.120300 | 0.846812 | 0.562309 |
| C | -6.677131 | -2.558958 | 1.697959 |
| C | -8.055111 | -2.411808 | 1.536429 |
| H | -9.641020 | -1.055697 | 0.988389 |
| O | 4.214560 | -0.903023 | 3.495469 |
| O | 1.051400 | -0.121731 | 0.642109 |
| C | 4.525480 | -1.071353 | 2.318409 |
| C | 3.563600 | -1.031902 | 1.185689 |
| O | 5.751569 | -1.411493 | 1.914509 |
| C | 6.716559 | -1.649534 | 2.959589 |
| H | 6.388079 | -2.476943 | 3.592419 |
| H | 7.639629 | -1.904684 | 2.440499 |
| H | 6.848750 | -0.753084 | 3.570149 |
| S | 2.000230 | -0.323512 | 1.771389 |
| C | 2.226720 | 1.253548 | 2.637199 |
| H | 2.589711 | 1.968538 | 1.894359 |
| H | 1.246431 | 1.562019 | 3.009289 |
| H | 2.944170 | 1.099748 | 3.443379 |
| C | 1.287159 | -1.440901 | 2.991859 |
| H | 1.916199 | -1.423012 | 3.881529 |
| H | 0.271810 | -1.083621 | 3.179149 |
| H | 1.299169 | -2.436371 | 2.524049 |
| C | 3.964520 | -0.426093 | -0.128431 |
| C | 4.837620 | 0.673917 | -0.193831 |
| C | 3.440880 | -0.950082 | -1.320541 |
| C | 5.186860 | 1.231057 | -1.422831 |
| H | 5.259150 | 1.087707 | 0.718359 |
| C | 3.788680 | -0.388032 | -2.549461 |
| H | 2.776099 | -1.806402 | -1.285401 |
| C | 4.659390 | 0.701567 | -2.603851 |
| H | 5.852131 | 2.088617 | -1.454791 |
| H | 3.380310 | -0.808392 | -3.463891 |
| H | 4.927480 | 1.138177 | -3.561851 |
| C | 0.851768 | -4.929621 | -0.620361 |
| C | 0.359668 | -5.346741 | -1.858041 |
| C | 1.181538 | -5.335941 | -2.987721 |
| C | 2.507518 | -4.915942 | -2.859641 |
| C | 3.006298 | -4.511822 | -1.620441 |
| C | 2.184208 | -4.501622 | -0.478011 |
| H | 0.208358 | -4.944661 | 0.254619 |
| H | -0.670832 | -5.685901 | -1.938391 |
| H | 0.797558 | -5.660801 | -3.951021 |
| H | 3.164158 | -4.915442 | -3.726811 |
| H | 4.045548 | -4.210503 | -1.524691 |
| S | 2.818108 | -4.029372 | 1.122139 |
| H | 3.222229 | -2.324342 | 1.017749 |
| H | -4.750281 | -1.612039 | 1.575949 |
| H | -6.263541 | -3.516718 | 2.003899 |
| H | -8.720731 | -3.251867 | 1.715969 |

| | | | |
|---|---|---|---|
| C | -2.313021 | -1.329160 | -1.943741 |
| C | 0.862311 | 3.619919 | -0.657501 |
| C | 0.280542 | 4.885609 | -0.497021 |
| C | 2.264401 | 3.487418 | -0.614241 |
| C | 1.120982 | 5.975799 | -0.309621 |
| H | -0.789378 | 5.026069 | -0.537271 |
| C | 3.044722 | 4.616518 | -0.415531 |
| H | 2.746631 | 2.531648 | -0.787981 |
| C | 2.508462 | 5.887478 | -0.256401 |
| H | 3.131833 | 6.760688 | -0.110451 |
| F | 0.558323 | 7.189269 | -0.152671 |
| F | 4.388582 | 4.469007 | -0.383261 |
| C | -2.090011 | -2.086060 | -0.617951 |
| H | -1.225221 | -1.714560 | -0.053501 |
| H | -2.975201 | -2.030690 | 0.024799 |
| H | -1.888941 | -3.142700 | -0.824261 |
| C | -1.050191 | -1.448940 | -2.825371 |
| H | -0.154480 | -1.046981 | -2.339771 |
| H | -1.184440 | -0.918240 | -3.775341 |
| H | -0.848811 | -2.502291 | -3.048361 |
| C | -3.486231 | -1.987219 | -2.698251 |
| H | -3.223831 | -3.025140 | -2.935091 |
| H | -3.697181 | -1.465069 | -3.637231 |
| H | -4.407701 | -1.987719 | -2.112561 |

TS Conformation 138

B3LYP/6-31G(d) Energy = -3411.867181
M06-2X/def2tzvpp/IEFPCM(chloroform) Energy = -3411.879064
M06-2X/def2tzvpp/IEFPCM(chloroform)//B3LYP/6-31G(d) Quasiharmonic Free Energy = -3411.153762

Frequencies (Top 3 out of 291)

1. -827.6669 cm-1
2. 9.3566 cm-1
3. 11.0947 cm-1

B3LYP/6-31G(d) Molecular Geometry in Cartesian Coordinates

| | | | |
|---|---|---|---|
| C | -4.770596 | -1.257254 | 0.275031 |
| C | -4.051019 | -0.719508 | -0.987550 |
| H | -4.778591 | -0.010224 | -1.387819 |
| C | -2.753610 | 1.422957 | -0.917739 |
| N | -1.484780 | 1.871694 | -0.599877 |
| H | -0.947454 | 1.244400 | -0.009080 |
| N | -2.847000 | 0.066370 | -0.747310 |
| H | -1.968056 | -0.429202 | -0.652804 |
| C | -2.682984 | -1.640447 | 1.755292 |
| C | -4.946132 | -2.178213 | 2.511349 |
| C | -2.636417 | -2.788447 | 2.796276 |
| H | -2.105446 | -1.930275 | 0.874012 |
| C | -3.933264 | -2.584853 | 3.590121 |
| H | -5.692513 | -1.458388 | 2.860331 |
| H | -5.496173 | -3.035667 | 2.107476 |
| H | -2.657344 | -3.751529 | 2.270925 |
| H | -1.733301 | -2.752224 | 3.410785 |
| H | -4.248147 | -3.480690 | 4.133009 |
| H | -3.802544 | -1.779234 | 4.320545 |
| N | -4.118979 | -1.587598 | 1.434234 |
| O | -5.990149 | -1.404918 | 0.203807 |
| S | -4.024582 | 2.395838 | -1.420924 |

| | | | |
|---|---|---|---|
| C | -2.110836 | -0.343836 | 2.326437 |
| C | -0.720526 | -0.236834 | 2.486518 |
| C | -2.919258 | 0.718653 | 2.743232 |
| C | -0.151322 | 0.896734 | 3.069937 |
| H | -0.078476 | -1.049932 | 2.153842 |
| C | -2.352633 | 1.857112 | 3.322054 |
| C | -0.971141 | 1.947170 | 3.493625 |
| H | 0.926328 | 0.962784 | 3.193007 |
| O | 3.751843 | -1.138511 | -3.530829 |
| O | 0.305882 | -0.785221 | -0.903841 |
| C | 3.867812 | -1.671301 | -2.429931 |
| C | 2.906331 | -1.501495 | -1.310129 |
| O | 4.932283 | -2.391216 | -2.062029 |
| C | 6.014800 | -2.442096 | -3.012929 |
| H | 6.401149 | -1.436820 | -3.195214 |
| H | 5.677567 | -2.879846 | -3.955614 |
| H | 6.774556 | -3.067361 | -2.545225 |
| S | 1.366592 | -0.788848 | -1.946259 |
| C | 1.704422 | 0.882260 | -2.530344 |
| H | 2.361640 | 0.812052 | -3.397038 |
| H | 0.736523 | 1.334226 | -2.759469 |
| H | 2.207544 | 1.396127 | -1.700520 |
| C | 0.770172 | -1.686846 | -3.403512 |
| H | 1.563360 | -1.689359 | -4.151043 |
| H | 0.530031 | -2.696210 | -3.062310 |
| H | -0.131946 | -1.181089 | -3.755498 |
| C | 2.622701 | -2.642830 | -0.375883 |
| C | 2.546894 | -3.968928 | -0.834191 |
| C | 2.415791 | -2.384352 | 0.987053 |
| C | 2.277583 | -5.011537 | 0.050337 |
| H | 2.725479 | -4.189495 | -1.883588 |
| C | 2.144160 | -3.429049 | 1.871289 |
| H | 2.493239 | -1.367277 | 1.355809 |
| C | 2.071711 | -4.742736 | 1.405652 |
| H | 2.230969 | -6.032853 | -0.317430 |
| H | 2.001266 | -3.213602 | 2.926475 |
| H | 1.864567 | -5.555974 | 2.095884 |
| C | 4.577843 | 0.008381 | 2.524658 |
| C | 4.494562 | 0.092522 | 3.915312 |
| C | 3.949699 | 1.224302 | 4.526069 |
| C | 3.487788 | 2.272098 | 3.725206 |
| C | 3.554078 | 2.186301 | 2.333140 |
| C | 4.099949 | 1.049626 | 1.706762 |
| H | 5.028949 | -0.863837 | 2.060159 |
| H | 4.872105 | -0.726055 | 4.524272 |
| H | 3.894773 | 1.293090 | 5.609255 |
| H | 3.072879 | 3.166427 | 4.185088 |
| H | 3.193569 | 3.007343 | 1.721255 |
| S | 4.237916 | 0.989071 | -0.069457 |
| H | 3.441955 | -0.463809 | -0.625176 |
| H | -3.993836 | 0.663356 | 2.599555 |
| H | -2.995640 | 2.675580 | 3.633930 |
| H | -0.531479 | 2.831532 | 3.946446 |
| C | -3.835472 | -1.819318 | -2.094229 |
| C | -0.881435 | 3.132576 | -0.768818 |
| C | -1.222930 | 4.024368 | -1.796598 |
| C | 0.180323 | 3.440777 | 0.099836 |
| C | -0.503655 | 5.207425 | -1.907215 |
| H | -2.038451 | 3.824261 | -2.475539 |
| C | 0.862863 | 4.635542 | -0.072074 |
| H | 0.460322 | 2.775168 | 0.908665 |
| C | 0.549909 | 5.551634 | -1.068362 |

| | | | |
|---|---|---|---|
| H | 1.092525 | 6.481741 | -1.181754 |
| F | -0.835859 | 6.061443 | -2.895896 |
| F | 1.875162 | 4.925458 | 0.772858 |
| C | -5.183415 | -2.473299 | -2.461152 |
| H | -5.913623 | -1.722449 | -2.779652 |
| H | -5.032835 | -3.177292 | -3.288977 |
| H | -5.621892 | -3.015626 | -1.621114 |
| C | -2.858040 | -2.920579 | -1.636326 |
| H | -1.859041 | -2.529505 | -1.405108 |
| H | -2.736701 | -3.667747 | -2.430266 |
| H | -3.231947 | -3.446130 | -0.750178 |
| C | -3.281670 | -1.127159 | -3.356641 |
| H | -2.313044 | -0.650701 | -3.168408 |
| H | -3.964036 | -0.347883 | -3.711721 |
| H | -3.153092 | -1.860342 | -4.162187 |

TS Conformation 139

B3LYP/6-31G(d) Energy = -3411.866874
M06-2X/def2tzvpp/IEFPCM(chloroform) Energy = -3411.879167
M06-2X/def2tzvpp/IEFPCM(chloroform)//B3LYP/6-31G(d) Quasiharmonic Free Energy = -3411.153597

Frequencies (Top 3 out of 291)

1. -854.2644 cm-1
2. 9.9093 cm-1
3. 12.8270 cm-1

B3LYP/6-31G(d) Molecular Geometry in Cartesian Coordinates

| | | | |
|---|---|---|---|
| C | -3.954387 | -2.388600 | -0.810764 |
| C | -2.686905 | -1.893830 | -1.552965 |
| H | -3.099133 | -1.625045 | -2.528339 |
| C | -2.353333 | 0.577874 | -1.490242 |
| N | -1.374384 | 1.479899 | -1.123189 |
| H | -0.510345 | 1.063859 | -0.786966 |
| N | -2.075741 | -0.677851 | -1.025457 |
| H | -1.232690 | -0.785770 | -0.472477 |
| C | -3.232219 | -1.815502 | 1.605616 |
| C | -5.388781 | -2.831093 | 1.092132 |
| C | -3.636432 | -2.790852 | 2.739818 |
| H | -2.204406 | -2.005915 | 1.291251 |
| C | -5.164776 | -2.834956 | 2.610951 |
| H | -6.279588 | -2.274731 | 0.784624 |
| H | -5.483701 | -3.840448 | 0.679780 |
| H | -3.293110 | -2.467863 | 3.725943 |
| H | -3.205479 | -3.778367 | 2.533837 |
| H | -5.603749 | -1.943558 | 3.072301 |
| H | -5.609480 | -3.712352 | 3.089196 |
| N | -4.164963 | -2.205761 | 0.535889 |
| O | -4.805140 | -2.967433 | -1.481921 |
| S | -3.709284 | 0.938778 | -2.410561 |
| C | -3.350406 | -0.353709 | 2.048619 |
| C | -2.590783 | 0.093179 | 3.141930 |
| C | -4.199884 | 0.556807 | 1.414187 |
| C | -2.678312 | 1.412682 | 3.589949 |
| H | -1.944606 | -0.606179 | 3.670576 |
| C | -4.287100 | 1.879801 | 1.857091 |
| C | -3.529621 | 2.314277 | 2.944256 |
| H | -2.101349 | 1.730322 | 4.455835 |
| O | 3.668863 | 1.378940 | 3.274009 |

| | | | |
|---|---|---|---|
| O | 0.710098 | -0.061629 | 0.453462 |
| C | 4.081283 | 1.196830 | 2.130483 |
| C | 3.292561 | 0.558446 | 1.045810 |
| O | 5.332296 | 1.461047 | 1.742628 |
| C | 6.226378 | 1.925994 | 2.773426 |
| H | 7.179110 | 2.088509 | 2.270750 |
| H | 5.855229 | 2.855318 | 3.212372 |
| H | 6.326469 | 1.168964 | 3.554475 |
| S | 1.559888 | 0.454987 | 1.563568 |
| C | 1.454913 | -0.644219 | 2.986121 |
| H | 1.958270 | -0.165432 | 3.825630 |
| H | 0.389802 | -0.806682 | 3.164271 |
| H | 1.977983 | -1.564205 | 2.689126 |
| C | 0.893837 | 2.041128 | 2.130770 |
| H | 1.529176 | 2.403586 | 2.939239 |
| H | 0.910110 | 2.712703 | 1.268419 |
| H | -0.136708 | 1.862931 | 2.452789 |
| C | 3.400626 | 1.061788 | -0.365015 |
| C | 3.264567 | 0.162812 | -1.434232 |
| C | 3.612256 | 2.423386 | -0.644389 |
| C | 3.345161 | 0.616511 | -2.751167 |
| H | 3.110389 | -0.891622 | -1.233728 |
| C | 3.697049 | 2.873750 | -1.960707 |
| H | 3.726430 | 3.133284 | 0.170389 |
| C | 3.560618 | 1.969556 | -3.017493 |
| H | 3.243504 | -0.092683 | -3.567514 |
| H | 3.847498 | 3.930495 | -2.158812 |
| H | 3.621796 | 2.320621 | -4.043831 |
| C | 2.970286 | -4.366174 | -0.030262 |
| C | 2.797944 | -5.150213 | -1.172080 |
| C | 3.561455 | -4.911823 | -2.317254 |
| C | 4.511321 | -3.887910 | -2.299974 |
| C | 4.695741 | -3.111687 | -1.154913 |
| C | 3.920538 | -3.329529 | -0.000599 |
| H | 2.375382 | -4.559149 | 0.857815 |
| H | 2.065749 | -5.954826 | -1.164291 |
| H | 3.426249 | -5.522298 | -3.206095 |
| H | 5.123693 | -3.698971 | -3.178855 |
| H | 5.452467 | -2.332534 | -1.142224 |
| S | 4.171774 | -2.371316 | 1.484591 |
| H | 3.667148 | -0.749229 | 1.104838 |
| H | -4.781804 | 0.234445 | 0.557337 |
| H | -4.952588 | 2.570075 | 1.346135 |
| H | -3.605678 | 3.340972 | 3.291645 |
| C | -1.611808 | -3.014515 | -1.811409 |
| C | -1.302425 | 2.874016 | -1.272966 |
| C | -2.418782 | 3.721778 | -1.259467 |
| C | -0.009766 | 3.423454 | -1.367077 |
| C | -2.205881 | 5.090675 | -1.350555 |
| H | -3.423791 | 3.332481 | -1.195606 |
| C | 0.133430 | 4.801652 | -1.444348 |
| H | 0.868579 | 2.791009 | -1.434566 |
| C | -0.945918 | 5.674788 | -1.439044 |
| H | -0.817197 | 6.747594 | -1.509559 |
| F | -3.280479 | 5.903371 | -1.331737 |
| F | 1.381429 | 5.313959 | -1.537778 |
| C | -2.271313 | -4.200999 | -2.544204 |
| H | -2.746660 | -3.877440 | -3.475927 |
| H | -1.504663 | -4.945021 | -2.790934 |
| H | -3.040949 | -4.685584 | -1.939260 |
| C | -0.964065 | -3.533989 | -0.512521 |
| H | -0.429606 | -2.749737 | 0.038831 |

| | | | |
|---|---|---|---|
| H | -1.705083 | -3.990654 | 0.154173 |
| H | -0.216749 | -4.298347 | -0.751056 |
| C | -0.512390 | -2.433768 | -2.727633 |
| H | -0.940685 | -2.044860 | -3.658702 |
| H | 0.047974 | -1.623692 | -2.249096 |
| H | 0.208799 | -3.217442 | -2.985063 |

TS Conformation 140

B3LYP/6-31G(d) Energy = -3411.863985
M06-2X/def2tzvpp/IEFPCM(chloroform) Energy = -3411.878929
M06-2X/def2tzvpp/IEFPCM(chloroform)//B3LYP/6-31G(d) Quasiharmonic Free Energy = -3411.153564

Frequencies (Top 3 out of 291)

1. -792.5906 cm-1
2. 4.9331 cm-1
3. 14.6057 cm-1

B3LYP/6-31G(d) Molecular Geometry in Cartesian Coordinates

| | | | |
|---|---|---|---|
| C | 3.505409 | 1.798810 | 0.545700 |
| C | 2.369159 | 1.923040 | 1.595820 |
| H | 2.515829 | 2.936050 | 1.977510 |
| C | 0.337269 | 2.958970 | 0.570070 |
| N | -0.876471 | 2.573210 | 0.013360 |
| H | -0.943491 | 1.589920 | -0.241960 |
| N | 1.013329 | 1.882960 | 1.057400 |
| H | 0.501329 | 1.010770 | 1.134980 |
| C | 4.492059 | 1.138600 | -1.575330 |
| C | 2.153389 | 0.481780 | -1.212780 |
| C | 3.822939 | 0.763110 | -2.919330 |
| H | 4.874749 | 2.162560 | -1.598060 |
| C | 2.671119 | -0.161850 | -2.506960 |
| H | 1.700689 | -0.254750 | -0.544040 |
| H | 1.403009 | 1.246010 | -1.443240 |
| H | 4.527799 | 0.297920 | -3.613440 |
| H | 3.426449 | 1.669500 | -3.393180 |
| H | 3.037619 | -1.174150 | -2.308090 |
| H | 1.884759 | -0.233870 | -3.265450 |
| N | 3.360609 | 1.091080 | -0.620570 |
| O | 4.573809 | 2.346590 | 0.808930 |
| S | 0.934439 | 4.526790 | 0.591840 |
| C | 5.652799 | 0.220680 | -1.209420 |
| C | 6.955979 | 0.611660 | -1.541600 |
| C | 5.465319 | -1.029870 | -0.607950 |
| C | 8.043499 | -0.224580 | -1.290300 |
| H | 7.120429 | 1.588220 | -1.991940 |
| C | 6.551989 | -1.868640 | -0.351590 |
| C | 7.844949 | -1.471400 | -0.694440 |
| H | 9.047059 | 0.102630 | -1.550090 |
| O | -1.399151 | -0.231870 | -1.014000 |
| O | -1.214411 | -0.330290 | 1.873840 |
| C | -2.087471 | -1.248360 | -1.017720 |
| C | -2.804071 | -1.843180 | 0.144990 |
| O | -2.256321 | -2.016830 | -2.099800 |
| C | -1.510921 | -1.637470 | -3.274000 |
| H | -0.440401 | -1.742700 | -3.081830 |
| H | -1.832081 | -2.331550 | -4.049950 |
| H | -1.737061 | -0.607560 | -3.559060 |
| S | -2.591821 | -0.795630 | 1.602700 |

| | | | |
|---|---|---|---|
| C | -3.737921 | 0.610580 | 1.604420 |
| H | -3.451311 | 1.271260 | 0.782660 |
| H | -3.610411 | 1.119510 | 2.563160 |
| H | -4.757411 | 0.244480 | 1.474530 |
| C | -3.167981 | -1.819370 | 2.969620 |
| H | -2.496671 | -2.686660 | 2.983040 |
| H | -4.203921 | -2.119730 | 2.807030 |
| H | -3.055791 | -1.215260 | 3.872620 |
| C | -4.246551 | -2.247000 | -0.076840 |
| C | -4.719391 | -3.486230 | 0.382800 |
| C | -5.132331 | -1.397930 | -0.766330 |
| C | -6.049231 | -3.856280 | 0.173510 |
| H | -4.035581 | -4.175940 | 0.866960 |
| C | -6.458101 | -1.772080 | -0.976590 |
| H | -4.780971 | -0.445170 | -1.156940 |
| C | -6.921631 | -3.001020 | -0.500100 |
| H | -6.397391 | -4.822230 | 0.528190 |
| H | -7.126141 | -1.106710 | -1.516120 |
| H | -7.955021 | -3.293440 | -0.664130 |
| C | 1.405389 | -4.064240 | 1.082550 |
| C | 2.631869 | -3.977770 | 0.422300 |
| C | 2.690979 | -4.004500 | -0.973570 |
| C | 1.504269 | -4.126240 | -1.701750 |
| C | 0.276529 | -4.222820 | -1.044650 |
| C | 0.205639 | -4.189460 | 0.360520 |
| H | 1.367789 | -4.043820 | 2.167570 |
| H | 3.546889 | -3.894020 | 1.004250 |
| H | 3.648079 | -3.944130 | -1.484700 |
| H | 1.536139 | -4.168370 | -2.788530 |
| H | -0.639741 | -4.345410 | -1.614760 |
| S | -1.354211 | -4.355250 | 1.208990 |
| H | -2.129381 | -2.940120 | 0.543610 |
| H | 4.466569 | -1.349500 | -0.325470 |
| H | 6.386919 | -2.833490 | 0.121980 |
| H | 8.690769 | -2.122950 | -0.491390 |
| C | 2.530779 | 0.940830 | 2.811620 |
| C | -1.970031 | 3.336790 | -0.414910 |
| C | -2.837391 | 2.722970 | -1.343030 |
| C | -2.288121 | 4.609420 | 0.085660 |
| C | -3.988961 | 3.387730 | -1.737710 |
| H | -2.595951 | 1.758880 | -1.775110 |
| C | -3.447351 | 5.225760 | -0.365990 |
| H | -1.645581 | 5.116440 | 0.790130 |
| C | -4.332891 | 4.649360 | -1.272260 |
| H | -5.230681 | 5.159250 | -1.598290 |
| F | -4.810971 | 2.769870 | -2.615810 |
| F | -3.747091 | 6.445760 | 0.120010 |
| C | 3.913209 | 1.149150 | 3.464100 |
| H | 4.055289 | 2.190960 | 3.770590 |
| H | 3.988999 | 0.516470 | 4.356830 |
| H | 4.731959 | 0.894220 | 2.788450 |
| C | 2.399109 | -0.535810 | 2.388450 |
| H | 3.159869 | -0.808720 | 1.649940 |
| H | 2.540649 | -1.185100 | 3.260630 |
| H | 1.414309 | -0.781080 | 1.974410 |
| C | 1.449859 | 1.279870 | 3.860910 |
| H | 1.509359 | 2.332840 | 4.160290 |
| H | 0.436779 | 1.087390 | 3.494370 |
| H | 1.600189 | 0.666300 | 4.757130 |

TS Conformation 141

B3LYP/6-31G(d) Energy = -3411.865071
M06-2X/def2tzvpp/IEFPCM(chloroform) Energy = -3411.87818
M06-2X/def2tzvpp/IEFPCM(chloroform)//B3LYP/6-31G(d) Quasiharmonic Free Energy = -3411.153458

Frequencies (Top 3 out of 291)

1. -780.1763 cm-1
2. 7.6768 cm-1
3. 8.3533 cm-1

B3LYP/6-31G(d) Molecular Geometry in Cartesian Coordinates

| | | | |
|---|---|---|---|
| C | -4.704799 | -1.891379 | -1.009235 |
| C | -3.529702 | -1.419133 | -1.901726 |
| H | -4.044790 | -0.876256 | -2.698009 |
| C | -2.643941 | 0.893455 | -1.548493 |
| N | -1.579317 | 1.533412 | -0.955859 |
| H | -1.037576 | 0.971187 | -0.307209 |
| N | -2.616433 | -0.455913 | -1.301316 |
| H | -1.774609 | -0.817724 | -0.867717 |
| C | -3.535540 | -1.679369 | 1.293665 |
| C | -5.823154 | -2.505043 | 1.052063 |
| C | -3.869235 | -2.659019 | 2.447985 |
| H | -2.582829 | -1.958280 | 0.837413 |
| C | -5.399771 | -2.585468 | 2.525214 |
| H | -6.706171 | -1.881094 | 0.884870 |
| H | -6.044384 | -3.489298 | 0.624279 |
| H | -3.551018 | -3.671502 | 2.167282 |
| H | -3.369813 | -2.386473 | 3.381568 |
| H | -5.702580 | -1.681381 | 3.064001 |
| H | -5.845192 | -3.444419 | 3.035329 |
| N | -4.644640 | -1.934946 | 0.360113 |
| O | -5.729971 | -2.263784 | -1.578591 |
| S | -3.856599 | 1.641744 | -2.436338 |
| C | -3.443417 | -0.237819 | 1.793756 |
| C | -2.344348 | 0.133759 | 2.582758 |
| C | -4.432951 | 0.714245 | 1.532108 |
| C | -2.245829 | 1.420075 | 3.112457 |
| H | -1.554102 | -0.587448 | 2.782454 |
| C | -4.334064 | 2.005250 | 2.057527 |
| C | -3.245755 | 2.361231 | 2.853030 |
| H | -1.384649 | 1.686638 | 3.719283 |
| O | 4.116618 | -2.963312 | 0.675757 |
| O | 0.278411 | -0.989110 | 0.232664 |
| C | 4.047629 | -1.844530 | 1.179785 |
| C | 2.911324 | -0.904357 | 0.985945 |
| O | 5.017983 | -1.302746 | 1.922479 |
| C | 6.239353 | -2.065160 | 2.015253 |
| H | 6.031892 | -3.082817 | 2.352863 |
| H | 6.851806 | -1.531642 | 2.741749 |
| H | 6.731140 | -2.088157 | 1.040117 |
| S | 1.512267 | -1.819823 | 0.281998 |
| C | 1.157415 | -3.336895 | 1.209545 |
| H | 0.826009 | -3.018756 | 2.200310 |
| H | 0.353112 | -3.858364 | 0.684997 |
| H | 2.070942 | -3.929598 | 1.257268 |
| C | 1.982005 | -2.369991 | -1.368727 |
| H | 1.090624 | -2.814569 | -1.816739 |
| H | 2.300955 | -1.457744 | -1.893481 |
| H | 2.809085 | -3.072653 | -1.269491 |
| C | 2.485067 | -0.018897 | 2.126253 |

| | | | |
|---|---|---|---|
| C | 2.387957 | -0.513933 | 3.438450 |
| C | 2.193944 | 1.331390 | 1.886649 |
| C | 2.014390 | 0.323762 | 4.487020 |
| H | 2.636412 | -1.552531 | 3.645204 |
| C | 1.822486 | 2.171794 | 2.938316 |
| H | 2.291032 | 1.729263 | 0.881756 |
| C | 1.730426 | 1.669620 | 4.237483 |
| H | 1.952993 | -0.069842 | 5.497910 |
| H | 1.604907 | 3.215667 | 2.733560 |
| H | 1.448539 | 2.325975 | 5.056504 |
| C | 5.525625 | -0.472642 | -2.984424 |
| C | 6.819758 | -0.939481 | -3.215726 |
| C | 7.819794 | -0.759900 | -2.257702 |
| C | 7.509258 | -0.097745 | -1.067412 |
| C | 6.216451 | 0.376265 | -0.835271 |
| C | 5.197460 | 0.191799 | -1.788440 |
| H | 4.755424 | -0.610486 | -3.738278 |
| H | 7.047323 | -1.445613 | -4.151203 |
| H | 8.828407 | -1.121249 | -2.439951 |
| H | 8.282604 | 0.068857 | -0.320199 |
| H | 5.989256 | 0.907974 | 0.084157 |
| S | 3.546335 | 0.811645 | -1.514743 |
| H | 3.220957 | -0.130918 | -0.068746 |
| H | -5.273090 | 0.453901 | 0.896580 |
| H | -5.107602 | 2.734176 | 1.833007 |
| H | -3.169181 | 3.366312 | 3.258398 |
| C | -2.760491 | -2.612856 | -2.581933 |
| C | -1.213565 | 2.894389 | -0.937012 |
| C | -0.524963 | 3.333045 | 0.206169 |
| C | -1.431909 | 3.777039 | -2.002333 |
| C | -0.069818 | 4.640354 | 0.255718 |
| H | -0.368423 | 2.673741 | 1.051605 |
| C | -0.962788 | 5.078938 | -1.881915 |
| H | -1.953554 | 3.470524 | -2.896439 |
| C | -0.272777 | 5.552156 | -0.771024 |
| H | 0.087089 | 6.571746 | -0.713284 |
| F | 0.594292 | 5.049640 | 1.364711 |
| F | -1.172754 | 5.927711 | -2.907216 |
| C | -2.100084 | -3.537087 | -1.538243 |
| H | -2.844358 | -3.994665 | -0.876529 |
| H | -1.561325 | -4.349990 | -2.040919 |
| H | -1.376574 | -2.997865 | -0.912268 |
| C | -1.679694 | -2.030717 | -3.517095 |
| H | -2.122209 | -1.349130 | -4.251522 |
| H | -1.185712 | -2.840767 | -4.067350 |
| H | -0.909630 | -1.470953 | -2.975698 |
| C | -3.731359 | -3.451614 | -3.438451 |
| H | -4.218362 | -2.831905 | -4.198456 |
| H | -4.519874 | -3.910746 | -2.839768 |
| H | -3.172726 | -4.243793 | -3.952290 |

TS Conformation 142

B3LYP/6-31G(d) Energy = -3411.868775
M06-2X/def2tzvpp/IEFPCM(chloroform) Energy = -3411.877151
M06-2X/def2tzvpp/IEFPCM(chloroform)//B3LYP/6-31G(d) Quasiharmonic Free Energy = -3411.153343

Frequencies (Top 3 out of 291)

1. -794.7262 cm-1
2. 5.4102 cm-1

3. 6.6553 cm-1

B3LYP/6-31G(d) Molecular Geometry in Cartesian Coordinates

| | | | |
|---|---|---|---|
| C | 4.272280 | -0.010553 | 0.040984 |
| C | 3.801889 | 1.161096 | 0.954038 |
| H | 4.332792 | 2.068103 | 0.643340 |
| C | 1.832294 | 2.639117 | 0.375598 |
| N | 0.473768 | 2.531812 | 0.148918 |
| H | 0.120117 | 1.593044 | -0.011403 |
| N | 2.388190 | 1.433788 | 0.686893 |
| H | 1.760988 | 0.650614 | 0.833573 |
| C | 4.424455 | -1.043789 | -2.168025 |
| C | 3.560558 | 1.248039 | -2.062060 |
| C | 4.172461 | -0.487734 | -3.589708 |
| H | 5.501107 | -1.170195 | -1.999032 |
| C | 4.253182 | 1.033044 | -3.413325 |
| H | 2.470003 | 1.287819 | -2.188414 |
| H | 3.867255 | 2.169735 | -1.564667 |
| H | 3.169180 | -0.777850 | -3.924399 |
| H | 4.890516 | -0.889792 | -4.310394 |
| H | 3.771188 | 1.592222 | -4.221250 |
| H | 5.298754 | 1.358607 | -3.353744 |
| N | 3.950468 | 0.054543 | -1.290059 |
| O | 4.892336 | -0.976345 | 0.478738 |
| S | 2.706857 | 4.067925 | 0.229301 |
| C | 3.753702 | -2.383632 | -1.917011 |
| C | 4.502330 | -3.558872 | -2.040383 |
| C | 2.388763 | -2.484646 | -1.619166 |
| C | 3.905349 | -4.809295 | -1.874968 |
| H | 5.566957 | -3.492988 | -2.254165 |
| C | 1.790287 | -3.732985 | -1.437119 |
| C | 2.547373 | -4.899881 | -1.568702 |
| H | 4.506021 | -5.710348 | -1.969232 |
| O | -4.900789 | -2.093719 | -2.124166 |
| O | -1.063638 | -0.147467 | -1.491441 |
| C | -4.677480 | -1.735041 | -0.969973 |
| C | -3.371152 | -1.218764 | -0.488510 |
| O | -5.556861 | -1.863450 | 0.028649 |
| C | -6.791832 | -2.527878 | -0.303876 |
| H | -6.591757 | -3.547998 | -0.639373 |
| H | -7.369568 | -2.532756 | 0.619813 |
| H | -7.321159 | -1.983492 | -1.089728 |
| S | -2.323702 | -0.813673 | -1.910794 |
| C | -1.955503 | -2.341157 | -2.794594 |
| H | -2.887235 | -2.744414 | -3.190537 |
| H | -1.235459 | -2.082104 | -3.574235 |
| H | -1.514914 | -3.013759 | -2.049524 |
| C | -3.186224 | 0.222180 | -3.126052 |
| H | -2.504080 | 0.362923 | -3.968557 |
| H | -4.099708 | -0.295306 | -3.419921 |
| H | -3.399482 | 1.181370 | -2.648294 |
| C | -3.317394 | -0.126439 | 0.541858 |
| C | -2.291812 | -0.126954 | 1.500028 |
| C | -4.265572 | 0.910550 | 0.561193 |
| C | -2.221561 | 0.885448 | 2.457711 |
| H | -1.566863 | -0.933433 | 1.509744 |
| C | -4.196425 | 1.916555 | 1.522562 |
| H | -5.073149 | 0.919988 | -0.166102 |
| C | -3.170839 | 1.908355 | 2.470828 |
| H | -1.431157 | 0.863434 | 3.202474 |
| H | -4.940073 | 2.708398 | 1.528800 |

| | | | |
|---|---|---|---|
| H | -3.115838 | 2.694053 | 3.219085 |
| C | 0.048951 | -3.371612 | 2.192987 |
| C | 0.584248 | -3.132553 | 3.461018 |
| C | -0.249668 | -3.051142 | 4.577906 |
| C | -1.627099 | -3.219861 | 4.414420 |
| C | -2.164528 | -3.464221 | 3.151002 |
| C | -1.336250 | -3.538474 | 2.016057 |
| H | 0.707384 | -3.440463 | 1.332244 |
| H | 1.659482 | -3.014628 | 3.572734 |
| H | 0.168498 | -2.866086 | 5.563916 |
| H | -2.288288 | -3.165775 | 5.276437 |
| H | -3.235108 | -3.602321 | 3.030792 |
| S | -2.032592 | -3.860147 | 0.401572 |
| H | -2.757723 | -2.325869 | -0.021430 |
| H | 1.792424 | -1.581426 | -1.507944 |
| H | 0.737918 | -3.799594 | -1.170019 |
| H | 2.080899 | -5.870003 | -1.420476 |
| C | 4.095607 | 0.951921 | 2.482460 |
| C | -0.468718 | 3.529139 | -0.174116 |
| C | -0.476303 | 4.810590 | 0.391406 |
| C | -1.489499 | 3.148184 | -1.056318 |
| C | -1.509470 | 5.672096 | 0.044360 |
| H | 0.294697 | 5.137805 | 1.073607 |
| C | -2.504402 | 4.044135 | -1.340660 |
| H | -1.478237 | 2.165000 | -1.507160 |
| C | -2.550033 | 5.327107 | -0.814602 |
| H | -3.344814 | 6.022232 | -1.054815 |
| F | -1.520512 | 6.904881 | 0.587397 |
| F | -3.494137 | 3.640289 | -2.180048 |
| C | 3.561791 | 2.169602 | 3.266087 |
| H | 2.473513 | 2.263896 | 3.189814 |
| H | 3.809943 | 2.052694 | 4.327621 |
| H | 4.002906 | 3.106798 | 2.912912 |
| C | 5.622107 | 0.881023 | 2.701985 |
| H | 6.058729 | 0.008680 | 2.215074 |
| H | 6.114534 | 1.783451 | 2.316465 |
| H | 5.836745 | 0.825938 | 3.775973 |
| C | 3.419191 | -0.320977 | 3.031397 |
| H | 3.622834 | -0.412062 | 4.105022 |
| H | 2.326828 | -0.282256 | 2.921920 |
| H | 3.789556 | -1.217585 | 2.531252 |

TS Conformation 143

B3LYP/6-31G(d) Energy = -3411.864491
M06-2X/def2tzvpp/IEFPCM(chloroform) Energy = -3411.877338
M06-2X/def2tzvpp/IEFPCM(chloroform)//B3LYP/6-31G(d) Quasiharmonic Free Energy = -3411.153194

Frequencies (Top 3 out of 291)

1. -878.2600 cm-1
2. 5.0736 cm-1
3. 8.1970 cm-1

B3LYP/6-31G(d) Molecular Geometry in Cartesian Coordinates

| | | | |
|---|---|---|---|
| C | 4.100290 | 0.697657 | 0.977782 |
| C | 2.793468 | 0.623312 | 1.812935 |
| H | 2.937953 | 1.424246 | 2.542814 |
| C | 1.137001 | 2.175427 | 0.776557 |
| N | -0.144692 | 2.174984 | 0.265848 |

| | | | |
|---|---|---|---|
| H | -0.678634 | 1.324898 | 0.418884 |
| N | 1.554418 | 0.918238 | 1.103155 |
| H | 0.946704 | 0.143233 | 0.861676 |
| C | 5.442496 | 0.653718 | -1.045356 |
| C | 3.020573 | 0.404911 | -1.343244 |
| C | 5.046053 | 0.930336 | -2.515935 |
| H | 5.929041 | 1.525351 | -0.600144 |
| C | 3.723011 | 0.174168 | -2.688095 |
| H | 2.316981 | -0.396595 | -1.099452 |
| H | 2.468552 | 1.350933 | -1.359966 |
| H | 5.821909 | 0.614716 | -3.218168 |
| H | 4.881548 | 2.005945 | -2.650626 |
| H | 3.913051 | -0.893613 | -2.847044 |
| H | 3.123440 | 0.537212 | -3.528640 |
| N | 4.132577 | 0.465980 | -0.375656 |
| O | 5.141723 | 0.916932 | 1.591736 |
| S | 2.108122 | 3.528548 | 0.991435 |
| C | 6.386369 | -0.532395 | -0.889090 |
| C | 7.763563 | -0.297761 | -0.791699 |
| C | 5.937741 | -1.858979 | -0.898077 |
| C | 8.669251 | -1.355618 | -0.714578 |
| H | 8.128027 | 0.726634 | -0.764065 |
| C | 6.839930 | -2.921555 | -0.818168 |
| C | 8.210247 | -2.674016 | -0.729111 |
| H | 9.733269 | -1.149485 | -0.632394 |
| O | -2.739376 | -4.938672 | -0.578135 |
| O | -0.814083 | -1.094896 | 0.082461 |
| C | -3.421782 | -3.954821 | -0.856616 |
| C | -3.028034 | -2.542176 | -0.608737 |
| O | -4.661712 | -4.030861 | -1.349311 |
| C | -5.198687 | -5.358974 | -1.504629 |
| H | -6.198919 | -5.214979 | -1.911558 |
| H | -4.581269 | -5.944342 | -2.190570 |
| H | -5.244766 | -5.863855 | -0.537043 |
| S | -1.297243 | -2.490269 | -0.097674 |
| C | -0.212455 | -3.337408 | -1.277972 |
| H | 0.799427 | -3.324820 | -0.865550 |
| H | -0.588504 | -4.351824 | -1.414475 |
| H | -0.258684 | -2.758901 | -2.203196 |
| C | -1.150823 | -3.423732 | 1.437878 |
| H | -1.318549 | -4.477070 | 1.216747 |
| H | -0.151824 | -3.221780 | 1.829590 |
| H | -1.941729 | -3.031124 | 2.093361 |
| C | -3.335489 | -1.515154 | -1.663601 |
| C | -3.156938 | -1.801758 | -3.028734 |
| C | -3.823917 | -0.254785 | -1.294079 |
| C | -3.466018 | -0.851810 | -3.999341 |
| H | -2.799700 | -2.782810 | -3.334460 |
| C | -4.135476 | 0.696225 | -2.267187 |
| H | -3.980328 | -0.027535 | -0.245287 |
| C | -3.955092 | 0.400566 | -3.618532 |
| H | -3.329976 | -1.089448 | -5.050859 |
| H | -4.517493 | 1.665433 | -1.962711 |
| H | -4.198570 | 1.141532 | -4.374981 |
| C | -2.993830 | 0.171099 | 3.143503 |
| C | -2.824363 | 1.524418 | 3.441366 |
| C | -3.795277 | 2.457300 | 3.068624 |
| C | -4.939055 | 2.019912 | 2.396383 |
| C | -5.111863 | 0.665926 | 2.101882 |
| C | -4.140499 | -0.285790 | 2.466877 |
| H | -2.244566 | -0.549843 | 3.459087 |
| H | -1.932363 | 1.850643 | 3.971011 |

| | | | |
|---|---|---|---|
| H | -3.661652 | 3.510397 | 3.299504 |
| H | -5.701855 | 2.735102 | 2.098580 |
| H | -6.010978 | 0.328547 | 1.594161 |
| S | -4.390453 | -2.020588 | 2.119991 |
| H | -3.645117 | -2.215136 | 0.566788 |
| H | 4.872213 | -2.063897 | -0.953101 |
| H | 6.470789 | -3.944301 | -0.822467 |
| H | 8.913323 | -3.500018 | -0.663250 |
| C | 2.667381 | -0.722226 | 2.617389 |
| C | -0.905989 | 3.210862 | -0.310340 |
| C | -0.350652 | 4.290769 | -1.011155 |
| C | -2.301031 | 3.076981 | -0.223009 |
| C | -1.213183 | 5.209867 | -1.593312 |
| H | 0.717144 | 4.428416 | -1.091518 |
| C | -3.104444 | 4.020439 | -0.842492 |
| H | -2.757685 | 2.270707 | 0.338642 |
| C | -2.599296 | 5.109494 | -1.539769 |
| H | -3.244813 | 5.840552 | -2.010316 |
| F | -0.676552 | 6.246653 | -2.267988 |
| F | -4.448092 | 3.861527 | -0.772835 |
| C | 2.627702 | -1.942362 | 1.673944 |
| H | 3.561189 | -2.041551 | 1.110684 |
| H | 2.492045 | -2.863373 | 2.254825 |
| H | 1.802125 | -1.878218 | 0.952613 |
| C | 1.382042 | -0.677208 | 3.472941 |
| H | 1.373539 | 0.207914 | 4.118917 |
| H | 0.465962 | -0.655931 | 2.873082 |
| H | 1.336502 | -1.561006 | 4.120527 |
| C | 3.865360 | -0.880762 | 3.576354 |
| H | 4.816247 | -0.927591 | 3.043877 |
| H | 3.921955 | -0.039135 | 4.274882 |
| H | 3.741791 | -1.799937 | 4.162178 |

TS Conformation 144

B3LYP/6-31G(d) Energy = -3411.867228
M06-2X/def2tzvpp/IEFPCM(chloroform) Energy = -3411.87786
M06-2X/def2tzvpp/IEFPCM(chloroform)//B3LYP/6-31G(d) Quasiharmonic Free Energy = -3411.152973

Frequencies (Top 3 out of 291)

1. -741.2304 cm-1
2. 6.4305 cm-1
3. 11.7595 cm-1

B3LYP/6-31G(d) Molecular Geometry in Cartesian Coordinates

| | | | |
|---|---|---|---|
| C | -3.688121 | 1.153464 | -0.877828 |
| C | -2.642691 | 1.192824 | -2.026057 |
| H | -2.964617 | 2.060774 | -2.605997 |
| C | -0.739447 | 2.700624 | -1.403887 |
| N | 0.555265 | 2.609890 | -0.927945 |
| H | 0.857090 | 1.672826 | -0.677642 |
| N | -1.269596 | 1.460605 | -1.621838 |
| H | -0.676215 | 0.660168 | -1.439271 |
| C | -4.437452 | 0.894570 | 1.421179 |
| C | -2.045812 | 0.526879 | 1.013579 |
| C | -3.643233 | 0.975521 | 2.747351 |
| H | -4.988456 | 1.819914 | 1.233407 |
| C | -2.380292 | 0.153340 | 2.464326 |
| H | -1.510075 | -0.277287 | 0.500609 |

| | | | |
|---|---|---|---|
| H | -1.424046 | 1.428589 | 0.990477 |
| H | -4.222643 | 0.605186 | 3.597032 |
| H | -3.374807 | 2.019941 | 2.946970 |
| H | -2.601063 | -0.917519 | 2.541598 |
| H | -1.549048 | 0.373916 | 3.140578 |
| N | -3.366353 | 0.782026 | 0.403264 |
| O | -4.846331 | 1.446029 | -1.166532 |
| S | -1.586164 | 4.124669 | -1.664632 |
| C | -5.439721 | -0.253710 | 1.394433 |
| C | -5.078428 | -1.565007 | 1.062756 |
| C | -6.764825 | -0.004907 | 1.774202 |
| C | -6.014324 | -2.600027 | 1.114600 |
| H | -4.061126 | -1.778323 | 0.746891 |
| C | -7.702178 | -1.035840 | 1.830522 |
| C | -7.329338 | -2.340550 | 1.501664 |
| H | -5.715223 | -3.610825 | 0.847777 |
| O | 4.556198 | -2.914262 | -2.134096 |
| O | 1.352821 | -0.309658 | -0.860860 |
| C | 3.788477 | -3.405819 | -1.310543 |
| C | 2.705660 | -2.653835 | -0.620847 |
| O | 3.889050 | -4.655159 | -0.851600 |
| C | 5.012447 | -5.418258 | -1.338038 |
| H | 4.923265 | -6.391706 | -0.857481 |
| H | 4.966679 | -5.515490 | -2.425458 |
| H | 5.948326 | -4.931735 | -1.054756 |
| S | 2.520027 | -1.041718 | -1.427505 |
| C | 4.044143 | -0.110222 | -1.180484 |
| H | 3.802803 | 0.936489 | -1.382070 |
| H | 4.326130 | -0.275935 | -0.129003 |
| H | 4.805975 | -0.511388 | -1.847999 |
| C | 2.309785 | -1.174028 | -3.222909 |
| H | 2.242741 | -0.156919 | -3.617685 |
| H | 3.161470 | -1.720609 | -3.627653 |
| H | 1.370492 | -1.708074 | -3.382802 |
| C | 1.371441 | -3.299309 | -0.382731 |
| C | 0.637854 | -2.966114 | 0.766301 |
| C | 0.831314 | -4.226542 | -1.290577 |
| C | -0.605058 | -3.554416 | 1.002692 |
| H | 1.047114 | -2.257168 | 1.477997 |
| C | -0.407145 | -4.817178 | -1.048312 |
| H | 1.393278 | -4.506966 | -2.177811 |
| C | -1.129706 | -4.479817 | 0.098956 |
| H | -1.157044 | -3.296288 | 1.902136 |
| H | -0.807190 | -5.540634 | -1.753171 |
| H | -2.094002 | -4.942668 | 0.289619 |
| C | 3.350674 | 0.551623 | 3.004461 |
| C | 2.540792 | 1.352737 | 3.810930 |
| C | 1.529240 | 0.781654 | 4.587336 |
| C | 1.348695 | -0.603416 | 4.556350 |
| C | 2.162263 | -1.407049 | 3.754605 |
| C | 3.172493 | -0.844193 | 2.951855 |
| H | 4.145067 | 1.010365 | 2.422973 |
| H | 2.711152 | 2.426090 | 3.829155 |
| H | 0.903215 | 1.404808 | 5.220484 |
| H | 0.581028 | -1.065882 | 5.173046 |
| H | 2.035546 | -2.486158 | 3.759727 |
| S | 4.237573 | -1.866628 | 1.947954 |
| H | 3.255526 | -2.296096 | 0.545156 |
| H | -7.066901 | 1.011343 | 2.017817 |
| H | -8.726566 | -0.818127 | 2.121547 |
| H | -8.058970 | -3.145075 | 1.538897 |
| C | -2.714509 | -0.058435 | -2.974150 |

| | | | |
|---|---|---|---|
| C | 1.463485 | 3.599271 | -0.507935 |
| C | 1.541236 | 4.884593 | -1.060612 |
| C | 2.376737 | 3.206183 | 0.487522 |
| C | 2.523127 | 5.743882 | -0.582394 |
| H | 0.852428 | 5.216296 | -1.823279 |
| C | 3.335432 | 4.111452 | 0.915085 |
| H | 2.312076 | 2.232945 | 0.962962 |
| C | 3.443078 | 5.397610 | 0.401911 |
| H | 4.193705 | 6.094553 | 0.753182 |
| F | 2.599090 | 6.979215 | -1.114348 |
| F | 4.191750 | 3.725635 | 1.888190 |
| C | -1.744356 | 0.165694 | -4.153456 |
| H | -0.700345 | 0.229053 | -3.826758 |
| H | -1.825714 | -0.662641 | -4.867455 |
| H | -1.979362 | 1.093766 | -4.686623 |
| C | -4.138816 | -0.202667 | -3.548377 |
| H | -4.879690 | -0.391596 | -2.769503 |
| H | -4.445924 | 0.706053 | -4.076627 |
| H | -4.160182 | -1.036256 | -4.261186 |
| C | -2.342815 | -1.361386 | -2.237904 |
| H | -1.322944 | -1.351358 | -1.831987 |
| H | -3.032074 | -1.561581 | -1.411237 |
| H | -2.401146 | -2.211991 | -2.927352 |

TS Conformation 145

B3LYP/6-31G(d) Energy = -3411.863142
M06-2X/def2tzvpp/IEFPCM(chloroform) Energy = -3411.877917
M06-2X/def2tzvpp/IEFPCM(chloroform)//B3LYP/6-31G(d) Quasiharmonic Free Energy = -3411.152847

Frequencies (Top 3 out of 291)

1. -781.2749 cm-1
2. 10.2836 cm-1
3. 11.4212 cm-1

B3LYP/6-31G(d) Molecular Geometry in Cartesian Coordinates

| | | | |
|---|---|---|---|
| C | 4.049680 | 0.529115 | 0.979762 |
| C | 2.825469 | 0.284997 | 1.902162 |
| H | 3.110147 | 0.830832 | 2.806143 |
| C | 1.210843 | 2.154390 | 1.524856 |
| N | -0.146522 | 2.332635 | 1.294677 |
| H | -0.702840 | 1.483782 | 1.372062 |
| N | 1.551008 | 0.839526 | 1.453450 |
| H | 0.836131 | 0.183526 | 1.157356 |
| C | 5.185027 | 0.941651 | -1.128190 |
| C | 2.736308 | 0.798751 | -1.214082 |
| C | 4.652984 | 1.549806 | -2.448949 |
| H | 5.746846 | 1.682736 | -0.553344 |
| C | 3.293097 | 0.867666 | -2.642945 |
| H | 2.031933 | -0.025991 | -1.083865 |
| H | 2.227837 | 1.737290 | -0.960119 |
| H | 5.342616 | 1.390983 | -3.281975 |
| H | 4.514844 | 2.630295 | -2.321496 |
| H | 3.424959 | -0.140052 | -3.052168 |
| H | 2.628054 | 1.418395 | -3.317001 |
| N | 3.942432 | 0.626416 | -0.384265 |
| O | 5.151309 | 0.576294 | 1.521131 |
| S | 2.319157 | 3.365908 | 1.862980 |
| C | 6.095103 | -0.261119 | -1.346878 |

| | | | |
|---|---|---|---|
| C | 5.599151 | -1.547414 | -1.592037 |
| C | 7.482108 | -0.067741 | -1.365394 |
| C | 6.465621 | -2.610404 | -1.854307 |
| H | 4.528097 | -1.725965 | -1.562690 |
| C | 8.351433 | -1.125703 | -1.630054 |
| C | 7.845282 | -2.403128 | -1.877517 |
| H | 6.060228 | -3.602557 | -2.036344 |
| O | -1.522908 | -0.260617 | 0.950166 |
| O | -0.360329 | -1.153788 | -1.605268 |
| C | -2.595035 | -0.745992 | 0.582584 |
| C | -3.027417 | -0.944618 | -0.829587 |
| O | -3.531705 | -1.191962 | 1.415089 |
| C | -3.196675 | -1.229139 | 2.817830 |
| H | -4.108673 | -1.553228 | 3.317070 |
| H | -2.892541 | -0.240649 | 3.170171 |
| H | -2.393719 | -1.951560 | 2.980268 |
| S | -1.662760 | -0.538141 | -1.935441 |
| C | -2.237579 | -1.124782 | -3.540230 |
| H | -2.361942 | -2.206938 | -3.421875 |
| H | -1.445602 | -0.897668 | -4.257041 |
| H | -3.179236 | -0.643320 | -3.807284 |
| C | -1.454861 | 1.250354 | -2.168388 |
| H | -0.723073 | 1.386713 | -2.968524 |
| H | -1.069486 | 1.644066 | -1.226327 |
| H | -2.415713 | 1.701660 | -2.420216 |
| C | -4.339193 | -0.295389 | -1.225576 |
| C | -5.310509 | -1.012135 | -1.941614 |
| C | -4.617327 | 1.031197 | -0.851273 |
| C | -6.519376 | -0.404296 | -2.286462 |
| H | -5.135092 | -2.055386 | -2.185140 |
| C | -5.827104 | 1.633870 | -1.189618 |
| H | -3.889455 | 1.598428 | -0.279028 |
| C | -6.779851 | 0.916492 | -1.916699 |
| H | -7.265483 | -0.973499 | -2.833885 |
| H | -6.016913 | 2.655444 | -0.873520 |
| H | -7.725435 | 1.380844 | -2.182734 |
| C | -3.521635 | -5.313434 | 0.948360 |
| C | -3.058840 | -5.744949 | 2.190821 |
| C | -1.792764 | -5.367446 | 2.646553 |
| C | -0.990772 | -4.561294 | 1.835772 |
| C | -1.445230 | -4.136799 | 0.584389 |
| C | -2.724222 | -4.499177 | 0.123818 |
| H | -4.510574 | -5.600497 | 0.603420 |
| H | -3.692634 | -6.377643 | 2.808205 |
| H | -1.432862 | -5.705347 | 3.614890 |
| H | 0.003634 | -4.272833 | 2.168953 |
| H | -0.801611 | -3.534839 | -0.051086 |
| S | -3.339015 | -3.947802 | -1.456493 |
| H | -3.143140 | -2.264406 | -1.075190 |
| H | 7.884608 | 0.921333 | -1.157832 |
| H | 9.424830 | -0.954224 | -1.632075 |
| H | 8.520442 | -3.230873 | -2.077662 |
| C | 2.648426 | -1.225316 | 2.303713 |
| C | -0.915843 | 3.478713 | 1.054371 |
| C | -2.311275 | 3.314035 | 1.173624 |
| C | -0.403902 | 4.716046 | 0.634686 |
| C | -3.150922 | 4.366885 | 0.846793 |
| H | -2.736012 | 2.385715 | 1.539049 |
| C | -1.301012 | 5.737146 | 0.347107 |
| H | 0.658935 | 4.888487 | 0.551470 |
| C | -2.684003 | 5.605185 | 0.427724 |
| H | -3.352531 | 6.421606 | 0.185334 |

| | | | |
|---|---|---|---|
| F | -4.487344 | 4.166200 | 0.928724 |
| F | -0.802553 | 6.920425 | -0.057759 |
| C | 3.947103 | -1.745398 | 2.953742 |
| H | 4.228049 | -1.138263 | 3.820874 |
| H | 3.791291 | -2.776082 | 3.294167 |
| H | 4.790296 | -1.732868 | 2.260739 |
| C | 2.312152 | -2.109486 | 1.085497 |
| H | 2.208136 | -3.153839 | 1.402106 |
| H | 1.370554 | -1.831116 | 0.596300 |
| H | 3.110064 | -2.072566 | 0.336728 |
| C | 1.516472 | -1.330989 | 3.350381 |
| H | 1.709463 | -0.674562 | 4.207179 |
| H | 1.455590 | -2.359471 | 3.724588 |
| H | 0.533109 | -1.075329 | 2.941476 |

TS Conformation 146

B3LYP/6-31G(d) Energy = -3411.868086
M06-2X/def2tzvpp/IEFPCM(chloroform) Energy = -3411.877717
M06-2X/def2tzvpp/IEFPCM(chloroform)//B3LYP/6-31G(d) Quasiharmonic Free Energy = -3411.151735

Frequencies (Top 3 out of 291)

1. -806.4062 cm-1
2. 10.9488 cm-1
3. 14.0223 cm-1

B3LYP/6-31G(d) Molecular Geometry in Cartesian Coordinates

| | | | |
|---|---|---|---|
| C | 3.877235 | 1.093545 | 0.297498 |
| C | 3.027317 | 1.977073 | 1.257653 |
| H | 3.352504 | 3.014788 | 1.118710 |
| C | 0.804338 | 3.042523 | 0.732968 |
| N | -0.522565 | 2.666459 | 0.664433 |
| H | -0.709984 | 1.694414 | 0.908410 |
| N | 1.620353 | 1.956793 | 0.853262 |
| H | 1.189504 | 1.040258 | 0.778917 |
| C | 4.571695 | 0.465296 | -1.957850 |
| C | 2.980760 | 2.322025 | -1.751156 |
| C | 4.273808 | 1.087346 | -3.342595 |
| H | 5.606996 | 0.680723 | -1.664473 |
| C | 3.820196 | 2.515473 | -3.020292 |
| H | 1.954551 | 2.021105 | -2.001842 |
| H | 2.922706 | 3.223193 | -1.138258 |
| H | 3.461699 | 0.530782 | -3.825644 |
| H | 5.146442 | 1.035909 | -4.000398 |
| H | 3.250766 | 2.985136 | -3.828755 |
| H | 4.684941 | 3.154062 | -2.802537 |
| N | 3.676894 | 1.228245 | -1.049940 |
| O | 4.717430 | 0.301285 | 0.718320 |
| S | 1.362715 | 4.631180 | 0.686851 |
| C | 4.384596 | -1.041663 | -1.927681 |
| C | 3.113890 | -1.625510 | -1.853483 |
| C | 5.501837 | -1.874632 | -2.046249 |
| C | 2.964801 | -3.013145 | -1.889259 |
| H | 2.236937 | -0.992869 | -1.743989 |
| C | 5.356775 | -3.261852 | -2.095439 |
| C | 4.087395 | -3.835226 | -2.015038 |
| H | 1.973943 | -3.453972 | -1.804572 |
| O | -1.301194 | -0.121766 | 1.291214 |
| O | -0.090993 | -0.147837 | -1.327936 |

| | | | |
|---|---|---|---|
| C | -2.033606 | -1.055048 | 0.974142 |
| C | -2.322038 | -1.556415 | -0.402667 |
| O | -2.661906 | -1.828966 | 1.860182 |
| C | -2.335541 | -1.611777 | 3.249625 |
| H | -2.968776 | -2.307038 | 3.798870 |
| H | -2.549406 | -0.579756 | 3.537309 |
| H | -1.280727 | -1.838840 | 3.417346 |
| S | -1.462321 | -0.591315 | -1.659538 |
| C | -1.430041 | -1.692566 | -3.089307 |
| H | -0.835544 | -2.559431 | -2.782100 |
| H | -0.940885 | -1.142336 | -3.895903 |
| H | -2.446095 | -1.987005 | -3.356696 |
| C | -2.441821 | 0.817091 | -2.254139 |
| H | -2.510151 | 1.537025 | -1.435573 |
| H | -3.432519 | 0.471988 | -2.552448 |
| H | -1.891394 | 1.244624 | -3.095981 |
| C | -3.775926 | -1.800428 | -0.753884 |
| C | -4.165789 | -3.003944 | -1.361935 |
| C | -4.761351 | -0.838820 | -0.460936 |
| C | -5.504848 | -3.230573 | -1.686064 |
| H | -3.424203 | -3.778388 | -1.533878 |
| C | -6.097318 | -1.070654 | -0.782953 |
| H | -4.494081 | 0.093236 | 0.031741 |
| C | -6.471534 | -2.265432 | -1.403288 |
| H | -5.791276 | -4.171034 | -2.148442 |
| H | -6.844371 | -0.318968 | -0.544160 |
| H | -7.513359 | -2.445747 | -1.653486 |
| C | -0.756253 | -5.250543 | 1.907502 |
| C | -0.272248 | -5.302375 | 3.214612 |
| C | 0.728036 | -4.421684 | 3.634975 |
| C | 1.243127 | -3.491447 | 2.729243 |
| C | 0.763744 | -3.439764 | 1.417622 |
| C | -0.247407 | -4.316717 | 0.987615 |
| H | -1.534623 | -5.935651 | 1.584775 |
| H | -0.677527 | -6.036585 | 3.907379 |
| H | 1.108938 | -4.467119 | 4.651988 |
| H | 2.034870 | -2.811730 | 3.034465 |
| H | 1.190355 | -2.730349 | 0.714747 |
| S | -0.875757 | -4.271208 | -0.685215 |
| H | -1.671387 | -2.735059 | -0.512798 |
| H | 6.495517 | -1.433496 | -2.087802 |
| H | 6.237517 | -3.893265 | -2.182360 |
| H | 3.971092 | -4.915498 | -2.038116 |
| C | 3.208579 | 1.632001 | 2.782000 |
| C | -1.691827 | 3.395619 | 0.412593 |
| C | -1.748973 | 4.627332 | -0.258421 |
| C | -2.893629 | 2.770959 | 0.810195 |
| C | -2.996360 | 5.189739 | -0.500144 |
| H | -0.852784 | 5.143368 | -0.568786 |
| C | -4.104723 | 3.376329 | 0.515754 |
| H | -2.876968 | 1.840382 | 1.365061 |
| C | -4.202706 | 4.595775 | -0.140950 |
| H | -5.156612 | 5.060871 | -0.355591 |
| F | -3.042490 | 6.370181 | -1.146647 |
| F | -5.239332 | 2.733133 | 0.876576 |
| C | 4.668272 | 1.906775 | 3.203914 |
| H | 4.945069 | 2.948248 | 2.993366 |
| H | 4.772873 | 1.753872 | 4.284982 |
| H | 5.367007 | 1.248153 | 2.688609 |
| C | 2.828821 | 0.167933 | 3.083210 |
| H | 3.472068 | -0.528401 | 2.541406 |
| H | 2.932165 | -0.027125 | 4.157562 |

| | | | |
|---|---|---|---|
| H | 1.782315 | -0.039048 | 2.821295 |
| C | 2.299916 | 2.556775 | 3.620285 |
| H | 2.507521 | 3.614585 | 3.430327 |
| H | 1.236721 | 2.382986 | 3.424526 |
| H | 2.473191 | 2.362127 | 4.685261 |

TS Conformation 147

B3LYP/6-31G(d) Energy = -3411.868086
M06-2X/def2tzvpp/IEFPCM(chloroform) Energy = -3411.877716
M06-2X/def2tzvpp/IEFPCM(chloroform)//B3LYP/6-31G(d) Quasiharmonic Free Energy = -3411.151734

Frequencies (Top 3 out of 291)

1. -806.3580 cm-1
2. 10.9503 cm-1
3. 14.0223 cm-1

B3LYP/6-31G(d) Molecular Geometry in Cartesian Coordinates

| | | | |
|---|---|---|---|
| C | 3.877319 | 1.093415 | 0.297325 |
| C | 3.027480 | 1.976938 | 1.257555 |
| H | 3.352693 | 3.014647 | 1.118634 |
| C | 0.804511 | 3.042477 | 0.733000 |
| N | -0.522405 | 2.666456 | 0.664490 |
| H | -0.709841 | 1.694402 | 0.908416 |
| N | 1.620494 | 1.956717 | 0.853237 |
| H | 1.189609 | 1.040197 | 0.778905 |
| C | 4.571605 | 0.465177 | -1.958078 |
| C | 2.980748 | 2.321958 | -1.751248 |
| C | 4.273656 | 1.087266 | -3.342791 |
| H | 5.606930 | 0.680560 | -1.664760 |
| C | 3.820113 | 2.515402 | -3.020431 |
| H | 1.954513 | 2.021080 | -2.001877 |
| H | 2.922765 | 3.223115 | -1.138327 |
| H | 3.461498 | 0.530742 | -3.825804 |
| H | 5.146248 | 1.035813 | -4.000648 |
| H | 3.250649 | 2.985102 | -3.828849 |
| H | 4.684894 | 3.153957 | -2.802716 |
| N | 3.676885 | 1.228138 | -1.050097 |
| O | 4.717531 | 0.301134 | 0.718077 |
| S | 1.362938 | 4.631117 | 0.686912 |
| C | 4.384450 | -1.041776 | -1.927923 |
| C | 3.113720 | -1.625574 | -1.853724 |
| C | 5.501657 | -1.874789 | -2.046504 |
| C | 2.964577 | -3.013203 | -1.889513 |
| H | 2.236791 | -0.992902 | -1.744222 |
| C | 5.356540 | -3.262002 | -2.095705 |
| C | 4.087138 | -3.835327 | -2.015303 |
| H | 1.973702 | -3.453992 | -1.804827 |
| O | -1.300986 | -0.121775 | 1.291198 |
| O | -0.091109 | -0.147812 | -1.328093 |
| C | -2.033529 | -1.054974 | 0.974184 |
| C | -2.322119 | -1.556311 | -0.402601 |
| O | -2.661834 | -1.828828 | 1.860276 |
| C | -2.335317 | -1.611684 | 3.249691 |
| H | -2.968541 | -2.306915 | 3.798986 |
| H | -2.549090 | -0.579655 | 3.537413 |
| H | -1.280501 | -1.838814 | 3.417306 |
| S | -1.462484 | -0.591242 | -1.659555 |
| C | -1.430387 | -1.692498 | -3.089326 |

| | | | |
|---|---|---|---|
| H | -0.941262 | -1.142297 | -3.895961 |
| H | -2.446481 | -1.986874 | -3.356633 |
| H | -0.835916 | -2.559397 | -2.782166 |
| C | -2.441988 | 0.817201 | -2.254057 |
| H | -3.432759 | 0.472157 | -2.552189 |
| H | -1.891672 | 1.244664 | -3.096008 |
| H | -2.510132 | 1.537171 | -1.435507 |
| C | -3.776049 | -1.800276 | -0.753681 |
| C | -4.166009 | -3.003784 | -1.361685 |
| C | -4.761414 | -0.838633 | -0.460647 |
| C | -5.505105 | -3.230371 | -1.685686 |
| H | -3.424463 | -3.778253 | -1.533691 |
| C | -6.097419 | -1.070425 | -0.782536 |
| H | -4.494068 | 0.093417 | 0.031999 |
| C | -6.471733 | -2.265196 | -1.402827 |
| H | -5.791608 | -4.170826 | -2.148029 |
| H | -6.844425 | -0.318713 | -0.543679 |
| H | -7.513588 | -2.445479 | -1.652925 |
| C | 0.763770 | -3.439896 | 1.417462 |
| C | 1.243205 | -3.491615 | 2.729063 |
| C | 0.728013 | -4.421748 | 3.634845 |
| C | -0.272424 | -5.302298 | 3.214552 |
| C | -0.756479 | -5.250430 | 1.907463 |
| C | -0.247532 | -4.316708 | 0.987526 |
| H | 1.190456 | -2.730565 | 0.714548 |
| H | 2.035063 | -2.812007 | 3.034230 |
| H | 1.108953 | -4.467210 | 4.651842 |
| H | -0.677783 | -6.036427 | 3.907359 |
| H | -1.534967 | -5.935430 | 1.584790 |
| S | -0.875951 | -4.271154 | -0.685279 |
| H | -1.671522 | -2.734969 | -0.512797 |
| H | 6.495354 | -1.433690 | -2.088057 |
| H | 6.237257 | -3.893449 | -2.182635 |
| H | 3.970792 | -4.915594 | -2.038390 |
| C | 3.208806 | 1.631805 | 2.781881 |
| C | -1.691654 | 3.395661 | 0.412730 |
| C | -1.748794 | 4.627402 | -0.258233 |
| C | -2.893457 | 2.771022 | 0.810363 |
| C | -2.996175 | 5.189855 | -0.499879 |
| H | -0.852603 | 5.143422 | -0.568618 |
| C | -4.104546 | 3.376438 | 0.515999 |
| H | -2.876797 | 1.840425 | 1.365196 |
| C | -4.202522 | 4.595911 | -0.140655 |
| H | -5.156424 | 5.061043 | -0.355236 |
| F | -3.042301 | 6.370322 | -1.146334 |
| F | -5.239158 | 2.733259 | 0.876847 |
| C | 2.300200 | 2.556566 | 3.620240 |
| H | 1.236992 | 2.382789 | 3.424541 |
| H | 2.473537 | 2.361895 | 4.685202 |
| H | 2.507801 | 3.614379 | 3.430292 |
| C | 4.668523 | 1.906535 | 3.203736 |
| H | 4.945320 | 2.948020 | 2.993244 |
| H | 4.773179 | 1.753559 | 4.284789 |
| H | 5.367225 | 1.247941 | 2.688351 |
| C | 2.829035 | 0.167733 | 3.083054 |
| H | 1.782519 | -0.039227 | 2.821163 |
| H | 3.472256 | -0.528593 | 2.541208 |
| H | 2.932408 | -0.027362 | 4.157397 |

TS Conformation 148

B3LYP/6-31G(d) Energy = -3411.863591
M06-2X/def2tzvpp/IEFPCM(chloroform) Energy = -3411.875804
M06-2X/def2tzvpp/IEFPCM(chloroform)//B3LYP/6-31G(d) Quasiharmonic Free Energy = -3411.1499

Frequencies (Top 3 out of 291)

1. -660.5971 cm-1
2. 9.0795 cm-1
3. 14.7480 cm-1

B3LYP/6-31G(d) Molecular Geometry in Cartesian Coordinates

| | | | |
|---|---|---|---|
| C | 4.311080 | -1.374690 | 1.662591 |
| C | 2.916750 | -0.882680 | 2.128301 |
| H | 3.189160 | -0.203200 | 2.939581 |
| C | 2.200280 | 1.282661 | 1.116891 |
| N | 1.111720 | 1.800731 | 0.448101 |
| H | 0.320240 | 1.171951 | 0.341141 |
| N | 2.158890 | -0.087039 | 1.165761 |
| H | 1.346860 | -0.528539 | 0.749361 |
| C | 3.755330 | -1.840070 | -0.822639 |
| C | 5.966180 | -2.243420 | 0.119751 |
| C | 4.389020 | -3.083470 | -1.495369 |
| H | 2.732330 | -2.056039 | -0.507449 |
| C | 5.890920 | -2.839770 | -1.293899 |
| H | 6.733670 | -1.470370 | 0.224701 |
| H | 6.167250 | -2.999310 | 0.885241 |
| H | 4.105130 | -3.194370 | -2.545039 |
| H | 4.071090 | -3.984950 | -0.956629 |
| H | 6.254560 | -2.123460 | -2.038419 |
| H | 6.488470 | -3.751120 | -1.387199 |
| N | 4.617450 | -1.676940 | 0.358111 |
| O | 5.172590 | -1.503200 | 2.529581 |
| S | 3.432241 | 2.183700 | 1.811101 |
| C | 3.740770 | -0.633180 | -1.766249 |
| C | 4.406110 | 0.558690 | -1.468699 |
| C | 3.048610 | -0.725330 | -2.985099 |
| C | 4.378400 | 1.632750 | -2.363439 |
| H | 4.931470 | 0.656470 | -0.524979 |
| C | 3.016650 | 0.345910 | -3.878799 |
| C | 3.684610 | 1.533430 | -3.568659 |
| H | 4.902841 | 2.550050 | -2.110019 |
| O | -3.456320 | -1.595269 | -3.509609 |
| O | -0.970160 | -0.304039 | -0.214059 |
| C | -3.785340 | -2.035339 | -2.410999 |
| C | -3.191020 | -1.595959 | -1.119319 |
| O | -4.794120 | -2.889668 | -2.223369 |
| C | -5.551560 | -3.239478 | -3.399699 |
| H | -6.318790 | -3.928388 | -3.048509 |
| H | -4.907740 | -3.719368 | -4.140919 |
| H | -6.004590 | -2.345938 | -3.834669 |
| S | -1.702720 | -0.618029 | -1.472479 |
| C | -0.571640 | -1.466129 | -2.602189 |
| H | 0.292530 | -0.812189 | -2.749679 |
| H | -0.271910 | -2.386069 | -2.094469 |
| H | -1.104120 | -1.676329 | -3.529459 |
| C | -2.211640 | 0.887291 | -2.323959 |
| H | -2.454930 | 0.632791 | -3.355069 |
| H | -3.110640 | 1.240531 | -1.794399 |
| H | -1.377840 | 1.588561 | -2.238649 |
| C | -2.936750 | -2.591059 | -0.024879 |
| C | -2.568510 | -3.917779 | -0.307919 |

| | | | |
|---|---|---|---|
| C | -3.056230 | -2.188669 | 1.314011 |
| C | -2.339560 | -4.825179 | 0.724261 |
| H | -2.488610 | -4.249529 | -1.340039 |
| C | -2.818880 | -3.097659 | 2.345271 |
| H | -3.346140 | -1.169099 | 1.545271 |
| C | -2.462290 | -4.415369 | 2.054551 |
| H | -2.068261 | -5.851079 | 0.490951 |
| H | -2.922440 | -2.774439 | 3.377071 |
| H | -2.287861 | -5.123919 | 2.859741 |
| C | -4.133329 | 2.644961 | 1.105041 |
| C | -3.777239 | 3.268531 | 2.302021 |
| C | -4.039899 | 2.651041 | 3.526931 |
| C | -4.674630 | 1.407162 | 3.538781 |
| C | -5.036800 | 0.784072 | 2.342941 |
| C | -4.762580 | 1.385872 | 1.100171 |
| H | -3.938619 | 3.147391 | 0.162351 |
| H | -3.294509 | 4.241841 | 2.267071 |
| H | -3.761829 | 3.136161 | 4.458721 |
| H | -4.900250 | 0.920922 | 4.485441 |
| H | -5.554180 | -0.171288 | 2.361191 |
| S | -5.246030 | 0.610012 | -0.433299 |
| H | -4.030220 | -0.658769 | -0.699369 |
| H | 2.547230 | -1.654659 | -3.250769 |
| H | 2.485400 | 0.248691 | -4.822729 |
| H | 3.666641 | 2.369190 | -4.262649 |
| C | 2.023860 | -2.010869 | 2.766791 |
| C | 0.808031 | 3.137001 | 0.121871 |
| C | 1.769131 | 4.053881 | -0.323549 |
| C | -0.544989 | 3.509541 | 0.186441 |
| C | 1.346781 | 5.328771 | -0.673959 |
| H | 2.814761 | 3.791450 | -0.387459 |
| C | -0.901389 | 4.798361 | -0.185599 |
| H | -1.302809 | 2.832701 | 0.567081 |
| C | 0.018861 | 5.741591 | -0.622809 |
| H | -0.278169 | 6.745301 | -0.900269 |
| F | 2.270161 | 6.210431 | -1.106749 |
| F | -2.203089 | 5.154641 | -0.103289 |
| C | 1.576510 | -3.069289 | 1.738361 |
| H | 2.434070 | -3.583399 | 1.289021 |
| H | 0.956070 | -3.827729 | 2.228861 |
| H | 0.961270 | -2.645569 | 0.933521 |
| C | 0.776340 | -1.349049 | 3.392521 |
| H | 0.112820 | -0.902029 | 2.645071 |
| H | 1.061490 | -0.565749 | 4.103831 |
| H | 0.191450 | -2.100349 | 3.935241 |
| C | 2.806150 | -2.717580 | 3.893081 |
| H | 3.149840 | -2.001530 | 4.646231 |
| H | 3.688190 | -3.242600 | 3.520191 |
| H | 2.151200 | -3.446089 | 4.386021 |

TS Conformation 149

B3LYP/6-31G(d) Energy = -3411.861203
M06-2X/def2tzvpp/IEFPCM(chloroform) Energy = -3411.873172
M06-2X/def2tzvpp/IEFPCM(chloroform)//B3LYP/6-31G(d) Quasiharmonic Free Energy = -3411.149017

Frequencies (Top 3 out of 291)

1. -770.9992 cm-1
2. 6.6903 cm-1
3. 11.1170 cm-1

B3LYP/6-31G(d) Molecular Geometry in Cartesian Coordinates

| | | | |
|---|---|---|---|
| C | 3.648353 | -3.005935 | 0.838921 |
| C | 3.227055 | -1.773769 | 1.676750 |
| H | 4.180052 | -1.421088 | 2.077109 |
| C | 3.355173 | 0.525939 | 0.677463 |
| N | 2.577841 | 1.372344 | -0.091564 |
| H | 1.866060 | 0.902674 | -0.642675 |
| N | 2.695058 | -0.654102 | 0.910121 |
| H | 1.694665 | -0.639371 | 0.757760 |
| C | 1.836633 | -2.825878 | -0.998873 |
| C | 3.501694 | -4.619320 | -0.965429 |
| C | 1.273165 | -4.091532 | -1.696638 |
| H | 1.115297 | -2.479471 | -0.256423 |
| C | 2.537950 | -4.838315 | -2.139048 |
| H | 4.549736 | -4.539504 | -1.269275 |
| H | 3.447346 | -5.423960 | -0.223380 |
| H | 0.717249 | -4.687037 | -0.961169 |
| H | 0.599417 | -3.841956 | -2.521208 |
| H | 2.936043 | -4.390356 | -3.055953 |
| H | 2.362896 | -5.900158 | -2.335075 |
| N | 3.042610 | -3.357072 | -0.342948 |
| O | 4.566422 | -3.699449 | 1.272382 |
| S | 4.906450 | 0.852971 | 1.218627 |
| C | 2.096487 | -1.706277 | -2.005701 |
| C | 1.007573 | -0.949564 | -2.468129 |
| C | 3.367237 | -1.441629 | -2.527248 |
| C | 1.183448 | 0.029283 | -3.449068 |
| H | 0.018395 | -1.125348 | -2.050941 |
| C | 3.544867 | -0.458686 | -3.503959 |
| C | 2.455009 | 0.274387 | -3.974592 |
| H | 0.326519 | 0.602698 | -3.793980 |
| O | -5.855218 | -1.907851 | -1.136380 |
| O | -1.610369 | -1.707970 | -0.225001 |
| C | -5.389721 | -0.773515 | -1.048440 |
| C | -4.022620 | -0.462625 | -0.557761 |
| O | -6.095093 | 0.336689 | -1.285664 |
| C | -7.491255 | 0.142683 | -1.585924 |
| H | -7.993257 | -0.341485 | -0.745239 |
| H | -7.609617 | -0.470633 | -2.482631 |
| H | -7.889049 | 1.144104 | -1.746640 |
| S | -3.035564 | -1.983066 | -0.529487 |
| C | -3.145147 | -2.882317 | -2.100946 |
| H | -4.194511 | -3.092769 | -2.306925 |
| H | -2.708735 | -2.227560 | -2.858607 |
| H | -2.550348 | -3.793135 | -1.996452 |
| C | -3.749519 | -3.094310 | 0.696860 |
| H | -3.818537 | -2.504956 | 1.622077 |
| H | -4.738865 | -3.396751 | 0.354061 |
| H | -3.055802 | -3.932638 | 0.794721 |
| C | -3.241597 | 0.673976 | -1.154745 |
| C | -3.323990 | 0.984597 | -2.523439 |
| C | -2.399633 | 1.440568 | -0.335101 |
| C | -2.591108 | 2.043964 | -3.056660 |
| H | -3.986616 | 0.413726 | -3.169229 |
| C | -1.664531 | 2.497408 | -0.872362 |
| H | -2.332181 | 1.218925 | 0.724107 |
| C | -1.755307 | 2.802985 | -2.231855 |
| H | -2.677116 | 2.280981 | -4.113756 |
| H | -1.024407 | 3.087617 | -0.223556 |
| H | -1.176420 | 3.626075 | -2.641812 |

| | | | |
|---|---|---|---|
| C | -2.205827 | 0.604015 | 3.618253 |
| C | -1.295415 | 1.529400 | 4.133072 |
| C | -1.603435 | 2.891127 | 4.148559 |
| C | -2.836188 | 3.316814 | 3.647447 |
| C | -3.747812 | 2.394597 | 3.133757 |
| C | -3.448062 | 1.019873 | 3.105239 |
| H | -1.964292 | -0.455309 | 3.619509 |
| H | -0.344274 | 1.182189 | 4.529950 |
| H | -0.894867 | 3.610254 | 4.550793 |
| H | -3.092119 | 4.373876 | 3.658491 |
| H | -4.706854 | 2.729947 | 2.750074 |
| S | -4.629727 | -0.152703 | 2.458925 |
| H | -4.210872 | -0.250890 | 0.756819 |
| H | 4.224651 | -1.991937 | -2.152574 |
| H | 4.540900 | -0.262027 | -3.890773 |
| H | 2.595955 | 1.038746 | -4.733589 |
| C | 2.320596 | -2.132982 | 2.913925 |
| C | 2.685774 | 2.741550 | -0.384788 |
| C | 1.893116 | 3.203485 | -1.451874 |
| C | 3.454744 | 3.654416 | 0.350853 |
| C | 1.879969 | 4.555828 | -1.748460 |
| H | 1.295967 | 2.520438 | -2.044117 |
| C | 3.407172 | 4.995082 | -0.010072 |
| H | 4.081310 | 3.339212 | 1.171438 |
| C | 2.629954 | 5.492891 | -1.050558 |
| H | 2.612044 | 6.546503 | -1.299143 |
| F | 1.090699 | 4.978505 | -2.765503 |
| F | 4.148093 | 5.867734 | 0.700284 |
| C | 0.958758 | -2.721902 | 2.493547 |
| H | 0.353039 | -2.025698 | 1.898895 |
| H | 1.080117 | -3.649290 | 1.920752 |
| H | 0.368692 | -2.963278 | 3.385530 |
| C | 2.090254 | -0.847367 | 3.735720 |
| H | 3.041192 | -0.382616 | 4.017553 |
| H | 1.544557 | -1.089208 | 4.655352 |
| H | 1.502162 | -0.102620 | 3.189213 |
| C | 3.049241 | -3.155632 | 3.809879 |
| H | 4.022492 | -2.773085 | 4.134753 |
| H | 3.227039 | -4.103354 | 3.297189 |
| H | 2.444435 | -3.351273 | 4.703517 |

TS Conformation 150

B3LYP/6-31G(d) Energy = -3411.863507
M06-2X/def2tzvpp/IEFPCM(chloroform) Energy = -3411.874364
M06-2X/def2tzvpp/IEFPCM(chloroform)//B3LYP/6-31G(d) Quasiharmonic Free Energy = -3411.148774

Frequencies (Top 3 out of 291)

1. -858.7381 cm-1
2. 10.4542 cm-1
3. 12.5717 cm-1

B3LYP/6-31G(d) Molecular Geometry in Cartesian Coordinates

| | | | |
|---|---|---|---|
| C | -4.049015 | -2.233601 | -0.781258 |
| C | -2.775175 | -1.792713 | -1.551535 |
| H | -3.203396 | -1.511192 | -2.516600 |
| C | -2.375099 | 0.668926 | -1.490147 |
| N | -1.370453 | 1.548413 | -1.140712 |
| H | -0.515628 | 1.113128 | -0.805303 |

| | | | |
|---|---|---|---|
| N | -2.109913 | -0.596693 | -1.045446 |
| H | -1.256000 | -0.728528 | -0.514790 |
| C | -3.224928 | -1.738049 | 1.602851 |
| C | -5.444107 | -2.495026 | 1.210176 |
| C | -3.554001 | -2.829988 | 2.686330 |
| H | -2.235110 | -1.924916 | 1.186380 |
| C | -4.872940 | -3.492148 | 2.224503 |
| H | -5.976937 | -1.677864 | 1.713232 |
| H | -6.092119 | -2.934701 | 0.454778 |
| H | -3.674944 | -2.364791 | 3.667961 |
| H | -2.741219 | -3.558637 | 2.767805 |
| H | -5.555870 | -3.689447 | 3.056633 |
| H | -4.673194 | -4.445253 | 1.722656 |
| N | -4.239659 | -1.955334 | 0.559229 |
| O | -4.917722 | -2.800439 | -1.436022 |
| S | -3.750876 | 1.062452 | -2.367807 |
| C | -3.233569 | -0.318679 | 2.178098 |
| C | -2.499130 | -0.037649 | 3.341486 |
| C | -3.933621 | 0.725621 | 1.566176 |
| C | -2.463932 | 1.251570 | 3.878283 |
| H | -1.970787 | -0.840112 | 3.853448 |
| C | -3.901341 | 2.016328 | 2.100746 |
| C | -3.167163 | 2.287128 | 3.255888 |
| H | -1.908315 | 1.440878 | 4.794086 |
| O | 3.696365 | 1.274145 | 3.251835 |
| O | 0.660175 | -0.043213 | 0.448664 |
| C | 4.088236 | 1.087201 | 2.101942 |
| C | 3.265484 | 0.485649 | 1.021593 |
| O | 5.343182 | 1.311284 | 1.700980 |
| C | 6.264921 | 1.735680 | 2.724785 |
| H | 7.216658 | 1.870066 | 2.211986 |
| H | 5.931308 | 2.673067 | 3.176540 |
| H | 6.348218 | 0.968431 | 3.497806 |
| S | 1.535655 | 0.435707 | 1.555721 |
| C | 1.405118 | -0.668965 | 2.971129 |
| H | 1.949815 | -0.221620 | 3.802281 |
| H | 0.336778 | -0.775816 | 3.171242 |
| H | 1.875014 | -1.611111 | 2.657068 |
| C | 0.929246 | 2.037727 | 2.144230 |
| H | -0.095345 | 1.887151 | 2.497795 |
| H | 1.597000 | 2.378070 | 2.936156 |
| H | 0.940798 | 2.711826 | 1.283878 |
| C | 3.376507 | 0.996643 | -0.386445 |
| C | 3.632949 | 2.352249 | -0.657257 |
| C | 3.198293 | 0.112040 | -1.461463 |
| C | 3.720192 | 2.810352 | -1.970770 |
| H | 3.780066 | 3.050873 | 0.161938 |
| C | 3.281508 | 0.573569 | -2.775550 |
| H | 3.009827 | -0.938061 | -1.267753 |
| C | 3.541504 | 1.920435 | -3.033322 |
| H | 3.905759 | 3.862822 | -2.161901 |
| H | 3.147334 | -0.124918 | -3.596435 |
| H | 3.604683 | 2.277683 | -4.057404 |
| C | 2.826734 | -4.430234 | -0.129337 |
| C | 2.652207 | -5.193016 | -1.285131 |
| C | 3.426628 | -4.945726 | -2.421027 |
| C | 4.389412 | -3.934620 | -2.380334 |
| C | 4.575973 | -3.179873 | -1.221362 |
| C | 3.790127 | -3.406676 | -0.076014 |
| H | 2.223163 | -4.629843 | 0.751396 |
| H | 1.909496 | -5.987922 | -1.295651 |
| H | 3.289621 | -5.539491 | -3.320849 |

| | | | |
|---|---|---|---|
| H | 5.010186 | -3.739048 | -3.251848 |
| H | 5.342473 | -2.410844 | -1.190937 |
| S | 4.042485 | -2.477653 | 1.427197 |
| H | 3.595985 | -0.835525 | 1.065590 |
| H | -4.498327 | 0.527750 | 0.660952 |
| H | -4.454582 | 2.811467 | 1.608637 |
| H | -3.151621 | 3.289731 | 3.674709 |
| C | -1.746141 | -2.948388 | -1.842550 |
| C | -1.267673 | 2.941757 | -1.277829 |
| C | -2.362908 | 3.817010 | -1.277943 |
| C | 0.039463 | 3.460699 | -1.348238 |
| C | -2.114467 | 5.181002 | -1.356756 |
| H | -3.378541 | 3.452826 | -1.238289 |
| C | 0.217750 | 4.834993 | -1.415493 |
| H | 0.903301 | 2.807609 | -1.405536 |
| C | -0.839689 | 5.734743 | -1.421526 |
| H | -0.683617 | 6.804426 | -1.483586 |
| F | -3.169204 | 6.019265 | -1.351394 |
| F | 1.479346 | 5.316424 | -1.486744 |
| C | -0.661275 | -2.401570 | -2.796507 |
| H | 0.026764 | -3.206836 | -3.076828 |
| H | -1.108766 | -2.000436 | -3.713214 |
| H | -0.061379 | -1.608144 | -2.338490 |
| C | -2.466035 | -4.114115 | -2.551492 |
| H | -1.730354 | -4.879546 | -2.825568 |
| H | -3.226538 | -4.577332 | -1.918906 |
| H | -2.965409 | -3.776353 | -3.465309 |
| C | -1.068165 | -3.490169 | -0.568424 |
| H | -0.495870 | -2.723278 | -0.030822 |
| H | -1.796446 | -3.933990 | 0.120663 |
| H | -0.348558 | -4.271197 | -0.836307 |

TS Conformation 151

B3LYP/6-31G(d) Energy = -3411.860035
M06-2X/def2tzvpp/IEFPCM(chloroform) Energy = -3411.873459
M06-2X/def2tzvpp/IEFPCM(chloroform)//B3LYP/6-31G(d) Quasiharmonic Free Energy = -3411.148439

Frequencies (Top 3 out of 291)

1. -784.9007 cm-1
2. 7.3794 cm-1
3. 10.5730 cm-1

B3LYP/6-31G(d) Molecular Geometry in Cartesian Coordinates

| | | | |
|---|---|---|---|
| C | -4.512583 | 0.784651 | 0.539442 |
| C | -3.245383 | 0.210859 | 1.232460 |
| H | -3.116784 | -0.789756 | 0.799955 |
| C | -1.713598 | 2.232955 | 0.752487 |
| N | -0.340731 | 2.435601 | 0.787021 |
| H | 0.203835 | 1.618873 | 1.051654 |
| N | -1.993950 | 0.898417 | 0.859749 |
| H | -1.190942 | 0.276870 | 0.849281 |
| C | -3.589751 | 0.041922 | -1.726028 |
| C | -5.715411 | 1.256334 | -1.529535 |
| C | -3.982479 | 0.660872 | -3.092788 |
| H | -2.582175 | 0.365380 | -1.451684 |
| C | -5.504576 | 0.836371 | -2.990167 |
| H | -6.660828 | 0.907140 | -1.104597 |
| H | -5.681114 | 2.345184 | -1.397867 |

| | | | |
|---|---|---|---|
| H | -3.668151 | 0.034993 | -3.932642 |
| H | -3.497549 | 1.639472 | -3.190648 |
| H | -6.008724 | -0.115402 | -3.192467 |
| H | -5.895786 | 1.576494 | -3.694410 |
| N | -4.577269 | 0.648425 | -0.819101 |
| O | -5.461307 | 1.248270 | 1.164647 |
| S | -2.857295 | 3.442681 | 0.590752 |
| C | -3.617884 | -1.486313 | -1.733721 |
| C | -4.763891 | -2.215935 | -1.392146 |
| C | -2.466376 | -2.186858 | -2.121156 |
| C | -4.762778 | -3.611561 | -1.448809 |
| H | -5.655597 | -1.690991 | -1.062075 |
| C | -2.463358 | -3.581291 | -2.177225 |
| C | -3.614239 | -4.298992 | -1.842858 |
| H | -5.660926 | -4.160756 | -1.178024 |
| O | 1.139236 | -0.086912 | 0.792817 |
| O | 0.818288 | -1.038610 | -2.008459 |
| C | 2.238174 | -0.643118 | 0.794900 |
| C | 3.084800 | -0.908407 | -0.401432 |
| O | 2.829660 | -1.120754 | 1.887262 |
| C | 2.062966 | -1.093853 | 3.108391 |
| H | 2.724647 | -1.510819 | 3.866230 |
| H | 1.781484 | -0.069396 | 3.365129 |
| H | 1.170262 | -1.712571 | 2.995756 |
| S | 2.179896 | -0.478490 | -1.897946 |
| C | 2.128052 | 1.307040 | -2.220017 |
| H | 3.138007 | 1.717633 | -2.182449 |
| H | 1.496818 | 1.745315 | -1.445608 |
| H | 1.675446 | 1.439001 | -3.205991 |
| C | 3.230362 | -1.131675 | -3.209575 |
| H | 4.224935 | -0.686617 | -3.161573 |
| H | 2.728180 | -0.906263 | -4.152811 |
| H | 3.268185 | -2.212527 | -3.033016 |
| C | 4.492454 | -0.345994 | -0.366467 |
| C | 5.596508 | -1.160055 | -0.662960 |
| C | 4.717478 | 0.995455 | -0.009434 |
| C | 6.889141 | -0.634165 | -0.619379 |
| H | 5.441794 | -2.211126 | -0.885538 |
| C | 6.008486 | 1.518143 | 0.035107 |
| H | 3.881524 | 1.639102 | 0.247713 |
| C | 7.098849 | 0.702956 | -0.276899 |
| H | 7.735182 | -1.277929 | -0.843304 |
| H | 6.150810 | 2.556771 | 0.318992 |
| H | 8.107582 | 1.105207 | -0.242171 |
| C | 0.746800 | -3.879993 | 0.222105 |
| C | -0.242682 | -4.172522 | 1.164701 |
| C | 0.062041 | -4.924550 | 2.301465 |
| C | 1.370342 | -5.382196 | 2.484776 |
| C | 2.361891 | -5.086196 | 1.549929 |
| C | 2.068035 | -4.328094 | 0.401558 |
| H | 0.492618 | -3.309946 | -0.666057 |
| H | -1.260143 | -3.829041 | 0.991451 |
| H | -0.710512 | -5.161052 | 3.028566 |
| H | 1.621183 | -5.974277 | 3.362207 |
| H | 3.378065 | -5.438907 | 1.700020 |
| S | 3.350296 | -3.954046 | -0.782635 |
| H | 3.187635 | -2.244407 | -0.561069 |
| H | -1.559904 | -1.639035 | -2.367397 |
| H | -1.557871 | -4.106806 | -2.468296 |
| H | -3.610955 | -5.384959 | -1.879862 |
| C | -3.381098 | -0.021046 | 2.777656 |
| C | 0.462147 | 3.553177 | 0.529908 |

| | | | |
|---|---|---|---|
| C | 0.052687 | 4.712692 | -0.146924 |
| C | 1.806886 | 3.436822 | 0.943832 |
| C | 0.996128 | 5.704629 | -0.385604 |
| H | -0.970997 | 4.847828 | -0.462550 |
| C | 2.699982 | 4.455253 | 0.655277 |
| H | 2.144196 | 2.574372 | 1.507787 |
| C | 2.333949 | 5.616191 | -0.012611 |
| H | 3.041236 | 6.408523 | -0.222873 |
| F | 0.596116 | 6.811888 | -1.039114 |
| F | 3.991114 | 4.297671 | 1.034244 |
| C | -3.564869 | 1.286566 | 3.569523 |
| H | -3.643084 | 1.058090 | 4.640242 |
| H | -2.711500 | 1.960157 | 3.436608 |
| H | -4.466443 | 1.810955 | 3.251622 |
| C | -2.099555 | -0.728895 | 3.271894 |
| H | -2.204438 | -0.985004 | 4.332261 |
| H | -1.906576 | -1.661553 | 2.724437 |
| H | -1.214339 | -0.089568 | 3.177388 |
| C | -4.573077 | -0.968682 | 3.032991 |
| H | -5.518460 | -0.497712 | 2.759689 |
| H | -4.469666 | -1.902164 | 2.463394 |
| H | -4.612345 | -1.235164 | 4.095884 |

TS Conformation 152

B3LYP/6-31G(d) Energy = -3411.863985
M06-2X/def2tzvpp/IEFPCM(chloroform) Energy = -3411.878929
M06-2X/def2tzvpp/IEFPCM(chloroform)//B3LYP/6-31G(d) Quasiharmonic Free Energy = -3411.153564

Frequencies (Top 3 out of 291)

1. -792.5906 cm-1
2. 4.9331 cm-1
3. 14.6057 cm-1

B3LYP/6-31G(d) Molecular Geometry in Cartesian Coordinates

| | | | |
|---|---|---|---|
| C | 3.505409 | 1.798810 | 0.545700 |
| C | 2.369159 | 1.923040 | 1.595820 |
| H | 2.515829 | 2.936050 | 1.977510 |
| C | 0.337269 | 2.958970 | 0.570070 |
| N | -0.876471 | 2.573210 | 0.013360 |
| H | -0.943491 | 1.589920 | -0.241960 |
| N | 1.013329 | 1.882960 | 1.057400 |
| H | 0.501329 | 1.010770 | 1.134980 |
| C | 4.492059 | 1.138600 | -1.575330 |
| C | 2.153389 | 0.481780 | -1.212780 |
| C | 3.822939 | 0.763110 | -2.919330 |
| H | 4.874749 | 2.162560 | -1.598060 |
| C | 2.671119 | -0.161850 | -2.506960 |
| H | 1.700689 | -0.254750 | -0.544040 |
| H | 1.403009 | 1.246010 | -1.443240 |
| H | 4.527799 | 0.297920 | -3.613440 |
| H | 3.426449 | 1.669500 | -3.393180 |
| H | 3.037619 | -1.174150 | -2.308090 |
| H | 1.884759 | -0.233870 | -3.265450 |
| N | 3.360609 | 1.091080 | -0.620570 |
| O | 4.573809 | 2.346590 | 0.808930 |
| S | 0.934439 | 4.526790 | 0.591840 |
| C | 5.652799 | 0.220680 | -1.209420 |
| C | 6.955979 | 0.611660 | -1.541600 |

| | | | |
|---|---|---|---|
| C | 5.465319 | -1.029870 | -0.607950 |
| C | 8.043499 | -0.224580 | -1.290300 |
| H | 7.120429 | 1.588220 | -1.991940 |
| C | 6.551989 | -1.868640 | -0.351590 |
| C | 7.844949 | -1.471400 | -0.694440 |
| H | 9.047059 | 0.102630 | -1.550090 |
| O | -1.399151 | -0.231870 | -1.014000 |
| O | -1.214411 | -0.330290 | 1.873840 |
| C | -2.087471 | -1.248360 | -1.017720 |
| C | -2.804071 | -1.843180 | 0.144990 |
| O | -2.256321 | -2.016830 | -2.099800 |
| C | -1.510921 | -1.637470 | -3.274000 |
| H | -0.440401 | -1.742700 | -3.081830 |
| H | -1.832081 | -2.331550 | -4.049950 |
| H | -1.737061 | -0.607560 | -3.559060 |
| S | -2.591821 | -0.795630 | 1.602700 |
| C | -3.737921 | 0.610580 | 1.604420 |
| H | -3.451311 | 1.271260 | 0.782660 |
| H | -3.610411 | 1.119510 | 2.563160 |
| H | -4.757411 | 0.244480 | 1.474530 |
| C | -3.167981 | -1.819370 | 2.969620 |
| H | -2.496671 | -2.686660 | 2.983040 |
| H | -4.203921 | -2.119730 | 2.807030 |
| H | -3.055791 | -1.215260 | 3.872620 |
| C | -4.246551 | -2.247000 | -0.076840 |
| C | -4.719391 | -3.486230 | 0.382800 |
| C | -5.132331 | -1.397930 | -0.766330 |
| C | -6.049231 | -3.856280 | 0.173510 |
| H | -4.035581 | -4.175940 | 0.866960 |
| C | -6.458101 | -1.772080 | -0.976590 |
| H | -4.780971 | -0.445170 | -1.156940 |
| C | -6.921631 | -3.001020 | -0.500100 |
| H | -6.397391 | -4.822230 | 0.528190 |
| H | -7.126141 | -1.106710 | -1.516120 |
| H | -7.955021 | -3.293440 | -0.664130 |
| C | 1.405389 | -4.064240 | 1.082550 |
| C | 2.631869 | -3.977770 | 0.422300 |
| C | 2.690979 | -4.004500 | -0.973570 |
| C | 1.504269 | -4.126240 | -1.701750 |
| C | 0.276529 | -4.222820 | -1.044650 |
| C | 0.205639 | -4.189460 | 0.360520 |
| H | 1.367789 | -4.043820 | 2.167570 |
| H | 3.546889 | -3.894020 | 1.004250 |
| H | 3.648079 | -3.944130 | -1.484700 |
| H | 1.536139 | -4.168370 | -2.788530 |
| H | -0.639741 | -4.345410 | -1.614760 |
| S | -1.354211 | -4.355250 | 1.208990 |
| H | -2.129381 | -2.940120 | 0.543610 |
| H | 4.466569 | -1.349500 | -0.325470 |
| H | 6.386919 | -2.833490 | 0.121980 |
| H | 8.690769 | -2.122950 | -0.491390 |
| C | 2.530779 | 0.940830 | 2.811620 |
| C | -1.970031 | 3.336790 | -0.414910 |
| C | -2.837391 | 2.722970 | -1.343030 |
| C | -2.288121 | 4.609420 | 0.085660 |
| C | -3.988961 | 3.387730 | -1.737710 |
| H | -2.595951 | 1.758880 | -1.775110 |
| C | -3.447351 | 5.225760 | -0.365990 |
| H | -1.645581 | 5.116440 | 0.790130 |
| C | -4.332891 | 4.649360 | -1.272260 |
| H | -5.230681 | 5.159250 | -1.598290 |
| F | -4.810971 | 2.769870 | -2.615810 |

| | | | |
|---|---|---|---|
| F | -3.747091 | 6.445760 | 0.120010 |
| C | 3.913209 | 1.149150 | 3.464100 |
| H | 4.055289 | 2.190960 | 3.770590 |
| H | 3.988999 | 0.516470 | 4.356830 |
| H | 4.731959 | 0.894220 | 2.788450 |
| C | 2.399109 | -0.535810 | 2.388450 |
| H | 3.159869 | -0.808720 | 1.649940 |
| H | 2.540649 | -1.185100 | 3.260630 |
| H | 1.414309 | -0.781080 | 1.974410 |
| C | 1.449859 | 1.279870 | 3.860910 |
| H | 1.509359 | 2.332840 | 4.160290 |
| H | 0.436779 | 1.087390 | 3.494370 |
| H | 1.600189 | 0.666300 | 4.757130 |

TS Conformation 153

B3LYP/6-31G(d) Energy = -3411.868775
M06-2X/def2tzvpp/IEFPCM(chloroform) Energy = -3411.877151
M06-2X/def2tzvpp/IEFPCM(chloroform)//B3LYP/6-31G(d) Quasiharmonic Free Energy = -3411.153343

Frequencies (Top 3 out of 291)

1. -794.7262 cm-1
2. 5.4102 cm-1
3. 6.6553 cm-1

B3LYP/6-31G(d) Molecular Geometry in Cartesian Coordinates

| | | | |
|---|---|---|---|
| C | 4.272280 | -0.010553 | 0.040984 |
| C | 3.801889 | 1.161096 | 0.954038 |
| H | 4.332792 | 2.068103 | 0.643340 |
| C | 1.832294 | 2.639117 | 0.375598 |
| N | 0.473768 | 2.531812 | 0.148918 |
| H | 0.120117 | 1.593044 | -0.011403 |
| N | 2.388190 | 1.433788 | 0.686893 |
| H | 1.760988 | 0.650614 | 0.833573 |
| C | 4.424455 | -1.043789 | -2.168025 |
| C | 3.560558 | 1.248039 | -2.062060 |
| C | 4.172461 | -0.487734 | -3.589708 |
| H | 5.501107 | -1.170195 | -1.999032 |
| C | 4.253182 | 1.033044 | -3.413325 |
| H | 2.470003 | 1.287819 | -2.188414 |
| H | 3.867255 | 2.169735 | -1.564667 |
| H | 3.169180 | -0.777850 | -3.924399 |
| H | 4.890516 | -0.889792 | -4.310394 |
| H | 3.771188 | 1.592222 | -4.221250 |
| H | 5.298754 | 1.358607 | -3.353744 |
| N | 3.950468 | 0.054543 | -1.290059 |
| O | 4.892336 | -0.976345 | 0.478738 |
| S | 2.706857 | 4.067925 | 0.229301 |
| C | 3.753702 | -2.383632 | -1.917011 |
| C | 4.502330 | -3.558872 | -2.040383 |
| C | 2.388763 | -2.484646 | -1.619166 |
| C | 3.905349 | -4.809295 | -1.874968 |
| H | 5.566957 | -3.492988 | -2.254165 |
| C | 1.790287 | -3.732985 | -1.437119 |
| C | 2.547373 | -4.899881 | -1.568702 |
| H | 4.506021 | -5.710348 | -1.969232 |
| O | -4.900789 | -2.093719 | -2.124166 |
| O | -1.063638 | -0.147467 | -1.491441 |
| C | -4.677480 | -1.735041 | -0.969973 |

| | | | |
|---|---|---|---|
| C | -3.371152 | -1.218764 | -0.488510 |
| O | -5.556861 | -1.863450 | 0.028649 |
| C | -6.791832 | -2.527878 | -0.303876 |
| H | -6.591757 | -3.547998 | -0.639373 |
| H | -7.369568 | -2.532756 | 0.619813 |
| H | -7.321159 | -1.983492 | -1.089728 |
| S | -2.323702 | -0.813673 | -1.910794 |
| C | -1.955503 | -2.341157 | -2.794594 |
| H | -2.887235 | -2.744414 | -3.190537 |
| H | -1.235459 | -2.082104 | -3.574235 |
| H | -1.514914 | -3.013759 | -2.049524 |
| C | -3.186224 | 0.222180 | -3.126052 |
| H | -2.504080 | 0.362923 | -3.968557 |
| H | -4.099708 | -0.295306 | -3.419921 |
| H | -3.399482 | 1.181370 | -2.648294 |
| C | -3.317394 | -0.126439 | 0.541858 |
| C | -2.291812 | -0.126954 | 1.500028 |
| C | -4.265572 | 0.910550 | 0.561193 |
| C | -2.221561 | 0.885448 | 2.457711 |
| H | -1.566863 | -0.933433 | 1.509744 |
| C | -4.196425 | 1.916555 | 1.522562 |
| H | -5.073149 | 0.919988 | -0.166102 |
| C | -3.170839 | 1.908355 | 2.470828 |
| H | -1.431157 | 0.863434 | 3.202474 |
| H | -4.940073 | 2.708398 | 1.528800 |
| H | -3.115838 | 2.694053 | 3.219085 |
| C | 0.048951 | -3.371612 | 2.192987 |
| C | 0.584248 | -3.132553 | 3.461018 |
| C | -0.249668 | -3.051142 | 4.577906 |
| C | -1.627099 | -3.219861 | 4.414420 |
| C | -2.164528 | -3.464221 | 3.151002 |
| C | -1.336250 | -3.538474 | 2.016057 |
| H | 0.707384 | -3.440463 | 1.332244 |
| H | 1.659482 | -3.014628 | 3.572734 |
| H | 0.168498 | -2.866086 | 5.563916 |
| H | -2.288288 | -3.165775 | 5.276437 |
| H | -3.235108 | -3.602321 | 3.030792 |
| S | -2.032592 | -3.860147 | 0.401572 |
| H | -2.757723 | -2.325869 | -0.021430 |
| H | 1.792424 | -1.581426 | -1.507944 |
| H | 0.737918 | -3.799594 | -1.170019 |
| H | 2.080899 | -5.870003 | -1.420476 |
| C | 4.095607 | 0.951921 | 2.482460 |
| C | -0.468718 | 3.529139 | -0.174116 |
| C | -0.476303 | 4.810590 | 0.391406 |
| C | -1.489499 | 3.148184 | -1.056318 |
| C | -1.509470 | 5.672096 | 0.044360 |
| H | 0.294697 | 5.137805 | 1.073607 |
| C | -2.504402 | 4.044135 | -1.340660 |
| H | -1.478237 | 2.165000 | -1.507160 |
| C | -2.550033 | 5.327107 | -0.814602 |
| H | -3.344814 | 6.022232 | -1.054815 |
| F | -1.520512 | 6.904881 | 0.587397 |
| F | -3.494137 | 3.640289 | -2.180048 |
| C | 3.561791 | 2.169602 | 3.266087 |
| H | 2.473513 | 2.263896 | 3.189814 |
| H | 3.809943 | 2.052694 | 4.327621 |
| H | 4.002906 | 3.106798 | 2.912912 |
| C | 5.622107 | 0.881023 | 2.701985 |
| H | 6.058729 | 0.008680 | 2.215074 |
| H | 6.114534 | 1.783451 | 2.316465 |
| H | 5.836745 | 0.825938 | 3.775973 |

| | | | |
|---|---|---|---|
| C | 3.419191 | -0.320977 | 3.031397 |
| H | 3.622834 | -0.412062 | 4.105022 |
| H | 2.326828 | -0.282256 | 2.921920 |
| H | 3.789556 | -1.217585 | 2.531252 |

TS Conformation 154

B3LYP/6-31G(d) Energy = -3411.863142
M06-2X/def2tzvpp/IEFPCM(chloroform) Energy = -3411.877917
M06-2X/def2tzvpp/IEFPCM(chloroform)//B3LYP/6-31G(d) Quasiharmonic Free Energy = -3411.152847

Frequencies (Top 3 out of 291)

1. -781.2749 cm-1
2. 10.2836 cm-1
3. 11.4212 cm-1

B3LYP/6-31G(d) Molecular Geometry in Cartesian Coordinates

| | | | |
|---|---|---|---|
| C | 4.049680 | 0.529115 | 0.979762 |
| C | 2.825469 | 0.284997 | 1.902162 |
| H | 3.110147 | 0.830832 | 2.806143 |
| C | 1.210843 | 2.154390 | 1.524856 |
| N | -0.146522 | 2.332635 | 1.294677 |
| H | -0.702840 | 1.483782 | 1.372062 |
| N | 1.551008 | 0.839526 | 1.453450 |
| H | 0.836131 | 0.183526 | 1.157356 |
| C | 5.185027 | 0.941651 | -1.128190 |
| C | 2.736308 | 0.798751 | -1.214082 |
| C | 4.652984 | 1.549806 | -2.448949 |
| H | 5.746846 | 1.682736 | -0.553344 |
| C | 3.293097 | 0.867666 | -2.642945 |
| H | 2.031933 | -0.025991 | -1.083865 |
| H | 2.227837 | 1.737290 | -0.960119 |
| H | 5.342616 | 1.390983 | -3.281975 |
| H | 4.514844 | 2.630295 | -2.321496 |
| H | 3.424959 | -0.140052 | -3.052168 |
| H | 2.628054 | 1.418395 | -3.317001 |
| N | 3.942432 | 0.626416 | -0.384265 |
| O | 5.151309 | 0.576294 | 1.521131 |
| S | 2.319157 | 3.365908 | 1.862980 |
| C | 6.095103 | -0.261119 | -1.346878 |
| C | 5.599151 | -1.547414 | -1.592037 |
| C | 7.482108 | -0.067741 | -1.365394 |
| C | 6.465621 | -2.610404 | -1.854307 |
| H | 4.528097 | -1.725965 | -1.562690 |
| C | 8.351433 | -1.125703 | -1.630054 |
| C | 7.845282 | -2.403128 | -1.877517 |
| H | 6.060228 | -3.602557 | -2.036344 |
| O | -1.522908 | -0.260617 | 0.950166 |
| O | -0.360329 | -1.153788 | -1.605268 |
| C | -2.595035 | -0.745992 | 0.582584 |
| C | -3.027417 | -0.944618 | -0.829587 |
| O | -3.531705 | -1.191962 | 1.415089 |
| C | -3.196675 | -1.229139 | 2.817830 |
| H | -4.108673 | -1.553228 | 3.317070 |
| H | -2.892541 | -0.240649 | 3.170171 |
| H | -2.393719 | -1.951560 | 2.980268 |
| S | -1.662760 | -0.538141 | -1.935441 |
| C | -2.237579 | -1.124782 | -3.540230 |
| H | -2.361942 | -2.206938 | -3.421875 |

| | | | |
|---|---|---|---|
| H | -1.445602 | -0.897668 | -4.257041 |
| H | -3.179236 | -0.643320 | -3.807284 |
| C | -1.454861 | 1.250354 | -2.168388 |
| H | -0.723073 | 1.386713 | -2.968524 |
| H | -1.069486 | 1.644066 | -1.226327 |
| H | -2.415713 | 1.701660 | -2.420216 |
| C | -4.339193 | -0.295389 | -1.225576 |
| C | -5.310509 | -1.012135 | -1.941614 |
| C | -4.617327 | 1.031197 | -0.851273 |
| C | -6.519376 | -0.404296 | -2.286462 |
| H | -5.135092 | -2.055386 | -2.185140 |
| C | -5.827104 | 1.633870 | -1.189618 |
| H | -3.889455 | 1.598428 | -0.279028 |
| C | -6.779851 | 0.916492 | -1.916699 |
| H | -7.265483 | -0.973499 | -2.833885 |
| H | -6.016913 | 2.655444 | -0.873520 |
| H | -7.725435 | 1.380844 | -2.182734 |
| C | -3.521635 | -5.313434 | 0.948360 |
| C | -3.058840 | -5.744949 | 2.190821 |
| C | -1.792764 | -5.367446 | 2.646553 |
| C | -0.990772 | -4.561294 | 1.835772 |
| C | -1.445230 | -4.136799 | 0.584389 |
| C | -2.724222 | -4.499177 | 0.123818 |
| H | -4.510574 | -5.600497 | 0.603420 |
| H | -3.692634 | -6.377643 | 2.808205 |
| H | -1.432862 | -5.705347 | 3.614890 |
| H | 0.003634 | -4.272833 | 2.168953 |
| H | -0.801611 | -3.534839 | -0.051086 |
| S | -3.339015 | -3.947802 | -1.456493 |
| H | -3.143140 | -2.264406 | -1.075190 |
| H | 7.884608 | 0.921333 | -1.157832 |
| H | 9.424830 | -0.954224 | -1.632075 |
| H | 8.520442 | -3.230873 | -2.077662 |
| C | 2.648426 | -1.225316 | 2.303713 |
| C | -0.915843 | 3.478713 | 1.054371 |
| C | -2.311275 | 3.314035 | 1.173624 |
| C | -0.403902 | 4.716046 | 0.634686 |
| C | -3.150922 | 4.366885 | 0.846793 |
| H | -2.736012 | 2.385715 | 1.539049 |
| C | -1.301012 | 5.737146 | 0.347107 |
| H | 0.658935 | 4.888487 | 0.551470 |
| C | -2.684003 | 5.605185 | 0.427724 |
| H | -3.352531 | 6.421606 | 0.185334 |
| F | -4.487344 | 4.166200 | 0.928724 |
| F | -0.802553 | 6.920425 | -0.057759 |
| C | 3.947103 | -1.745398 | 2.953742 |
| H | 4.228049 | -1.138263 | 3.820874 |
| H | 3.791291 | -2.776082 | 3.294167 |
| H | 4.790296 | -1.732868 | 2.260739 |
| C | 2.312152 | -2.109486 | 1.085497 |
| H | 2.208136 | -3.153839 | 1.402106 |
| H | 1.370554 | -1.831116 | 0.596300 |
| H | 3.110064 | -2.072566 | 0.336728 |
| C | 1.516472 | -1.330989 | 3.350381 |
| H | 1.709463 | -0.674562 | 4.207179 |
| H | 1.455590 | -2.359471 | 3.724588 |
| H | 0.533109 | -1.075329 | 2.941476 |

TS Conformation 155

B3LYP/6-31G(d) Energy = -3411.868086

M06-2X/def2tzvpp/IEFPCM(chloroform) Energy = -3411.877717
M06-2X/def2tzvpp/IEFPCM(chloroform)//B3LYP/6-31G(d) Quasiharmonic Free Energy = -3411.151735

Frequencies (Top 3 out of 291)

1. -806.4062 cm-1
2. 10.9488 cm-1
3. 14.0223 cm-1

B3LYP/6-31G(d) Molecular Geometry in Cartesian Coordinates

| | | | |
|---|---|---|---|
| C | 3.877235 | 1.093545 | 0.297498 |
| C | 3.027317 | 1.977073 | 1.257653 |
| H | 3.352504 | 3.014788 | 1.118710 |
| C | 0.804338 | 3.042523 | 0.732968 |
| N | -0.522565 | 2.666459 | 0.664433 |
| H | -0.709984 | 1.694414 | 0.908410 |
| N | 1.620353 | 1.956793 | 0.853262 |
| H | 1.189504 | 1.040258 | 0.778917 |
| C | 4.571695 | 0.465296 | -1.957850 |
| C | 2.980760 | 2.322025 | -1.751156 |
| C | 4.273808 | 1.087346 | -3.342595 |
| H | 5.606996 | 0.680723 | -1.664473 |
| C | 3.820196 | 2.515473 | -3.020292 |
| H | 1.954551 | 2.021105 | -2.001842 |
| H | 2.922706 | 3.223193 | -1.138258 |
| H | 3.461699 | 0.530782 | -3.825644 |
| H | 5.146442 | 1.035909 | -4.000398 |
| H | 3.250766 | 2.985136 | -3.828755 |
| H | 4.684941 | 3.154062 | -2.802537 |
| N | 3.676894 | 1.228245 | -1.049940 |
| O | 4.717430 | 0.301285 | 0.718320 |
| S | 1.362715 | 4.631180 | 0.686851 |
| C | 4.384596 | -1.041663 | -1.927681 |
| C | 3.113890 | -1.625510 | -1.853483 |
| C | 5.501837 | -1.874632 | -2.046249 |
| C | 2.964801 | -3.013145 | -1.889259 |
| H | 2.236937 | -0.992869 | -1.743989 |
| C | 5.356775 | -3.261852 | -2.095439 |
| C | 4.087395 | -3.835226 | -2.015038 |
| H | 1.973943 | -3.453972 | -1.804572 |
| O | -1.301194 | -0.121766 | 1.291214 |
| O | -0.090993 | -0.147837 | -1.327936 |
| C | -2.033606 | -1.055048 | 0.974142 |
| C | -2.322038 | -1.556415 | -0.402667 |
| O | -2.661906 | -1.828966 | 1.860182 |
| C | -2.335541 | -1.611777 | 3.249625 |
| H | -2.968776 | -2.307038 | 3.798870 |
| H | -2.549406 | -0.579756 | 3.537309 |
| H | -1.280727 | -1.838840 | 3.417346 |
| S | -1.462321 | -0.591315 | -1.659538 |
| C | -1.430041 | -1.692566 | -3.089307 |
| H | -0.835544 | -2.559431 | -2.782100 |
| H | -0.940885 | -1.142336 | -3.895903 |
| H | -2.446095 | -1.987005 | -3.356696 |
| C | -2.441821 | 0.817091 | -2.254139 |
| H | -2.510151 | 1.537025 | -1.435573 |
| H | -3.432519 | 0.471988 | -2.552448 |
| H | -1.891394 | 1.244624 | -3.095981 |
| C | -3.775926 | -1.800428 | -0.753884 |
| C | -4.165789 | -3.003944 | -1.361935 |
| C | -4.761351 | -0.838820 | -0.460936 |

| | | | |
|---|---|---|---|
| C | -5.504848 | -3.230573 | -1.686064 |
| H | -3.424203 | -3.778388 | -1.533878 |
| C | -6.097318 | -1.070654 | -0.782953 |
| H | -4.494081 | 0.093236 | 0.031741 |
| C | -6.471534 | -2.265432 | -1.403288 |
| H | -5.791276 | -4.171034 | -2.148442 |
| H | -6.844371 | -0.318968 | -0.544160 |
| H | -7.513359 | -2.445747 | -1.653486 |
| C | -0.756253 | -5.250543 | 1.907502 |
| C | -0.272248 | -5.302375 | 3.214612 |
| C | 0.728036 | -4.421684 | 3.634975 |
| C | 1.243127 | -3.491447 | 2.729243 |
| C | 0.763744 | -3.439764 | 1.417622 |
| C | -0.247407 | -4.316717 | 0.987615 |
| H | -1.534623 | -5.935651 | 1.584775 |
| H | -0.677527 | -6.036585 | 3.907379 |
| H | 1.108938 | -4.467119 | 4.651988 |
| H | 2.034870 | -2.811730 | 3.034465 |
| H | 1.190355 | -2.730349 | 0.714747 |
| S | -0.875757 | -4.271208 | -0.685215 |
| H | -1.671387 | -2.735059 | -0.512798 |
| H | 6.495517 | -1.433496 | -2.087802 |
| H | 6.237517 | -3.893265 | -2.182360 |
| H | 3.971092 | -4.915498 | -2.038116 |
| C | 3.208579 | 1.632001 | 2.782000 |
| C | -1.691827 | 3.395619 | 0.412593 |
| C | -1.748973 | 4.627332 | -0.258421 |
| C | -2.893629 | 2.770959 | 0.810195 |
| C | -2.996360 | 5.189739 | -0.500144 |
| H | -0.852784 | 5.143368 | -0.568786 |
| C | -4.104723 | 3.376329 | 0.515754 |
| H | -2.876968 | 1.840382 | 1.365061 |
| C | -4.202706 | 4.595775 | -0.140950 |
| H | -5.156612 | 5.060871 | -0.355591 |
| F | -3.042490 | 6.370181 | -1.146647 |
| F | -5.239332 | 2.733133 | 0.876576 |
| C | 4.668272 | 1.906775 | 3.203914 |
| H | 4.945069 | 2.948248 | 2.993366 |
| H | 4.772873 | 1.753872 | 4.284982 |
| H | 5.367007 | 1.248153 | 2.688609 |
| C | 2.828821 | 0.167933 | 3.083210 |
| H | 3.472068 | -0.528401 | 2.541406 |
| H | 2.932165 | -0.027125 | 4.157562 |
| H | 1.782315 | -0.039048 | 2.821295 |
| C | 2.299916 | 2.556775 | 3.620285 |
| H | 2.507521 | 3.614585 | 3.430327 |
| H | 1.236721 | 2.382986 | 3.424526 |
| H | 2.473191 | 2.362127 | 4.685261 |

TS Conformation 156

B3LYP/6-31G(d) Energy = -3411.868086
M06-2X/def2tzvpp/IEFPCM(chloroform) Energy = -3411.877716
M06-2X/def2tzvpp/IEFPCM(chloroform)//B3LYP/6-31G(d) Quasiharmonic Free Energy = -3411.151734

Frequencies (Top 3 out of 291)

1. -806.3580 cm-1
2. 10.9503 cm-1
3. 14.0223 cm-1

B3LYP/6-31G(d) Molecular Geometry in Cartesian Coordinates

| | | | |
|---|---|---|---|
| C | 3.877319 | 1.093415 | 0.297325 |
| C | 3.027480 | 1.976938 | 1.257555 |
| H | 3.352693 | 3.014647 | 1.118634 |
| C | 0.804511 | 3.042477 | 0.733000 |
| N | -0.522405 | 2.666456 | 0.664490 |
| H | -0.709841 | 1.694402 | 0.908416 |
| N | 1.620494 | 1.956717 | 0.853237 |
| H | 1.189609 | 1.040197 | 0.778905 |
| C | 4.571605 | 0.465177 | -1.958078 |
| C | 2.980748 | 2.321958 | -1.751248 |
| C | 4.273656 | 1.087266 | -3.342791 |
| H | 5.606930 | 0.680560 | -1.664760 |
| C | 3.820113 | 2.515402 | -3.020431 |
| H | 1.954513 | 2.021080 | -2.001877 |
| H | 2.922765 | 3.223115 | -1.138327 |
| H | 3.461498 | 0.530742 | -3.825804 |
| H | 5.146248 | 1.035813 | -4.000648 |
| H | 3.250649 | 2.985102 | -3.828849 |
| H | 4.684894 | 3.153957 | -2.802716 |
| N | 3.676885 | 1.228138 | -1.050097 |
| O | 4.717531 | 0.301134 | 0.718077 |
| S | 1.362938 | 4.631117 | 0.686912 |
| C | 4.384450 | -1.041776 | -1.927923 |
| C | 3.113720 | -1.625574 | -1.853724 |
| C | 5.501657 | -1.874789 | -2.046504 |
| C | 2.964577 | -3.013203 | -1.889513 |
| H | 2.236791 | -0.992902 | -1.744222 |
| C | 5.356540 | -3.262002 | -2.095705 |
| C | 4.087138 | -3.835327 | -2.015303 |
| H | 1.973702 | -3.453992 | -1.804827 |
| O | -1.300986 | -0.121775 | 1.291198 |
| O | -0.091109 | -0.147812 | -1.328093 |
| C | -2.033529 | -1.054974 | 0.974184 |
| C | -2.322119 | -1.556311 | -0.402601 |
| O | -2.661834 | -1.828828 | 1.860276 |
| C | -2.335317 | -1.611684 | 3.249691 |
| H | -2.968541 | -2.306915 | 3.798986 |
| H | -2.549090 | -0.579655 | 3.537413 |
| H | -1.280501 | -1.838814 | 3.417306 |
| S | -1.462484 | -0.591242 | -1.659555 |
| C | -1.430387 | -1.692498 | -3.089326 |
| H | -0.941262 | -1.142297 | -3.895961 |
| H | -2.446481 | -1.986874 | -3.356633 |
| H | -0.835916 | -2.559397 | -2.782166 |
| C | -2.441988 | 0.817201 | -2.254057 |
| H | -3.432759 | 0.472157 | -2.552189 |
| H | -1.891672 | 1.244664 | -3.096008 |
| H | -2.510132 | 1.537171 | -1.435507 |
| C | -3.776049 | -1.800276 | -0.753681 |
| C | -4.166009 | -3.003784 | -1.361685 |
| C | -4.761414 | -0.838633 | -0.460647 |
| C | -5.505105 | -3.230371 | -1.685686 |
| H | -3.424463 | -3.778253 | -1.533691 |
| C | -6.097419 | -1.070425 | -0.782536 |
| H | -4.494068 | 0.093417 | 0.031999 |
| C | -6.471733 | -2.265196 | -1.402827 |
| H | -5.791608 | -4.170826 | -2.148029 |
| H | -6.844425 | -0.318713 | -0.543679 |
| H | -7.513588 | -2.445479 | -1.652925 |
| C | 0.763770 | -3.439896 | 1.417462 |

| | | | |
|---|---|---|---|
| C | 1.243205 | -3.491615 | 2.729063 |
| C | 0.728013 | -4.421748 | 3.634845 |
| C | -0.272424 | -5.302298 | 3.214552 |
| C | -0.756479 | -5.250430 | 1.907463 |
| C | -0.247532 | -4.316708 | 0.987526 |
| H | 1.190456 | -2.730565 | 0.714548 |
| H | 2.035063 | -2.812007 | 3.034230 |
| H | 1.108953 | -4.467210 | 4.651842 |
| H | -0.677783 | -6.036427 | 3.907359 |
| H | -1.534967 | -5.935430 | 1.584790 |
| S | -0.875951 | -4.271154 | -0.685279 |
| H | -1.671522 | -2.734969 | -0.512797 |
| H | 6.495354 | -1.433690 | -2.088057 |
| H | 6.237257 | -3.893449 | -2.182635 |
| H | 3.970792 | -4.915594 | -2.038390 |
| C | 3.208806 | 1.631805 | 2.781881 |
| C | -1.691654 | 3.395661 | 0.412730 |
| C | -1.748794 | 4.627402 | -0.258233 |
| C | -2.893457 | 2.771022 | 0.810363 |
| C | -2.996175 | 5.189855 | -0.499879 |
| H | -0.852603 | 5.143422 | -0.568618 |
| C | -4.104546 | 3.376438 | 0.515999 |
| H | -2.876797 | 1.840425 | 1.365196 |
| C | -4.202522 | 4.595911 | -0.140655 |
| H | -5.156424 | 5.061043 | -0.355236 |
| F | -3.042301 | 6.370322 | -1.146334 |
| F | -5.239158 | 2.733259 | 0.876847 |
| C | 2.300200 | 2.556566 | 3.620240 |
| H | 1.236992 | 2.382789 | 3.424541 |
| H | 2.473537 | 2.361895 | 4.685202 |
| H | 2.507801 | 3.614379 | 3.430292 |
| C | 4.668523 | 1.906535 | 3.203736 |
| H | 4.945320 | 2.948020 | 2.993244 |
| H | 4.773179 | 1.753559 | 4.284789 |
| H | 5.367225 | 1.247941 | 2.688351 |
| C | 2.829035 | 0.167733 | 3.083054 |
| H | 1.782519 | -0.039227 | 2.821163 |
| H | 3.472256 | -0.528593 | 2.541208 |
| H | 2.932408 | -0.027362 | 4.157397 |

TS Conformation 157

B3LYP/6-31G(d) Energy = -3411.860035
M06-2X/def2tzvpp/IEFPCM(chloroform) Energy = -3411.873459
M06-2X/def2tzvpp/IEFPCM(chloroform)//B3LYP/6-31G(d) Quasiharmonic Free Energy = -3411.148439

Frequencies (Top 3 out of 291)

1. -784.9007 cm-1
2. 7.3794 cm-1
3. 10.5730 cm-1

B3LYP/6-31G(d) Molecular Geometry in Cartesian Coordinates

| | | | |
|---|---|---|---|
| C | -4.512583 | 0.784651 | 0.539442 |
| C | -3.245383 | 0.210859 | 1.232460 |
| H | -3.116784 | -0.789756 | 0.799955 |
| C | -1.713598 | 2.232955 | 0.752487 |
| N | -0.340731 | 2.435601 | 0.787021 |
| H | 0.203835 | 1.618873 | 1.051654 |
| N | -1.993950 | 0.898417 | 0.859749 |

| | | | |
|---|---|---|---|
| H | -1.190942 | 0.276870 | 0.849281 |
| C | -3.589751 | 0.041922 | -1.726028 |
| C | -5.715411 | 1.256334 | -1.529535 |
| C | -3.982479 | 0.660872 | -3.092788 |
| H | -2.582175 | 0.365380 | -1.451684 |
| C | -5.504576 | 0.836371 | -2.990167 |
| H | -6.660828 | 0.907140 | -1.104597 |
| H | -5.681114 | 2.345184 | -1.397867 |
| H | -3.668151 | 0.034993 | -3.932642 |
| H | -3.497549 | 1.639472 | -3.190648 |
| H | -6.008724 | -0.115402 | -3.192467 |
| H | -5.895786 | 1.576494 | -3.694410 |
| N | -4.577269 | 0.648425 | -0.819101 |
| O | -5.461307 | 1.248270 | 1.164647 |
| S | -2.857295 | 3.442681 | 0.590752 |
| C | -3.617884 | -1.486313 | -1.733721 |
| C | -4.763891 | -2.215935 | -1.392146 |
| C | -2.466376 | -2.186858 | -2.121156 |
| C | -4.762778 | -3.611561 | -1.448809 |
| H | -5.655597 | -1.690991 | -1.062075 |
| C | -2.463358 | -3.581291 | -2.177225 |
| C | -3.614239 | -4.298992 | -1.842858 |
| H | -5.660926 | -4.160756 | -1.178024 |
| O | 1.139236 | -0.086912 | 0.792817 |
| O | 0.818288 | -1.038610 | -2.008459 |
| C | 2.238174 | -0.643118 | 0.794900 |
| C | 3.084800 | -0.908407 | -0.401432 |
| O | 2.829660 | -1.120754 | 1.887262 |
| C | 2.062966 | -1.093853 | 3.108391 |
| H | 2.724647 | -1.510819 | 3.866230 |
| H | 1.781484 | -0.069396 | 3.365129 |
| H | 1.170262 | -1.712571 | 2.995756 |
| S | 2.179896 | -0.478490 | -1.897946 |
| C | 2.128052 | 1.307040 | -2.220017 |
| H | 3.138007 | 1.717633 | -2.182449 |
| H | 1.496818 | 1.745315 | -1.445608 |
| H | 1.675446 | 1.439001 | -3.205991 |
| C | 3.230362 | -1.131675 | -3.209575 |
| H | 4.224935 | -0.686617 | -3.161573 |
| H | 2.728180 | -0.906263 | -4.152811 |
| H | 3.268185 | -2.212527 | -3.033016 |
| C | 4.492454 | -0.345994 | -0.366467 |
| C | 5.596508 | -1.160055 | -0.662960 |
| C | 4.717478 | 0.995455 | -0.009434 |
| C | 6.889141 | -0.634165 | -0.619379 |
| H | 5.441794 | -2.211126 | -0.885538 |
| C | 6.008486 | 1.518143 | 0.035107 |
| H | 3.881524 | 1.639102 | 0.247713 |
| C | 7.098849 | 0.702956 | -0.276899 |
| H | 7.735182 | -1.277929 | -0.843304 |
| H | 6.150810 | 2.556771 | 0.318992 |
| H | 8.107582 | 1.105207 | -0.242171 |
| C | 0.746800 | -3.879993 | 0.222105 |
| C | -0.242682 | -4.172522 | 1.164701 |
| C | 0.062041 | -4.924550 | 2.301465 |
| C | 1.370342 | -5.382196 | 2.484776 |
| C | 2.361891 | -5.086196 | 1.549929 |
| C | 2.068035 | -4.328094 | 0.401558 |
| H | 0.492618 | -3.309946 | -0.666057 |
| H | -1.260143 | -3.829041 | 0.991451 |
| H | -0.710512 | -5.161052 | 3.028566 |
| H | 1.621183 | -5.974277 | 3.362207 |

| | | | |
|---|---|---|---|
| H | 3.378065 | -5.438907 | 1.700020 |
| S | 3.350296 | -3.954046 | -0.782635 |
| H | 3.187635 | -2.244407 | -0.561069 |
| H | -1.559904 | -1.639035 | -2.367397 |
| H | -1.557871 | -4.106806 | -2.468296 |
| H | -3.610955 | -5.384959 | -1.879862 |
| C | -3.381098 | -0.021046 | 2.777656 |
| C | 0.462147 | 3.553177 | 0.529908 |
| C | 0.052687 | 4.712692 | -0.146924 |
| C | 1.806886 | 3.436822 | 0.943832 |
| C | 0.996128 | 5.704629 | -0.385604 |
| H | -0.970997 | 4.847828 | -0.462550 |
| C | 2.699982 | 4.455253 | 0.655277 |
| H | 2.144196 | 2.574372 | 1.507787 |
| C | 2.333949 | 5.616191 | -0.012611 |
| H | 3.041236 | 6.408523 | -0.222873 |
| F | 0.596116 | 6.811888 | -1.039114 |
| F | 3.991114 | 4.297671 | 1.034244 |
| C | -3.564869 | 1.286566 | 3.569523 |
| H | -3.643084 | 1.058090 | 4.640242 |
| H | -2.711500 | 1.960157 | 3.436608 |
| H | -4.466443 | 1.810955 | 3.251622 |
| C | -2.099555 | -0.728895 | 3.271894 |
| H | -2.204438 | -0.985004 | 4.332261 |
| H | -1.906576 | -1.661553 | 2.724437 |
| H | -1.214339 | -0.089568 | 3.177388 |
| C | -4.573077 | -0.968682 | 3.032991 |
| H | -5.518460 | -0.497712 | 2.759689 |
| H | -4.469666 | -1.902164 | 2.463394 |
| H | -4.612345 | -1.235164 | 4.095884 |

## 9. References


1. Knowles, R. R.; Lin, S.; Jacobsen, E. N*. J. Am. Chem. Soc.* **2010**, *132*, 5030-5032.
2. Banik, S. M.; Levina, A.; Hyde, A. M.; Jacobsen, E. N.; *Science* **2017**, *358*, 761-764.
3. Schafer, A. G.; Wieting, J. M.; Fisher, T. J.; Mattson, A. E. *Angew. Chem. Int. Ed.* **2013**, *52*, 11321 – 11324.
4. (a)Sorrentino, E.; Connon, S. J. *Org. Lett.*, **2016**, *18*, 5204 – 5207. (b) Vakulya, B.; Varga, S.; Csámpai, A.; Soós, T. *Org. Lett.* **2005**, *7*, 1967-1969.
5. Dias, R. M. P.; Burtoloso, A. C. B. *Org. Lett*. **2016**, *18*, 3034–3037.
6. Janot, C.; Palamini, P.; Dobson, B. C.; Muir, J.; Aissa, C., *Org. Lett.* **2019,** *211,* 296-299.
7. La, M. T.; Kim, H. K., *Tetrahedron,* **2018**, *74*, 3748-3754.
8. (a) Tortoreto, C.; Rackl, D.; Davies, H. M. L. *Org. Lett.* **2017**, 19, 770-773. (b)Talero, A. G. New methodologies in synthesis using α-β-unsaturated diazoketones and β-ketosulfoxonium ylides. **2019**. Doctoral Thesis. University of Sao Paulo. Available at: https://teses.usp.br/teses/disponiveis/75/75133/tde-15102019-161419/en.php, https://doi.org/10.11606/T.75.2019.tde-15102019-161419 (Accessed: 13 of March 2020).
9. Nakamura, S.; Nakagawa, R.; Watanabe, Y.; Toru, T., *J. Am. Chem. Soc.* **2000,** *122*, 46, 11340-11347.
10. Keipour, H.; Jalba, A.; Tanbouza, N.; Carreras, V.; Ollevier, T., *Org. Biomol. Chem.* **2019**, *17*, 3098-3102.
11. Keipour, H.; Jalba, A.; Delage-Laurin, L.; Ollevier, T., *J. Org. Chem.* **2017**, *82*, 3000-3010.
12. Mohamadi, F.; Richards, N. G. J.; Guida, W. C.; Liskamp, R.; Lipton, M.; Caufield, C.; Chang, G.; Hendrickson, T.; Still, W. C. *J. Comput. Chem.* **1990**, *11*, 440–467.
13. Macromodel, Version 11.6. Schrödinger, LLC: New York, NY **2019**.
14. Banks, J. L.; Beard, H. S.; Cao, Y.; Cho, A. E.; Damm, W.; Farid, R.; Felts, A. K.; Halgren, T. A.; Mainz, D. T.; Maple, J. R. *et al.*, *J. Comput. Chem.* **2005**, *26*, 1752–1780.

15. Chang, G.; Guida, W. C.; Still, W. C. *J. Am. Chem. Soc.* **1989**, *111*, 4379–4386.
16. Kolossvary, I.; Guida, W. C. *J. Comput. Chem.* **1999**, *20*, 1671–1684.
17. Frisch, M. J.; Trucks, G. W.; Schlegel, H. B.; Scuseria, G. E.; Robb, M. A.; Cheeseman, J. R.; Scalmani, G.; Barone, V.; Petersson, G. A. et al. Gaussian 16, Revision A.03, Gaussian Inc., Wallingford CT, **2016**.
18. Becke, A. D. *J. Chem. Phys.* **1993**, *98*, 5648–5652.
19. Stephens, P. J.; Devlin, F. J.; Chabalowski, C. F.; Frisch, M. J. *J. Phys. Chem.* **1994**, *98*, 11623–11627.
20. Hehre, W. J.; Ditchfield, R.; Pople, J. A. *J. Chem. Phys.* **1972**, *56*, 2257–2261.
21. Simón, L.; Goodman, J. M. *Org. Biomol. Chem.* **2011**, *9*, 689–700.
22. Zhao, Y.; Truhlar, D. G. *Theor. Chem. Acc.* **2008**, *120*, 215–241.
23. Weigend, F.; Ahlrichs, R. *Phys. Chem. Chem. Phys.* **2005**, *7*, 3297.
24. Mennucci, B.; Cammi, R.; Tomasi, J. *J. Chem. Phys.* **1998**, *109*, 2798–2807.
25. Grimme, S. *Chem. A Eur. J.* **2012**, *18*, 9955–9964.
26. Funes-Ardoiz, I.; Paton, R. S. **2018**, DOI 10.5281/zenodo.595246.
27. Alecu, I. M.; Zheng, J.; Zhao, Y.; Truhlar, D. G. *J. Chem. Theory Comput.* **2010**, *6*, 2872–2887.
28. Grayson, M. N.; Houk, K. N. *J. Am. Chem. Soc.* **2016**, *138*, 9041–9044.
29. Grayson, M. N. *J. Org. Chem.* **2017**, *82*, 4396–4401.
30. Pedregal, J. R.-G.; Gómez-Orellana, P.; Maréchal, J.-D. *J. Chem. Inf. Model.* **2018**, *58*, 561–564.
